# Fluorescence Amplification of Unsaturated Oxazolones Using Palladium: Photophysical and Computational Studies

**DOI:** 10.1021/acs.inorgchem.3c00601

**Published:** 2023-06-14

**Authors:** David Dalmau, Olga Crespo, Jon M. Matxain, Esteban P. Urriolabeitia

**Affiliations:** † Instituto de Síntesis Química y Catálisis Homogénea, ISQCH (CSIC-Universidad de Zaragoza), Pedro Cerbuna 12, 50009 Zaragoza, Spain; ‡ Polimero eta Material Aurreratuak: Fisika, Kimika eta Teknologia Saila, Kimika Fakultatea, Euskal Herriko Unibertsitatea UPV/EHU and Donostia International Physics Center (DIPC) PK 1072, 20080 Donostia, Euskadi, Spain

## Abstract

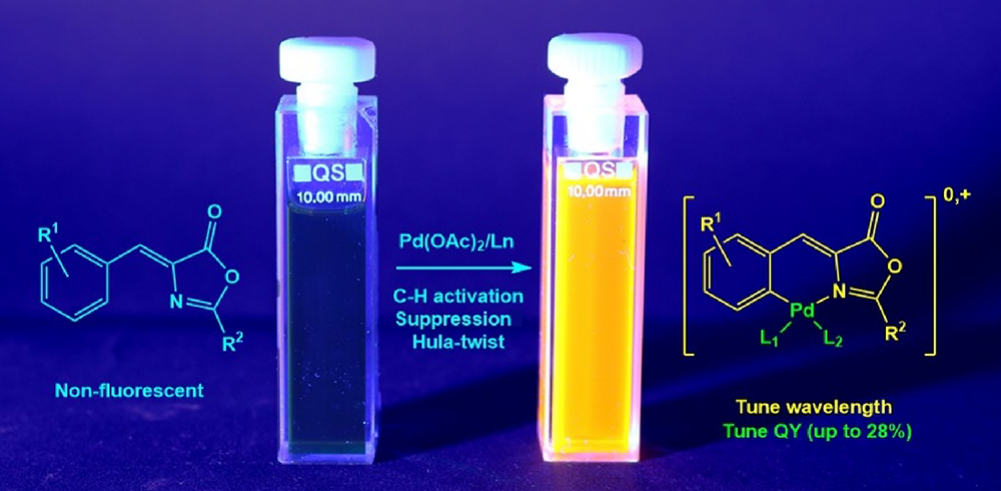

Weakly fluorescent (*Z*)-4-arylidene-5-(4*H*)-oxazolones (**1**), Φ_PL_ < 0.1%, containing a variety of conjugated aromatic fragments and/or charged arylidene moieties, have been orthopalladated by reaction with Pd(OAc)_2_. The resulting dinuclear complexes (**2**) have the oxazolone ligands bonded as a C^N-chelate, restricting intramolecular motions involving the oxazolone. From **2**, a variety of mononuclear derivatives, such as [Pd(C^N-oxazolone)(O_2_CCF_3_)(py)] (**3**), [Pd(C^N-oxazolone)(py)_2_](ClO_4_) (**4**), [Pd(C^N-oxazolone)(Cl)(py)] (**5**), and [Pd(C^N-oxazolone)(X)(NHC)] (**6**, **7**), have been prepared and fully characterized. Most of complexes **3**–**6** are strongly fluorescent in solution in the range of wavelengths from green to yellow, with values of Φ_PL_ up to 28% (**4h**), which are among the highest values of quantum yield ever reported for organometallic Pd complexes with bidentate ligands. This means that the introduction of the Pd in the oxazolone scaffold produces in some cases an amplification of the fluorescence of several orders of magnitude from the free ligand **1** to complexes **3**–**6**. Systematic variations of the substituents of the oxazolones and the ancillary ligands show that the wavelength of emission is tuned by the nature of the oxazolone, while the quantum yield is deeply influenced by the change of ligands. TD-DFT studies of complexes **3**–**6** show a direct correlation between the participation of the Pd orbitals in the HOMO and the loss of emission through non-radiative pathways. This model allows the understanding of the amplification of the fluorescence and the future rational design of new organopalladium systems with improved properties.

## Abstract 2

The incorporation of Pd to the molecular skeleton of non-fluorescent 4-aryliden-5(4*H*)-oxazolones through C−H bond activation promotes, in some cases, an amplification of the fluorescence of the oxazolone of several orders of magnitude. In the final orthopalladated compounds, the emission wavelength is tuned by the nature of the oxazolone, while modification of the photoluminescent quantum yield resides in the Pd center and the ancillary ligands. The amplification has been fully explained by TDDFT calculations.

## Introduction

**Figure 1:**
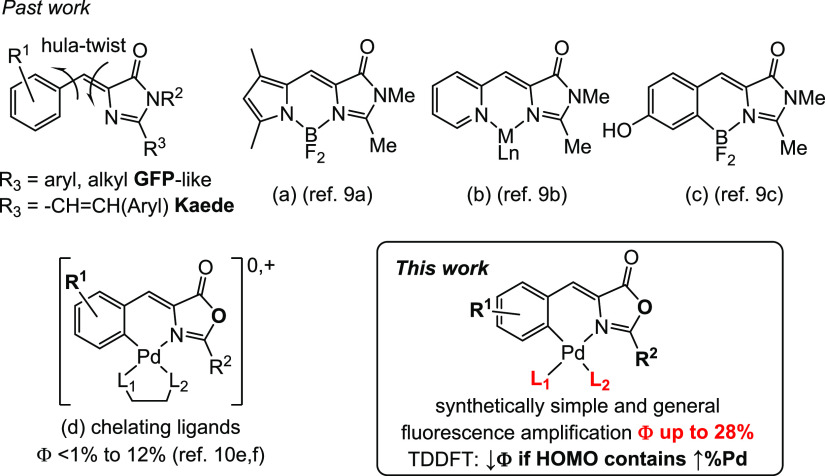
Molecular strategies based on intramolecular lock to increase fluorescence in GFP-like chromophores (L_2_ can be a neutral L_1_ fragment or an anionic X_1_ ligand).

The synthesis of new luminescent compounds and the study of their photophysical properties are research areas undergoing nowadays a strong development due to the deep social impact of their applications. The awareness of a more rational use of energetic resources and a simultaneous daily increase of the energy demand prompt this development. In this respect, luminescent-based technologies (chemoluminescence and fluorescent sensors) have been declared as “one of the top ten emerging technologies” in 2021 and 2022 by the IUPAC.^1^ Despite the increasing number of reported luminescent compounds, their “a priori” rational design searching for tunable properties is not a trivial task due to the high number of variables involved: charge separation, conformational restrictions, planarity, electronic delocalization, presence (or not) of metals, among others.^2^ Due to these facts, it is still not possible to make accurate predictions about luminescence and, in general, modular approaches are used.^3^ Therefore, gathering additional information for the building of new luminescent systems is highly desirable. Following models found in nature, we focused our attention on two intensely fluorescent proteins, green fluorescent protein (GFP) and Kaede protein, whose chromophores are small molecules containing the (*Z*)-4-arylidene-5(4*H*)-imidazolone skeleton (Figure 1).

The emissive properties of these chromophores have been studied in depth,^4^ and it has been determined that the rigid environment present in the inner part of the protein—the β-barrel, where the imidazolone is located—is the main responsible of the bright fluorescence observed. In the absence of a tight local environment, the isolated chromophores relax through different deactivation channels, such as internal motions (hula twist), excited-state proton transfer (ESPT), and/or isomerization, and the fluorescence in solution is almost completely lost.^5^ Typical values of luminescence quantum yields for such species are in the range 0.1–0.01%, depending on the substituents. Different solutions have been proposed to mitigate this loss. The immobilization of the imidazolone core in different confined media (MOF, COF, hosting proteins) allows a partial recovery of the fluorescence.^6^ Substrate engineering based on the choice of substituent positions, for instance the *meta-effect*, has also achieved success, though limited.^7^ The most promising strategies share the necessity of an intramolecular lock between the imidazolone and the arylidene fragments to increase the rigidity and decrease the number of degrees of freedom. As far as we know, only two strategies, namely, (i) the intramolecular constraint^8^ and (ii) the building of BODIPY-like structures using BF_2_ as bridge,^9^ have been successfully implemented as general methods for the recovery and amplification of the fluorescence in these molecules (Figure 1a–c).

However, these achievements are strictly limited to arylidene-imidazolones. (*Z*)-4-Aryliden-5(4*H*)-oxazolones are structurally related to imidazolones and show high fluorescence quantum yields (Φ) in the solid state. Despite this, their use as luminescent sources is scarcely represented due to the low quantum yields exhibited in solution (Φ < 0.1% in general).^10a−10d^ In clear contrast to the chemistry reported for imidazolones,^9^ there are no reports in the literature about the use of intramolecular locks to amplify the fluorescence of oxazolones in solution, except those coming from our group using Pd (Figure 1d).^10e−10g^ Our choice for the use of Pd as intramolecular lock is due to the promising previous results obtained, with increments of the quantum yield of one order of magnitude after Pd incorporation in some cases, despite the fact that Pd is not a metal often used for the building of luminescent compounds.^11^ In this respect, there are no orthometallated oxazolones with other transition metals typically used in luminescent complexes (Pt, Au, Ir, Ru, Re, for instance),^11a^ which remains an open field to discover. However, the increase of the fluorescence by Pd incorporation is not a general trend, showing that the intramolecular lock was not enough to promote by itself the amplification of the fluorescence. Due to these facts, and taking advantage of our knowledge about the orthopalladation of oxazolones, we aim to find a general method to amplify the luminescence of oxazolones in solution, using Pd as the intramolecular bridge between the oxazolone and arylidene rings. To achieve this task, we need to fully understand the reasons of the puzzling behavior shown by the Pd complexes, as well as the role of the Pd center and the ancillary ligands in the fluorescence.

In this contribution, we have prepared a collection of highly luminescent Pd complexes from orthometallated oxazolones following synthetically simple methods, avoiding the use of sophisticated ligands of high denticity. The coordination sphere of the Pd is completed with apparently innocent ancillary ligands, such as pyridine (py), chloride (Cl), *N*-heterocyclic carbene (NHC), and/or trifluoroacetate, providing a versatile system for fine-tuning of the photophysical properties. Despite its simplicity, the resulting complexes have structural environments, which result in intense emissive properties, achieving fluorescent quantum yields as high as 28% in solution at room temperature, which are similar to the highest values reported up to now in the bibliography for chelating systems.^11^ In addition, we have studied by TD-DFT these simple models, we have been able to determine the origin of the fluorescence and the orbitals involved, and, more importantly, we have established a correlation between the participation of the Pd orbitals in the absorption–emission process and the fluorescence intensity, fully explaining previous results and allowing us to make predictions for the design of improved systems. We present here the obtained results.

## Results and Discussion

### 1. Synthesis and Characterization of Oxazolones and Orthopalladated Derivatives

**Figure 2:**
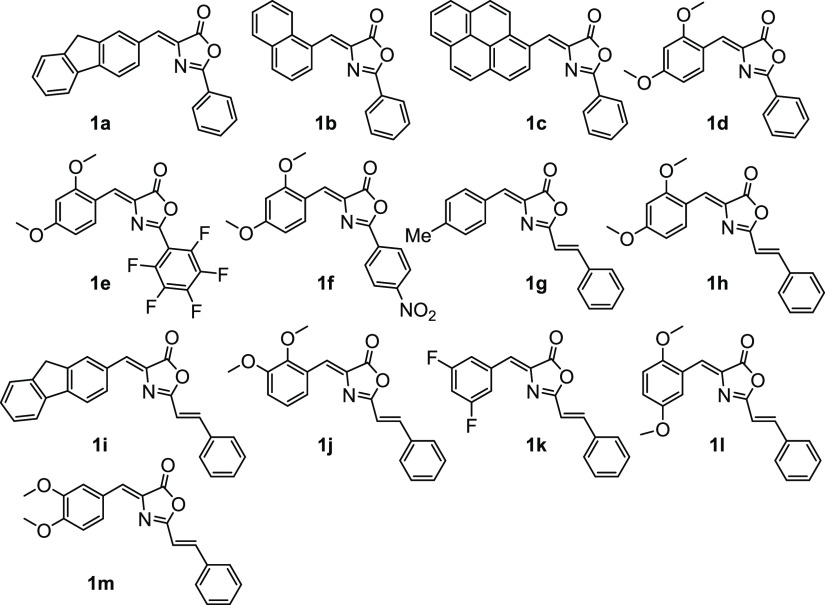
(*Z*)-4-Aryliden-5(4*H*)-oxazolones **1a**–**1m** used in this work.

The oxazolones **1a**–**1m** shown in Figure 2 have been prepared by the classical Erlenmeyer–Plöchl method,^12^ while the hippuric acids have been prepared by the Schotten–Baumann method.^13^ Oxazolones **1a**–**1d** and **1f**–**1h** appear on SciFinder,^14^ while **1e** and **1i**–**1m** are new species.

We have selected a variety of 4-arylidene fragments, taking into account facts directly involved on the luminescent properties of the oxazolones, which could increase their quantum yields,^10,15^ such as a highly conjugated electronic nature (**1a**, **1b**, **1c**, **1i**) or a push–pull character (**1d**–**1f**, **1h**, **1j**, **1l**, **1m**), aiming to increase intraligand (IL) charge transfer effects. The group in position 2 of the oxazolone either contains strong electroattracting moieties (**1e**, **1f**) or is able to extend the delocalization of the charge density (**1g**–**1m**). The molecular structures of **1a** and **1e** have been determined by X-ray diffraction methods (SI, Figures S1 and S2).

**Figure 3:**
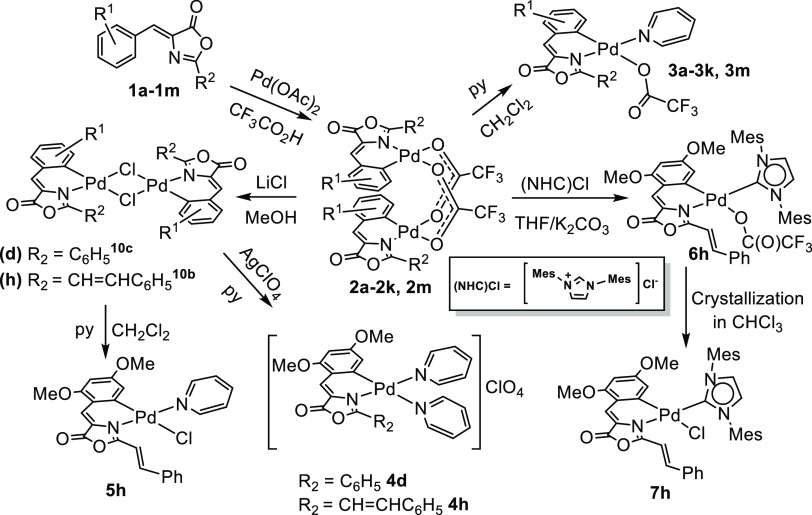
Orthopalladation of oxazolones **1** and reactivity of complexes **2**.

The orthopalladation of all oxazolones **1a**–**1m** has been attempted. The reaction works under standard conditions in all cases,^10,15,16^ except for **1l**, probably due to the steric hindrance of the methoxy group in 5-position. The corresponding dinuclear derivatives with carboxylate bridges **2a**–**2k** and **2m** were obtained as shown in Figure 3.

The yields of isolated compounds **2a**–**2k** and **2m** are in most of the cases higher than 90%, showing that the orthopalladation of oxazolones through C–H bond activation is a general process. Their characterization shows that they are obtained as dimers—as it is evident from the HRMS spectra—and that the relative arrangement of the orthometallated moieties is *transoid*, as inferred from the observance of a single peak in the ^19^F NMR spectra. Only in the case of **2b** were additional ^19^F signals due to the *cisoid* isomer observed. The selectivity in the position of the C–H activation is also remarkable, because only one position of the arylidene ring is activated in cases where more than one C–H bond is able to be activated (**1a**–**1c**, **1i**, **1m**).

Aiming to understand the role of each ligand in the luminescence of the complexes, we have studied the effect of the change of the oxazolone and the ancillary ligands on the luminescent properties of the resulting complexes. On the one hand, we have first screened different oxazolones **1a**–**1m** while keeping two simple ancillary ligands (pyridine and trifluoroacetate), completing the coordination sphere of the Pd. As shown in Figure 3, the dinuclear derivatives **2a**–**2k** and **2m** react in CH_2_Cl_2_ with pyridine (1:2 molar ratio) to give the highly soluble monomeric complexes **3a**–**3k** and **3m**, which can be isolated in good yields and show an intense luminescence. On the other hand, we have also examined the effect of the change of the ancillary ligands around the Pd center for the most emissive palladated oxazolone fragments (**3d** and **3h**). To prepare these new complexes, we have selected trifluoroacetate, chloride, pyridine, and NHC as ligands, aiming to cover a wide range of different *trans* influences. Treatment of **2h** with LiCl (1:4 molar ratio) in MeOH affords the known dinuclear chloride bridging complex shown in Figure 3,^10f^ which in turn reacts with pyridine (1:2 molar ratio) in CH_2_Cl_2_ to give mononuclear **5h**, or with AgClO_4_ and pyridine (1:2:4 molar ratio) in CH_2_Cl_2_/acetone to give the cationic bis-pyridine derivative **4h**. Complex **4d** has been prepared from **2d** following the same synthetic procedure. In addition, **2h** reacts with the bis(mesityl)imidazolium salt in the presence of K_2_CO_3_ in MeOH, as shown in Figure 3, to give the corresponding NHC-derivative **6h**. Complexes **4d**, **4h**, **5h**, and **6h** show similar or higher emissive quantum yields than **3d** and **3h** and allow emission energy tuning. The characterization of all prepared complexes is straightforward from their NMR and HRMS data, as shown in SI. In general, the orthopalladation takes place selectively in one position and the ligand arrangement follows the antisymbiotic effect shown by the soft Pd(II) center,^17^ with the pyridine ligand *cis* to the Pd–C bond.^18^ The X-ray crystal structures of complexes **3c**, **3d**, **3g**, and **7h** have been determined by diffraction methods and are discussed in detail in the SI (Figures S3–S7). Crystals of compound **7h** were obtained when complex **6h** was left to crystallize in CHCl_3_, probably due to the presence of residual HCl in the chlorinated solvent.

### 2.- Photophysical Studies of Orthopalladated Complexes **3**–**6**

**Figure 4:**
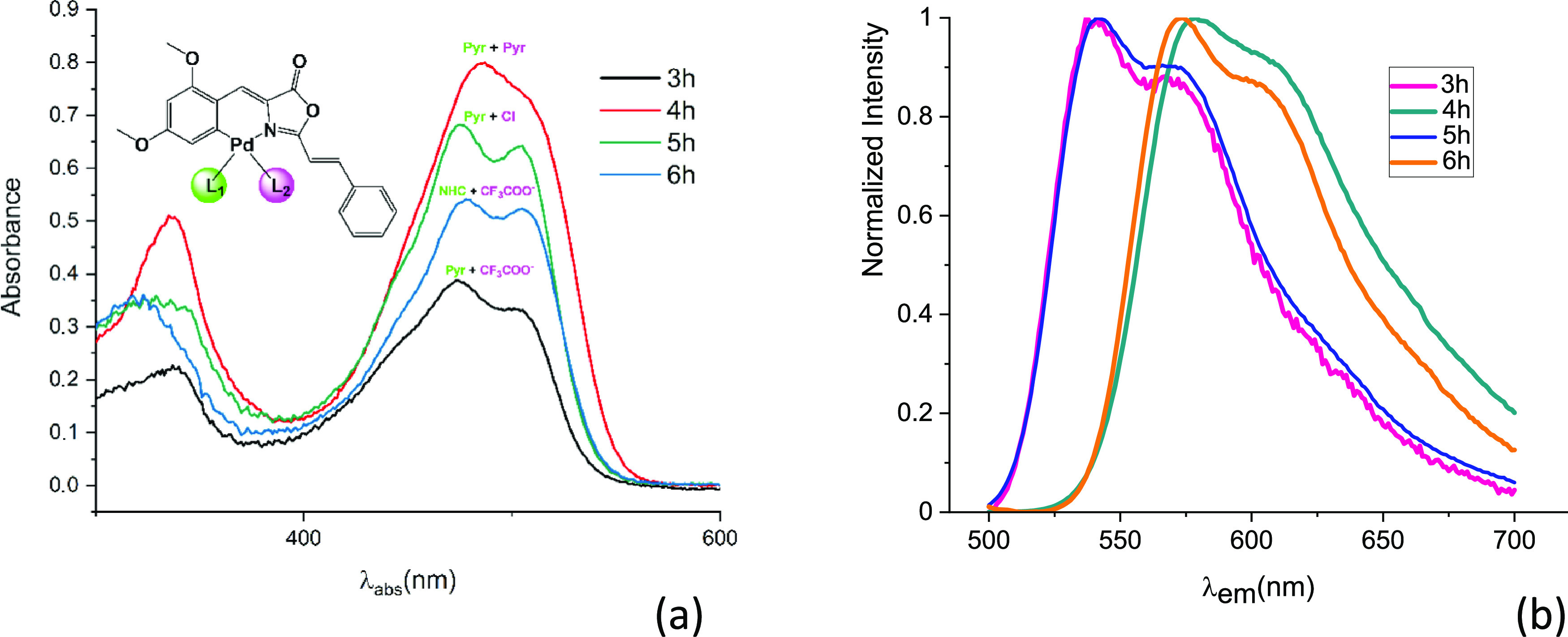
Absorption (a) and emission (b) spectra of 10^–5^ M CH_2_Cl_2_ solutions of complexes with the highest molar absorptivity and highest quantum yields (**3h**, **4h**, **5h**, **6h**).

Photophysical data of **1h**, **3a**–**3k**, **3m**, **4d**, **4h**, **5h**, and **6h** including their absorption, emission maxima, life times, and emission quantum yields (ϕ_em_) are summarized in Table 1. Most oxazolones have the strongest absorption maxima in the UV region of the spectrum, corresponding to a π– π* charge transfer from the 4-arylidene ring to the oxazolone heterocyclic moiety.^10a^^,b^ In the reported orthopalladated derivatives, the maxima are red-shifted to the blue-green region with respect to free oxazolones.^10e^ The complexes reported in this study follow this trend. Their absorption spectra in CH_2_Cl_2_ solutions resemble each other and display an intense absorption in the blue-green region (424–516 nm) accompanied by a less intense band in the UV region. For complexes **3g**, **3k**, and **3m**, the absorption maxima at 386 or 371 nm show a similar intensity than those observed in the visible region, at λ > 440 nm (see Table 1). It is worth noting that the complexes with the styryl fragment in the R^2^ position have the most intense absorption band more shifted to a higher wavelength compared to those with R^2^ = Ph (Figure 1), which could be attributable to an extended π conjugation. This red shift is also observed upon increasing aromaticity from **3b** to **3c** at R_1_. The bands with the highest absorbance are assigned to IL π → π* transition of the oxazolone fragment, as explained above. This assignment is supported by similar absorption bands present in the free oxazolones and by computational MO calculations (see below). The most intense absorptions are observed for those complexes with R^2^ = styryl and R^1^ = 2,4-OMe-C_6_H_4_ (**3h**, **4h**, **5h**, and **6h**) (Figure 4a).

**Table 1:** Absorption and Emission Maxima, Half-Life Times, and Fluorescence Quantum Yield for **1h**, **3a**–**3k**, **3m**, **4d**, **4h**, **5h**, and **6h**

compound^a^	λabs (nm)	λem (nm)	Φ_em_ (%)	τ (ns)
**1h**^b^	315, 428	505	<1	0.2
**3a**	306; 386 ;448, 468	521, 542	5	**0.3**
**3b**	290; 367; 460, 485	525, 550	4	**0.5**
**3c**	251; 336; 482, 516	577	<1	0.8
**3d**	254; 306; 450, 471	539	7	**0.7**
**3e**	252; 301; 457	534, 564	5	**0.5**
**3f**	261; 326; 474	630	4	**0.4**
**3g**	317; 378; 445, 466	534, 550	<1	0.4
**3h**	255; 337; 475, 501	539, 570	18	**1.2**
**3i**	311; 349, 474, 500	544, 574	3	0.3
**3j**	314, 322; 424, 465	533	<1	1.1
**3k**	315; 386; 445	485, 510, 550	<1	1.0
**3m**	324, 386; 486	605	<1	0.2
**4d**	258, 312; 471	526, 553	10	0.8
**4h**	261, 341; 488, 510	558, 590	28	**1.7**
**5h**	263, 332; 481, 507	542, 593	12	**0.8**
**6h**	277, 321; 483, 510	548, 570	15	**0.9**

a Measured in 10^–5^ M CH_2_Cl_2_ solution at room temperature.

b Reference (10b). Red values correspond to the lower energy absorptions with the maximum peak underlined.

**Figure 5:**
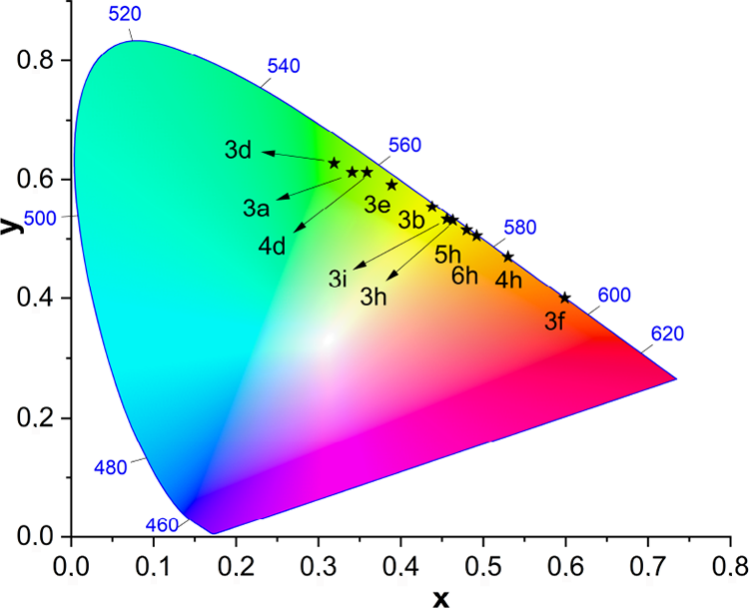
CIE 1931 x, y coordinates (2°).

Palladacycles are usually fraught with low-efficiency emissions, only detected at low temperature, due to the thermal deactivation of the π → π* excited state to the low-lying metal-centered orbitals (MC; d–d*), which experience non-radiative decay.^19^ In this work, the emissive properties of the orthometallated complexes **3**–**6** have been studied. These complexes have been built in order to carry out a systematic study of the influence of R^1^, R^2^, L_1_, and L_2_ (Figure 1) in the emissive properties. Their excitation spectra closely match the corresponding absorption spectra (Figure 4b and Figure SI), and their emission maxima in dichloromethane solution (from 485 to 630 nm) almost cover the whole visible spectrum (Table 1). This point is illustrated in Figure 5, which shows the CIE 1931 coordinates.

**Figure 6:**
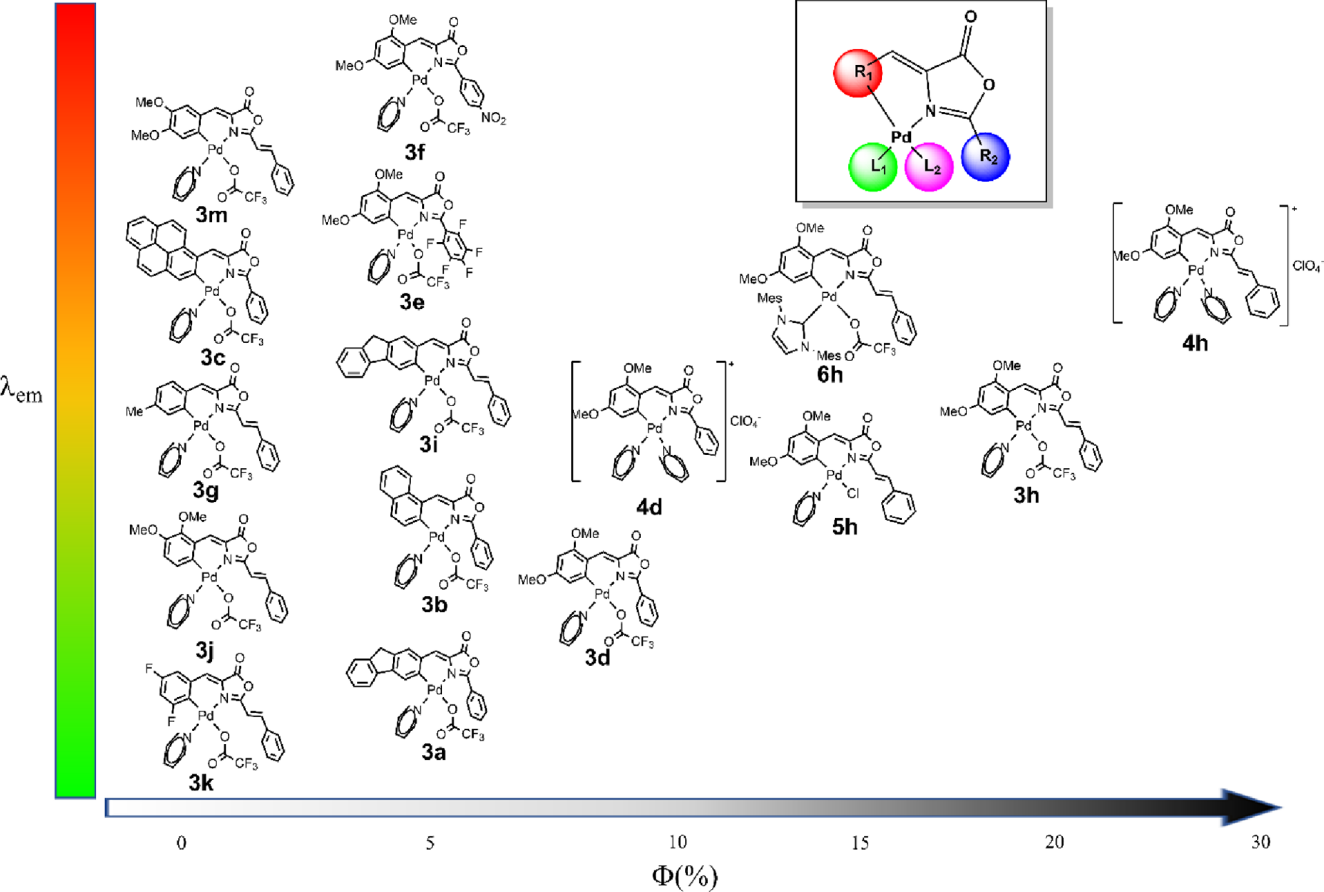
Representation of the wavelength (Y-axis) and quantum yield (X-axis) for the studied palladium complexes showing the oxazolone R_1_ and R_2_ building blocks and the ancillary ligands L_1_ and L_2_.

Emission lifetimes (τ) of the cyclometallated complexes range from 0.2 to 1.7 ns. An IL-dominated ^1^ILCT π → π* emissive state origin of the emission is strongly suggested on the basis on the observed lifetime values, which resemble that reported for the free oxazolone **1h** (τ = 0.2 ns) and to the fact that the emission properties remain unchanged in the presence of air, as was proved for the most emissive sample **4h**. Quantum yield values range from 3 to 7% for complexes **3a**, **3b**, **3d**, **3e**, **3f**, and **3i** and from 10 to 28% for **3h**, **4d**, **4h**, **5h**, and **6h**, while **3c**, **3g**, **3j**, **3k**, and **3m** are slightly emissive (Φ < 1%). Figure 6 shows a pseudo-2D distribution of the complexes, taking into account the measured quantum yield (X-axis) and the observed emission wavelength (Y-axis) for each compound and may lead to the following trends.

#### R^1^ Substituent

From Figure 6, it can be inferred that the presence of emissive groups in the starting oxazolone (pyrenyl **3c**, naphthyl **3b**, fluorenyl **3a** or **3i**) does not guarantee the obtention of a fluorescent complex. Figure 6 also shows that the presence of methoxy groups at 3- and 5-positions of the R^1^ ring (that is, in *meta* to the Pd–C bond) is almost mandatory to achieve high quantum yields. These positions, and not others, gave the best results. If we consider the series of complexes **3j** [3,4-(OMe)_2_], **3m** [4,5-(OMe)_2_], and **3h** [3,5-(OMe)_2_], the observed quantum yields range from <1% for **3j** and **3m** to 18% for **3h** (Figure 6). The presence of other substituents such as fluorine in 3,5 positions do not cause an increase of the quantum yield, as observed in previous studies where the intensity of emission of the acetylacetonate complex was very close to that obtained for the free oxazolone.^10f^

#### R^2^ Substituent

Once the best R^1^ group (2,4-OMe-C_6_H_4_) was found, the influence of the R^2^ fragment may be studied. An attempt was made to have larger charge distributions in the oxazolone fragment using more pronounced push–pull systems **3f** (R^2^ = C_6_H_4_-NO_2_) and **3e** (R^2^ = C_6_F_5_). This is a valid strategy used to improve the emissive properties of free oxazolones.^10c^^,d^ However, for the studied complexes, that was not the case and complex **3d** with R^2^ = C_6_H_5_ displays a higher quantum yield compared with those with R^2^ = C_6_H_4_-NO_2_ or R^2^ = C_6_F_5_. When the R^2^ = C_6_H_5_ fragment was replaced by a styryl fragment, a marked increase in the quantum yield was found for **3d** (7%) vs **3h** (18%) or for **4d** (10%) vs **4h** (28%).

#### Ancillary Ligands (L_1_ and L_2_)

Aiming to increase the σ-donor ability of the auxiliary ligands, a bulky NHC^20^ was used instead of pyridine in complex **6h**. While this complex is one of the most strongly luminescent (Φ = 15%) complexes presented in this work, it shows lower emission efficiency with respect to pyridine derivatives **3h** or **4h**, which is especially important upon comparison with **4h**. Substitution of the CF_3_CO_2_ ligand in **3h** by a chloride is detrimental, and the corresponding complex **5h** shows a lower value of quantum yield (Φ = 12%). Better results were obtained changing the trifluoroacetate ligand by a neutral ligand such as another pyridine, from **3h** (Φ **=** 18%) to **4h** (Φ **=** 28%). The same trend has been observed from **3d** (Φ = 7%) to **4d** (Φ = 10%), although the increase is lower. In this case, it must be taken into account the change in the complex charge, from neutral to cationic.

The quantum yield of **4h** (Φ = 28%) is one of the highest values found for an organometallic palladium complex.^11^ This result is remarkable not only from the point of view of the absolute value but mostly because it arises from a ligand, which is almost non-luminescent in free state in solution.^10f^ That is, we have produced an amplification of the luminescence of several orders of magnitude, which is a very scarce phenomenon despite the fact that intensely luminescent Pd complexes are known.^11^ In fact, this is the opposite behavior to what is commonly found in Pd complexes, because in most reported cases, the incorporation of the palladium causes a big decrease or almost complete loss of luminescence.^10a,11,21^ Aiming to gain more insight about the intimate reasons for this amplification, we have tried to correlate the molecular parameters with the observed fluorescence. Following the crystallographic analysis performed in complexes **3c**, **3d**, **3g**, and **6h**, detailed in the Supporting Information, it is possible to observe that molecules with similar distortions (for instance, **3c** and **3d**) show very different values of quantum yield (<1 and 7%, respectively) or that highly distorted molecules as **5h** (by similarity with **6h**) show high values of quantum yield (15% for **5h**). These facts suggest that Φ does not correlate in a straight way with the steric factors, and that is probably more related with electronic factors. Therefore, we have studied these systems by molecular modeling using DFT/TDDFT.

### 3.- Computational Simulations

The absorption and emission properties of the whole set of compounds **3**–**6** have been calculated using the M06-2X functional, as described in the Computational Details section. The obtained results are summarized in Tables 2 and 3, which contain information regarding the ground state, vertical absorption properties from the ground-state optimized geometries, and emission properties from the optimized geometries of S_1_ excited states.

**Table 2:** HOMO and LUMO Orbital Energies, in Hartree, along with the Pd Contribution in Parenthesis

	ground state	
complex	**HOMO**	**LUMO**
**3a**	–0.268 (13%)	–0.085 (2%)
**3b**	–0.265 (8%)	–0.086 (2%)
**3c**	–0.251 (3%)	–0.090 (4%)
**3d**	–0.262 (8%)	–0.077 (5%)
**3e**	–0.269 (8%)	–0.084 (5%)
**3f**	–0.267 (9%)	–0.061 (4%)
**3g**	–0.267 (10%)	–0.085 (5%)
**3h**	–0.257 (5%)	–0.081 (5%)
**3i**	–0.266 (13%)	–0.089 (2%)
**3j**	–0.269 (14%)	–0.087 (4%)
**3k**	–0.278 (22%)	–0.091 (15%)
**3m**	–0.254 (11%)	–0.083 (3%)
**4d**	–0.294 (3%)	–0.054 (4%)
**4h**	–0.268 (5%)	–0.093 (4%)
**5h**	–0.256 (11%)	–0.080 (15%)
**6h**	–0.251 (4%)	–0.077 (6%)

**Table 3:** Calculated Absorption and Emission Properties for T_1_ and S_1_ Excited Electronic States, along with the Experimental Data in nm^a^

	absorption			emission		
complex	T_1_	S_1_	exp	S_1_	exp	Φ
**3a**	583	405 (0.052)	468	489 (0.586)	542	5%
**3b**	646	424 (0.466)	485	541 (0.759)	550	4%
**3c**	693	453 (1.075)	516	585 (1.471)	577	<1%
**3d**	628	413 (0.470)	450	509 (0.904)	539	7%
**3e**	642	417 (0.465)	457	518 (0.853)	564	5%
**3f**	647	429 (0.650)	474	566 (0.952)	630	4%
**3g**	641	407 (0.667)	445	532 (1.353)	534	<1%
**3h**	675	431 (0.965)	475	560 (1.302)	570	18%
**3i**	635	412 (0.590)	474	532 (1.212)	574	3%
**3j**	630	406 (0.361)	424	526 (1.262)	533	<1%
**3k**	618	409 (0.038)	445	514 (1.345)	550	<1%
**3m**	683	438 (0.816)	486	561 (1.167)	605	<1%
**4d**	630	411 (0.602)	471	516 (0.884)	553	10%
**4h**	676	434 (0.964)	488	567 (1.300)	590	28%
**5h**	676	432 (0.891)	481	562 (1.273)	593	12%
**6h**	692	438 (0.945)	483	560 (1.301)	593	15%

a Oscillator strengths for S_1_ states are given in parenthesis. Excited-state T_1_ always cross ground-state S_0_. Experimental quantum yields Φ.

**Figure 7:**
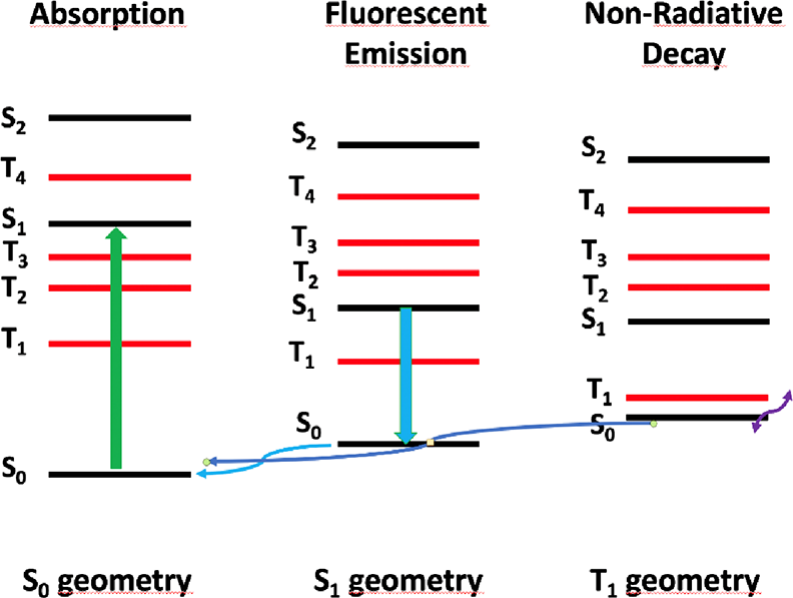
Schematic Jablonski diagram representation of the calculated excited states for different oxazolone complexes at different S_0_, S_1_, and T_1_ optimized geometries, according to TDDFT calculations.

In general, all studied compounds show a similar characteristic excited states behavior, summarized in Figure 7, even if they show different experimental quantum yield values. According to this Jablonski diagram, the absorption properties correspond to the excitation from the ground state (S_0_) to the first excited singlet state (S_1_), which may be seen as HOMO to LUMO transition. In between these singlet states, several triplet excited states are located. These calculated triplet states and the calculated transition probabilities to higher singlet excited states are collected in Tables S4 and S5 of the Supporting Information. The S_2_ excited states lie much higher in energy and have much lower oscillator strengths. In addition, the calculated wavelengths for the S_1_ state compare rather well with the experimental ones. According to these facts, the emission properties would be determined by the evolution of the S_1_ electronic state.

In order to explain the observed emitted light, geometry optimizations of the S_1_ state have been carried out. Calculated emission wavelengths match pretty well with the experimental ones, and hence, we can conclude that the fluorescent emission occurs from the S_1_ to S_0_ states, as one might expect. In addition to this, comparing the oscillator strengths for absorption and emission, given in Tables 2 and 3, one may observe that in both cases they are high, indicating that these transitions are very probable. Despite these facts, the quantum yields in solution are in general small for many studied compounds and we wonder about the reasons of this behavior.

A more detailed analysis of Figure 7 provides strong clues to understanding our orthopalladated systems. We can observe that in the S_1_ optimization, this electronic excited state crosses several triplet states (see Table S6 of SI for more details). Hence, the evolution of the S_1_ electronic state could follow an intersystem crossing (ISC) to a triplet excited state because of the presence of a heavy atom such as Pd. In this case, eventually the system would end up in the lowest triplet state (T_1_) by internal conversion. Noticeably, during the T_1_ optimization, there is an inverse electronic state crossing with S_0_. Nevertheless, we should keep in mind that the TDDFT method fails when two electronic states are near degenerate (see the Supporting Information for further discussion).This near-degeneracy would imply a non-radiative decay from T_1_ to S_0_ via vibrational states, which would be responsible for decreasing the quantum yields. In summary, S_1_ evolution may follow two different paths, which would end up in fluorescent radiative or non-radiative decays.

The probability of both paths occurring would be determined by the spin–orbit coupling between S_1_ and triplet states. These calculations are very time consuming; moreover, we must keep in mind that for different oxazolone-Pd compounds, this crossing may occur with different triplet states. Therefore, the calculation of the spin–orbit couplings for these compounds is out of the scope of this work. Nevertheless, it is known that transition metals increase this coupling and, hence, the presence of the Pd center in these compounds is the main responsible for this second alternative non-radiative path.

**Figure 8:**
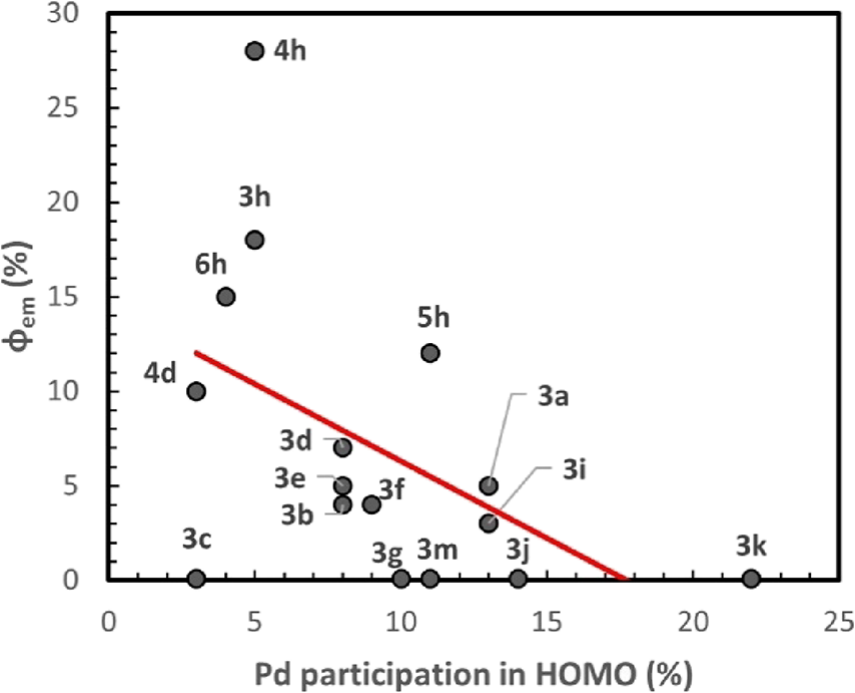
Experimental quantum yield versus metal participation in HOMO for the selected compounds.

An alternative and more approximate way to estimate the S_1_–T_i_ crossing is by the Pd participation in the HOMO orbital of the ground state.^22^ Since the S_0_–S_1_ transition is predominantly a HOMO–LUMO transition, the higher metal participation in the HOMO orbital would be connected with a higher spin–orbit coupling between S_1_ and a triplet state, enhancing the transition to the triplet state. In Figure 8, the participation of the Pd atom in the HOMO orbital versus the observed experimental quantum yield is depicted. Notice that the nature of the oxazolones used in these compounds is very different, and, despite this fact, a clear trend may be observed in Figure 8. In general, larger participation of the metal in the HOMO orbital relates to smaller quantum yields, as expected. Recall that large metal participation would relate to more probable non-radiative decay, and hence low metal participation would decrease the probability for the non-radiative decay process. Unfortunately, although the observed trend is clear, it is not possible to quantitatively connect the calculated metal participation in the HOMO with the experimentally measured quantum yield, since the nature of the oxazolone compounds is rather different.

**Figure 9:**
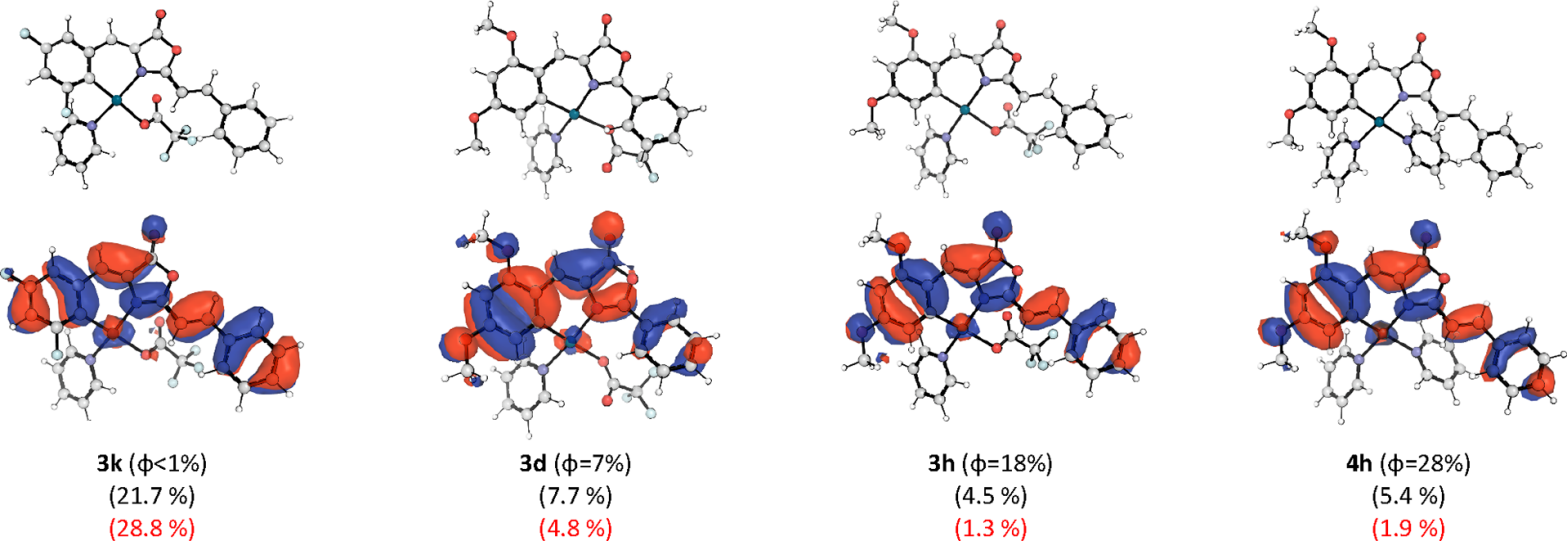
Above, optimized structures for the selected Pd-oxazolone complexes. Below, HOMO orbitals with the corresponding Pd contributions (black) and adjacent C atom (red) in parentheses.

According to the experimental data, one could conclude that there are three main factors to obtain fluorescent Pd-oxazolone complexes. The most important one would be the substituents used and their position in the aromatic ring, and then, the extension of the π-system of the oxazolone and the nature of the ligands attached to the Pd atom. In order to rationalize this experimental evidence with the metal participation in the HOMO orbital, we have focused on six different oxazolones with different fluorescence activities, aiming to cover all representative situations. In Figure 9, four of these oxazolone complexes are collected, from left to right, with increasing quantum yields of 1, 7, 18, and 28%, corresponding to complexes **3k**, **3d**, **3h**, and **4h**, respectively. **3k** shows no fluorescence activity, like the free oxazolone. This compound has F substituents in 3- and 5-positions. **3k** is representative of a set of oxazolone complexes where no amplification is produced, despite the π-extension and/or the presence of py/trifluoroacetate ligands (**3g**, or **3i**, or any of the GFP derivatives: **3a**, **3b**, **3c**, and so on). Complex **3d** is representative of oxazolone complexes with amplification without π-extension and with the “standard” ligands py/trifluoroacetate. It has OMe substituents in 2- and 4-positions and can be considered as the simplest complex among those experiencing amplification. Notice that this change in substituents increases the quantum yield from <1 to 7%. Interestingly, the metal participation in the HOMO orbital decreases significantly from 21.7 to 7.7%. Introduction of π-extension in **3h** further increases the quantum yield to 18%, while the metal participation in the HOMO orbital decreases even more to 4.5%.

Finally, in **4h**, one of the ligands has been substituted and we observe the same small metal participation in agreement with the observed high quantum yield. However, the differences in the observed quantum yields for **3h** and **4h** cannot be explained only with the calculated metal participation in the HOMO. In Figure 9, the participation of the C atom belonging to the phenyl ring of the oxazolone is also included. In complexes with observed fluorescence activity, the participation of both atoms decreases significantly. This may indicate a decrease of the electronic density around the Pd atom, which would be the reason to decrease the Pd participation in the HOMO and hence the increase of the fluorescence quantum yield.

**Figure 10:**
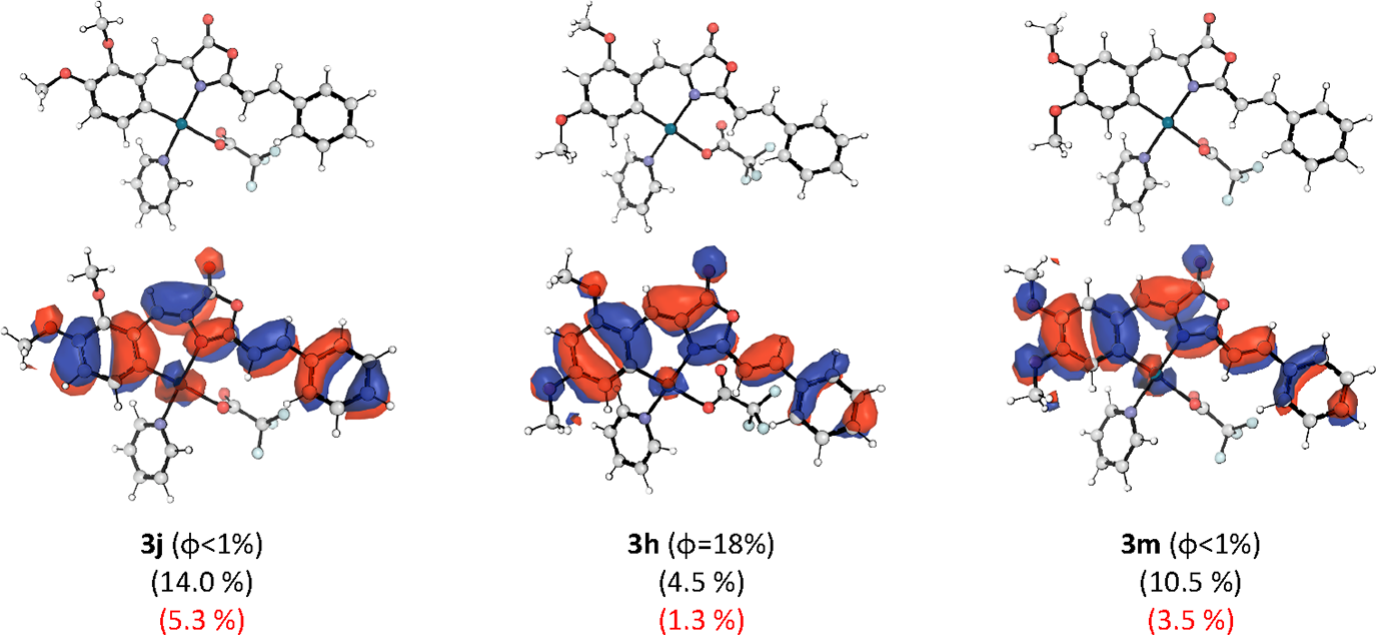
Above, optimized structures for the selected Pd-oxazolone complexes. Below, HOMO orbitals with the corresponding Pd contributions (black) and C6 atom (red) in parentheses.

In order to analyze the influence of the substituent positions, we summarize the metal and C_6_ participation of **3j**, **3h**, and **3m** complexes in Figure 10. The only difference is the position of the OMe groups in these compounds. It may be seen that the presence of the OMe groups in 2- and 4-positions favors the decrease of participation of these atoms in the HOMO orbital, in agreement with previous results. Aiming to shed more light on this, we have also calculated the Natural Charges by means of NBO calculations^23^ of some selected complexes, including those with the highest quantum yields. All data are collected in Table 4.

**Table 4:**
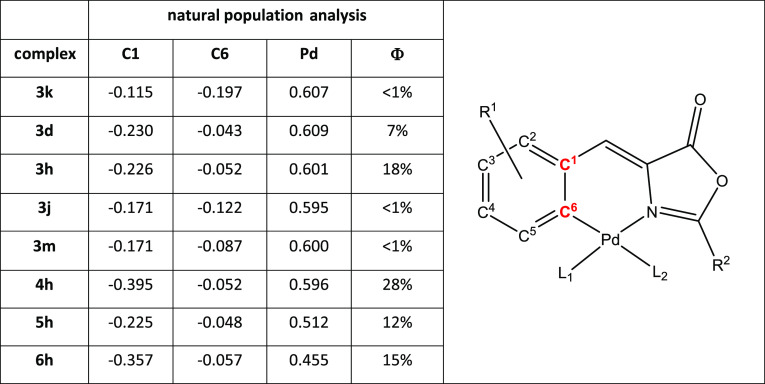
Natural Charges along Phenyl Ring C Atoms and Pd Atom of the Selected Compounds

The calculated charge of C6, which is linked to the Pd atom, is significantly decreased in the compounds with observed fluorescent activity. This is in agreement with the decrease of the participation of this atom in the HOMO orbital. Noticeably, the charge of the C1 atom, which is linked with the rest of the oxazolone ligand, increases in these cases. This is an indication that the electronic density is moved from those atoms of the oxazolone ring to the rest of the ligand. The large C1 charge observed in **4h** may indicate that this delocalization is larger in this compound, helping to the increase of the quantum yield. In summary, the OMe groups in the 2- and 4-positions have the resonant effect of decreasing the participation of the C6 and Pd atom in the HOMO. The extra CH=CH group and ligand effect help in the extra delocalization of the HOMO in the oxazolone, increasing in this way the observed experimental fluorescent quantum yields.

## Conclusions

The amplification of the fluorescence of 4-aryliden-5(4*H*)-oxazolones through intramolecular lock in Pd(II) complexes has been achieved. Starting from non-luminescent ligands (Φ < 0.01%), values of the quantum yield up to 28% can be obtained in the corresponding Pd derivatives. The amplification is quite sensible to the nature of substituents on the 4-arylidene ring, with the presence of two OMe groups in meta to the Pd atom being the case where the highest increases are produced. As a general trend, the wavelength of the emission is tuned by the nature of the oxazolone for identical ancillary ligands, while the quantum yield is modulated by the PdL_2_ fragment in complexes with the same oxazolone. DFT and TD-DFT modeling of the prepared complexes show that the emission band has a clear IL character, with a small yet critical participation of the Pd orbitals, and that there is a clear inverse relationship between the quantum yield measured and the participation of the Pd in the HOMO. The main non-radiative deactivation pathway has been determined also by TD-DFT and is due to the ISC of the S_1_ state with lower-energy triplets (S_1_ → T_2_ or S_1_ → T_1_) and the final non radiative process from T_1_ to the ground state (T_1_ → S_0_). The probability of ISC increases as the contribution of the Pd increases, due to the spin–orbit coupling constant of the Pd. Therefore, this mechanism explains the role of the Pd in the fluorescence loss and allows to make predictions for the design of new Pd complexes with improved fluorescent properties.

## Experimental Section

### General Methods

Solvents were obtained from commercial sources and were used without further purification. All reactions were performed without special precautions against air and moisture. Electrospray ionization (ESI^+^) mass spectra were recorded using Bruker Esquire 3000 Plus or Amazon Speed ion-trap mass spectrometers equipped with standard ESI sources. High-resolution mass spectra-ESI (HRMS-ESI) were recorded using either a Bruker micrOTOF-Q system equipped with an API-ESI source and a QToF mass analyzer, or a TIMS-TOF system, both allowing a maximum error in the measurement of 5 ppm. Acetonitrile was used as solvent. For all types of MS measurements, samples were introduced in a continuous flow of 0.2 mL/min and nitrogen served as both the nebulizer gas and the dry gas. The ^1^H, ^13^C, and ^19^F NMR spectra of the isolated products were recorded in CDCl_3_, CD_2_Cl_2_, and dmso-d_6_ solutions at 25 °C (other conditions were specified) on Bruker AV300, AV400, or Bruker AV500 spectrometers (δ in ppm, *J* in Hz) at ^1^H operating frequencies of 300.13, 400.13, and 500.13 MHz, respectively. The ^1^H and ^13^C NMR spectra were referenced using the solvent signal as internal standard, while ^19^F NMR spectra were referenced to CFCl_3_. The assignment of ^1^H NMR peaks has been performed through standard 2D ^1^H COSY (2 K points in *t*_2_ using a spectral width of 10 ppm; 128 *t*_1_ experiments were recorded and zero-filled to 1 K; for each *t*_1_ value, four scans were signal-averaged using a recycle delay of 1 s) and selective 1D ^1^H-SELNOE experiments. Typical mixing times in the case of selective 1D-SELNOE experiments were in the range 0.8–2 s, as a function of the irradiated signal. These values of optimized mixing times were set equal to the longitudinal relaxation time T_1_, determined using the inversion–recovery sequence. The ^13^C NMR peaks were identified using standard ^1^H–^13^C edited-HSQC and ^1^H–^13^C HMBC 2D experiments. In both cases, 4 K points in *t*_2_ using spectral widths of 10 ppm (^1^H) and 200 ppm (^13^C) were used, with averaged values of the coupling constants ^1^*J*_CH_ = 145 Hz and long-range *^n^J*_CH_ = 10 Hz. Typically, 128 *t*_1_ experiments were recorded and zero-filled to 2 K. For each *t*_1_ value, 8 (HSQC) or 32 (HMBC) scans were signal-averaged using a recycle delay of 1 s. Absorption spectra were measured on a Thermo Scientific Evolution 600 spectrophotometer. The steady-state excitation–emission spectra were measured on a Jobin Yvon Horiba FluoroLog FL3-11 spectrofluorometer. All measurements were carried out at room temperature on solutions of 10^–5^ M concentration using quartz cuvettes of 1 cm path length. Lifetime measurements were carried out in a FluoTime 300 (PicoQuant) fluorescence spectrometer, using excitation LEDs of 450 nm. The measurement of the quantum yield values (Φ_PL_) was carried out using the absolute method on a Quantaurus-QY C11347 spectrometer. Two different CH_2_Cl_2_ solutions of each compound (10^–5^ M) were measured in order to check data reproducibility. Relative uncertainty for the absolute method has been determined as less than 6%, although studies were carried out for different substances using both the absolute method and the comparative one.^24^ In addition, one solution of compounds **4d** and **3h** as representative examples was deoxygenated by passing argon through it, and the value of the QY was redetermined to check the influence of O_2_ in the intensity of the luminescence. The oxazolones **1a**–**1m** were prepared using the Erlenmeyer–Plöchl method, by reaction of the corresponding hippuric acids and aldehydes in acetic anhydride.^12^ The hippuric acids were prepared by the Schotten–Baumann method.^13^

### X-ray Crystallography

Single crystals of **1a**, **1e**, **3c**, **3d**, **3g**, and **7h** CHCl_3_ of suitable quality for X-ray diffraction measurements were grown by slow diffusion of *n*-pentane into CH_2_Cl_2_ or CHCl_3_ solutions of the crude products at −18 °C for several weeks, except for **7h**. Crystals of **7h** were obtained when **6h** was left to crystallize in CHCl_3_, due to the presence of residual HCl in the chlorinated solvent. One selected single crystal of each compound was mounted at the end of a quartz fiber in a random orientation, covered with perfluorinated oil (magic oil) and placed under a cold stream of N_2_ gas. Crystallographic measurements were carried out at 100 K on a Bruker APEX D8 Venture CCD diffractometer, using graphite monochromated Mo Kα radiation (λ = 0.71073 Å). A hemisphere of data was collected in each case based on ω-scan or φ-scan runs. The diffraction frames were integrated using the program SAINT,^25^ and the integrated intensities were corrected for absorption with SADABS.^26^ The structures were solved by direct methods with SHELXT-2014.^27^ All non-hydrogen atoms were refined with anisotropic displacement parameters. The hydrogen atoms were placed at idealized positions and treated as riding atoms. Each hydrogen atom was assigned an isotropic displacement parameter equal to 1.2–1.5 times the equivalent isotropic displacement parameter of its parent atom. For structure solving and refinement, the SHELXL-2016^28^ program in the WINGX Package was used.^29^ The structures were refined to F_o_^2^, and all reflections were used in least-square calculations. CCDC-2122909 (**1a**), CCDC-2122910 (**1e**), CCDC-2122911 (**3c**), CCDC-2122912 (**3d**), CCDC-2122913 (**3g**), and CCDC-2145279 (**7h**) contain supplementary crystallographic data, which can be obtained free of charge from the Cambridge Crystallographic Data Centre via www.ccdc.cam.ac.uk/data_request/cif.

### Computational Details

All calculations were carried out within the density functional theory (DFT),^30^ using the Gaussian 16 program package.^31^ First, in order to characterize the ground electronic state of the selected complexes, geometry optimizations and harmonic frequency calculations were performed by using the wB97XD^32^ and M06-2X^33^ exchange-correlation functionals, combined with the 6-31+G(d,p) basis set for the non-metal atom,^34^ and the ECP10MDF Sttutgart–Cologne relativistic core potentials along with the aug-cc-pVDZ-PP basis set for the Pd atom.^35^ Solvent effects (dichloromethane) were taken into account by means of the integral equation formalism of the polarized continuum model (IEFPCM).^36^ Finally, in order to study the photoabsorption and photoemission processes, time-dependent density functional theory (TDDFT)^37^ was used, at the same levels of theory used in the characterization of the ground states. Vertical transitions were calculated for absorption properties, and geometry optimizations and frequency calculations were carried out for the first singlet and triplet excited states in order to account for emission properties. Both ωB97XD and M06-2X provide similar qualitative and quantitative values for both absorption and emission (see Tables S1–S6 of the Supporting Information). In order to choose one functional, the absorption wavelengths have been calculated and compared to the experimental values for a group of selected complexes. The obtained results are collected in Table 1. It may be observed that both functionals provide similar results for all complexes. Nevertheless, comparing the differences with respect to the experimental value (D1 for ωB97XD and D2 for M06-2X), it may be seen that systematically M06-2X is closer to the experimental value. The calculated average D1 (54 nm) and D2 (48 nm) values confirm that indeed M06-2X is slightly closer than ωB97XD from the experimental value by 6 nm. Hence, for the sake of clarity hereafter, only the M06-2X values will be considered. The molecular orbitals representations were generated by *PyMOL*(38) using Paton Research Group openly accessible display settings.^39^

### General Synthesis and Characterization of Oxazolones **1a**–**1m**

The oxazolones **1a**,^14a^**1b**,^14b^**1c**,^14c^**1d**,^14d^**1f**,^14e^**1g**,^14f^ and **1h**(10f) appear on SciFinder as previously reported. They were characterized by comparison of their NMR data with those previously published. The oxazolones **1e** and **1i**–**1m** have not been previously reported, or they appear on SciFinder without references associated, so they have been fully characterized here. The oxazolones were prepared following the Erlenmeyer–Plöchl method, which is exemplified here with the detailed synthesis of **1a**. The synthesis and characterization of all the other oxazolones appear as the Supporting Information.

##### Synthesis of (*Z*)-4-((9*H*-Fluoren-3-yl) methylene)-2-phenyl-5(4*H*)-oxazolone (**1a**)^14a^

Sodium acetate (420.0 mg, 5.15 mmol) and fluorene-2-carboxaldehyde (1000.0 mg, 5.15 mmol) were added to a solution of hippuric acid (923.0 mg, 5.15 mmol) in acetic anhydride (10 mL). The suspension was heated to the reflux temperature (100 °C) for 3 h and then allowed to cool to room temperature. The solid mass formed upon cooling was treated with distilled water (30 mL) to give **1a** as a yellow solid, which was filtered off, washed with water (5 mL) and cold ethanol (10 mL), and dried under vacuum. Obtained: 1450.0 mg (84% yield).

### General Synthesis and Characterization of Orthopalladated Dimers with Trifluoroacetate Bridges **2**

The orthopalladated dimers **2d**(10g) and **2h**(10f) have been previously described. They were characterized by comparison of their NMR data with those previously published. Complexes **2a**–**2k** and **2m** have been obtained following the same synthetic procedure, which is detailed here for the synthesis of **2a**. The synthesis and characterization of all the other compounds type **2** is given as SI. In all cases, and despite the use of long accumulation times, signals due to the ^13^C nuclei of the CF_3_COO ligand and the C_6_F_5_ group were not observed in the ^13^C NMR spectra, due to multiple ^13^C–^19^F couplings and to the low solubility of the compounds.

#### Synthesis of Orthopalladated **2a**

Pd(OAc)_2_ (200.0 mg, 0.89 mmol) was added to a solution of **1a** (300.0 mg, 0.89 mmol) in CF_3_CO_2_H (5 mL). The resulting mixture was heated in an oil bath to the reflux temperature of the solvent (72.4 °C) for 3 h. After the reaction time, the resulting mixture was cooled to room temperature and distilled water (10 mL) was added. The resulting precipitate was filtered off, washed with more distilled water (3 × 10 mL) until the characteristic smell of trifluoroacetic acid disappeared, dried under vacuum, and identified as **2a** (orange solid). Obtained: 455.0 mg (92% yield). ^1^H NMR (CDCl_3_, 300.13 MHz): δ 7.68–7.63 (m, 2H, C_13_H_8_), 7.60 (m, 2H, H_o_, C_6_H_5_), 7.57 (s, 1H, =CH_vinyl_), 7.56, 7.52 (2s, 2H, H_2_, H_4_, C_13_H_8_), 7.47–7.43 (m, 3H, H_p_, C_6_H_5_, C_13_H_8_), 7.21 (m, 2H, H_m_, C_6_H_5_), 4.10, 3.99 (AB spin system, 2H, *J* = 22 Hz, CH_2_, C_13_H_8_). ^13^C{^1^H} NMR (CDCl_3_, 75.47 MHz): δ 167.6 (C=N), 161.1 (C=O), 145.4 (C_q_, C_13_H_8_), 145.3(C_q_, C_13_H_8_), 141.3 (C_q_, C_13_H_8_), 140.5 (C_q_, C_13_H_8_), 139.2 (=CH, C_vinyl_), 135.1 (=C), 134.5 (C_p_, C_6_H_5_), 130.6 (C_o_, C_6_H_5_), 129.8 (C_3_, C_13_H_8_), 128.8 (CH, C_13_H_8_), 128.4 (C_m_, C_6_H_5_), 128.0 (C_i_, C_6_H_5_), 127.5 (CH, C_13_H_8_), 125.6 (CH, C_13_H_8_), 125.0 (C_5_, C_13_H_8_), 122.5 (C_2_, C_13_H_8_), 122.4 (C_q_, C-Pd), 121.2 (CH, C_13_H_8_), 36.1 (CH_2_, C_13_H_8_). ^19^F NMR (CDCl_3_, 282.40 MHz): δ −74.98. HRMS (ESI) *m*/*z*: [M–CF_3_COO]^+^ calcd for [C_48_H_28_F_3_N_2_O_6_Pd_2_]^+^ 998.9973. Found 998.9965.

### General Synthesis and Characterization of Mononuclear Orthopalladated Pyridine and bis-Pyridine Complexes (**3a**–**3d**, **3f**–**3k**, **3m**, **4d**, **4h**, **5h**, **6h**)

The synthesis of derivatives **3** containing one pyridine ligand has been carried out in all cases using the same procedure, detailed here for the synthesis of **3a**. The synthesis and characterization of all compounds **3** is given as SI. For all prepared complexes, signals assigned to the quaternary carbons of the CF_3_COO ligand were not found in the ^13^C NMR spectra. This is due to a dynamic fast coordination release of this ligand, which takes place at room temperature. In the case of **3e**, as representative, the ^13^C NMR spectrum was measure at low temperature (233 K) and all signals were observed.

#### Synthesis of Orthopalladated **3a**

Pyridine (14.5 μL, 0.185 mmol) was added to a stirred suspension of **2a** (100.0 mg, 0.090 mmol) in CH_2_Cl_2_ (10 mL) at room temperature. The starting suspension gradually dissolved, and a yellow solution was obtained after a few minutes. The mixture was further stirred at room temperature for 30 min. At this point, any remaining insoluble residue was removed by filtration. The clear yellow solution was evaporated to dryness, and the obtained yellow solid of **3a** was dried under vacuum. Obtained: 96.7 mg (86% yield). ^1^H NMR (CD_2_Cl_2_, 300.13 MHz): δ 8.72 (m, 2H, H_o_, C_5_H_5_N), 8.49 (m, 2H, H_o_, C_6_H_5_), 7.85 (tt, 1H, *J* = 7.8 Hz, *J* = 1.4 Hz, H_p_, C_5_H_5_N), 7.70 (t, 1H, *J* = 7.3 Hz, H_p_, C_6_H_5_), 7.68 (s, 1H, =CH_vinyl_), 7.61 (m, 2H, H_m_, C_6_H_5_), 7.53–7.51 (m, 2H, C_13_H_8_), 7.42 (m, 1H, C_13_H_8_), 7.37 (m, 2H, H_m_, C_5_H_5_N), 7.34–7.28 (m, 2H, C_13_H_8_), 7.06 (s, 1H, C_13_H_8_), 3.87 (s, 2H, CH_2_, C_13_H_8_). ^13^C{^1^H} NMR (CD_2_Cl_2_, 75.47 MHz): δ 167.5 (C=N), 162.0 (C=O), 153.5 (C_o_, C_5_H_5_N), 143.4 (2C overlapped, 2C_q_), 143.2 (C_q_), 141.0 (C_q_), 140.6 (C_q_), 140.0 (=CH, C_vinyl_), 139.2 (C_p_, C_5_H_5_N), 134.9 (C_p_, C_6_H_5_), 132.4 (C_q_), 130.9 (C_o_, C_6_H_5_), 129.1 (CH), 129.1 (CH), 128.9 (C_m_, C_6_H_5_), 128.5 (CH), 127.3 (CH), 125.7 (CH), 125.6 (C_m_, C_5_H_5_N), 124.2 (C_q_), 123.4 (C_q_), 120.7 (CH), 36.4 (CH_2_). ^19^F NMR (CD_2_Cl_2_, 282.40 MHz): δ −75.99 (CF_3_COO). HRMS (ESI) *m*/*z*: [M – CF_3_COO + H]^+^ calcd for [C_28_H_19_N_2_O_2_Pd]^+^ 524.0461. Found 524.0463. Elem. anal. calc for [C_30_H_18_F_3_N_2_O_4_Pd]: C, 56.84; H, 2.86; N, 4.42. Found: C, 56.74; H, 3.16; N, 4.70.

#### Synthesis of Orthopalladated bis-Pyridine Complex **4d**

The dinuclear chloride bridge precursor[Pd(μ-Cl)(C^N-**1d**)]_2_, containing the orthopalladated oxazolone **1d**, was prepared as described previously.^10g^ A suspension of [Pd(μ-Cl)(C^N-**1d**)]_2_ (115.0 mg, 0.128 mmol) in 10 mL of CH_2_Cl_2_/acetone (8/2) was treated with AgClO_4_ (53 mg, 0.256 mmol), and the resulting mixture was stirred for 30 min at room temperature under exclusion of light. After the reaction time, the precipitated AgCl was removed by filtration. The resulting clear orange solution was treated with pyridine (40.6 μL, 0.512 mmol) and further stirred for 30 min. The clear yellow solution thus obtained was evaporated to dryness, and the obtained orange solid of **4d** was dried under vacuum. Obtained: 143.0 mg (83% yield). ^1^H NMR (CDCl_3_, 300.13 MHz): δ 9.03 (m, 2H, H_o_, C_5_H_5_N), 8.47 (m, 2H, H_o_, C_5_H_5_N), 8.30 (s, 1H, =CH_vinyl_), 8.10 (m, 2H, H_o_, C_6_H_5_), 7.80 (t, 1H, *J* = 7.7 Hz, H_p_, C_5_H_5_N), 7.56 (m, 2H, H_m_, C_6_H_5_), 7.53–7.47 (m, 3H, H_m_, C_5_H_5_N; H_p_, C_6_H_5_), 7.40 (t, 1H, *J* = 7.7 Hz, H_p_, C_5_H_5_N), 7.03 (m, 2H, H_m_, C_5_H_5_N), 6.14 (d, 1H, *J* = 2.1 Hz, H_4_, C_6_H_2_), 5.97 (d, 1H, *J* = 2.1 Hz, H_6_, C_6_H_2_), 3.88 (s, 3H, OMe), 3.55 (s, 3H, OMe). ^13^C{^1^H} NMR (CDCl_3_, 75.47 MHz): δ 165.8 (C=N), 163.5 (C_3/5_-OMe, C_6_H_2_), 161.7 (C=O), 160.8 (C_3/5_-OMe, C_6_H_2_), 152.5 (C_o_, C_5_H_5_N), 150.2 (C_o_, C_5_H_5_N), 149.5 (=C), 139.3 (C_p_, C_5_H_5_N), 138.4 (C_p_, C_5_H_5_N), 135.9 (=CH, C_vinyl_), 134.6 (C_p_, C_6_H_5_), 130.1 (C_o_, C_6_H_5_), 129.7 (C_m_, C_6_H_5_), 126.9 (C_m_, C_5_H_5_N), 125.5 (C_m_, C_5_H_5_N), 122.9 (C_i_, C_6_H_5_), 119.5 (C_1_-Pd, C_6_H_2_), 116.7 (C_2_, C_6_H_2_), 114.5 (C_6_, C_6_H_2_), 96.1 (C_4_, C_6_H_2_), 56.0 (OMe), 55.8 (OMe). HRMS (ESI) *m*/*z*: [M–ClO_4_–py + H]^+^ calcd for [C_23_H_19_N_2_O_4_Pd]^+^ 493.0380. Found 493.0371. Elem. anal. calc for [C_28_H_22_ClN_3_O_8_Pd]: C, 50.17; H, 3.31; N, 6.27. Found: C, 50.13; H, 3.64; N, 5.96.

#### Synthesis of Orthopalladated bis-Pyridine Complex **4h**

Complex **4h** was prepared following the same procedure than that reported for **4d** but starting from [Pd(μ-Cl)(C^N-**1h**)]_2_.^10f^ Therefore, [Pd(μ-Cl)(C^N-**1h**)]_2_ (155.0 mg, 0.163 mmol) was reacted with AgClO_4_ (68.0 mg, 0.326 mmol) and pyridine (51.6 μL, 0.652 mmol) in CH_2_Cl_2_/acetone (8/2, 10 mL) under exclusion of light to give **4h** as a red solid. Obtained: 185.0 mg (81% yield). ^1^H NMR (CDCl_3_, 400.13 MHz, 233 K) δ: 9.03 (d, *J* = 5.4 Hz, 2H, H_o_, C_5_H_5_N), 8.97 (d, *J* = 5.4 Hz, 2H, H_o_, C_5_H_5_N), 8.18 (s, 1H, =CH_vinyl_), 7.87 (t, 1H, *J* = 8.1 Hz, H_p_, C_5_H_5_N), 7.62–7.55 (m, 3H, H_m_ + H_p_, C_5_H_5_N), 7.48 (m, 2H, H_m_, C_5_H_5_N), 7.40–7.26 (m, 6H, H_o_ + H_m_ + H_p_ C_6_H_5_, =CH_olef_), 6.25 (d, *J* = 16.0 Hz, 1H, =CH_olef_) 6.10 (s, 1H, H_4_, C_6_H_2_), 5.64 (s, 1H, H_6_, C_6_H_2_), 3.86 (s, 3H, OMe), 3.49 (s, 3H, OMe). ^13^C{^1^H} NMR (CDCl_3_, 100.6 MHz, 233 K) δ: 162.8 (2C, C-OMe, C_6_H_2_ + C=N), 161.7 (C=O), 160.4 (C-OMe, C_6_H_2_), 151.9 (C_o_, C_5_H_5_N), 150.2 (C_o_, C_5_H_5_N), 148.7 (=C), 146.3 (=CH, C_olef_), 139.4 (2C, 2C_p_, C_5_H_5_N), 134.2 (=CH, C_vinyl_), 132.7 (C_i_, C_6_H_5_), 132.1 (C_p_, C_6_H_5_), 129.3 (C_o_/C_m_ C_6_H_5_), 129.0 (C_o_/C_m_, C_6_H_5_), 127.0 (C_m_, C_5_H_5_N), 126.7 (C_m_, C_5_H_5_N), 118.9 (C_2_, C_6_H_2_), 116.1 (C_1_-Pd, C_6_H_2_), 114.3 (C_6_, C_6_H_2_), 109.1 (=CH, C_olef_), 96.7 (C_4_, C_6_H_2_), 55.9 (OMe), 55.6 (OMe). HRMS (ESI) *m*/*z*: [M–ClO_4_–py + H]^+^ calcd for [C_25_H_21_N_2_O_4_Pd]^+^ 519.0541. Found 519.0554. Elem. anal. calc for [C_30_H_24_ClN_3_O_8_Pd]: C, 51.74; H, 3.47; N, 6.03. Found: C, 51.89; H, 3.74; N, 5.94.

#### Synthesis of Orthopalladated Chloride Complex **5h**

The dinuclear precursor[Pd(μ-Cl)(C^N-**1h**)]_2_, containing the orthopalladated oxazolone **1h**, was prepared as described previously.^10f^ A suspension of [Pd(μ-Cl)(C^N-**1h**)]_2_ (136.0 mg, 0.143 mmol) in CH_2_Cl_2_ (10 mL) at room temperature was treated with pyridine (22.6 μL, 0.286 mmol). The initial suspension gradually dissolved, and a clear solution was obtained after a few minutes. This solution was stirred for 30 min, and any remaining solid was removed by filtration after the reaction time. The resulting solution was evaporated to dryness, and the residue treated with Et_2_O (20 mL) and stirring to give **5h** as an orange solid. Obtained: 120.0 mg (76% yield). ^1^H NMR (CD_2_Cl_2_, 300.13 MHz): δ 8.74 (m, 2H, H_o_, C_5_H_5_N), 8.06 (s, 1H, =CH_vinyl_), 8.06 (d, 1H, *J* = 16.2 Hz, =CH_olef_), 7.86 (t, 1H, *J* = 7.7 Hz, H_p_, C_5_H_5_N), 7.70 (m, 2H, H_o_, C_6_H_5_,), 7.61 (d, 1H, *J* = 16.2 Hz, =CH_olef_), 7.45 (m, 3H, H_m_, H_p_, C_6_H_5_), 7.39 (m, 2H, H_m_, C_5_H_5_N), 6.14 (d, *J* = 2.1 Hz, 1H, H_4_, C_6_H_2_), 5.56 (d, *J* = 2.1 Hz, 1H, H_6_, C_6_H_2_), 3.87 (s, 3H, OMe), 3.47 (s, 3H, OMe). ^13^C{^1^H} NMR (CD_2_Cl_2_, 75.47 MHz): δ 165.1 (C=N), 163.1 (C_3/5_-OMe, C_6_H_2_), 161.9 (C=O), 160.3 (C_3/5_-OMe, C_6_H_2_), 153.8 (C_o_, C_5_H_5_N), 143.8 (=C), 145.4 (=CH, C_olef_), 138.8 (C_p_, C_5_H_5_N), 135.7 (C_i_, C_6_H_5_), 133.0 (=CH, C_vinyl_), 131.4 (C_p_, C_6_H_5_), 129.9 (C_1_-Pd, C_6_H_2_), 129.6 (C_o_, C_6_H_5_), 129.3 (C_m_, C_6_H_5_), 125.7 (C_m_, C_5_H_5_N), 117.0 (C_2_, C_6_H_2_), 115.5 (C_6_, C_6_H_2_), 115.4 (=CH, C_olef_), 95.3 (C_4_, C_6_H_2_), 56.3 (OMe), 55.7 (OMe). HRMS (ESI) *m*/*z*: [M–py + Na]^+^ calcd for [C_20_H_16_ClNNaO_4_Pd]^+^ 497.9700. Found 497.9696. Elem. anal. calc for [C_25_H_20_ClN_2_O_4_Pd]: C, 54.17; H, 3.64; N, 5.05. Found: C, 54.18; H, 3.92; N, 5.02.

#### Synthesis of Orthopalladated NHC Complex **6h**

To a solution of **2h** (100.0 mg, 0.09 mmol) in dry THF (5 mL) under an Ar atmosphere, K_2_CO_3_ (25.0 mg, 0.18 mmol) and 1,3-bis-(2,4,6-trimethylphenyl)imidazolium chloride (61.9 mg, 0.18 mmol) were added. The resulting mixture was heated in an oil bath to the reflux temperature (66 °C) for 24 h. After the reaction time, the cooled solution was evaporated to dryness and the residue was purified by flash column chromatography, using silica gel as support and Et_2_O as eluent. The orange-yellowish band developed was collected, the solvent evaporated to dryness, and the orange solid residue characterized as complex **6h**. Obtained: 50 mg (32% yield). ^1^H NMR (CD_2_Cl_2_, 300.13 MHz): δ 7.63 (s, 1H, =CH_vinyl_), 7.44–7.35 (m, 6H, =CH_olef_, H_o_ + H_m_ + H_p_, C_6_H_5_), 7.11 (s, 2H, =CH, NHC), 7.01 (s, 2H, H_ar_, NHC-C_6_H_2_), 6.86 (s, 2H, H_ar_, NHC-C_6_H_2_), 6.35 (d, 1H, *J* = 16.2 Hz, =CH_olef_), 6.18 (d, 1H, *J* = 2.2 Hz, H_4_, C_6_H_2_), 5.93 (d, 1H, *J* = 2.2 Hz, H_6_, C_6_H_2_), 3.75 (s, 3H, OMe), 3.71 (s, 3H, OMe), 2.38 (s, 6H, Me), 2.31 (s, 6H, Me), 2.17 (s, 6H, Me). ^13^C{^1^H} NMR (CD_2_Cl_2_, 75.47 MHz): δ 170.9 (Pd=C, NHC), 163.4 (C=N), 163.1 (C=O), 162.2 (C_3/5_-OMe, C_6_H_2_), 161.0 (C_3/5_-OMe, C_6_H_2_), 151.4 (=C), 143.8 (=CH, C_olef_), 138.9 (C_q_, NHC-C_6_H_2_), 137.1 (C_q_, NHC-C_6_H_2_), 135.7 (C_q_, NHC-C_6_H_2_), 135.1 (C_i_, C_6_H_5_), 133.9 (C_q_, NHC-C_6_H_2_), 133.7 (=CH, C_vinyl_), 131.2 (CH, C_6_H_5_), 129.9 (CH, NHC-C_6_H_2_), 129.4 (CH, C_6_H_5_), 129.0 (CH, NHC-C_6_H_2_), 128.9 (CH, C_6_H_5_), 123.8 (CH, NHC), 121.9 (C_1_-Pd, C_6_H_2_), 119.1 (C_6_, C_6_H_2_), 118.7 (C_2_, C_6_H_2_), 112.3 (=CH, C_olef_), 93.7 (C_4_, C_6_H_2_), 55.9 (OMe), 55.6 (OMe), 21.3 (Me), 20.0 (Me), 19.3 (Me). ^19^F NMR (CD_2_Cl_2_, 282.40 MHz): δ −74.27 (CF_3_COO). HRMS (ESI) *m*/*z*: [M–CF_3_COO]^+^ calcd for [C_41_H_40_N_3_O_4_Pd]^+^ 744.2054. Found 744.2041. Elem. anal. calc for [C_43_H_40_F_3_N_3_O_6_Pd]: C, 60.18; H, 4.70; N, 4.90. Found: C, 60.18; H, 5.04; N, 4.74.

## Supporting Information Available

Complete experimental section; copies of ^1^H and ^13^C NMR spectra for all new compounds; copies of the absorption (UV–vis) and excitation/emission spectra of complexes **3**–**6**; decay curves and fitting data for the determination of the half-life time of complexes **3**–**6**; Cartesian coordinates of all new optimized complexes **3**–**6** in the ground state using the wB97XD and M06-2X functionals; Cartesian coordinates of S1 optimized geometries using the wB97XD and M06-2X functionals; tables of calculated orbital energies (Hartree) and metal participation, along with the HOMO–LUMO gap (eV) using the wB97XD and M06-2X functionals for the ground state of **3**–**6**; tables with the calculated vertical transitions for the lowest T_1_, T_2_, T_3_, S_1_, and S_2_ excited states of **3**–**6** with the wB97XD and M06-2X functionals; tables with the calculated emission properties from the S1 optimized geometry for the lowest T_1_, T_2_, T_3_, S_1_, and S_2_ excited states of **3**–**6** with the wB97XD and M06-2X functionals; crystallographic tables of compounds **1a**, **1e**, **3c**, **3d**, **3g** and **7h**; and discussion of the structures of compounds **1a**, **1e**, **3c**, **3d**, **3g**, and **7h** (PDF)
