# Supplementary material for: Fluorescence Amplification of Unsaturated Oxazolones Using Palladium: Photophysical and Computational Studies
Source: Inorg Chem. 2023 Jun 14;62(25):9792–806. doi: 10.1021/acs.inorgchem.3c00601 (PMC10302897; doi:10.1021/acs.inorgchem.3c00601)
Supplement: Supplementary file 1 — ic3c00601_si_001.pdf [file ic3c00601_si_001.pdf]

Supporting Information

Fluorescence amplification of unsaturated oxazolones using palladium: photophysical and computational studies

David Dalmau,† Olga Crespo,† Jon M. Matxain,‡ Esteban P. Urriolabeitia*†

† Instituto de Síntesis Química y Catálisis Homogénea, ISQCH (CSIC-Universidad de Zaragoza), Pedro Cerbuna 12, 50009 Zaragoza (Spain)

‡ Polimero eta Material Aurreratuak: Fisika, Kimika eta Teknologia Saila. Kimika Fakultatea. Euskal Herriko Unibertsitatea UPV/EHU and Donostia International Physics Center (DIPC) PK 1072, 20080, Donostia, Euskadi, Spain.

Corresponding author: Esteban P. Urriolabeitia, e-mail: esteban.u.a@csic.es; esteban@unizar.es

Contents

1.- **Complete Experimental Section** S3

2.- **NMR spectra of starting (*Z*)-4-arylidene-5(4*H*)-oxazolones (1)** S21

3.- **NMR spectra of dinuclear μ-trifluoroacetate-orthopalladated derivatives (2)** S38

4.- **NMR spectra of mononuclear trifluoroacetate-pyridine orthopalladated derivatives (3)** S63

5.- **NMR spectra of mononuclear bis-pyridine orthopalladated derivatives (4)** S98

6.- **NMR spectra of mononuclear orthopalladated derivatives (5)** and **(6)** S103

7.- **Absorption UV-Vis spectra** S108

8.- **Excitation-emission UV-Vis spectra** S114

9.- **Decay curves and fitting data for the determination of the half-life times of complexes 3,4,5,and 6** S122

10.- **Computational Results** S138
10.1.- Ground State S138
10.1.1. wB97XD (orbital energies and optimized geometries) S138
10.1.2. M06-2X (orbital energies and optimized geometries) S158
10.2. Absorption Properties S178
10.2.1. wB97XD (vertical excitations S178
10.2.2 M06-2X (vertical excitations) S179
10.3. Emission from S1 S180
10.3.1. wB97XD (vertical excitations and optimized geometries) S180
10.3.2. M06-2X (vertical excitations and optimized geometries) S200

10.4. Summary Table for wB97XD79 S220
10.5. T_1 vs S_0 relative energies S221

11. Crystallographic results S222

Table S10: Crystal data of compound 1a S222

Table S11: Crystal data of compound 1e S223

Discussion of the X-ray crystal structures of oxazolones 1a and 1e (Figures S1 and S2) S224

Table S12: Crystal data of compound 3c S226

Table S13: Crystal data of compound 3d S227

Table S14: Crystal data of compound 3g S228

Table S15: Crystal data of compound 7h S229

Discussion of the X-ray crystal structures of orthopalladated complexes 3c, 3d, 3g and 7h (Figures S3 - S7) S230

References corresponding to the Supporting Information S235

1.- Complete Experimental Section

General Information. Solvents were obtained from commercial sources and were used without further purification. All reactions were performed without special precautions against air and moisture. Electrospray ionization (ESI^+) mass spectra were recorded using Bruker Esquire3000 plus™ or Amazon Speed ion-trap mass spectrometers equipped with standard ESI sources. High-resolution mass spectra-ESI (HRMS-ESI) were recorded using either a Bruker MicroToF-Q™ system equipped with an API-ESI source and a Q-ToF mass analyzer, or a TIMS-TOF system, both allowing a maximum error in the measurement of 5 ppm. Acetonitrile was used as solvent. For all types of MS measurements, samples were introduced in a continuous flow of 0.2 mL/min and nitrogen served both as the nebulizer gas and the dry gas. The ^{1}H, ^{13}C and ^{19}F NMR spectra of the isolated products were recorded in $CDCl_3$, CD_2Cl_2 and dmso-d_6 solutions at 25 °C (other conditions were specified) on Bruker AV300, AV400 or Bruker AV500 spectrometers (δ in ppm, J in Hz) at ^{1}H operating frequencies of 300.13, 400.13 and 500.13 MHz, respectively. The ^{1}H and ^{13}C NMR spectra were referenced using the solvent signal as internal standard, while ^{19}F NMR spectra were referenced to $CFCl_3$. The assignment of ^{1}H NMR peaks has been performed through standard 2D ^{1}H−COSY (2K points in t_2 using a spectral width of 10 ppm; 128 t_1 experiments were recorded and zero-filled to 1K; for each t_1 value four scans were signal-averaged using a recycle delay of 1 s) and selective 1D ^{1}H-SELNOE experiments. Typical mixing times in the case of selective 1D-SELNOE experiments were in the range 1.2-1.8 s, as a function of the irradiated signal. These values of optimized mixing times were set equal to the longitudinal relaxation time T_1, determined using the inversion−recovery sequence. The ^{13}C NMR peaks were identified using standard ^{1}H−^{13}C edited-HSQC and ^{1}H−^{13}C HMBC 2D-experiments. In both cases 4K points in t_2 using spectral widths of 10 ppm (^{1}H) and 200 ppm (^{13}C) were used, with averaged values of the coupling constants $^{1}J_{CH}$ = 145 Hz and long-range $^{n}J_{CH}$ = 10 Hz. Typically, 128 t_1 experiments were recorded and zero-filled to 2K. For each t_1 value 8 (HSQC) or 32 (HMBC) scans were signal-averaged using a recycle delay of 1 s. Absorption spectra were measured on a Thermo Scientific Evolution 600BB spectrophotometer. The steady-state excitation–emission spectra were measured on a Jobin-Yvon Horiba Fluorolog FL-3-11 spectrofluorimeter. All measurements were carried out at room temperature on solutions of 10^{-5}M concentration using quartz cuvettes of 1 cm path length. The measurement of the quantum yield values (Φ_{PL}) was carried out using the absolute method on a Quantaurus-QY C11347 spectrometer. Lifetime measurements were carried out in a FluoTime 300 (PicoQuant) fluorescence spectrometer, using excitation LEDs of 450 nm. The measurement of the quantum yield values (Φ_{PL}) was carried out using the absolute method on a Quantaurus-QY C11347 spectrometer. Two different CH_2Cl_2 solutions of each compound (10^{-5} M) were measured in

order to check data reproducibility. In addition, one solution of **3d**, **4d** and **4h** was deoxygenated by passing argon through it, and the value of the QY was redetermined to check the influence of the O_2 in the intensity of the luminescence. The oxazolones **1a-1m** were prepared using the Erlenmeyer–Plöchl method, by reaction of the corresponding hippuric acids and aldehydes in acetic anhydride.[1] The hippuric acids were prepared by the Schotten–Baumann method.[2]

X-ray Crystallography and Structural Data. Single crystals of **1a, 1e, 3c, 3d, 3g** and **7h**·$CHCl_3$ of suitable quality for X-ray diffraction measurements were grown by slow diffusion of *n*-pentane into CH_2Cl_2 or $CHCl_3$ solutions of the crude products at –18 °C for several weeks, except for **7h**. Crystals of **7h** were obtained when **6h** was left to crystallize in $CHCl_3$, due to the presence of residual HCl in the chlorinated solvent. One selected single crystal of each compound was mounted at the end of a quartz fiber in a random orientation, covered with perfluorinated oil (magic oil) and placed under a cold stream of N_2 gas. Crystallographic measurements were carried out at 100 K on a Bruker APEXD8 Venture CCD diffractometer, using graphite monochromated Mo Kα radiation (λ = 0.71073 Å). A hemisphere of data was collected in each case based on ω-scan or ϕ-scan runs. The diffraction frames were integrated using the program SAINT[3] and the integrated intensities were corrected for absorption with SADABS.[4] The structures were solved by direct methods with SHELXT-2014.[5] All non-hydrogen atoms were refined with anisotropic displacement parameters. The hydrogen atoms were placed at idealized positions and treated as riding atoms. Each hydrogen atom was assigned an isotropic displacement parameter equal to 1.2–1.5 times the equivalent isotropic displacement parameter of its parent atom. For structure solving and refinement, the SHELXL-2016[6] program in the WINGX Package was used.[7] The structures were refined to F_o^2, and all reflections were used in the least-squares calculations. CCDC-2122909 (**1a**), CCDC-2122910 (**1e**), CCDC-2122911 (**3c**), CCDC-2122912 (**3d**), CCDC-2122913 (**3g**) and CCDC-2145279 (**7h**) contain the supplementary crystallographic data. These data can be obtained free of charge from the Cambridge Crystallographic Data Centre via www.ccdc.cam.ac.uk/data_request/cif.

Computational Details. All calculations were carried out within the Density Functional Theory (DFT)[8], using the Gaussian16 program package.[9] First, in order to characterize the ground electronic state of the selected complexes, geometry optimizations and harmonic frequency calculations were performed by using the wB97XD[10] and M06-2X[11] exchange-correlation functionals, combined with the 6-31+G(d,p) basis set for the non-metal atom,[12] and the ECP10MDF Sttutgart-Cologne relavistic core potentials along with the aug-cc-pVDZ-PP basis set for Pd atom.[13] Solvent effects (dichloromethane) were taken into account by means of the integral equation formalism of the polarized

continuum model (IEFPCM)[14] Finally, in order to study the photoabsorption and photoemission processes, Time-Dependent Density Functional Theory (TDDFT)[15] was used, at the same levels of theory used in the characterization of the ground states. Vertical transitions were calculated for absorption properties, and geometry optimizations and frequency calculations were carried out for the first singlet and triplet excited states in order to account for emission properties. Both ωB97XD and M06-2X provide similar qualitative and quantitative values for both absorption and emission (see Tables S1 to S8). In order to choose one functional, the absorption wave lengths have been calculated and compared to the experimental values for a group of selected complexes. The obtained results are collected in Table S1. It may be observed that both functionals provide similar results for all complexes. Nevertheless, comparing the differences with respect to the experimental value (D1 for wB97XD and D2 for M06-2X) it may be seen that systematically M06-2X is closer to the experimental value. The calculated average D1 (54 nm) and D2 (48 nm) values confirm that indeed M06-2X is slightly closer than ωB97XD from the experimental value by 6 nm. Hence, for the sake of clarity hereafter only the M06-2X values will be considered. The molecular orbitals representations were generated by *PyMOL*[16] using Paton Research Group openly accessible display settings.[17]

Table S1: *Experimental and calculated absorption wavelengths for both ωB97XD and M06-2X functionals, and their differences (D1 for ωB97XD and D2 for M06-2X) with respect to the experimental values, in nm, for a selected group of complexes. The average difference is provided for both functionals.*

Absorption wave lengths (nm)					
**Compound**	**wB97XD**	**D_1**	**M06-2X**	**D_2**	**Exp**
**3a**	396	72	405	63	468
**3b**	424	61	424	61	485
**3c**	446	70	453	63	516
**3d**	406	44	413	37	450
**3e**	410	47	417	40	457
**3f**	419	55	429	45	474
**3g**	405	40	407	38	445
**3h**	429	46	431	44	475
**3i**	412	62	412	62	474
**3j**	406	18	406	18	424
**3k**	396	49	409	36	445
**3m**	437	49	438	48	486
**4d**	407	64	411	60	471
**4h**	429	59	434	54	488
**6h**	429	52	432	49	481
**7h**	429	54	438	45	483
**Av.**	-	53	-	48	

General Synthesis and Characterization of Oxazolones 1a-1m. The oxazolones **1a**,[18a] **1b**,[18b] **1c**,[18c] **1d**,[18d] **1f**,[18e] **1g**[18f] and **1h**[19a] appear on Scifinder as previously reported. They were characterized by comparison of their NMR data with those previously published. The oxazolones **1e** and **1i-1m** have not been previously reported, or they appear on Scifinder without references associated, so they have been fully characterized here. The oxazolones were prepared following the Erlenmeyer-Plöchl method,[1] which is exemplified here with the detailed synthesis of **1a**.

*Synthesis of (Z)-4-((9H-fluoren-3-yl) methylene)-2-phenyl-5(4H)-oxazolone (**1a**).*[18a] Sodium acetate (420.0 mg, 5.15 mmol) and fluorene-2-carboxaldehyde (1000.0 mg, 5.15 mmol) were added to a solution of hippuric acid (923.0 mg, 5.15 mmol) in acetic anhydride (10 mL). The suspension was heated to the reflux temperature (100 ^{o}C) for 3 h, and then was allowed to cool to room temperature. The solid mass formed upon cooling was treated with distilled water (30 mL) to give **1a** as a yellow solid, which was filtered off, washed with water (5 mL) and cold ethanol (10 mL), and dried under vacuum. Obtained: 1450.0 mg (84% yield).

*Synthesis of (Z)-4-(naphthalen-1-ylmethylene)-2-phenyl-5(4H)-oxazolone (**1b**).*[18b] Oxazolone **1b** was prepared following the same procedure than that reported for **1a**. Therefore, sodium acetate (2630.0 mg, 32.0 mmol), 1-naphthylaldehyde (5000.0 mg, 32.0 mmol) and hippuric acid (5740.0 mg, 32.0 mmol) reacted in acetic anhydride (20 mL) at the reflux temperature to give **1b** as an orange solid. Obtained: 4880.0 mg (51% yield).

*Synthesis of (Z)-2-phenyl-4-(pyren-1-ylmethylene)-5(4H)-oxazolone (**1c**).*[18c] Oxazolone **1c** was prepared following the same procedure than that reported for **1a**. Therefore, sodium acetate (891.0 mg, 10.86 mmol), pyrene-1-carbaldehyde (2500.0 mg, 10.86 mmol) and hippuric acid (1950.0 mg, 10.86 mmol) reacted in acetic anhydride (10 mL) at the reflux temperature to give **1c** as an orange solid. Obtained: 3050.0 mg (75% yield).

*Synthesis of (Z)-4-(2,4-dimethoxybenzylidene)-2-phenyl-5(4H)-oxazolone (**1d**).*[18d] Oxazolone **1d** was prepared following the same procedure than that reported for **1a**. Therefore, sodium acetate (1640.0 mg, 20.00 mmol), 2,4-dimethoxybenzaldehyde (3330.0 mg, 20.00 mmol) and hippuric acid (3583.6 mg, 20.00 mmol) reacted in acetic anhydride (10 mL) at the reflux temperature to give **1d** as a yellow-orange solid. Obtained: 3210.0 mg (52% yield).

*Synthesis of (Z)-4-(2,4-dimethoxybenzylidene)-2-(pentafluorophenyl)-5(4H)-oxazolone (**1e**).* Oxazolone **1e** was prepared following the same procedure than that reported for **1a**. Thus, sodium acetate (305.0 mg, 3.72 mmol), 2,4-dimethoxybenzaldehyde (617.0 mg, 3.72 mmol) and pentafluorobenzoylglycine (1000.0 mg, 3.72 mmol) reacted in acetic anhydride (5 mL) at the reflux temperature to give **1e** as an orange solid. Obtained: 230.0 mg (16% yield). This product was crystallized from CH_2Cl_2/pentane and, depending of the batch, the obtained crystals showed a small

amount of CH_2Cl_2 of crystallization. ^1H NMR ($CDCl_3$, 300.13 MHz): δ 8.78 (d, 1H, J = 8.9 Hz, H_6, C_6H_3), 7.97 (s, 1H, =CH_{vinyl}), 6.63 (dd, 1H, J = 8.9 Hz, J = 2.4 Hz, H_5, C_6H_3,), 6.44 (d, 1H, J = 2.4 Hz, H_3, C_6H_3), 3.91 (s, 3H, OMe), 3.90 (s, 3H, OMe). $^{13}C\{^1H\}$ NMR ($CDCl_3$, 75.47 MHz): δ 165.4 (C=O), 165.2 (C=N), 162.0 (overlaped C_4-OMe and C_2-OMe, C_6H_3), 157.8, 154.7, 148.0, 144. 6 (4m, 4C, C_6F_5), 135.2 (C_6, C_6H_3), 130.2 (=CH, C_{vinyl}), 127.8 (=C), 115.9 (C_1, C_6H_3), 106.9 (C_5, C_6H_3), 98.3 (C_3, C_6H_3), 55.9 (OMe), 55.8 (OMe). ^{19}F NMR ($CDCl_3$, 282.40 MHz): δ -134.96 (m, 2F, F_o, C_6F_5), -147.23 (tt, 1F, J = 21 Hz, J = 5 Hz, F_p, C_6F_5), -160.10 (m, 2F, F_m, C_6F_5). MS (ESI) *m/z*: $[M-CH_3OH-CH_3O]^+$ Calcd for $[C_{16}H_3F_5NO_2]$: 336.0; found 336.1. Elem. Anal. Calc for $[C_{18}H_{10}F_5NO_4]\cdot 0.15CH_2Cl_2$: C, 52.91; H, 2.52; N, 3.40. Found: C, 53.12; H, 2.53; N, 3.57.

*Synthesis of (Z)-4-(2,4-dimethoxybenzylidene)-2-(4-nitrophenyl)-5(4H)-oxazolone (**1f**).*[18e] Oxazolone **1f** was prepared following the same procedure than that reported for **1a**. Thus, sodium acetate (366.0 mg, 4.46 mmol), 2,4-dimethoxybenzaldehyde (741.0 mg, 4.46 mmol) and 4-nitrohippuric acid (1000.0 mg, 4.46 mmol) reacted in acetic anhydride (5 mL) at the reflux temperature to give **1f** as a red solid. Obtained: 729.0 mg (46% yield).

*Synthesis of 4-((Z)-4-methylbenzylidene)-2-((E)-styryl)-5(4H)-oxazolone (**1g**).*[18f] Oxazolone **1g** was prepared following the same procedure than that reported for **1a**. Thus, sodium acetate (410.0 mg, 4.88 mmol), 4-methylbenzaldehyde (0.54 mL, 4.88 mmol) and *N*-cinnamoylglycine (1000.0 mg, 4.88 mmol) reacted in acetic anhydride (8 mL) at the reflux temperature to give **1g** as a red solid. Obtained: 400.0 mg (28% yield).

*Synthesis of 4-((Z)-2,4-dimethoxybenzylidene)-2-((E)-styryl)-5(4H)-oxazolone (**1h**).*[19a] Oxazolone **1h** was prepared following the same procedure than that reported for **1a**. Thus, sodium acetate (820.0 mg, 9.99 mmol), 2,4-dimethoxybenzaldehyde (1660.0 mg, 9.99 mmol) and *N*-cinnamoylglycine (2000.0 mg, 9.75 mmol) reacted in acetic anhydride (10 mL) at the reflux temperature to give **1h** as a red solid. Obtained: 1220.0 mg (36% yield).

*Synthesis of (Z)-4-((9H-fluoren-2-yl)methylene)-2-((E)-styryl)-5(4H)-oxazolone (**1i**).* Oxazolone **1i** was prepared following the same procedure than that reported for **1a**. Thus, sodium acetate (420.0 mg, 5.12 mmol), fluorene-2-carboxaldehyde (1000.0mg, 5.15 mmol) and *N*-cinnamoylglycine (1056.0 mg, 5.15 mmol) reacted in acetic anhydride (8 mL) at the reflux temperature to give **1i** as a red solid. Obtained: 860.0 mg (46% yield). ^1H NMR ($CDCl_3$, 300.13 MHz): δ 8.41 (s, 1H, H_2, $C_{13}H_9$), 8.07 (d, 1H, J = 9 Hz, H_{aro}, $C_{13}H_9$), 7.84 (2d, 2H overlaped, H_{aro}, $C_{13}H_9$), 7.69 (d, 1H, J = 16.2 Hz, =CH_{olef}), 7.62-7.58 (m, 3H, H_{aro}, $C_{13}H_9$ + C_6H_5), 7.45-7.37 (m, 5H, H_{aro}, $C_{13}H_9$ + C_6H_5), 7.27 (s, 1H, =CH_{vinyl}), 6.86 (d, 1H, J = 16.2 Hz, =CH_{olef}), 3.98 (s, 2H, CH_2, $C_{13}H_9$). $^{13}C\{^1H\}$ NMR ($CDCl_3$, 75.47 MHz): δ 167.6 (C=O), 163.1 (C=N), 145.1 (C_q, C_{aro}), 144.6 (C_q, C_{aro}), 143.9 (C_q, C_{aro}), 143.6 (=CH, C_{olef}), 140.9 (C_q, C_{aro}), 134.8 (C_i, C_6H_5), 132.8 (C_q, C_{aro}), 132.3 (C_q, C_{aro}), 132.1 (CH_{aro}), 132.1 (CH_{aro}), 130.8 (CH_{aro}), 129.2 (C_o, C_6H_5), 128.7 (CH_{aro}), 128.3 (C_m, C_6H_5), 128.1 (CH_{aro}), 127.2 (CH_{aro}), 125.4

(CH_{aro}), 120.9 (CH_{aro}), 120.4 (CH_{aro}), 113.6 (=CH, C_{olef}), 37.03 (CH_2). HRMS (ESI) *m/z*: $[M+Na]^+$ Calcd for $[C_{25}H_{17}NNaO_2]^+$ 386.1151. Found 386.1147.

*Synthesis of 4-((Z)-2,3-dimethoxybenzylidene)-2-((E)-styryl)-5(4H)-oxazolone (**1j**)*. Oxazolone **1j** was prepared following the same procedure than that reported for **1a**. Thus, sodium acetate (494.0 mg, 6.02 mmol), 2,3-dimethoxybenzaldehyde (1000.0mg, 6.02 mmol) and *N*-cinnamoylglycine (1235.0 mg, 6.02 mmol) reacted in acetic anhydride (10 mL) at the reflux temperature to give **1j** as a red solid. Obtained: 587.0 mg (29% yield). ^{1}H NMR ($CDCl_3$, 300.13 MHz): δ 8.33 (dd, 1H, J = 8.0 Hz, J = 1.4 Hz, H_6, C_6H_3), 7.71 (s, 1H, =CH_{vinyl}), 7.70 (d, 1H, J = 16.2 Hz, =CH_{olef}), 7.59 (m, 2H, H_o, C_6H_5), 7.44–7.41 (m, 3H, H_m, H_p, C_6H_5), 7.17 (t, 1H, J = 8.0 Hz, H_5, C_6H_3), 7.01 (dd, 1H, J = 8.0 Hz, J = 1.4 Hz, H_4, C_6H_3), 6.82 (d, 1H, J = 16.2 Hz, =CH_{olef}), 3.93 (s, 3H, OMe), 3.90 (s, 3H, OMe). $^{13}C\{^1H\}$ NMR ($CDCl_3$, 75.47 MHz): δ 167.5 (C=O), 163.5 (C=N), 152.8 (C–O, C_6H_3), 149.9 (C–O, C_6H_3), 143.9 (=CH, C_{olef}), 134.7 (C_i, C_6H_5), 134.1 (=C), 130.9 (C_p, C_6H_5), 129.2 (C_m, C_6H_5), 128.3 (C_o, C_6H_5), 127.9 (C_1, C_6H_3), 125.6 (=CH, C_{vinyl}), 124.4 (C_5, C_6H_3), 124.1 (C_6, C_6H_3), 115.4 (C_4, C_6H_3), 113.6 (=CH, C_{olef}), 62.0 (s, OMe), 56.0 (s, OMe). HRMS (ESI) *m/z*: $[M+Na]^+$ Calcd for $[C_{20}H_{17}NNaO_4]^+$ 358.1050. Found 358.1052.

*Synthesis of 4-((Z)-3,5-difluorobenzylidene)-2-((E)-styryl) oxazol-5(4H)-one (**1k**)*. Oxazolone **1k** was prepared following the same procedure than that reported for **1a**. Thus, sodium acetate (820.0 mg, 9.99 mmol), 3,5-difluorobenzaldehyde (1000.0mg, 7.03 mmol) and *N*-cinnamoylglycine (1440.0 mg, 7.03 mmol) reacted in acetic anhydride (10 mL) at the reflux temperature to give **1k** as a red solid. Obtained: 1000.0 mg (46% yield). ^{1}H NMR ($CDCl_3$, 300.13 MHz): δ 7.75 (d, 1H, J = 16.2 Hz, =CH_{olef}), 7.69 (dd, 2H, J = 8.4 Hz, J = 2.1 Hz, H_o, $C_6H_3F_2$), 7.61 (m, 2H, H_o, C_6H_5), 7.46–7.44 (m, 3H, H_m, H_p, C_6H_5), 7.04 (s, 1H, =CH_{vinyl}), 6.88 (tt, 1H, J = 8.6 Hz, J = 2.3 Hz, H_p, $C_6H_3F_2$), 6.84 (d, 1H, J = 16.2 Hz, =CH_{olef}). $^{13}C\{^1H\}$ NMR ($CDCl_3$, 75.47 MHz): δ 166.8 (C=O), 164.7 (C=N), 163.1 (dd, $^1J_{CF}$ = 248.9 Hz, $^3J_{CF}$ = 12.8 Hz, $C_{3,5}$–F, $C_6H_3F_2$), 144.3 (=CH, C_{olef}), 136.4 (t, $^3J_{CF}$ = 9.8 Hz, C_1, $C_6H_3F_2$), 135.7 (=C), 134.5 (C_i, C_6H_5), 131.3 (C_p, C_6H_5), 129.3 (C_m, C_6H_5), 128.5 (C_o, C_6H_5), 127.8 (t, $^4J_{CF}$ = 3.3 Hz, =CH, C_{vinyl}), 114.6 (d, $^2J_{CF}$ = 25.8 Hz, $C_{2,6}$, $C_6H_3F_2$), 113.1 (=CH, C_{olef}), 106.3 (t, $^2J_{CF}$ = 25.6 Hz, C_4, $C_6H_3F_2$). ^{19}F NMR ($CDCl_3$, 282.40 MHz): δ –108.97 (pseudot, 2F, J = 8.3 Hz). HRMS (ESI) *m/z*: $[M+Na]^+$ Calcd for $[C_{18}H_{11}F_2NNaO_2]^+$ 334.0650. Found 334.0639.

*Synthesis of 4-((Z)-2,5-dimethoxybenzylidene)-2-((E)-styryl)-5(4H)-oxazolone (**1l**)*. Oxazolone **1l** was prepared following the same procedure than that reported for **1a**. Thus, sodium acetate (494.0 mg, 6.02 mmol), 2,5-dimethoxybenzaldehyde (1000.0mg, 6.02 mmol) and *N*-cinnamoylglycine (1235.0 mg, 6.02 mmol) reacted in acetic anhydride (10 mL) at the reflux temperature to give **1l** as a red solid. Obtained: 753.0 mg (37% yield). ^{1}H NMR ($CDCl_3$,

300.13 MHz): δ 8.39 (d, 1H, J = 3.1 Hz, H_6, C_6H_3), 7.76 (s, 1H, $=CH_{vinyl}$), 7.67 (d, 1H, J = 16.2 Hz, $=CH_{olef}$), 7.58 (m, 2H, H_o, C_6H_5), 7.45–7.41 (m, 3H, H_m, H_p, C_6H_5), 6.99 (dd, 1H, J = 9.0 Hz, J = 3.1 Hz, H_4, C_6H_3), 6.85 (d, 1H, J = 9.0 Hz, H_3, C_6H_3), 6.80 (d, 1H, J = 16.2 Hz, $=CH_{olef}$), 3.87 (s, 3H, OMe), 3.86 (s, 3H, OMe). $^{13}C\{^1H\}$ NMR ($CDCl_3$, 75.47 MHz): δ 167.6 (C=O), 163.0 (C=N), 154.2 (C–O, C_6H_3), 153.7 (C–O, C_6H_3), 143.4 (=CH, C_{olef}), 134.8 (C_i, C_6H_5), 133.0 (=C), 130.8 (C_p, C_6H_5), 129.2 (C_m, C_6H_5), 128.3 (C_o, C_6H_5), 125.4 (=CH, C_{vinyl}), 123.2 (C_1, C_6H_3), 119.6 (C_4, C_6H_3), 116.6 (C_6, C_6H_3), 113.7 (=CH, C_{olef}), 112.0 (C_3, C_6H_3), 56.3 (s, OMe), 56.0 (s, OMe). HRMS (ESI) *m/z*: $[M+Na]^+$ Calcd for $[C_{20}H_{17}NNaO_4]^+$ 358.1049. Found 358.1046.

*Synthesis of 4-((Z)-3,4-dimethoxybenzylidene)-2-((E)-styryl)-5(4H)-oxazolone (**1m**)*. Oxazolone **1m** was prepared following the same procedure than that reported for **1a**. Thus, sodium acetate (494.0 mg, 6.02 mmol), 3,4-dimethoxybenzaldehyde (1000.0mg, 6.02 mmol) and *N*-cinnamoylglycine (1235.0 mg, 6.02 mmol) reacted in acetic anhydride (10 mL) at the reflux temperature to give **1g** as a red solid. Obtained: 603.0 mg (30% yield). ^1H NMR ($CDCl_3$, 300.13 MHz): δ 8.01 (d, 1H, J = 2.0 Hz, H_2, C_6H_3), 7.67 (d, 1H, J = 16.2 Hz, $=CH_{olef}$), 7.64-7.54 (m, 3H, H_o, C_6H_5, H_5, C_6H_3), 7.45–7.41 (m, 3H, H_m, H_p, C_6H_5), 7.15 (s, 1H, $=CH_{vinyl}$), 6.93 (d, 1H, J = 8.4 Hz, H_6, C_6H_3), 6.80 (d, 1H, J = 16.2 Hz, $=CH_{olef}$), 4.00 (s, 3H, OMe), 3.96 (s, 3H, OMe). $^{13}C\{^1H\}$ NMR ($CDCl_3$, 75.47 MHz): δ 167.7 (C=O), 162.5 (C=N), 152.2 (C–O, C_6H_3), 149.4 (C–O, C_6H_3), 143.2 (=CH, C_{olef}), 134.9 (2C overlapped, C_i, C_6H_5, =C), 131.7 (=CH, C_{vinyl}), 130.7 (C_p, C_6H_5), 129.2 (C_m, C_6H_5), 128.2 (C_o, C_6H_5), 127.7 (C_5, C_6H_3), 127.1 (C_1, C_6H_3), 114.1 (C_2, C_6H_3), 113.7 (=CH, C_{olef}), 111.1 (C_6, C_6H_3), 56.2 (s, OMe), 56.1 (s, OMe). HRMS (ESI) *m/z*: $[M+Na]^+$ Calcd for $[C_{20}H_{17}NO_4Na]^+$ 358.1050. Found 358.1061.

General Synthesis and Characterization of Orthopalladated Dimers with Trifluoroacetate Bridges 2. The ortopalladated dimers **2d**[19b] and **2h**[19a] have been previously described. They were characterized by comparison of their NMR data with those previously published. Orthopalladated complexes **2a-2k** and **2m** have been obtained following the same synthetic procedure, which is detailed here for the synthesis of **2a**. For all compounds **2**, and despite the use of long accumulation times, signals due to the ^{13}C nuclei of the CF_3COO ligand and the C_6F_5 group were not observed in the ^{13}C NMR spectra, due to multiple ^{13}C-^{19}F couplings and to the low solubility of the compounds.

*Synthesis of Orthopalladated **2a***. $Pd(OAc)_2$ (200.0 mg, 0.89 mmol) was added to a solution of **1a** (300.0 mg, 0.89 mmol) in CF_3CO_2H (5 mL). The resulting mixture was heated in an oil bath to the reflux temperature of the solvent (72.4 °C) for 3 h. After the reaction time, the resulting mixture was cooled to room temperature and distilled water (10 mL) was added. The resulting precipitate was filtered off, washed with more distilled water (3×10 mL) until the characteristic smell of trifluoroacetic acid disappeared, dried under vacuum, and identified as **2a** (orange solid). Obtained: 455.0 mg (92% yield). ^1H NMR ($CDCl_3$, 300.13 MHz): δ 7.68–7.63 (m, 2H, $C_{13}H_8$), 7.60 (m, 2H, H_o, C_6H_5), 7.57 (s, 1H, $=CH_{vinyl}$), 7.56,

7.52 (2s, 2H, H_2, H_4, $C_{13}H_8$), 7.47-7.43 (m, 3H, H_p, C_6H_5, $C_{13}H_8$), 7.21 (m, 2H, H_m, C_6H_5), 4.10, 3.99 (AB spin system, 2H, J = 22 Hz, CH_2, $C_{13}H_8$). $^{13}C\{^1H\}$ NMR ($CDCl_3$, 75.47 MHz): δ 167.6 (C=N), 161.1 (C=O), 145.4 (C_q, $C_{13}H_8$), 145.3(C_q, $C_{13}H_8$), 141.3 (C_q, $C_{13}H_8$), 140.5 (C_q, $C_{13}H_8$), 139.2 (=CH, C_{vinyl}), 135.1 (=C), 134.5 (C_p, C_6H_5), 130.6 (C_o, C_6H_5), 129.8 (C_3, $C_{13}H_8$), 128.8 (CH, $C_{13}H_8$), 128.4 (C_m, C_6H_5), 128.0 (C_i , C_6H_5), 127.5 (CH, $C_{13}H_8$), 125.6 (CH, $C_{13}H_8$), 125.0 (C_5, $C_{13}H_8$), 122.5 (C_2, $C_{13}H_8$), 122.4 (C_q, C-Pd), 121.2 (CH, $C_{13}H_8$), 36.1 (CH_2, $C_{13}H_8$). ^{19}F NMR ($CDCl_3$, 282.40 MHz): δ −74.98. HRMS (ESI) *m/z*: $[M–CF_3COO]^+$ Calcd for $[C_{48}H_{28}F_3N_2O_6Pd_2]^+$ 998.9973. Found 998.9965.

*Synthesis of Orthopalladated **2b***. Following the same synthetic procedure than the described for **2a**, $Pd(OAc)_2$ (233.0 mg, 1.04 mmol) was reacted with **1b** (300.0 mg, 1.00 mmol) in CF_3CO_2H (5 mL) at 72.4 °C to give **2b** as a red solid. Complex **2b** was obtained as a mixture of the *trans* and *cis* isomers, in molar ratio 1/0.4. Only the major *trans* isomer could be fully characterized. Obtained: 480.0 mg (93% yield). ^1H NMR ($CDCl_3$, 300.13 MHz): δ 8.40 (s, 1H, =CH_{vinyl}), 8.37 (m, 1H, $C_{10}H_6$), 7.92 (d, 1H, J = 7.8 Hz, $C_{10}H_6$), 7.79 (t, 1H, J = 6.8 Hz, $C_{10}H_6$), 7.74 (m, 2H, H_o, C_6H_5), 7.66-7.50 (m, 4H, $3H_{ar}$, $C_{10}H_6$, H_p, C_6H_5), 7.41 (m, 2H, H_m, C_6H_5). $^{13}C\{^1H\}$ NMR ($CDCl_3$, 75.47 MHz): δ 167.7 (C=N), 160.3 (C=O), 142.0 (C_q, $C_{10}H_6$), 134.7 (C_p, C_6H_5), 132.3 (=CH, C_{vinyl}), 132.2 (=C), 131.6 (C_q, $C_{10}H_6$), 131.3 (CH, $C_{10}H_6$), 131.0 (CH, $C_{10}H_6$), 130.6 (C_o, C_6H_5), 128.9 (CH, $C_{10}H_6$), 128.3 (C_m, C_6H_5), 128.1 (CH, $C_{10}H_6$), 126.3 (CH, $C_{10}H_6$), 124.8 (C_q, $C_{10}H_6$), 122.7 (C_q, $C_{10}H_6$), 122.0 (C_i , C_6H_5), 121.6 (CH, $C_{10}H_6$). ^{19}F NMR ($CDCl_3$, 282.40 MHz): δ −73.66, −74.56 (minor *cis*-isomer), −75.05 (major *trans*-isomer). HRMS (ESI) *m/z*: $[M+Na]^+$ Calcd for $[C_{44}H_{24}F_6N_2NaO_8Pd_2]^+$ 1058.9408. Found 1058.9428.

*Synthesis of Orthopalladated **2c***. Following the same synthetic procedure than described for **2a**, $Pd(OAc)_2$ (233.0 mg, 1.04 mmol) was reacted with **1c** (388.0 mg, 1.04 mmol) in CF_3CO_2H (5 mL) at 72.4 °C to give **2c** as a red solid. Obtained: 610 mg (99% yield).^1H NMR (DMSO-d_6, 300.13 MHz): δ 8.93 (d, 1H, J=9.4 Hz, $C_{16}H_8$), 8.85 (s, 1H, =CH_{vinyl}), 8.48 (m, 2H, H_o, C_6H_5), 8.40-8.34 (m, 3H, H_m, H_p, C_6H_5), 8.28i (d, 1H, J=9.4 Hz, $C_{16}H_8$), 8.20 (m, 1H, $C_{16}H_8$), 8.11-8.04 (m, 2H, $C_{16}H_8$), 7.83 (m, 1H, $C_{16}H_8$), 7.75-7.70 (m, 2H, $C_{16}H_8$). $^{13}C\{^1H\}$ NMR (DMSO-d_6, 75.47 MHz): δ 166.7 (C=N), 161.2 (C=O), 138.3 (=C), 134.7 (CH, $C_{16}H_8$), 132.7 (=CH, C_{vinyl}), 131.9 (C_q, $C_{16}H_8$), 131.8 (CH, $C_{16}H_8$), 130.7 (C_q, $C_{16}H_8$), 130.4 (CH, $C_{16}H_8$), 130.3 ($2C_q$ overlaped, $C_{16}H_8$), 130.1 (C_o, C_6H_5), 129.7 (C_i, C_6H_5), 129.3 (C_p, C_6H_5), 128.8 (C_m, C_6H_5), 126.9 (CH, $C_{16}H_8$), 126.7 (CH, $C_{16}H_8$), 126.6 (CH, $C_{16}H_8$), 126.4 (CH, $C_{16}H_8$), 124.6 (C_q, $C_{16}H_8$), 123.6 (C_q, $C_{16}H_8$), 123.0 (C_q, $C_{16}H_8$), 122.4 (C_q, $C_{16}H_8$), 122.3 (CH, $C_{16}H_8$). ^{19}F NMR (DMSO-d_6, 282.40 MHz): δ −74.19. HRMS (ESI) *m/z*: $[M+Na]^+$ Calcd for $[C_{56}H_{28}F_6N_2NaO_8Pd_2]^+$ 1206.9721. Found 1206.9732.

*Synthesis of Orthopalladated **2d**.*[19b] Following the same synthetic procedure than described for **2a**, $Pd(OAc)_2$ (233 mg, 1.04 mmol) was reacted with **1d** (321.2 mg, 1.04 mmol) in CF_3CO_2H (5 mL) at 72.4 °C to give **2d** as a red solid. Obtained: 467.0 mg (85% yield).

*Synthesis of Orthopalladated **2e***. Following the same synthetic procedure than described for **2a**, $Pd(OAc)_2$ (117 mg, 0.52 mmol) was reacted with **1e** (208 mg, 0.52 mmol) in CF_3CO_2H (5 mL) at 72.4 °C to give **2e** as a red solid. Obtained: 308 mg (96% yield). ^{1}H NMR ($CDCl_3$, 300.13 MHz): δ 8.25 (s, 1H, =CH_{vinyl}), 6.24, 6.19 (AB spin system, 2H, $^4J_{HH}$ = 2.0 Hz, H_3 + H_5, C_6H_2), 3.96 (s, 3H, OMe), 3.79 (s, 3H, OMe). $^{13}C\{^1H\}$ NMR ($CDCl_3$, 75.47 MHz): δ 164.0 (C-OMe, C_6H_2), 163.0 (C=N), 160.6 (C-OMe, C_6H_2), 159.7 (C=O), 141.3 (=C), 136.1 (=CH, C_{vinyl}), 116.5 (C_1-Pd, C_6H_2), 113.3 (C_2, C_6H_2), 110.2, 96.7 (C_4, C_6, C_6H_2), 56.2 (s, OMe), 55.6 (s,OMe). ^{19}F NMR ($CDCl_3$, 282.40 MHz): δ −75.20 (s,3F, CF_3), -131.97 (AA' part, AA'BB'C spin system, 1F, F_o, C_6F_5), -137.51 (AA' part, AA'BB'C spin system, 1F, F_o, C_6F_5), -144.71 (pseudott, 1F, J = 20.8 Hz, J = 5.2 Hz, F_p, C_6F_5), -159.03 (BB' part, AA'BB'C spin system, 1F, F_m, C_6F_5), -161.05 (BB' part, AA'BB'C spin system, 1F, F_m, C_6F_5). HRMS (ESI) *m/z*: $[M+Na]^+$ Calcd for $[C_{40}H_{18}F_{16}N_2NaO_{12}Pd_2]^+$ 1258.8576. Found 1258.8587.

*Synthesis of Orthopalladated **2f***. Following the same synthetic procedure than described for **2a**, $Pd(OAc)_2$ (233 mg, 1.04 mmol) was reacted with **1f** (368 mg, 1.04 mmol) in CF_3CO_2H (5 mL) at 72.4 °C to give **2f** as a red solid. Obtained: 532 mg (90% yield).^{1}H NMR ($CDCl_3$, 300.13 MHz): δ 8.30 (m, 2H, H_m, $C_6H_4NO_2$), 8.16 (s, 1H, =CH_{vinyl}), 8.14 (m, 2H, H_o, $C_6H_4NO_2$), 6.30, 6.23 (AB spin system, 2H, $^4J_{HH}$ = 2.1 Hz, H_3 + H_5, C_6H_2), 4.01 (s, 3H, OMe), 3.77 (s, 3H, OMe).$^{13}C\{^1H\}$ NMR ($CDCl_3$, 75.47 MHz): δ 163.1 (C=N), 163.0 (C-OMe, C_6H_2), 160.0 (C-OMe, C_6H_2), 160.0 (C=O), 150.6 (C-NO_2, $C_6H_4NO_2$), 141.1 (=C), 134.9 (=CH, C_{vinyl}), 131.3 (C_m, $C_6H_4NO_2$), 128.7 (C_i, $C_6H_4NO_2$), 123.6 (C_o, $C_6H_4NO_2$), 118.5 (C_1-Pd, C_6H_2), 114.0 (C_2, C_6H_2), 109.8, 96.9 (C_4+C_6, C_6H_2), 56.3 (OMe), 55.8 (OMe). ^{19}F NMR (376 MHz, $CDCl_3$): δ −75.14. HRMS (ESI) *m/z*: $[M-CF_3COO]^+$ Calcd for $[C_{38}H_{26}F_3N_4O_{14}Pd_2]^+$ 1032.9471. Found 1032.9475.

*Synthesis of Orthopalladated **2g***. Following the same synthetic procedure than described for **2a**, $Pd(OAc)_2$ (233.0 mg, 1.04 mmol) was reacted with **1g** (290.0 mg, 1.0 mmol) in CF_3CO_2H (5 mL) at 72.4 °C to give **2g** as a red solid. Obtained: 486 mg (96% yield). ^{1}H NMR ($CDCl_3$, 300.13 MHz): δ 7.52 (m, 2H, H_o, C_6H_5), 7.46-7.41 (m, 3H, H_m+H_p, C_6H_5), 7.36 (d, 1H, J = 16 Hz, =CH_{olef}), 7.25 (s, 1H, =CH_{vinyl}), 7.05 (d, 1H, J = 16 Hz, =CH_{olef}), 7.04 (d, 1H, J=7.4 Hz, H_3, C_6H_3), 6.87 (d, 1H, J = 8 Hz, H_4, C_6H_3), 6.85 (s, br, 1H, H_6, C_6H_3), 1.98 (s, 3H, Me). $^{13}C\{^1H\}$ NMR ($CDCl_3$, 75.47 MHz): δ 165.3 (C=N), 160.9 (C=O), 147.9 (=CH, C_{olef}), 142.6 (C_5-Me, C_6H_3), 136.6 (=CH, C_{vinyl}), 135.1 (=C), 134.3, 127.4 (C_4/C_6, C_6H_3), 133.9 (C_i, C_6H_5), 133.1 (C_3, C_6H_3), 132.2 (C_p, C_6H_5), 130.0 (C_m, C_6H_5), 129.2 (C_o, C_6H_5), 127.0 (C_1-Pd, C_6H_3), 121.8 (C_2, C_6H_3), 110.4 (=CH, C_{olef}),

21.8 (Me). ^{19}F NMR ($CDCl_3$, 282.40 MHz): δ −74.93. HRMS (ESI) *m/z*: $[M\text{-}CF_3COO]^+$ Calcd for $[C_{40}H_{28}F_3N_2O_6Pd_2]^+$ 902.9973. Found 902.9963.

*Synthesis of Orthopalladated **2h***.[19a] Following a similar synthetic procedure than described for **2a**, $Pd(OAc)_2$ (233 mg, 1.04 mmol) was reacted with **1h** (335.0 mg, 1.0 mmol) in CF_3CO_2H (5 mL) *at room temperature* to give **2h** as a red solid. Obtained: 543 mg (98% yield).

*Synthesis of Orthopalladated **2i***. Following the same synthetic procedure than described for **2a**, $Pd(OAc)_2$ (233 mg, 1.04 mmol) was reacted with **1i** (377.3 mg, 1.04 mmol) in CF_3CO_2H (5 mL) at 72.4 °C to give **2i** as a red solid. Obtained: 590 mg (98% yield). ^1H NMR (DMSO-d_6, 300.13 MHz): δ 7.85-7.77 (m, 4H, H_{ar}), 7.74 (s, 1H, $=CH_{vinyl}$), 7.71-7.68 (m, 2H, H_{ar}), 7.60 (d, 1H, J = 7.0Hz, H_{ar}), 7.53-7.51 (m, 3H, H_{ar}), 7.45-7.38 (m, 2H, H_{ar}), 7.32 (d, 1H, J = 16.2 Hz, $=CH_{olef}$), 3.91 (s, 2H, CH_2, $C_{13}H_8$). $^{13}C\{^1H\}$ NMR (DMSO-d_6, 75.47 MHz): δ 164.8 (C=N), 161.1 (C=O), 145.6 (=CH, C_{olef}), 144.8 (C_q), 143.3 (C_q), 140.1 (C_q), 139.9 (C_q), 136.8 (=CH, C_{vinyl}), 136.5 (C_q), 134.2 (C_q), 131.7 (CH_{ar}), 130.4 (C_q), 129.3 (CH_{ar}), 129.0 (CH_{ar}), 128.9 (CH_{ar}), 128.1 (CH_{ar}), 127.2 (CH_{ar}), 125.7 (CH_{ar}), 125.5 (CH_{ar}), 122.9 (C_q), 120.2 (CH_{ar}), 112.3 (=CH, C_{olef}), 35.4 (CH_2). ^{19}F NMR (DMSO-d_6, 282.40 MHz): δ −73.96. HRMS (ESI) *m/z*: $[M\text{-}CF_3COO]^+$ Calcd for $[C_{52}H_{32}F_3N_2O_6Pd_2]^+$ 1051.0286. Found 1051.0300.

*Synthesis of Orthopalladated **2j***. Following the same synthetic procedure than described for **2a**, $Pd(OAc)_2$ (233 mg, 1.04 mmol) was reacted with **1j** (348 mg, 1.04 mmol) in CF_3CO_2H (5 mL) at 72.4 °C to give **2j** as a red solid. Obtained: 512 mg (89% yield). ^1H NMR ($CDCl_3$, 300.13 MHz): δ 7.81 (s, 1H, $=CH_{vinyl}$), 7.51 (m, 2H, H_o, C_6H_5), 7.46-7.41 (m, 4H, H_m + H_p C_6H_5 + $=CH_{olef}$), 7.04 (d, 1H, J=16 Hz, $=CH_{olef}$), 6.67, 6.49 (AB spin system, 2H, J = 8 Hz, H_5/H_6 C_6H_2), 3.98 (s, 3H, OMe), 3.69 (s, 3H, OMe). $^{13}C\{^1H\}$ NMR ($CDCl_3$, 75.47 MHz): δ 165.4 (C=N), 160.2 (C=O), 150.3 (C-OMe, C_6H_2), 148.1 (=CH, C_{olef}), 147.8 (C-OMe, C_6H_2), 133.9 (C_i, C_6H_5), 132.2 (C_p, C_6H_5), 130.6 (=CH, C_{vinyl}), 129.3 (C_o, C_6H_5), 129.2 (C_m, C_6H_5), 128.0 (CH, C_6H_2), 123.8 (=C), 123.6 (C_q, C_6H_2), 123.1 (C_q, C_6H_2), 115.5 (CH, C_6H_2), 110.5 (=CH, C_{olef}), 62.0 (OMe), 56.1 (OMe). ^{19}F NMR ($CDCl_3$, 282.40 MHz): δ −74.63. HRMS (ESI) *m/z*: $[M\text{-}CF_3COO]^+$ Calcd for $[C_{42}H_{32}F_3N_2O_{10}Pd_2]^+$ 995.0083. Found 995.0087.

*Synthesis of Orthopalladated **2k***. Following the same synthetic procedure than described for **2a**, $Pd(OAc)_2$ (233 mg, 1.04 mmol) was reacted with **1k** (323 mg, 1.04 mmol) in CF_3CO_2H (5 mL) at 72.4 °C to give **2k** as a red solid. Obtained: 509 mg (93% yield). This compound was totally insoluble in the usual NMR solvents (even DMSO), precluding its characterization in solution. HRMS (ESI) *m/z*: $[M+Na]^+$ Calcd for $[C_{40}H_{20}F_{10}N_2NaO_8Pd_2]^+$ 1082.9031. Found 1082.9055.

*Synthesis of Orthopalladated **2m***. Following a similar synthetic procedure than described for **2a**, $Pd(OAc)_2$ (233 mg, 1.04 mmol) was reacted with **1m** (348 mg, 1.04 mmol) in CF_3CO_2H (5 mL) *at room temperature* to give **2m** as a red solid. Obtained: 553 mg (96% yield). ^1H NMR ($CDCl_3$, 300.13 MHz): δ 7.56 (m, 2H, H_o, C_6H_5), 7.45-7.41 (m, 3H, H_m, H_p, C_6H_5), 7.36 (d, 1H, J=16 Hz, =CH_{olef}), 7.25 (s, 1H, =CH_{vinyl}), 6.99 (d, 1H, J=16 Hz, =CH_{olef}), 6.68 (s, 1H, H_3 C_6H_2), 6.56 (s, 1H, H_6, C_6H_2), 3.98 (s, 3H, OMe), 3.51 (s, 3H, OMe). $^{13}C\{^1H\}$ NMR ($CDCl_3$, 75.47 MHz): δ 164.7 (C=N), 160.9 (C=O), 150.3 (C-OMe, C_6H_2), 147.6 (C-OMe, C_6H_2), 147.5 (=CH, C_{olef}), 136.6 (=CH, C_{vinyl}), 133.8 ($C_{i,}$ C_6H_5), 132.1 (C_p, C_6H_5), 129.2 (2C overlapped, C_o+C_m, C_6H_5), 128.6 (=C), 122.2 (C_q, C_6H_2), 120.7 (C_q, C_6H_2), 115.0 (CH, C_6, C_6H_2), 113.8 (CH, C_3, C_6H_2), 110.3 (=CH, C_{olef}), 56.2 (s, OMe), 55.7 (s, OMe). ^{19}F NMR ($CDCl_3$, 282.40 MHz): δ −74.38. HRMS (ESI) *m/z*: $[M-CF_3COO]^+$ Calcd for $[C_{42}H_{32}F_3N_2O_{10}Pd_2]^+$ 995.0083. Found 995.0098.

General Synthesis and Characterization of Mononuclear Orthopalladated Pyridine and Bis-Pyridine Complexes (3a-3d, 3f-3k, 3m, 4d, 4h, 5h, 6h). The synthesis of derivatives **3** containing one pyridine ligand has been carried out in all cases using the same procedure, detailed here for the synthesis of **3a**. For all prepared complexes, signals assigned to the quaternary carbons of the $\underline{C}F_3\underline{C}OO$ ligand were not found in the ^{13}C NMR spectra. This is due to a dynamic coordination-release of this ligand, and also to the fact that NMR spectra were recorded at room temperature. In the case of complex **3e**, as representative, the ^{13}C NMR spectrum was measure at low temperature (233 K) and all signals were observed.

*Synthesis of orthopalladated **3a**.* Pyridine (14.5 µL, 0.185 mmol) was added to a stirred suspension of **2a** (100.0 mg, 0.090 mmol) in CH_2Cl_2 (10 mL) at room temperature. The starting suspension gradually dissolved and a yellow solution was obtained after few minutes. The mixture was further stirred at room temperature for 30 min. At this point, any remaining insoluble residue was removed by filtration. The clear yellow solution was evaporated to dryness, and the obtained yellow solid of **3a** was dried under vacuum. Obtained: 96.7 mg (86% yield). ^1H NMR (CD_2Cl_2, 300.13 MHz): δ 8.72 (m, 2H, H_o, C_5H_5N), 8.49 (m, 2H, H_o, C_6H_5), 7.85 (tt, 1H, J = 7.8 Hz, J=1.4 Hz, H_p, C_5H_5N), 7.70 (t, 1H, J = 7.3Hz, H_p, C_6H_5), 7.68 (s, 1H, =CH_{vinyl}), 7.61 (m, 2H, H_m, C_6H_5), 7.53-7.51 (m, 2H, $C_{13}H_8$), 7.42 (m, 1H, $C_{13}H_8$), 7.37 (m, 2H, H_m, C_5H_5N), 7.34-7.28 (m, 2H, $C_{13}H_8$), 7.06 (s, 1H, $C_{13}H_8$), 3.87 (s, 2H, CH_2, $C_{13}H_8$). $^{13}C\{^1H\}$ NMR (CD_2Cl_2, 75.47 MHz): δ 167.5 (C=N), 162.0 (C=O), 153.5 (C_o, C_5H_5N), 143.4 (2C overlaped, 2C_q), 143.2 (C_q), 141.0 (C_q), 140.6 (C_q), 140.0 (=CH, C_{vinyl}), 139.2 (C_p, C_5H_5N), 134.9 (C_p, C_6H_5), 132.4 (C_q), 130.9 (C_o, C_6H_5), 129.1 (CH), 129.1 (CH), 128.9 (C_m, C_6H_5), 128.5 (CH), 127.3 (CH), 125.7 (CH), 125.6 (C_m, C_5H_5N), 124.2 (C_q), 123.4 (C_q), 120.7 (CH), 36.4 (CH_2). ^{19}F NMR (CD_2Cl_2, 282.40 MHz): δ –

75.99 (CF_3COO). HRMS (ESI) *m/z*: $[M - CF_3COO+H]^+$ Calcd for $[C_{28}H_{19}N_2O_2Pd]^+$ 524.0461. Found 524.0463. Elem. Anal. Calc for $[C_{30}H_{18}F_3N_2O_4Pd]$: C, 56.84; H, 2.86; N, 4.42. Found: C, 56.74; H, 3.16; N, 4.70.

*Synthesis of orthopalladated **3b***. Compound **3b** was obtained following the same experimental procedure than that described for **3a**: pyridine (15.3 µL, 0.194 mmol) was reacted with **2b** (100.0 mg, 0.097 mmol) in CH_2Cl_2 (10 mL) at room temperature to give **3b** as a yellow solid. Obtained: 113.0 mg (98% yield). ^1H NMR (CD_2Cl_2, 300.13 MHz): δ 8.60 (m, 2H, H_o, C_5H_5N), 8.56 (s, 1H, $=CH_{vinyl}$), 8.50 (m, 2H, H_o, C_6H_5), 8.35 (d, 1H, J = 9.0 Hz, $C_{10}H_6$), 7.84 (tt, 1H, J = 7.7 Hz, J = 1.6 Hz, H_p, C_5H_5N), 7.78 (d, 1H, J = 9.0 Hz, $C_{10}H_6$), 7.70 (t, 1H, J=7.4 Hz, H_p, C_6H_5), 7.65-7.59 (m, 3H, H_m, C_6H_5, H_{ar}, $C_{10}H_6$), 7.48 (d, 1H, J = 9 Hz, $C_{10}H_6$), 7.43 (d, 1H, J = 8 Hz, $C_{10}H_6$), 7.34 (m, 2H, H_m, C_5H_5N), 6.96 (d, 1H, J = 9 Hz, $C_{10}H_6$). $^{13}C\{^1H\}$ NMR (CD_2Cl_2, 75.47 MHz): δ 168.2 (C=N), 162.1 (C=O), 153.2 (C_o, C_5H_5N), 149.3 (C_q), 139.2 (C_p, C_5H_5N), 135.6 (CH, $C_{10}H_6$), 135.1 (C_p, C_6H_5), 133.9 (=CH, C_{vinyl}), 132.4 (C_q), 132.3 (C_q), 131.0 (C_o, C_6H_5), 131.0 (CH, $C_{10}H_6$), 129.2 (CH, $C_{10}H_6$), 129.0 (C_m, C_6H_5), 128.6 (C_q), 128.0 (CH, $C_{10}H_6$), 125.9 (CH, $C_{10}H_6$), 125.7 (C_m, C_5H_5N), 125.0 (C_q), 123.2 (C_q), 121.8 (CH, $C_{10}H_6$). ^{19}F NMR (CD_2Cl_2, 282.40 MHz): δ –76.02 (CF_3COO). HRMS (ESI) *m/z*: $[M - CF_3COO+H]^+$ Calcd for $[C_{25}H_{17}N_2O_2Pd]^+$ 483.0335. Found 483.0334. Elem. Anal. Calc for $[C_{27}H_{16}F_3N_2O_4Pd]$: C, 54.43; H, 2.71; N, 4.70. Found: C, 54.33; H, 3.09; N, 5.10.

*Synthesis of orthopalladated **3c***. Compound **3c** was obtained following the same experimental procedure than that described for **3a**: pyridine (13.4 µL, 0.173 mmol) was reacted with **2c** (100.0 mg, 0.085 mmol) in CH_2Cl_2 (10 mL) at room temperature to give **3c** as a red solid. Obtained: 105.0 mg (92% yield). ^1H NMR (CD_2Cl_2, 300.13 MHz): δ 8.76 (s, 1H, $=CH_{vinyl}$), 8.63 (d, 1H, J = 8 Hz, $C_{16}H_8$), 8.61 (m, 2H, H_o, C_5H_5N), 8.56 (m, 2H, H_o, C_6H_5), 8.26-8.23 (m, 2H, $C_{16}H_8$), 8.18 (d, 1H, J = 9 Hz, $C_{16}H_8$), 8.05-7.97 (m, 2H, $C_{16}H_8$), 7.82 (tt, 1H, J = 7.8 Hz, J = 1.5Hz, H_p, C_5H_5N), 7.73 (tt, 1H, J = 7.4 Hz, J = 1.3 Hz, H_p, C_6H_5), 7.66-7.61 (m, 3H, H_m, C_6H_5, $C_{16}H_8$), 7.45 (s, 1H, $C_{16}H_8$), 7.30 (m, 2H, H_m, C_5H_5N). $^{13}C\{^1H\}$ NMR (CD_2Cl_2, 75.47 MHz): δ 167.6 (C=N), 162.3 (C=O), 153.3 (C_o, C_5H_5N), 143.7 (C_q), 139.1 (C_p, C_5H_5N), 135.0 (C_p, C_6H_5), 134.7 (CH, $C_{16}H_8$), 134.0 (=CH, C_{vinyl}), 133.3 (C_q), 133.2 (C_q), 131.4 (C_q), 131.1 (C_q), 130.9 (C_o, C_6H_5), 130.7 (CH, $C_{16}H_8$), 130.5 (C_q), 129.9 (CH, $C_{16}H_8$), 129.0 (C_m, C_6H_5), 127.1 (2C overlapped, CH, $C_{16}H_8$), 127.1 (CH, $C_{16}H_8$), 127.0 (C_q), 126.6 (CH, $C_{16}H_8$), 125.6 (C_m, C_5H_5N), 124.8 (C_q), 123.5 (C_q), 123.3 (C_q), 122.1 (CH, $C_{16}H_8$). ^{19}F NMR (CD_2Cl_2, 282.40 MHz): δ –75.97 (CF_3COO). HRMS (ESI) *m/z*: $[M - CF_3COO+H]^+$ Calcd for $[C_{31}H_{19}N_2O_2Pd]^+$ 557.0494. Found 557.0505. Elem. Anal. Calc for $[C_{33}H_{18}F_3N_2O_4Pd]$: C, 59.16; H, 2.71; N, 4.18. Found: C, 59.30; H, 3.00; N, 4.45.

*Synthesis of orthopalladated **3d***. Compound **3d** was obtained following the same experimental procedure than that described for **3a**: pyridine (15.0 µL, 0.194 mmol) was reacted with **2d** (100.0 mg, 0.095 mmol) in CH_2Cl_2 (10 mL) at

room temperature to give **3d** as a red solid. Obtained: 72.0 mg (63% yield). ^{1}H NMR (CD_2Cl_2, 300.13 MHz): δ 8.69 (m, 2H, H_o, C_5H_5N), 8.41 (m, 2H, H_o, C_6H_5), 8.18 (s, 1H, =CH_{vinyl}), 7.85 (t, 1H, J = 7.7 Hz, H_p, C_5H_5N), 7.66 (t, 1H, J = 7.2 Hz, H_p, C_6H_5), 7.57 (m, 2H, H_m, C_6H_5), 7.36 (m, 2H, H_m, C_5H_5N), 6.17 (d, 1H, J=2.2 Hz, H_4, C_6H_2), 5.75 (d, 1H, J=2.2 Hz, H_6, C_6H_2), 3.88 (s, 3H, OMe), 3.55 (s, 3H, OMe). ^{13}C{^{1}H} NMR (CD_2Cl_2, 75.47 MHz): δ 165.9 (C=N), 162.8 ($C_{3/5}$-OMe, C_6H_2), 162.3 (C=O), 160.3 ($C_{3/5}$-OMe, C_6H_2), 153.4 (C_o, C_5H_5N), 148.8 (=C), 139.1 (C_p, C_5H_5N), 134.3 (C_p, C_6H_5), 134.1 (=CH, C_{vinyl}), 130.5 (C_o, C_6H_5), 128.8 (C_m, C_6H_5), 125.5 (C_m, C_5H_5N), 123.7 (C_i, C_6H_5), 121.4 (C_1-Pd, C_6H_2), 117.2 (C_2, C_6H_2), 115.6 (C_6, C_6H_2), 95.2 (C_4, C_6H_2), 56.2 (OMe), 55.7 (OMe). ^{19}F NMR (CD_2Cl_2, 282.40 MHz): δ –76.03 (CF_3COO). HRMS (ESI) *m/z*: [M–CF_3COO+H]$^+$ Calcd for $[C_{23}H_{19}N_2O_4Pd]^+$ 493.0380. Found 493.0385. Elem. Anal. Calc for $[C_{25}H_{18}F_3N_2O_6Pd]$: C, 49.56; H, 2.99; N, 4.62. Found: C, 49.83; H, 3.32; N, 5.00.

*Synthesis of orthopalladated **3e***. Compound **3e** was obtained following a similar experimental procedure than that described for **3a**: pyridine (14.5 µL, 0.183 mmol) was reacted with **2e** (100.0 mg, 0.092 mmol) in CH_2Cl_2 (10 mL) at room temperature to give **3e** as a reddish-orange solid. Obtained: 93.0 mg (77% yield). ^{1}H NMR (CD_2Cl_2, 400.13 MHz, 233 K): δ 8.56 (m, 2H, H_o, C_5H_5N), 8.32 (s, 1H, =CH_{vinyl}), 7.87 (m, 1H, H_p, C_5H_5N), 7.37 (m, 2H, H_m, C_5H_5N), 6.12 (d, 1H, J = 2.1 Hz, H_4, C_6H_2), 5.48 (d, 1H, J = 2.1 Hz, H_6, C_6H_2), 3.86 (s, 3H, OMe), 3.49 (s, 3H, OMe). ^{13}C{^{1}H} NMR (CD_2Cl_2, 100.61 MHz, 233 K): δ 163.6 ($C_{3/5}$-OMe, C_6H_2), 161.8 (C=N), 161.3 ($C_{3/5}$-OMe, C_6H_2), 161.2 (C=O), 160.6 (q, J = 36 Hz, O$\underline{C}$OCF_3), 154.4 (C_i, C_6F_5), 145.5 (d, J = 261 Hz, C_o, C_6F_5), 144.1 (d, J = 261 Hz, C_p, C_6F_5), 152.7 (C_o, C_5H_5N), 149.9 (=C), 139.3 (C_p, C_5H_5N), 137.8 (d, J = 260 Hz, C_m, C_6F_5) 137.3 (=CH, C_{vinyl}), 125.6 (C_m, C_5H_5N), 117.6 (C_2, C_6H_2), 115.9 (q, J = 290 Hz, $\underline{C}F_3$), 115.1 (C_1-Pd, C_6H_2), 116.0 (C_6, C_6H_2), 94.6 (C_4, C_6H_2), 56.1 (s, OMe), 55.7 (s, OMe). ^{19}F NMR (CD_2Cl_2, 376.49 MHz, 233 K): δ = –75.72 (s, 3F, CF_3COO), -134.32 (AA' part of an AA'XX'Z spin system, 2F, F_o, C_6F_5), -146.16 (t, 1F, J = 21.1 Hz, F_p, C_6F_5), -160.75 (XX' part of an AA'XX'Z spin system, 2F, F_m, C_6F_5). HRMS (ESI) *m/z*: [M–CF_3COO+H]$^+$ Calcd for $[C_{23}H_{14}F_5N_2O_4Pd]^+$ 582.9909; found: 582.9928. Elem. Anal. Calc for $[C_{25}H_{13}F_8N_2O_6Pd]$: C, 43.16; H, 1.88; N, 4.03. Found: C, 42.94; H, 2.16; N, 4.41.

*Synthesis of orthopalladated **3f**.* Compound **3f** was obtained following the same experimental procedure than that described for **3a**: pyridine (15.0 µL, 0.194 mmol) was reacted with **2f** (100.0 mg, 0.097 mmol) in CH_2Cl_2 (10 mL) at room temperature to give **3f** as an orange solid. Obtained: 94.0 mg (75% yield). ^{1}H NMR (CD_2Cl_2, 300.13 MHz): δ 8.66 (m, 2H, H_o, C_5H_5N), 8.56 (m, 2H, H_o, $C_6H_4NO_2$), 8.37 (m, 2H, H_m, $C_6H_4NO_2$), 8.28 (s, 1H, =CH_{vinyl}), 7.87 (t, 1H, J = 7.7 Hz, H_p, C_5H_5N), 7.38 (m, 2H, H_m, C_5H_5N), 6.18 (d, 1H, J = 2.2 Hz, H_4, C_6H_2), 5.75 (d, 1H, J = 2.2Hz, H_6, C_6H_2), 3.90 (s, 3H, OMe), 3.57 (s, 3H, OMe). ^{13}C{^{1}H} NMR (CD_2Cl_2, 75.47 MHz): δ 165.9 (2C overlapped, C=N, $C_{3/5}$-OMe, C_6H_2), 161.7 (C=O), 161.2 ($C_{3/5}$-

OMe, C_6H_2), 153.3 (C_o, C_5H_5N), 150.7 (C_p-NO_2, $C_6H_4NO_2$), 150.0 (=C), 139.3 (C_p, C_5H_5N), 136.3 (=CH, C_{vinyl}), 131.5 (C_o, $C_6H_4NO_2$), 129.4 (C_i, $C_6H_4NO_2$), 125.6 (C_m, C_5H_5N), 123.8 (C_m, $C_6H_4NO_2$), 120.6 (C_1-Pd, C_6H_2), 117.1 (C_2, C_6H_2), 116.1 (C_6, C_6H_2), 95.2 (C_4, C_6H_2), 56.3 (OMe), 55.8 (OMe). ^{19}F NMR (CD_2Cl_2, 282.40 MHz): δ –76.17 (CF_3COO). HRMS (ESI) *m/z*: [M–CF_3COO+H]$^+$ Calcd for $C_{23}H_{18}N_3O_6Pd^+$ 538.0234. Found 538.0232 Elem. Anal. Calc for [$C_{25}H_{17}F_3N_3O_8Pd$]: C, 46.14; H, 2.63; N, 6.46. Found: C, 46.46; H, 3.06; N, 6.76.

*Synthesis of orthopalladated **3g**.* Compound **3g** was obtained following the same experimental procedure than that described for **3a**: pyridine (15.5 µL, 0.200 mmol) was reacted with **2g** (100.0 mg, 0.098 mmol) in CH_2Cl_2 (10 mL) at room temperature to give **3g** as an orange solid. Obtained: 80.0 mg (70% yield). ^{1}H NMR (CD_2Cl_2, 300.13 MHz): δ 8.73 (m, 2H, H_o, C_5H_5N), 7.90 (t, 1H, J = 7.7 Hz, H_p, C_5H_5N), 7.77-7.69 (m, 3H, =CH_{olef}, H_o, C_6H_5), 7.50-7.39 (m, 7H, H_m, H_p, C_6H_5, =CH_{vinyl}, H_m, C_5H_5N, =CH_{olef}), 7.19 (d, 1H, J = 7.7 Hz, H_3, C_6H_3), 6.91 (d, 1H, J = 7.7 Hz, H_4, C_6H_3), 6.29 (s, 1H, H_6, C_6H_3), 2.05 (s, 3H, Me). ^{13}C{^{1}H} NMR (CD_2Cl_2, 75.47 MHz): δ 165.9 (C=N), 162.0 (C=O), 153.2 (C_o, C_5H_5N), 147.5 (=CH, C_{olef}), 143.1 (=C), 142.5 (C_5-Me, C_6H_3), 139.3 (C_p, C_5H_5N), 139.3 (C_6, C_6H_3), 138.3 (C_p, C_6H_5), 134.8 (C_i, C_6H_5), 133.2 (C_3, C_6H_3), 132.2 (=CH, C_{vinyl}), 131.1 (C_2, C_6H_3), 129.7 (C_o, C_6H_5), 129.6 (C_m, C_6H_5), 127.0 (C_4, C_6H_3), 125.9 (C_m, C_5H_5N), 123.1 (C_1-Pd, C_6H_3), 112.1 (=CH, C_{olef}), 22.0 (Me). ^{19}F NMR (CD_2Cl_2, 282.40 MHz): δ –75.62 (CF_3COO). HRMS (ESI) *m/z*: [M–CF_3COO+H]$^+$ Calcd for [$C_{24}H_{19}N_2O_2Pd$]$^+$ 473.0481. Found 473.0483. Elem. Anal. Calc for [$C_{26}H_{18}F_3N_2O_4Pd$]: C, 53.30; H, 3.10; N, 4.78. Found: C, 53.10; H, 3.30; N, 5.12.

*Synthesis of orthopalladated **3h***. Compound **3h** was obtained following the same experimental procedure than that described for **3a**: pyridine (14.5 µL, 0.183 mmol) was reacted with **2h** (100.0 mg, 0.091 mmol) in CH_2Cl_2 (10 mL) at room temperature to give **3h** as a reddish-orange solid. Obtained: 80.0 mg (70% yield). ^{1}H NMR (CD_2Cl_2, 300.13 MHz): δ 8.78 (m, 2H, H_o, C_5H_5N), 8.04 (s, 1H, =CH_{vinyl}), 7.90 (t, 1H, J = 7.8 Hz, H_p, C_5H_5N), 7.69-7.63 (m, 3H, H_o, C_6H_5, =CH_{olef}), 7.45-7.37 (m, 6H, H_m, C_5H_5N, H_m, H_p, C_6H_5, =CH_{olef}), 6.15 (d, 1H, J = 2.2 Hz, H_4, C_6H_2), 5.60 (d, 1H, J = 2.2 Hz, H_6, C_6H_2), 3.87 (s, 3H, OMe), 3.46 (s, 3H, OMe). ^{13}C{^{1}H} NMR (CD_2Cl_2, 75.47 MHz): δ 164.1 (C=N), 162.5 (C-OMe, C_6H_2), 162.1 (C=O), 160.3 (C-OMe, C_6H_2), 153.1 (C_o, C_5H_5N), 147.6 (=C), 145.8 (=CH, C_{olef}), 139.2 (C_p, C_5H_5N), 134.8 (C_i, C_6H_5), 132.4 (=CH, C_{vinyl}), 131.6 (C_p, C_6H_5), 129.4 (C_o, C_6H_5), 129.3 (C_m, C_6H_5), 125.7 (C_m, C_5H_5N), 120.5 (C_1-Pd, C_6H_2), 116.8 (C_2, C_6H_2), 115.9 (C_6, C_6H_2), 112.1 (=CH, C_{olef}), 95.23 (C_4, C_6H_2), 56.2 (OMe), 55.5 (OMe). ^{19}F NMR (CD_2Cl_2, 282.40 MHz): δ –75.52 (CF_3COO). HRMS (ESI) *m/z*: [M–CF_3COO+H]$^+$ Calcd for [$C_{25}H_{21}N_2O_4Pd$]$^+$ 519.0536. Found 519.0543. Elem. Anal. Calc for [$C_{27}H_{20}F_3N_2O_6Pd$]: C, 51.32; H, 3.19; N, 4.43. Found: C, 51.18; H, 3.47; N, 4.56.

*Synthesis of orthopalladated **3i***. Compound **3i** was obtained following the same experimental procedure than that described for **3a**: pyridine (14.0 µL, 0.172 mmol) was reacted with **2i** (100.0 mg, 0.086 mmol) in CH_2Cl_2 (10 mL) at room temperature to give **3i** as an orange solid. Obtained: 87.0 mg (77% yield). ^{1}H NMR (CD_2Cl_2, 300.13 MHz): δ 8.83 (m, 2H, H_o, C_5H_5N), 7.92 (t, 1H, J = 7.7 Hz, H_p, C_5H_5N), 7.73-7.66 (m, 3H, H_o, C_6H_5, =CH_{olef}), 7.52-7.41 (m, 9H, $C_{13}H_8$ + C_6H_5), 7.31-7.26 (m, 3H, $C_{13}H_8$ + C_6H_5), 6.87 (s, 1H, H_4, $C_{13}H_8$), 3.85 (s, 2H, CH_2, $C_{13}H_8$). $^{13}C\{^1H\}$ NMR (CD_2Cl_2, 75.47 MHz): δ 165.7 (C=N), 162.0 (C=O), 153.4 (C_o, C_5H_5N), 147.4 (=CH, C_{olef}), 145.5 (C_q), 145.1 (C_q), 142.2 (C_q), 141.1 (C_q), 140.7 (C_q), 139.4 (C_p, C_5H_5N), 138.5 (CH, $C_{13}H_8$), 134.8 (C_q), 132.2 (=CH_{vinyl}), 129.7 (C_o, C_6H_5), 129.6 (C_m, C_6H_5), 129.6 (C_p, C_6H_5), 129.5 (CH, C_4, $C_{13}H_8$), 128.6 (CH, $C_{13}H_8$), 127.4 (CH, $C_{13}H_8$), 126.0 (CH, $C_{13}H_8$), 125.8 (C_m, C_5H_5N), 123.3 (C_q), 120.8 (CH, $C_{13}H_8$), 115.0 (C_q), 112.1 (=CH_{olef}), 36.4 (CH_2). ^{19}F NMR (CD_2Cl_2, 282.40 MHz): δ –75.54 (CF_3COO). HRMS (ESI) *m/z*: $[M–CF_3COO+H]^+$ Calcd for $[C_{30}H_{21}N_2O_2Pd]^+$ 547.0639; found: 547.0708. Elem. Anal. Calc for $[C_{32}H_{20}F_3N_2O_4Pd]$: C, 58.24; H, 3.05; N, 4.24. Found: C, 58.16; H, 3.32; N, 4.24.

*Synthesis of orthopalladated **3j***. Compound **3j** was obtained following the same experimental procedure than that described for **3a**: pyridine (14.5 µL, 0.183 mmol) was reacted with **2j** (100.0 mg, 0.091 mmol) in CH_2Cl_2 (10 mL) at room temperature to give **3j** as a reddish-orange solid Obtained: 53.0 mg (46% yield). ^{1}H NMR (CD_2Cl_2, 300.13 MHz): δ 8.72 (d, 2H, J = 7.0 Hz, H_o, C_5H_5N), 8.03 (s, 1H, =CH_{vinyl}), 7.89 (t, 1H, J=7 Hz, H_p, C_5H_5N), 7.79-7.71 (m, 3H, H_o, C_6H_5, =CH_{olef}), 7.51-7.38 (m, 6H, H_m, C_5H_5N; H_m, H_p, C_6H_5; =CH_{olef}), 6.58 (d, 1H, J = 8.6 Hz, H_5, C_6H_2), 6.09 (d, 1H, J = 8.6 Hz, H_6, C_6H_2), 3.92 (s, 3H, OMe), 3.76 (s, 3H, OMe). $^{13}C\{^1H\}$ NMR (CD_2Cl_2, 75.47 MHz): δ 166.2 (C=N), 162.0 (C=O), 153.2 (C_o, C_5H_5N), 150.6 ($C_{3/4}$-OMe, C_6H_2), 149.0 (=C), 148.9 ($C_{3/4}$-OMe, C_6H_2), 147.9 (=CH, C_{olef}), 139.2 (C_p, C_5H_5N), 134.8 (C_i, C_6H_5), 132.9 (C_6, C_6H_2), 132.4 (=CH, C_{vinyl}), 132.3 (C_p, C_6H_5), 129.8 (C_o, C_6H_5), 129.6 (C_m, C_6H_5), 127.2 (C_2, C_6H_2), 125.9 (C_m, C_5H_5N), 124.8 (C_1-Pd, C_6H_2), 116.3 (C_5, C_6H_2), 112.2 (=CH, C_{olef}), 62.4 (s, OMe), 56.4 (s, OMe). ^{19}F NMR (CD_2Cl_2, 282.40 MHz): δ –75.57 (CF_3COO). HRMS (ESI) *m/z*: $[M–CF_3COO+H]^+$ Calcd for $[C_{25}H_{21}N_2O_4Pd]^+$ 519.0536. Found 519.0535. Elem. Anal. Calc for $[C_{27}H_{20}F_3N_2O_6Pd]$: C, 51.32; H, 3.19; N, 4.43. Found: C, 50.99; H, 3.44; N, 4.42.

*Synthesis of orthopalladated **3k***. Compound **3k** was obtained following the same experimental procedure than that described for **3a**: pyridine (15.3 µL, 0.193 mmol) was reacted with **2k** (100.0 mg, 0.095 mmol) in CH_2Cl_2 (10 mL) at room temperature to give **3k** as a yellow solid. Obtained: 93.0 mg (81% yield). ^{1}H NMR ($CDCl_3$, 300.13 MHz): δ 8.61 (m, 2H, H_o, C_5H_5N), 7.83 (tt, 1H, J = 7.7 Hz, J = 1.5 Hz, H_p, C_5H_5N), 7.80 (d, 1H, J = 16.0 Hz, =CH_{olef}), 7.72 (m, 2H, H_o, C_6H_5,), 7.50-7.46 (m, 3H, H_m, H_p, C_6H_5), 7.39 (d, 1H, J = 16.0 Hz, =CH_{olef}), 7.38 (s, 1H, =CH_{vinyl}), 7.33 (m, 2H, H_m, C_5H_5N), 6.95 (dd, 1H, J = 8.4 Hz, J = 2.5 Hz, H_3, $C_6H_2F_2$), 6.46 (td, 1H, J = 8.6 Hz, J = 2.5 Hz, H_5, $C_6H_2F_2$). $^{13}C\{^1H\}$ NMR ($CDCl_3$, 75.47 MHz): δ

166.9 (C=N), 164.9 (d, J_{CF} = 248 Hz, C_6-F, $C_6H_2F_2$), 161.5 (d, J_{CF} = 249 Hz, C_4-F, $C_6H_2F_2$), 160.7 (C=O), 152.6 (C_o, C_5H_5N), 149.4 (=CH, C_{olef}), 138.6 (C_p, C_5H_5N), 136.0 (=C), 135.6 (=CH, C_{vinyl}), 133.8 (C_i, C_6H_5), 132.3 (C_p, C_6H_5), 129.6 (C_o, C_6H_5), 129.2 (C_m, C_6H_5), 125.5 (C_1-Pd, $C_6H_2F_2$), 124.9 (C_m, C_5H_5N), 118.3 (C_2, $C_6H_2F_2$), 114.1 (dd, J_{CF} = 21 Hz, J_{CF} = 3 Hz, C_3, $C_6H_2F_2$), 110.85 (=CH, C_{olef}), 106.7 (dd, J_{CF} = 34 Hz, J_{CF} = 24 Hz, C_5, $C_6H_2F_2$). ^{19}F NMR ($CDCl_3$, 282.40 MHz): δ –75.33 (s, 3F, CF_3COO), -87.50 (t, 1F, J = 8 Hz, C_6-F), -116.34 (q, 1F, J = 8 Hz, C_4-F). HRMS (ESI) *m/z*: $[M–CF_3COO+H]^+$ Calcd for $[C_{23}H_{15}F_2N_2O_2Pd]^+$ 495.0136. Found 495.0132. Elem. Anal. Calc for $[C_{25}H_{14}F_5N_2O_4Pd]$: C, 49.40; H, 2.32; N, 4.61. Found: C, 49.02; H, 2.63; N, 4.84.

*Synthesis of orthopalladated **3m**.* Compound **3m** was obtained following the same experimental procedure than that described for **3a**: pyridine (14.5 µL, 0.183 mmol) was reacted with **2m** (100.0 mg, 0.091 mmol) in CH_2Cl_2 (10 mL) at room temperature to give **3m** as a reddish-orange solid. Obtained: 91.0 mg (79% yield). ^{1}H NMR ($CDCl_3$, 300.13 MHz): δ 8.84 (m, 2H, H_o, C_5H_5N), 7.90 (t, 1H, J = 7.7 Hz, H_p, C_5H_5N), 7.74-7.67 (m, 3H, H_o, C_6H_5; =CH_{olef}), 7.47-7.39 (m, 7H, H_m, C_5H_5N; H_m, H_p, C_6H_5; =CH_{olef}; =CH_{vinyl}), 6.80 (s, 1H, H_3, C_6H_2), 5.88 (s, 1H, H_6, C_6H_2), 3.86 (s, 3H, OMe), 3.40 (s, 3H, OMe). $^{13}C\{^1H\}$ NMR ($CDCl_3$, 75.47 MHz): δ 165.1 (C=N), 161.6 (C=O), 153.1 (C_o, C_5H_5N), 150.4 (C-OMe, C_6H_2), 147.6 (C-OMe, C_6H_2), 147.3 (=CH, C_{olef}), 138.8 (C_p, C_5H_5N), 137.6 (=CH, C_{vinyl}), 135.9 (=C), 134.3 (C_i, C_6H_5), 131.8 (C_p, C_6H_5), 129.4 (C_o, C_6H_5), 129.2 (C_m, C_6H_5), 126.0 (C_2, C_6H_2), 125.5 (C_m, C_5H_5N), 121.8 (C_1-Pd, C_6H_2), 119.7 (C_6, C_6H_2), 114.5 (C_3, C_6H_2), 111.5 (=CH, C_{olef}), 56.0 (OMe), 55.6 (OMe). ^{19}F NMR ($CDCl_3$, 282.40 MHz): δ –75.35 (CF_3COO). HRMS (ESI) *m/z*: $[M–CF_3COO+H]^+$ Calcd for $[C_{25}H_{21}N_2O_4Pd]^+$ 519.0536. Found 519.0528. Elem. Anal. Calc for $[C_{27}H_{20}F_3N_2O_6Pd]$: C, 51.32; H, 3.19; N, 4.43. Found: C, 51.37; H, 3.43; N, 4.67.

*Synthesis of orthopalladated bis-pyridine complex **4d**.* The dinuclear chloride bridge precursor[Pd(μ-Cl)(C^N-**1d**)]$_2$, containing the orthopalladated oxazolone **1d**, was prepared as described previously.[19b] A suspension of [Pd(μ-Cl)(C^N-**1d**)]$_2$ (115.0 mg, 0.128 mmol) in 10mL of CH_2Cl_2/acetone (8/2) was treated with $AgClO_4$ (53 mg, 0.256 mmol), and the resulting mixture was stirred for 30 minutes at room temperature under exclusion of light. After the reaction time, the precipitated AgCl was removed by filtration. The resulting clear orange solution was treated with pyridine (40.6 µL, 0.512 mmol), and further stirred for 30 minutes. The clear yellow solution thus obtained was evaporated to dryness, and the obtained orange solid of **4d** was dried under vacuum. Obtained: 143.0 mg (83% yield). ^{1}H NMR ($CDCl_3$, 300.13 MHz): δ 9.03 (m, 2H, H_o, C_5H_5N), 8.47 (m, 2H, H_o, C_5H_5N), 8.30 (s, 1H, =CH_{vinyl}), 8.10 (m, 2H, H_o, C_6H_5), 7.80 (t, 1H, J = 7.7 Hz, H_p, C_5H_5N), 7.56 (m, 2H, H_m, C_6H_5), 7.53-7.47 (m, 3H, H_m, C_5H_5N; H_p, C_6H_5), 7.40 (t, 1H, J = 7.7 Hz, H_p, C_5H_5N), 7.03 (m, 2H, H_m, C_5H_5N), 6.14 (d, 1H, J = 2.1 Hz, H_4, C_6H_2), 5.97 (d, 1H, J = 2.1 Hz, H_6, C_6H_2), 3.88 (s, 3H, OMe), 3.55 (s, 3H,

OMe). $^{13}C\{^1H\}$ NMR ($CDCl_3$, 75.47 MHz): δ 165.8 (C=N), 163.5 ($C_{3/5}$-OMe, C_6H_2), 161.7 (C=O), 160.8 ($C_{3/5}$-OMe, C_6H_2), 152.5 (C_o, C_5H_5N), 150.2 (C_o, C_5H_5N), 149.5 (=C), 139.3 (C_p, C_5H_5N), 138.4 (C_p, C_5H_5N), 135.9 (=CH, C_{vinyl}), 134.6 (C_p, C_6H_5), 130.1 (C_o, C_6H_5), 129.7 (C_m, C_6H_5), 126.9 (C_m, C_5H_5N), 125.5 (C_m, C_5H_5N), 122.9 ($C_{i,}$ C_6H_5), 119.5 (C_1-Pd, C_6H_2), 116.7 (C_2, C_6H_2), 114.5 (C_6, C_6H_2), 96.1 (C_4, C_6H_2), 56.0 (OMe), 55.8 (OMe). HRMS (ESI) *m/z*: $[M–ClO_4–py+H]^+$ Calcd for $[C_{23}H_{19}N_2O_4Pd]^+$ 493.0380. Found 493.0371. Elem. Anal. Calc for $[C_{28}H_{22}ClN_3O_8Pd]$: C, 50.17; H, 3.31; N, 6.27. Found: C, 50.13; H, 3.64; N, 5.96.

*Synthesis of orthopalladated bis-pyridine complex **4h**.* Complex **4h** was prepared following the same procedure than that reported for **4d**, but starting from $[Pd(\mu\text{-}Cl)(C^\wedge N\text{-}\mathbf{1h})]_2$.[19a] Therefore, $[Pd(\mu\text{-}Cl)(C^\wedge N\text{-}\mathbf{1h})]_2$ (155.0 mg, 0.163 mmol) was reacted with $AgClO_4$ (68.0 mg, 0.326 mmol) and pyridine (51.6 µL, 0.652 mmol) in CH_2Cl_2/acetone (8/2, 10 mL) under exclusion of light to give **4h** as a red solid. Obtained: 185.0 mg (81% yield). ^1H NMR ($CDCl_3$, 400.13 MHz, 233 K) δ: 9.03 (d, J = 5.4Hz, 2H, H_o, C_5H_5N), 8.97 (d, J = 5.4Hz, 2H, H_o, C_5H_5N), 8.18 (s, 1H, =CH_{vinyl}), 7.87 (t, 1H, J = 8.1 Hz, H_p, C_5H_5N), 7.62-7.55 (m, 3H, H_m + H_p, C_5H_5N), 7.48 (m, 2H, H_m, C_5H_5N), 7.40-7.26 (m, 6H, H_o+H_m+H_p $C_6H_{5,}$ =CH_{olef}), 6.25 (d, J = 16.0Hz, 1H, =CH_{olef}) 6.10 (s, 1H, H_4, C_6H_2), 5.64 (s, 1H, H_6, C_6H_2), 3.86 (s, 3H, OMe), 3.49 (s, 3H, OMe). $^{13}C\{^1H\}$ NMR ($CDCl_3$, 100.6 MHz, 233 K) δ: 162.8 (2C, C-OMe, C_6H_2 + C=N), 161.7 (C=O), 160.4 (C-OMe, C_6H_2), 151.9 (C_o, C_5H_5N), 150.2 (C_o, C_5H_5N), 148.7 (=C), 146.3 (=CH, C_{olef}), 139.4 (2C, 2C_p, C_5H_5N), 134.2 (=CH, C_{vinyl}), 132.7 ($C_{i,}$ C_6H_5), 132.1 (C_p, C_6H_5), 129.3 (C_o/C_m C_6H_5), 129.0 (C_o/C_m, C_6H_5), 127.0 (C_m, C_5H_5N), 126.7 (C_m, C_5H_5N), 118.9 (C_2, C_6H_2), 116.1 (C_1-Pd, C_6H_2), 114.3 (C_6, C_6H_2), 109.1 (=CH, C_{olef}), 96.7 (C_4, C_6H_2), 55.9 (OMe), 55.6 (OMe). HRMS (ESI) *m/z*: $[M–ClO_4–py+H]^+$ Calcd for $[C_{25}H_{21}N_2O_4Pd]^+$ 519.0541. Found 519.0554. Elem. Anal. Calc for $[C_{30}H_{24}ClN_3O_8Pd]$: C, 51.74; H, 3.47; N, 6.03. Found: C, 51.89; H, 3.74; N, 5.94.

*Synthesis of orthopalladated chloride complex **5h**.* The dinuclear precursor$[Pd(\mu\text{-}Cl)(C^\wedge N\text{-}\mathbf{1h})]_2$, containing the orthopalladated oxazolone **1h**, was prepared as described previously.[19a] A suspension of $[Pd(\mu\text{-}Cl)(C^\wedge N\text{-}\mathbf{1h})]_2$ (136.0 mg, 0.143 mmol) in CH_2Cl_2 (10 mL) at room temperature was treated with pyridine (22.6 µL, 0.286 mmol). The initial suspension gradually dissolved, and a clear solution was obtained after few minutes. This solution was stirred for 30 minutes, and any remaining solid was removed by filtration after the reaction time. The resulting solution was evaporated to dryness, and the residue treated with Et_2O (20 mL) and stirring to give **5h** as an orange solid. Obtained: 120.0 mg (76% yield). ^1H NMR (CD_2Cl_2, 300.13 MHz): δ 8.74 (m, 2H, H_o, C_5H_5N), 8.06 (s, 1H, =CH_{vinyl}), 8.06 (d, 1H, J = 16.2 Hz, =CH_{olef}), 7.86 (t, 1H, J = 7.7 Hz, H_p, C_5H_5N), 7.70 (m, 2H, H_o, $C_6H_{5,}$), 7.61 (d, 1H, J = 16.2 Hz, =CH_{olef}), 7.45 (m, 3H, H_m, H_p, C_6H_5), 7.39 (m, 2H, H_m, C_5H_5N), 6.14 (d, J = 2.1 Hz, 1H, H_4, C_6H_2), 5.56 (d, J = 2.1 Hz, 1H, H_6, C_6H_2), 3.87 (s, 3H,

OMe), 3.47 (s, 3H, OMe). $^{13}C\{^1H\}$ NMR (CD_2Cl_2, 75.47 MHz): δ 165.1 (C=N), 163.1 ($C_{3/5}$-OMe, C_6H_2), 161.9 (C=O), 160.3 ($C_{3/5}$-OMe, C_6H_2), 153.8 (C_o, C_5H_5N), 143.8 (=C), 145.4 (=CH, C_{olef}), 138.8 (C_p, C_5H_5N), 135.7 (C_i, C_6H_5), 133.0 (=CH, C_{vinyl}), 131.4 (C_p, C_6H_5), 129.9 (C_1-Pd, C_6H_2), 129.6 (C_o, C_6H_5), 129.3 (C_m, C_6H_5), 125.7 (C_m, C_5H_5N), 117.0 (C_2, C_6H_2), 115.5 (C_6, C_6H_2), 115.4 (=CH, C_{olef}), 95.3 (C_4, C_6H_2), 56.3 (OMe), 55.7 (OMe). HRMS (ESI) *m/z*: $[M{-}py{+}Na]^+$ Calcd for $[C_{20}H_{16}ClNNaO_4Pd]^+$ 497.9700. Found 497.9696. Elem. Anal. Calc for $[C_{25}H_{20}ClN_2O_4Pd]$: C, 54.17; H, 3.64; N, 5.05. Found: C, 54.18; H, 3.92; N, 5.02.

*Synthesis of orthopalladated-NHC complex **6h**.* To a solution of **2h** (100.0 mg, 0.09 mmol) in dry THF (5 mL) under Ar atmosphere, 1,3-bis(2,4,6-trimethylphenyl)imidazolium chloride (61.9 mg, 0.18 mmol) and K_2CO_3 (25.0 mg, 0.18 mmol) were added. The resulting mixture was heated in an oil bath to the reflux temperature (66 °C) for 24 h. After the reaction time, the cooled solution was evaporated to dryness and the residue was purified by flash column chromatography, using silica gel as support and Et_2O as eluent. The orange-yellowish band developed was collected, the solvent evaporated to dryness and the orange solid residue characterized as complex **6h**. Obtained: 50 mg (32% yield). ^1H NMR (CD_2Cl_2, 300.13 MHz): δ 7.63 (s, 1H, =CH_{vinyl}), 7.44-7.35 (m, 6H, =CH_{olef}, H_o+H_m+H_p, C_6H_5), 7.11 (s, 2H, =CH, NHC), 7.01 (s, 2H, $H_{ar,}$ NHC-C_6H_2), 6.86 (s, 2H, $H_{ar,}$ NHC-C_6H_2), 6.35 (d, 1H, J = 16.2 Hz, =CH_{olef}), 6.18 (d, 1H, J = 2.2 Hz, H_4, C_6H_2), 5.93 (d, 1H, J = 2.2 Hz, H_6, C_6H_2), 3.75 (s, 3H, OMe), 3.71 (s, 3H, OMe), 2.38 (s, 6H, Me), 2.31 (s, 6H, Me), 2.17 (s, 6H, Me). $^{13}C\{^1H\}$ NMR (CD_2Cl_2, 75.47 MHz): δ 170.9 (Pd=C, NHC), 163.4 (C=N), 163.1 (C=O), 162.2 ($C_{3/5}$-OMe, C_6H_2), 161.0 ($C_{3/5}$-OMe, C_6H_2), 151.4 (=C), 143.8 (=CH, C_{olef}), 138.9 (C_q, NHC-C_6H_2), 137.1 (C_q, NHC-C_6H_2), 135.7 (C_q, NHC-C_6H_2), 135.1 ($C_{i,}$ C_6H_5), 133.9 (C_q, NHC-C_6H_2), 133.7 (=CH, C_{vinyl}), 131.2 (CH, C_6H_5), 129.9 (CH, NHC-C_6H_2), 129.4 (CH, C_6H_5), 129.0 (CH, NHC-C_6H_2), 128.9 (CH, C_6H_5), 123.8 (CH, NHC), 121.9 (C_1-Pd, C_6H_2), 119.1 (C_6, C_6H_2), 118.7 (C_2, C_6H_2), 112.3 (=CH, C_{olef}), 93.7 (C_4, C_6H_2), 55.9 (OMe), 55.6 (OMe), 21.3 (Me), 20.0 (Me), 19.3 (Me). ^{19}F NMR (CD_2Cl_2, 282.40 MHz): δ –74.27 (CF_3COO). HRMS (ESI) *m/z*: $[M{-}CF_3COO]^+$ Calcd for $[C_{41}H_{40}N_3O_4Pd]^+$ 744.2054. Found 744.2041. Elem. Anal. Calc for $[C_{43}H_{40}F_3N_3O_6Pd]$: C, 60.18; H, 4.70; N, 4.90. Found: C, 60.18; H, 5.04; N, 4.74.

2.- NMR spectra of starting (*Z*)-4-arylidene-5(4*H*)-oxazolones (1)

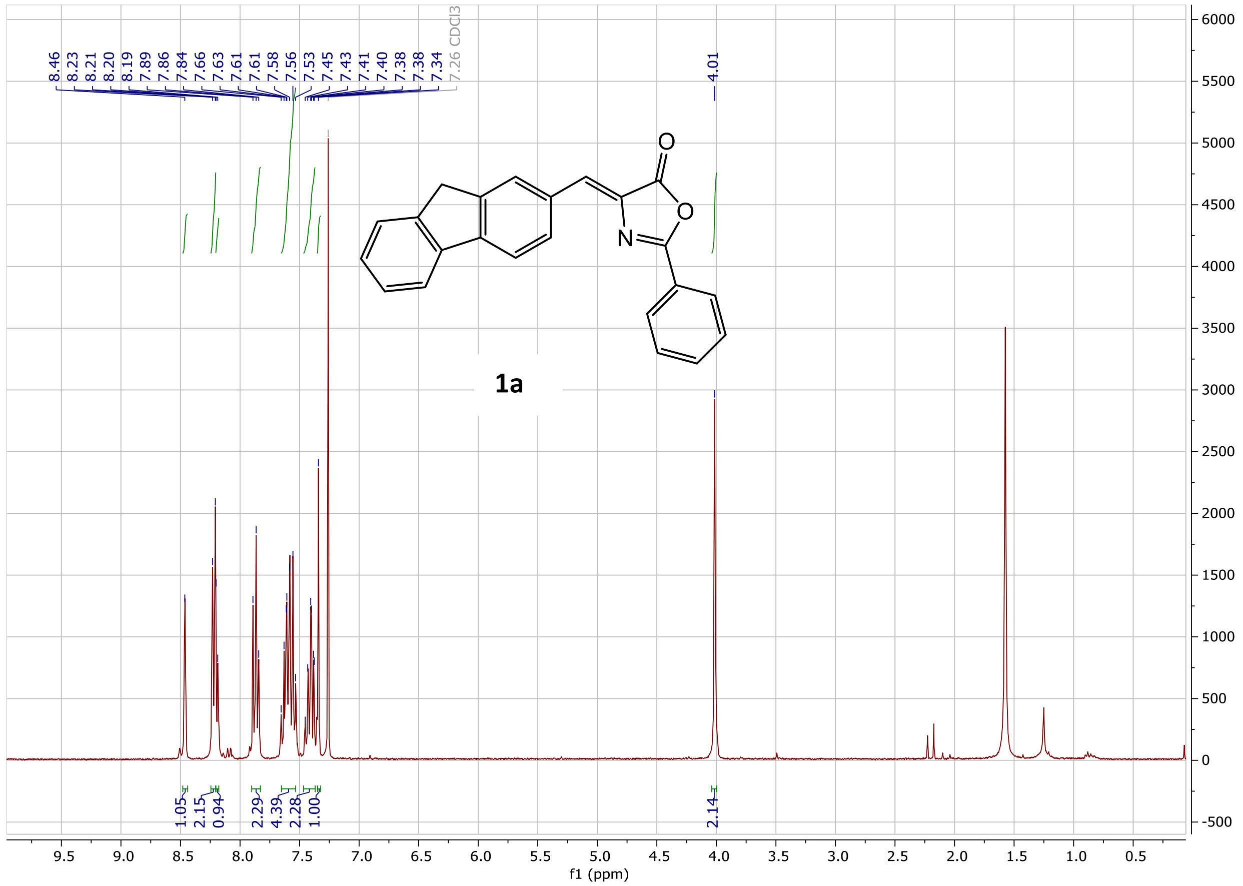

^{1}H-NMR spectrum (CDCl$_3$, 300.13 MHz) of **1a**[18a]

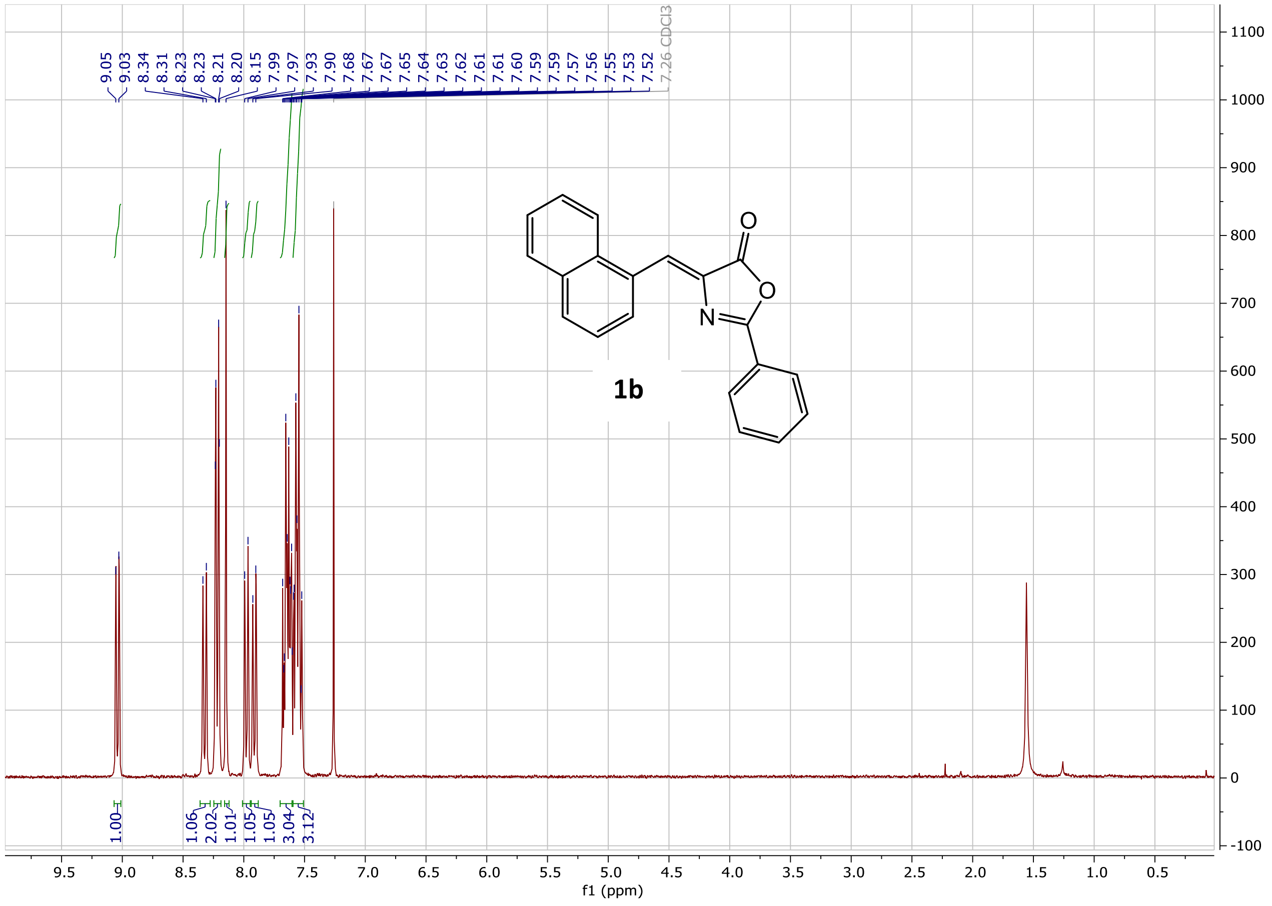

^{1}H-NMR spectrum (CDCl$_3$, 300.13 MHz) of **1b**[18b]

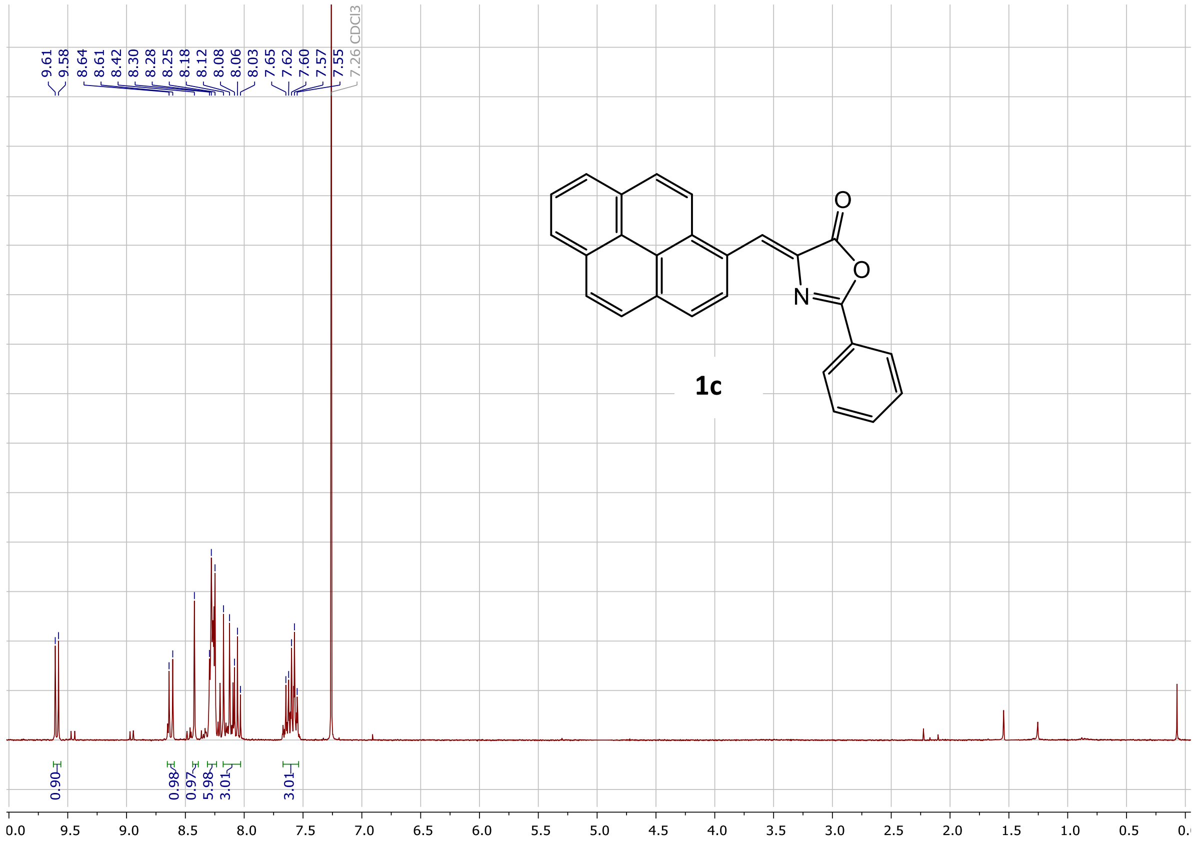

^{1}H-NMR spectrum ($CDCl_3$, 300.13 MHz) of **1c**[18c]

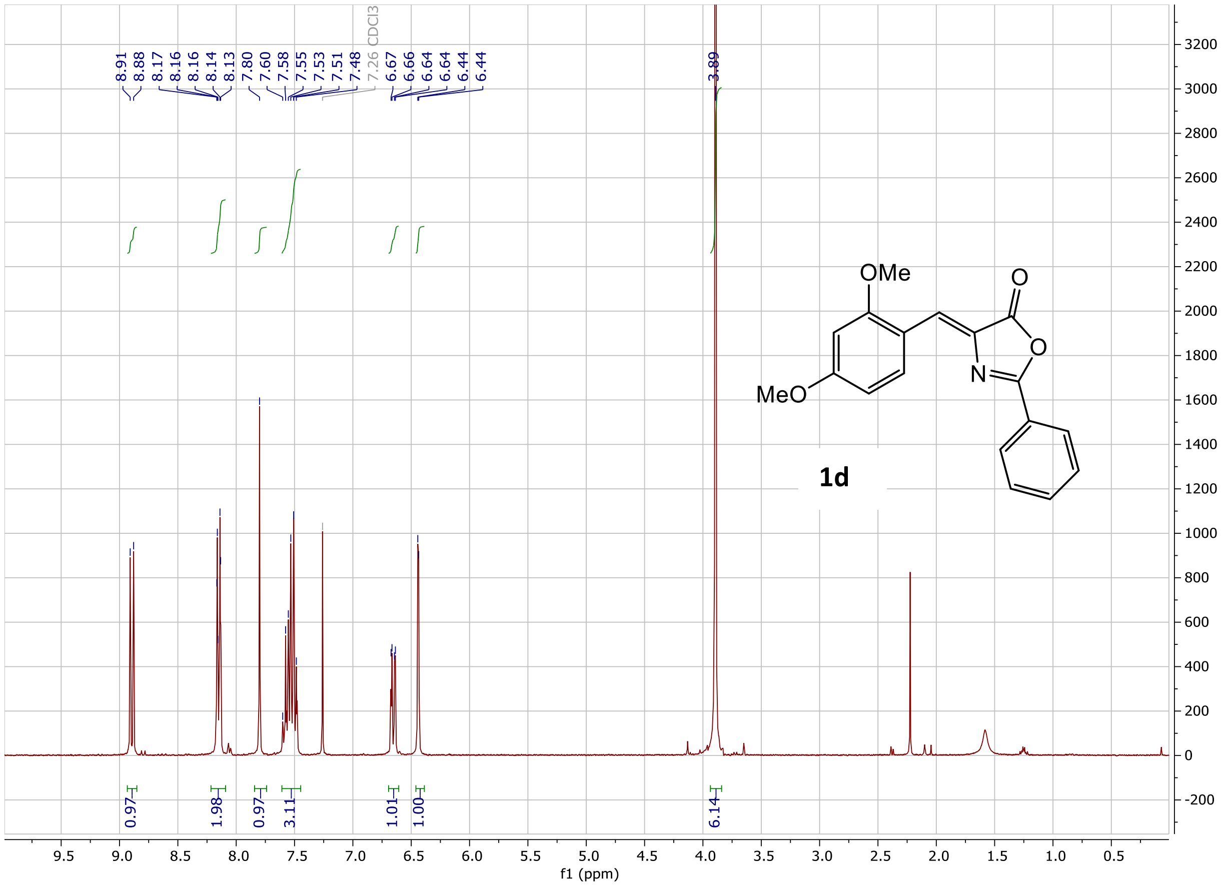

^{1}H-NMR spectrum ($CDCl_3$, 300.13 MHz) of **1d**[18d]

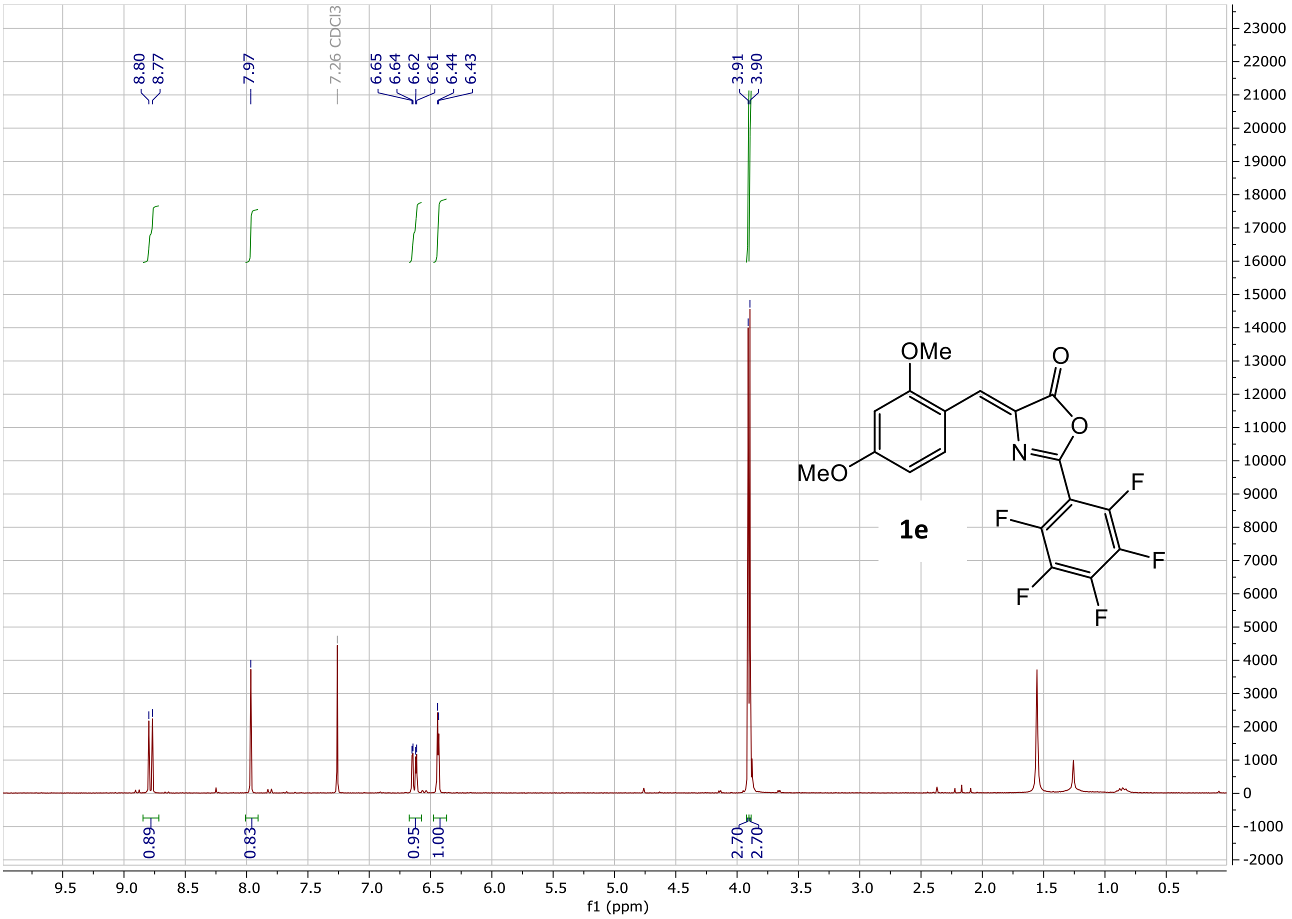

^{1}H-NMR spectrum (CDCl₃, 300.13 MHz) of **1e**

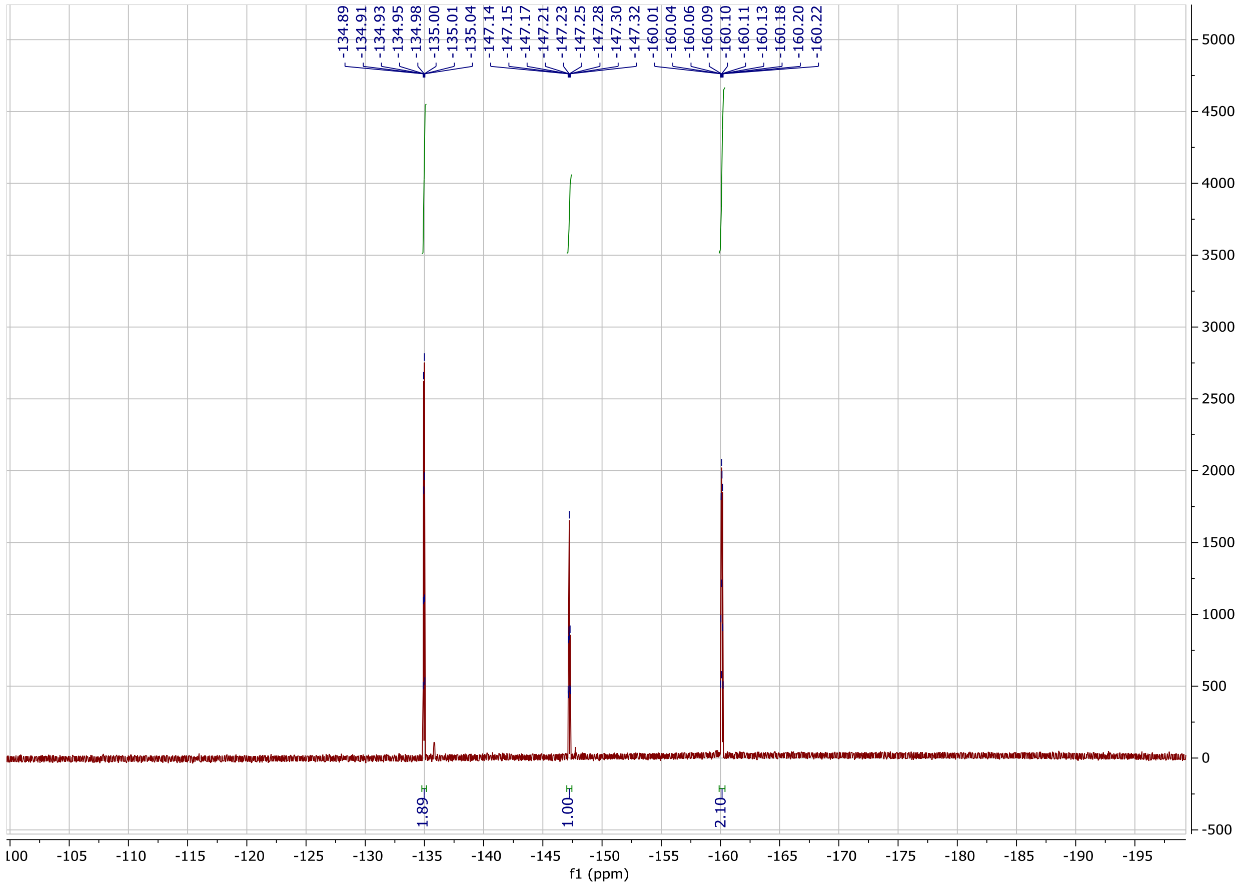

^{19}F-NMR spectrum (CDCl₃, 282.40 MHz) of **1e**

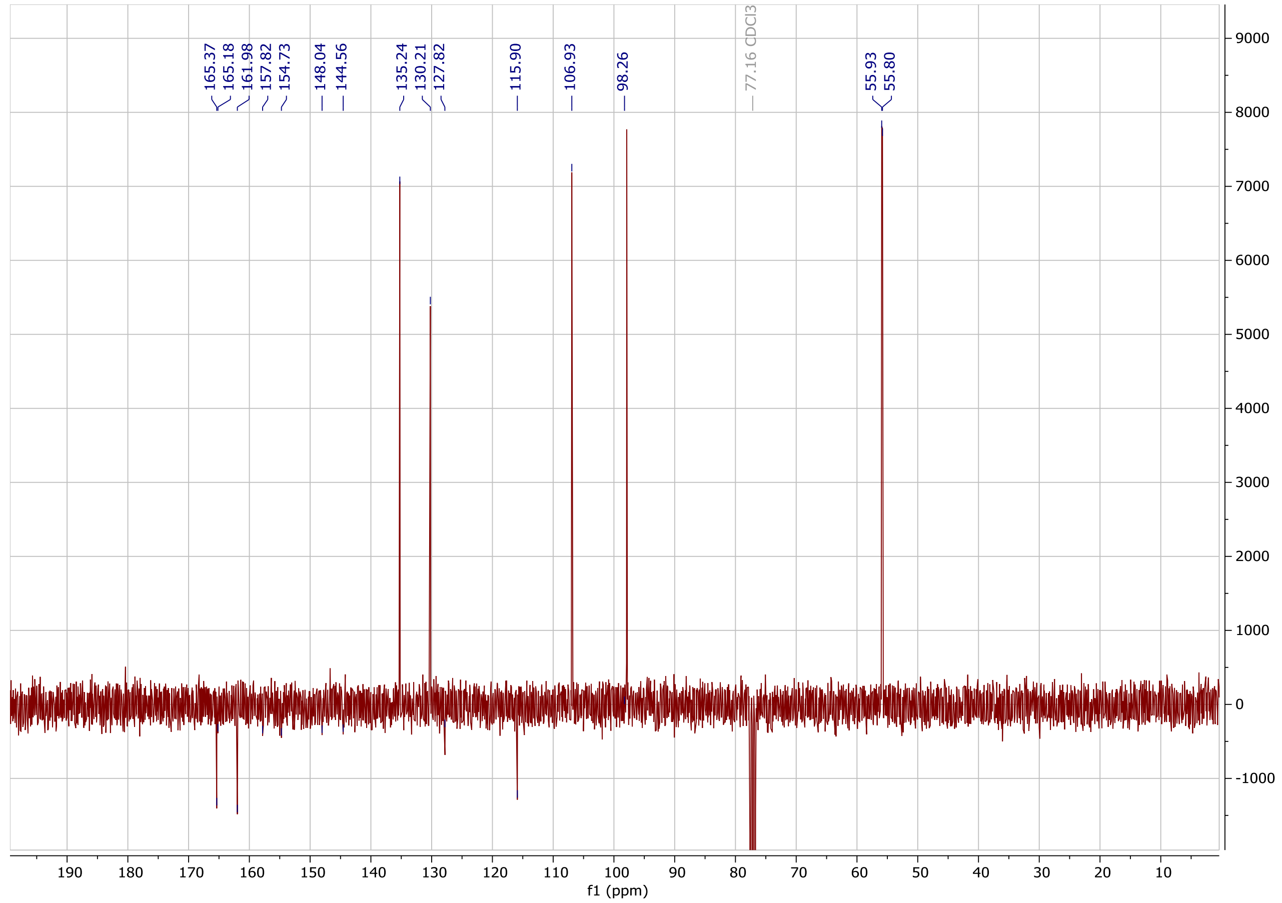

$^{13}C\{^{1}H\}$ (APT) NMR spectrum ($CDCl_3$, 75.47 MHz) of **1e**

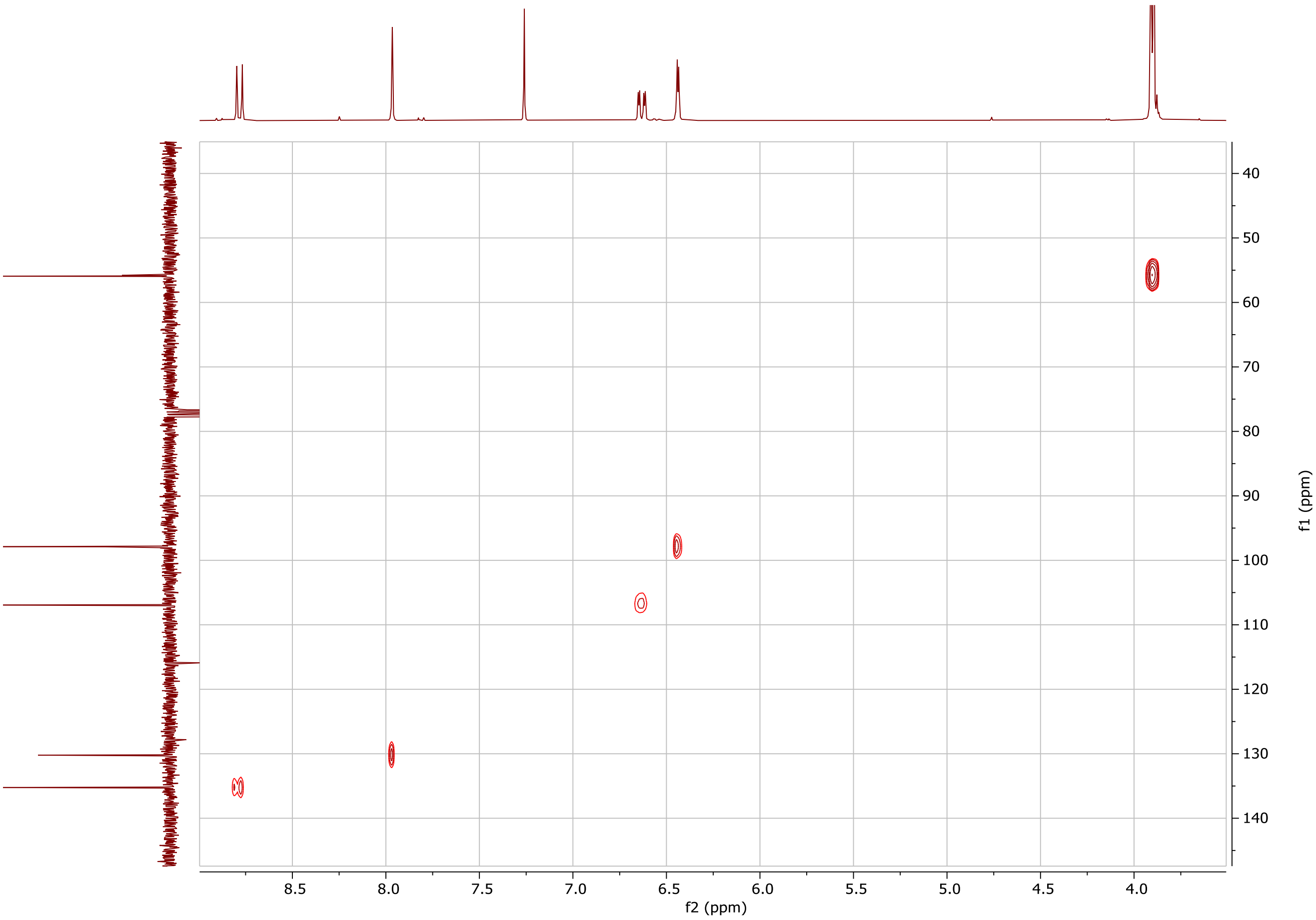

^{1}H - ^{13}C HSQC correlation spectrum of **1e**

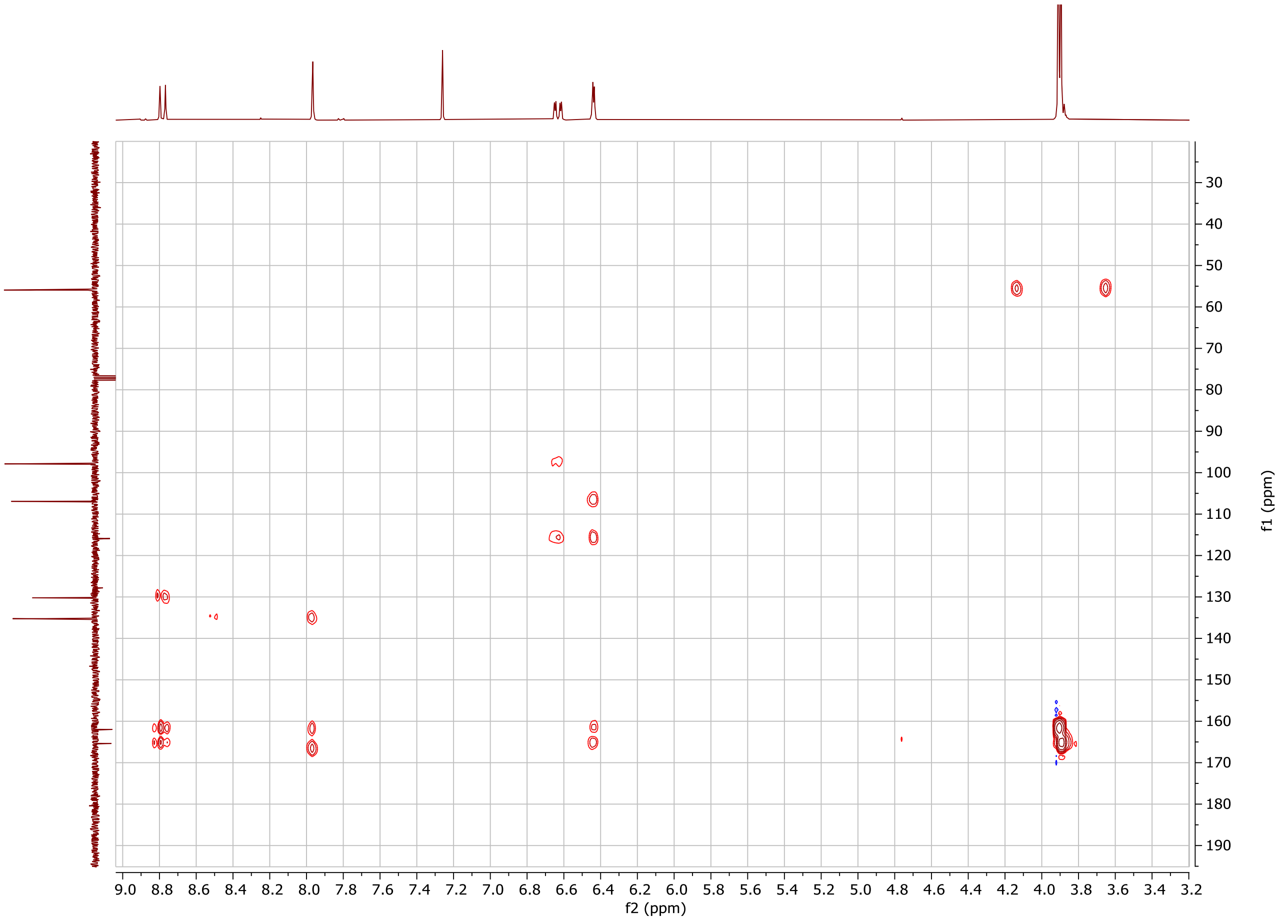

^{1}H - ^{13}C HMBC correlation spectrum of **1e**

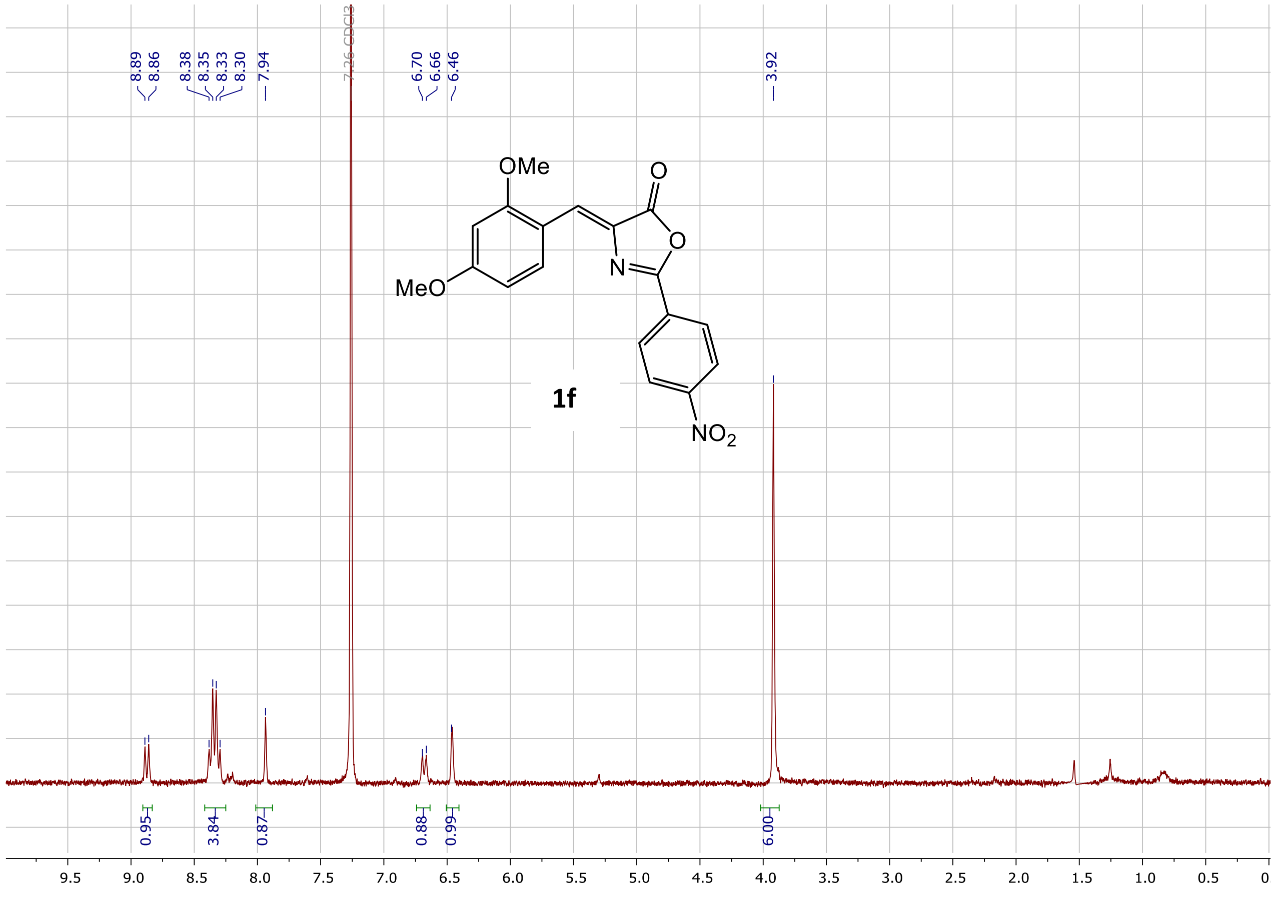

^{1}H-NMR spectrum ($CDCl_3$, 300.13 MHz) of **1f**[18e]

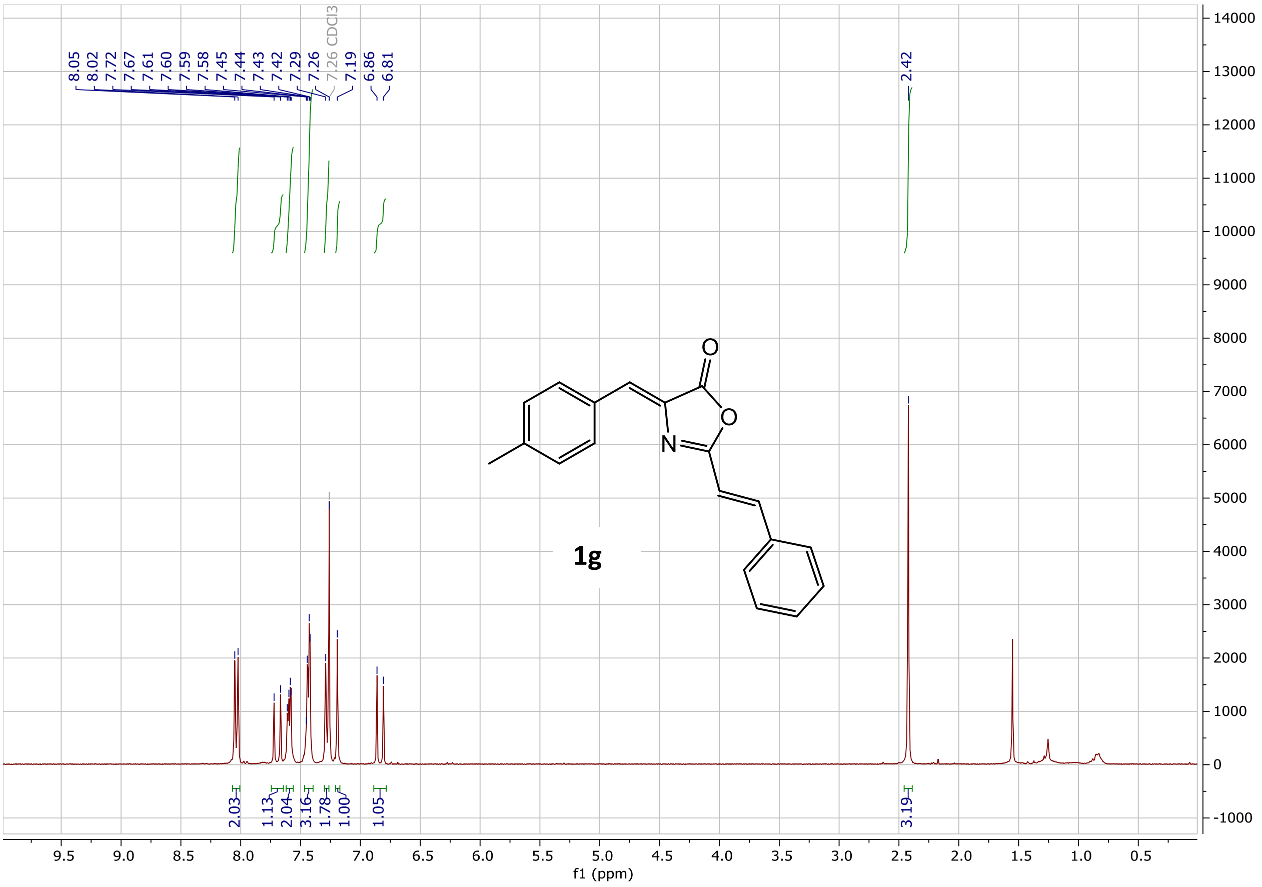

^{1}H-NMR spectrum ($CDCl_3$, 300.13 MHz) of **1g**[18f]

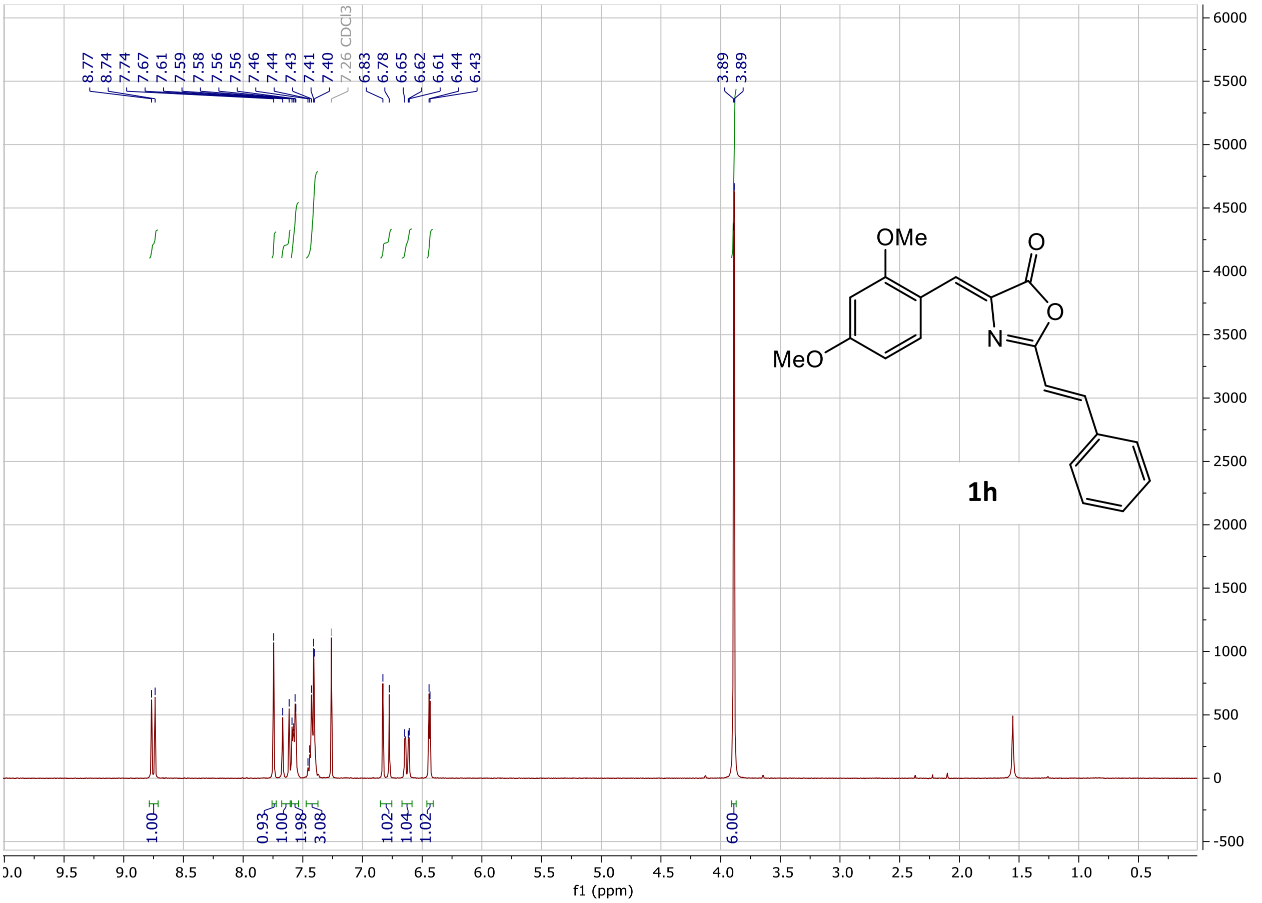

^{1}H-NMR spectrum ($CDCl_3$, 300.13 MHz) of **1h**[19b]

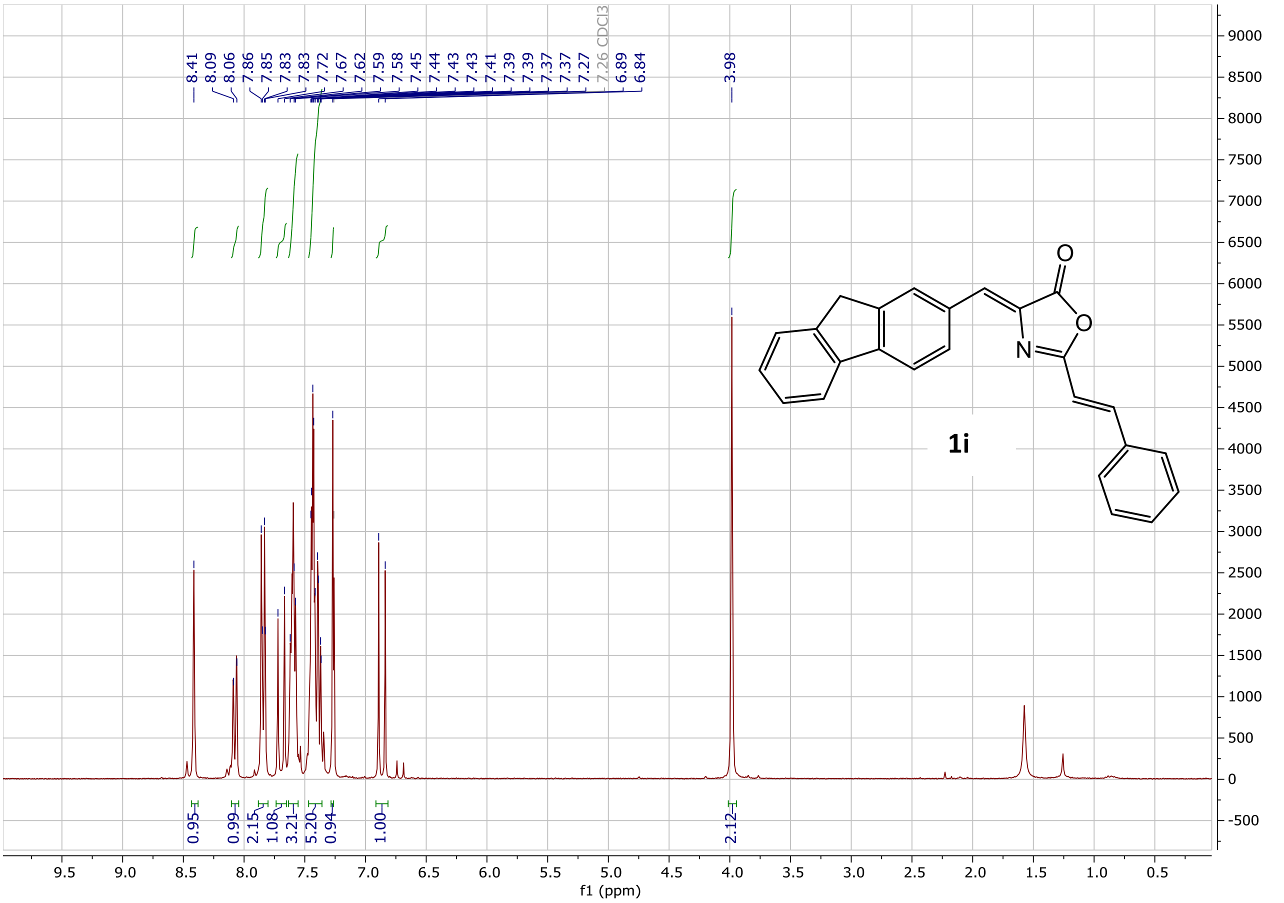

^{1}H-NMR spectrum ($CDCl_3$, 300.13 MHz) of **1i**

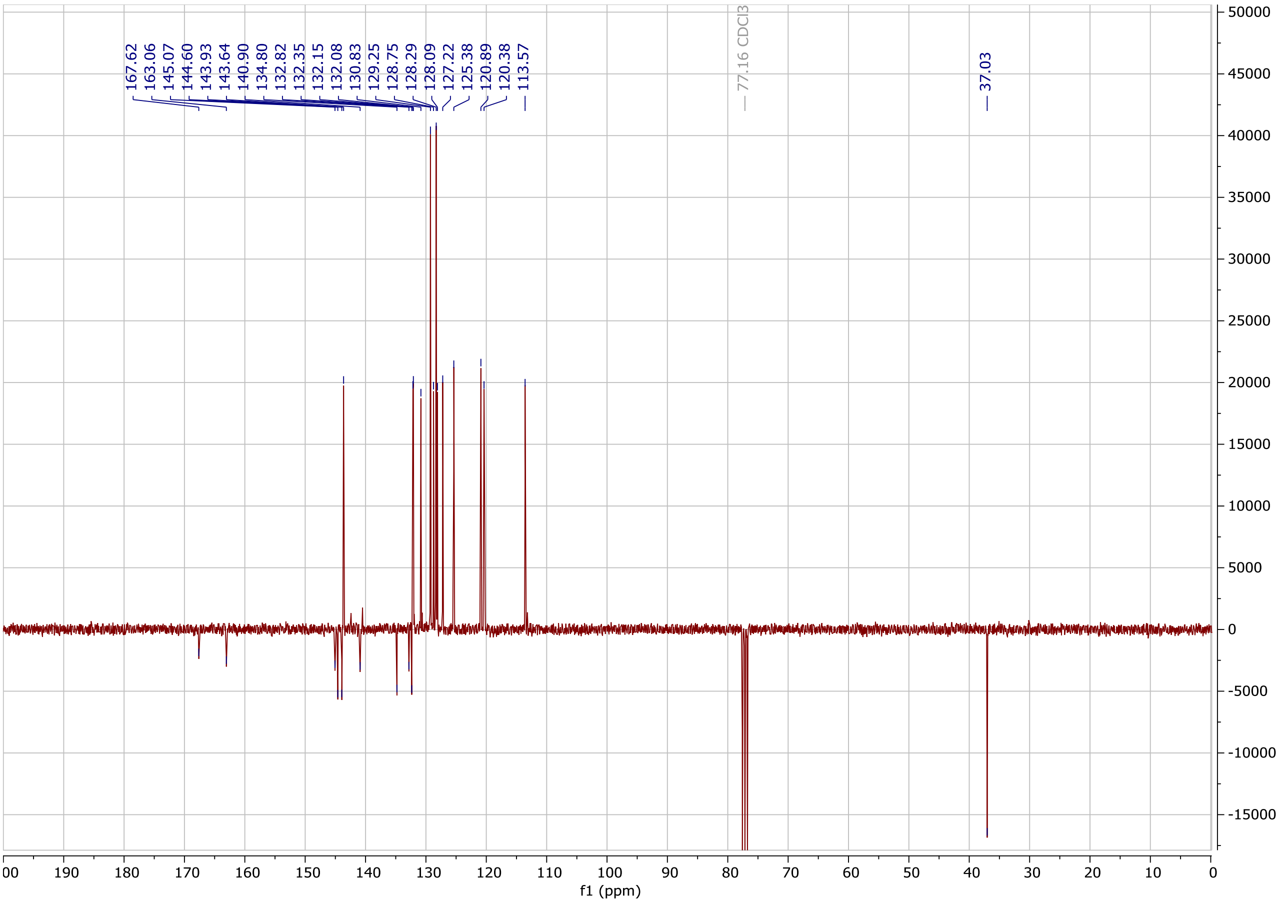

$^{13}C\{^{1}H\}$ (APT) NMR spectrum ($CDCl_3$, 75.47 MHz) of **1i**

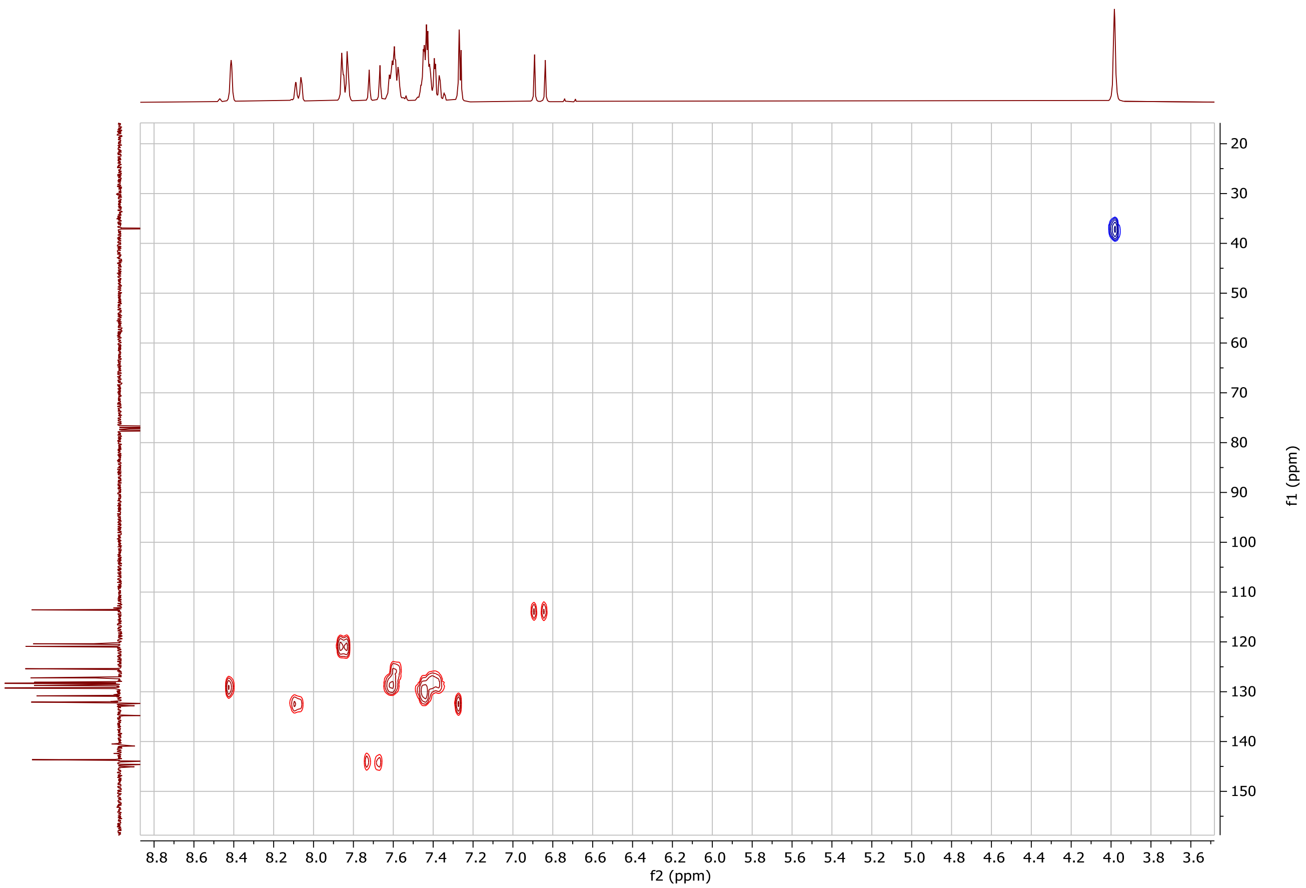

^{1}H - ^{13}C HSQC correlation spectrum of **1i**

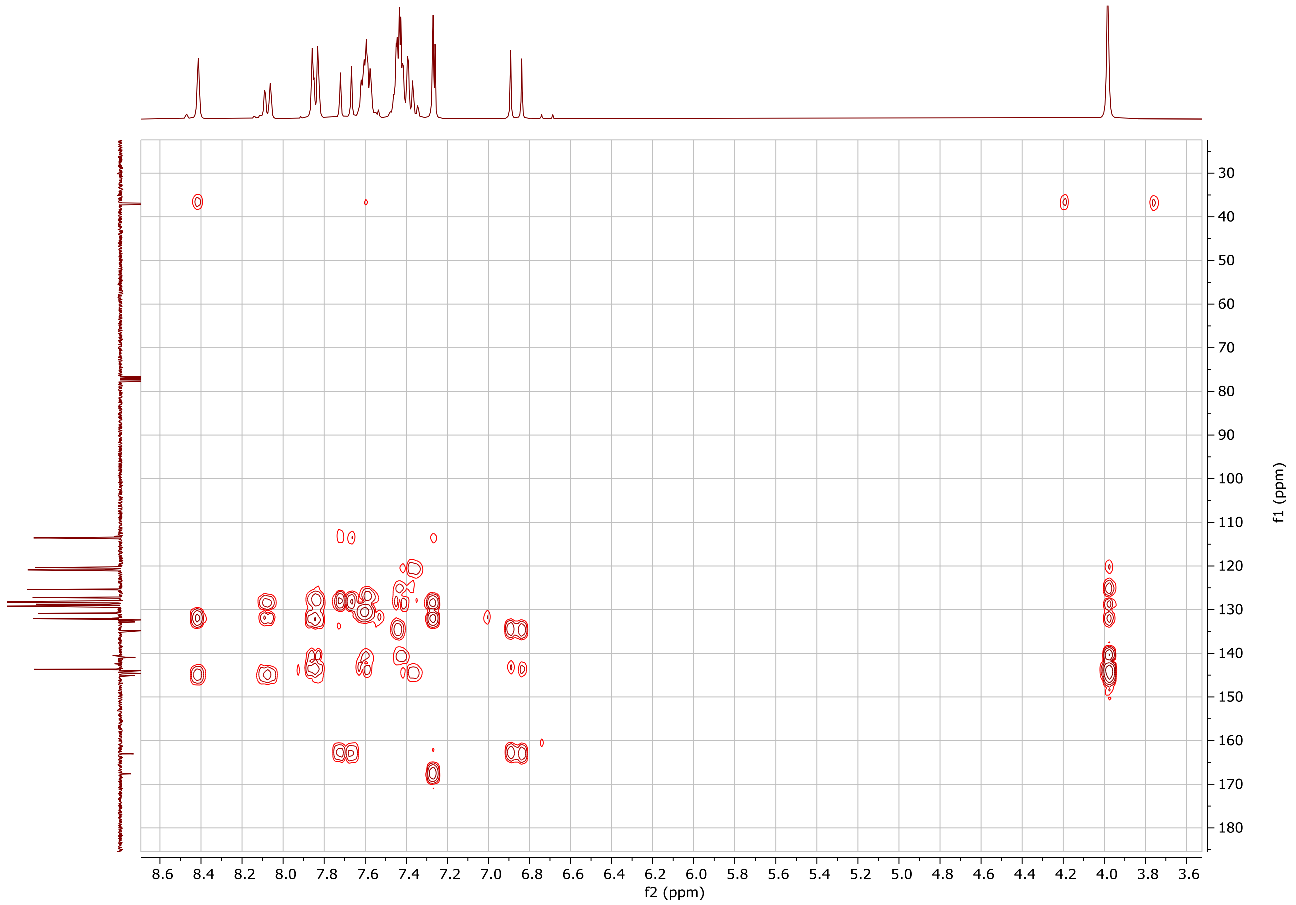

^{1}H - ^{13}C HMBC correlation spectrum of **1i**

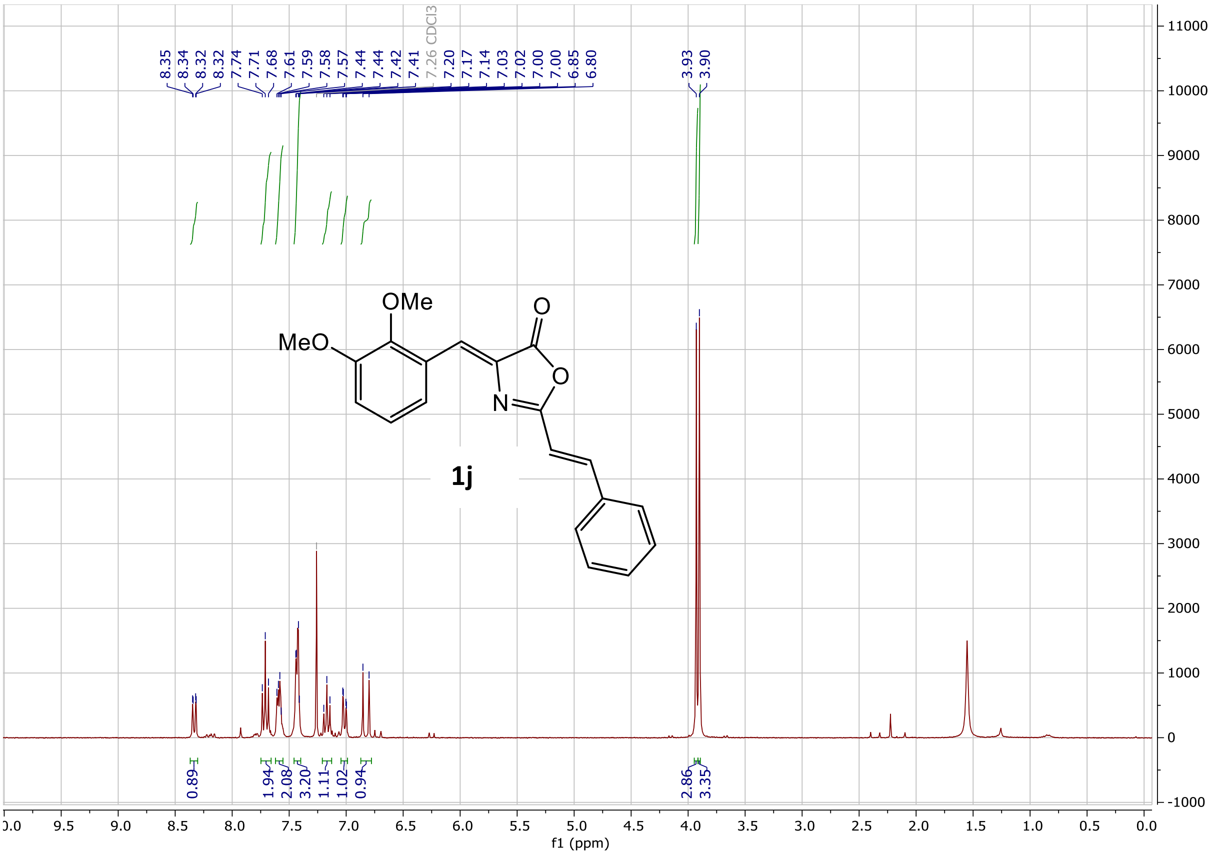

^{1}H-NMR spectrum (CDCl$_3$, 300.13 MHz) of **1j**

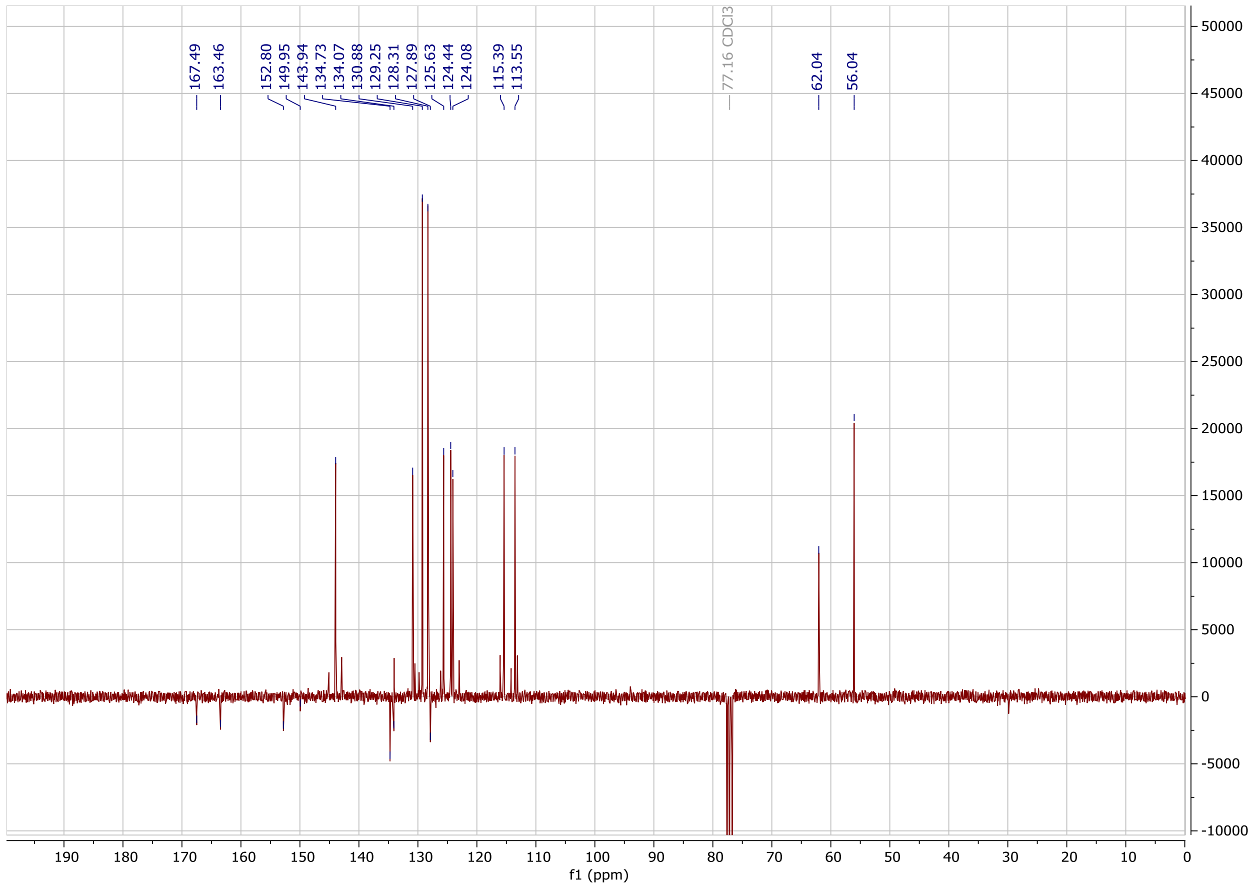

^{13}C{^{1}H} (APT) NMR spectrum (CDCl$_3$, 75.47 MHz) of **1j**

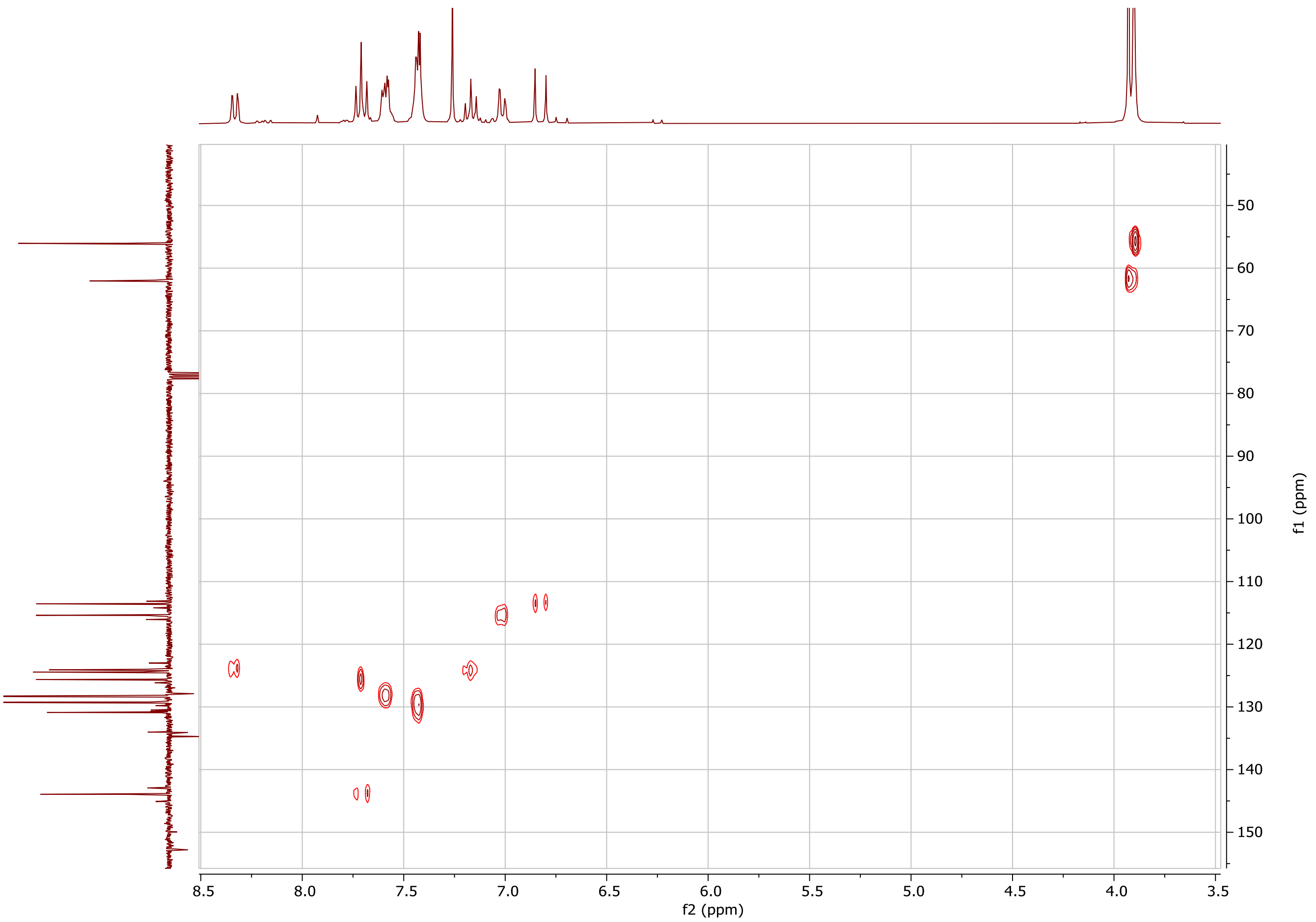

^{1}H - ^{13}C HSQC correlation spectrum of **1j**

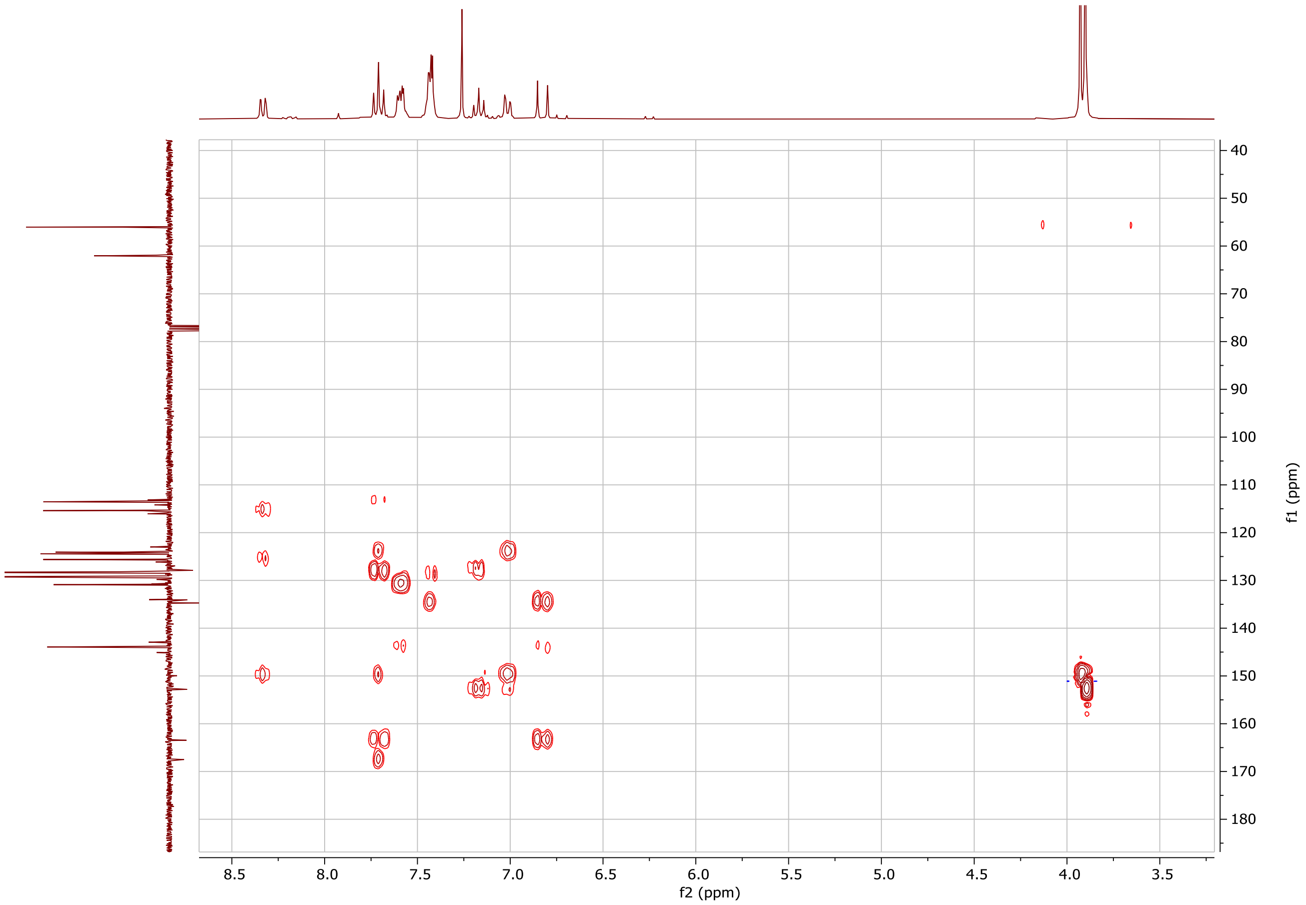

^{1}H - ^{13}C HMBC correlation spectrum of **1j**

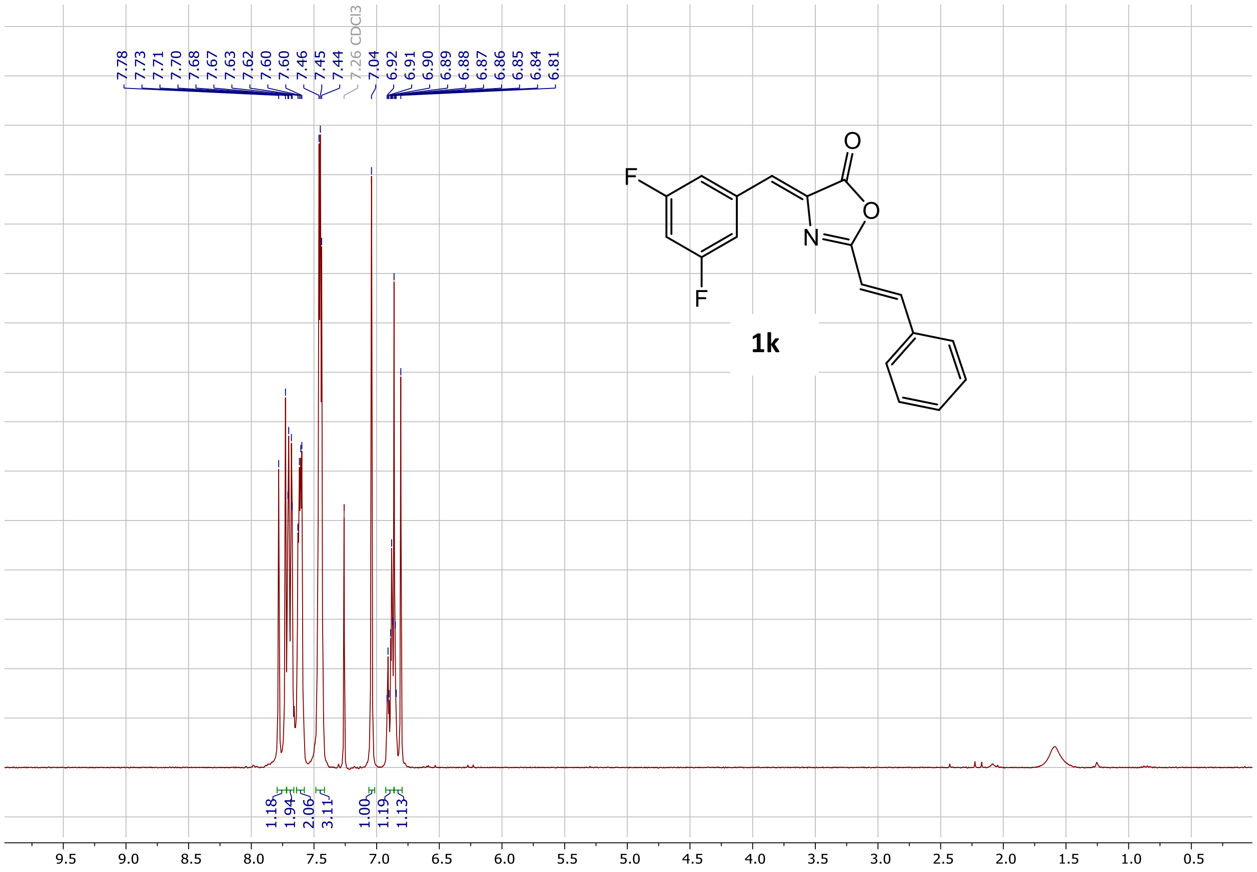

^{1}H-NMR spectrum ($CDCl_3$, 300.13 MHz) of **1k**

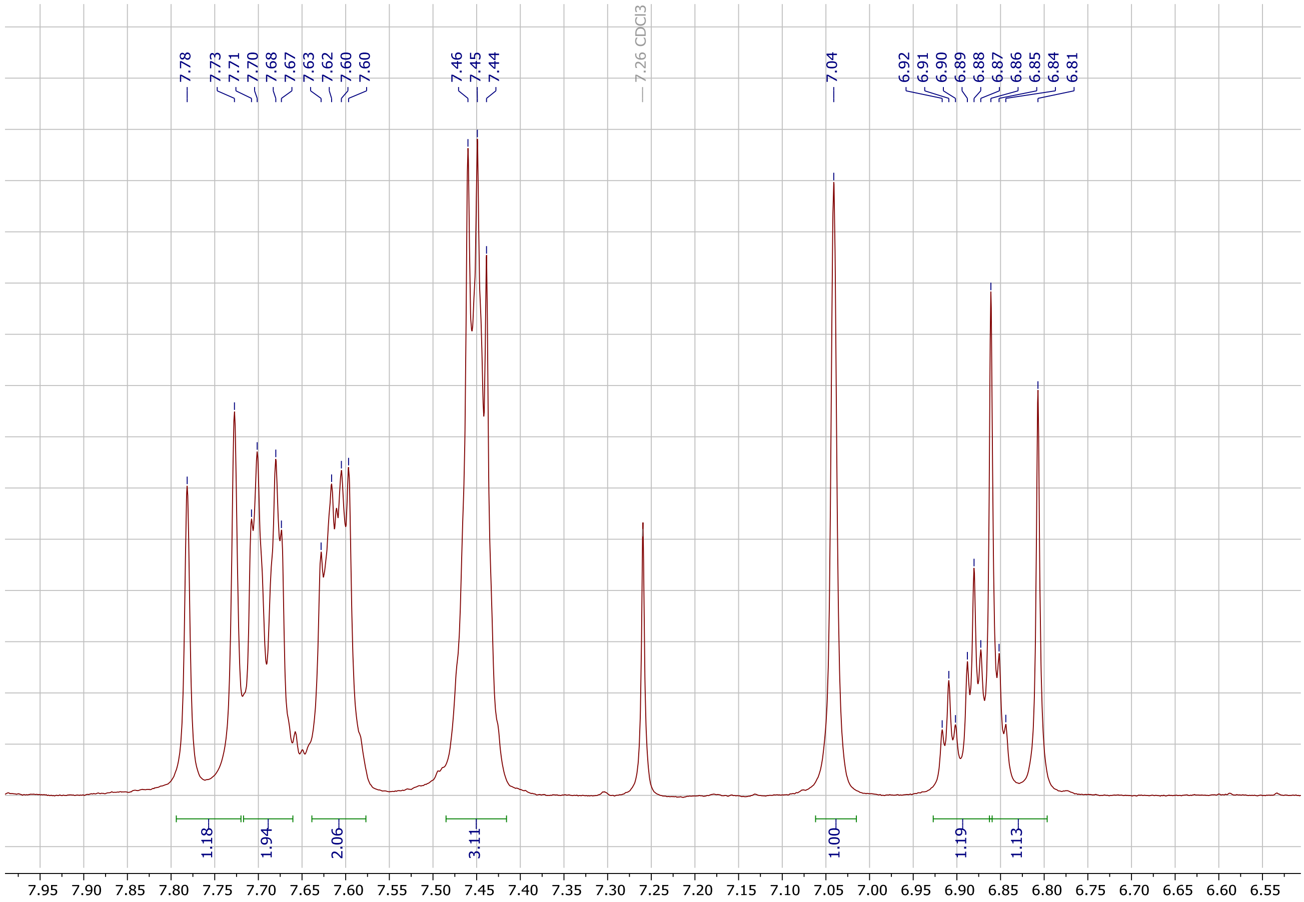

^{1}H-NMR spectrum ($CDCl_3$, 300.13 MHz) of **1k** (zoom aromatics)

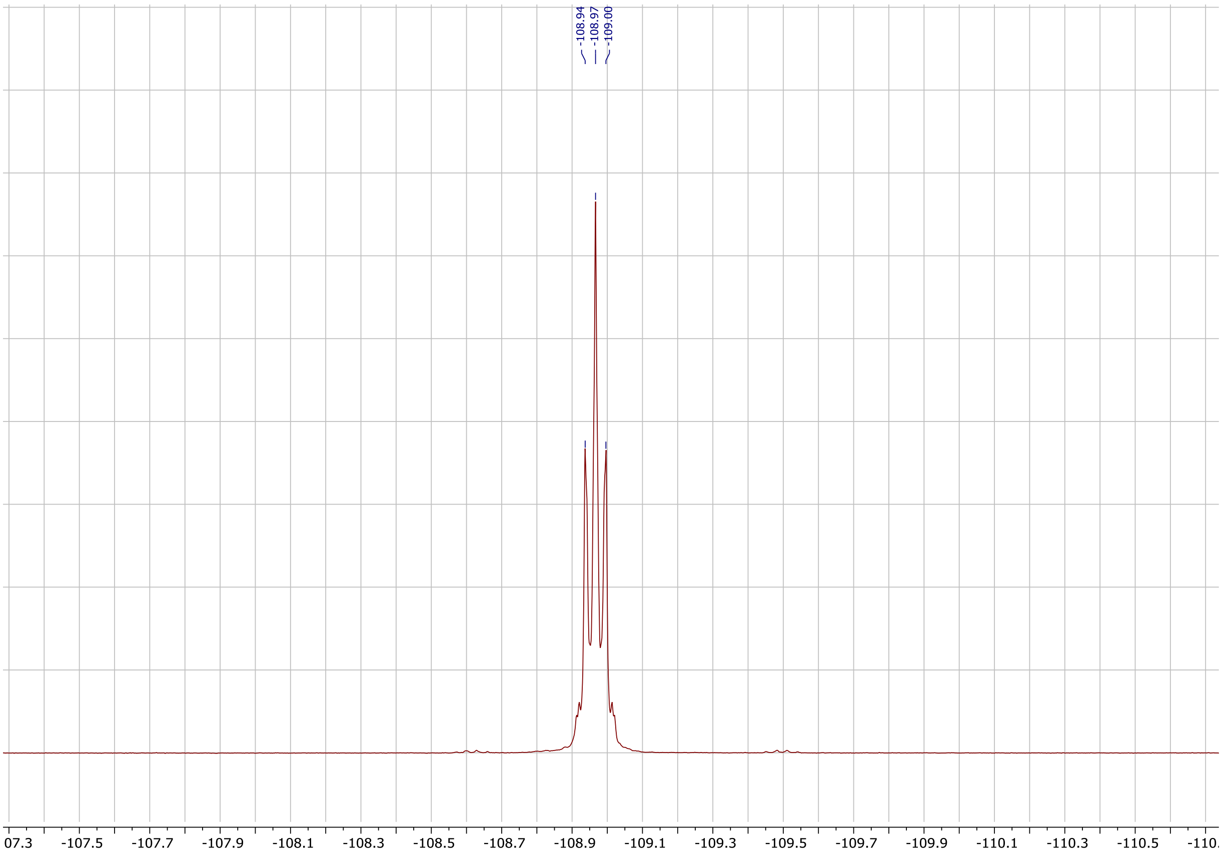

^{19}F-NMR spectrum ($CDCl_3$, 282.40 MHz) of **1k**

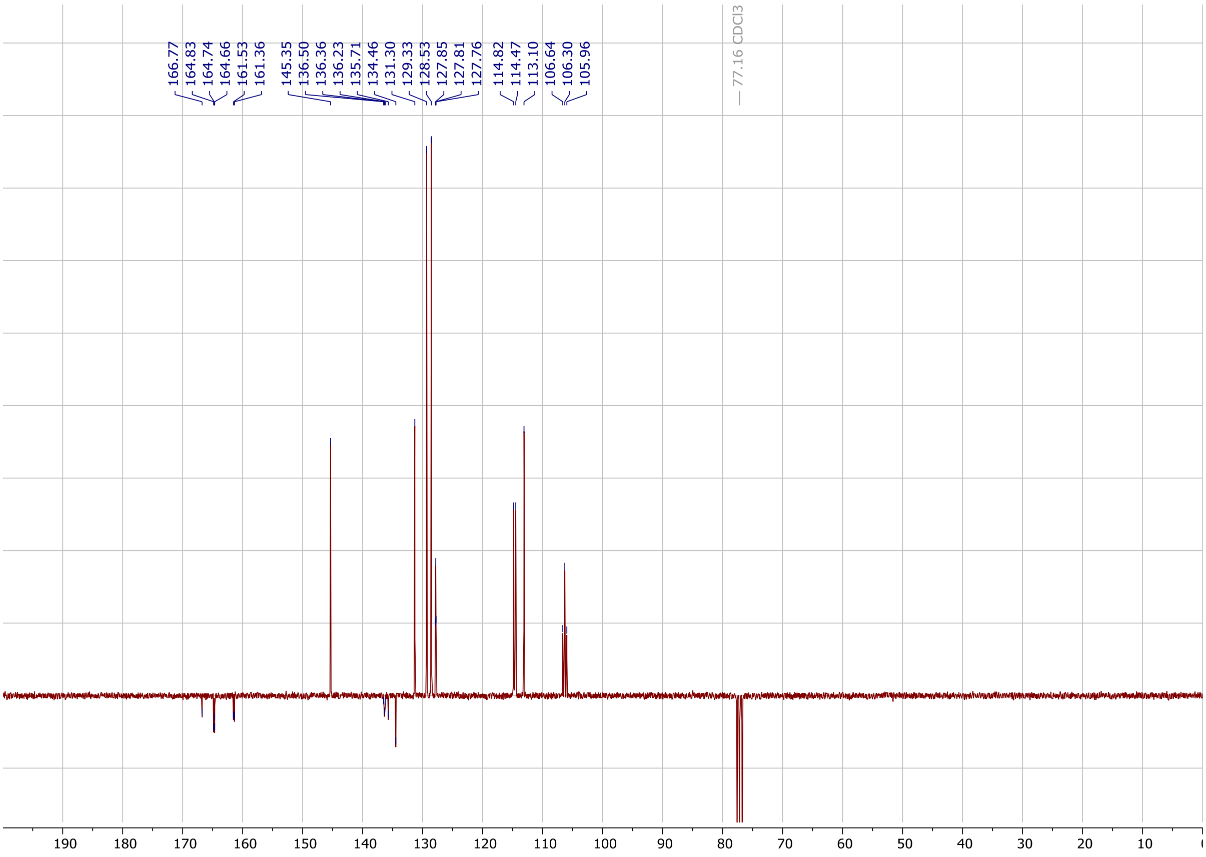

^{13}C{^{1}H} (APT) NMR spectrum ($CDCl_3$, 75.47 MHz) of **1k**

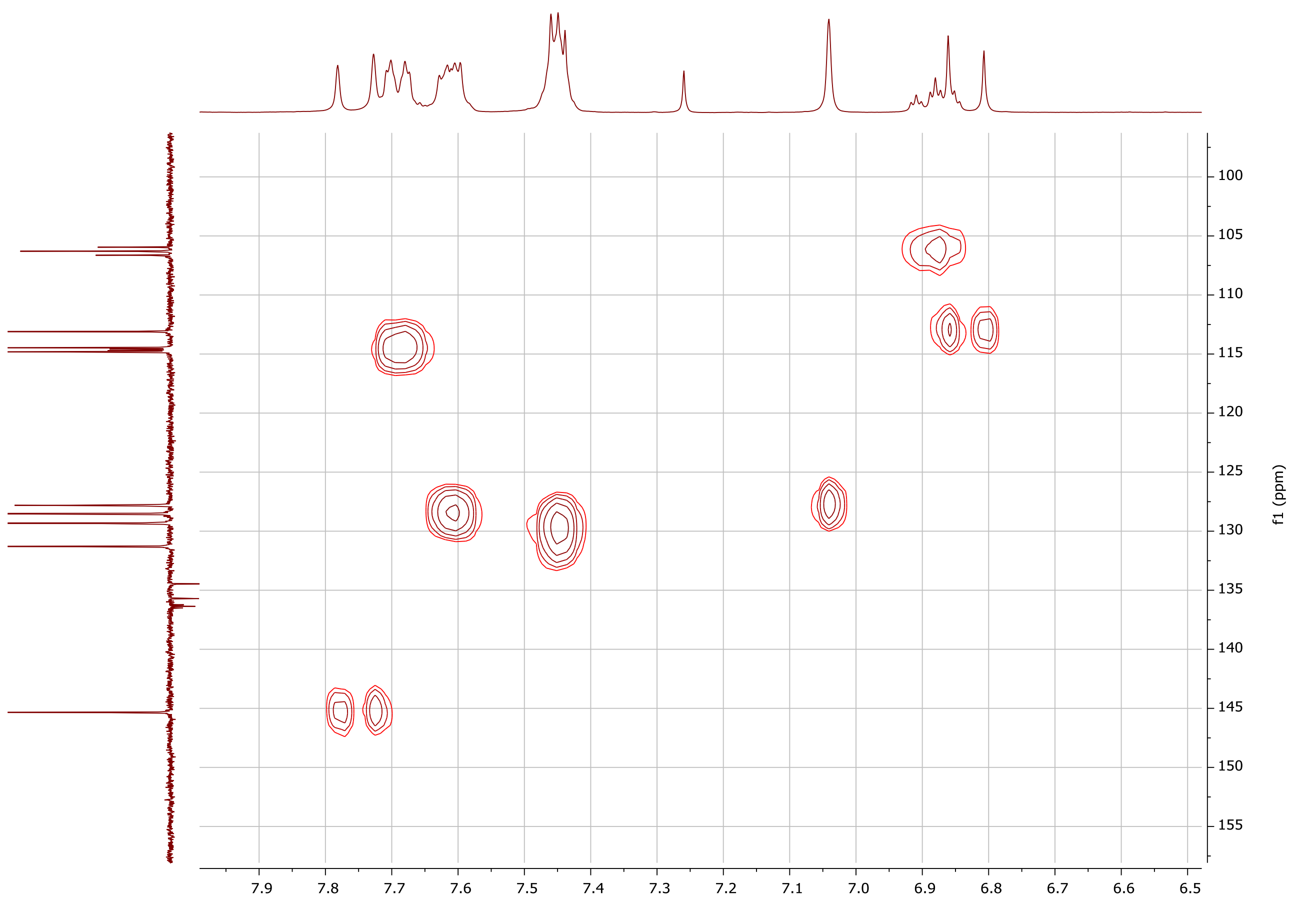

^{1}H - ^{13}C HSQC correlation spectrum of **1k**

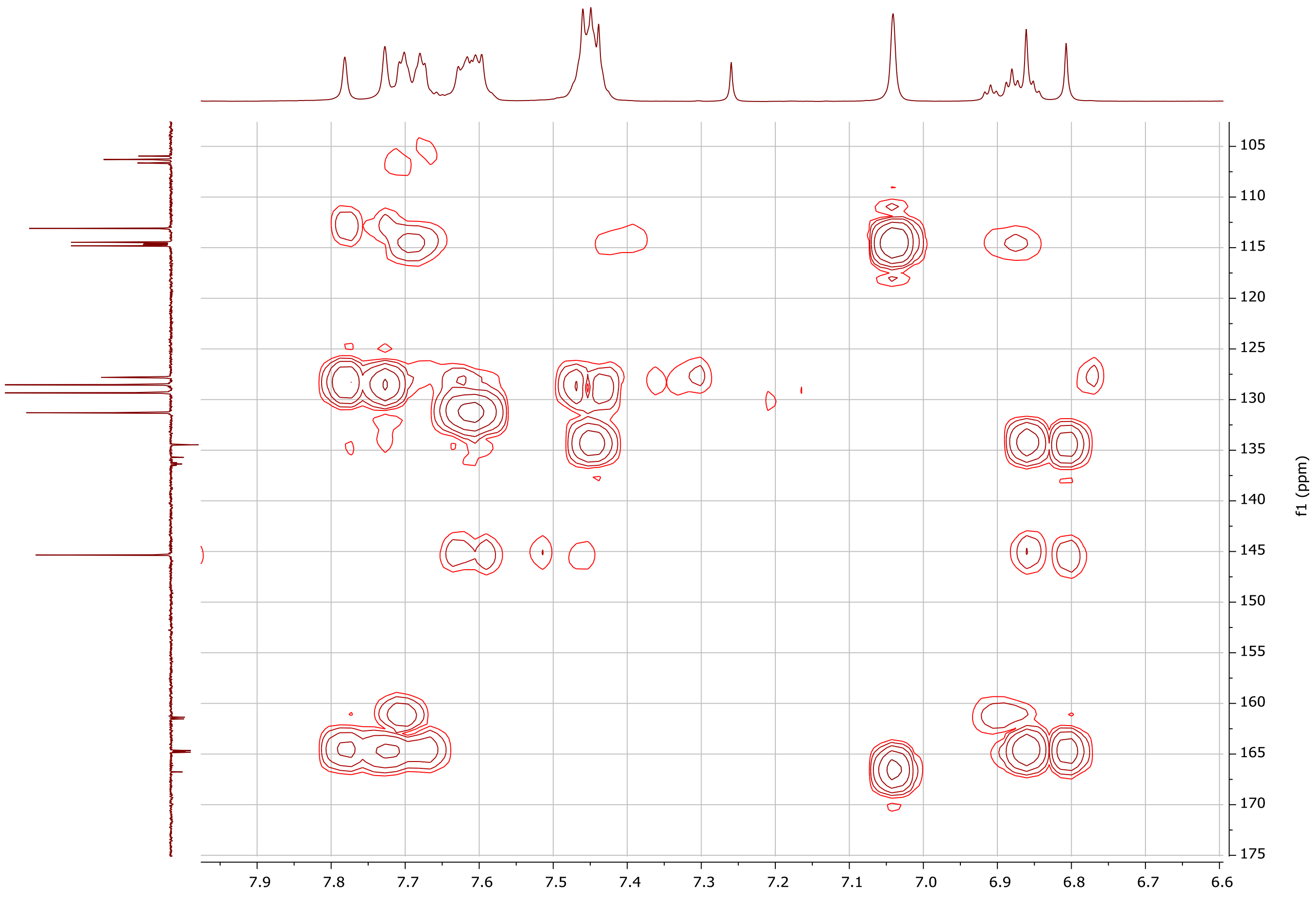

^{1}H - ^{13}C HMBC correlation spectrum of **1k**

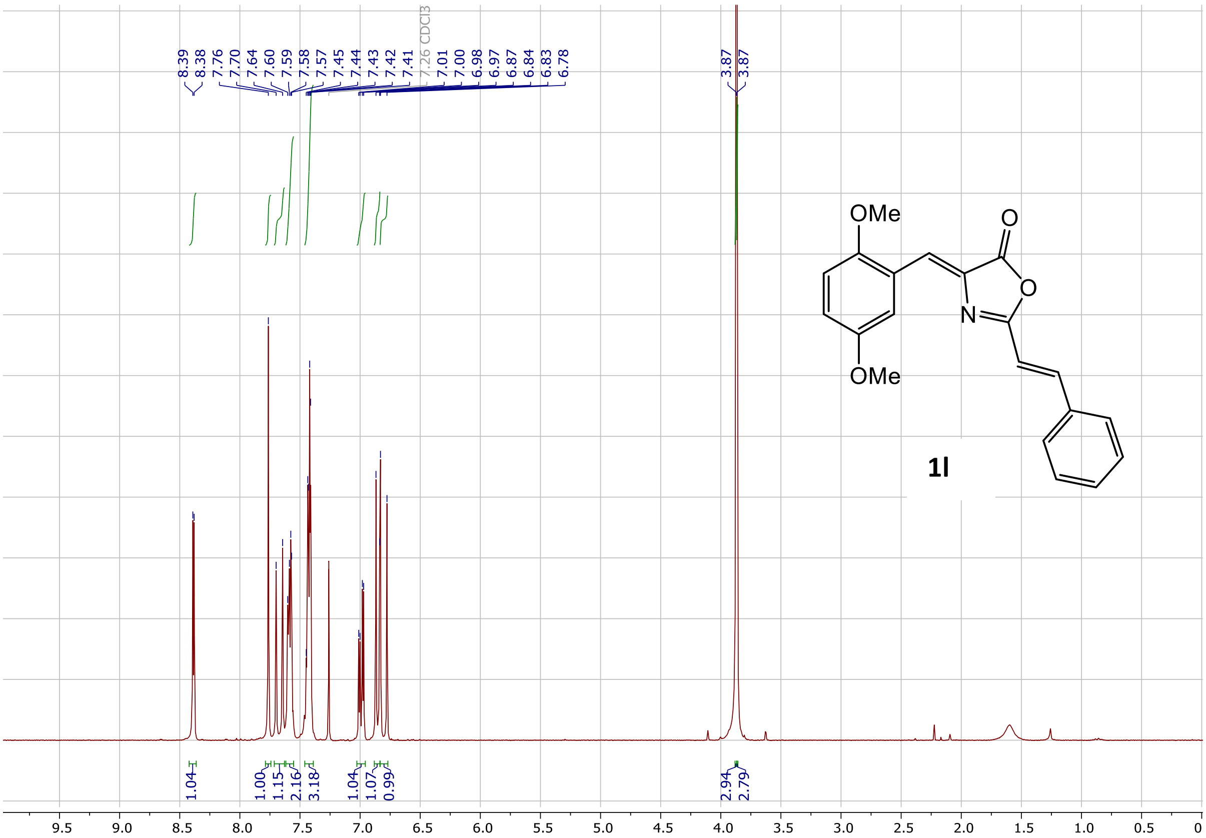

^{1}H-NMR spectrum ($CDCl_3$, 300.13 MHz) of **1l**

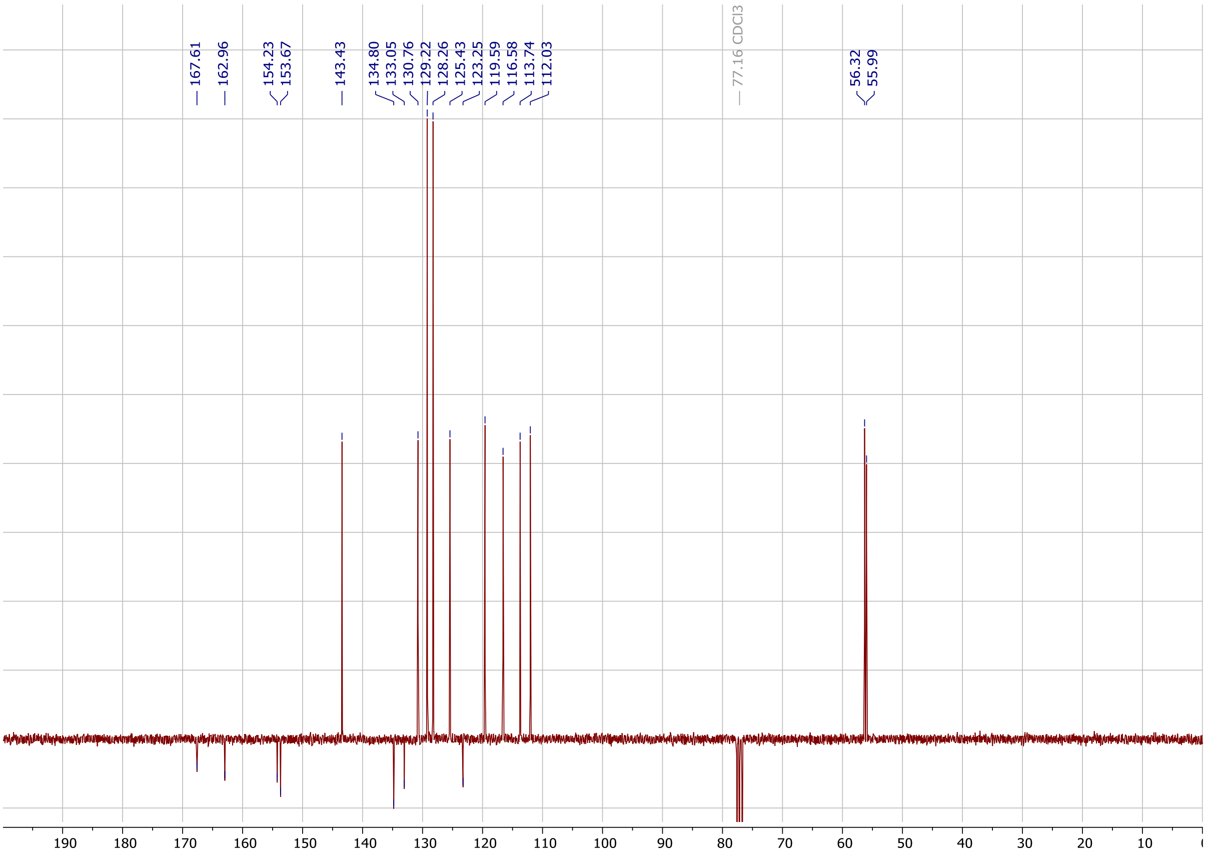

$^{13}C\{^{1}H\}$ (APT) NMR spectrum ($CDCl_3$, 75.47 MHz) of **1l**

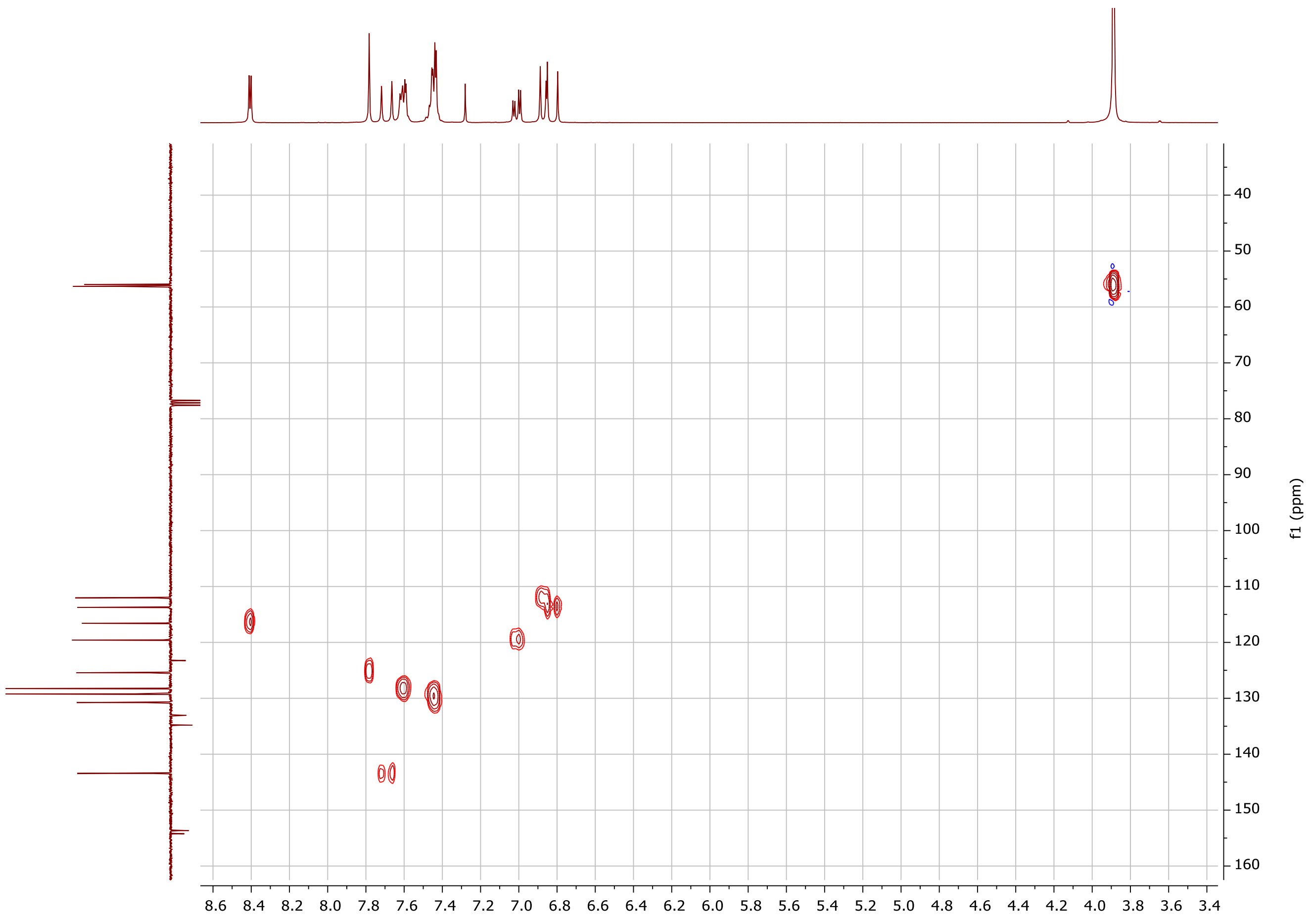

^{1}H - ^{13}C HSQC correlation spectrum of **1l**

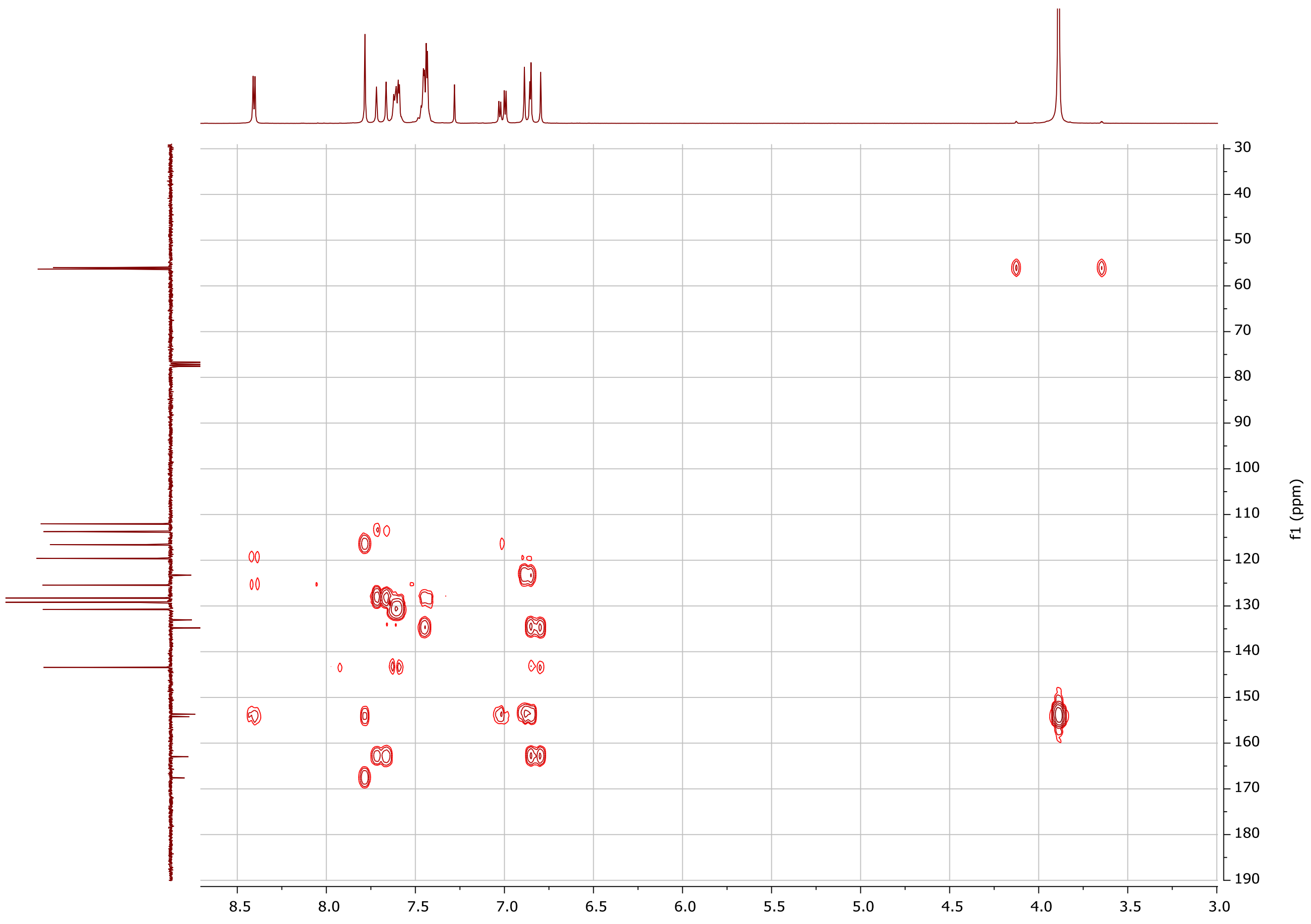

^{1}H - ^{13}C HMBC correlation spectrum of **1l**

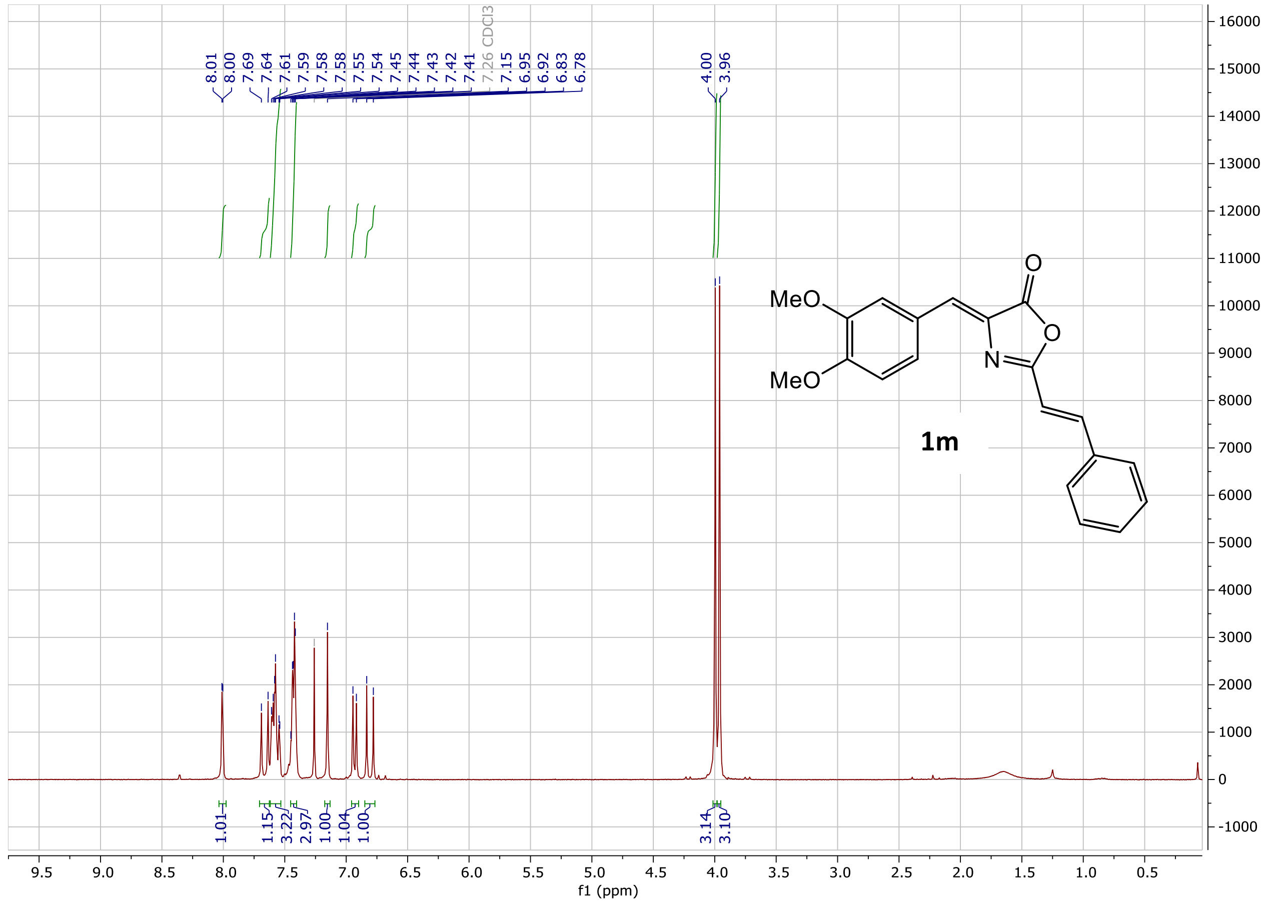

^{1}H-NMR spectrum (CDCl₃, 300.13 MHz) of **1m**

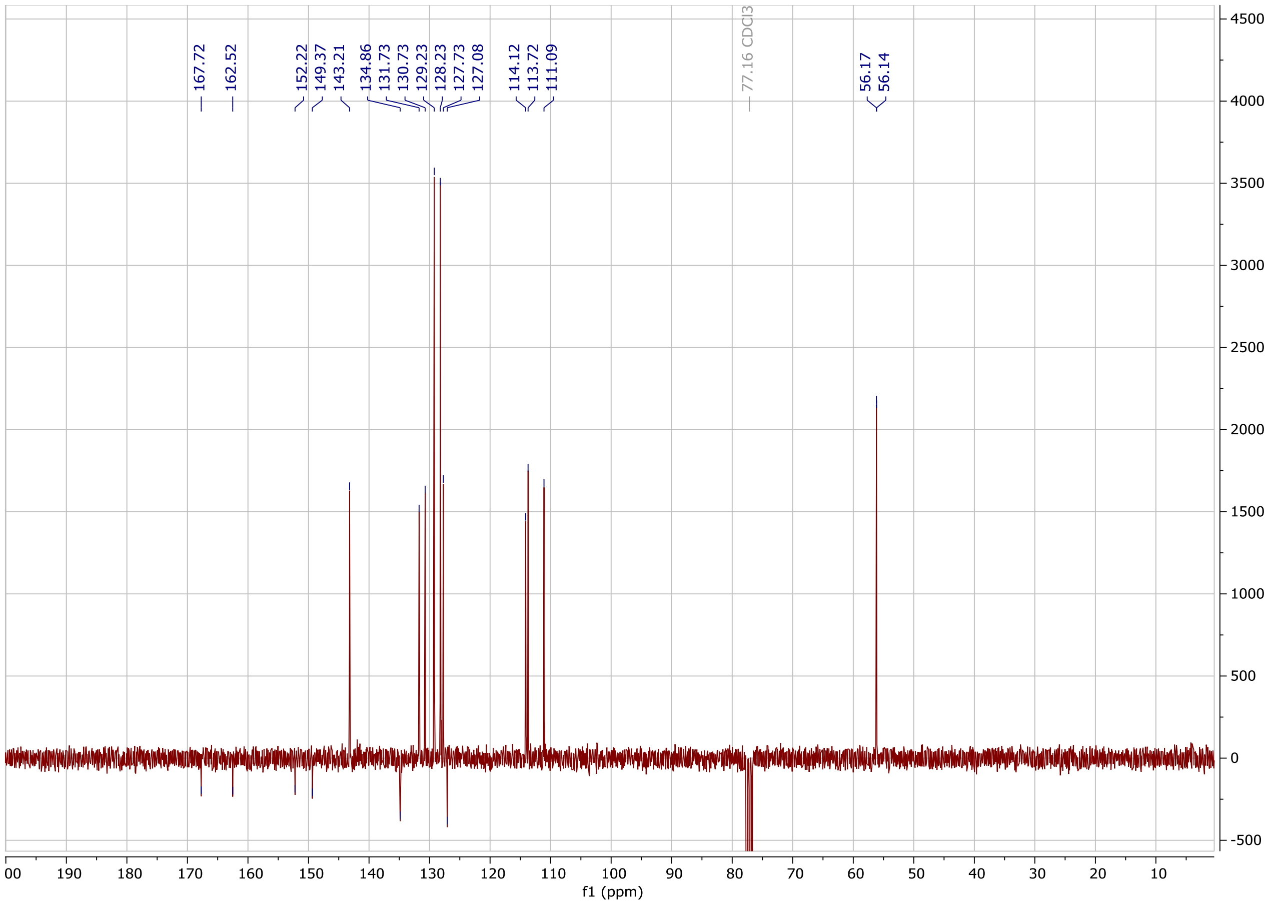

^{13}C{^{1}H} (APT) NMR spectrum (CDCl₃, 75.47 MHz) of **1m**

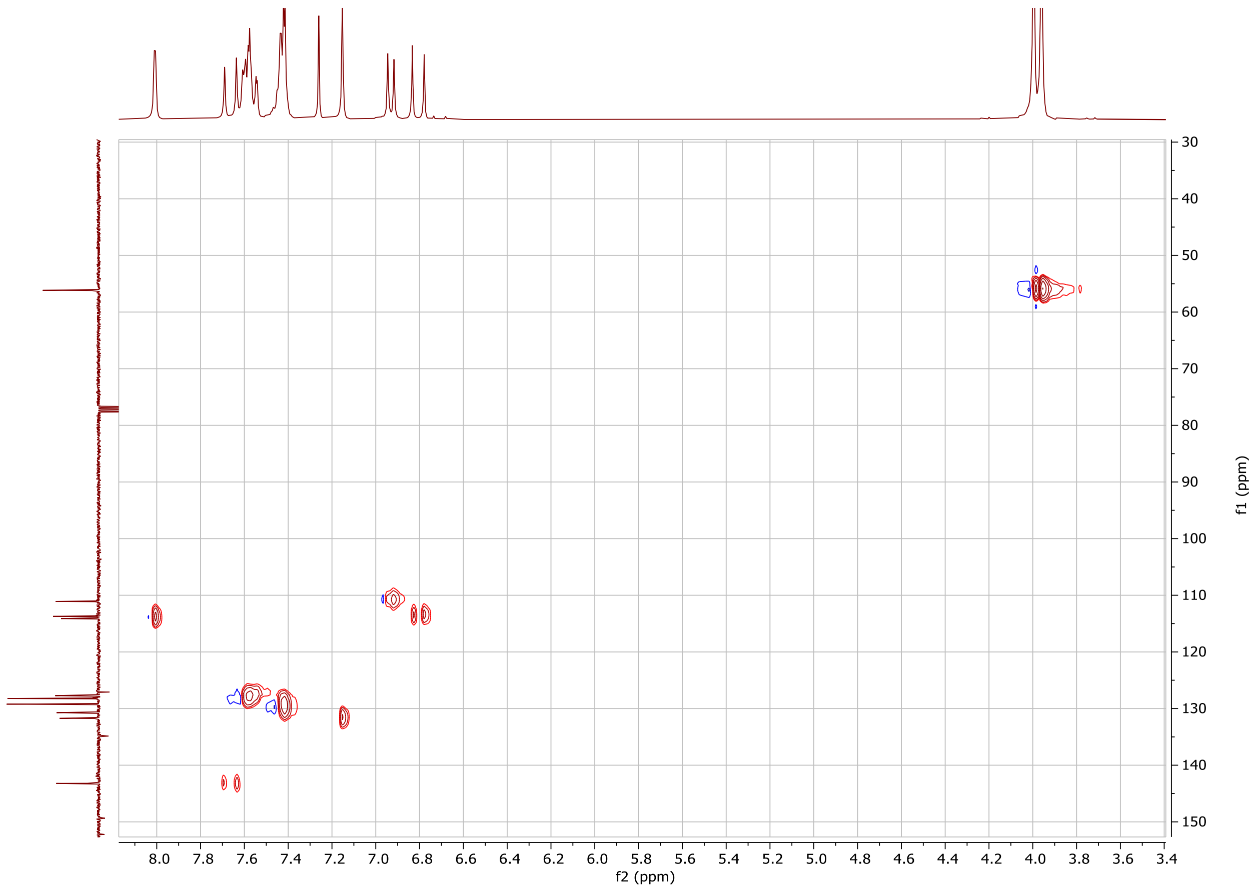

^{1}H - ^{13}C HSQC correlation spectrum of **1m**

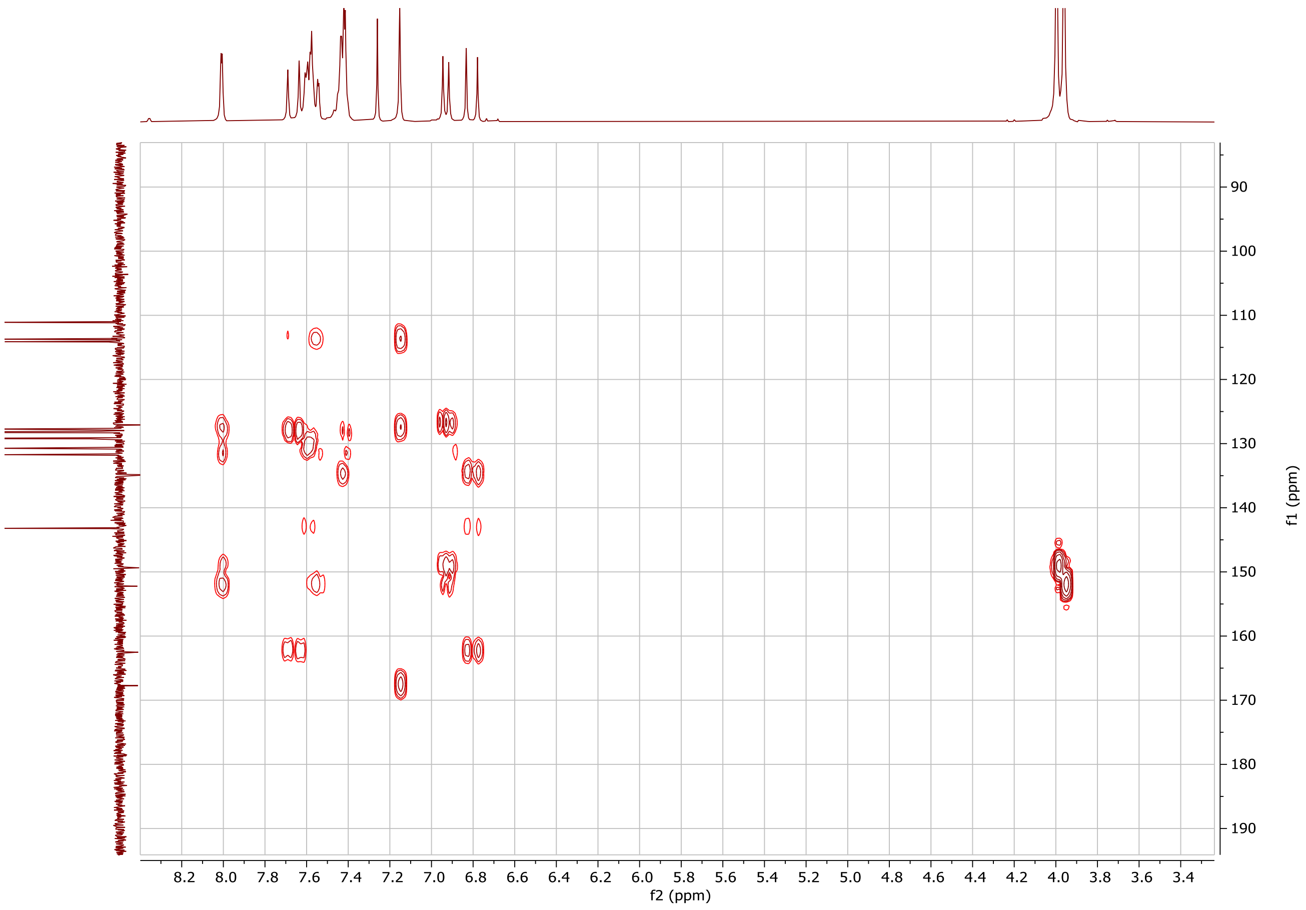

^{1}H - ^{13}C HMBC correlation spectrum of **1m**

3.- NMR spectra of dinuclear μ-trifluoroacetate-orthopalladated derivatives 2a-2m

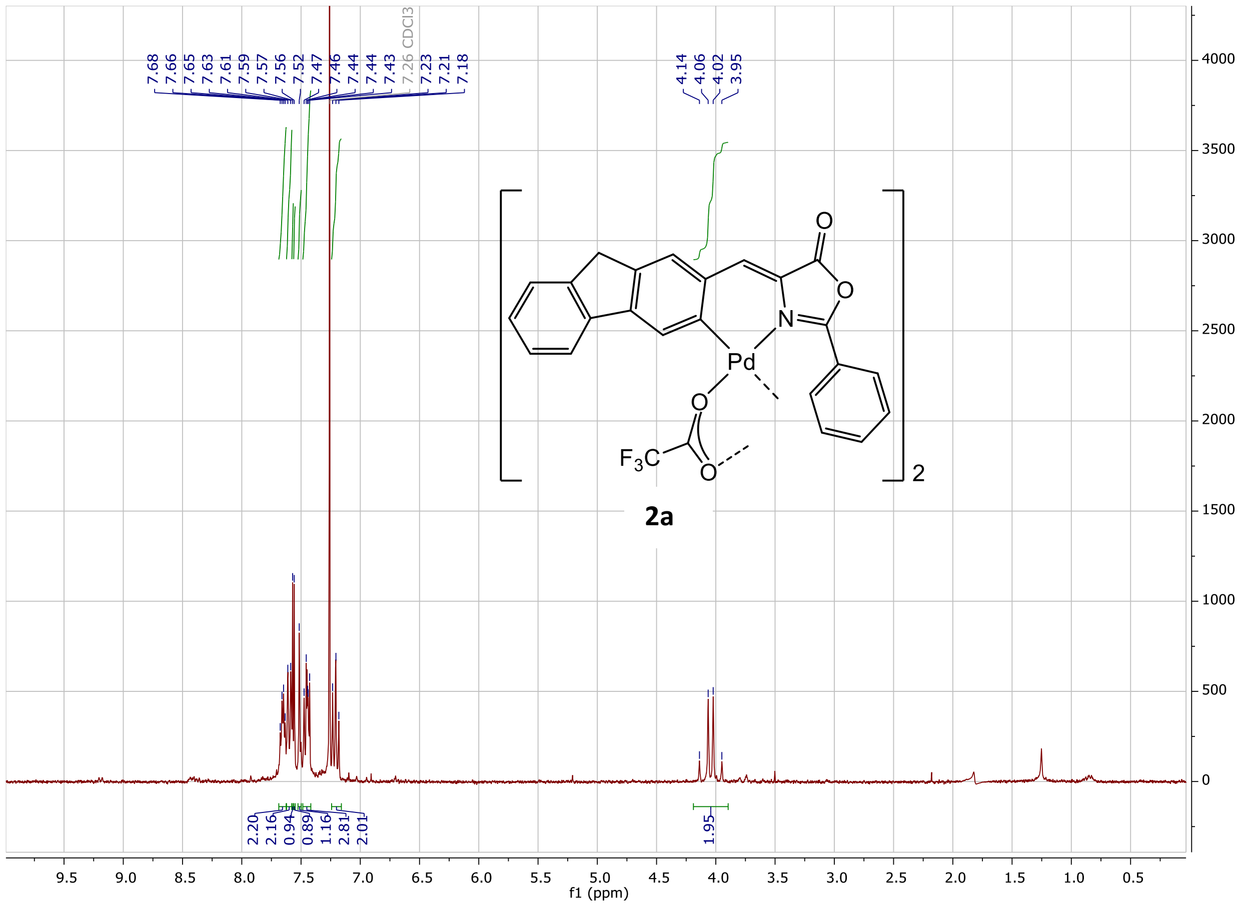

^{1}H-NMR spectrum ($CDCl_3$, 300.13 MHz) of **2a**

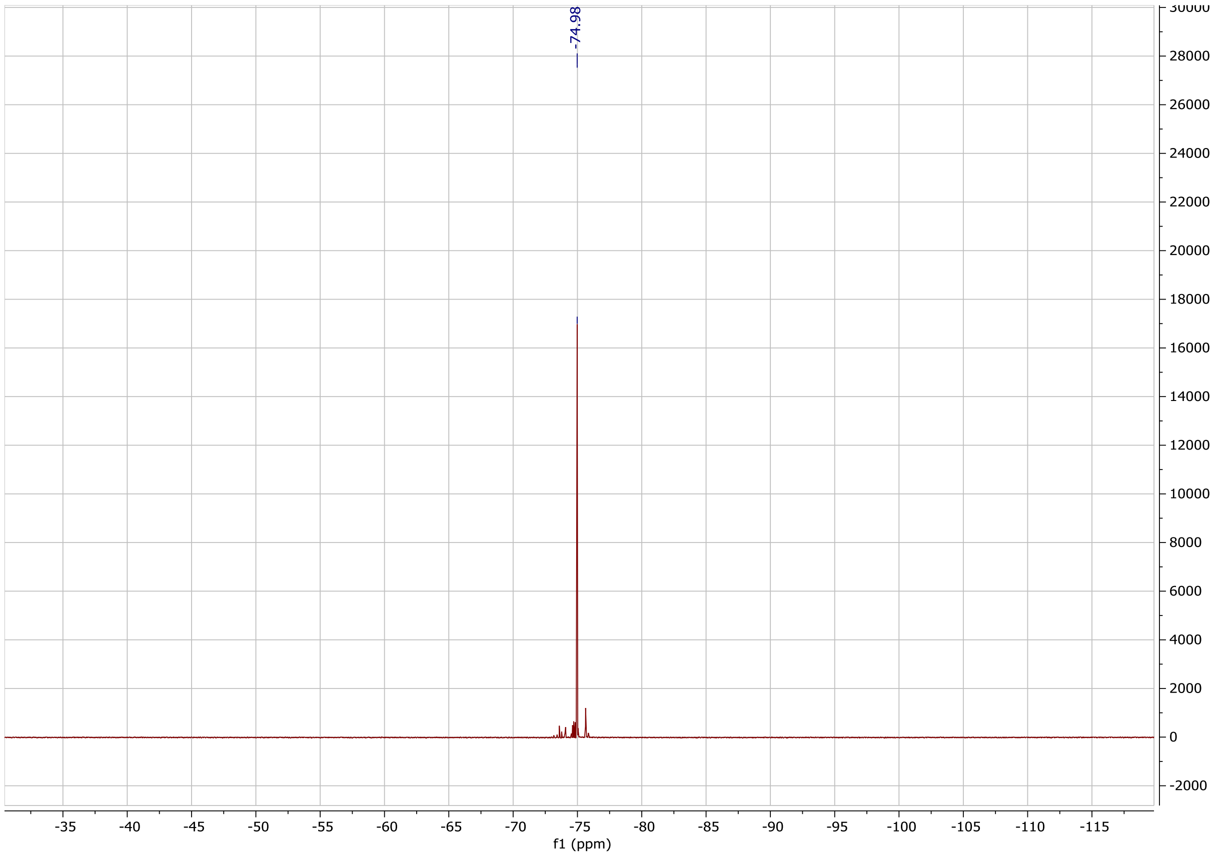

^{19}F-NMR spectrum ($CDCl_3$, 282.40 MHz) of **2a**

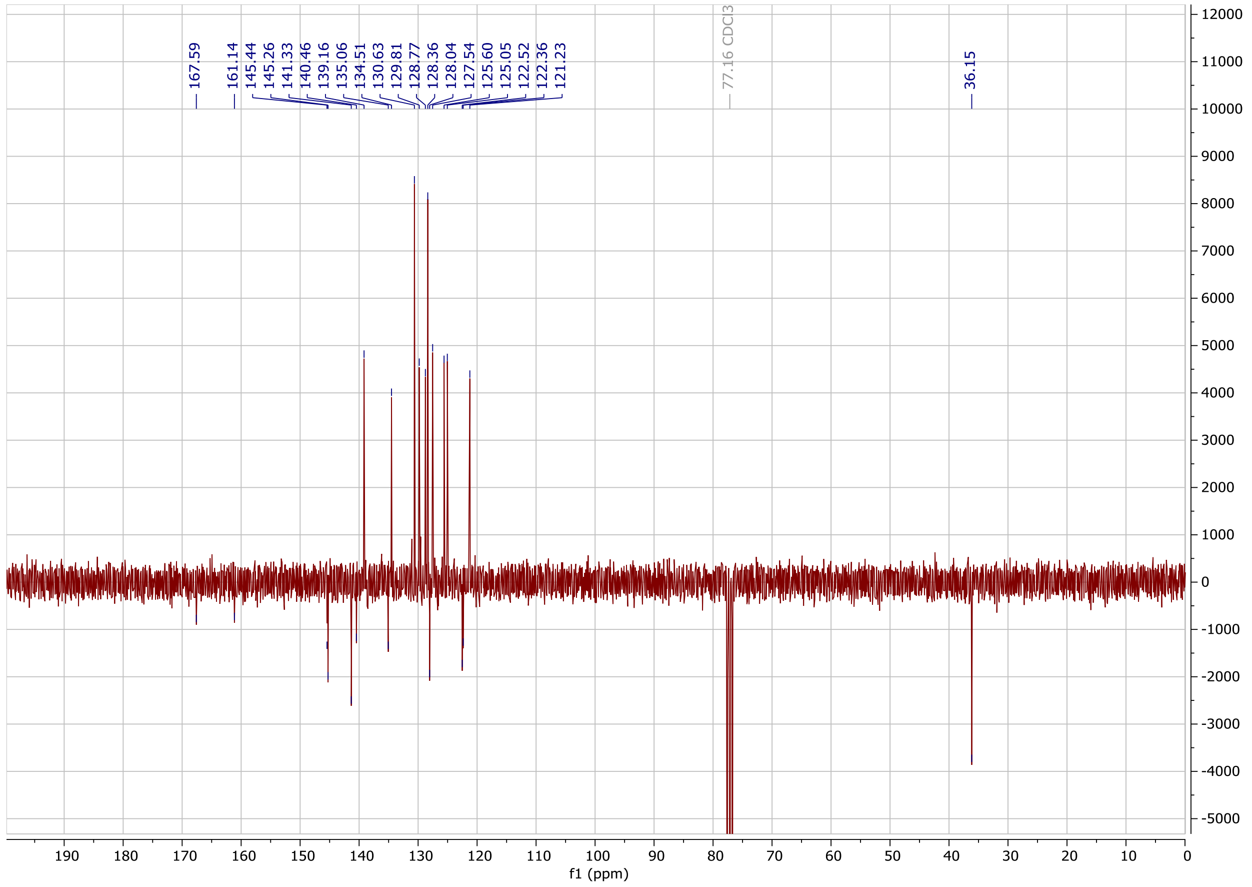

$^{13}C\{^1H\}$ (APT) NMR spectrum ($CDCl_3$, 75.47 MHz) of **2a**

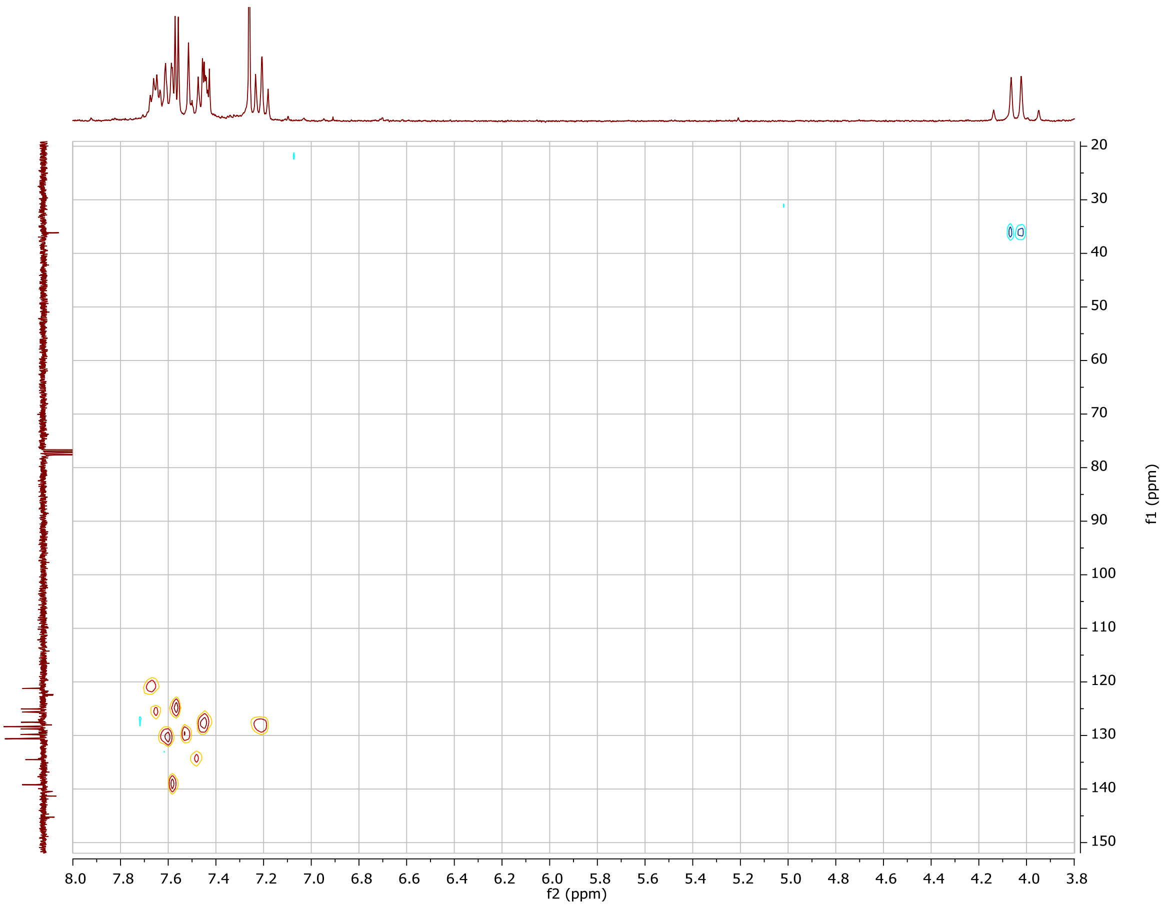

^1H - ^{13}C HSQC correlation spectrum of **2a**

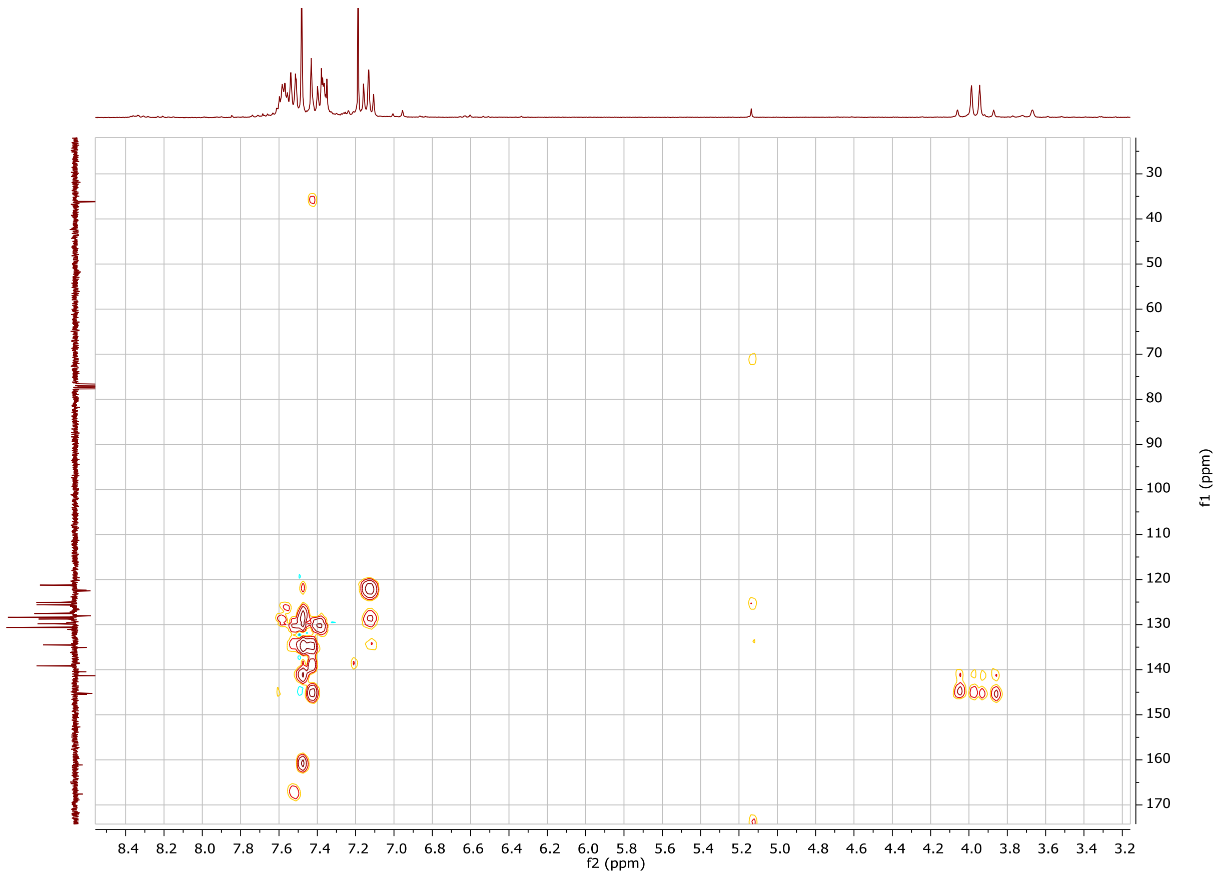

^{1}H - ^{13}C HMBC correlation spectrum of **2a**

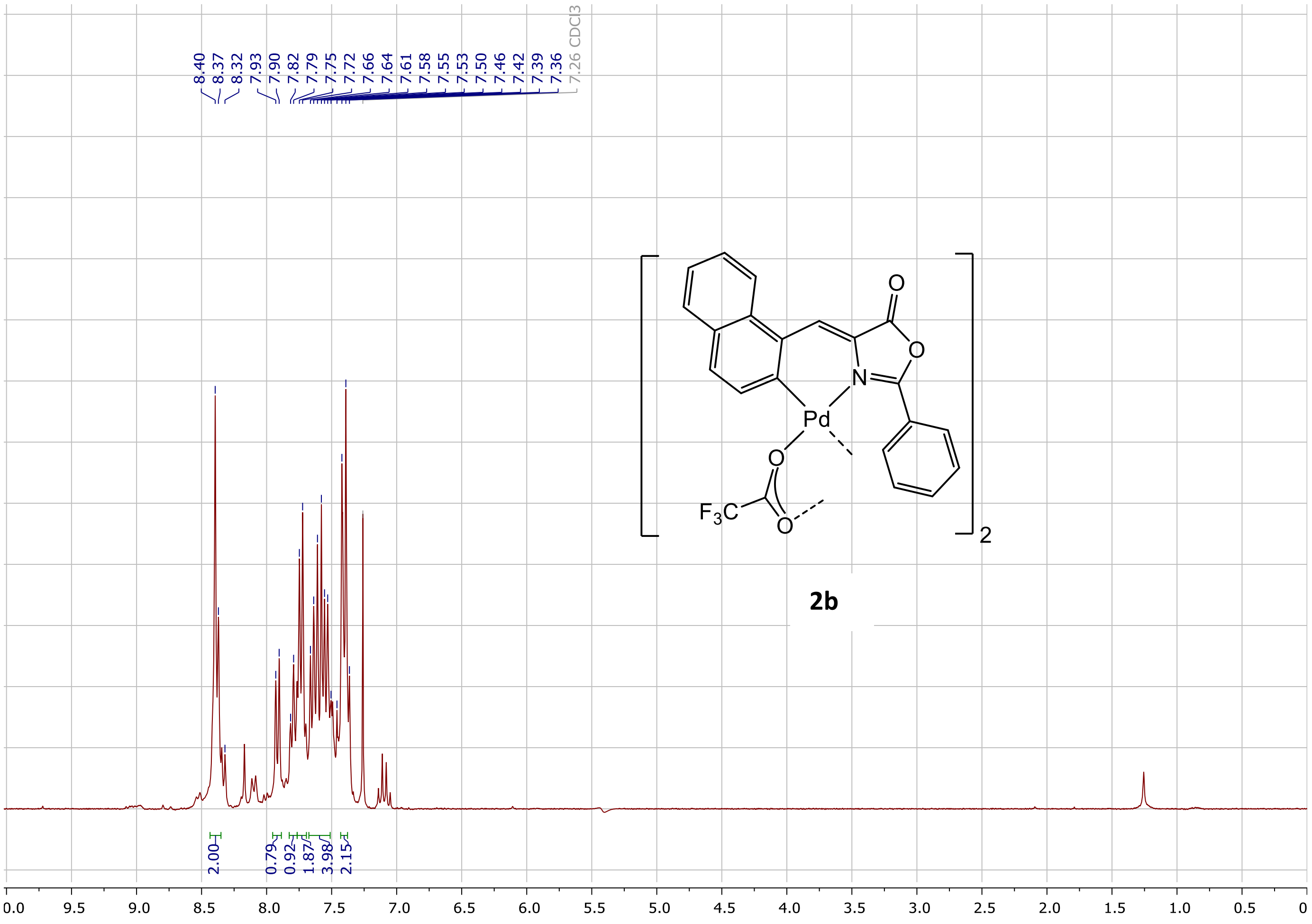

^{1}H-NMR spectrum ($CDCl_3$, 300.13 MHz) of **2b** (mixture transoid/cisoid)

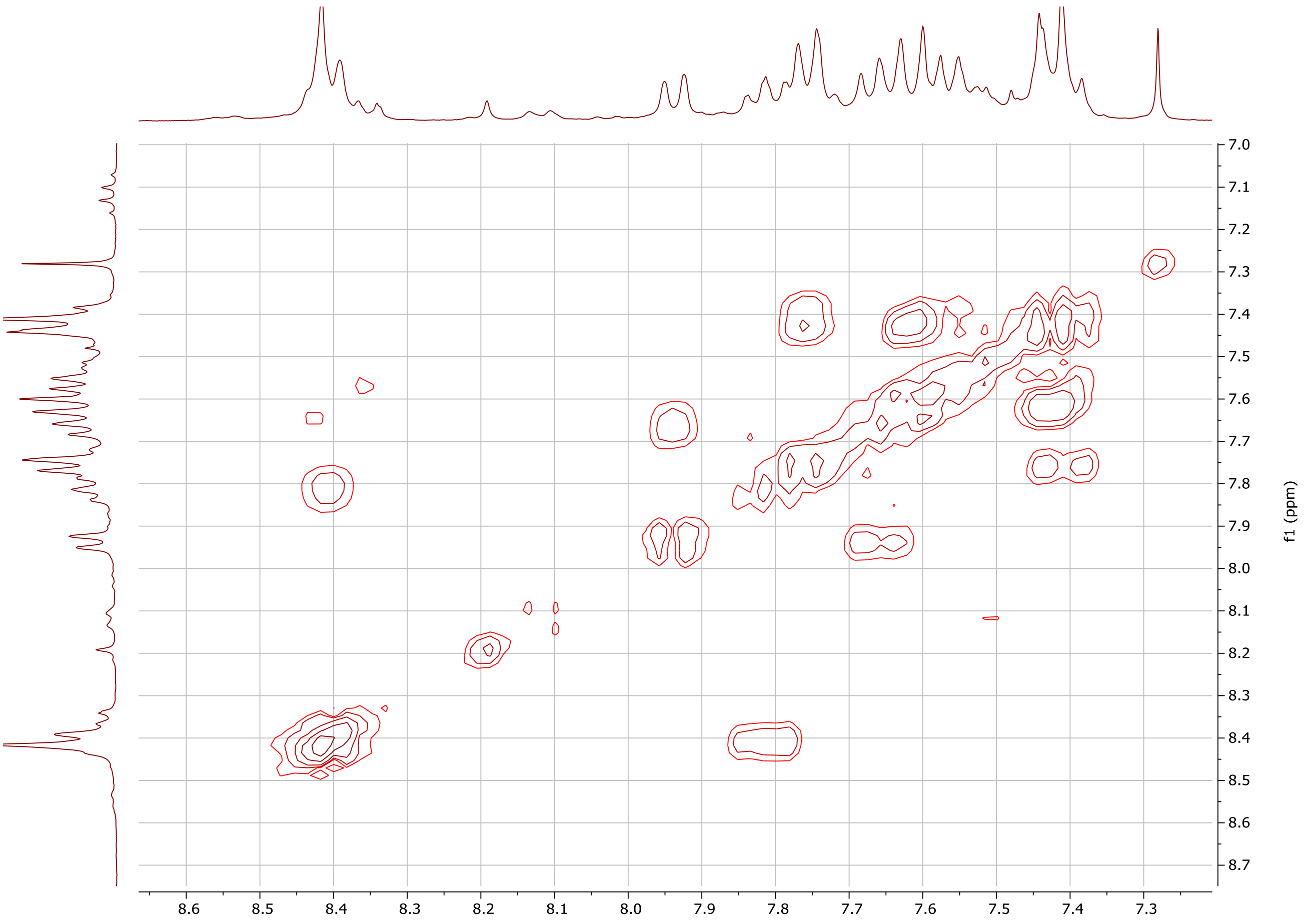

^{1}H – ^{1}H COSY correlation spectrum of **2b** (mixture transoid/cisoid)

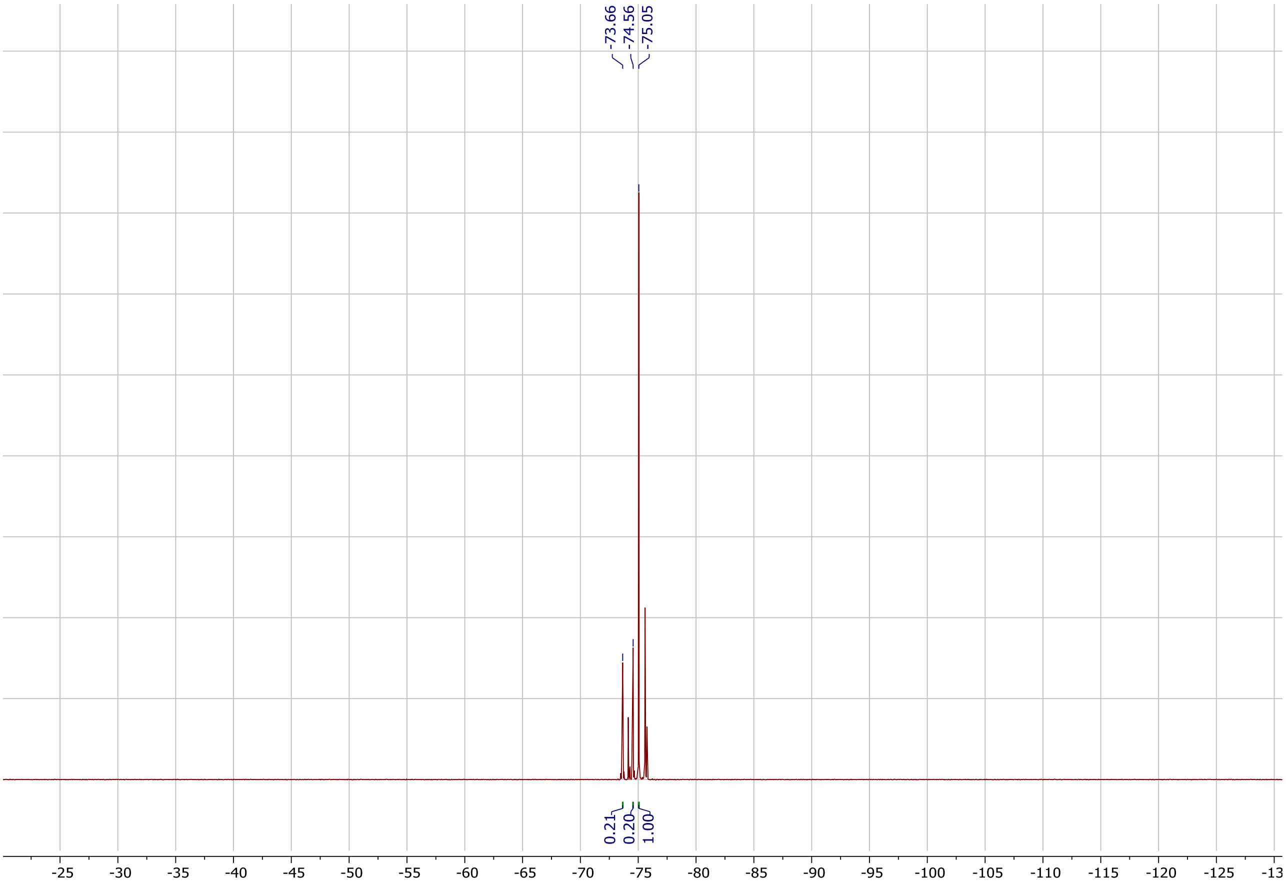

^{19}F-NMR spectrum ($CDCl_3$, 282.40 MHz) of **2b** (mixture transoid/cisoid)

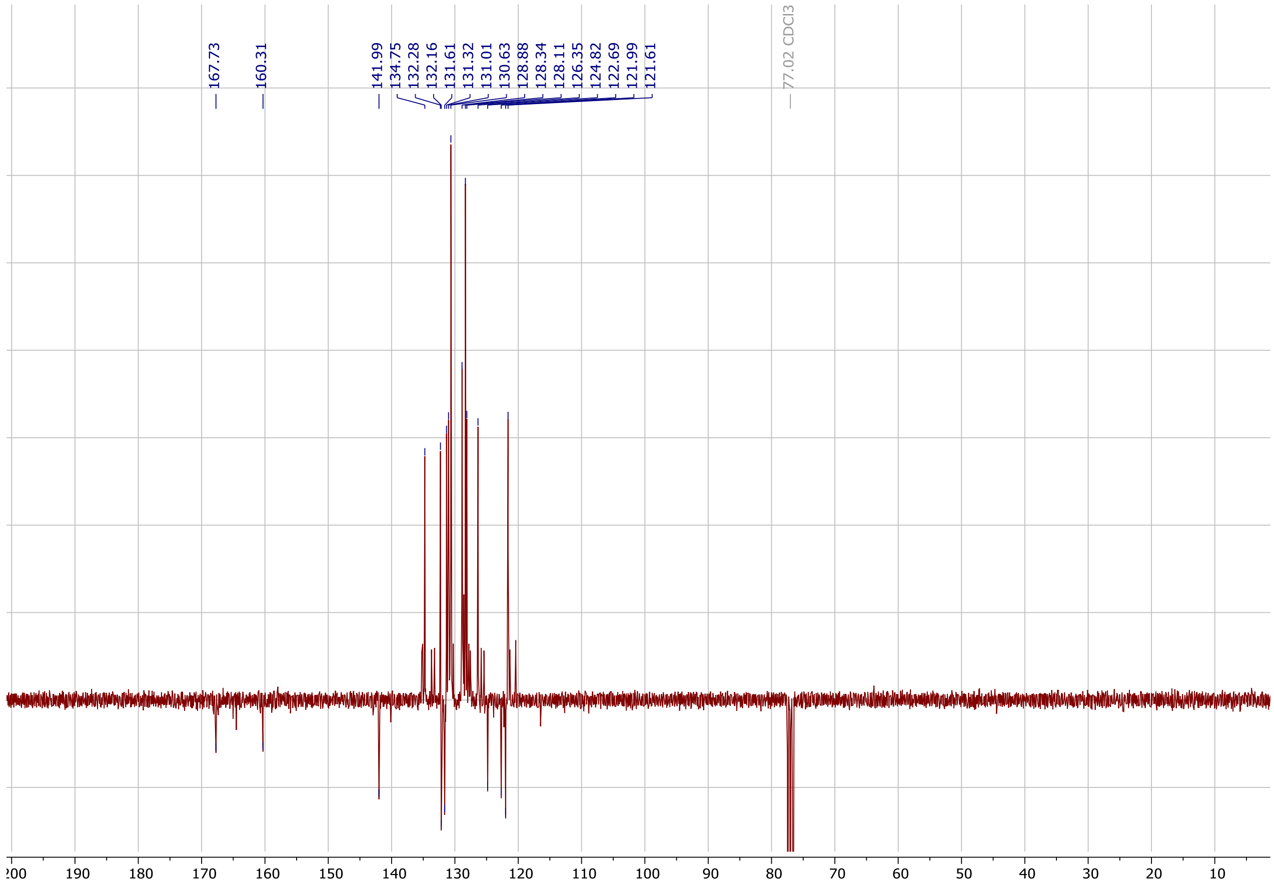

$^{13}C\{^{1}H\}$ (APT) NMR spectrum ($CDCl_3$, 75.47 MHz) of **2b** (mixture transoid/cisoid)

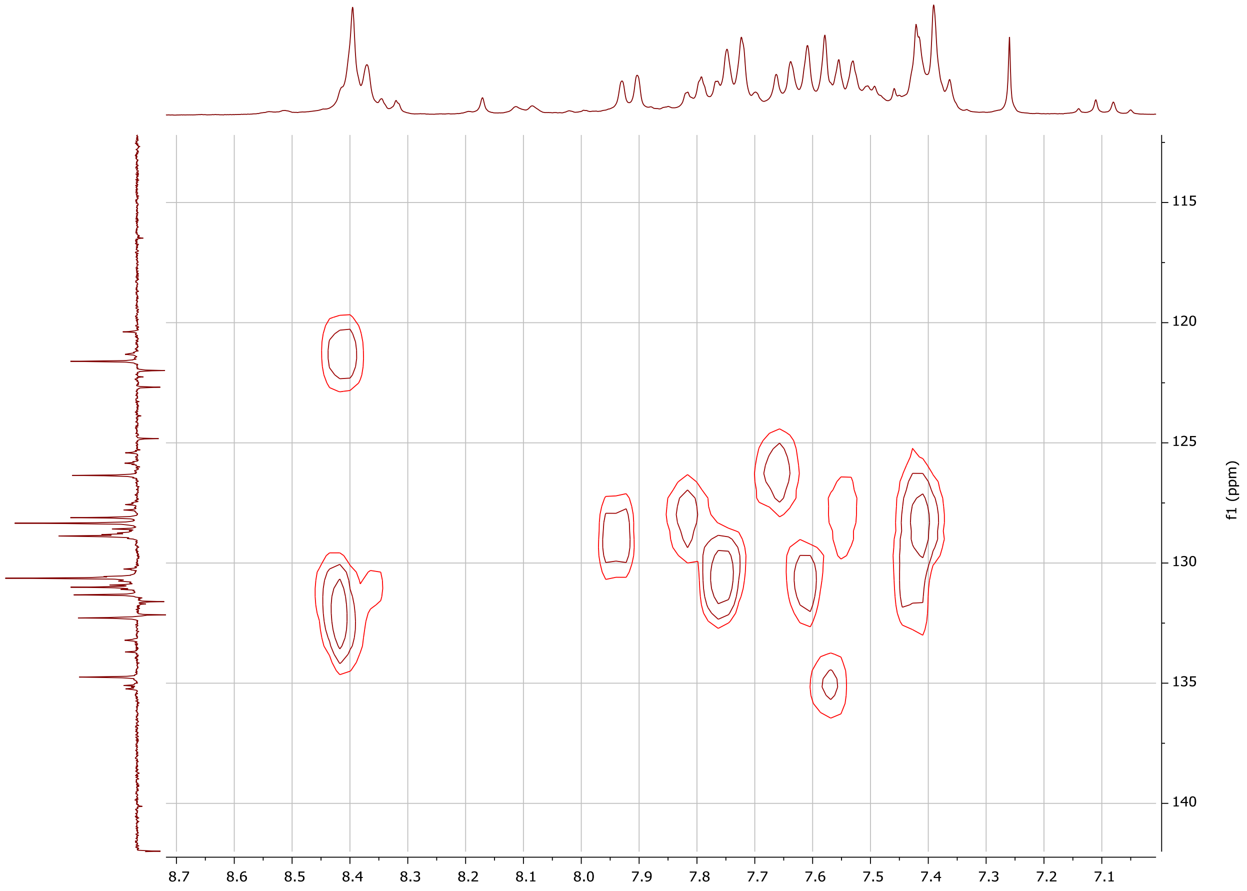

^{1}H - ^{13}C HSQC correlation spectrum of **2b** (mixture transoid/cisoid)

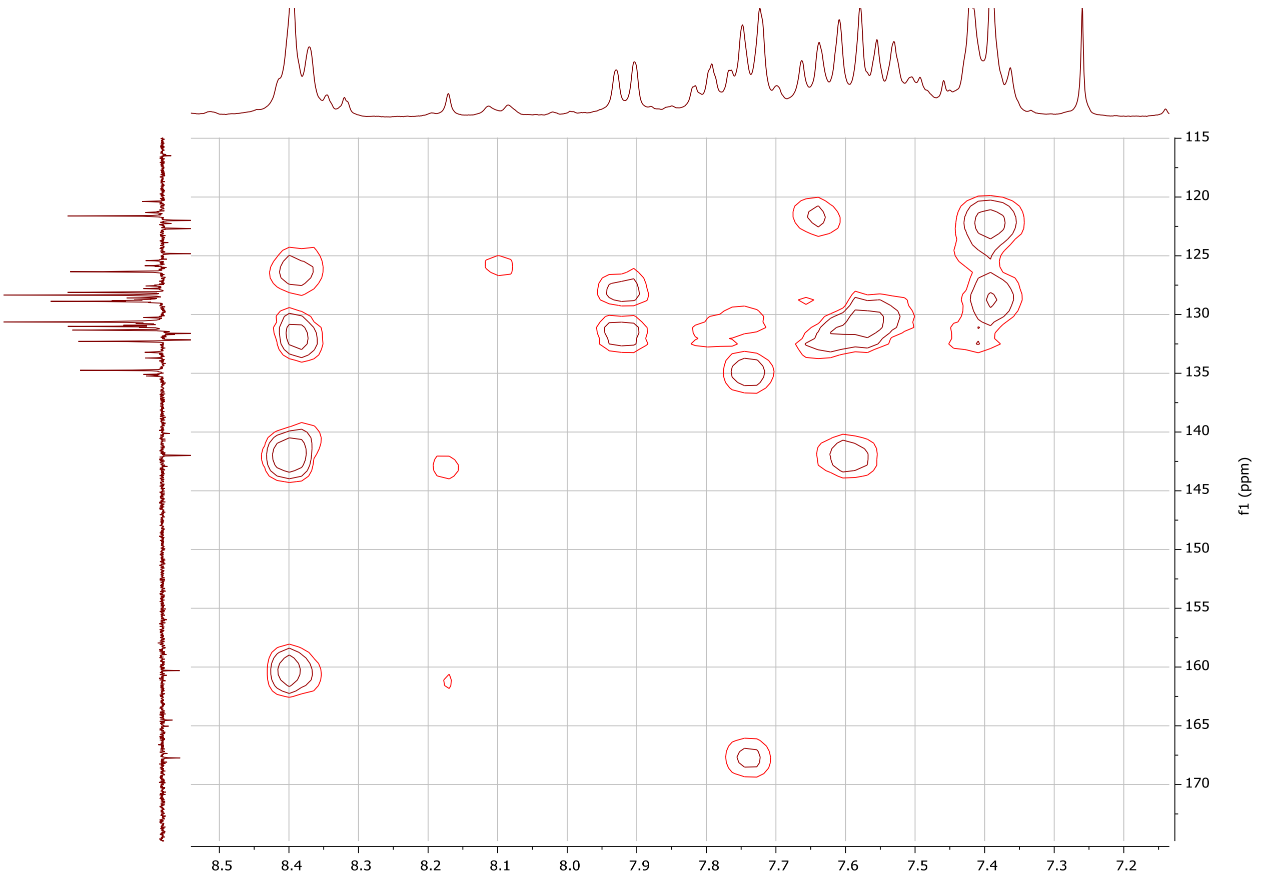

^{1}H - ^{13}C HMBC correlation spectrum of **2b** (mixture transoid/cisoid)

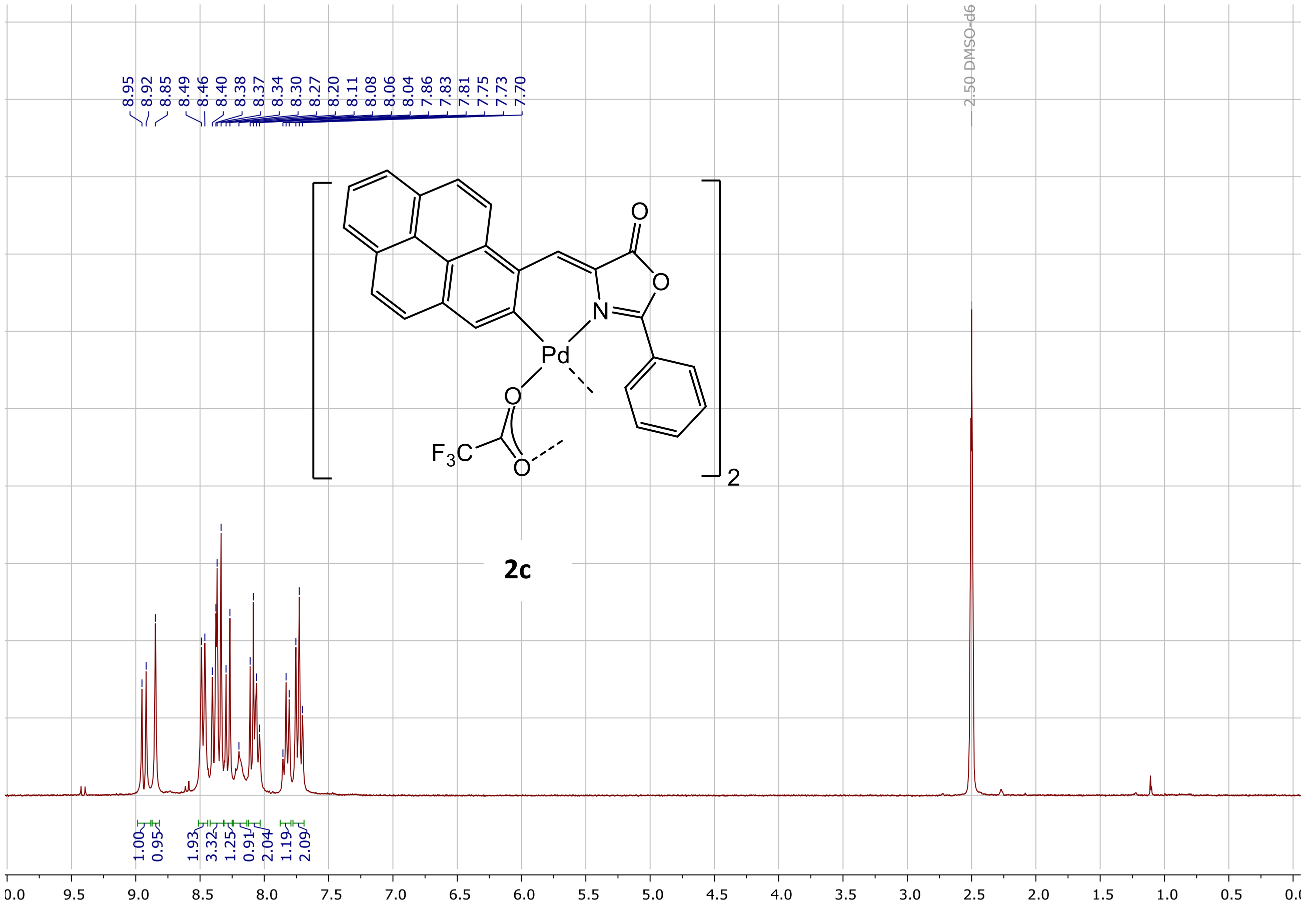

2c

^{1}H-NMR spectrum (DMSO-d_6, 300.13 MHz) of **2c**

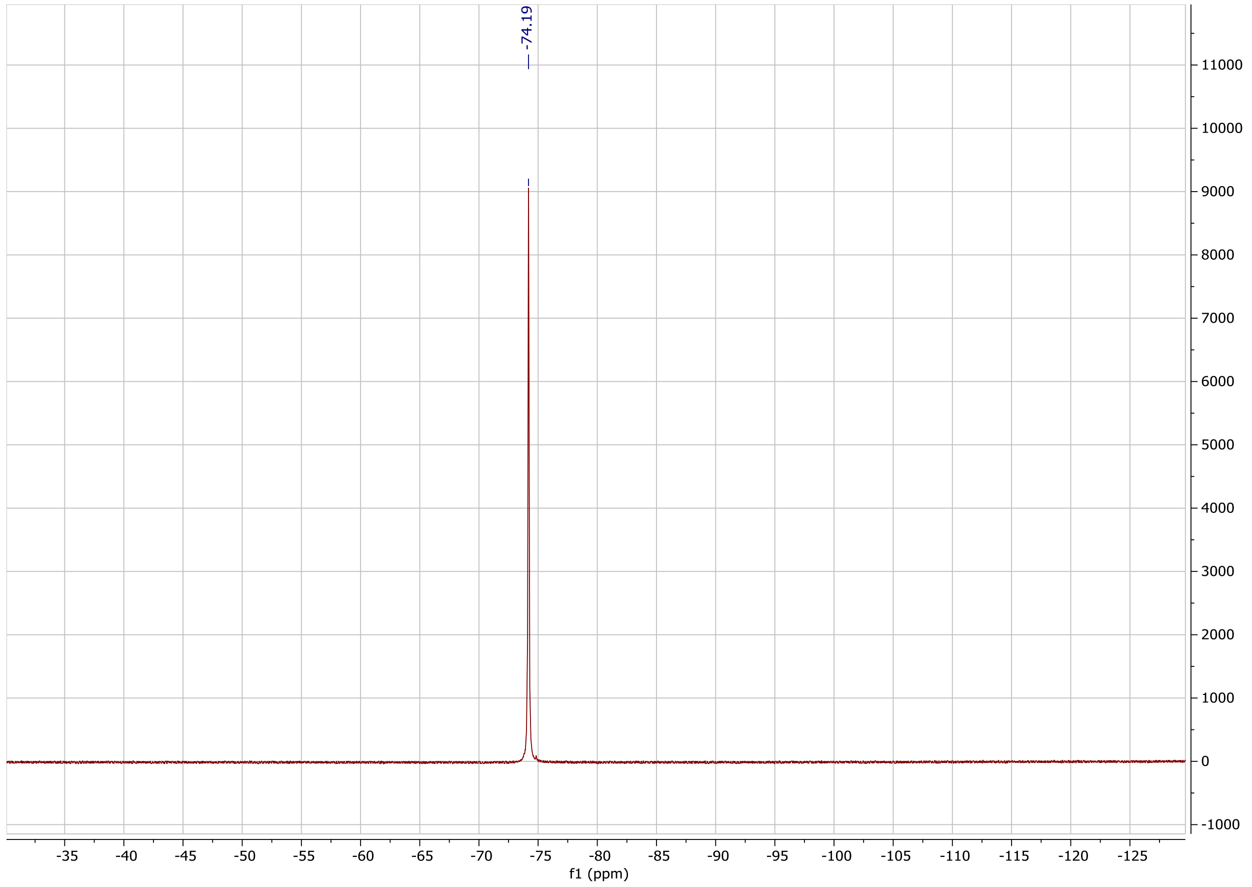

^{19}F-NMR spectrum (DMSO-d$_6$, 282.40 MHz) of **2c**

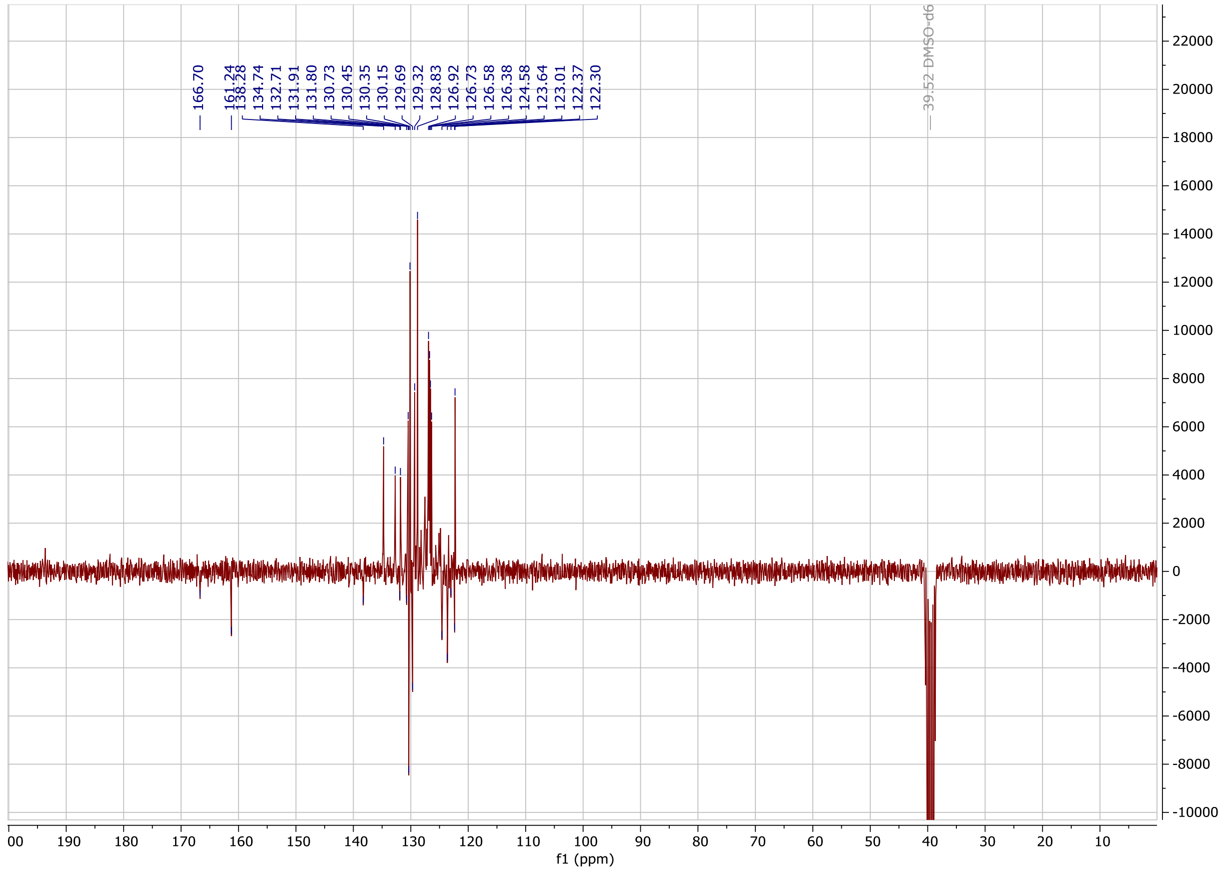

^{13}C{^{1}H} (APT) NMR spectrum (DMSO-d$_6$, 75.47 MHz) of **2c**

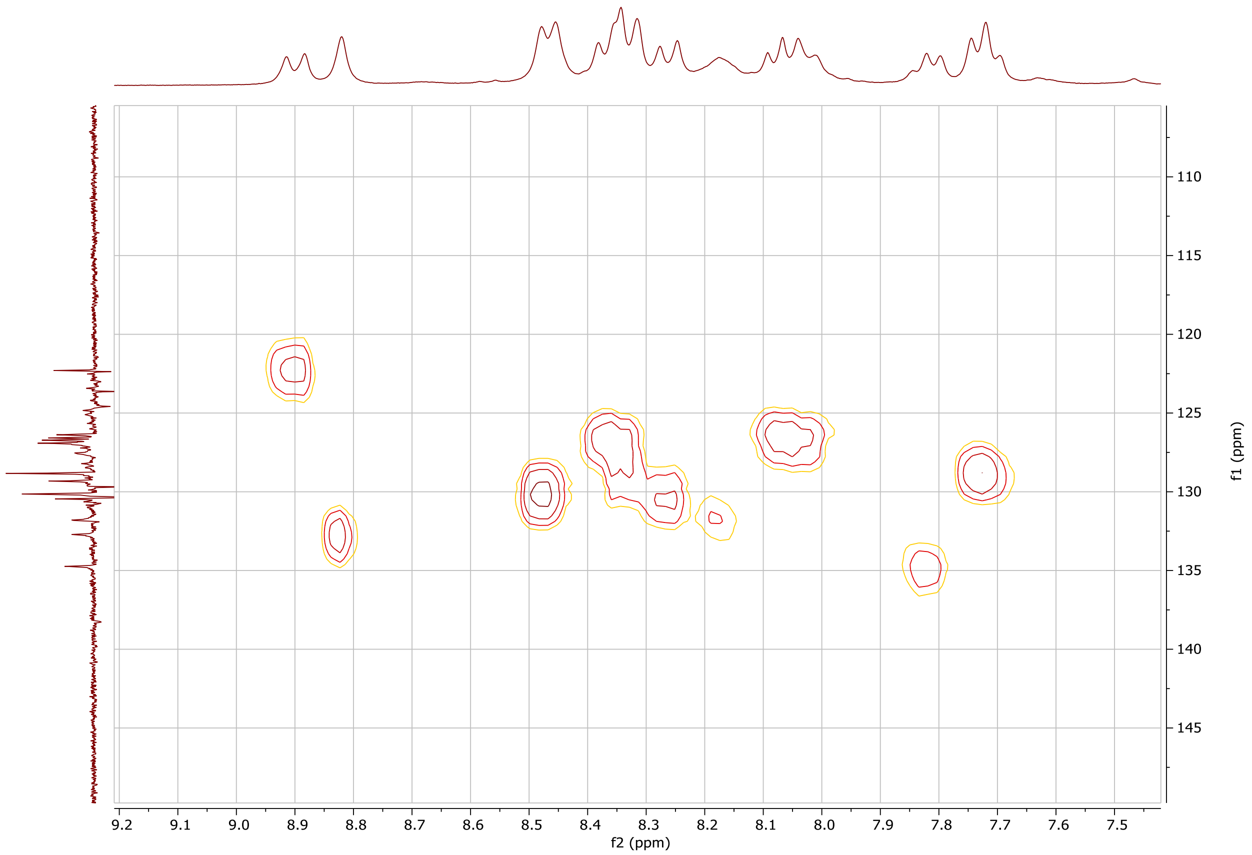

^{1}H - ^{13}C HSQC correlation spectrum of **2c**

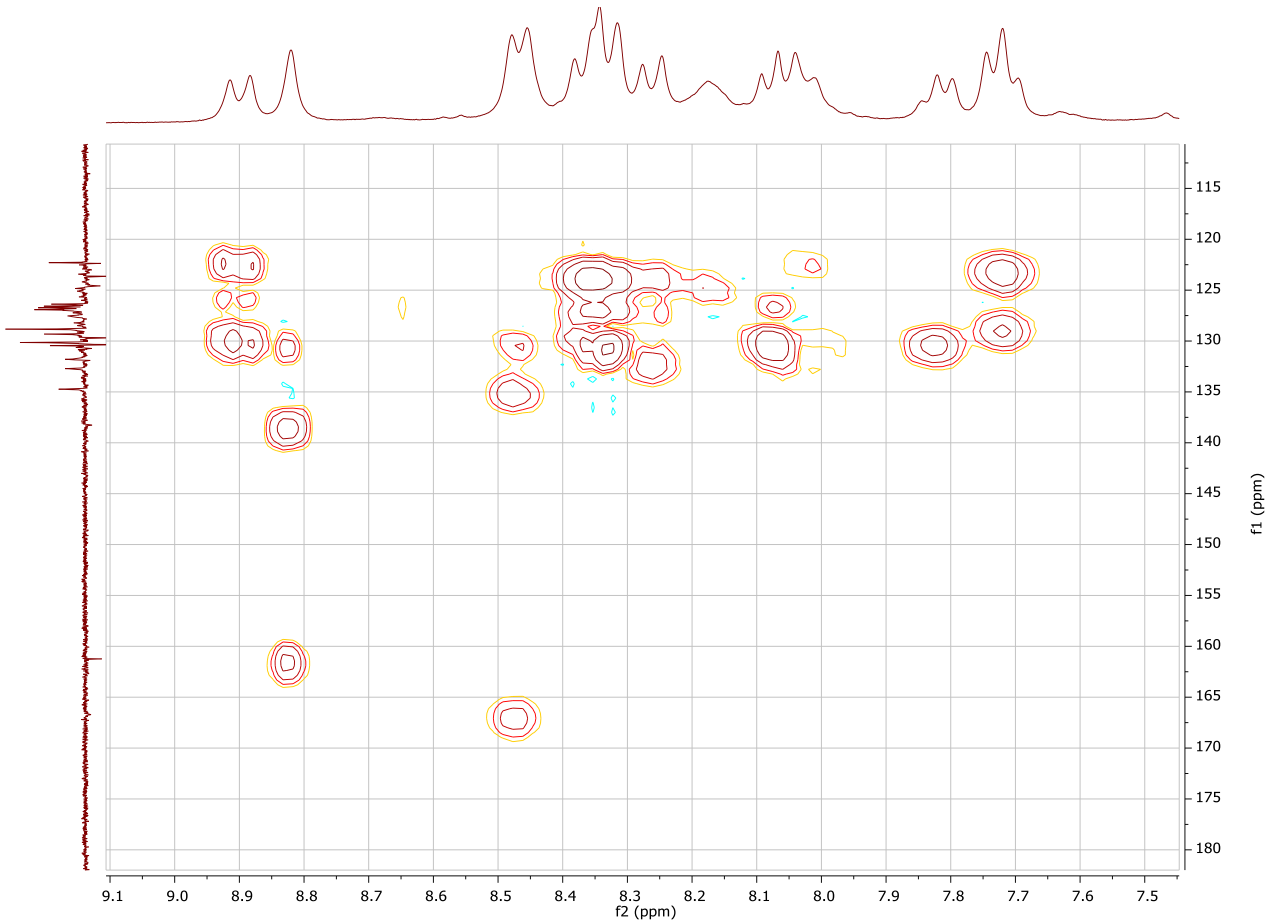

^{1}H - ^{13}C HMBC correlation spectrum of **2c**

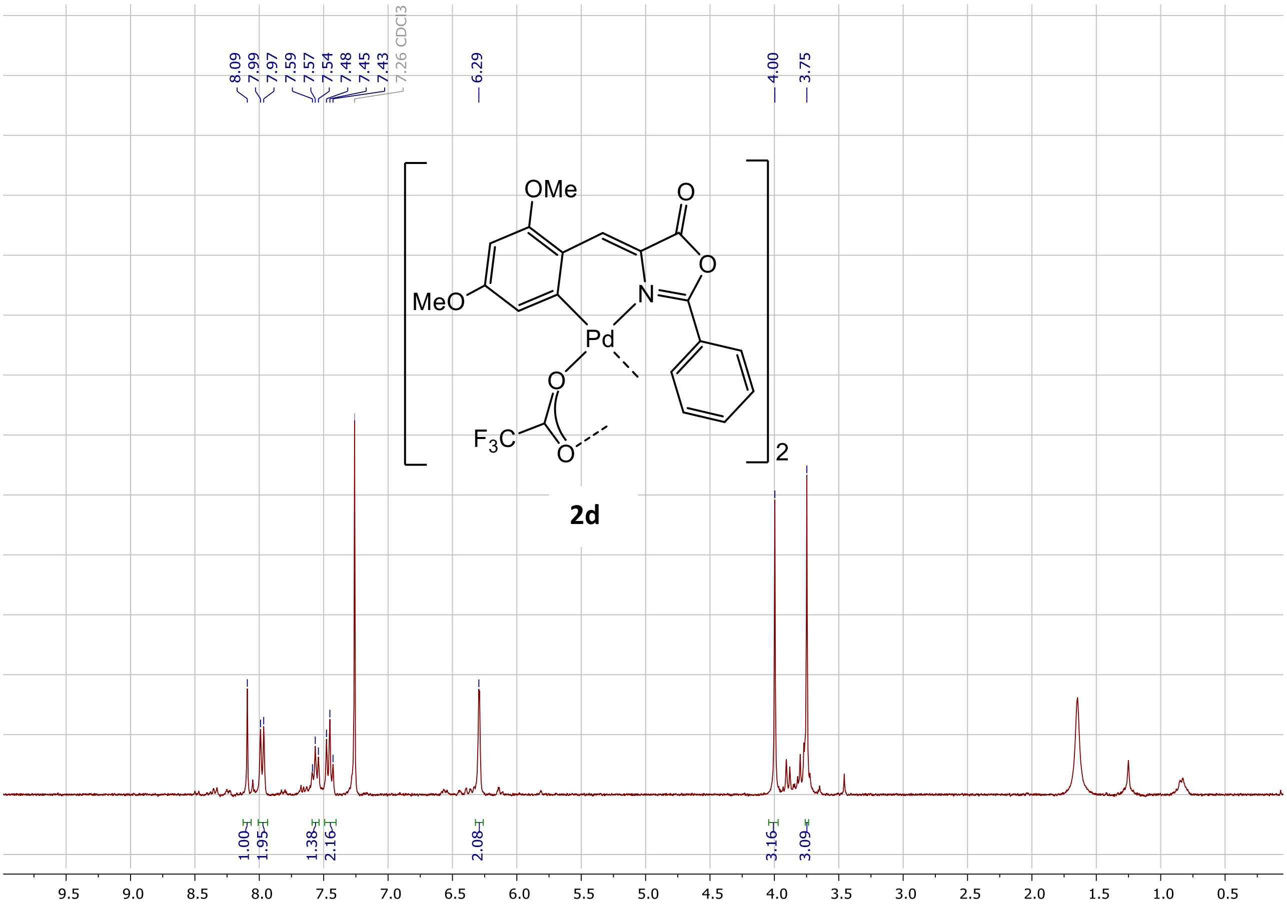

^{1}H-NMR spectrum ($CDCl_3$, 300.13 MHz) of **2d**[19c]

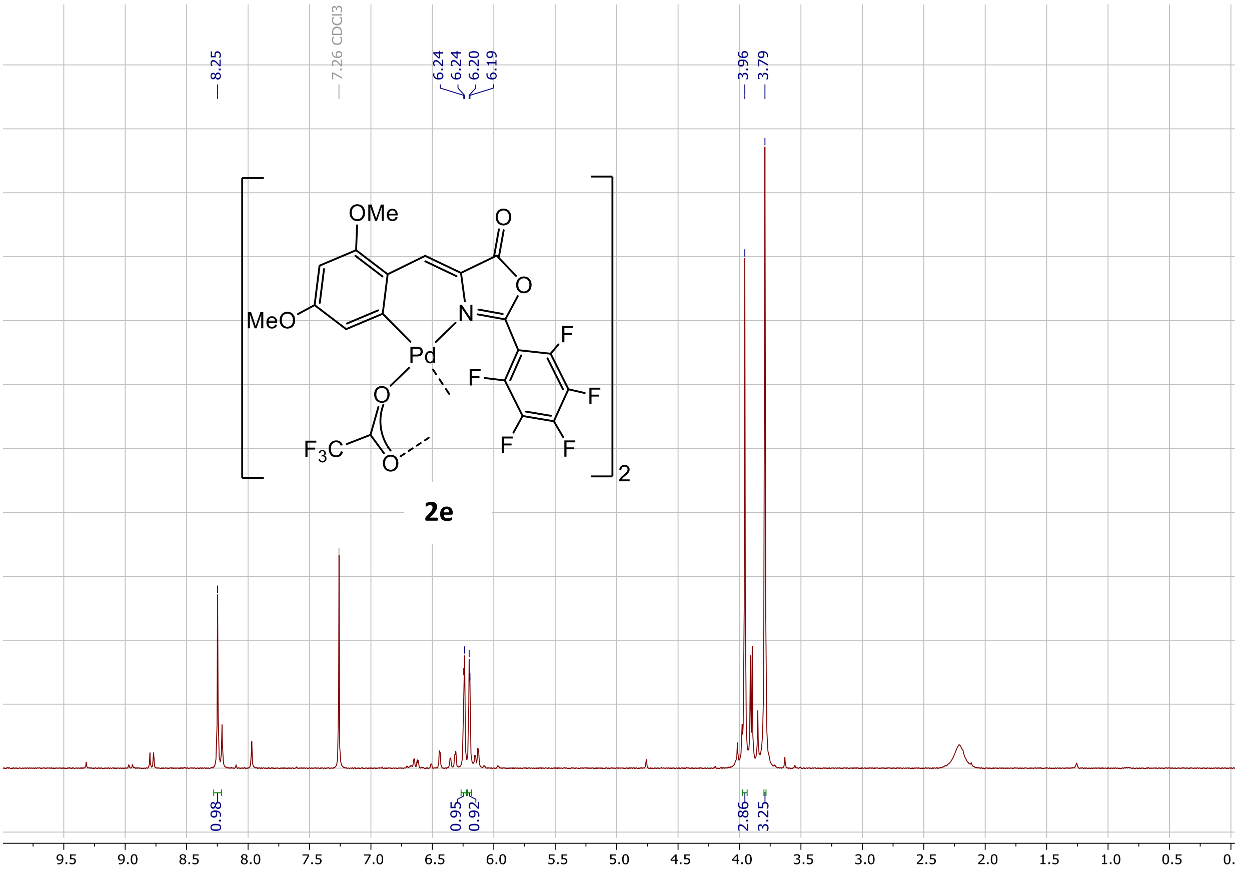

^{1}H-NMR spectrum ($CDCl_3$, 300.13 MHz) of **2e**

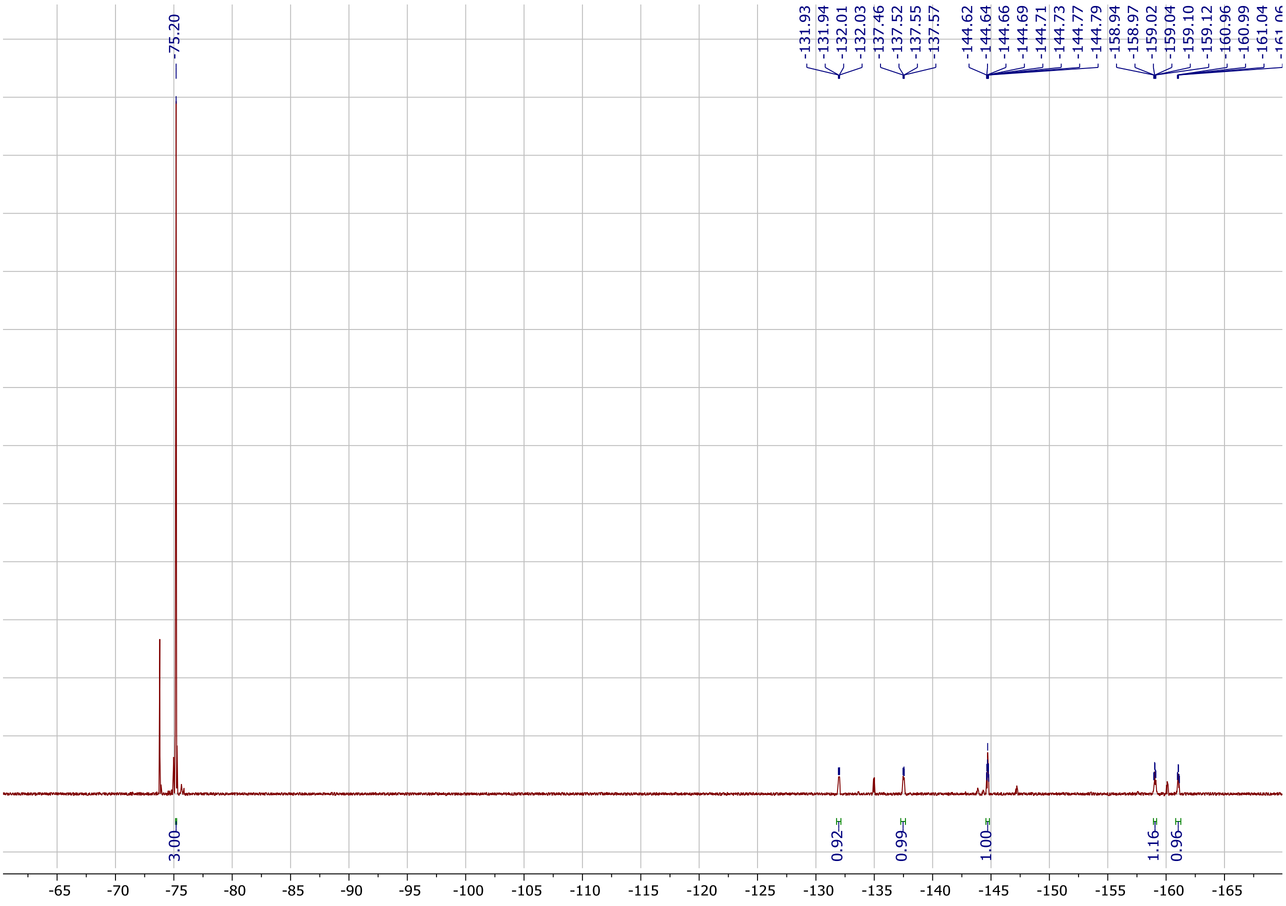

^{19}F-NMR spectrum ($CDCl_3$, 282.40 MHz) of **2e**

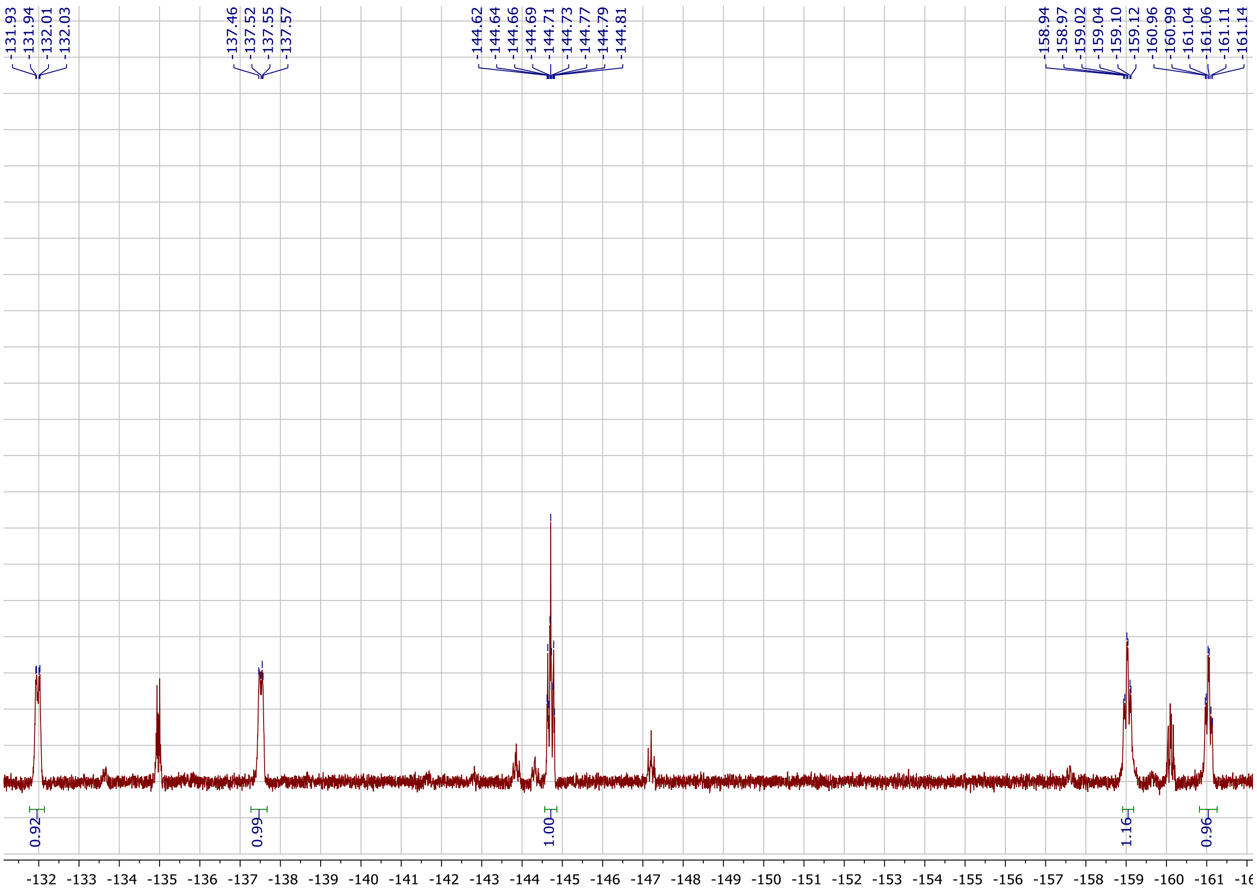

^{19}F-NMR spectrum ($CDCl_3$, 282.40 MHz) of **2e**(zoom C_6F_5 region)

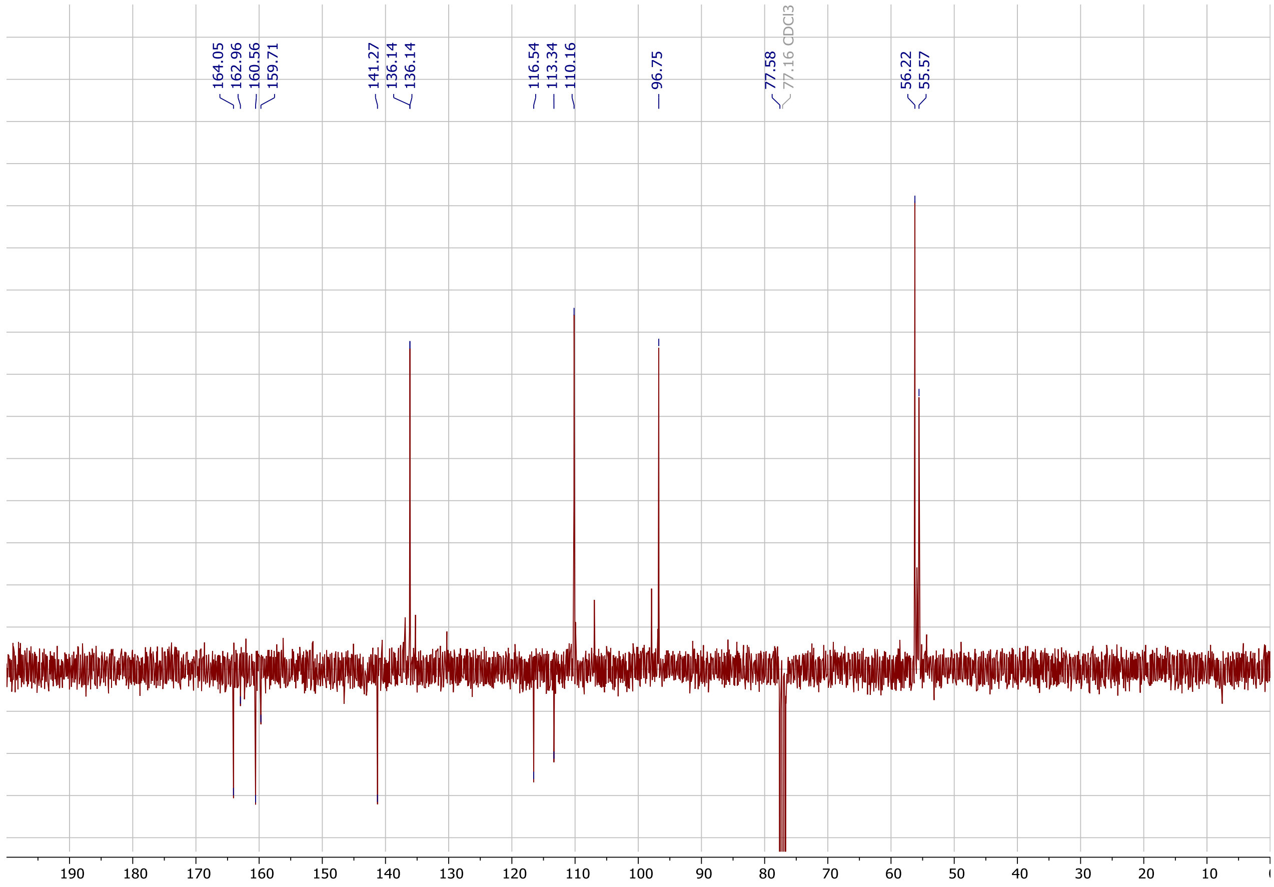

$^{13}C\{^1H\}$ (APT) NMR spectrum ($CDCl_3$, 75.47 MHz) of **2e**

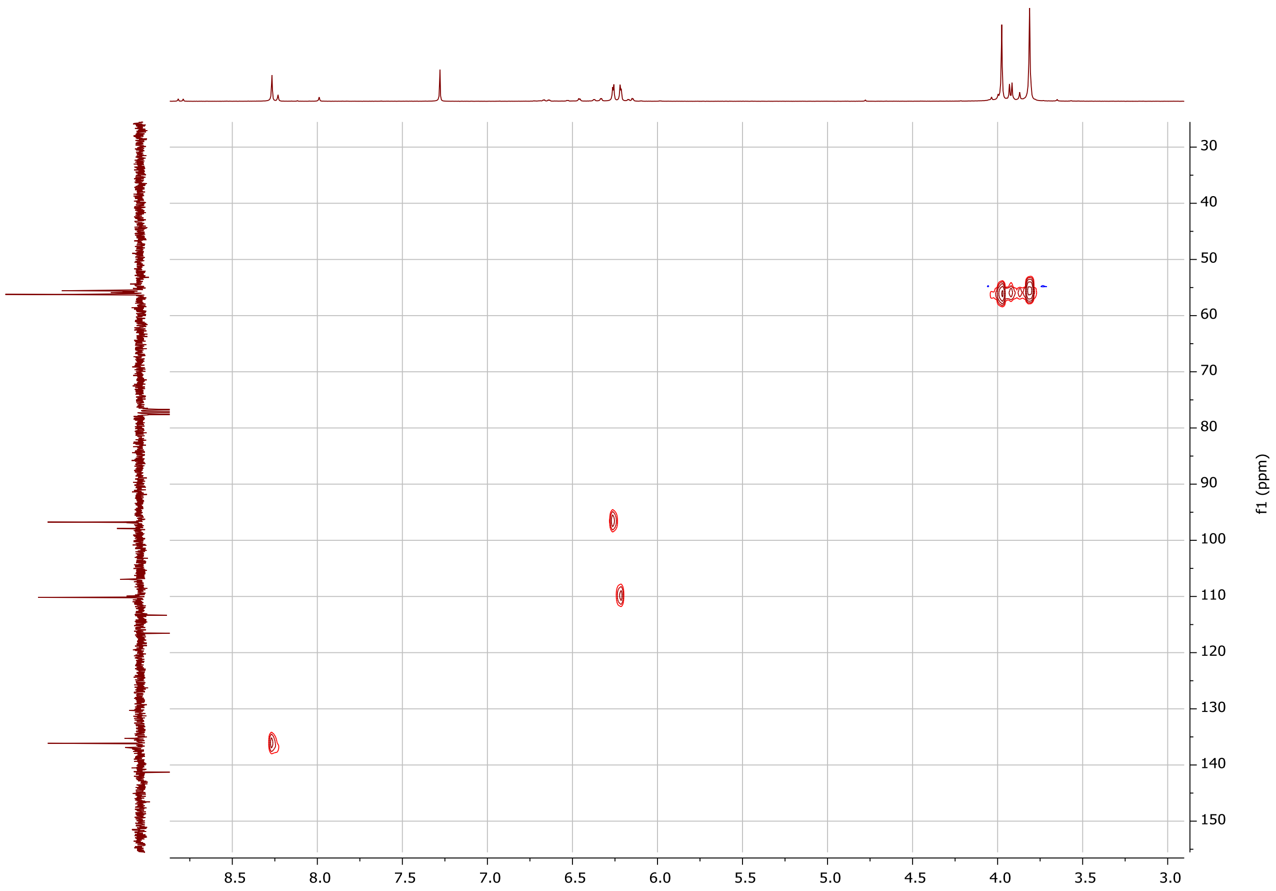

^1H - ^{13}C HSQC correlation spectrum of **2e**

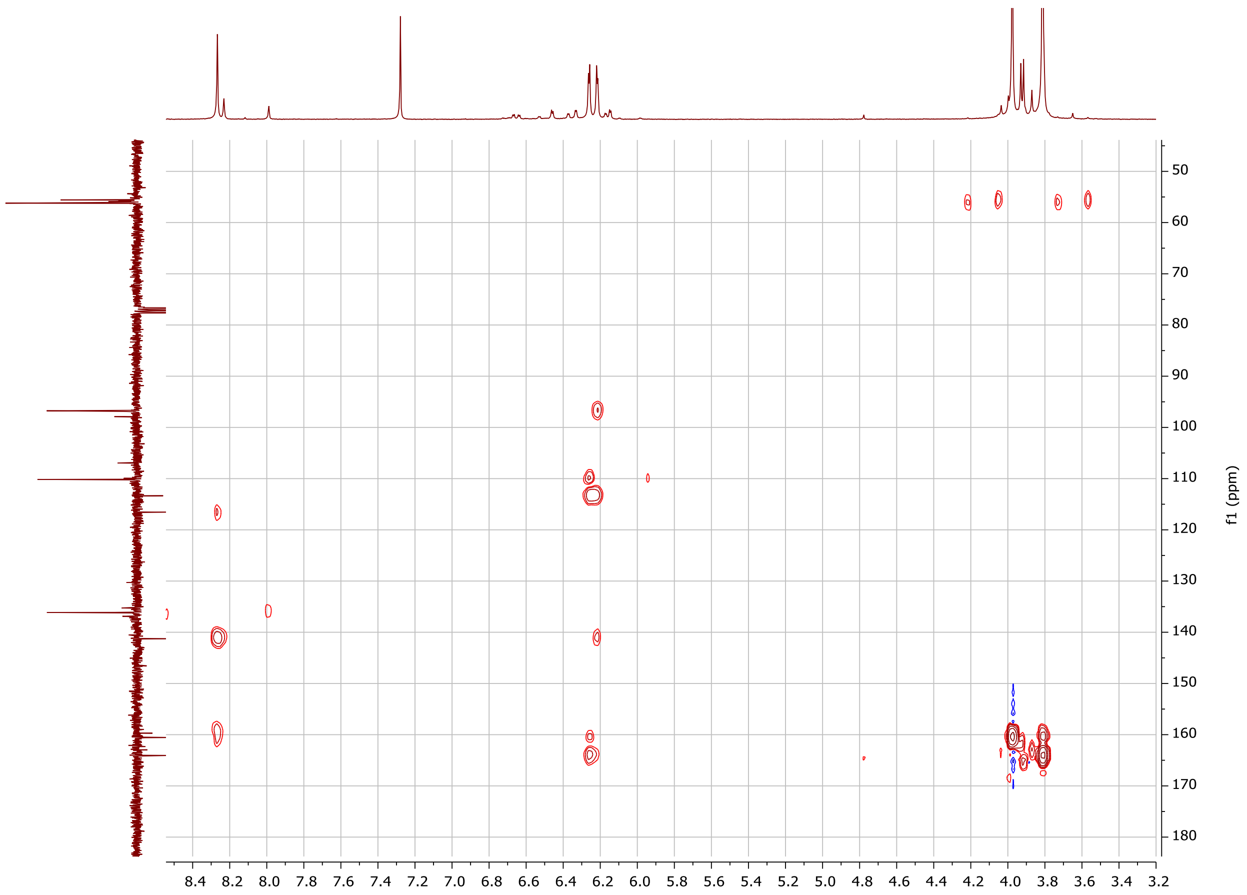

^1H - ^{13}C HMBC correlation spectrum of **2e**

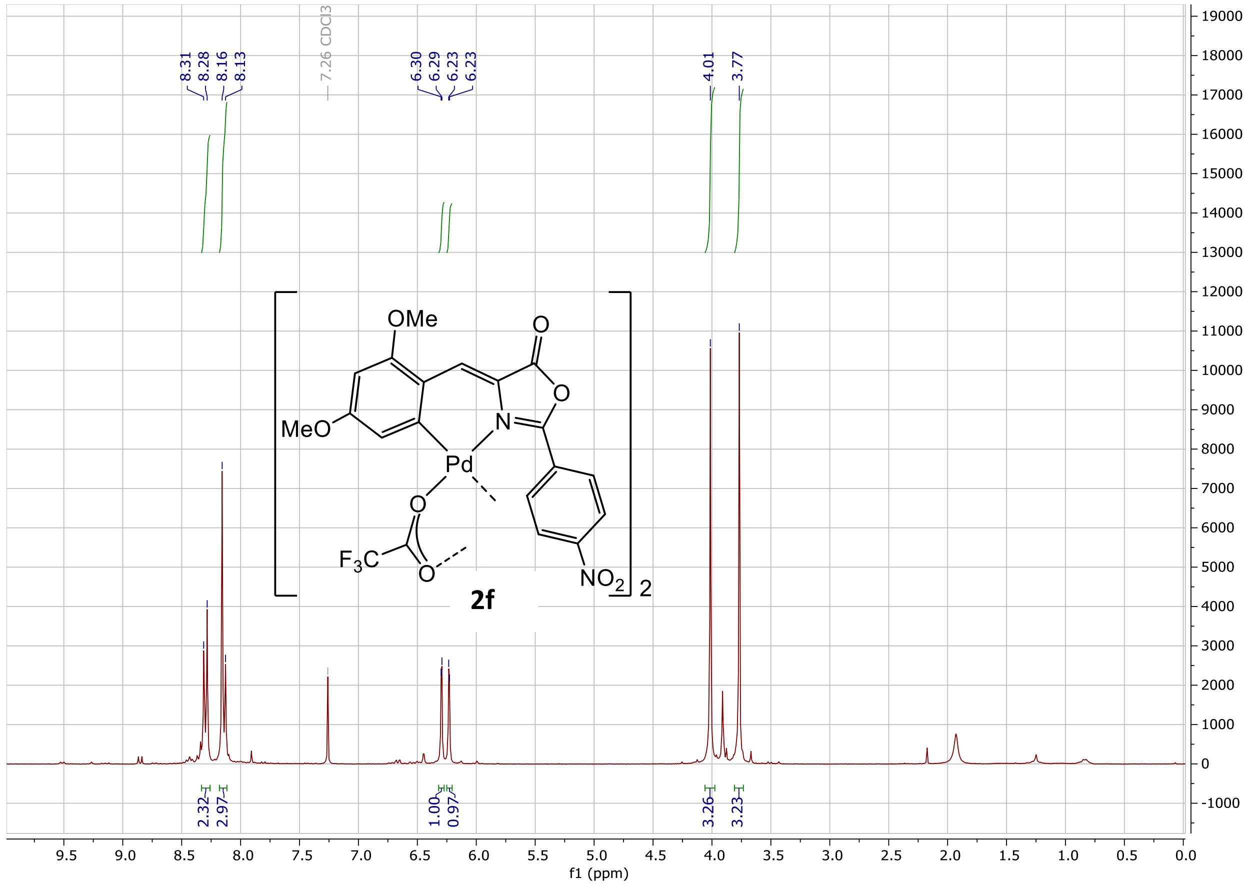

^1H-NMR spectrum ($CDCl_3$, 300.13 MHz) of **2f**

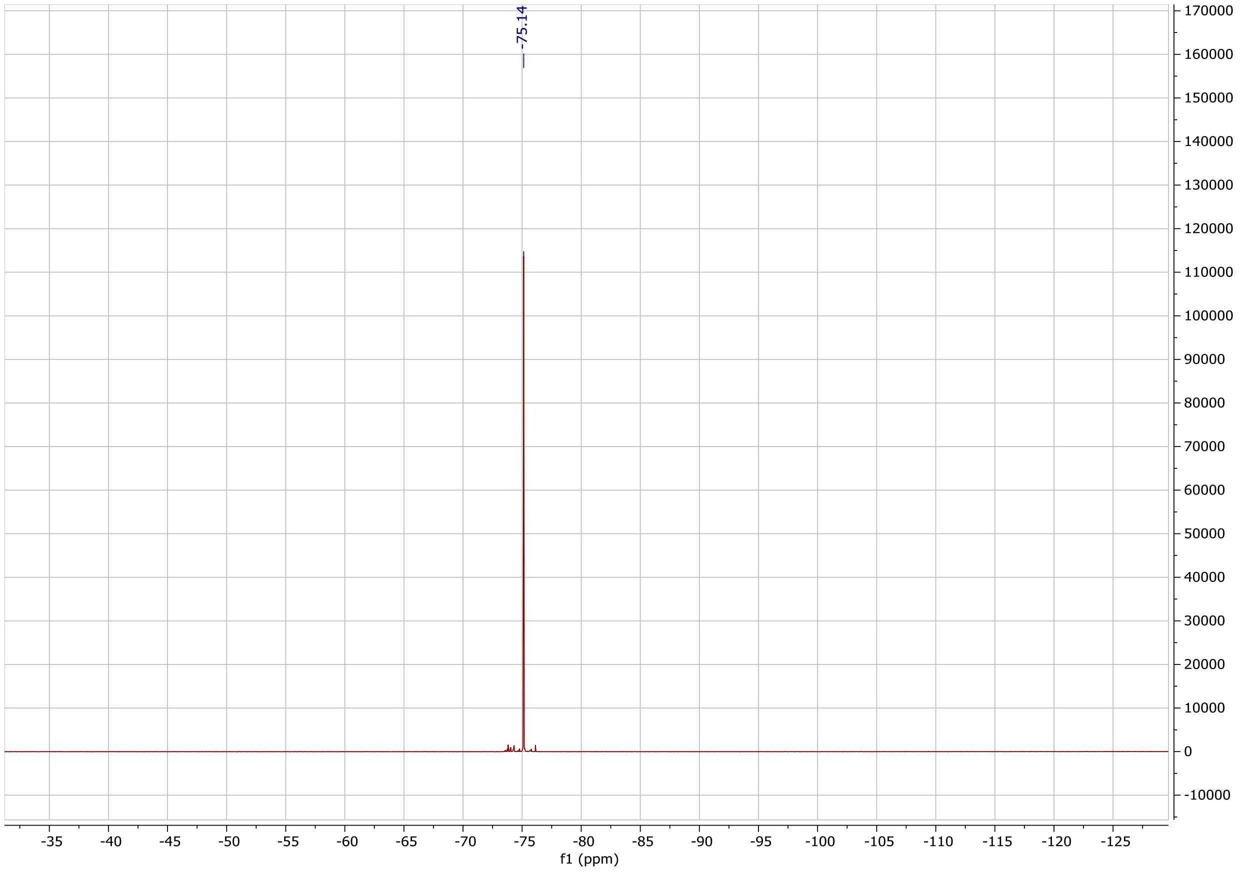

^{19}F-NMR spectrum ($CDCl_3$, 282.40 MHz) of **2f**

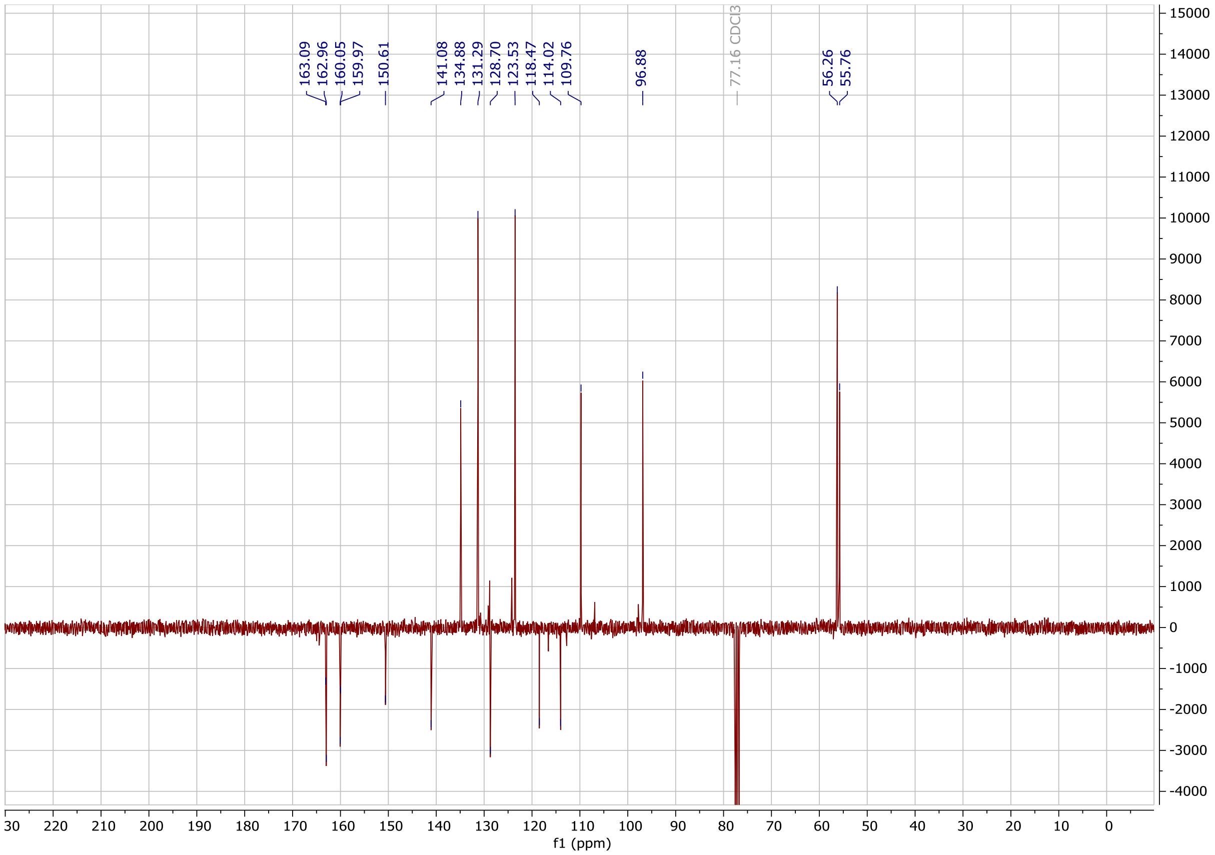

^{13}C{^{1}H} (APT) NMR spectrum ($CDCl_3$, 75.47 MHz) of **2f**

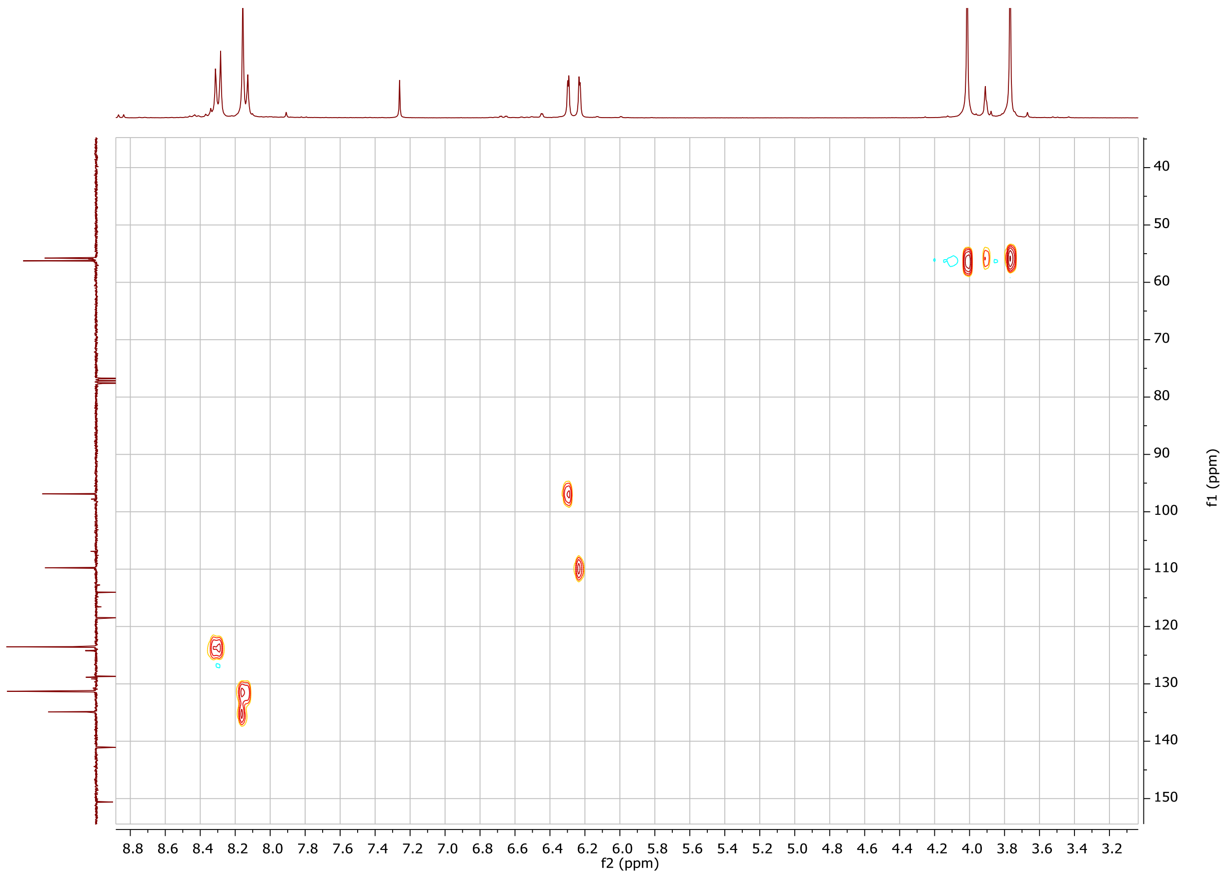

^{1}H - ^{13}C HSQC correlation spectrum of **2f**

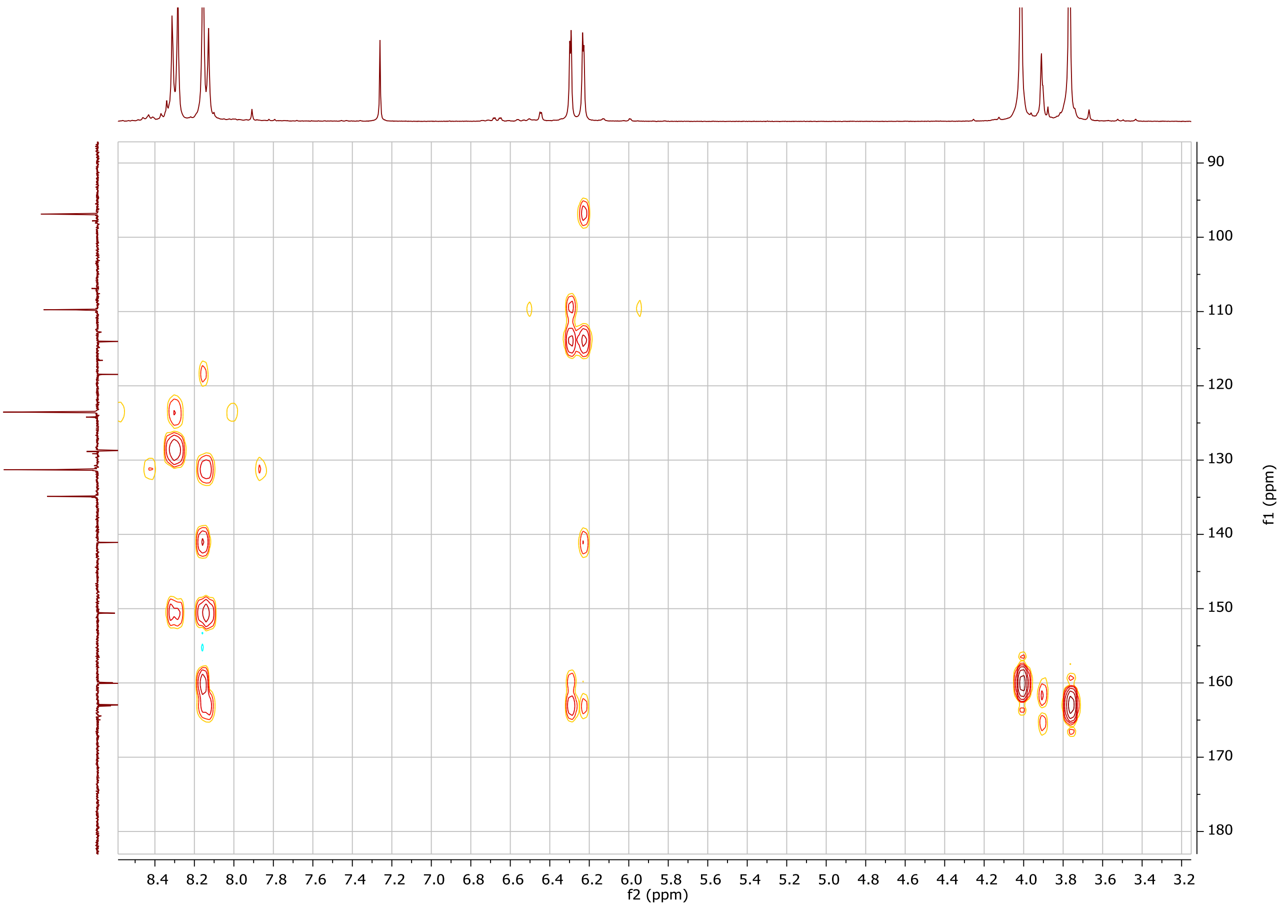

^{1}H - ^{13}C HMBC correlation spectrum of **2f**

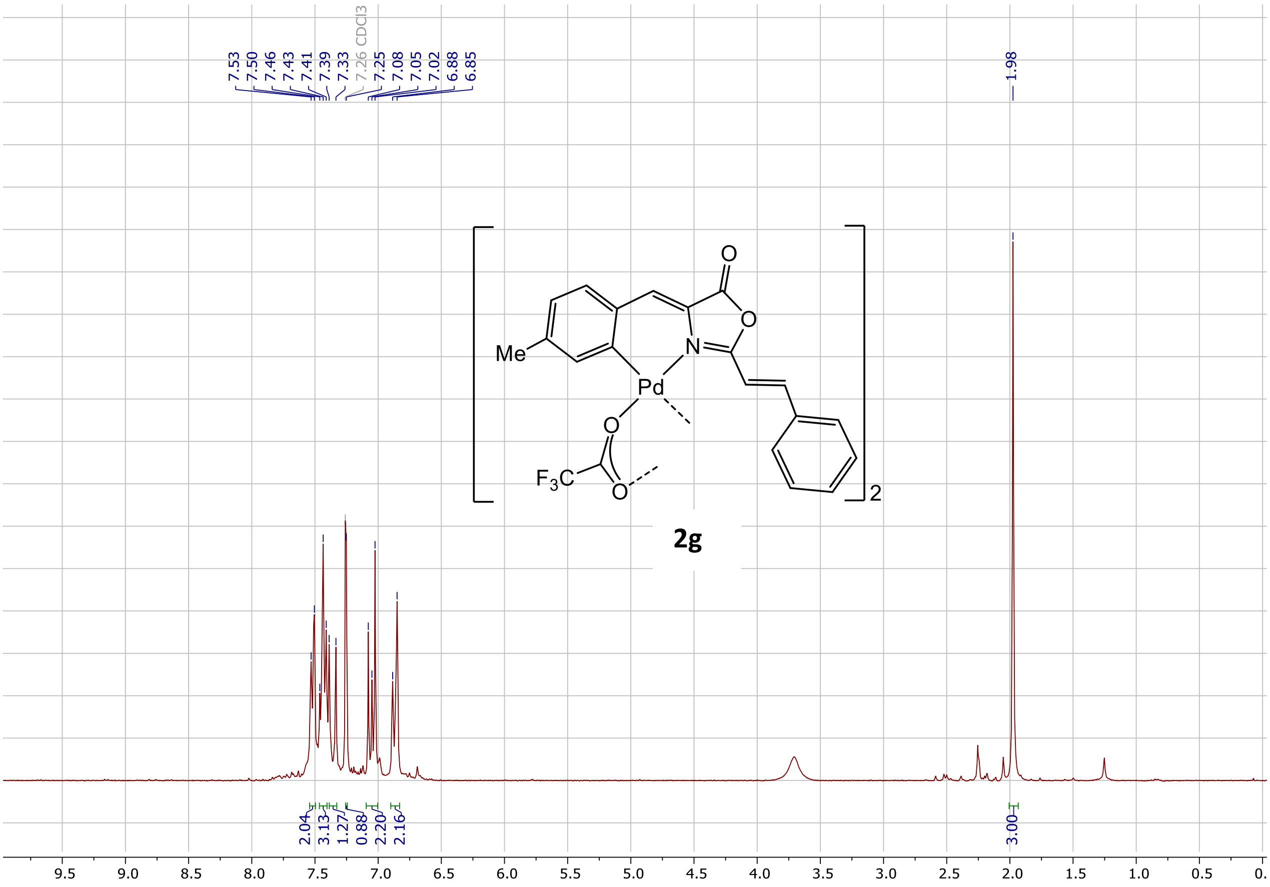

^{1}H-NMR spectrum ($CDCl_3$, 300.13 MHz) of **2g**

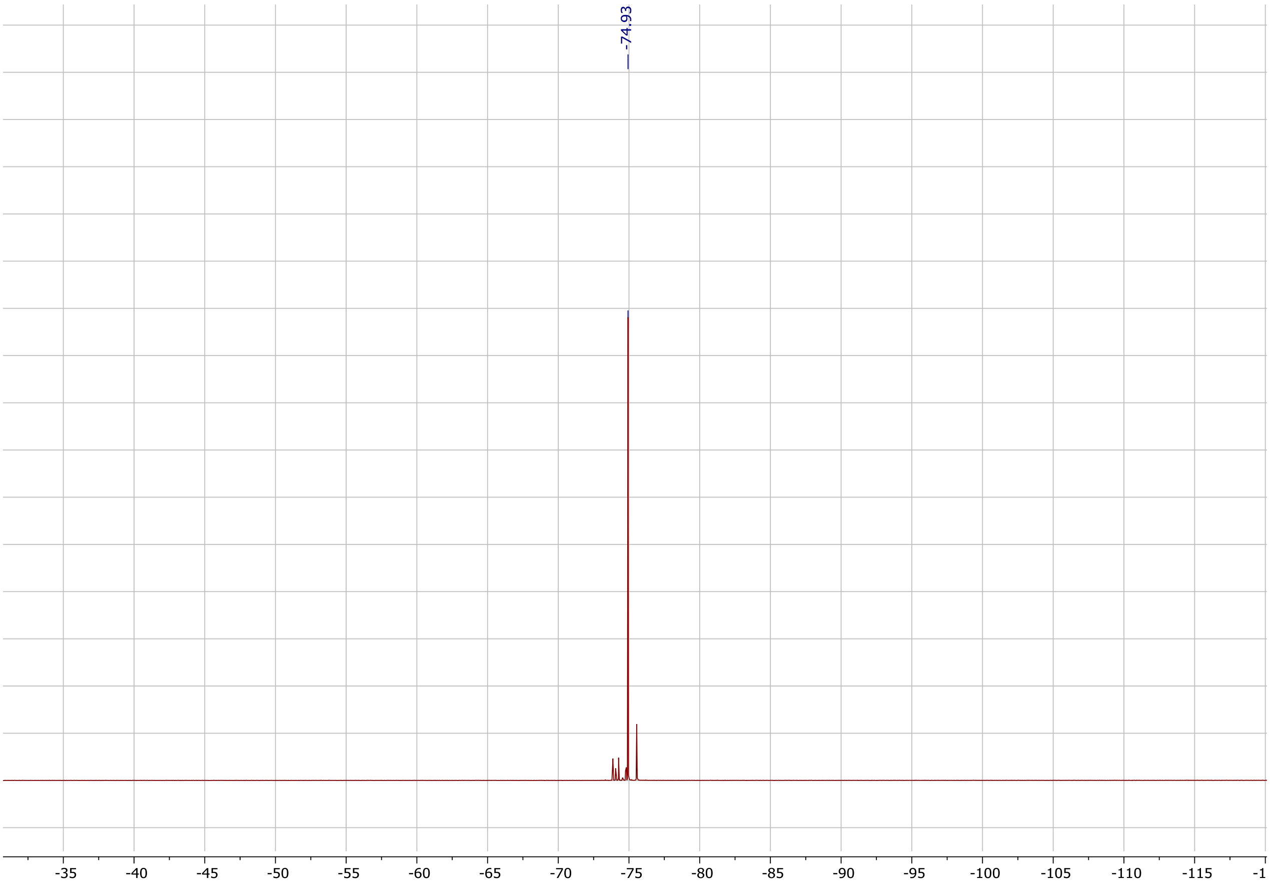

^{19}F-NMR spectrum ($CDCl_3$, 282.40 MHz) of **2g**

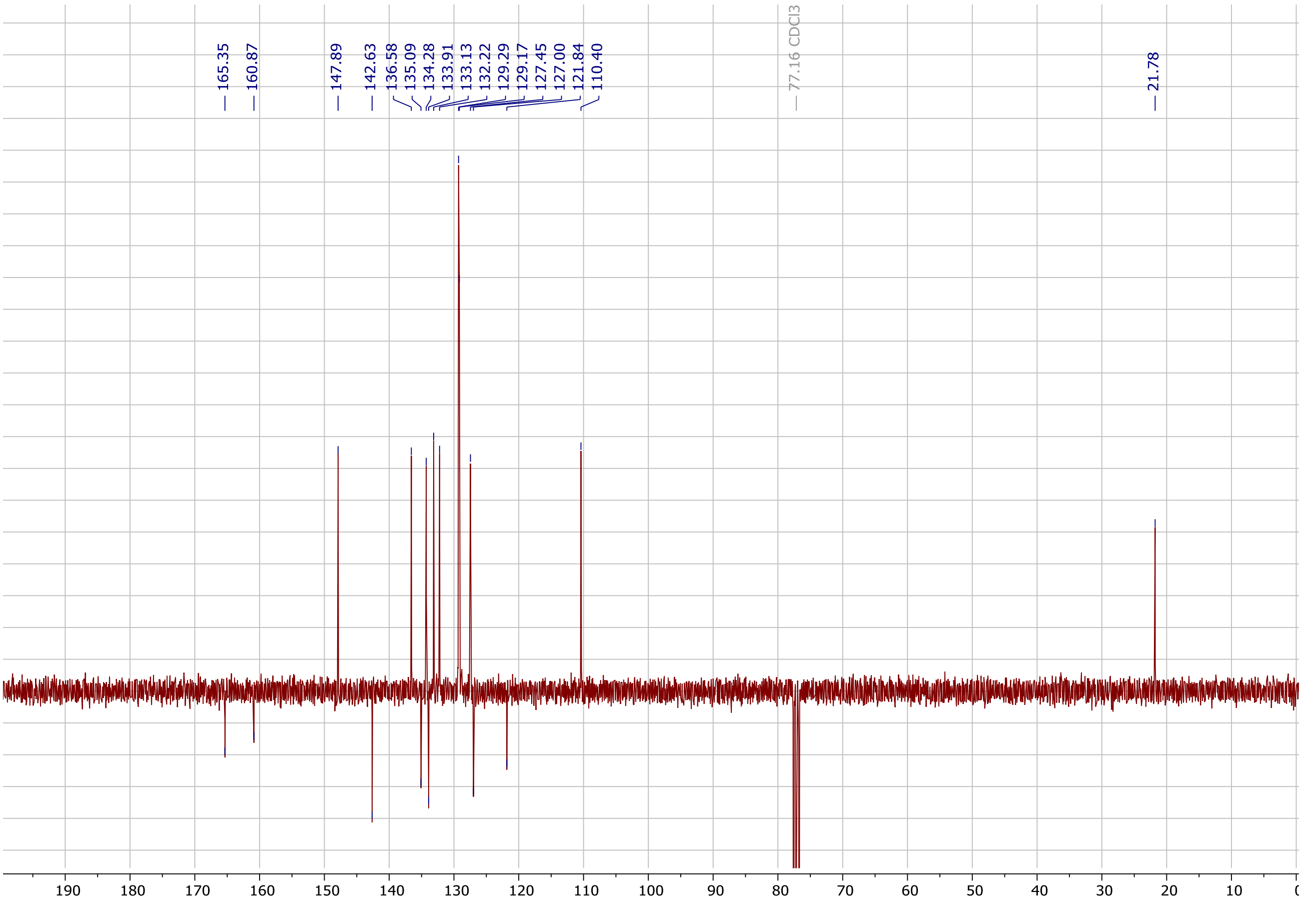

$^{13}C\{^1H\}$ (APT) NMR spectrum ($CDCl_3$, 75.47 MHz) of **2g**

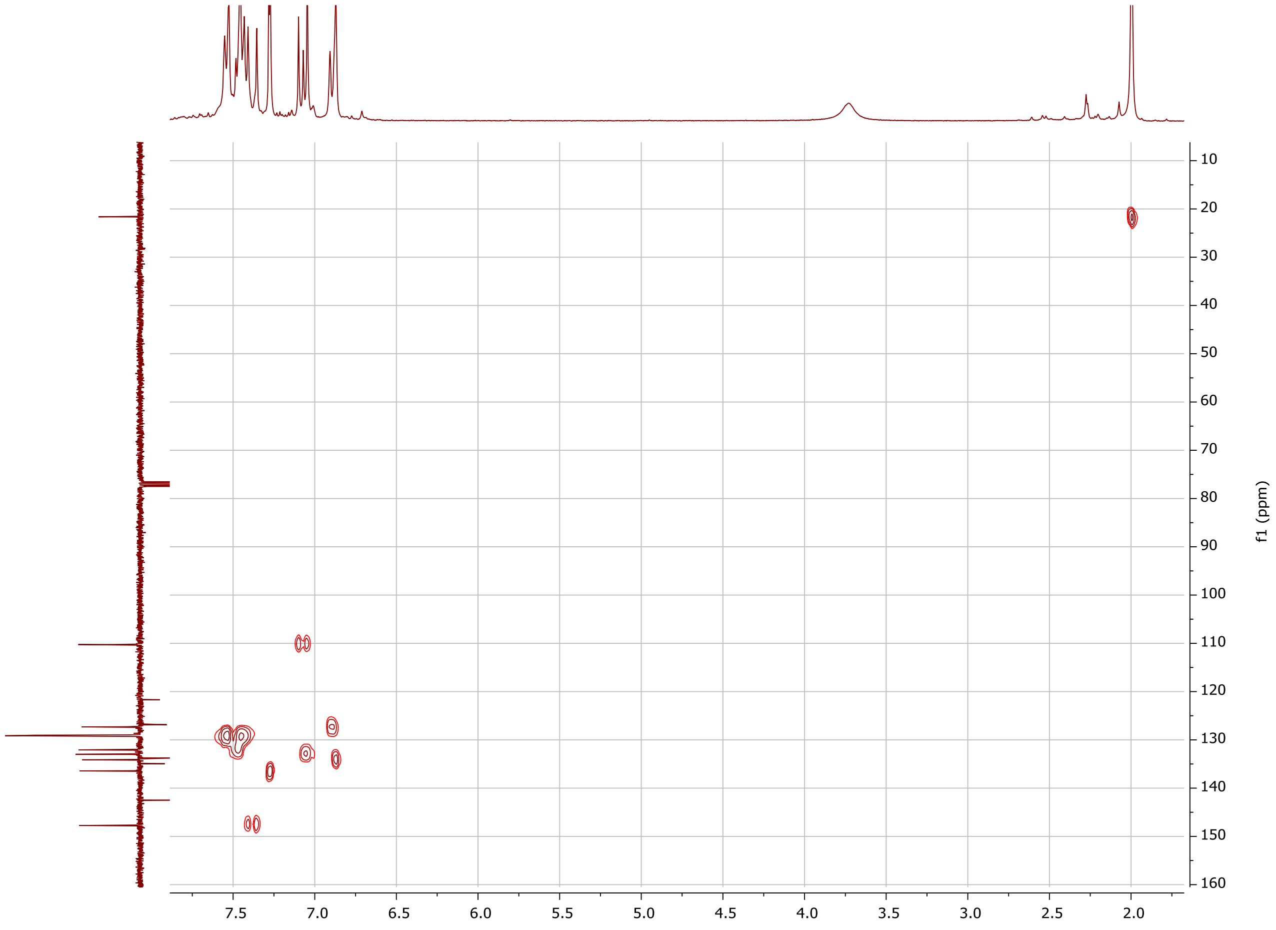

^1H - ^{13}C HSQC correlation spectrum of **2g**

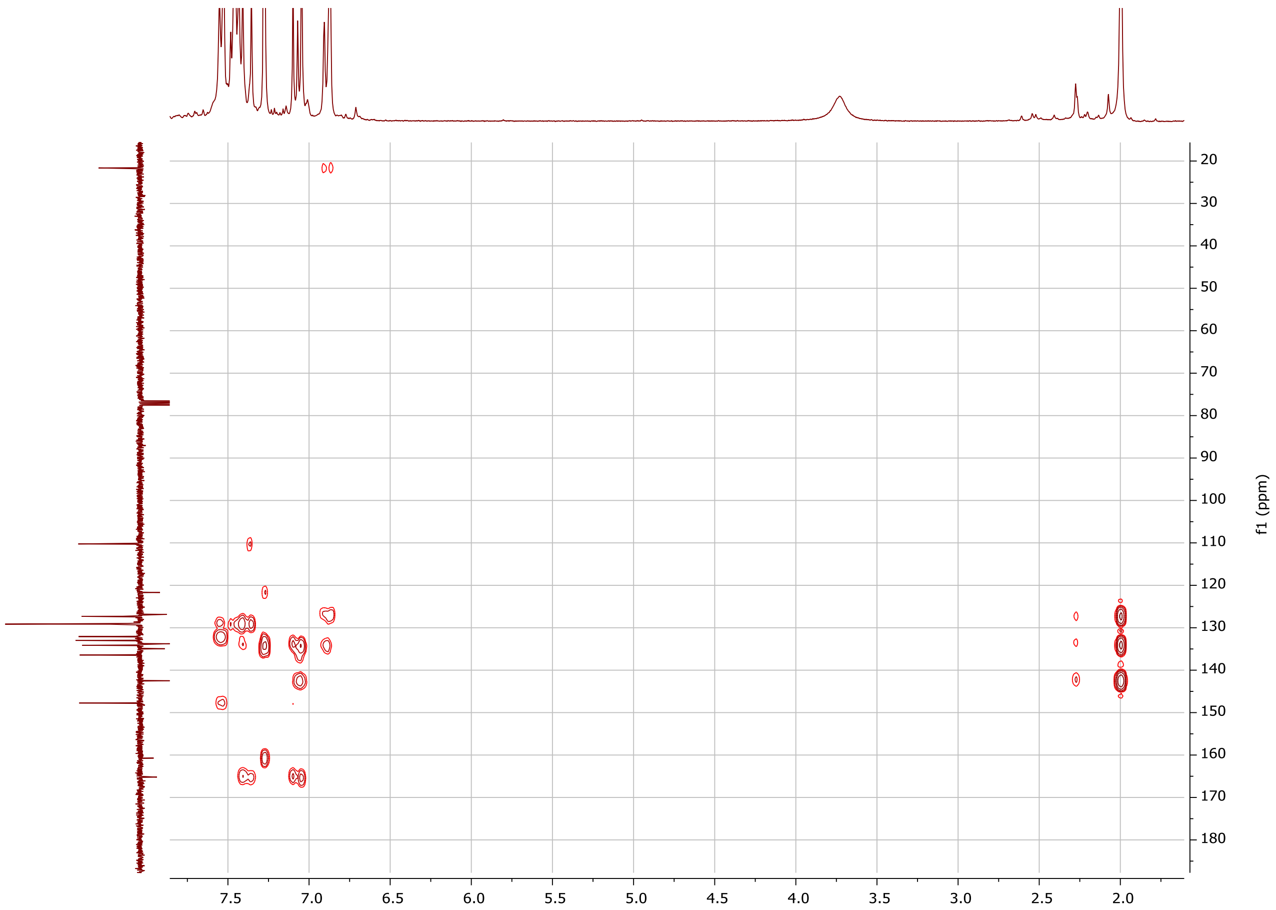

^1H - ^{13}C HMBC correlation spectrum of **2g**

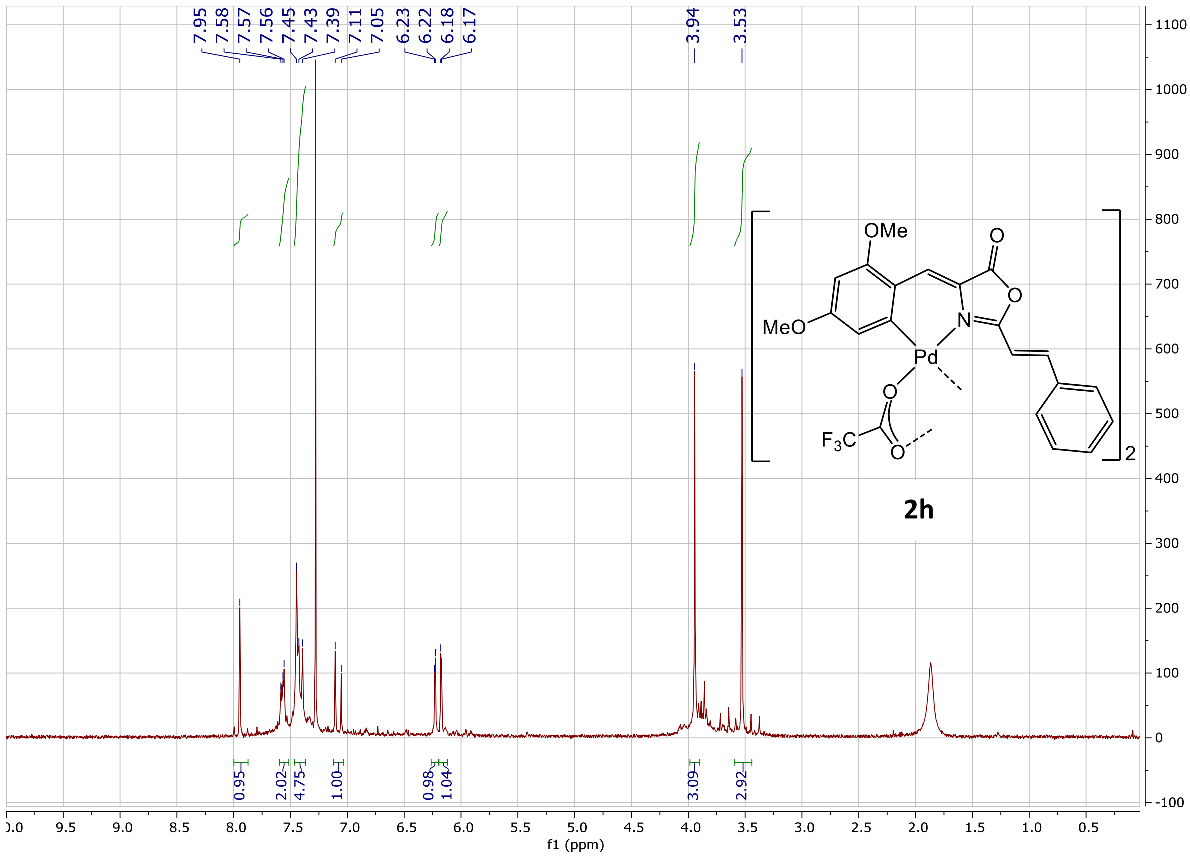

^1H-NMR spectrum ($CDCl_3$, 300.13 MHz) of **2h**[19b]

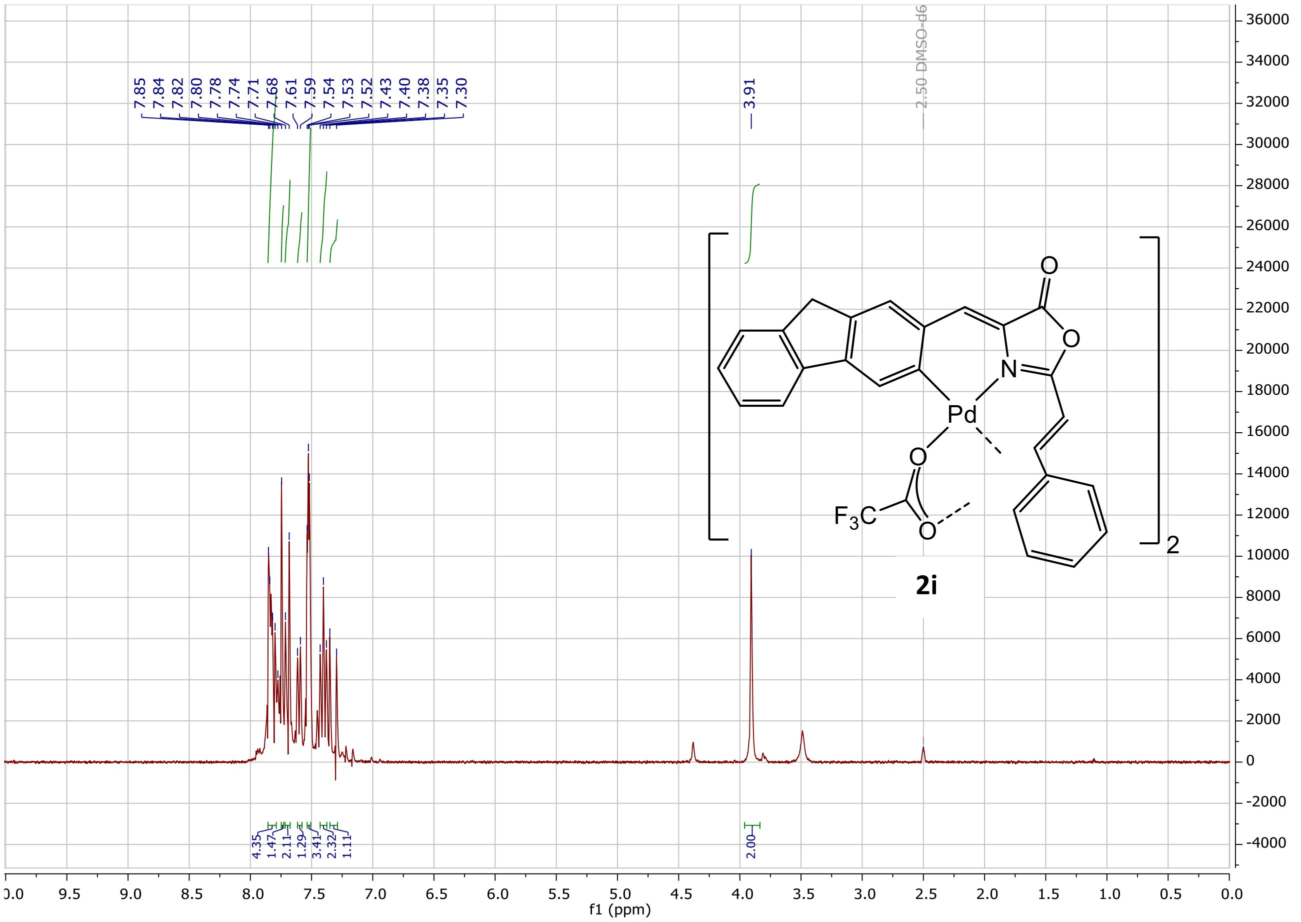

^{1}H-NMR spectrum (DMSO-d$_6$, 300.13 MHz) of **2i**

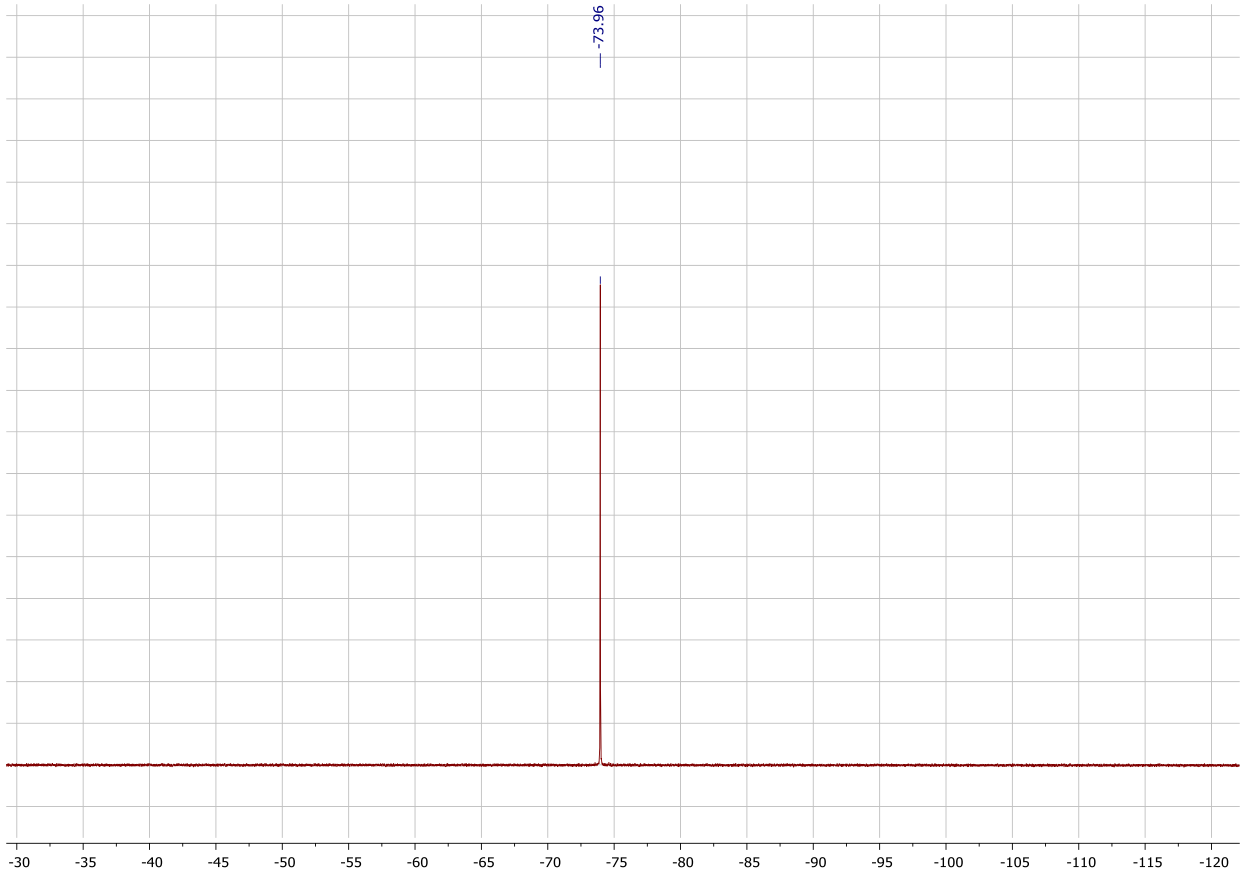

^{19}F-NMR spectrum (DMSO-d$_6$, 282.40 MHz) of **2i**

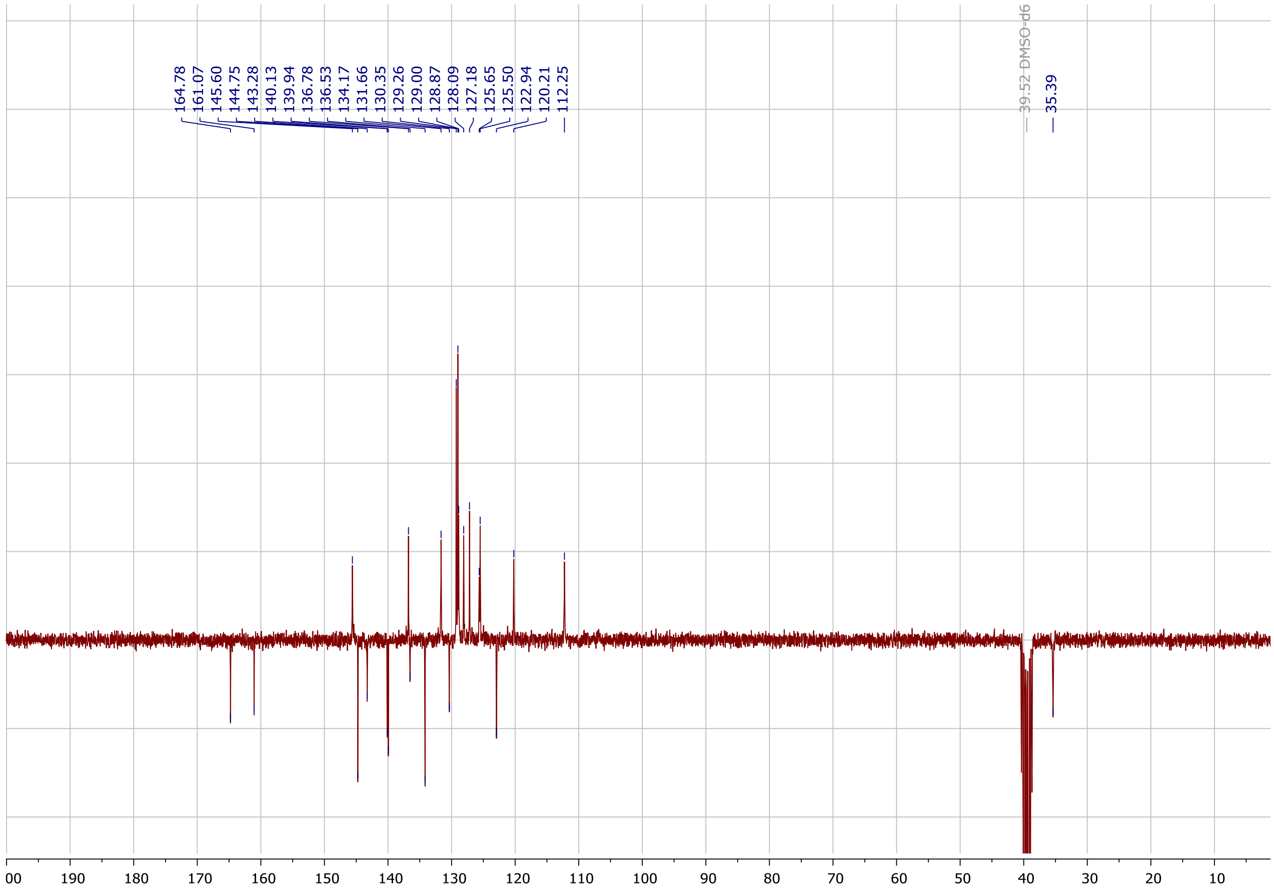

$^{13}C\{^{1}H\}$ (APT) NMR spectrum (DMSO-d_6, 75.47 MHz) of **2i**

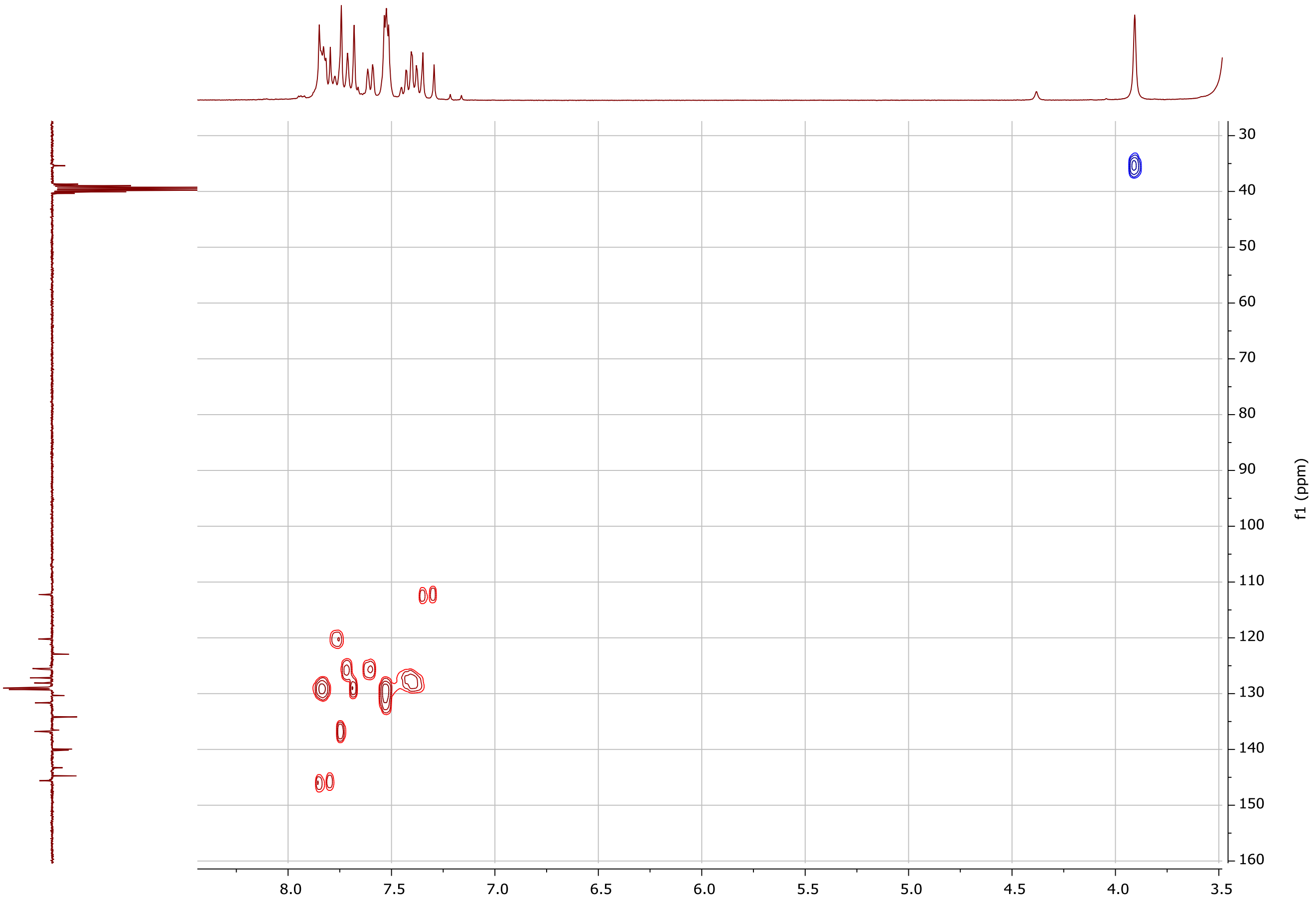

^{1}H - ^{13}C HSQC correlation spectrum of **2i**

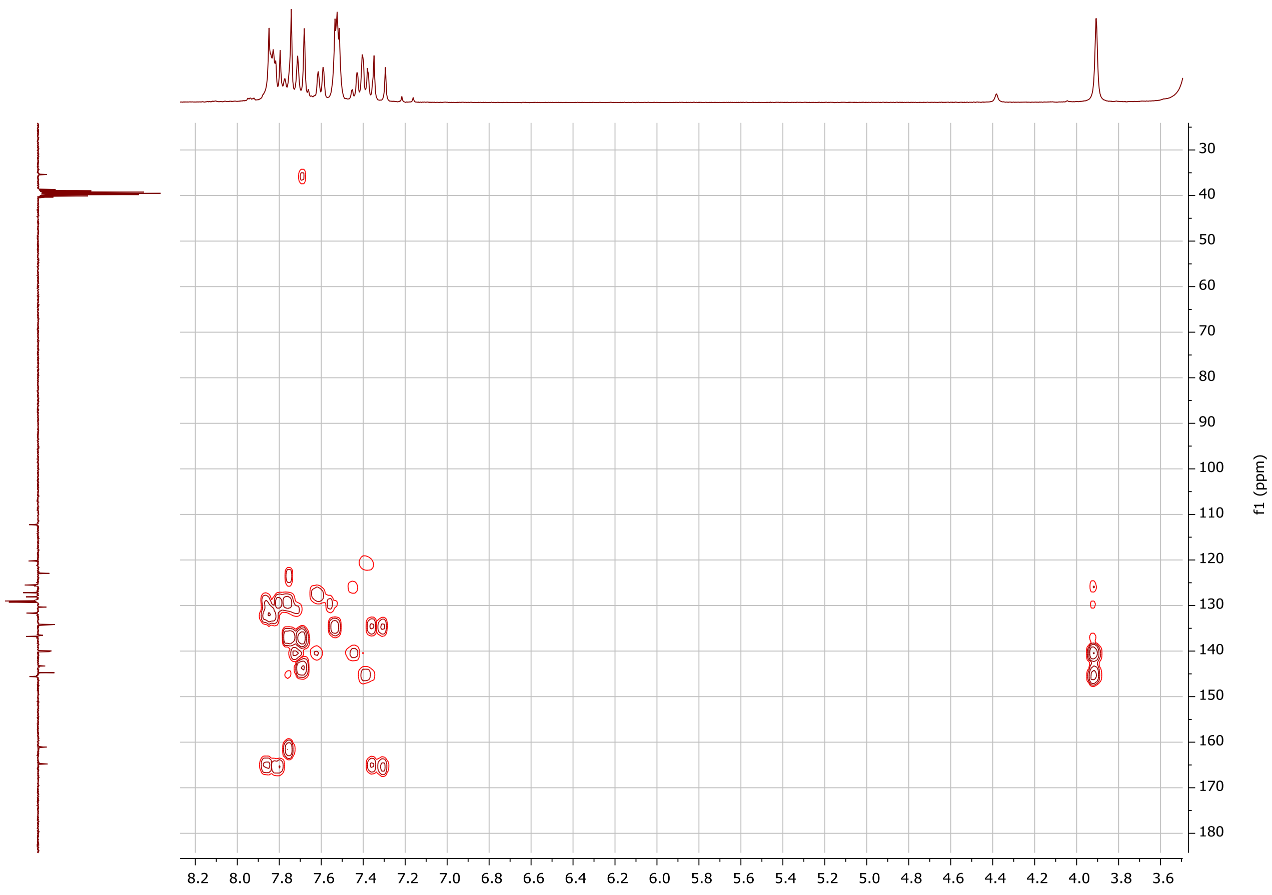

^{1}H - ^{13}C HMBC correlation spectrum of **2i**

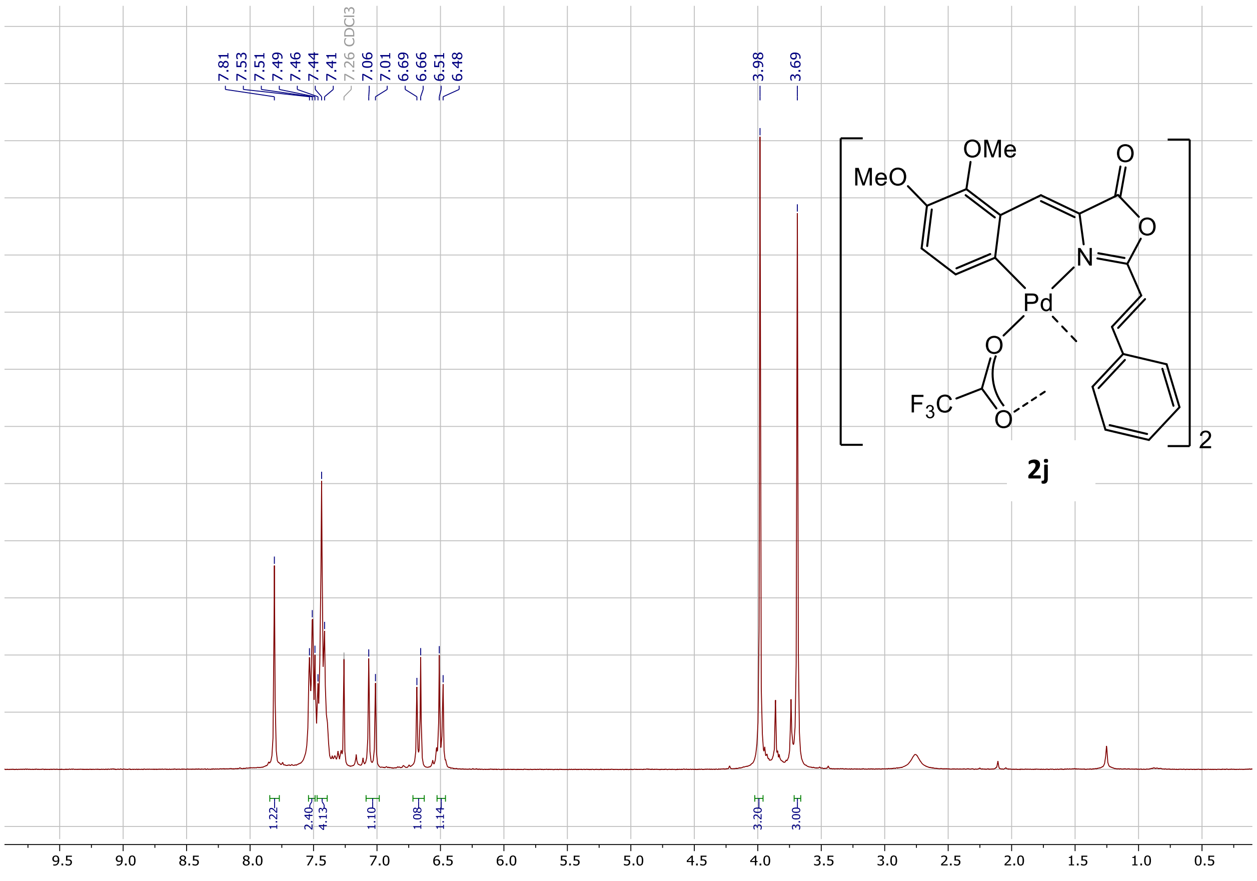

^{1}H-NMR spectrum ($CDCl_3$, 300.13 MHz) of **2j**

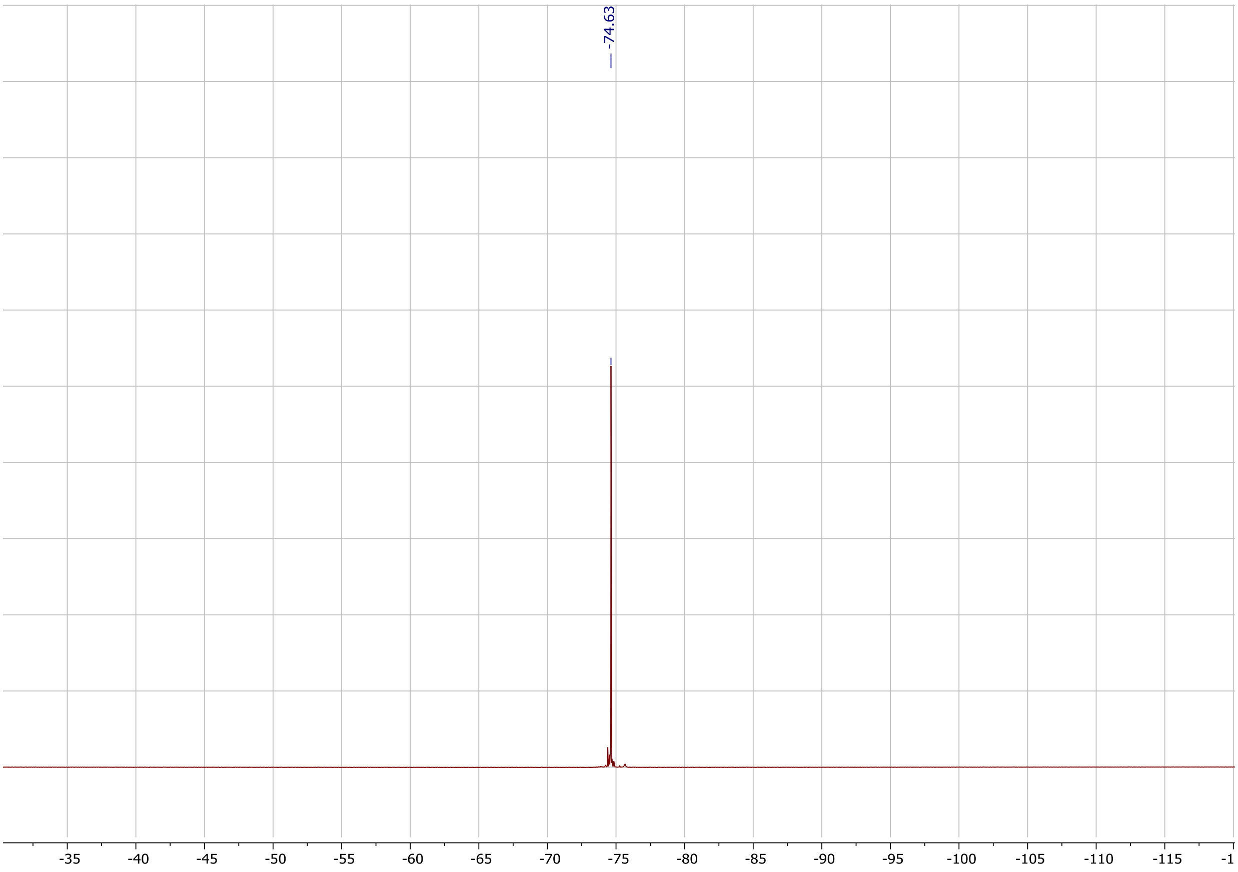

^{19}F-NMR spectrum ($CDCl_3$, 282.40 MHz) of **2j**

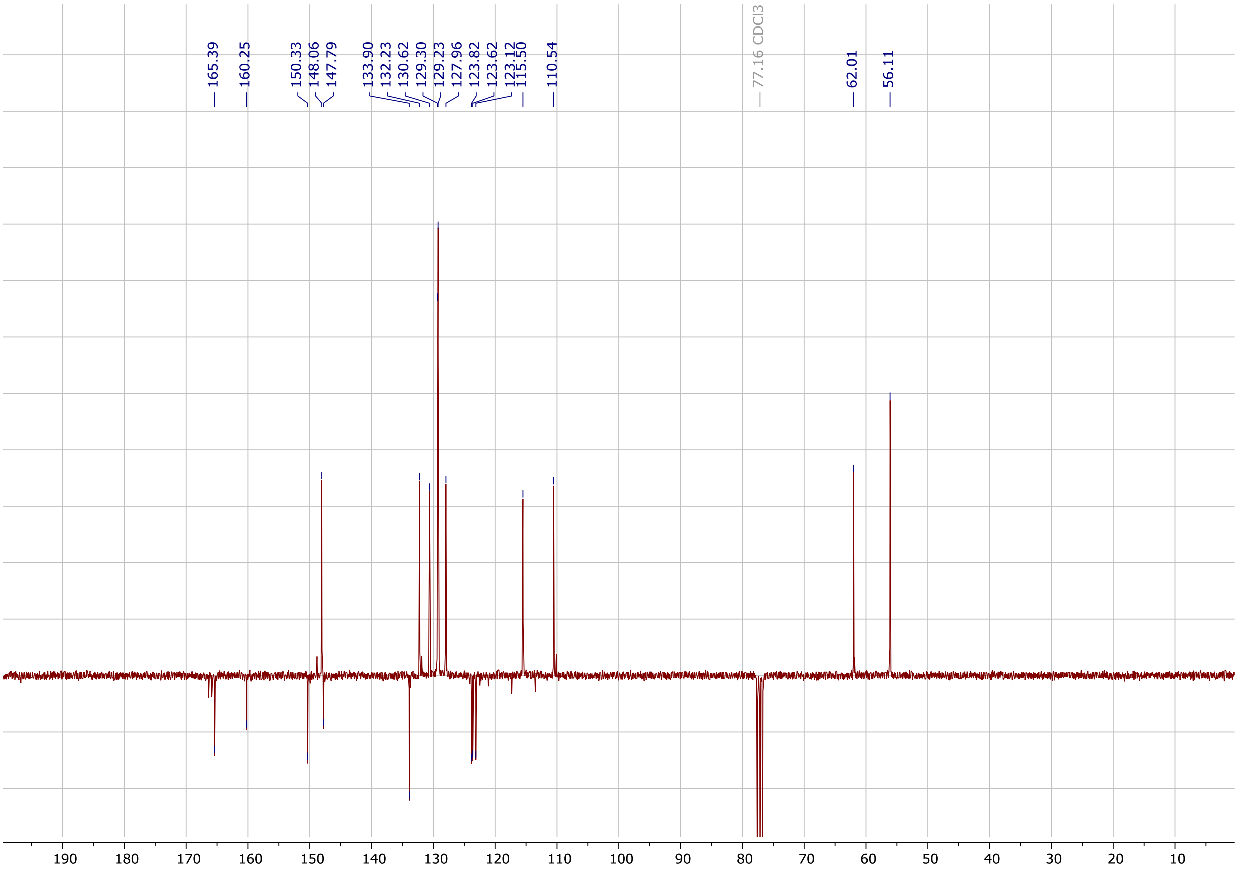

$^{13}C\{^1H\}$ (APT) NMR spectrum ($CDCl_3$, 75.47 MHz) of **2j**

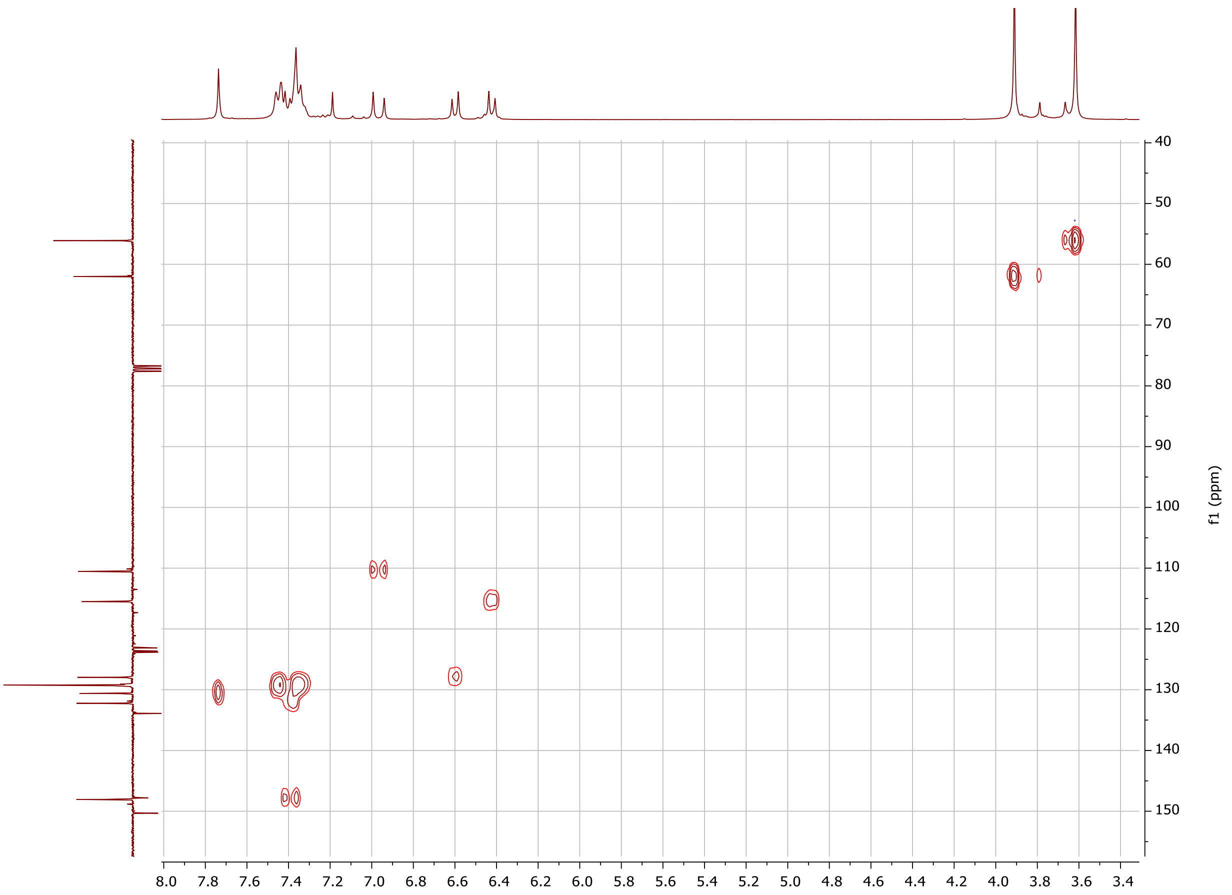

^{1}H - ^{13}C HSQC correlation spectrum of **2j**

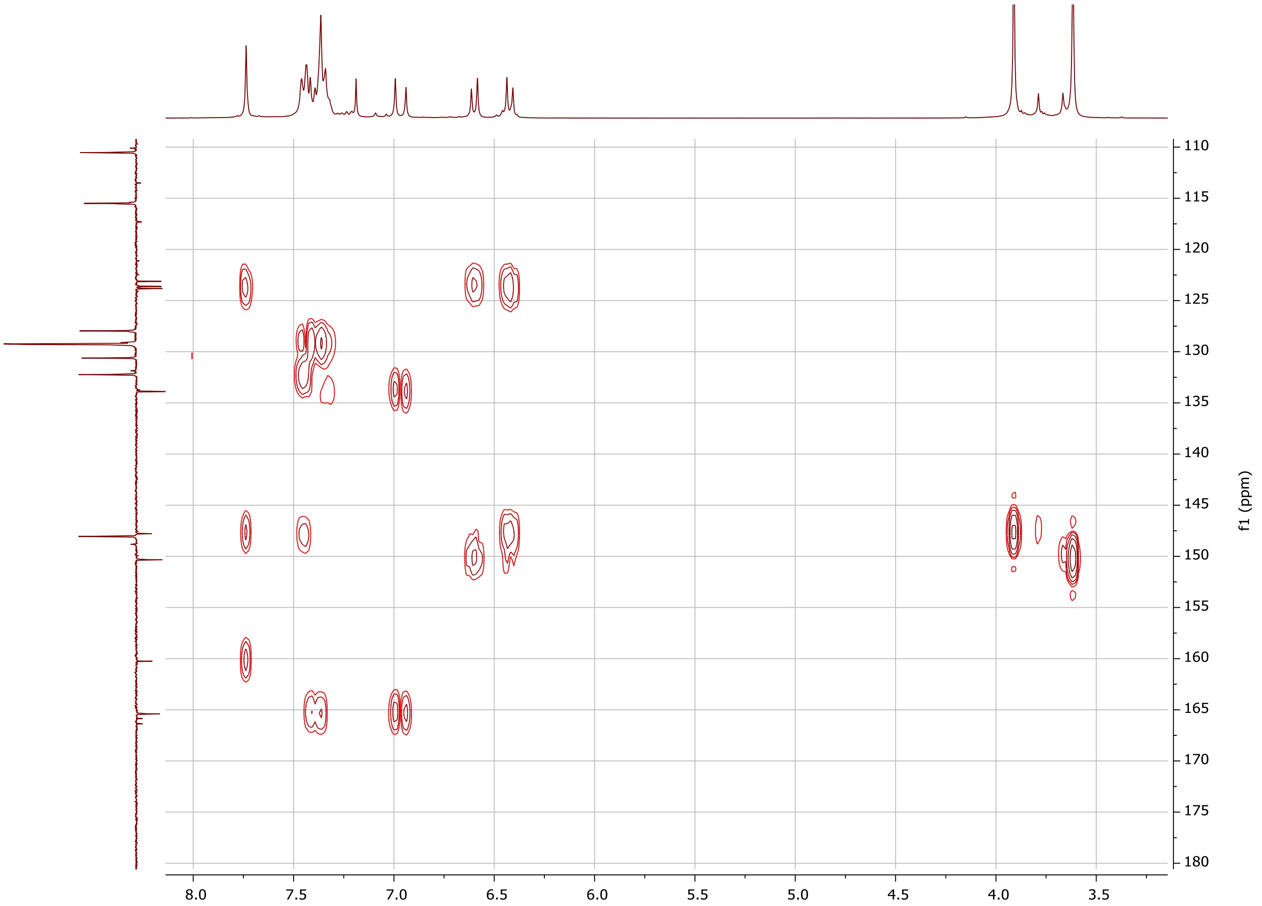

^{1}H - ^{13}C HMBC correlation spectrum of **2j**

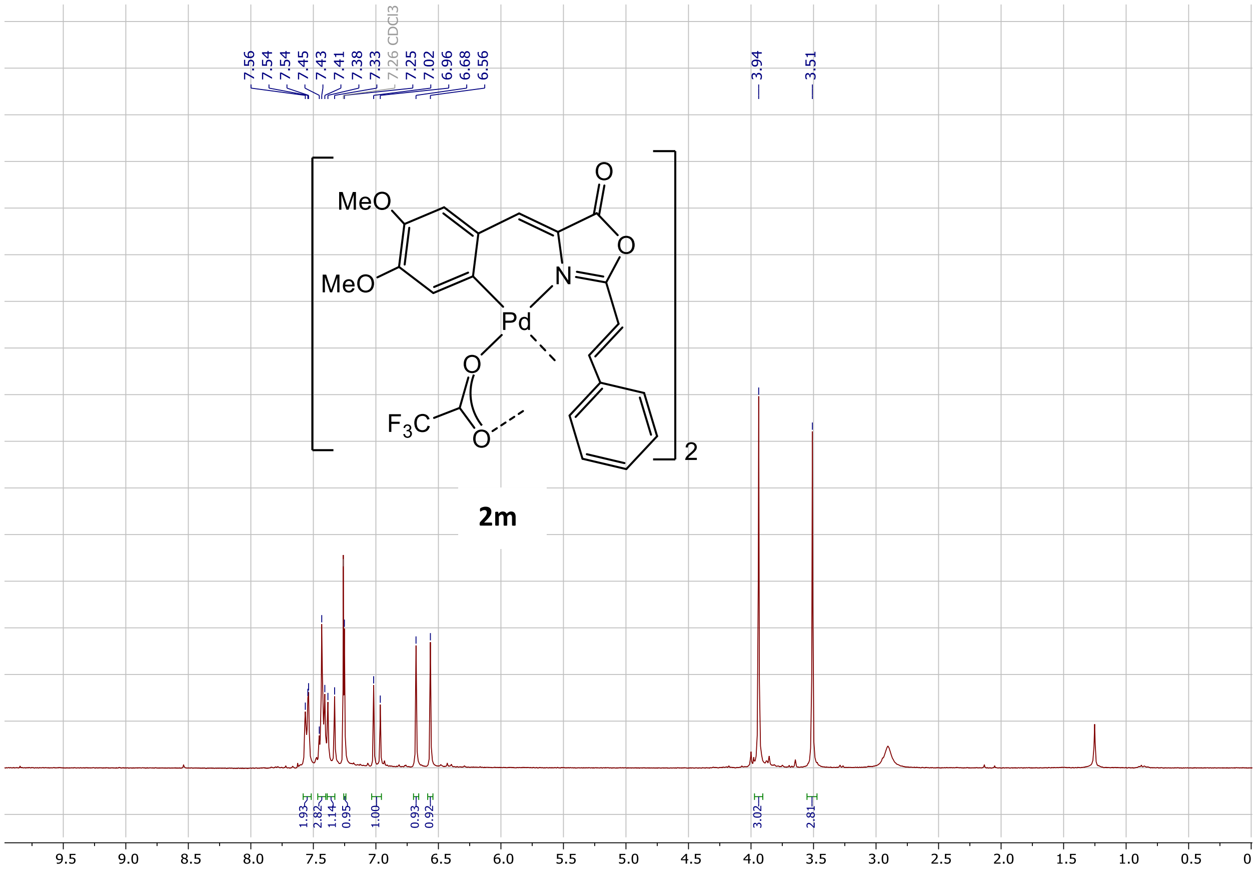

^{1}H-NMR spectrum ($CDCl_3$, 300.13 MHz) of **2m**

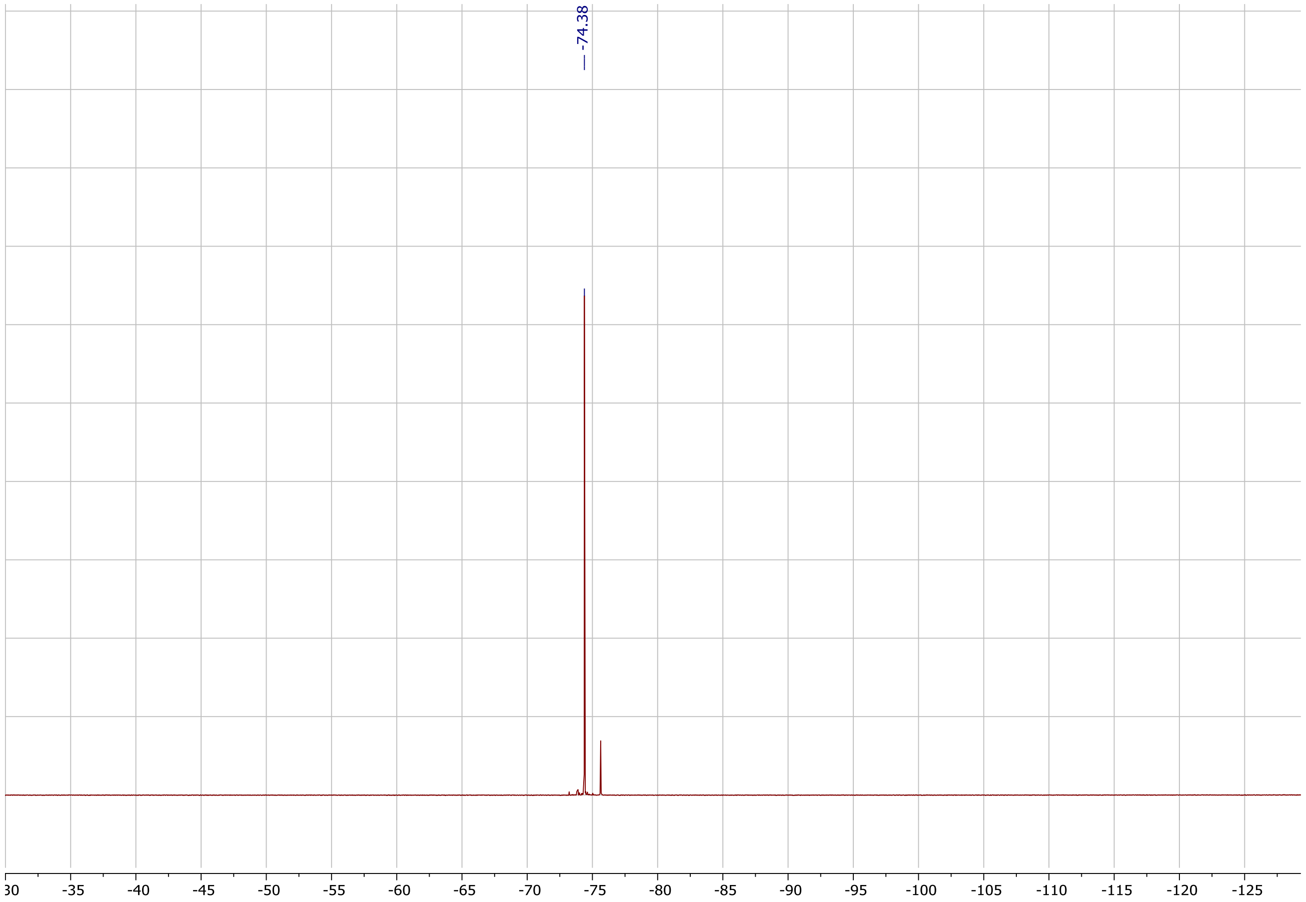

^{19}F-NMR spectrum ($CDCl_3$, 282.40 MHz) of **2m**

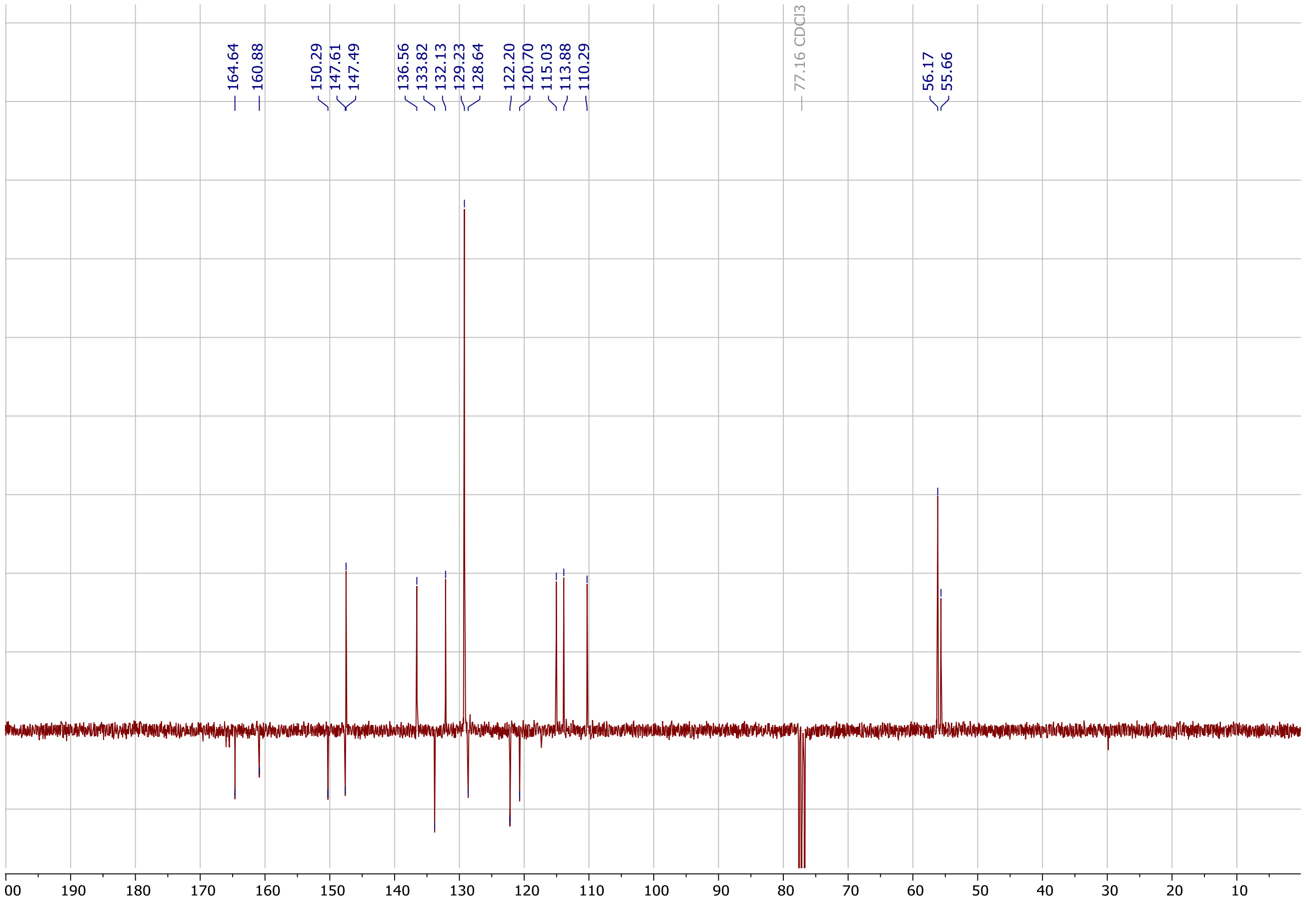

$^{13}C\{^{1}H\}$ (APT) NMR spectrum ($CDCl_3$, 75.47 MHz) of **2m**

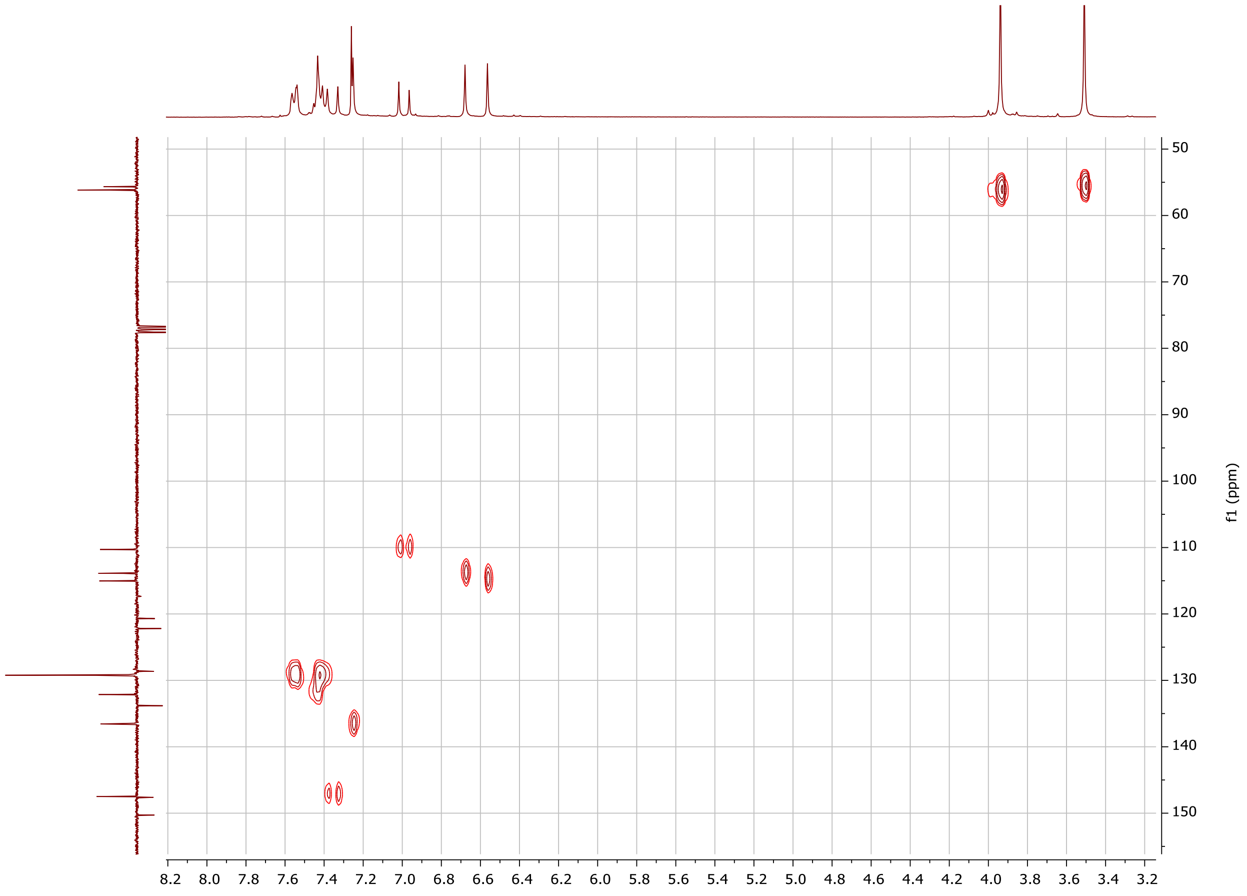

^{1}H - ^{13}C HSQC correlation spectrum of **2m**

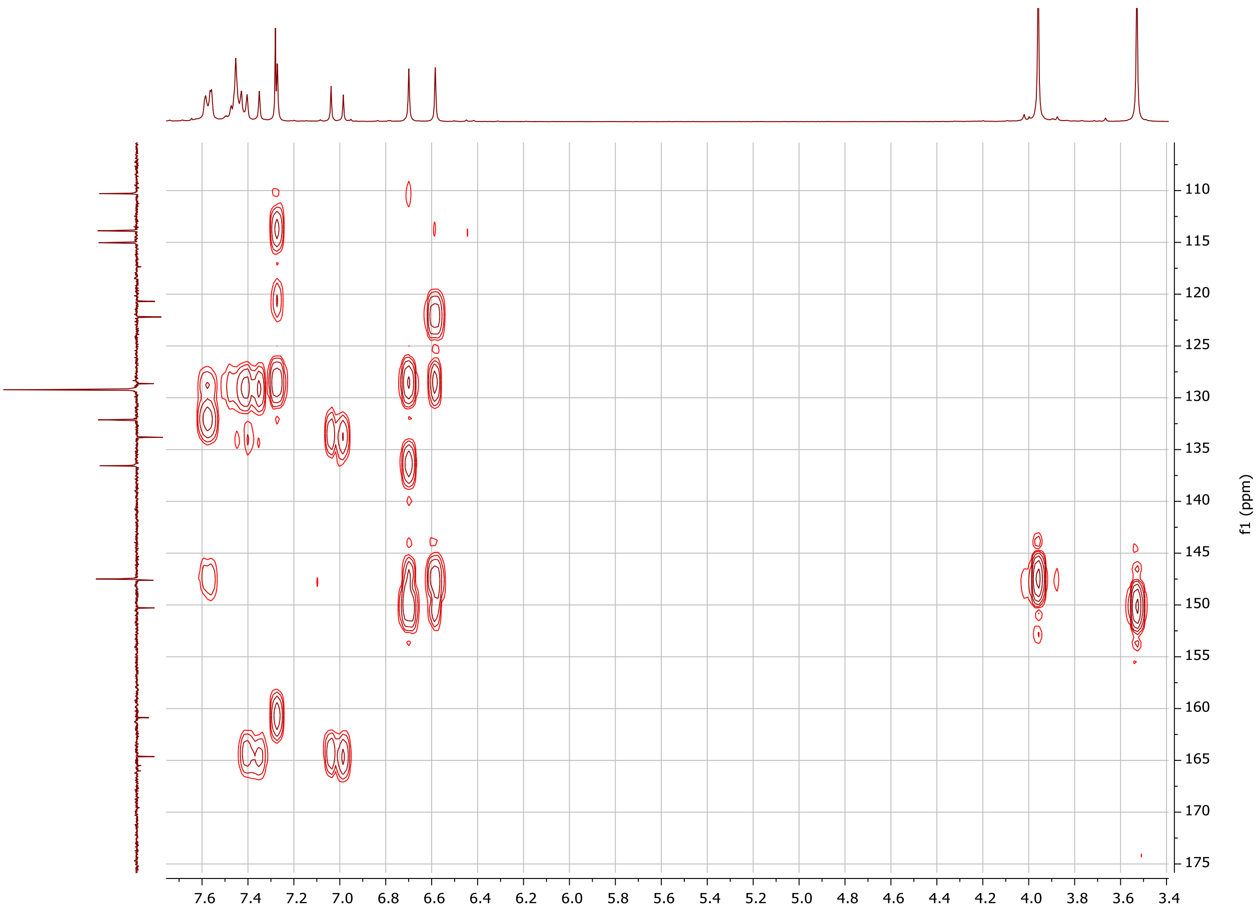

^{1}H - ^{13}C HMBC correlation spectrum of **2m**

4.- NMR spectra of mononuclear trifluoroacetate-pyridine orthopalladated derivatives (3)

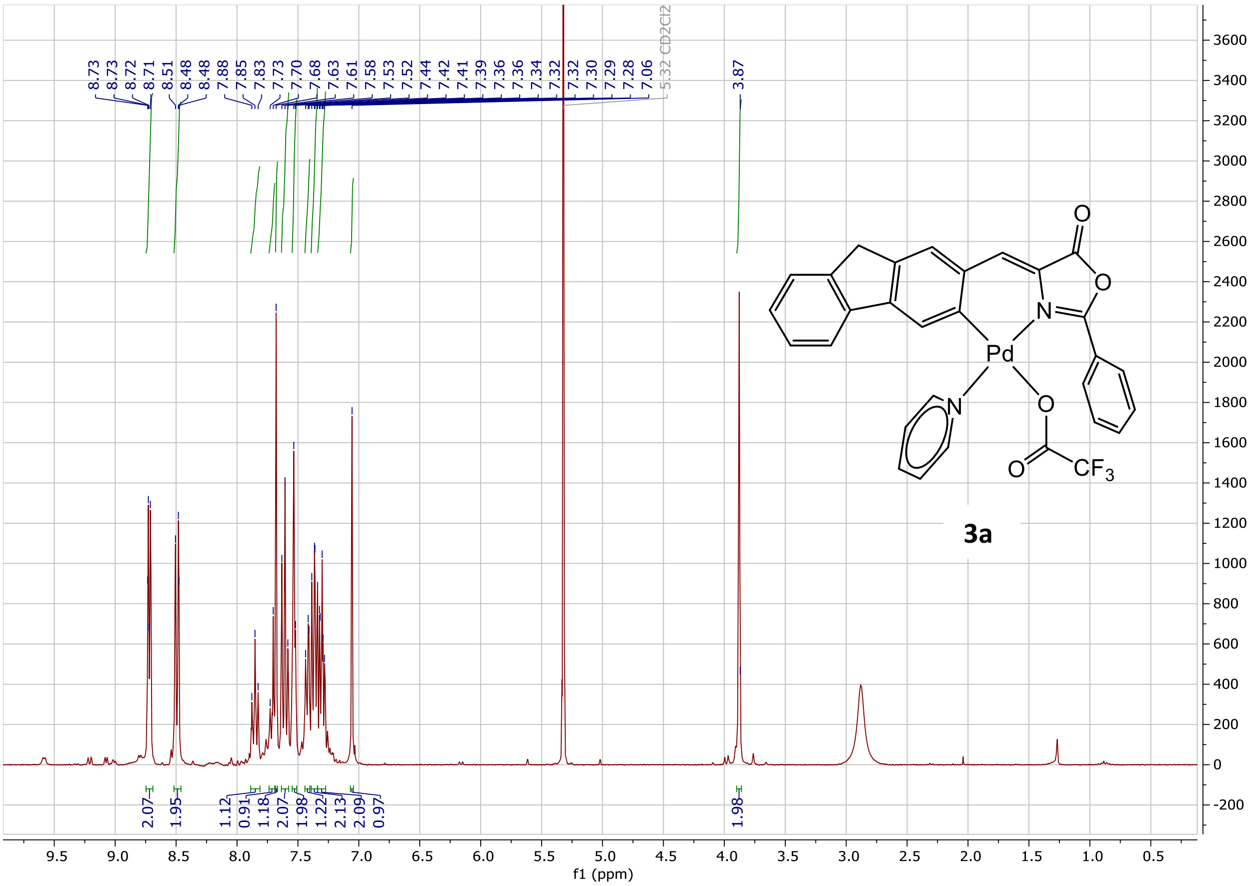

^{1}H-NMR spectrum (CD_2Cl_2, 300.13 MHz) of **3a**

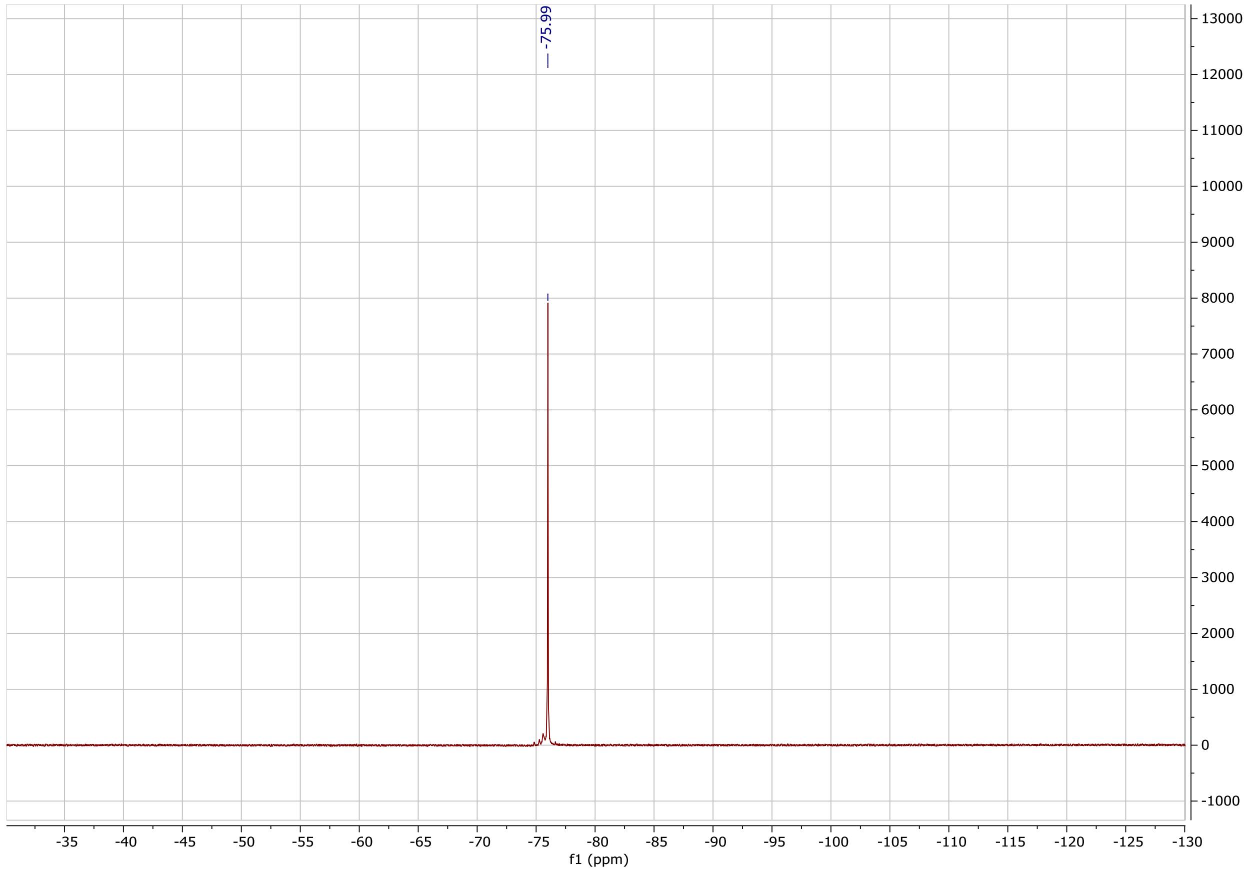

^{19}F-NMR spectrum (CD_2Cl_2, 282.40 MHz) of **3a**

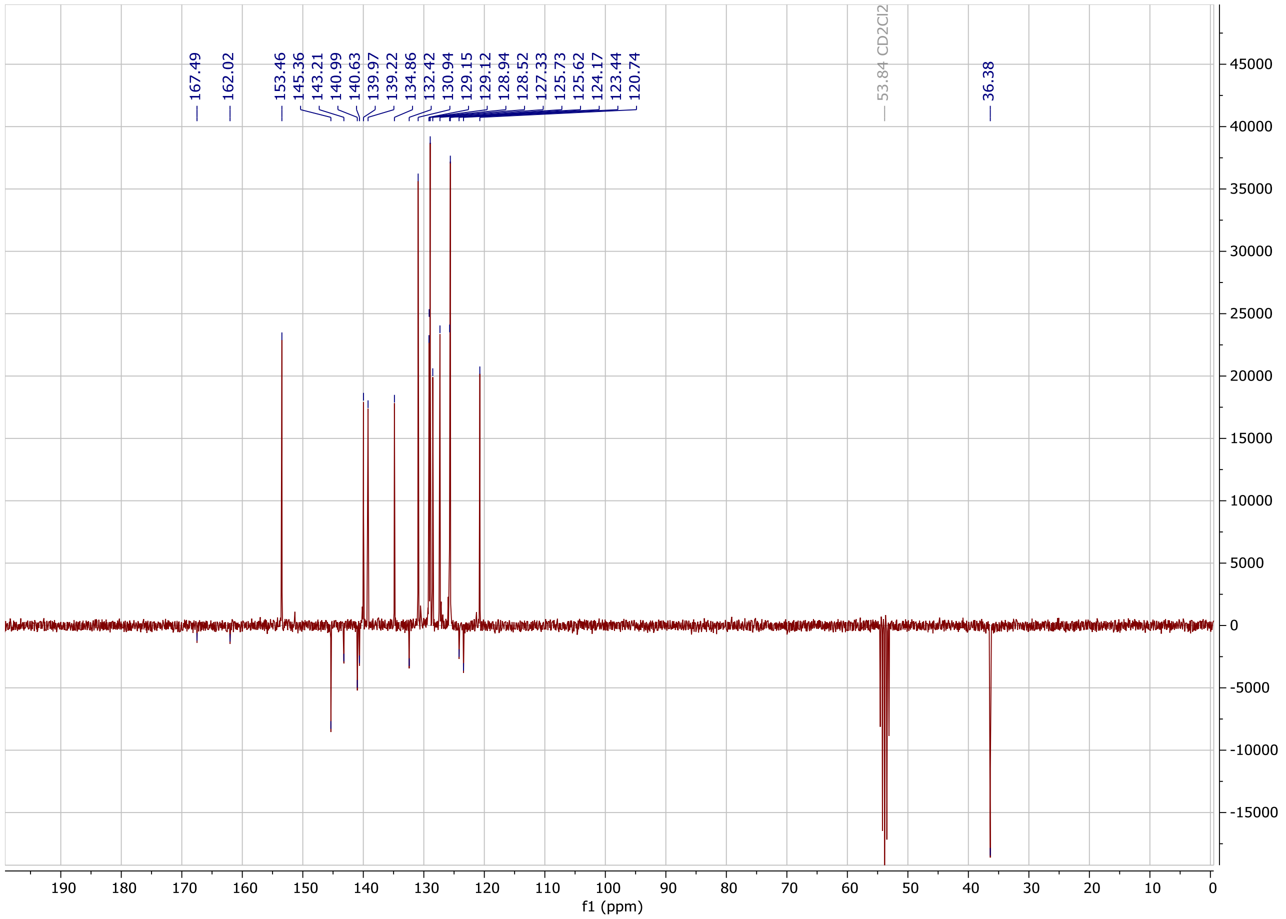

$^{13}C\{^1H\}$ (APT) NMR spectrum (CD_2Cl_2, 75.47 MHz) of **3a**

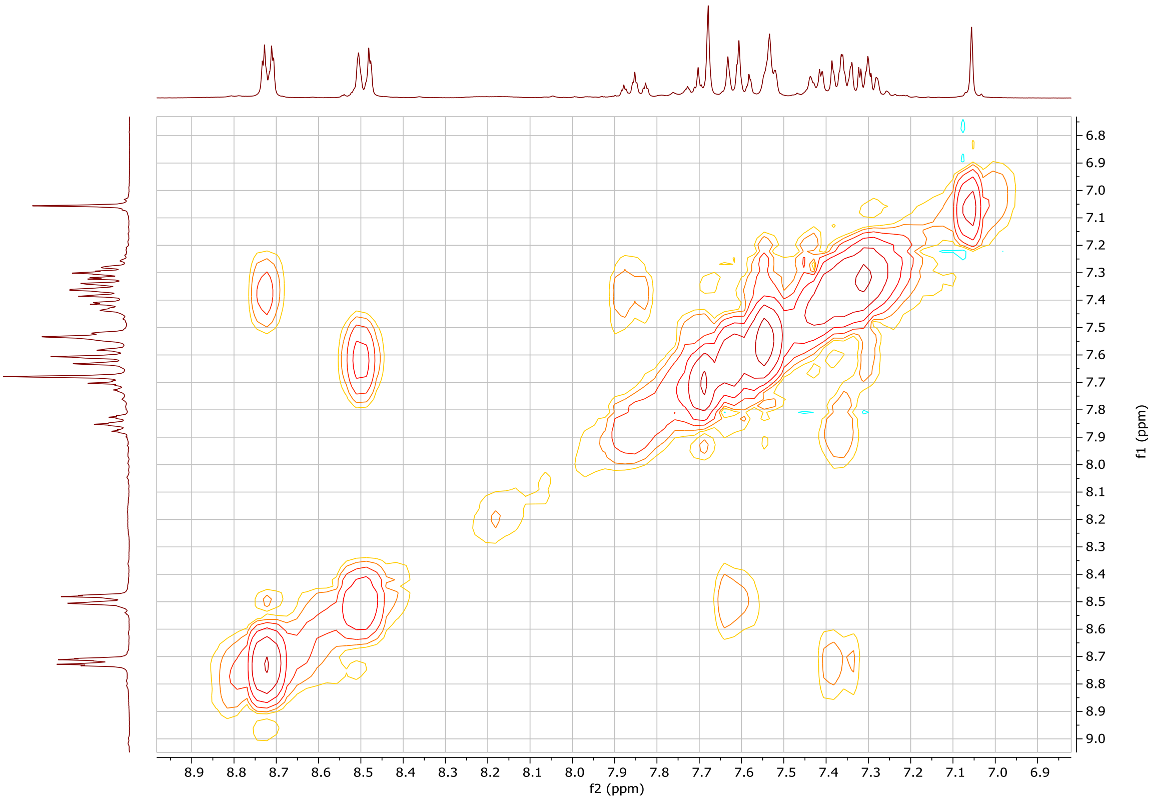

$^1H-^1H$ COSY correlation spectrum of **3a** (aromatic region)

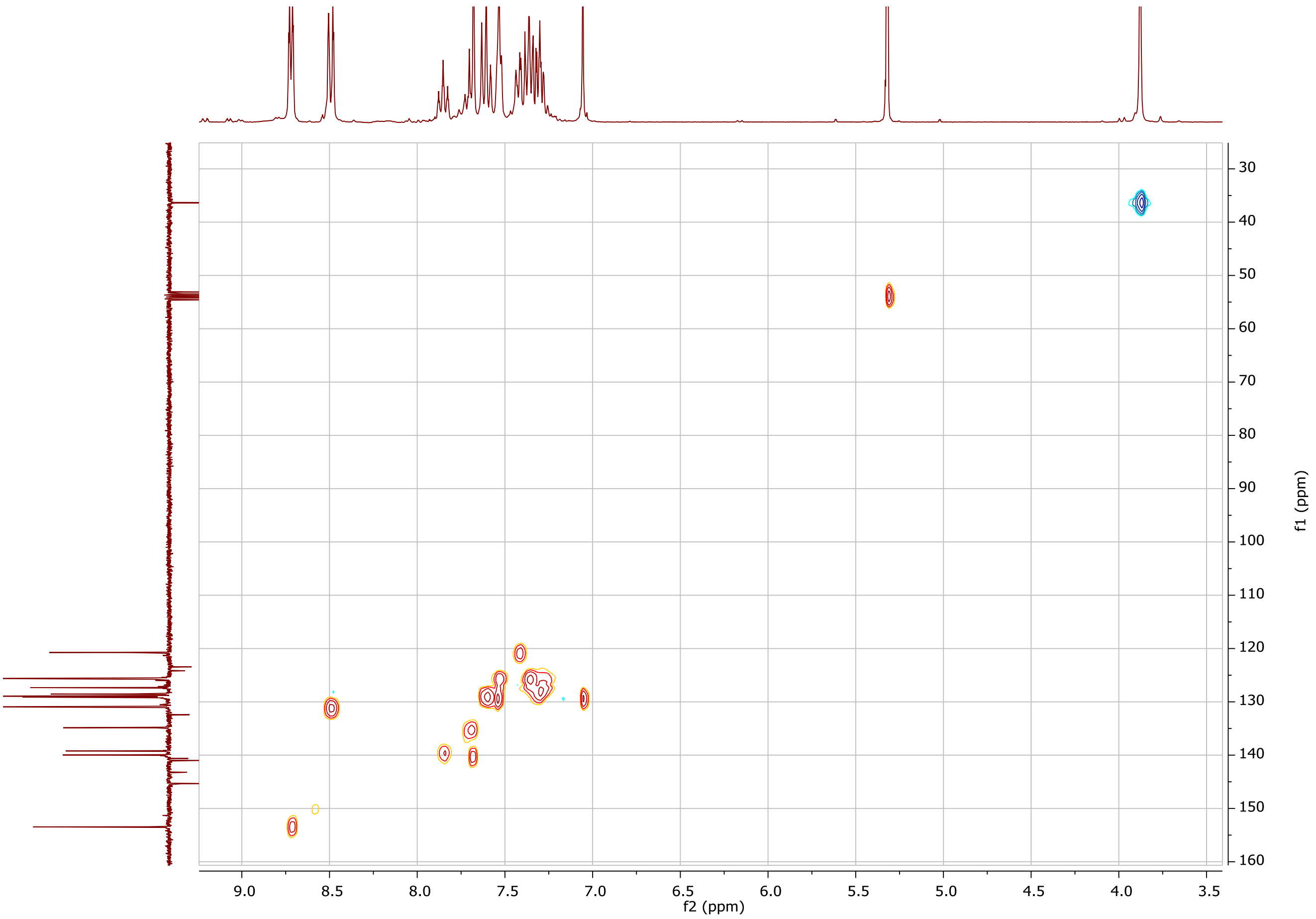

^{1}H - ^{13}C HSQC correlation spectrum of **3a**

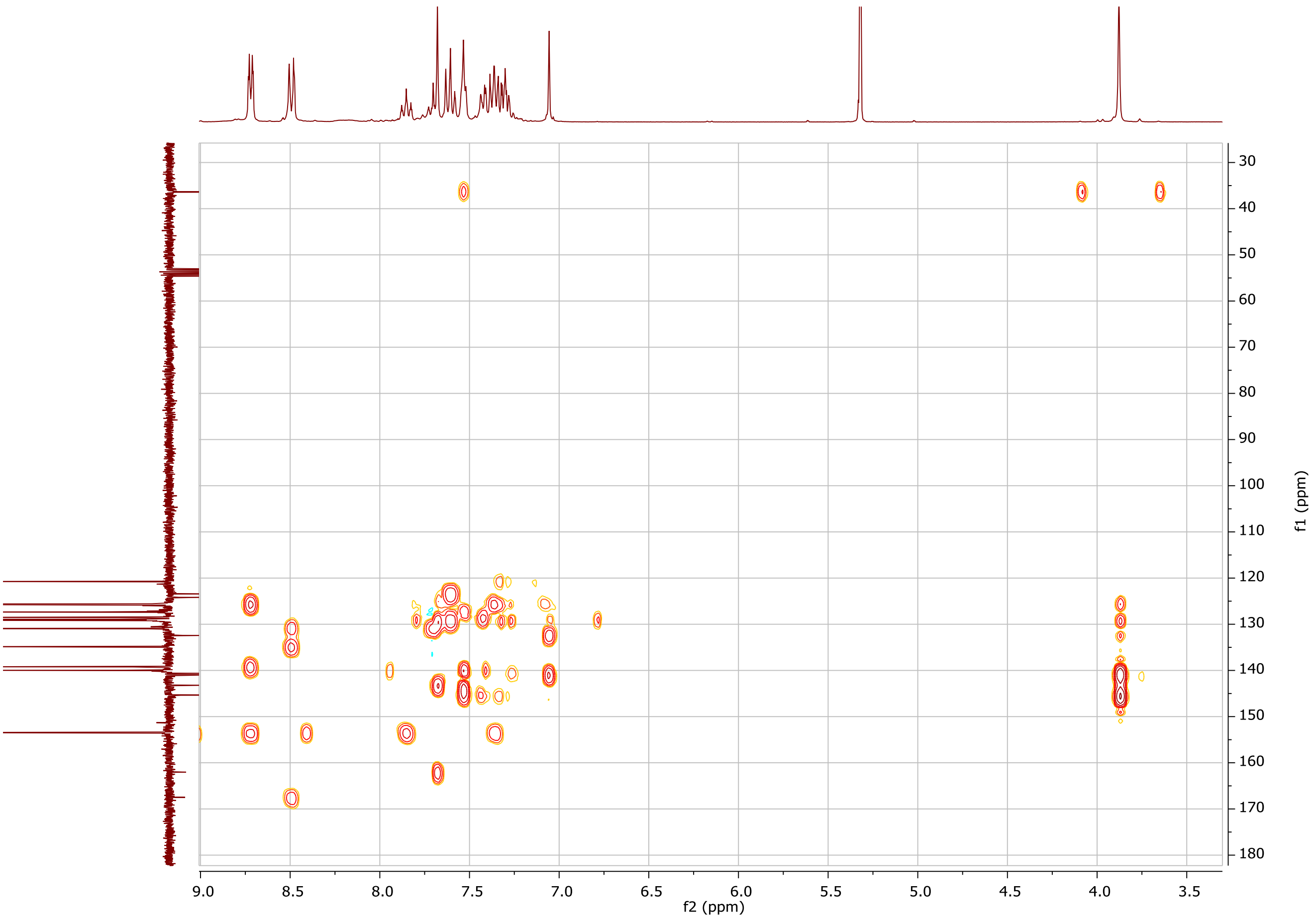

^{1}H - ^{13}C HMBC correlation spectrum of **3a**

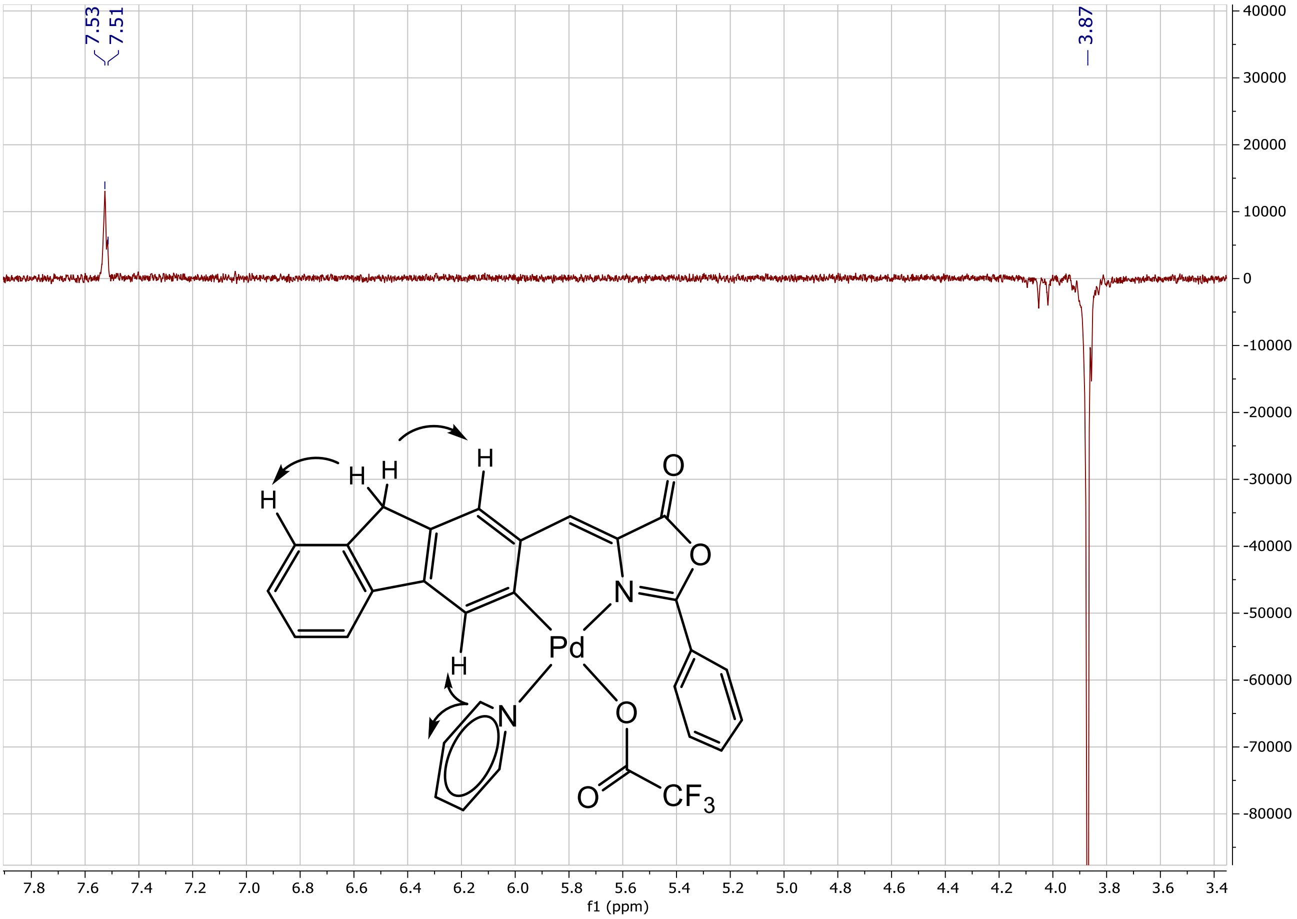

1D-SELNOE experiment (CD_2Cl_2) of **3a**: Irradiation a 3.87 ppm (CH_2) gives NOE at 7.53 (s) and 7.52 (d) ppm

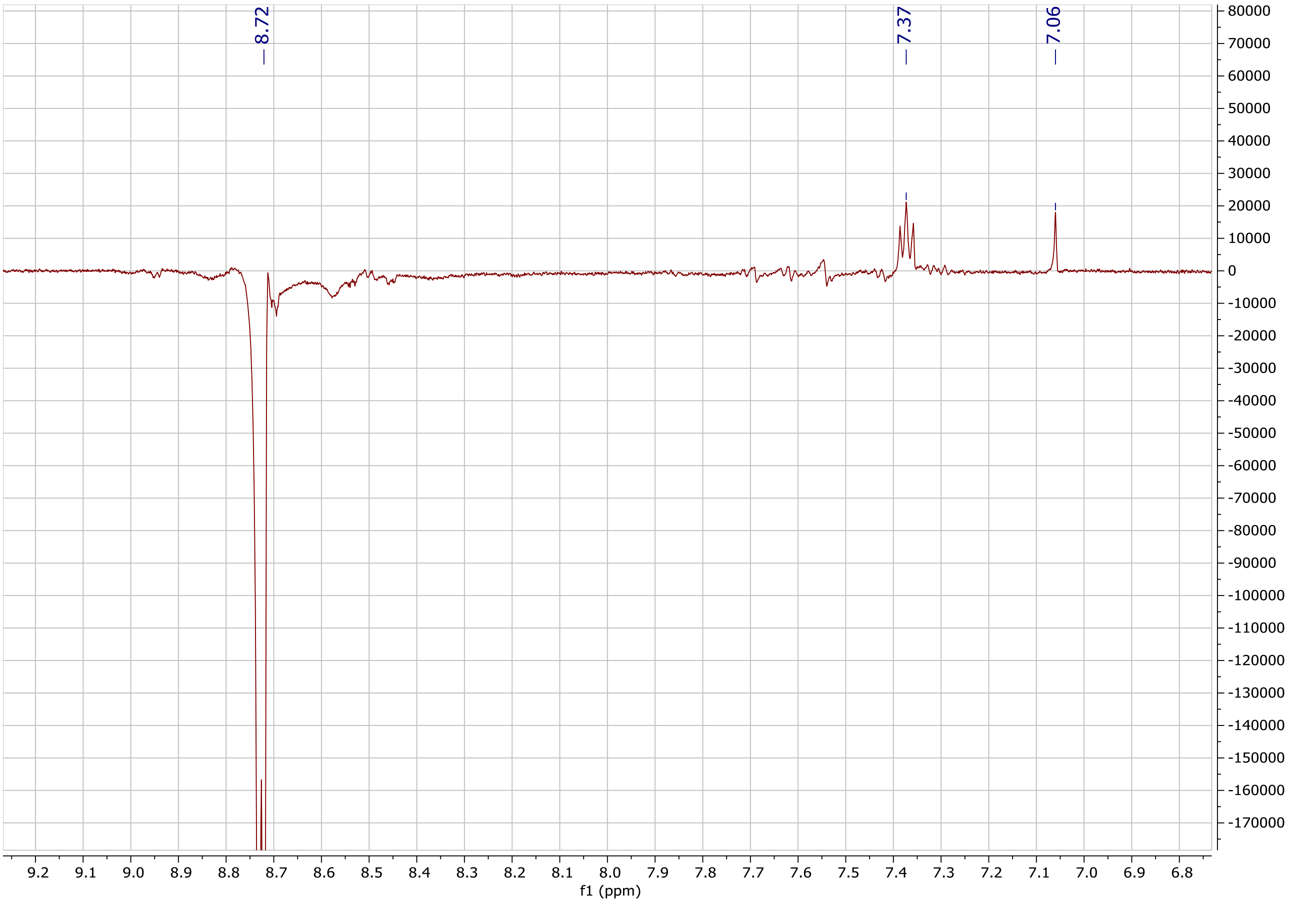

1D-SELNOE experiment (CD_2Cl_2) of **3a**: Irradiation at 8.72 ppm (H_o, py) gives NOE at 7.06 (s) and 7.51 (H_m, py) ppm

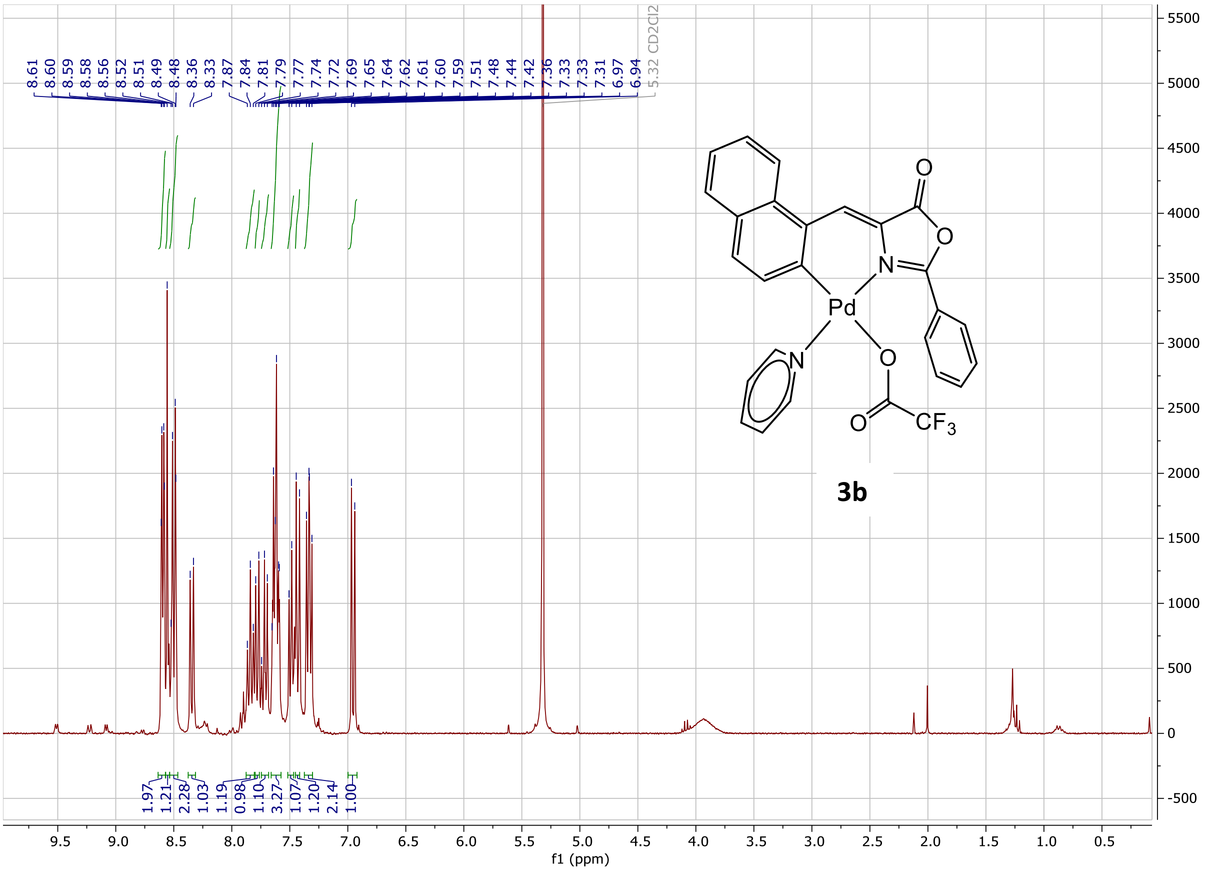

^{1}H-NMR spectrum (CD_2Cl_2, 300.13 MHz) of **3b**

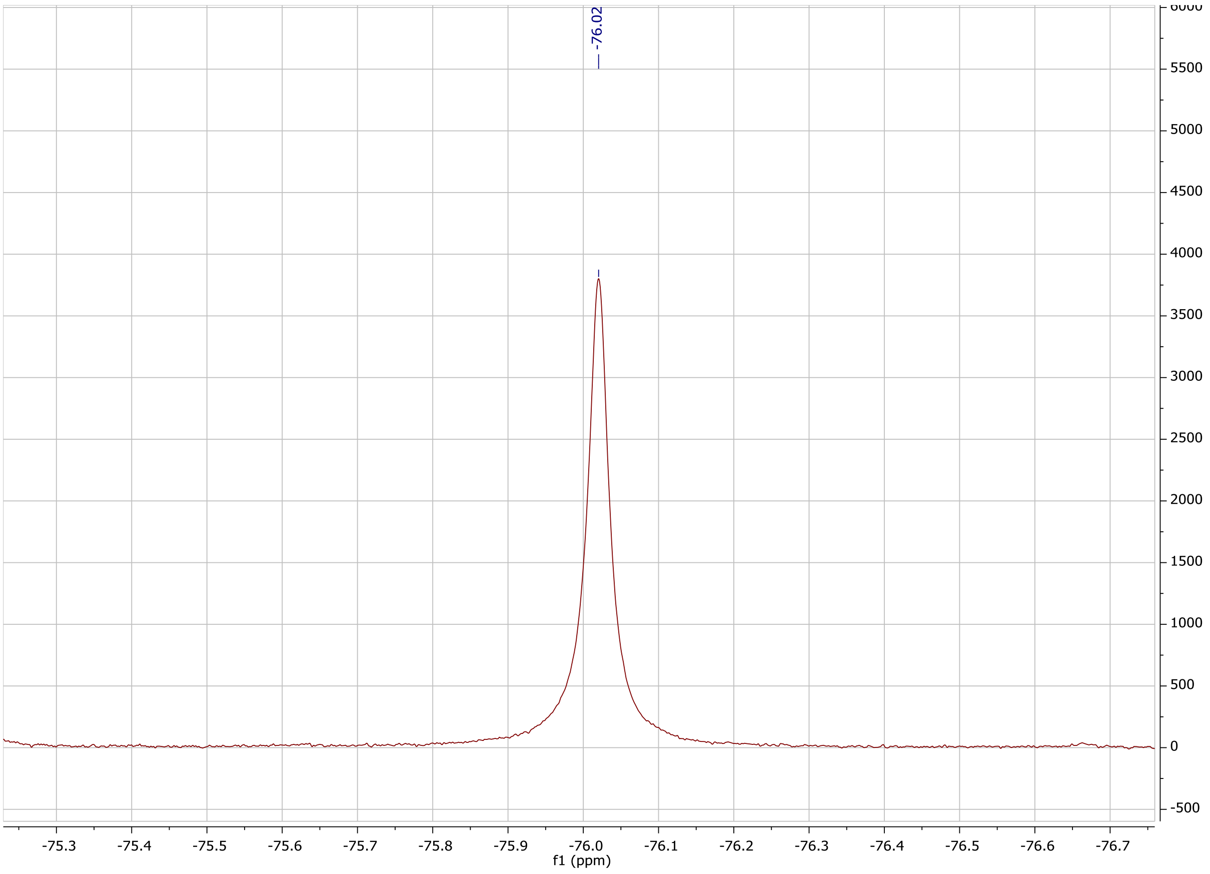

^{19}F-NMR spectrum (CD_2Cl_2, 282.40 MHz) of **3b**

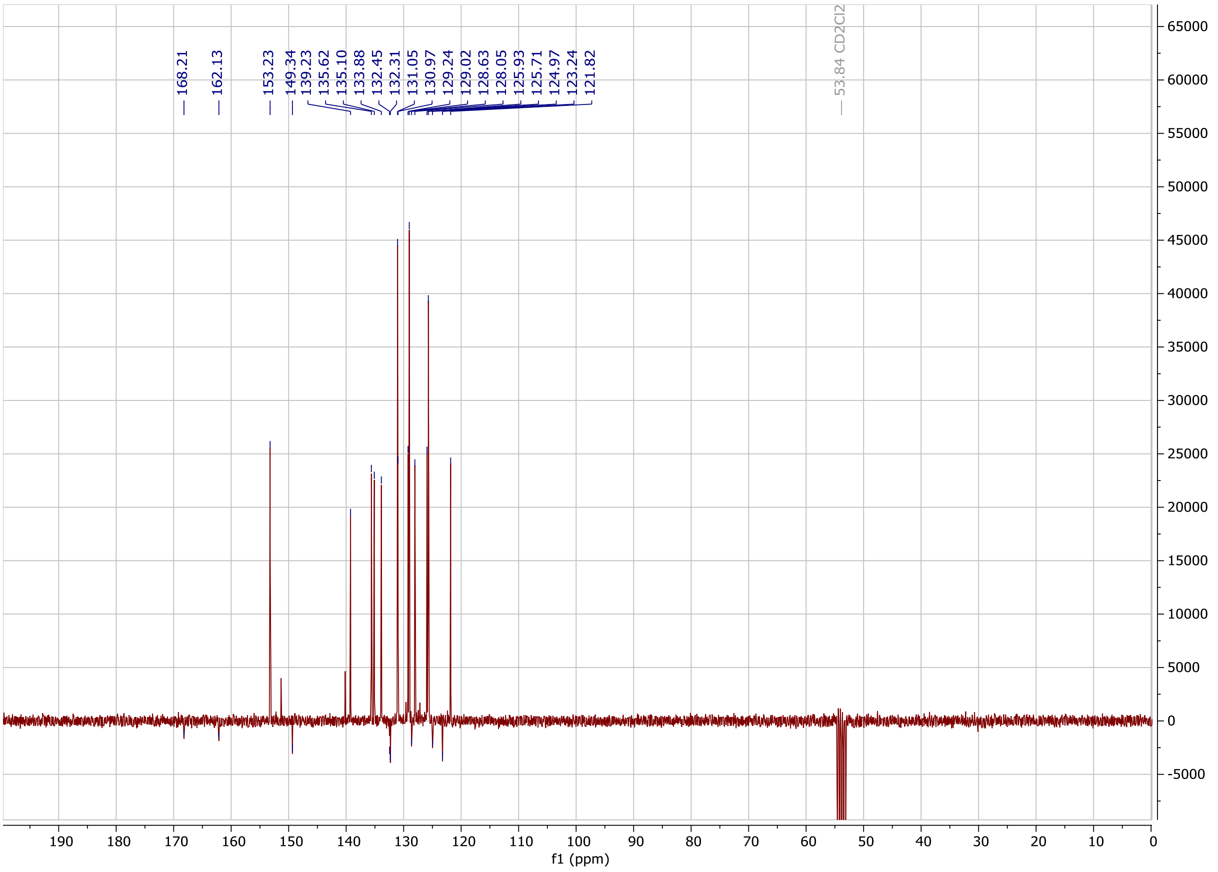

$^{13}C\{^{1}H\}$ (APT) NMR spectrum (CD_2Cl_2, 75.47 MHz) of **3b**

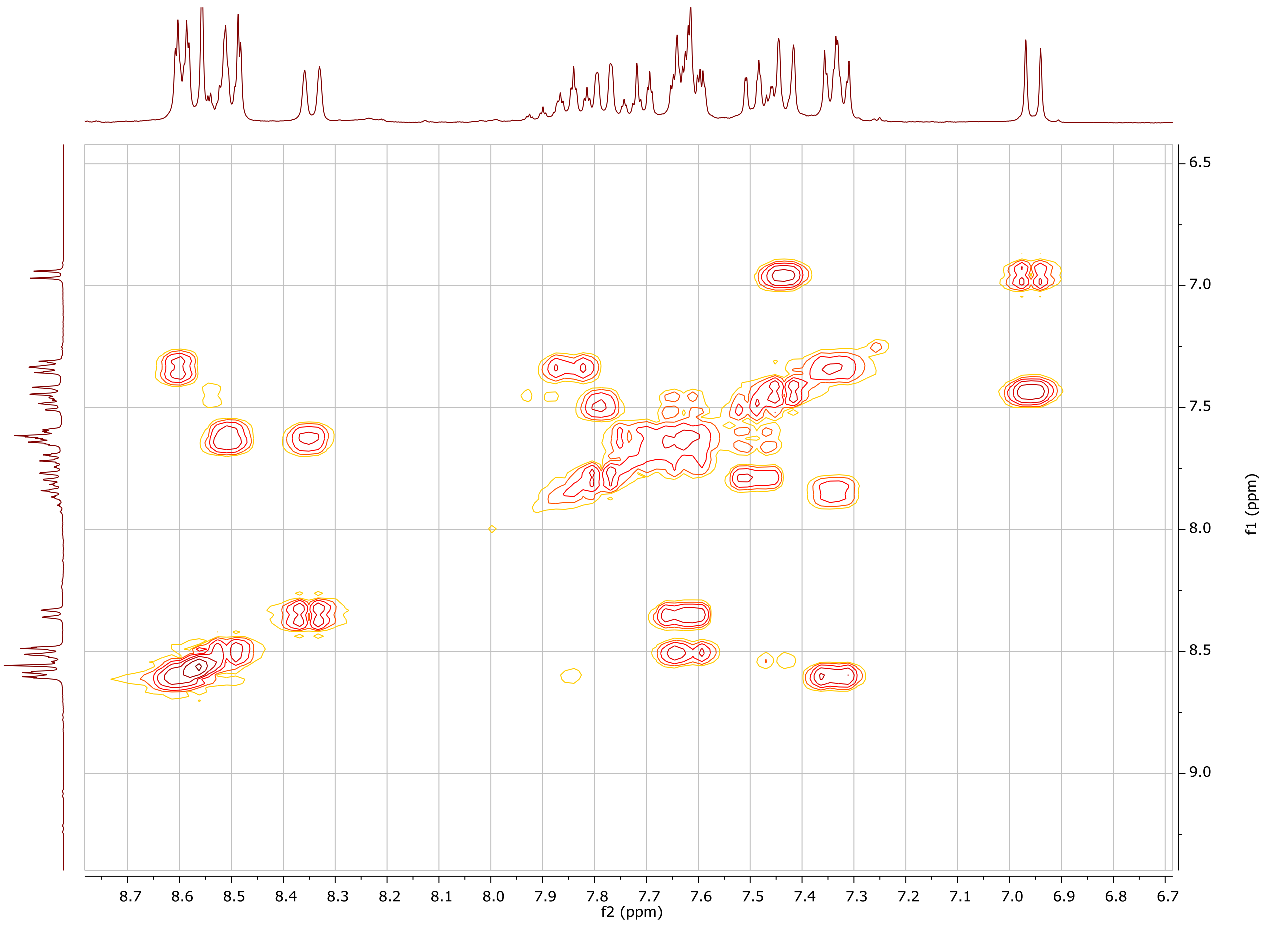

$^{1}H-^{1}H$ COSY correlation spectrum of **3b**

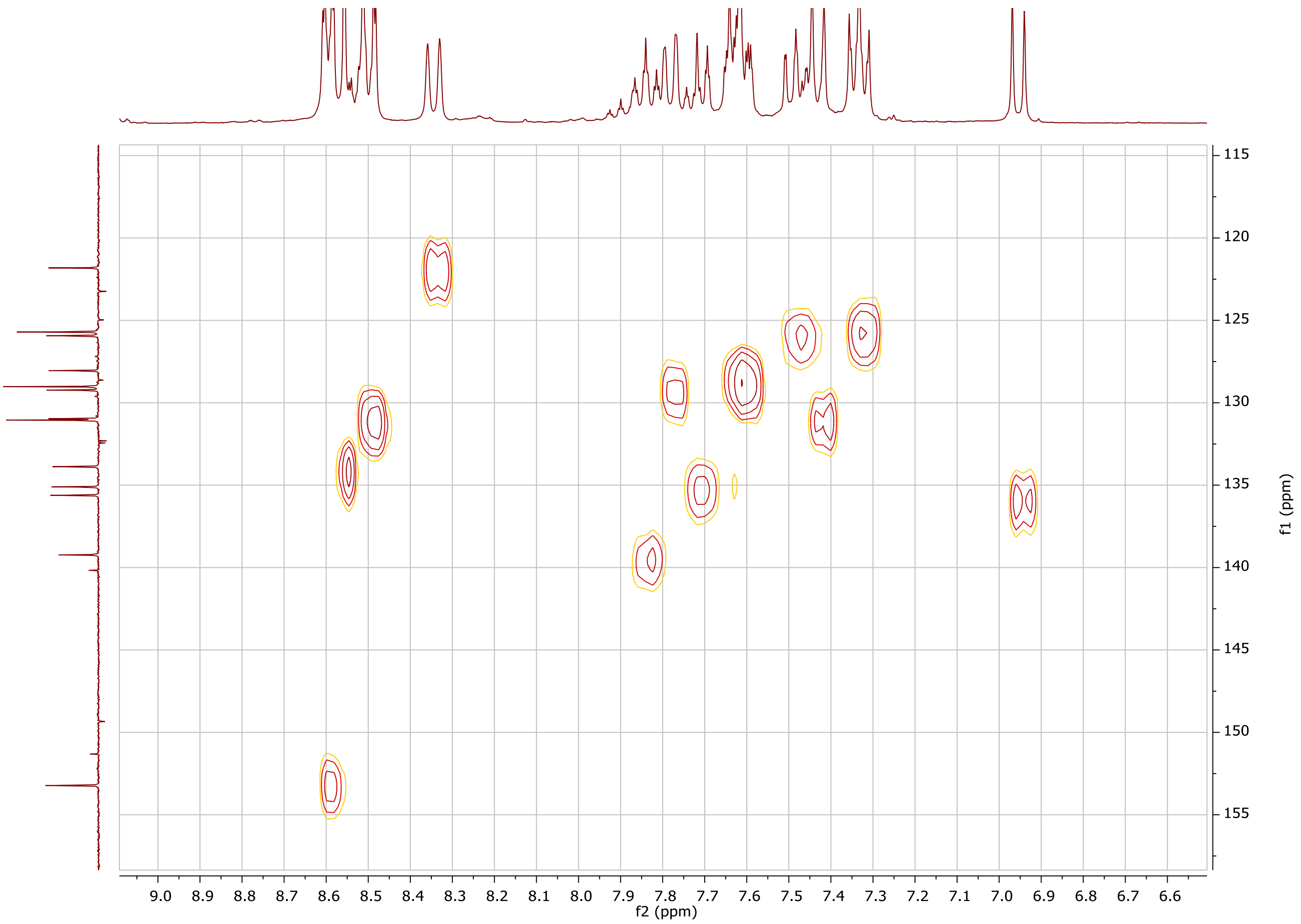

^{1}H - ^{13}C HSQC correlation spectrum of **3b**

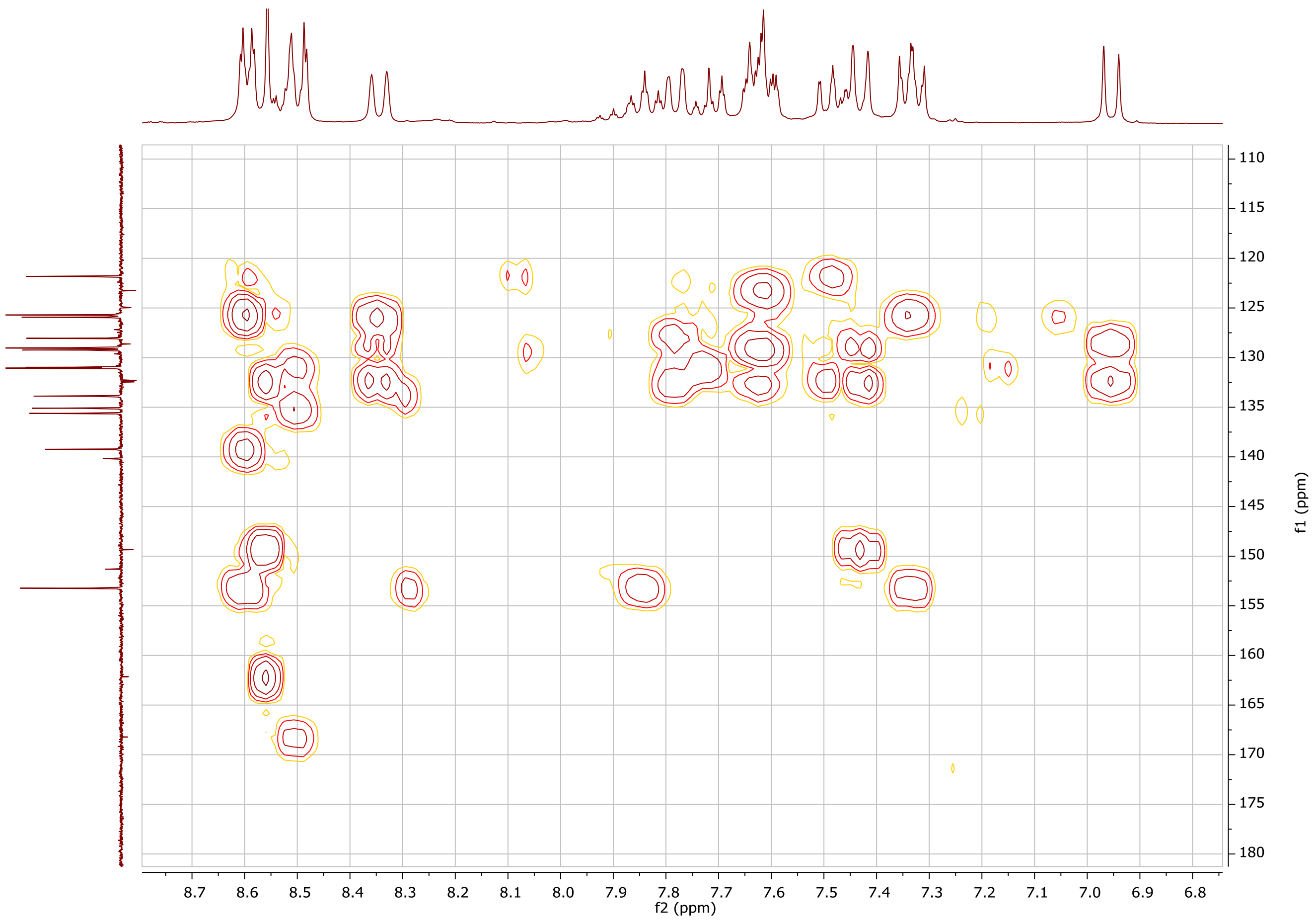

^{1}H - ^{13}C HMBC correlation spectrum of **3b**

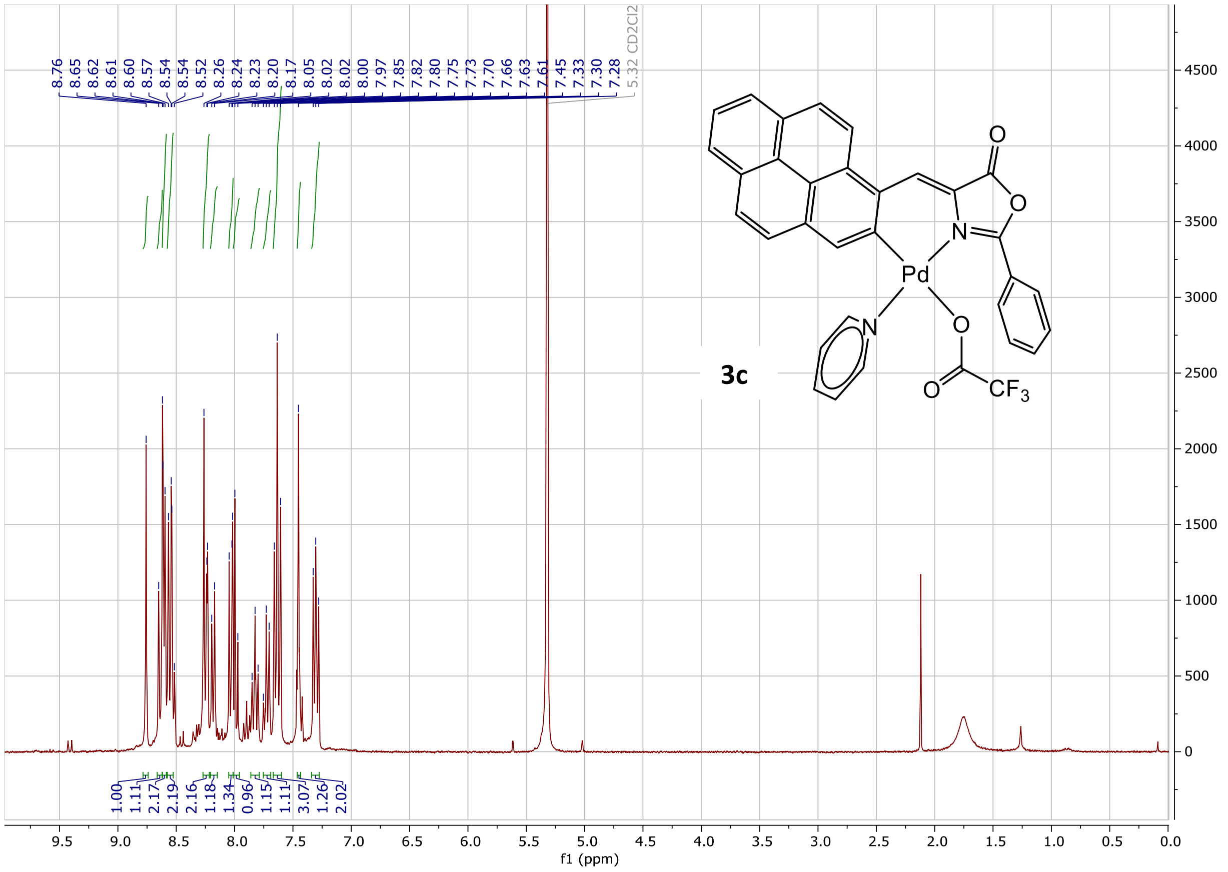

^{1}H-NMR spectrum (CD_2Cl_2, 300.13 MHz) of **3c**

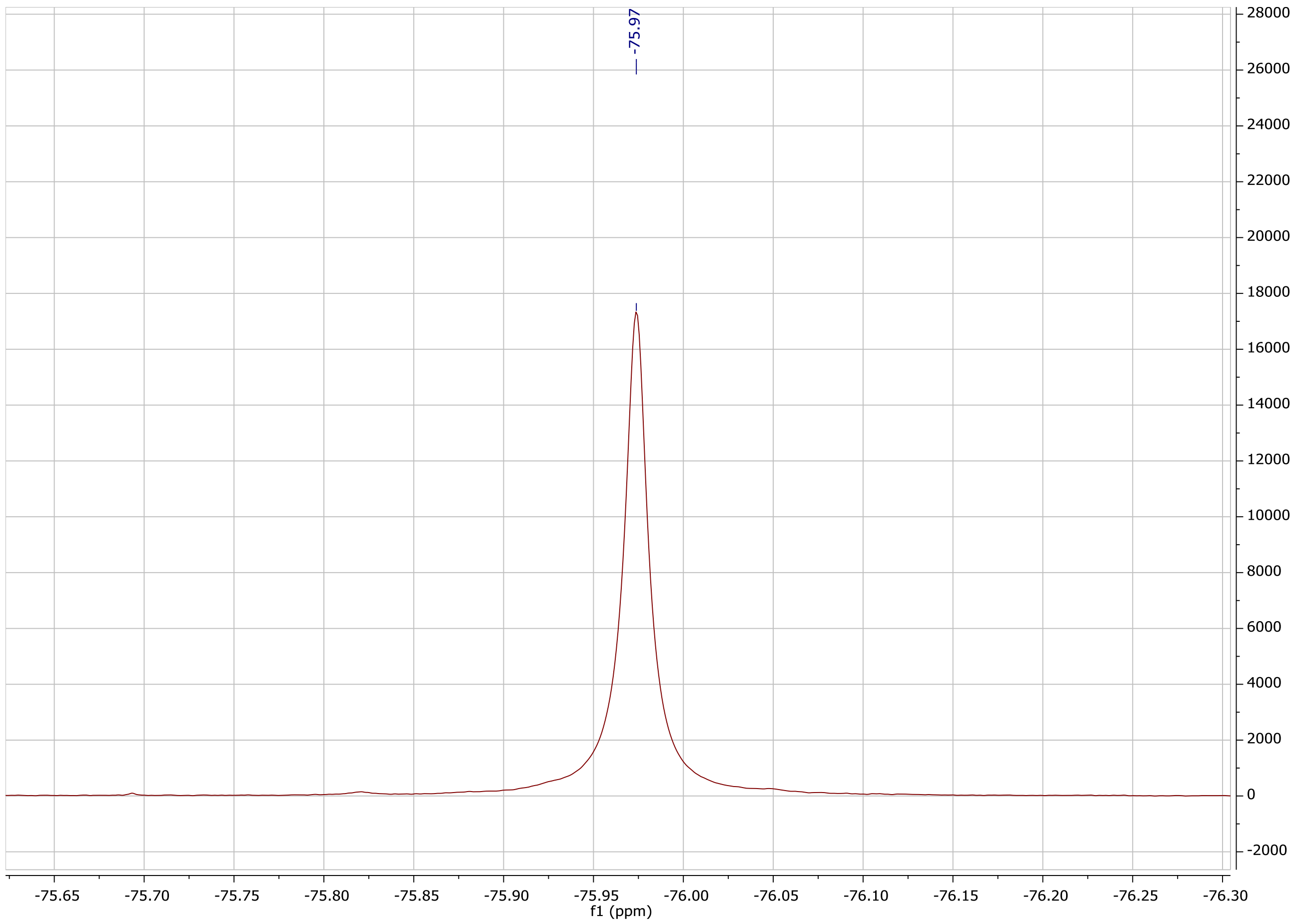

^{19}F-NMR spectrum (CD_2Cl_2, 282.40 MHz) of **3c**

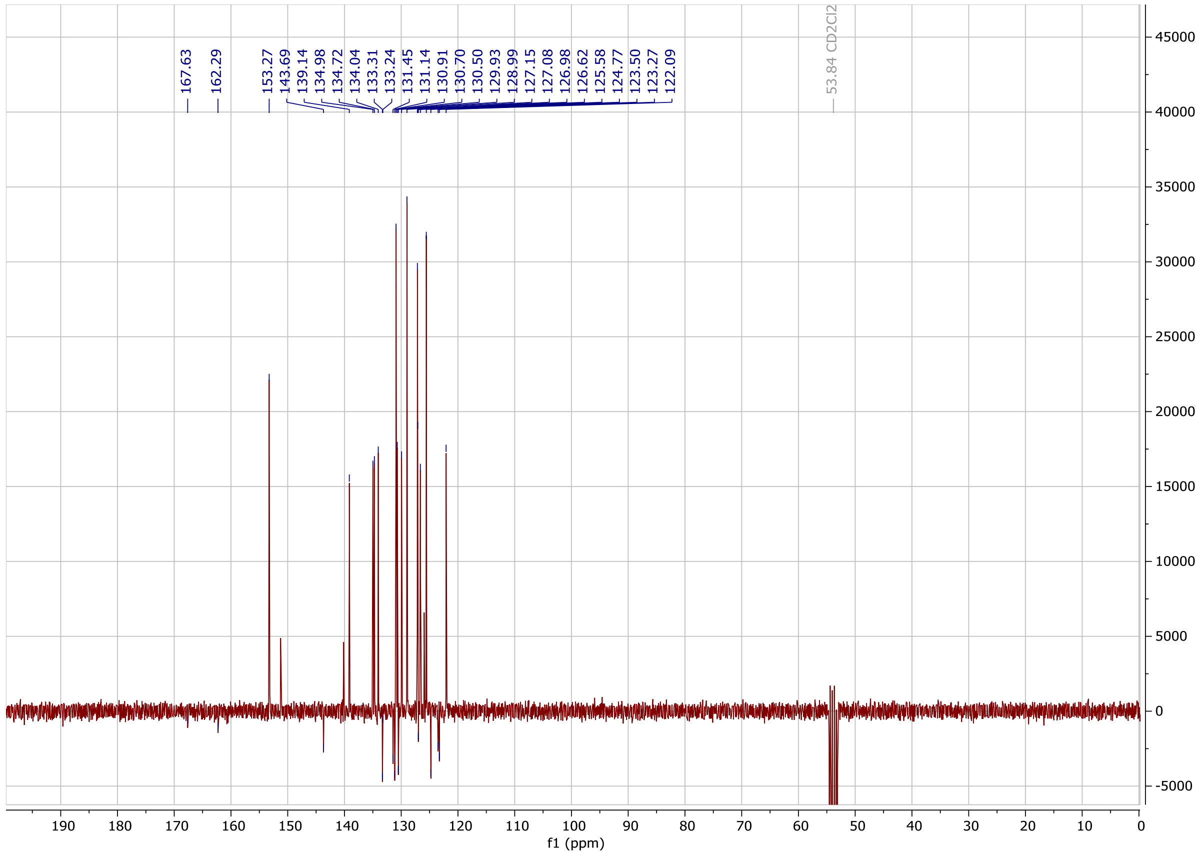

$^{13}C\{^1H\}$ (APT) NMR spectrum (CD_2Cl_2, 75.47 MHz) of **3c**

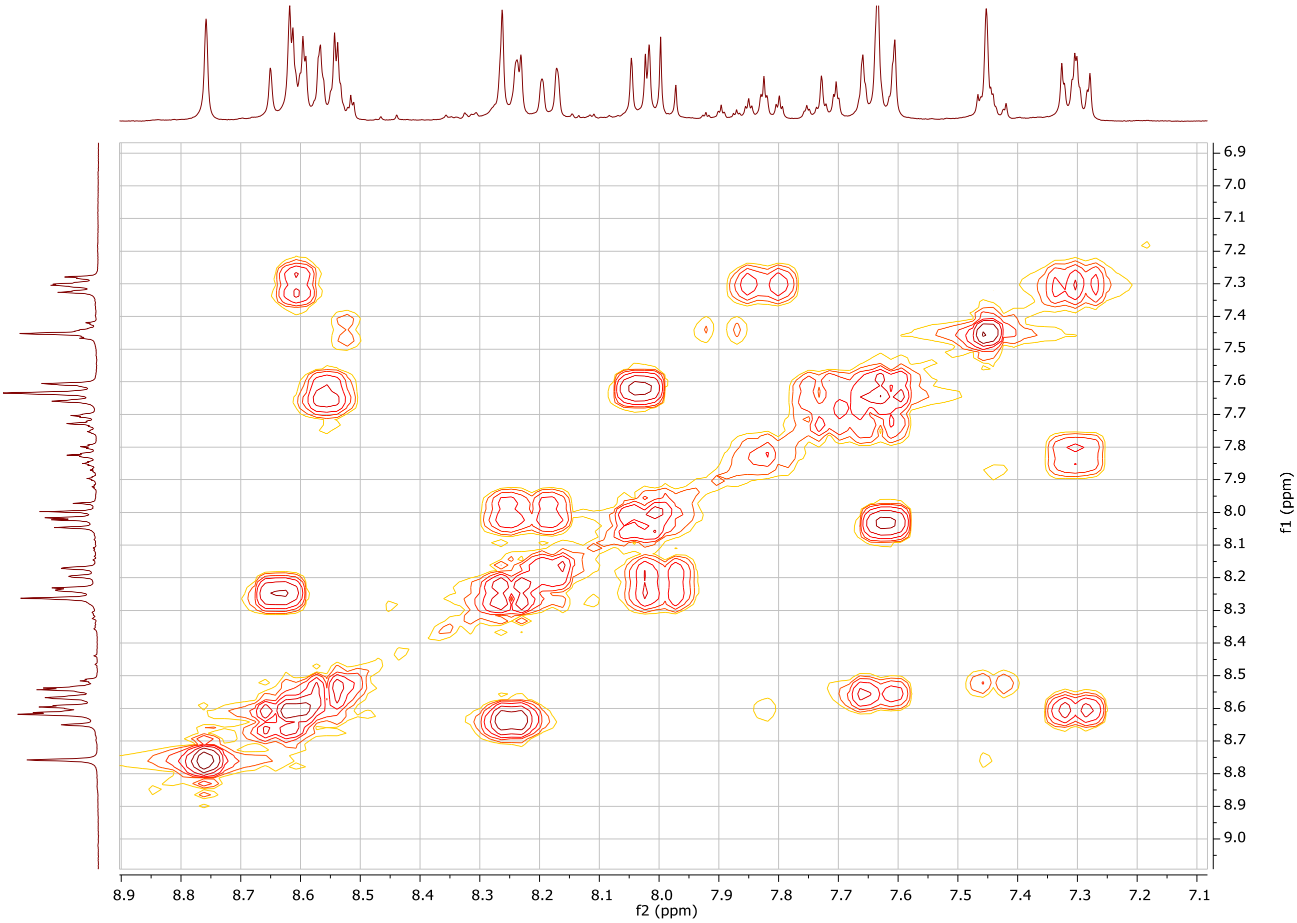

$^1H - ^1H$ COSY correlation spectrum of **3c**

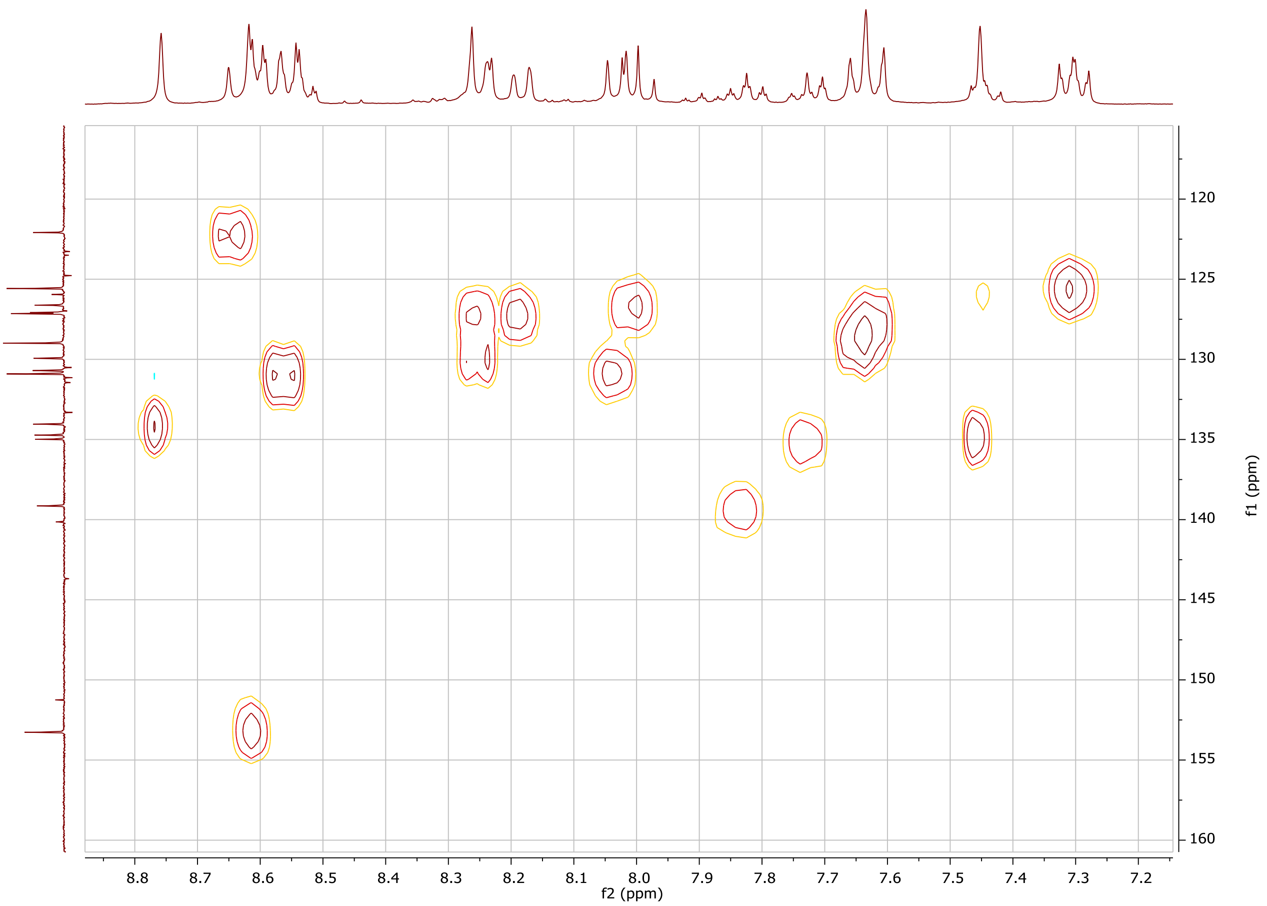

^{1}H - ^{13}C HSQC correlation spectrum of **3c**

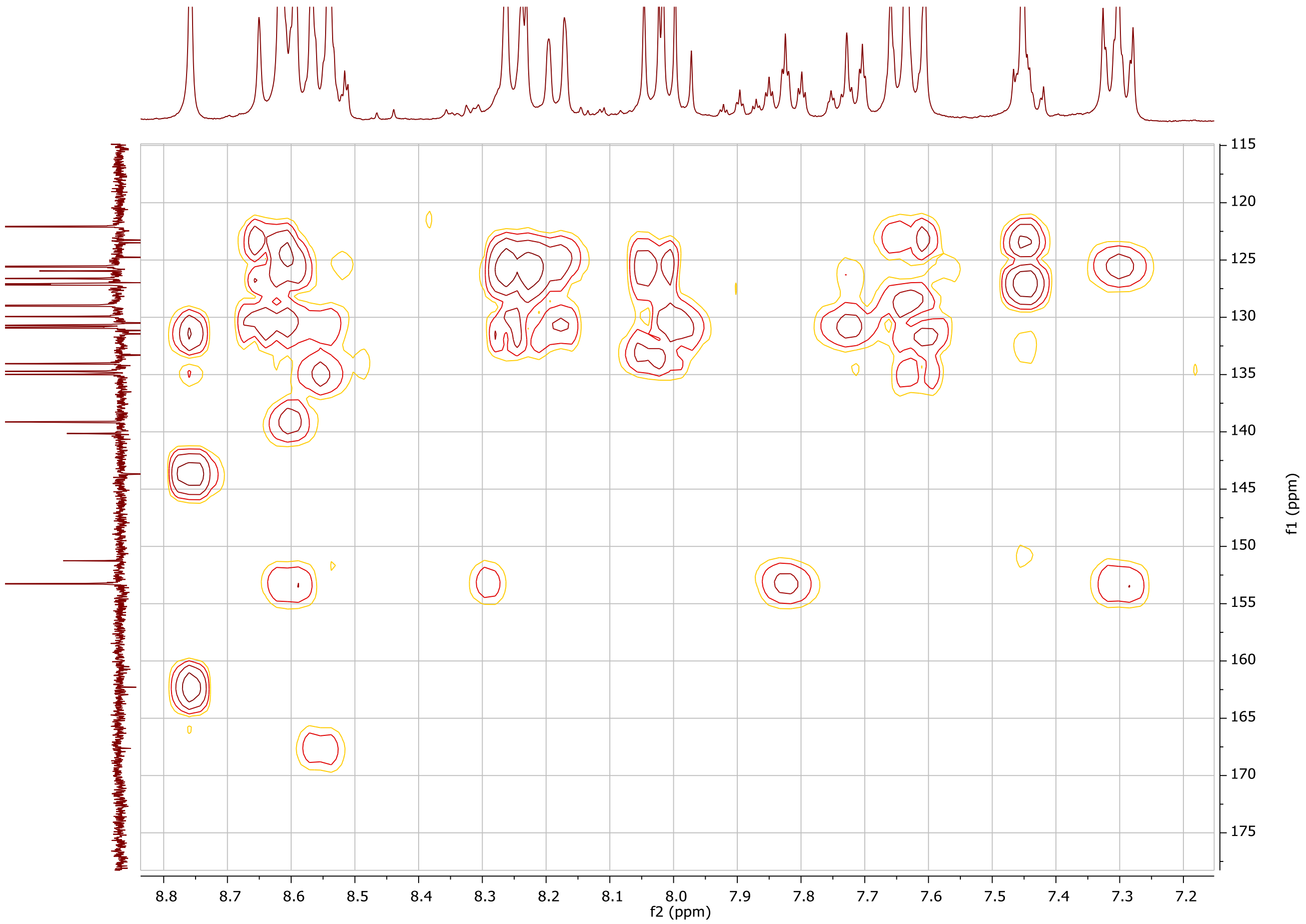

^{1}H - ^{13}C HMBC correlation spectrum of **3c**

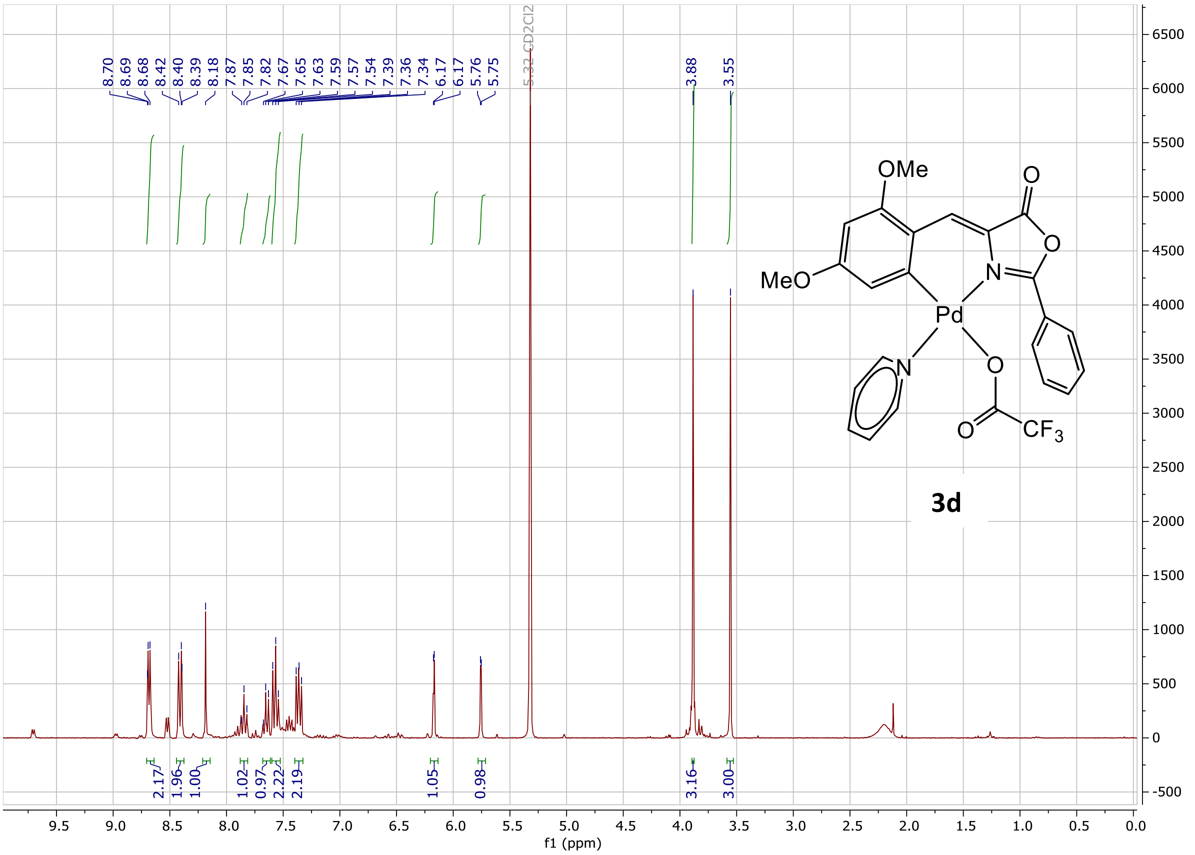

^{1}H-NMR spectrum (CD_2Cl_2, 300.13 MHz) of **3d**

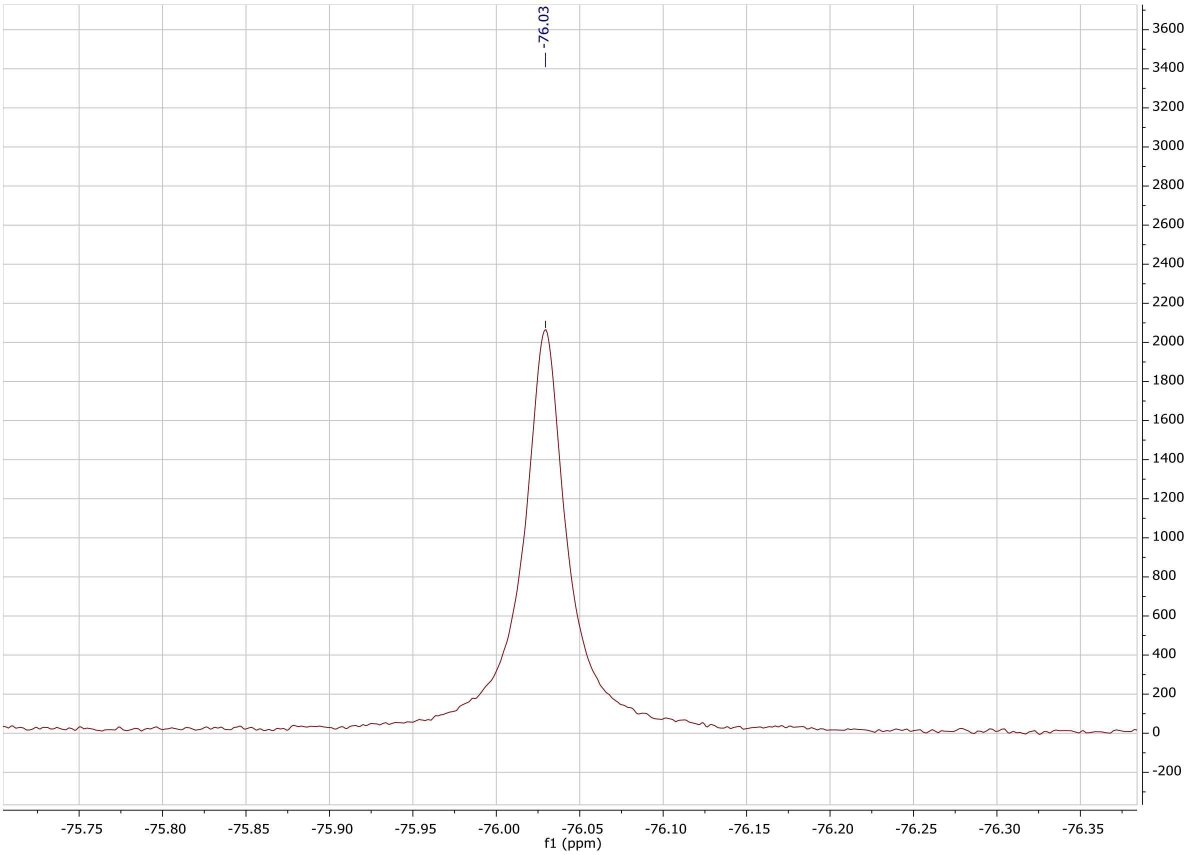

^{19}F-NMR spectrum (CD_2Cl_2, 282.40 MHz) of **3d**

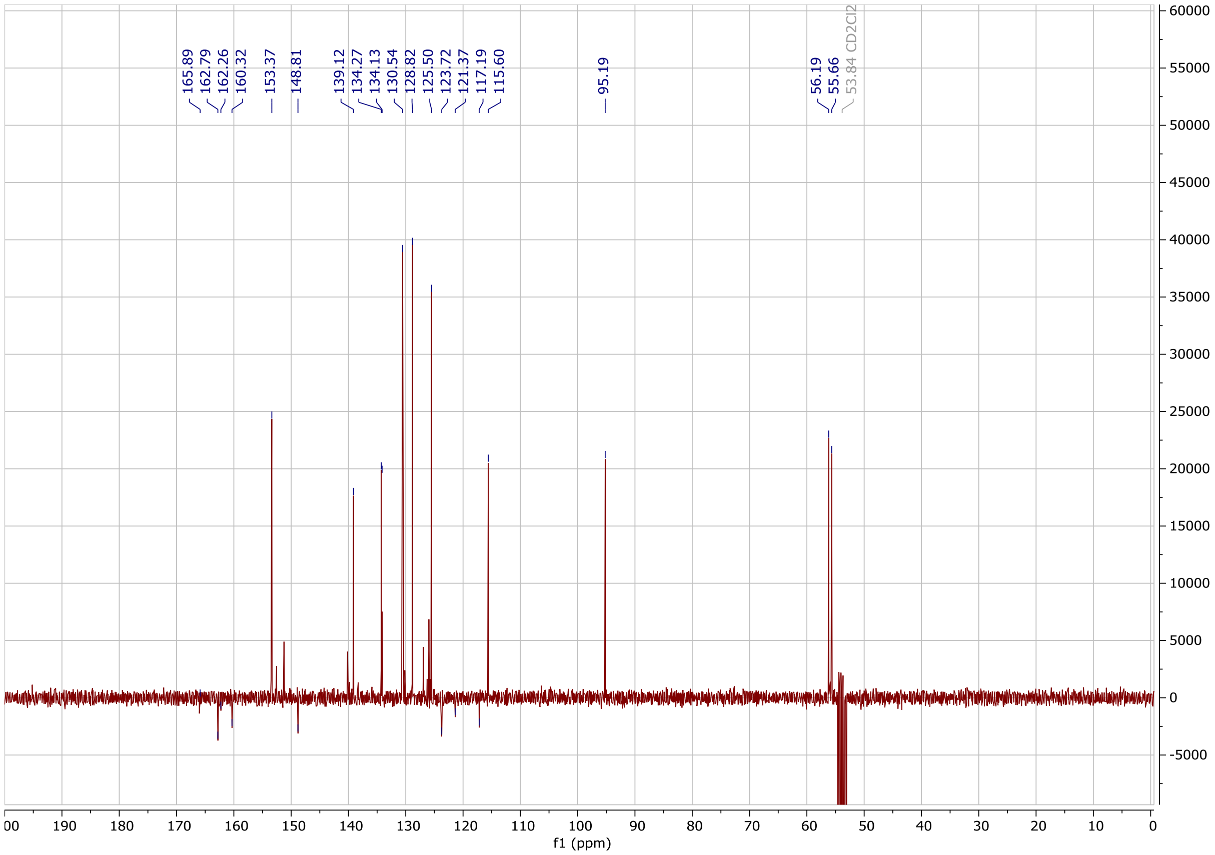

$^{13}C\{^{1}H\}$ (APT) NMR spectrum (CD_2Cl_2, 75.47 MHz) of **3d**

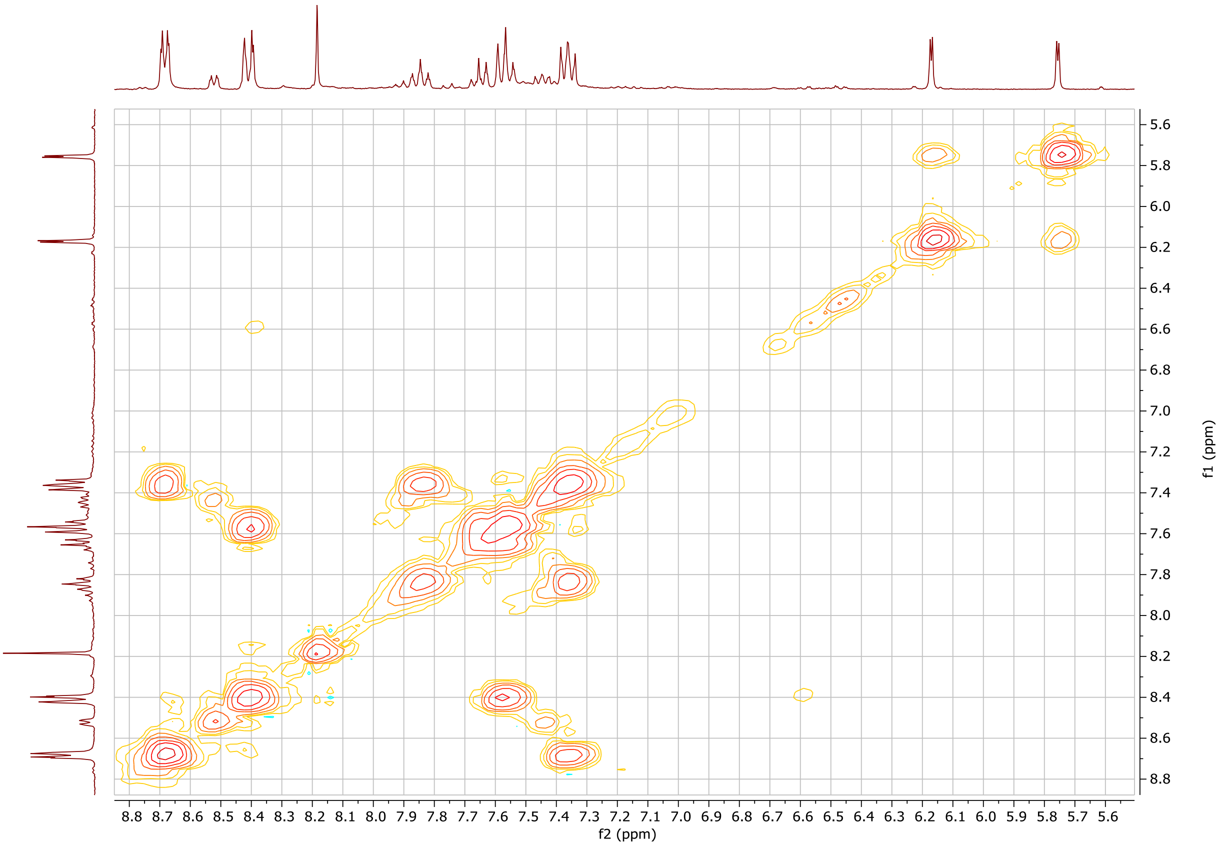

$^{1}H-^{1}H$ COSY correlation spectrum of **3d**

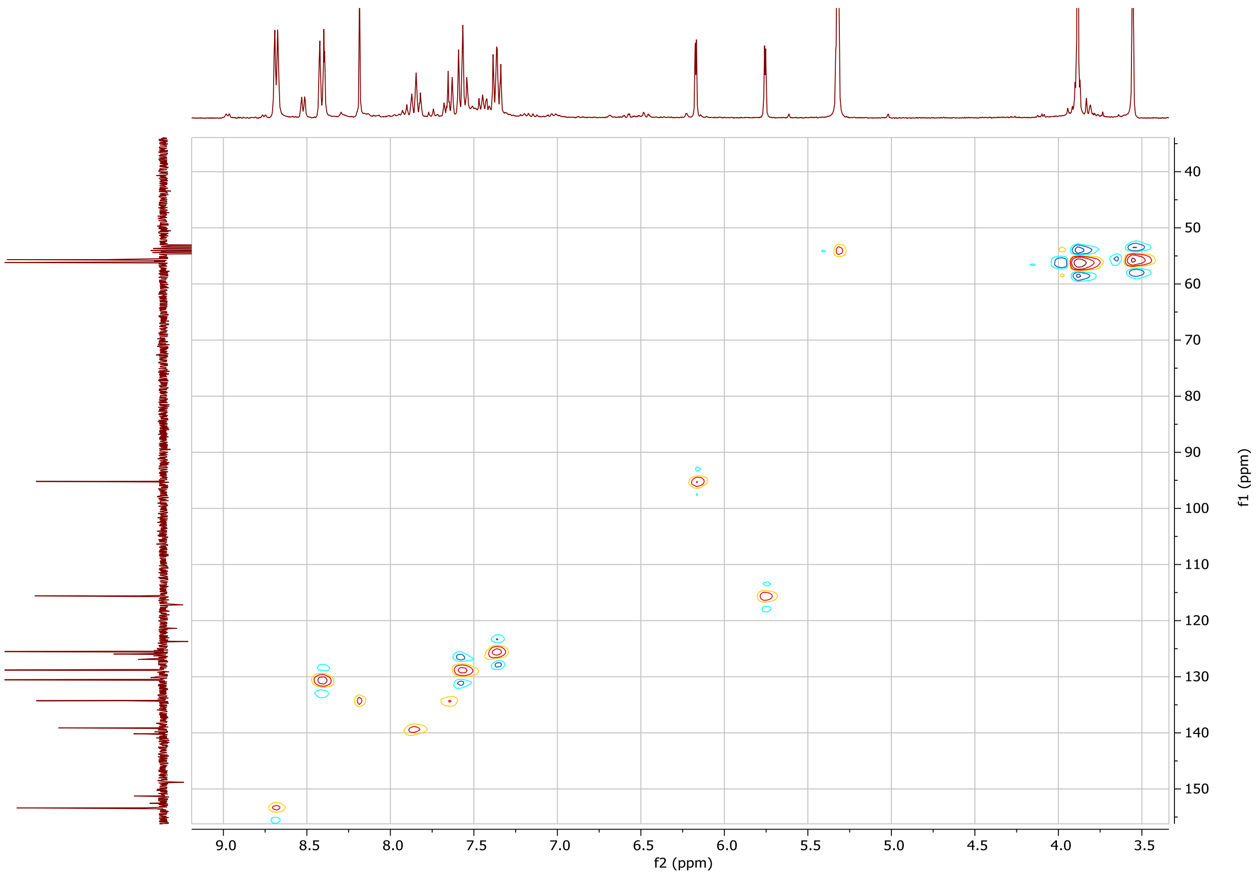

^1H - ^{13}C HSQC correlation spectrum of **3d**

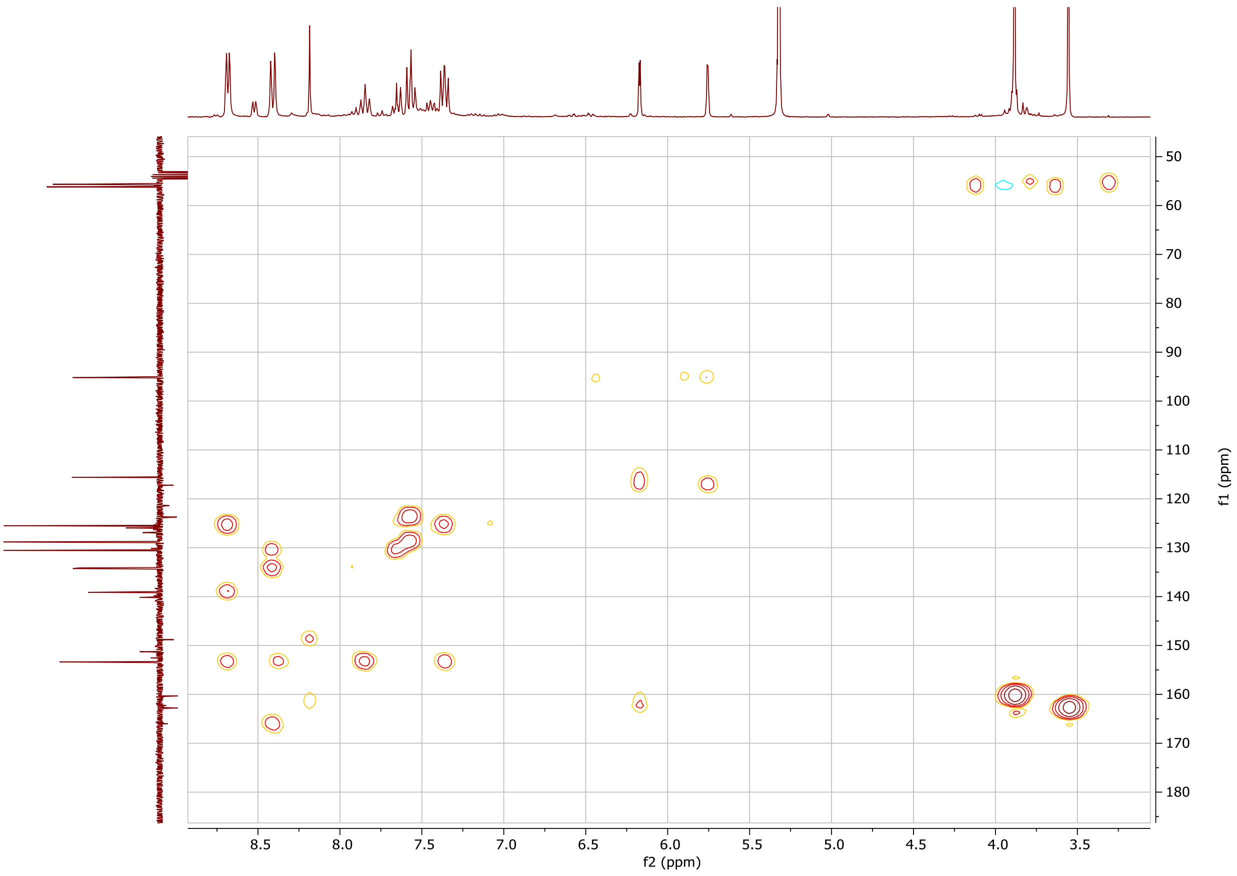

^1H - ^{13}C HMBC correlation spectrum of **3d**

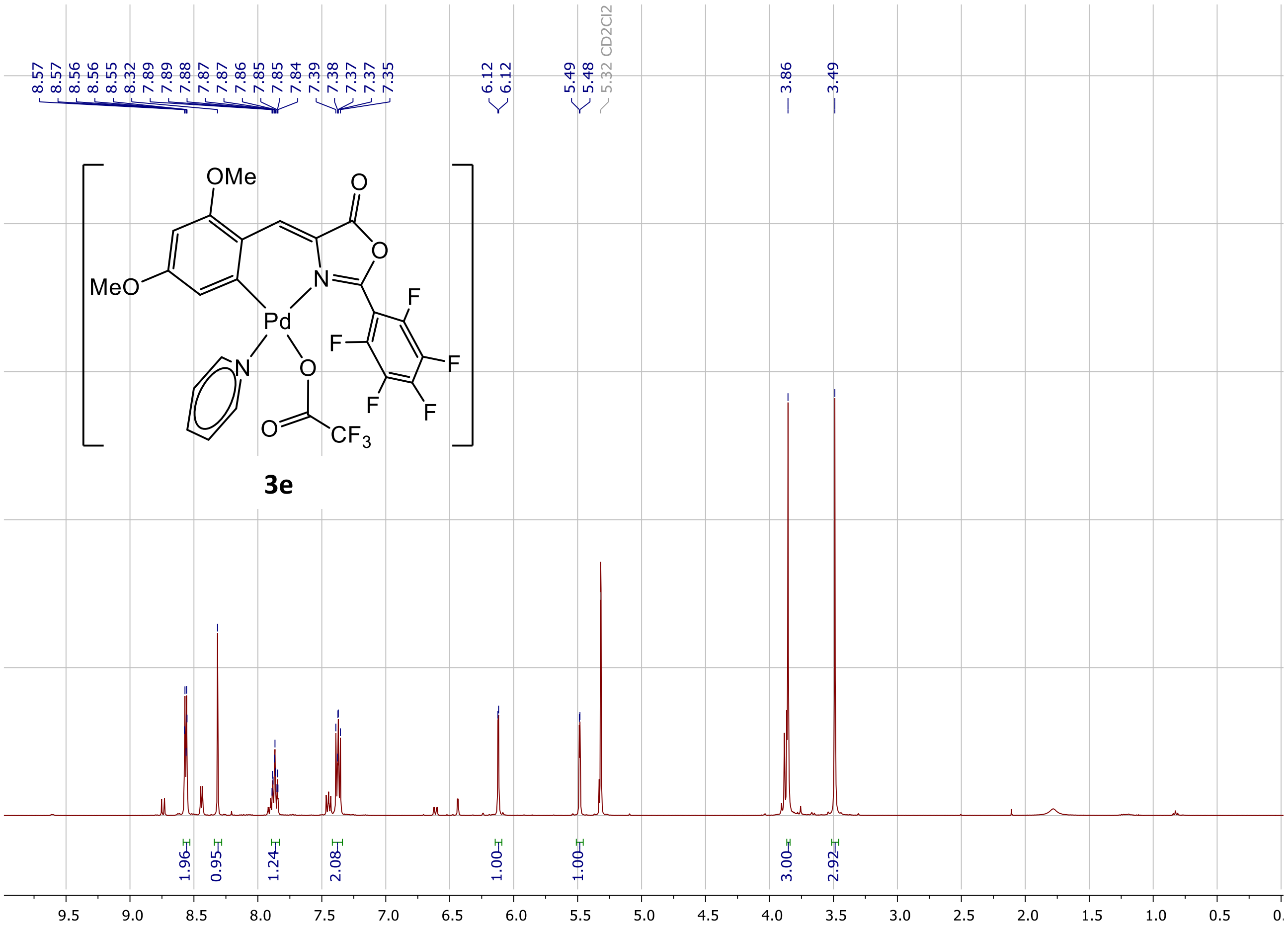

^{1}H-NMR spectrum (CD_2Cl_2, 400.13 MHz, 233 K) of **3e**

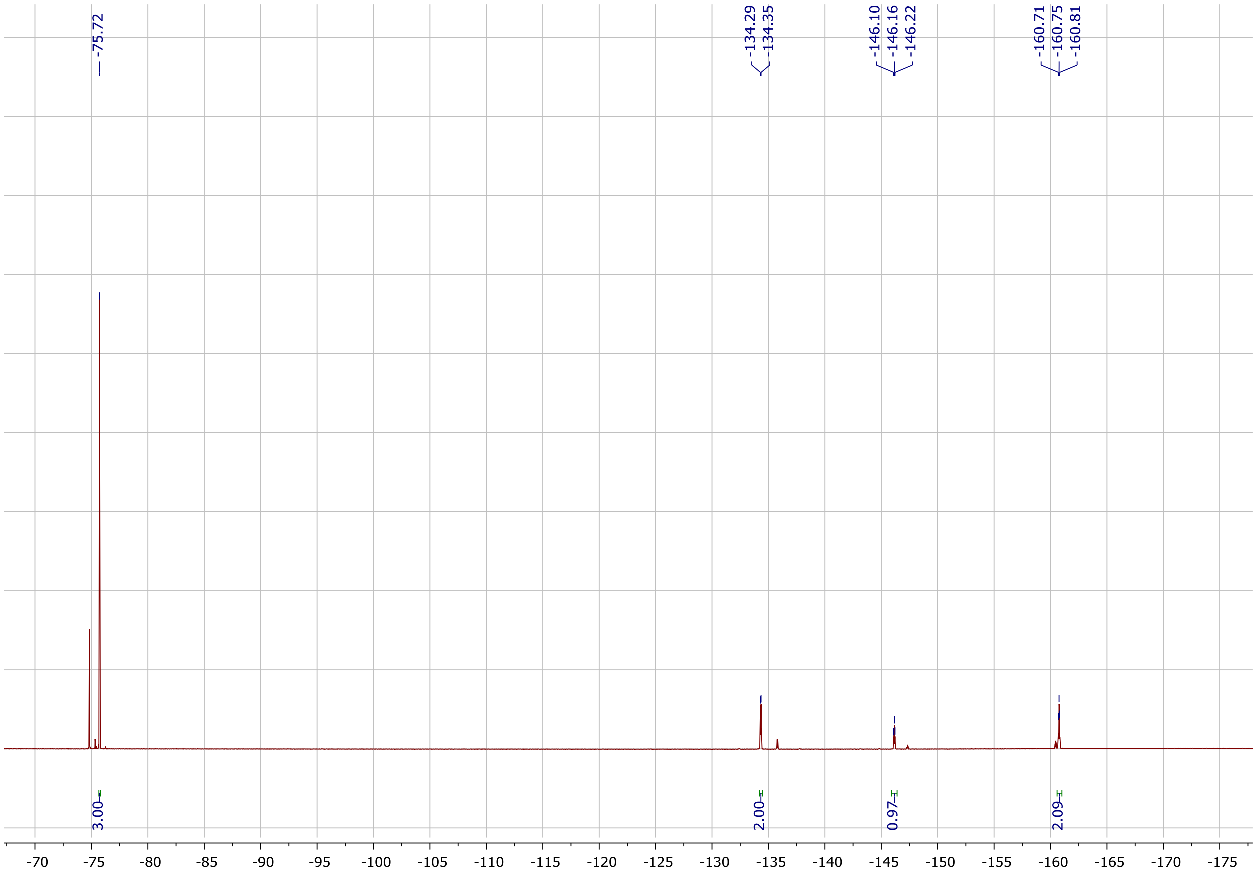

^{19}F-NMR spectrum (CD_2Cl_2, 376.49 MHz, 233 K) of **3e**

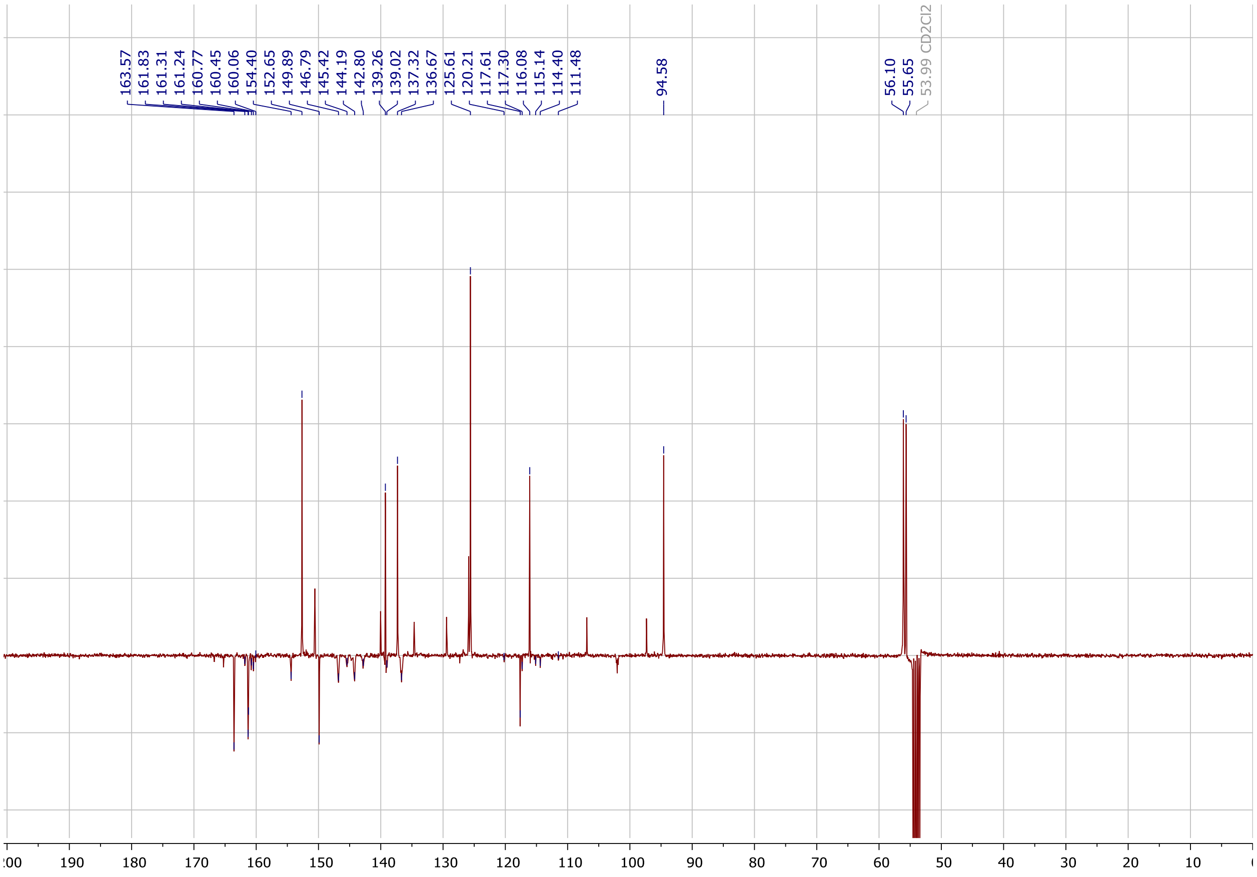

$^{13}C\{^{1}H\}$ (APT) NMR spectrum (CD_2Cl_2, 100.61 MHz, 233 K) of **3e**

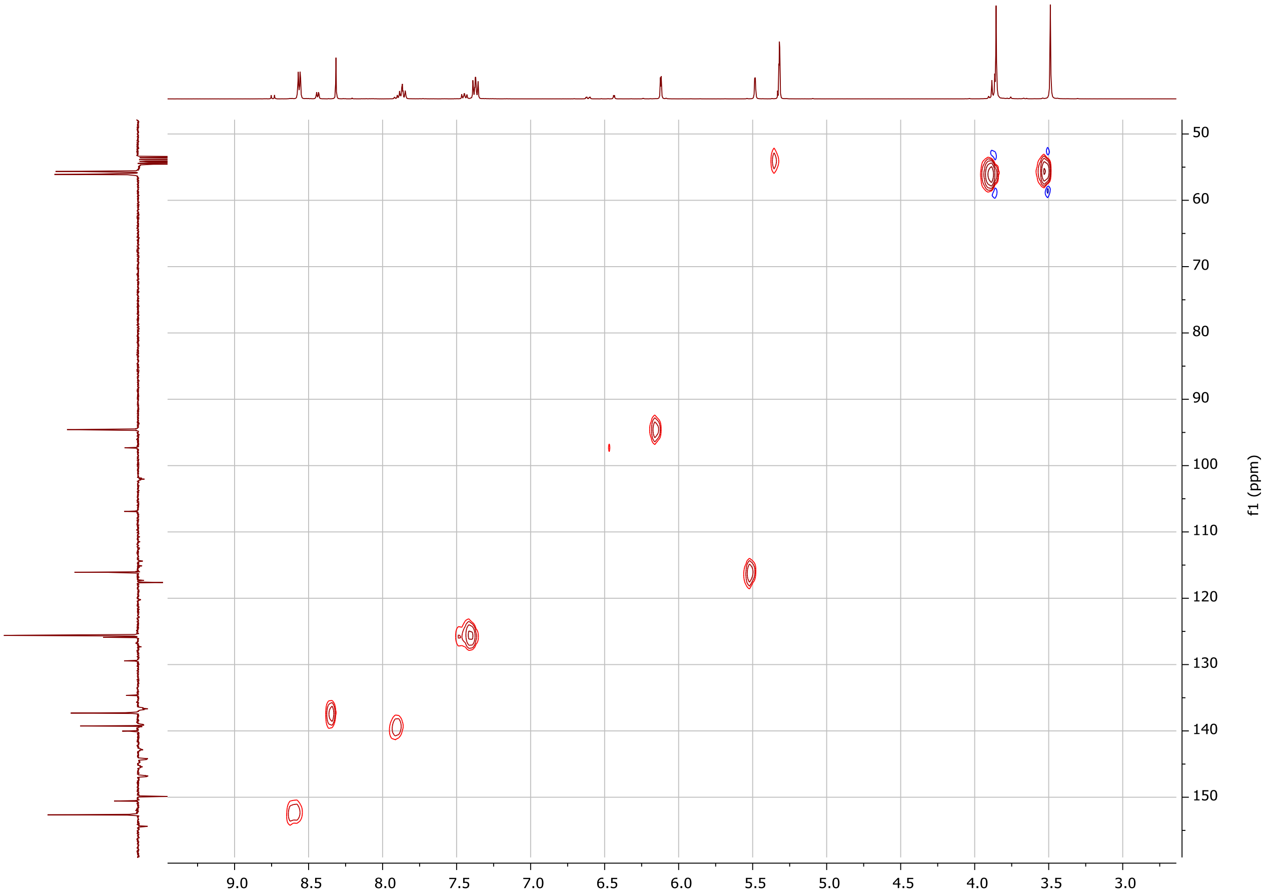

^{1}H - ^{13}C HSQC correlation spectrum of **3e**

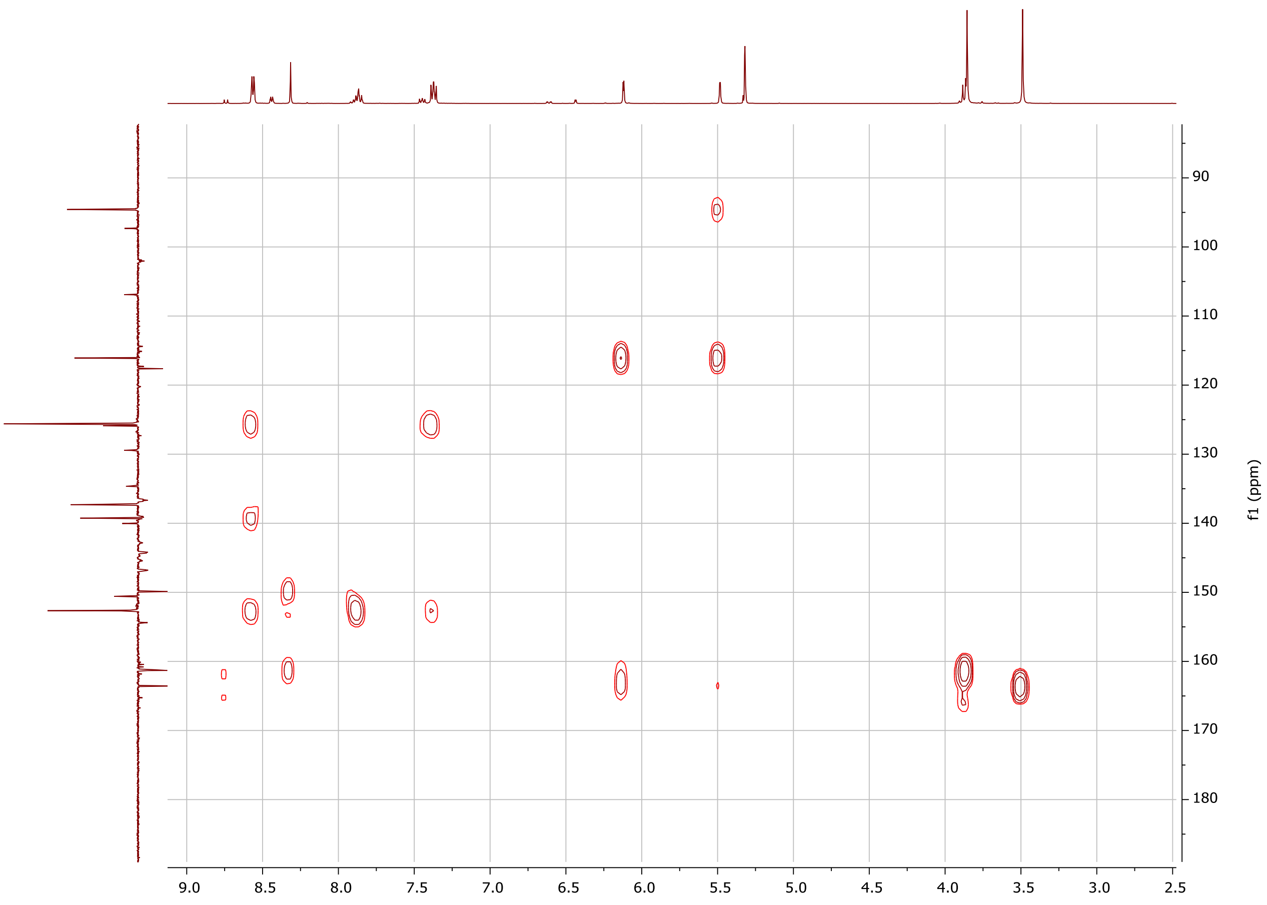

^{1}H - ^{13}C HMBC correlation spectrum of **3e**

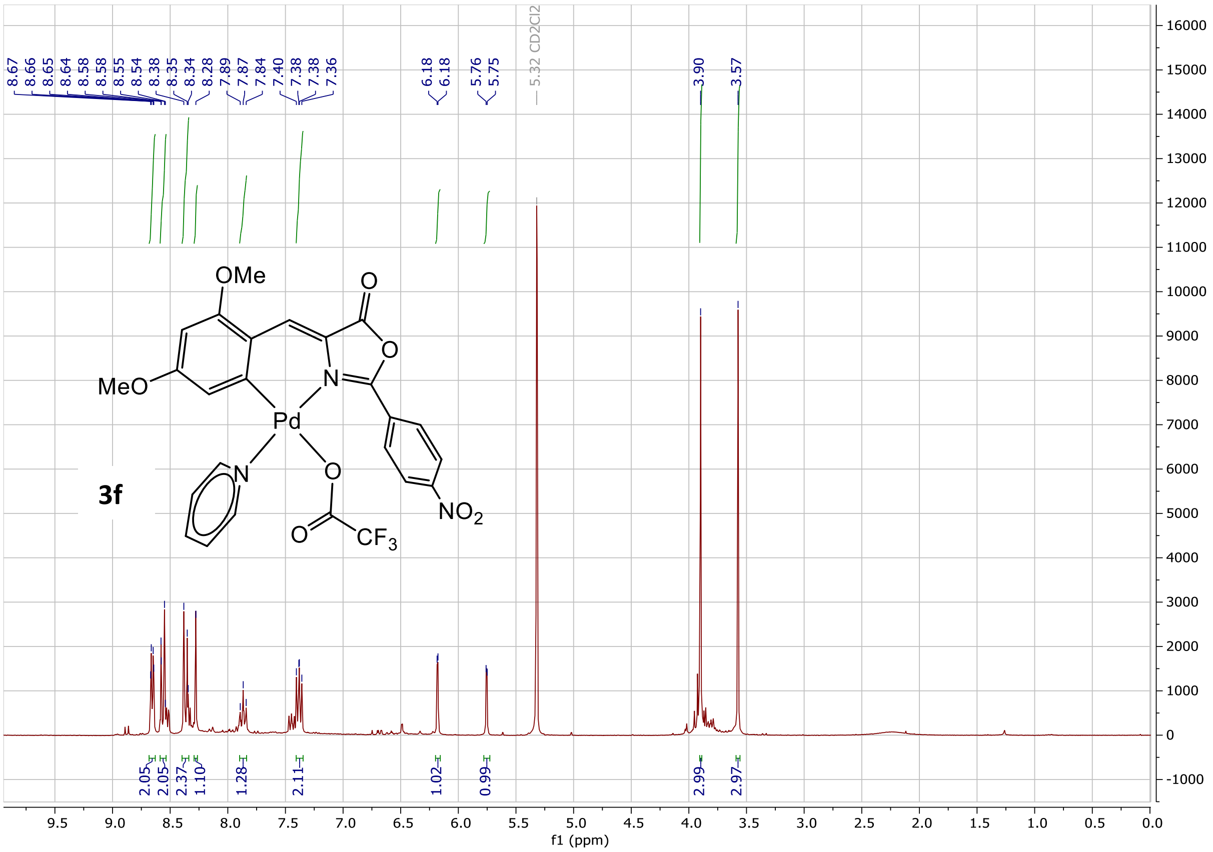

^{1}H-NMR spectrum (CD_2Cl_2, 300.13 MHz) of **3f**

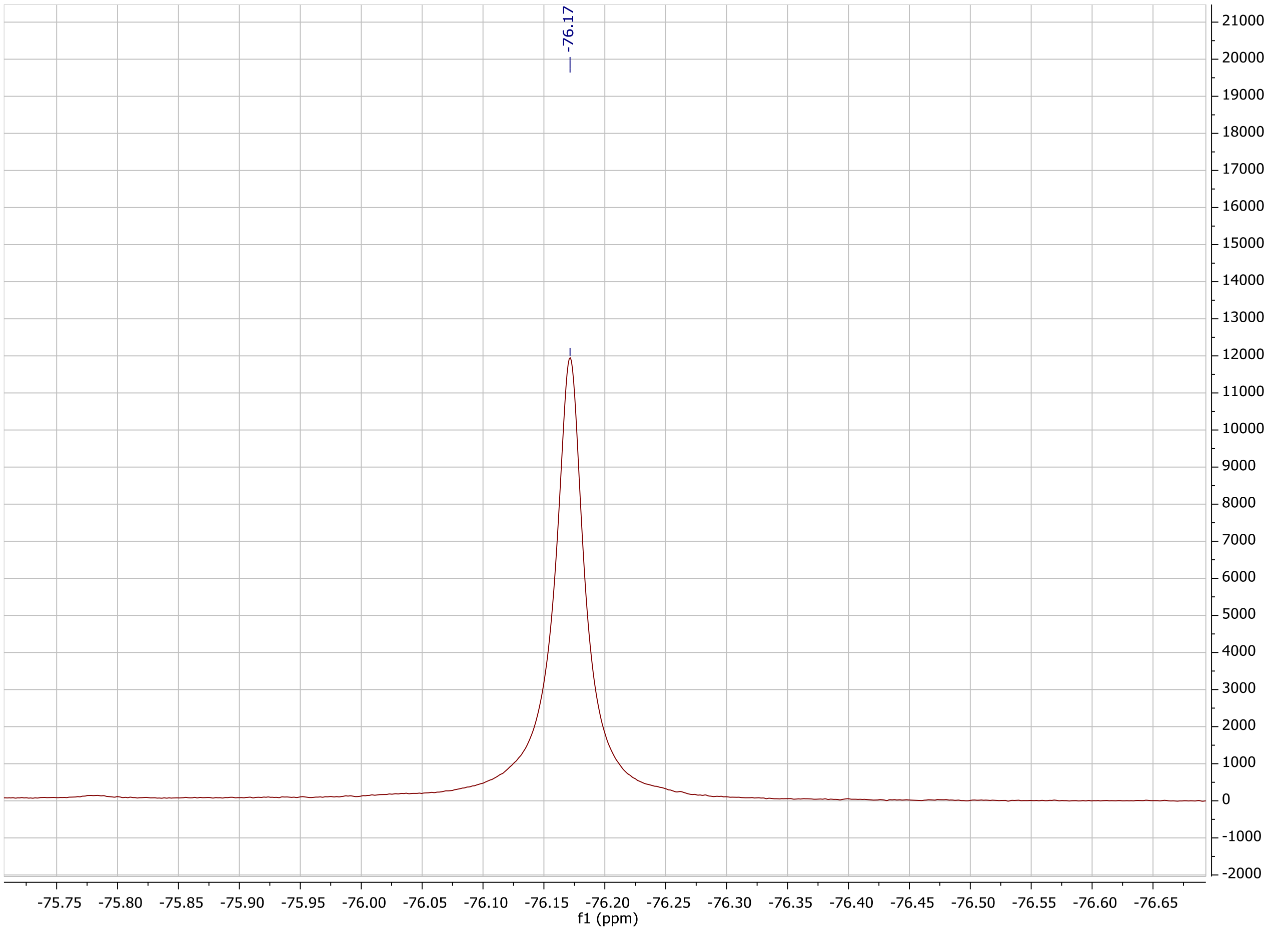

^{19}F-NMR spectrum (CD_2Cl_2, 282.40 MHz) of **3f**

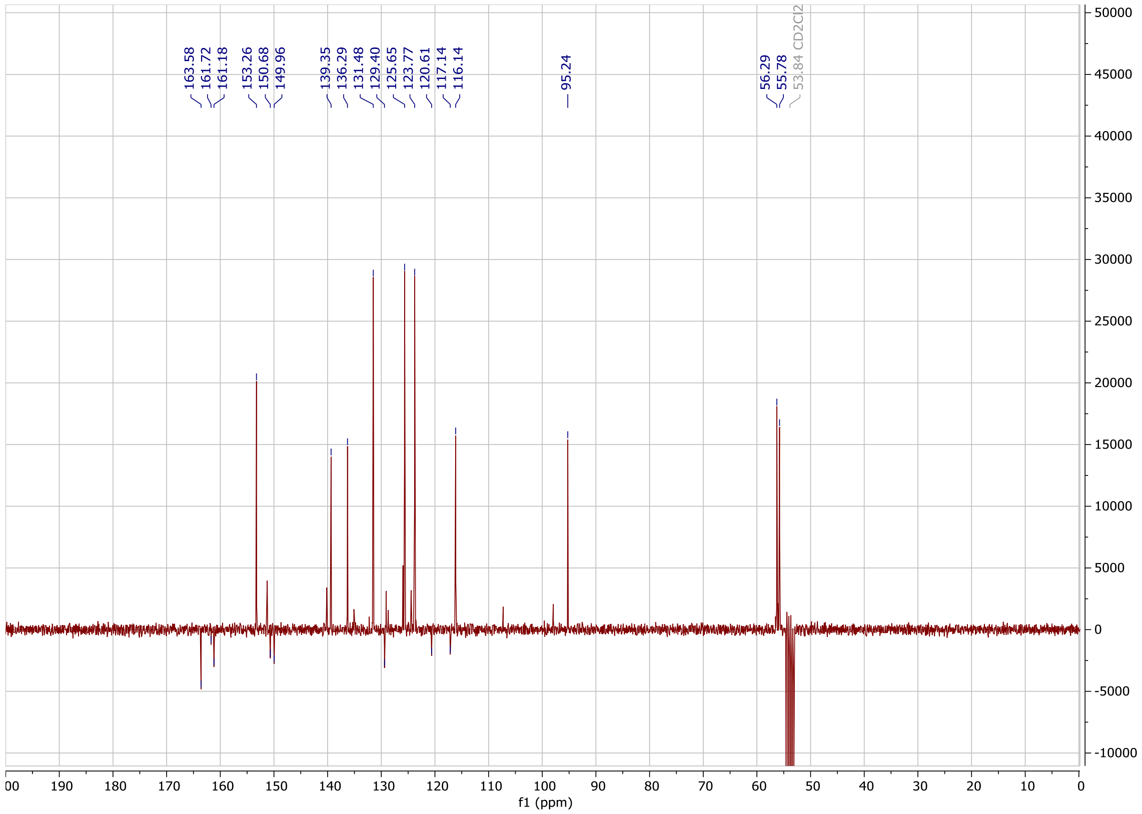

^{13}C{^{1}H} (APT) NMR spectrum (CD_2Cl_2, 75.47 MHz) of **3f**

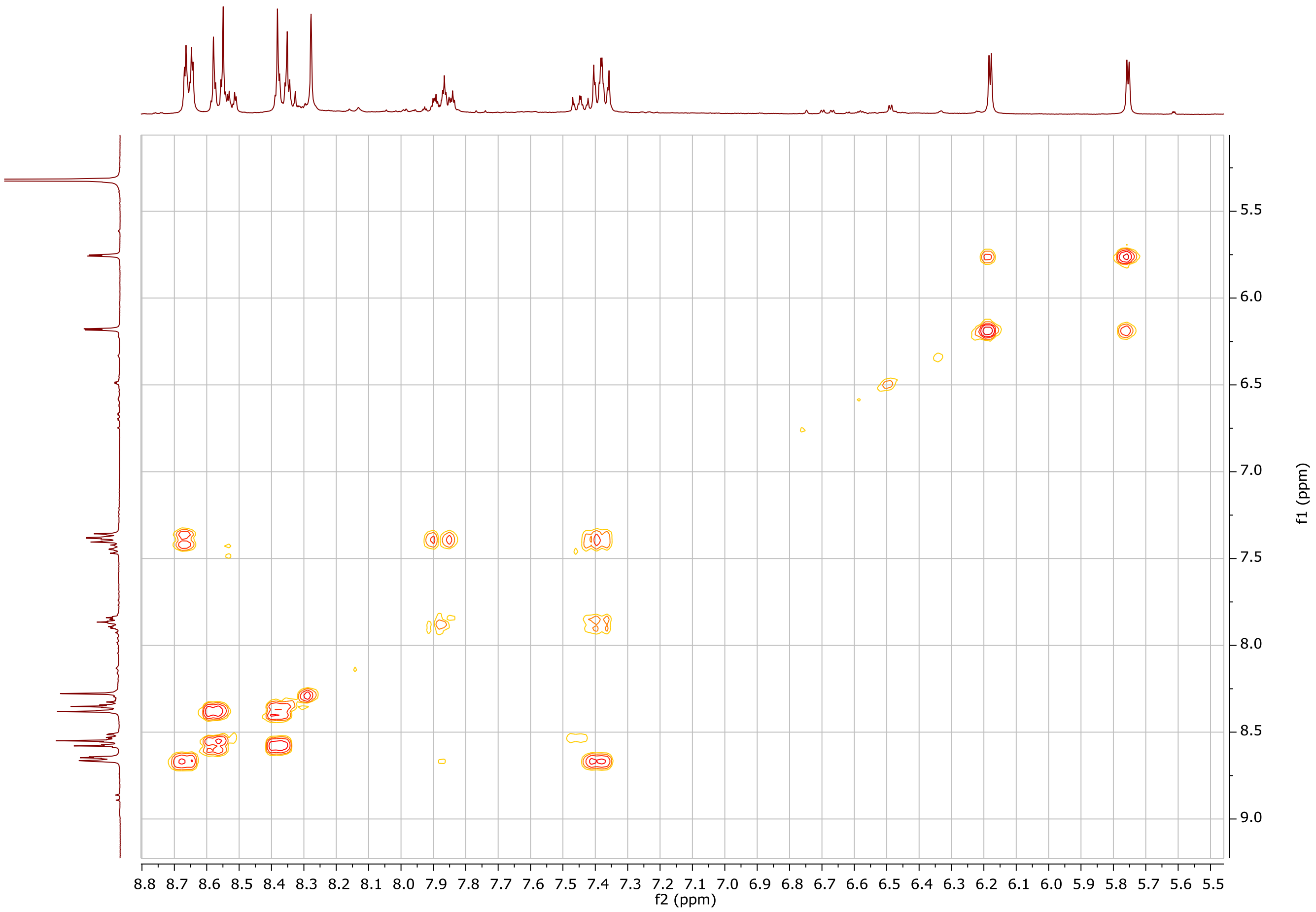

$^{1}H - ^{1}H$ COSY correlation spectrum of **3f** (aromatic region)

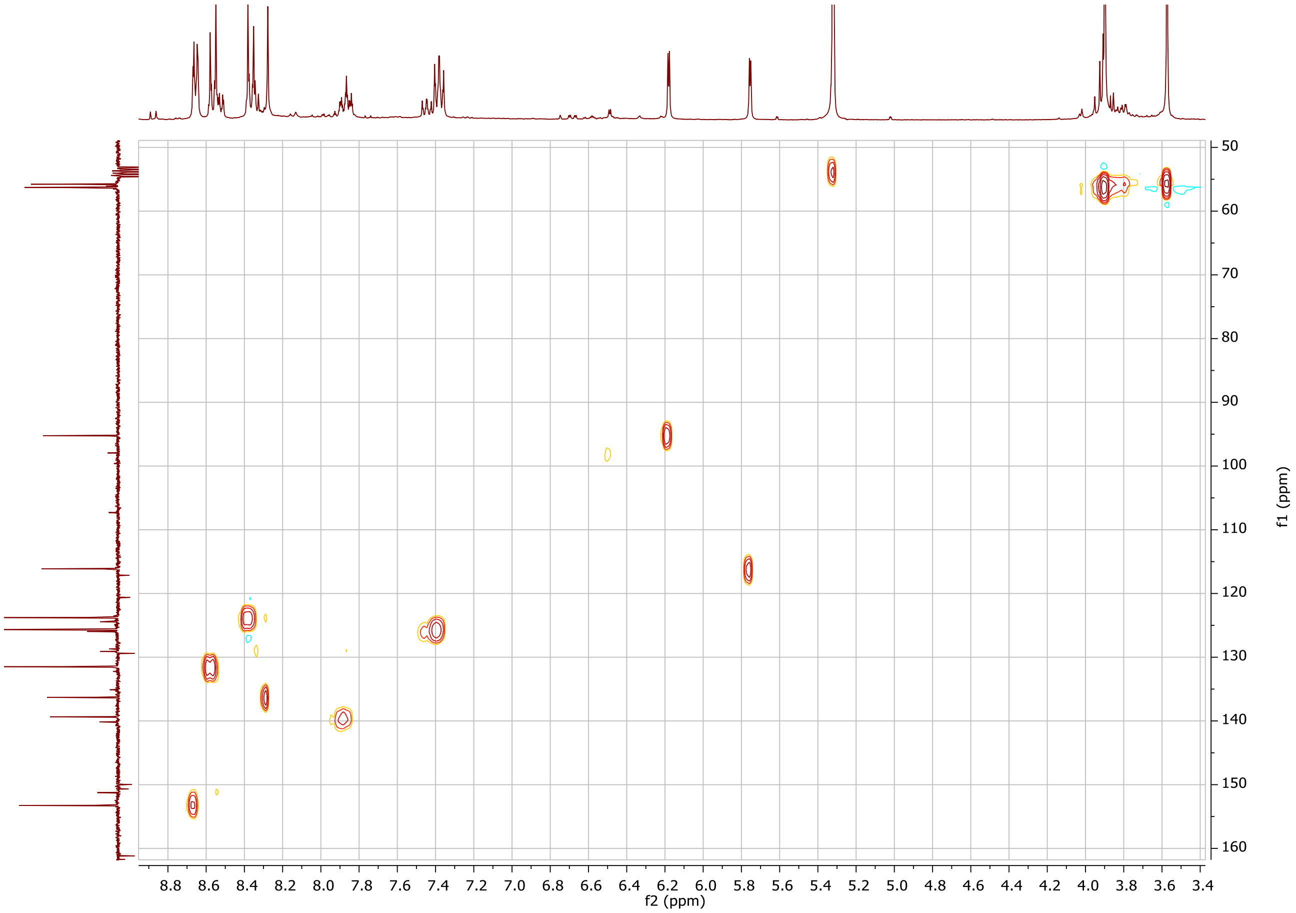

^{1}H - ^{13}C HSQC correlation spectrum of **3f**

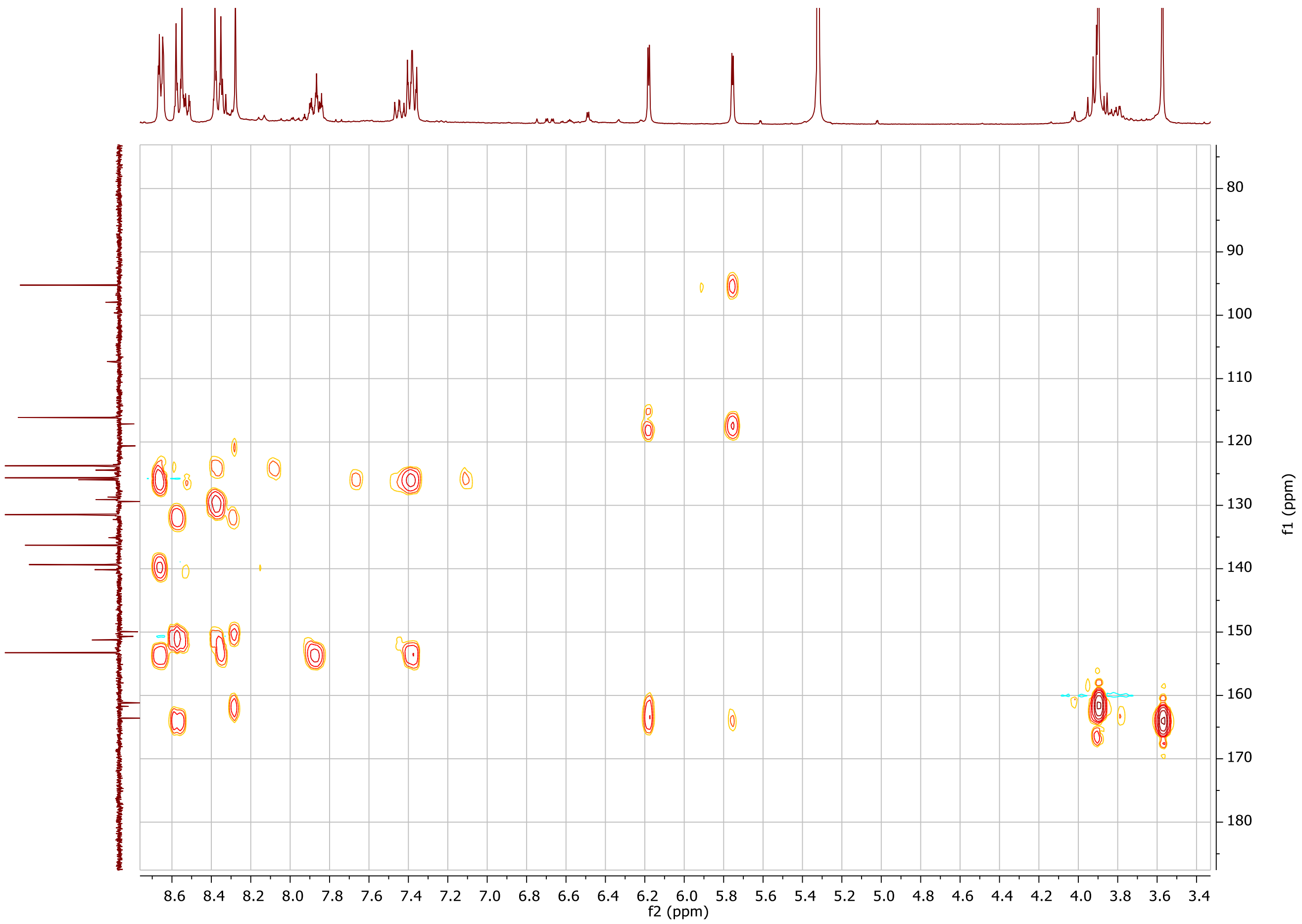

^{1}H - ^{13}C HMBC correlation spectrum of **3f**

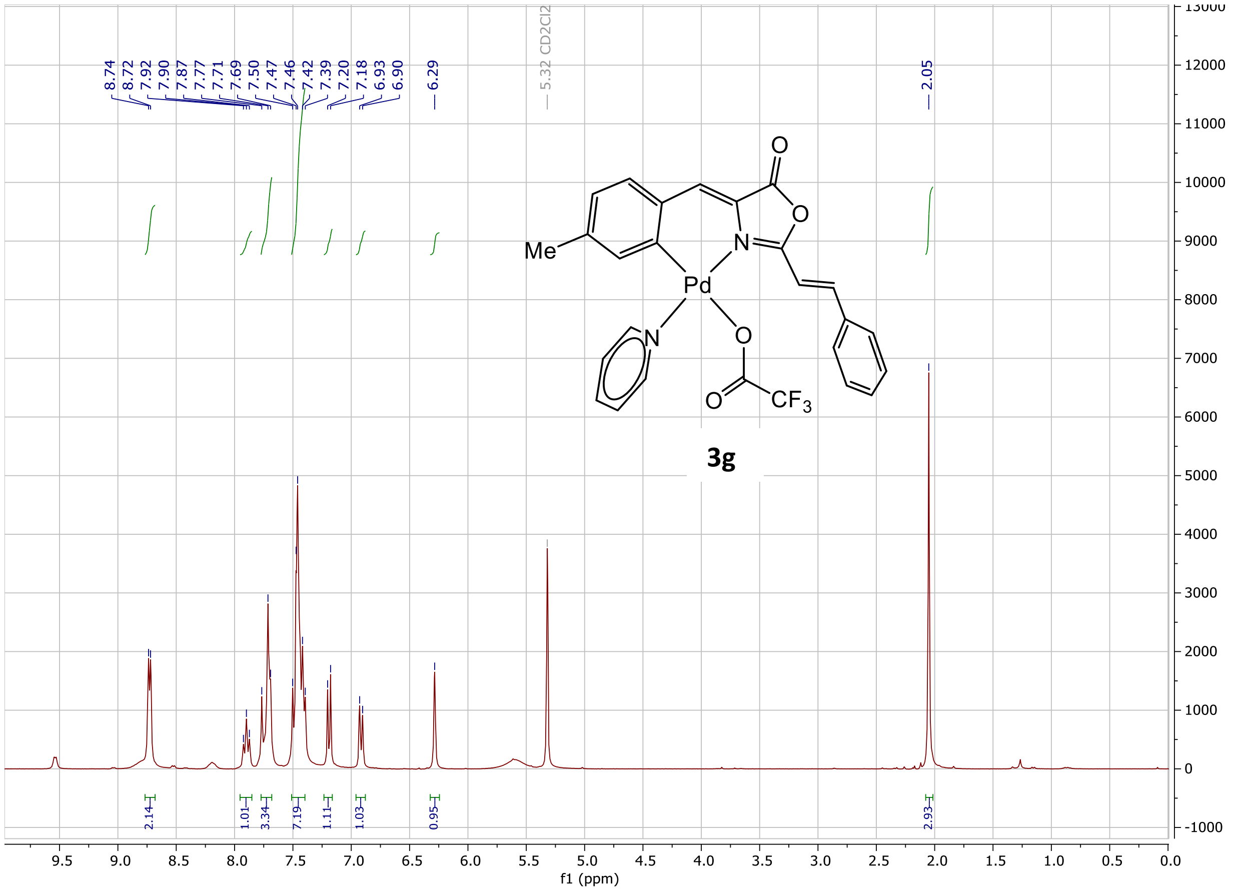

^{1}H-NMR spectrum (CD_2Cl_2, 300.13 MHz) of **3g**

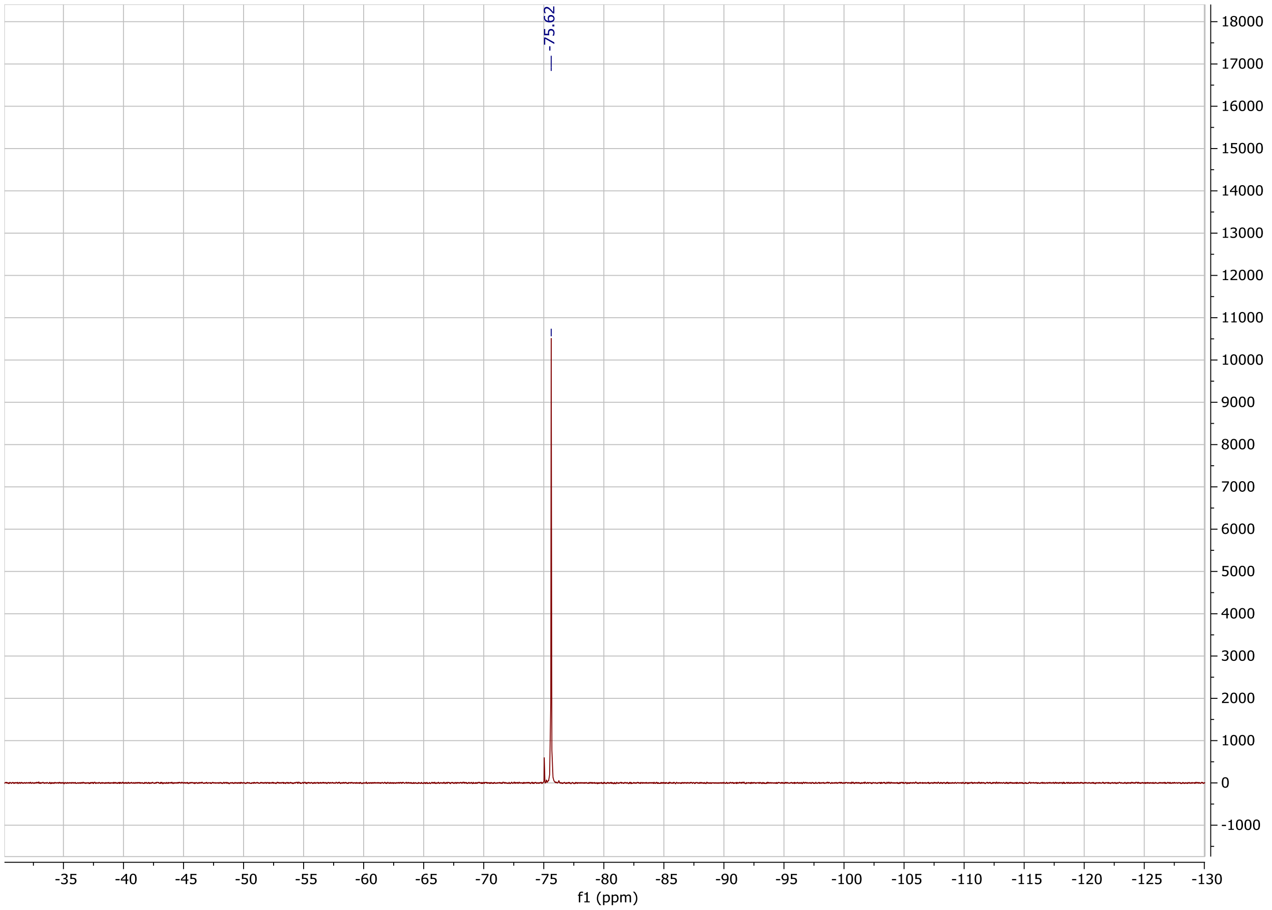

^{19}F-NMR spectrum (CD_2Cl_2, 282.40 MHz) of **3g**

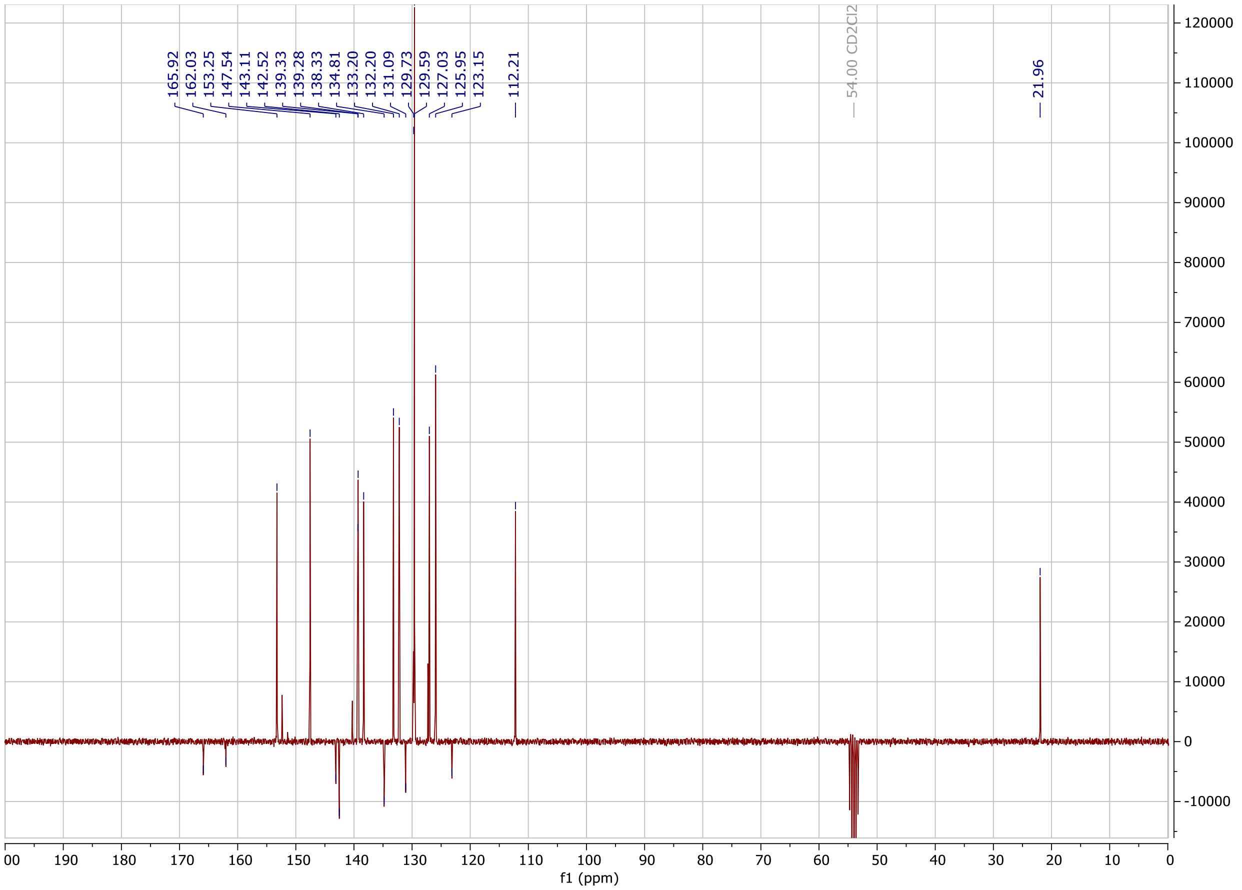

$^{13}C\{^1H\}$ (APT) NMR spectrum (CD_2Cl_2, 75.47 MHz) of **3g**

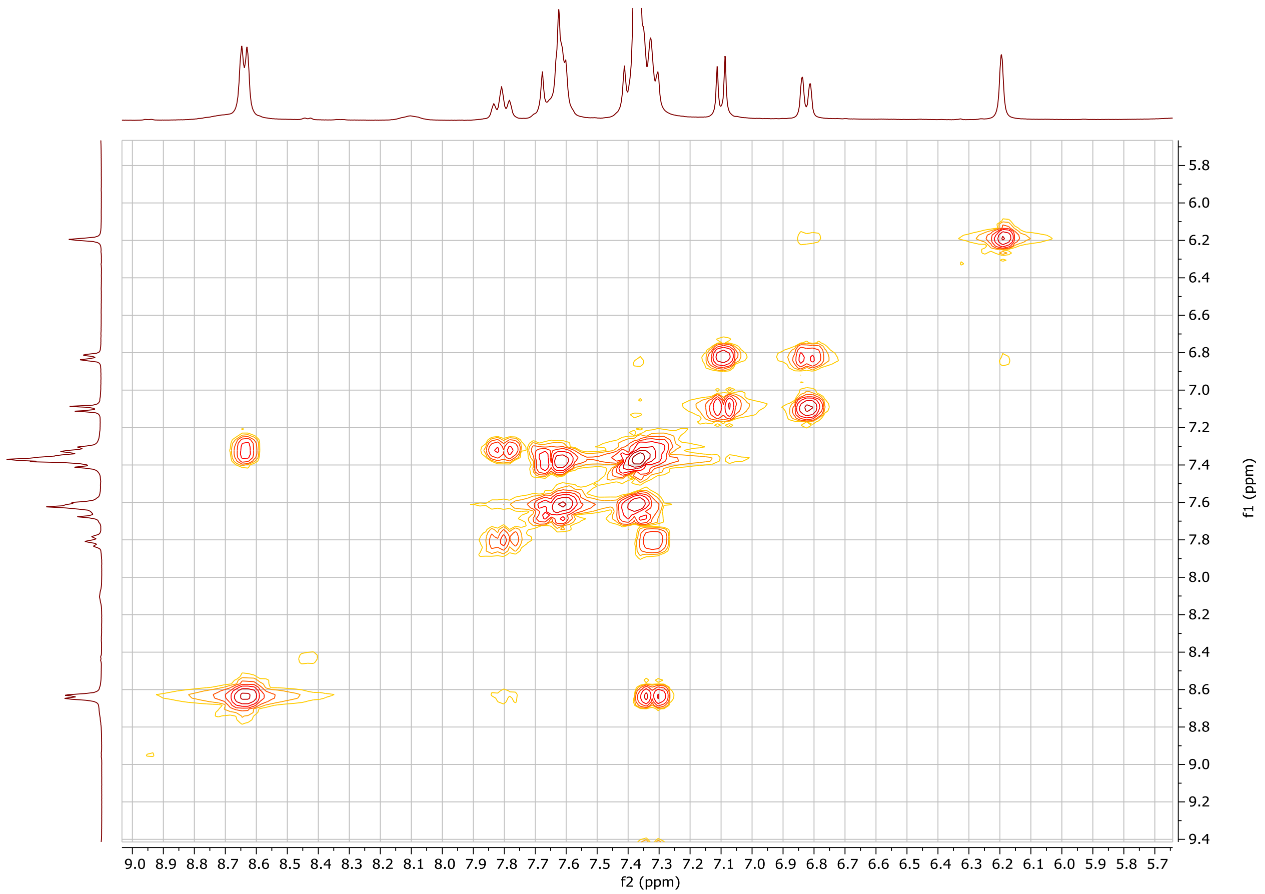

^{1}H – ^{1}H COSY correlation spectrum of **3g** (aromatic region)

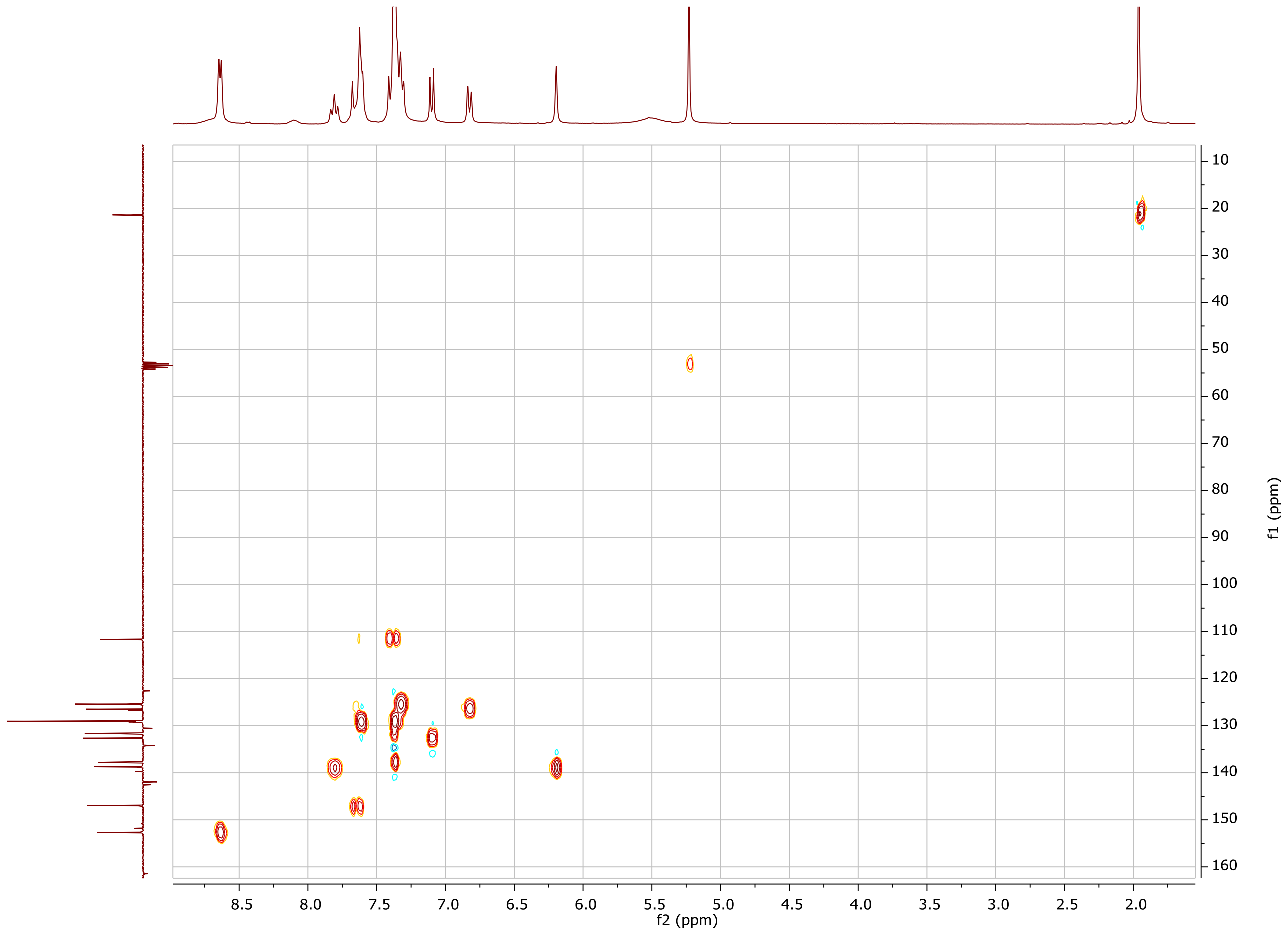

^{1}H - ^{13}C HSQC correlation spectrum of **3g**

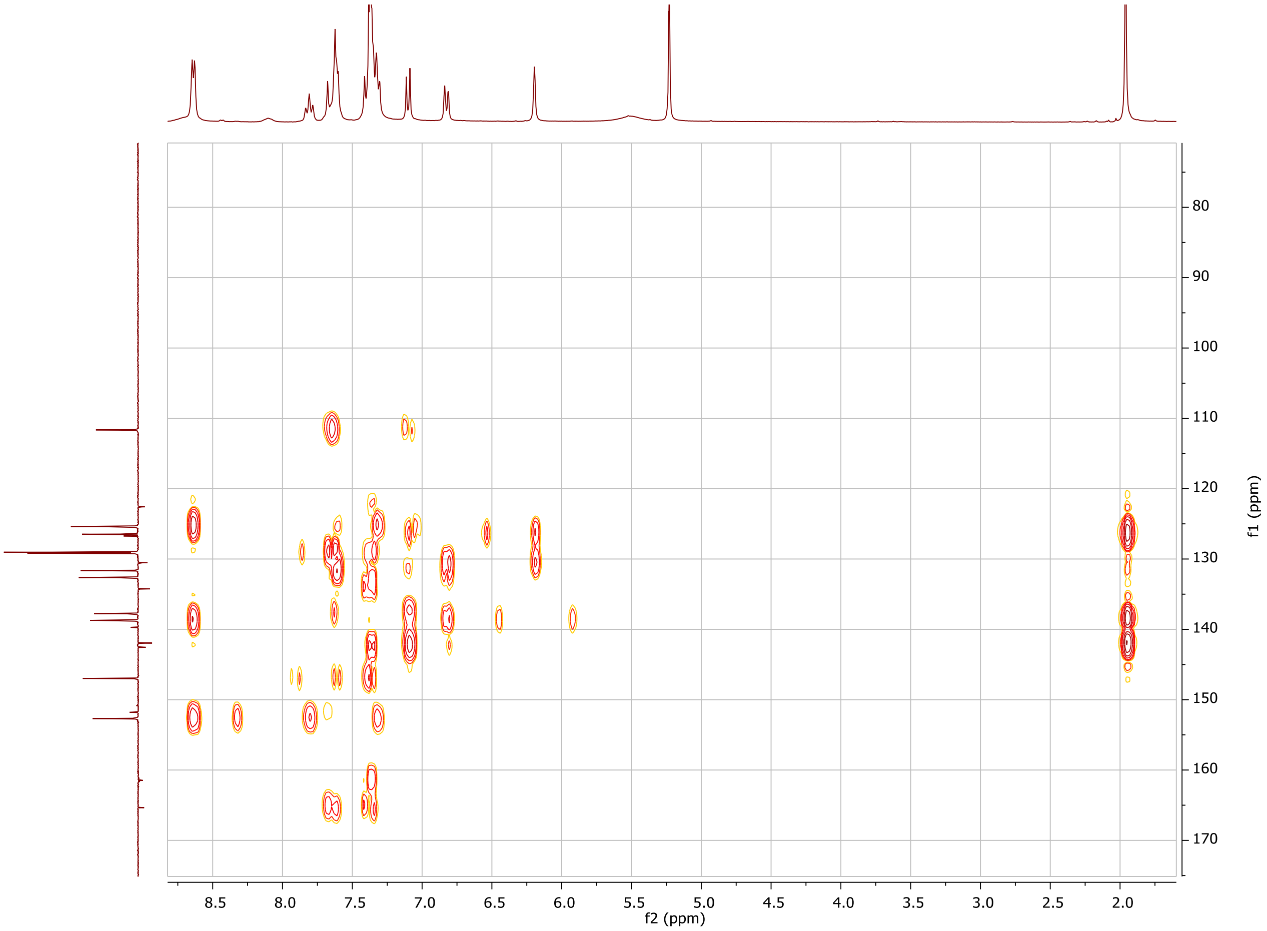

^1H - ^{13}C HMBC correlation spectrum of **3g**

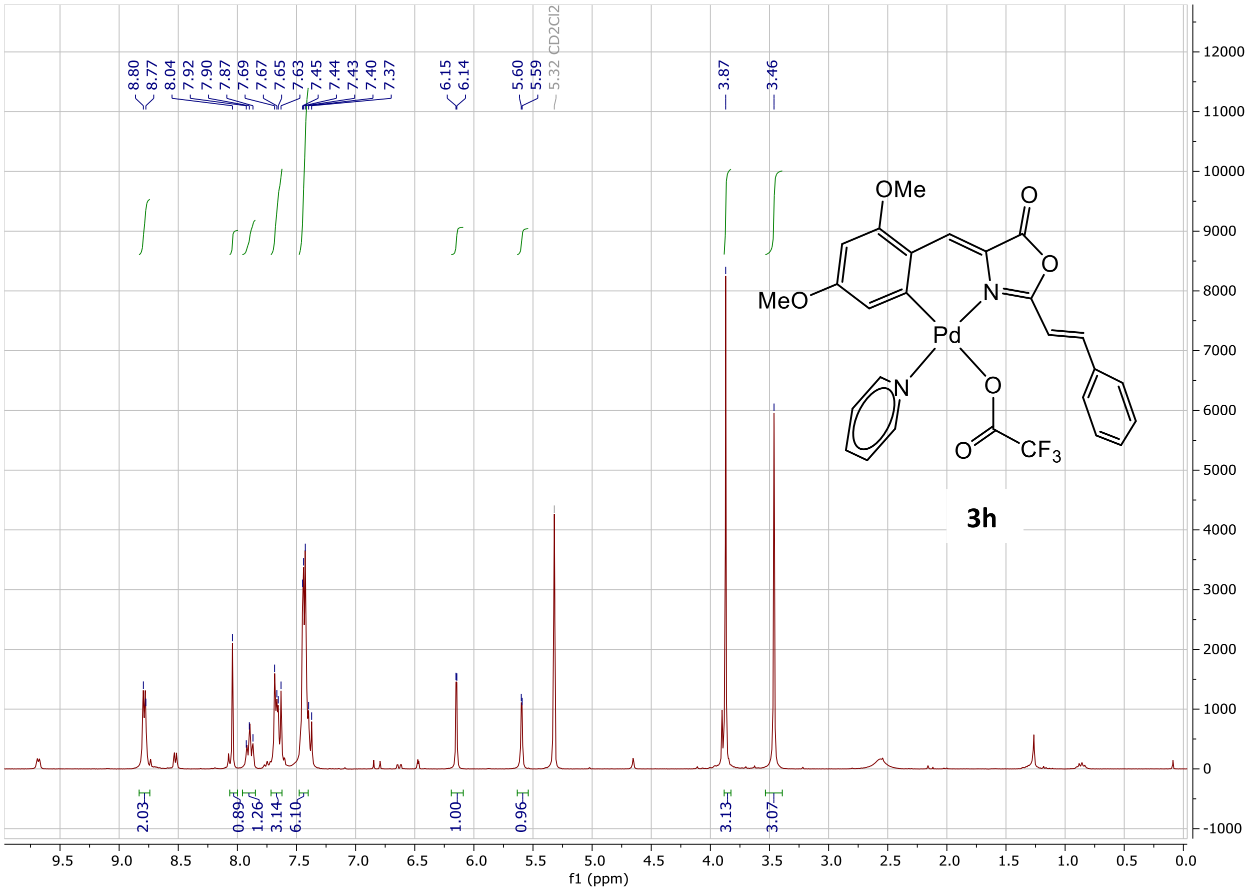

^1H-NMR spectrum (CD_2Cl_2, 300.13 MHz) of **3h**

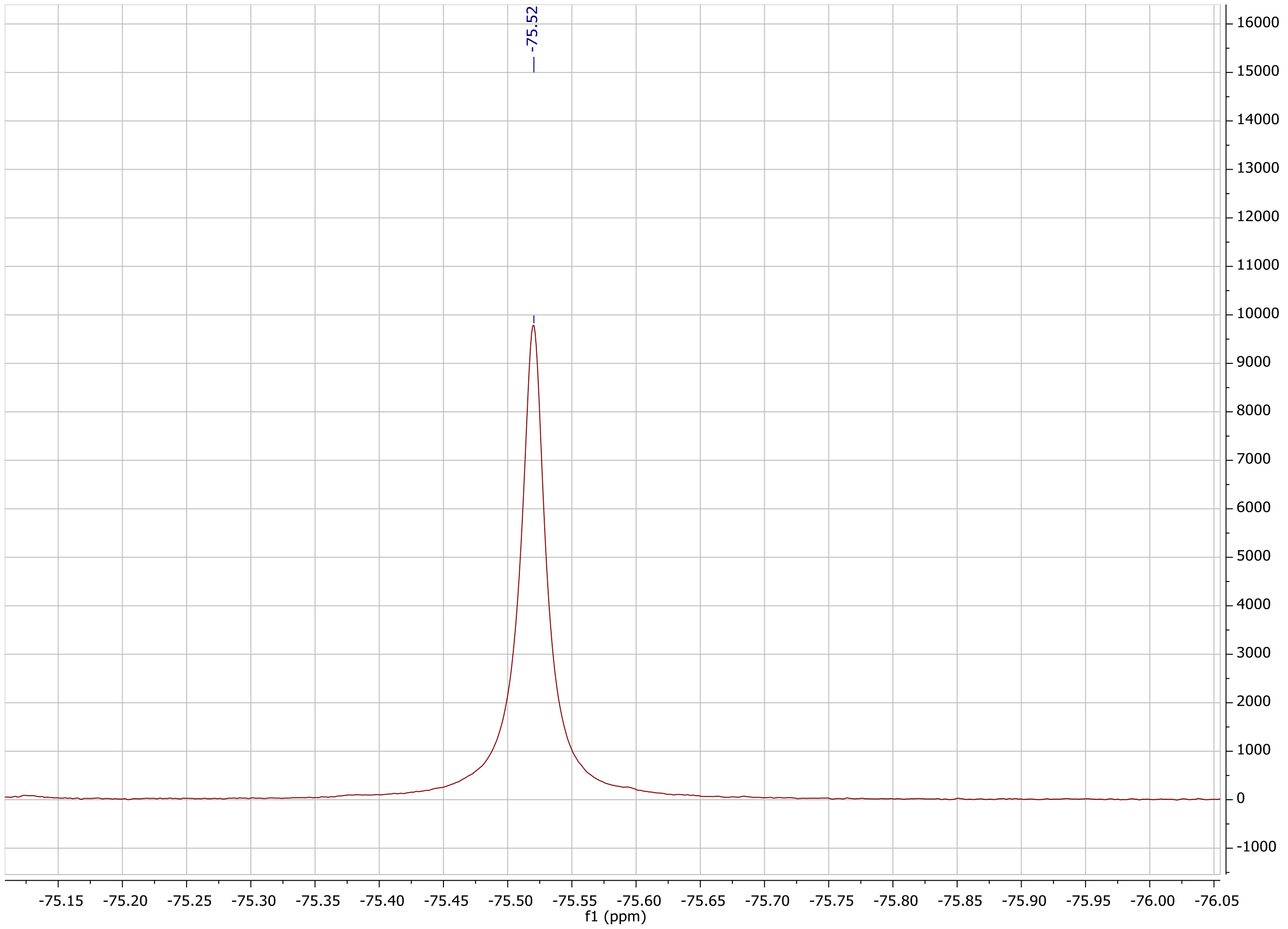

^{19}F-NMR spectrum (CD_2Cl_2, 282.40 MHz) of **3h**

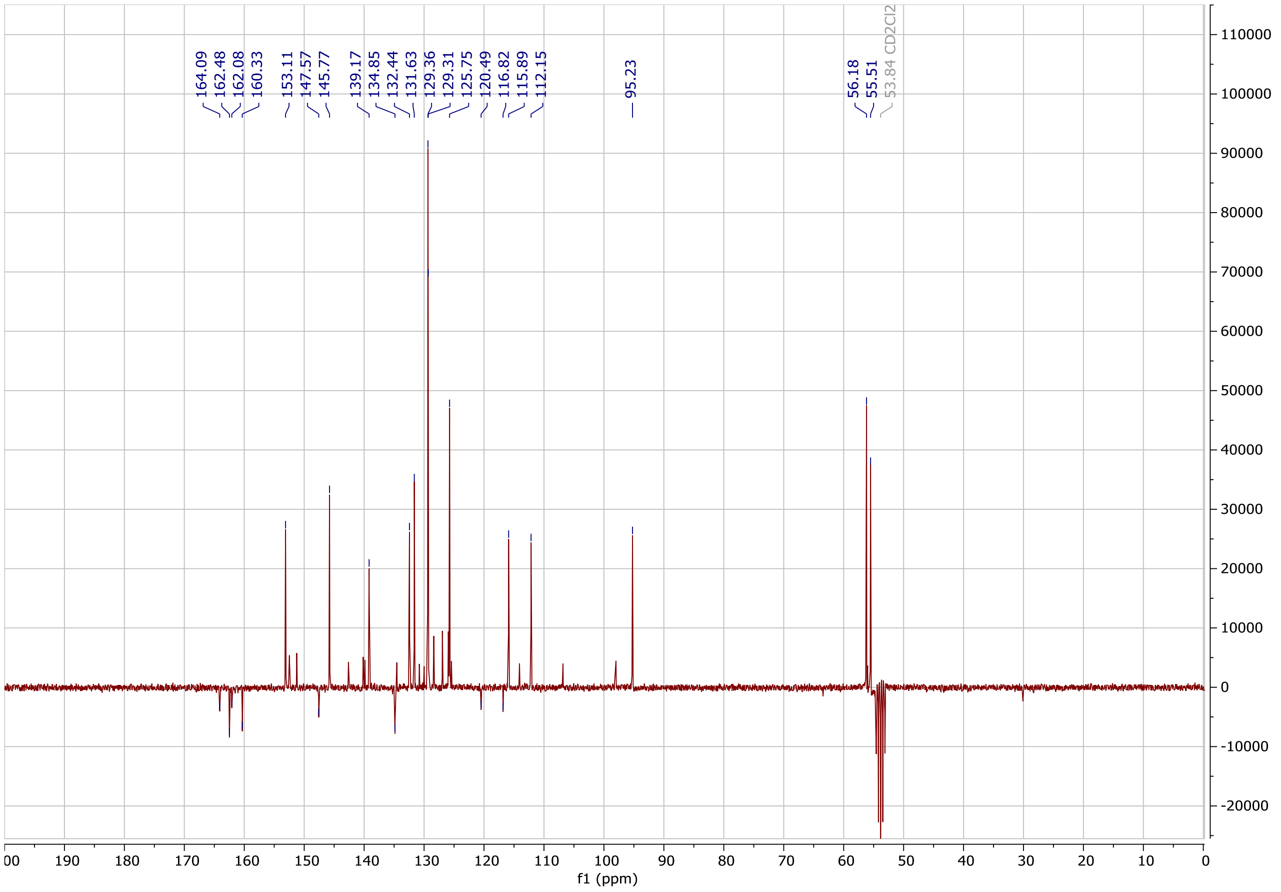

^{13}C{^{1}H} (APT) NMR spectrum (CD_2Cl_2, 75.47 MHz) of **3h**

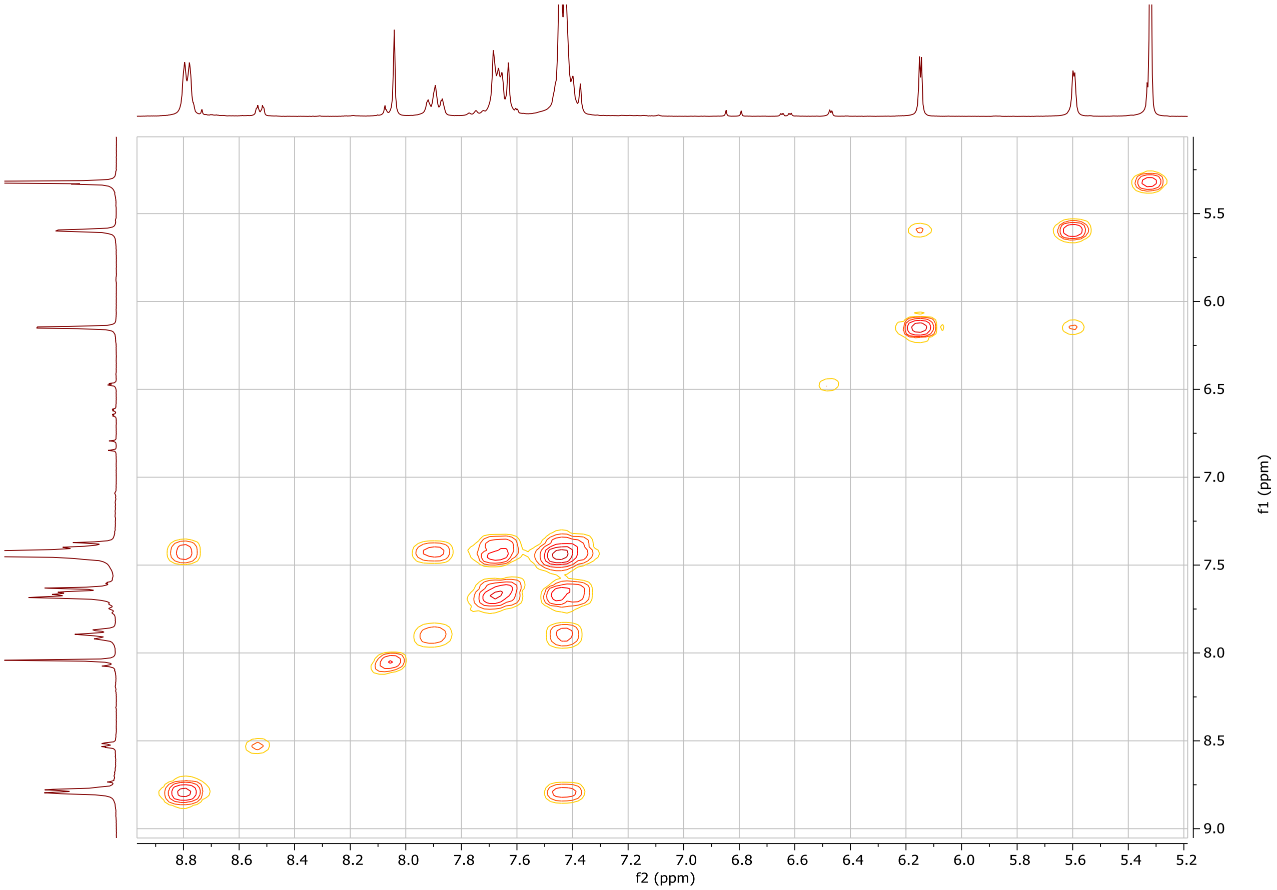

^{1}H – ^{1}H COSY correlation spectrum of **3h** (aromatic region)

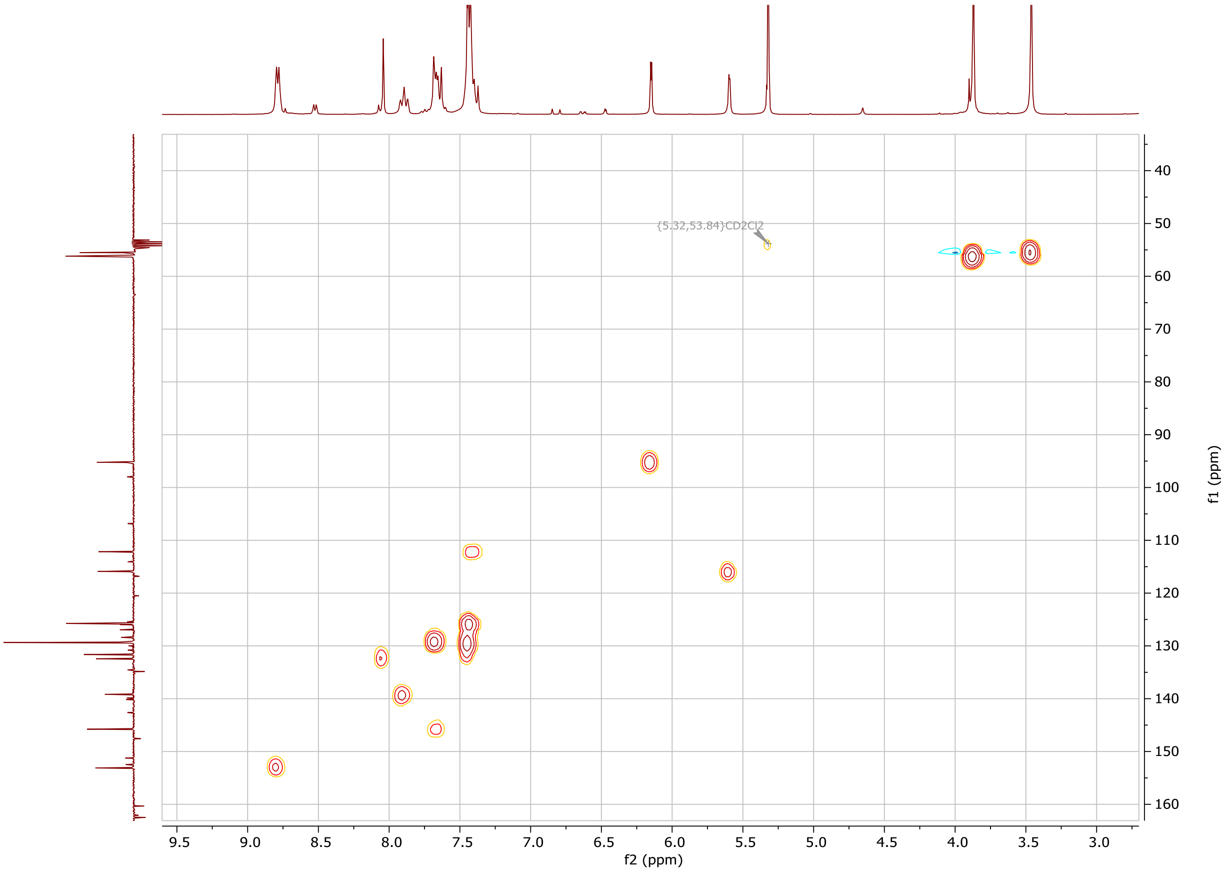

^{1}H - ^{13}C HSQC correlation spectrum of **3h**

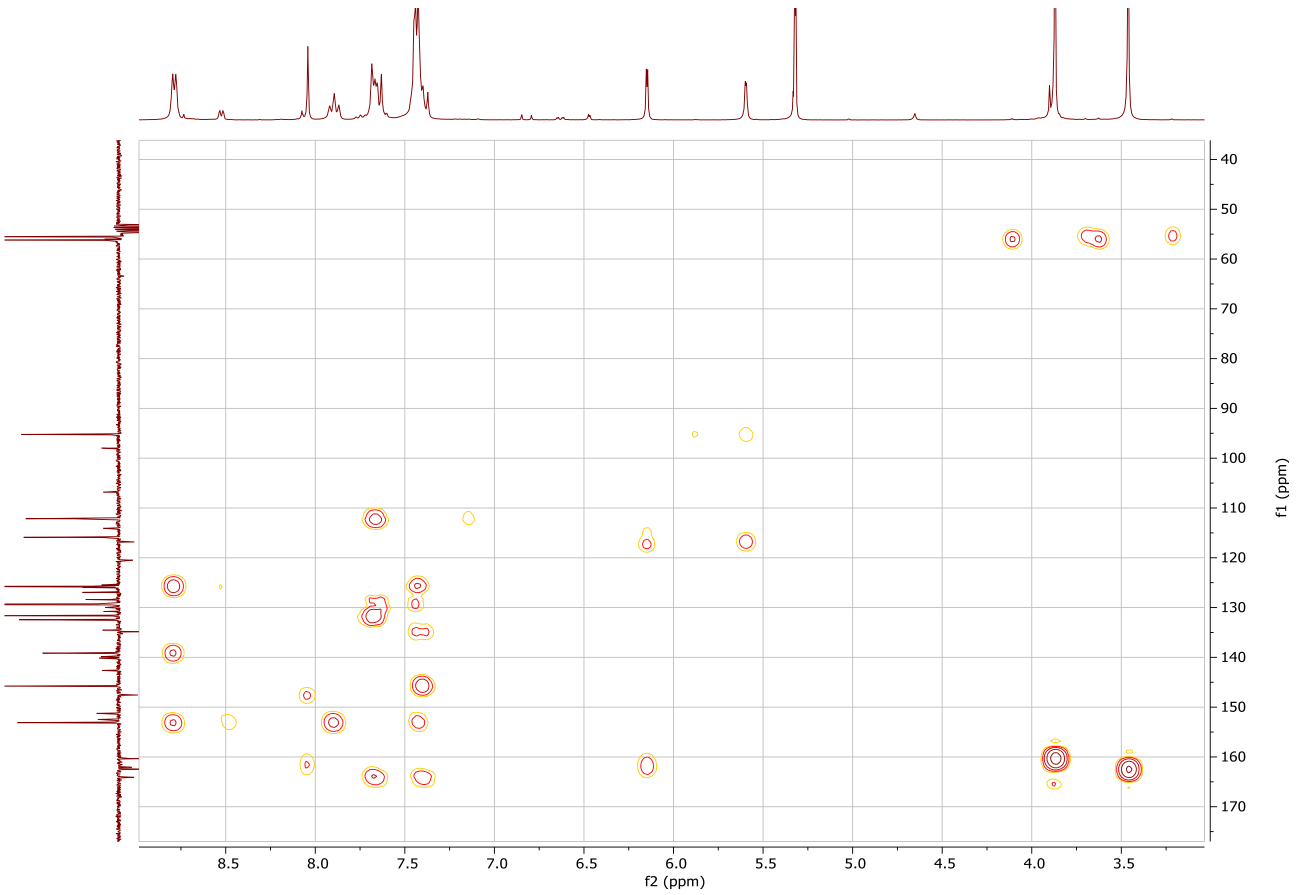

^{1}H - ^{13}C HMBC correlation spectrum of **3h**

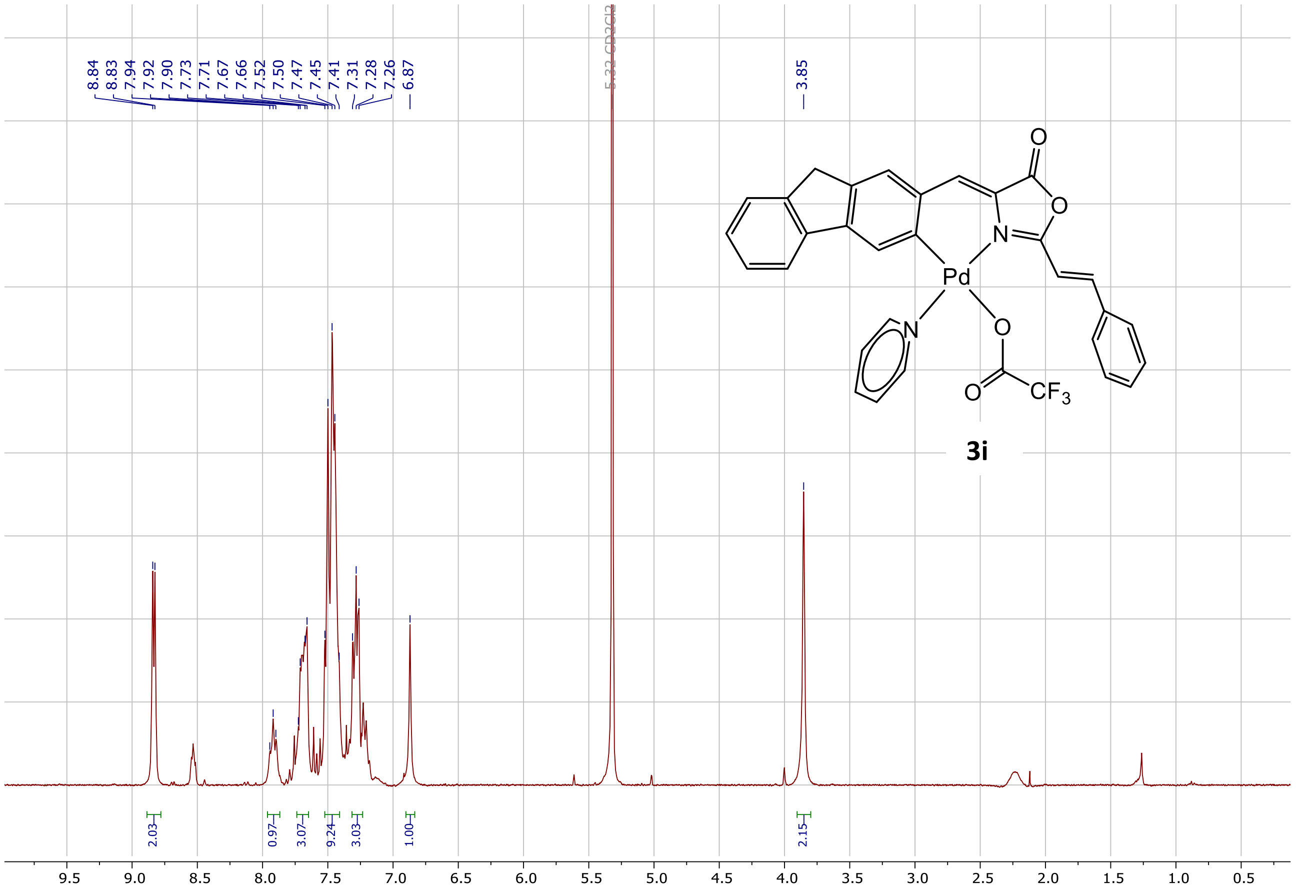

^{1}H-NMR spectrum (CD_2Cl_2, 300.13 MHz) of **3i**

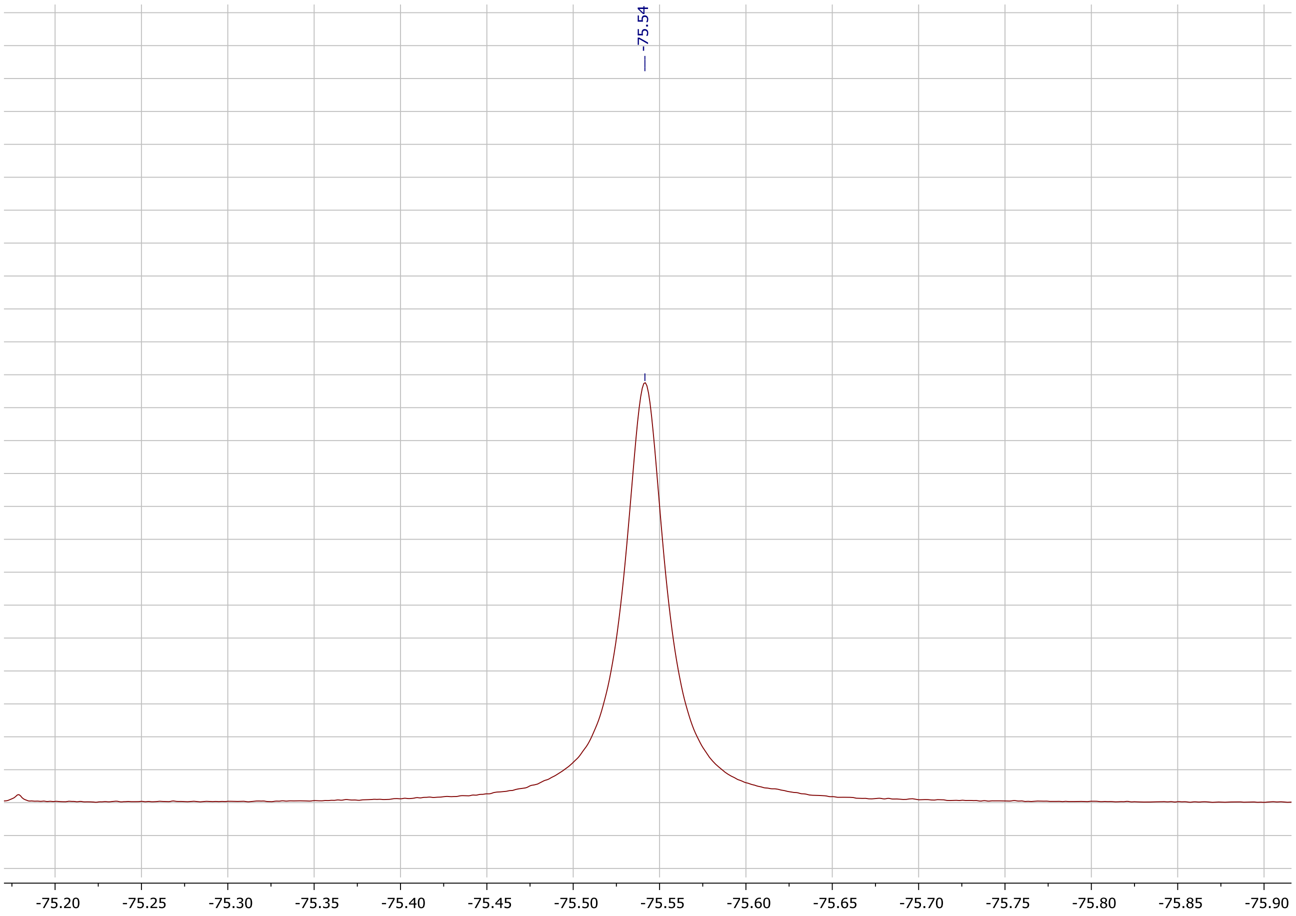

^{19}F-NMR spectrum (CD_2Cl_2, 282.40 MHz) of **3i**

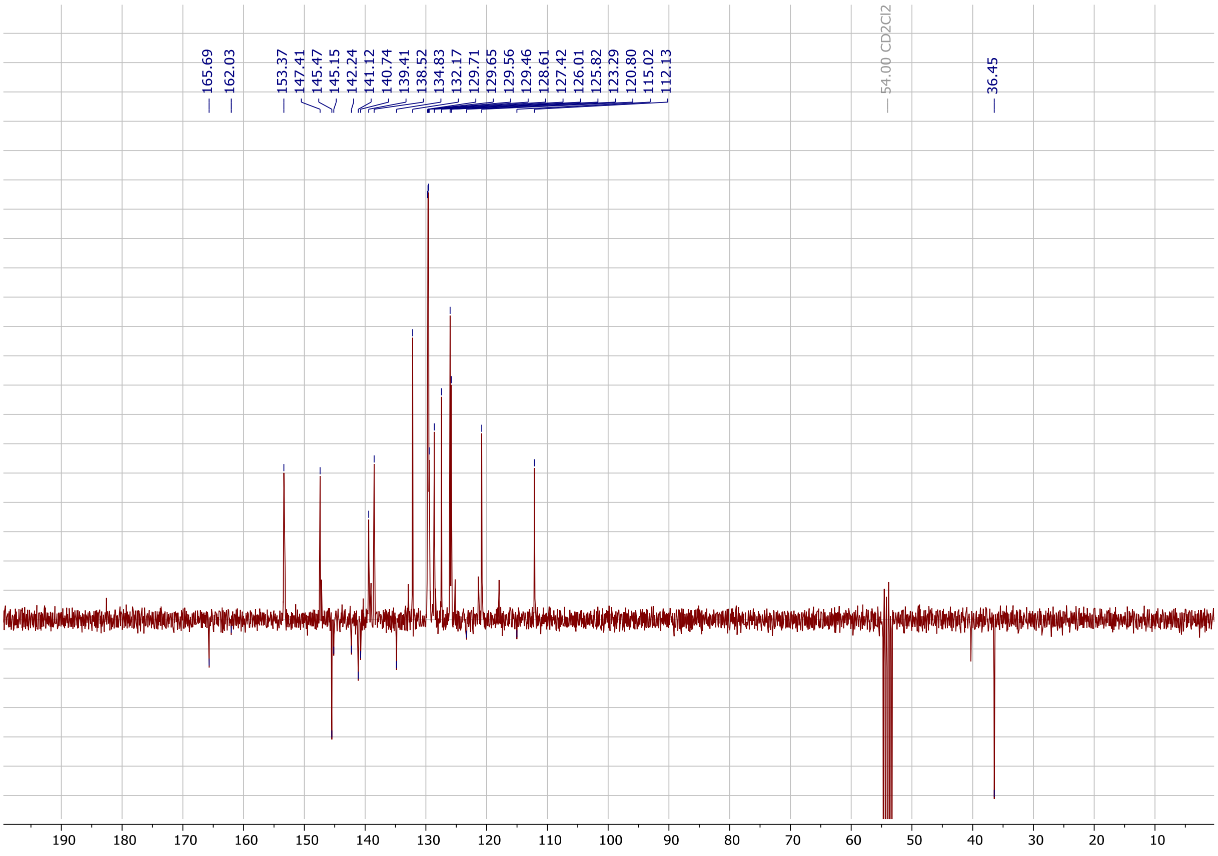

^{13}C{^{1}H} (APT) NMR spectrum (CD_2Cl_2, 75.47 MHz) of **3i**

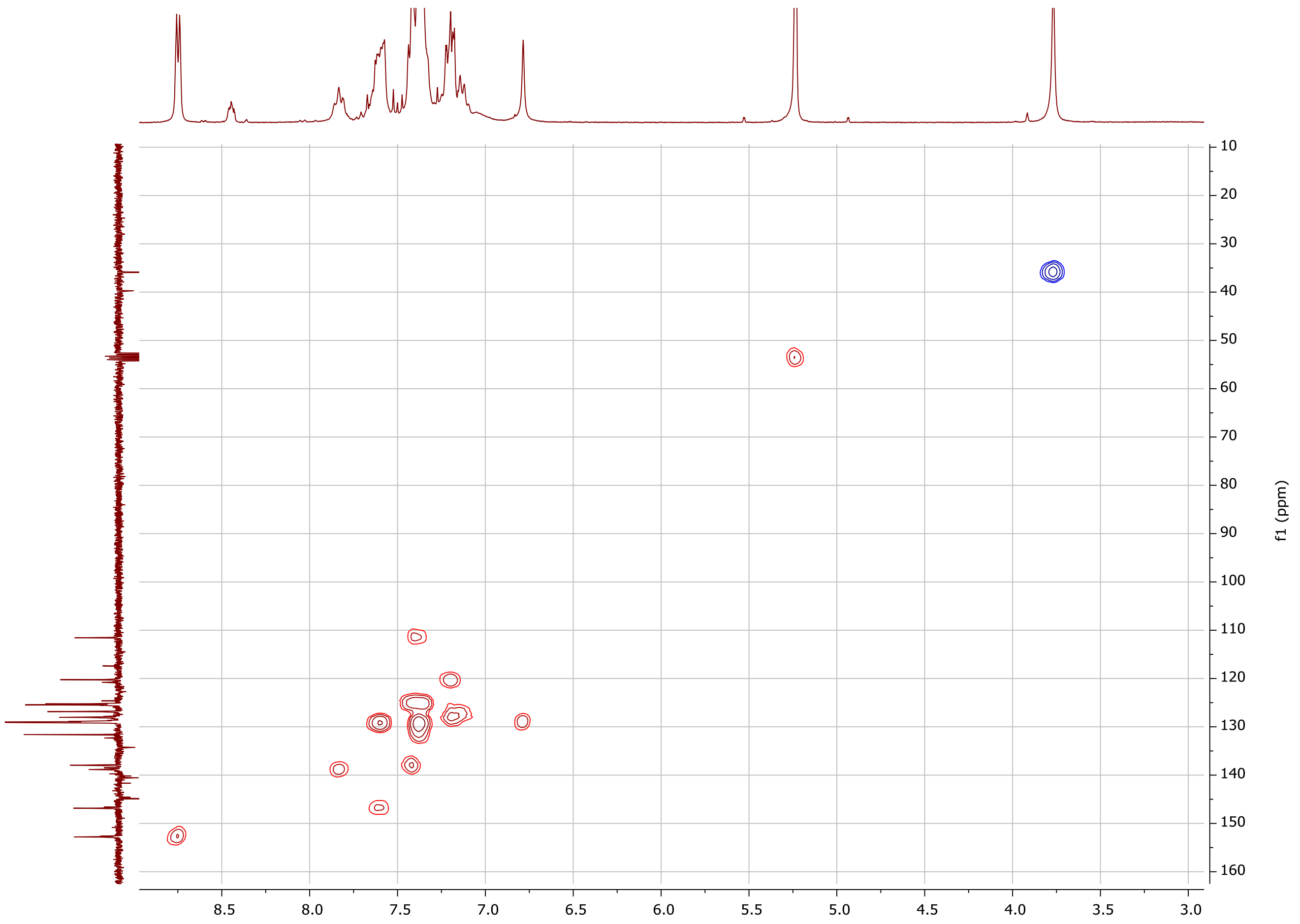

^{1}H - ^{13}C HSQC correlation spectrum of **3i**

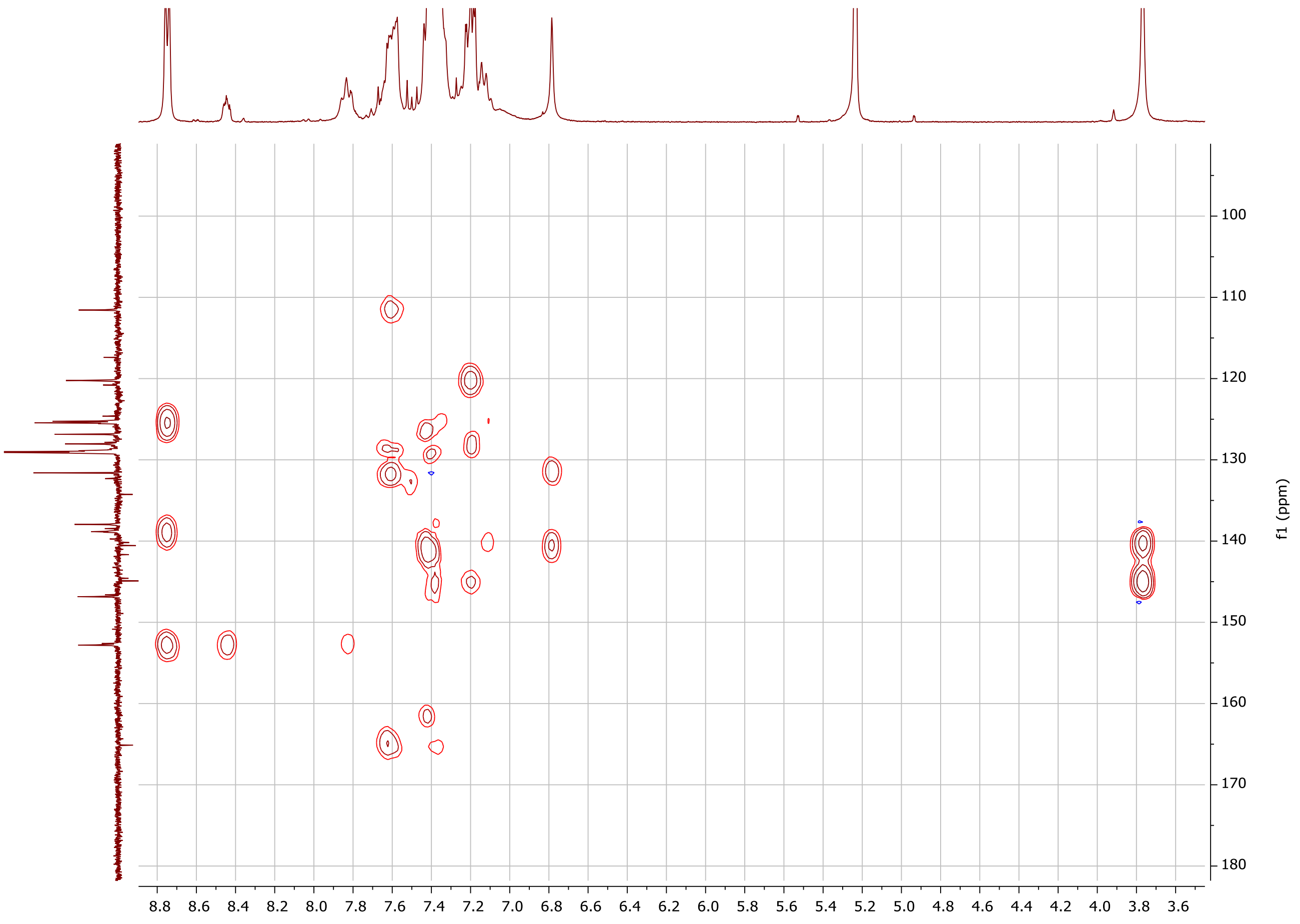

^{1}H - ^{13}C HMBC correlation spectrum of **3i**

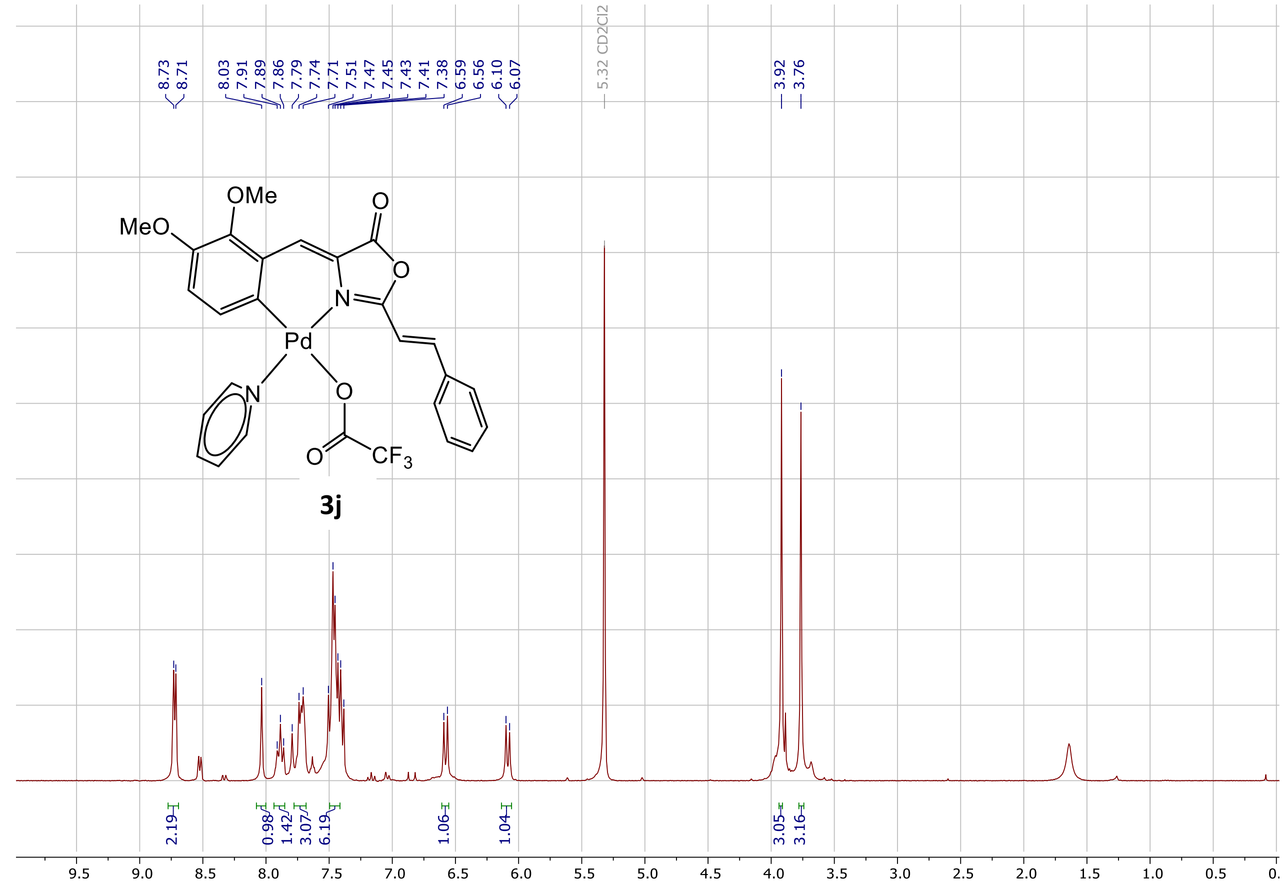

^{1}H-NMR spectrum (CD_2Cl_2, 300.13 MHz) of **3j**

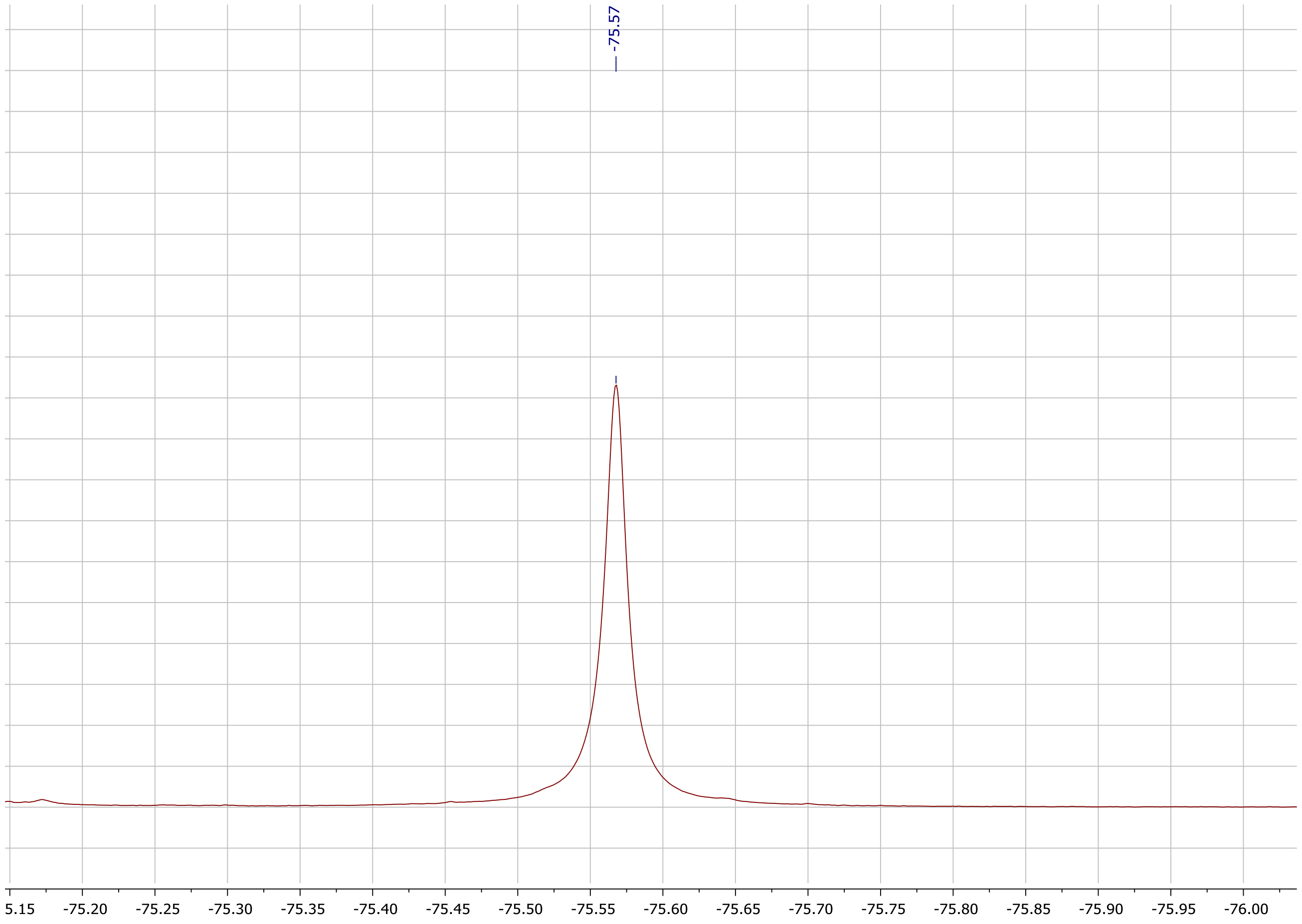

^{19}F-NMR spectrum (CD_2Cl_2, 282.40 MHz) of **3j**

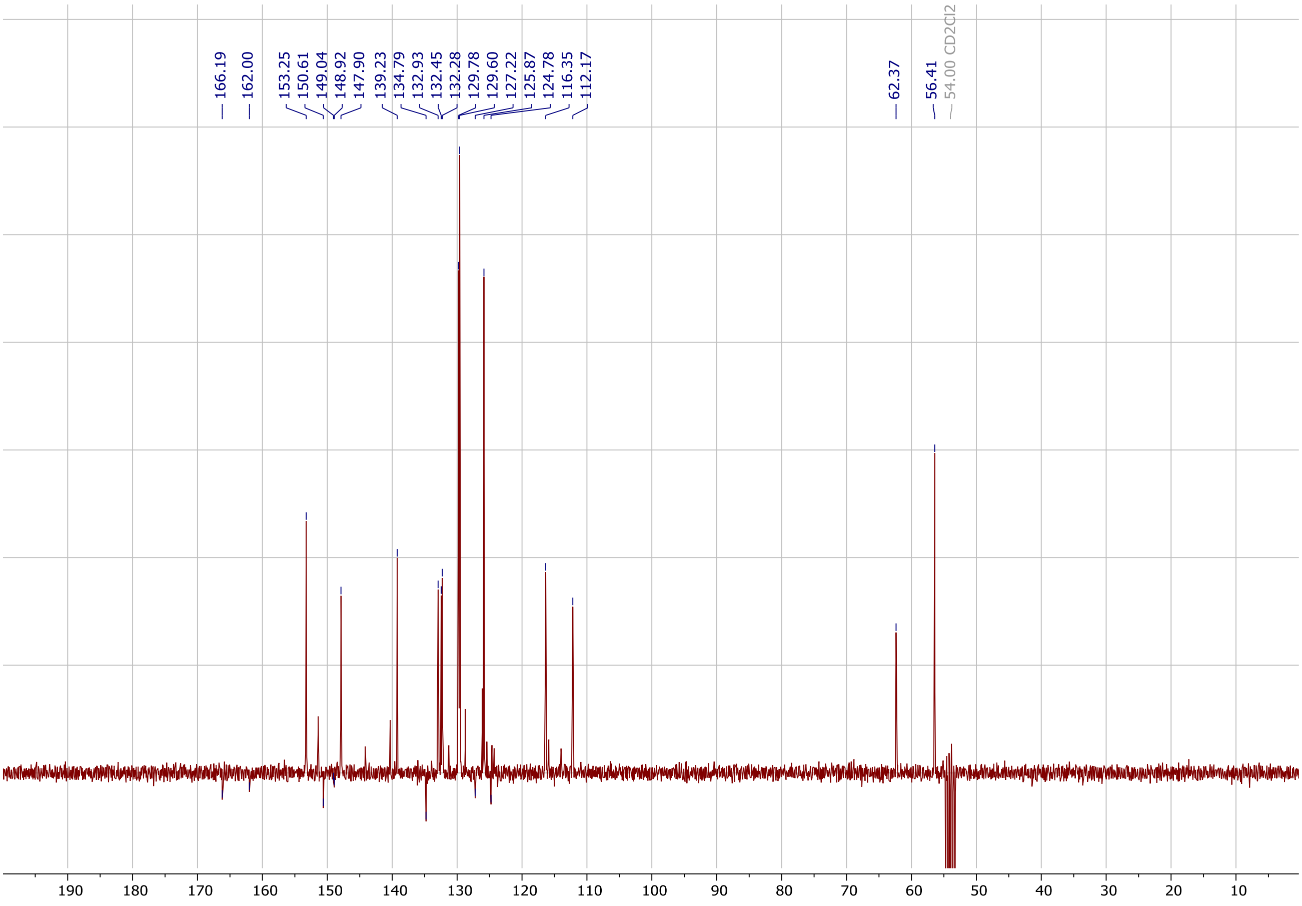

$^{13}C\{^{1}H\}$ (APT) NMR spectrum (CD_2Cl_2, 75.47 MHz) of **3j**

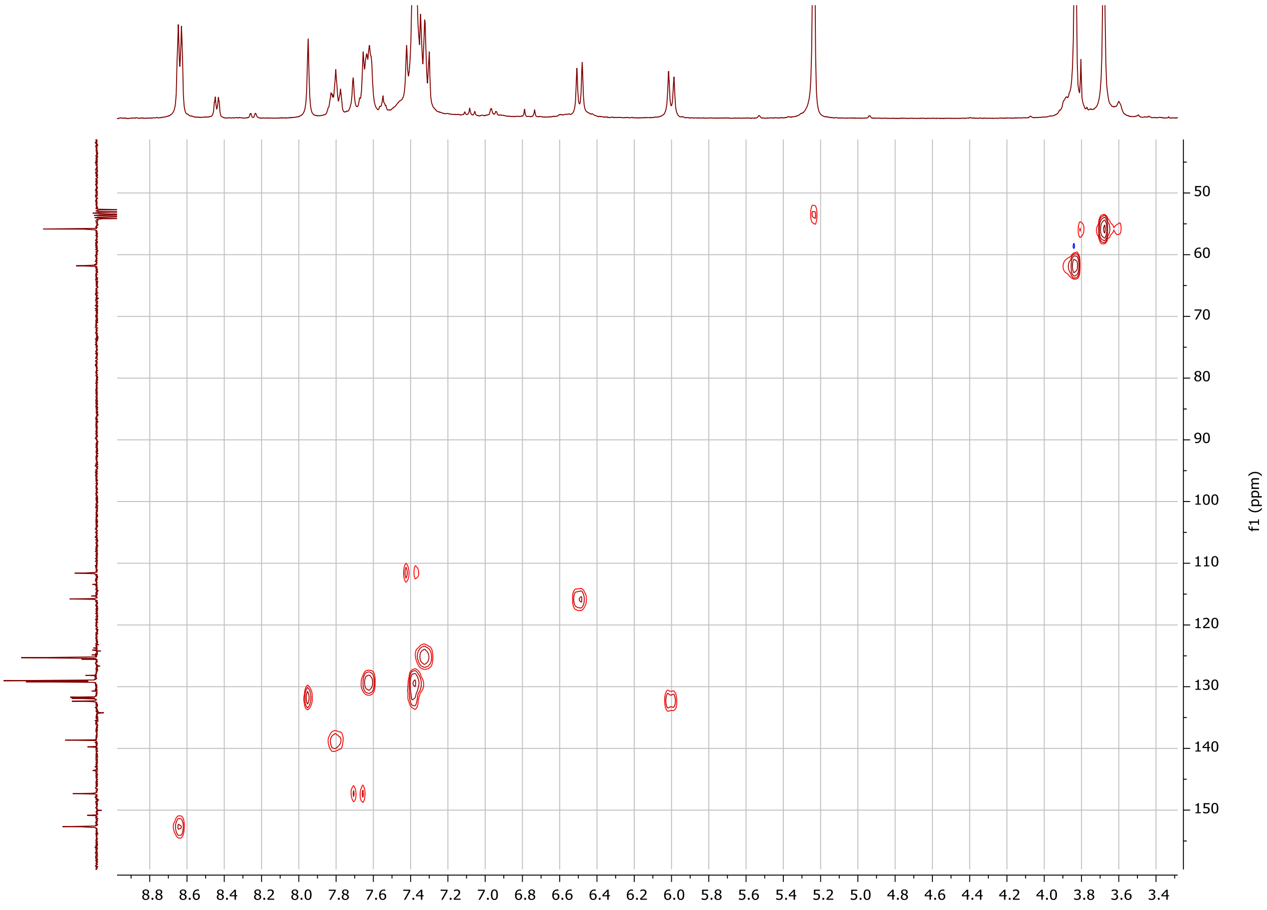

^{1}H - ^{13}C HSQC correlation spectrum of **3j**

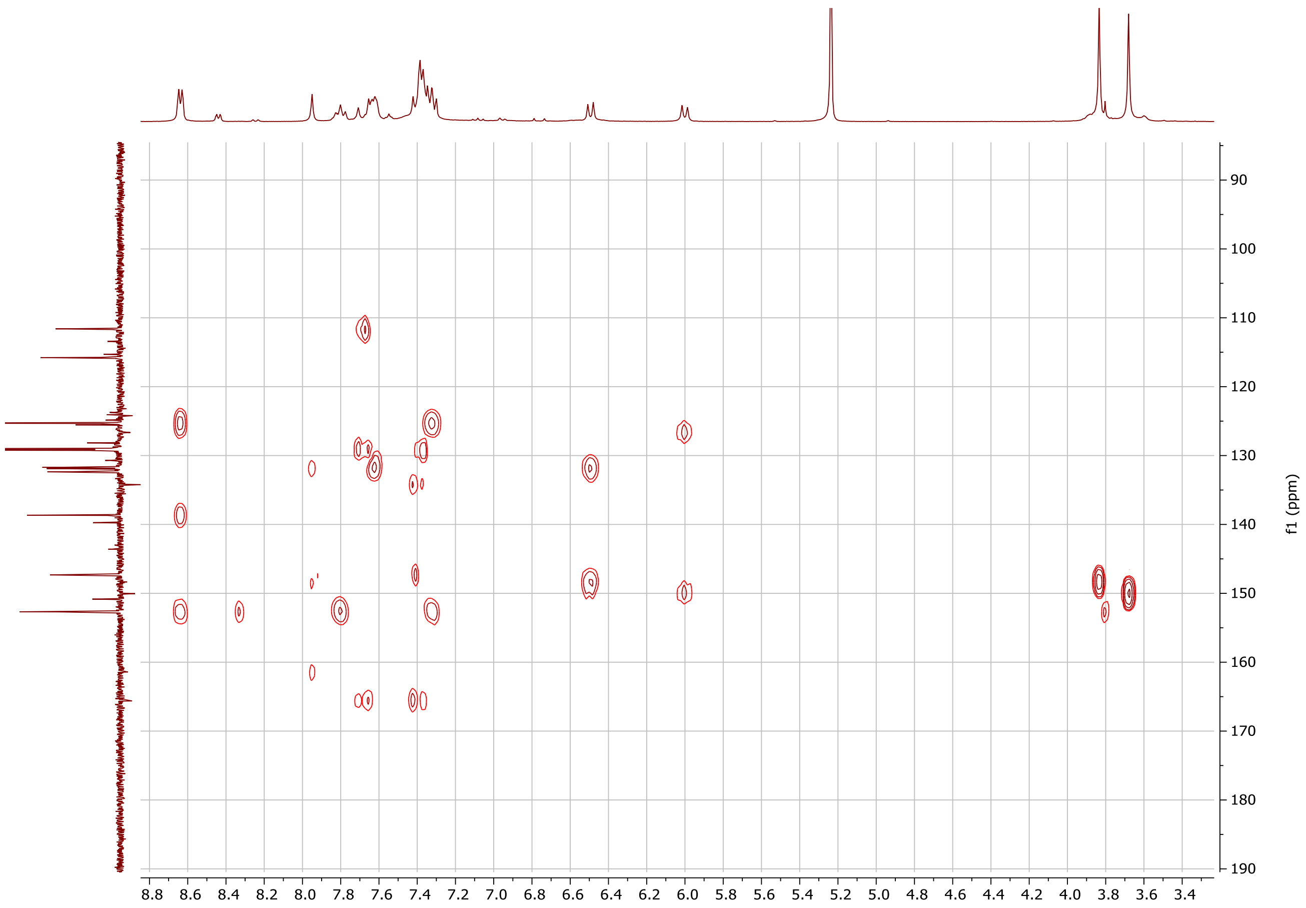

^{1}H - ^{13}C HMBC correlation spectrum of **3j**

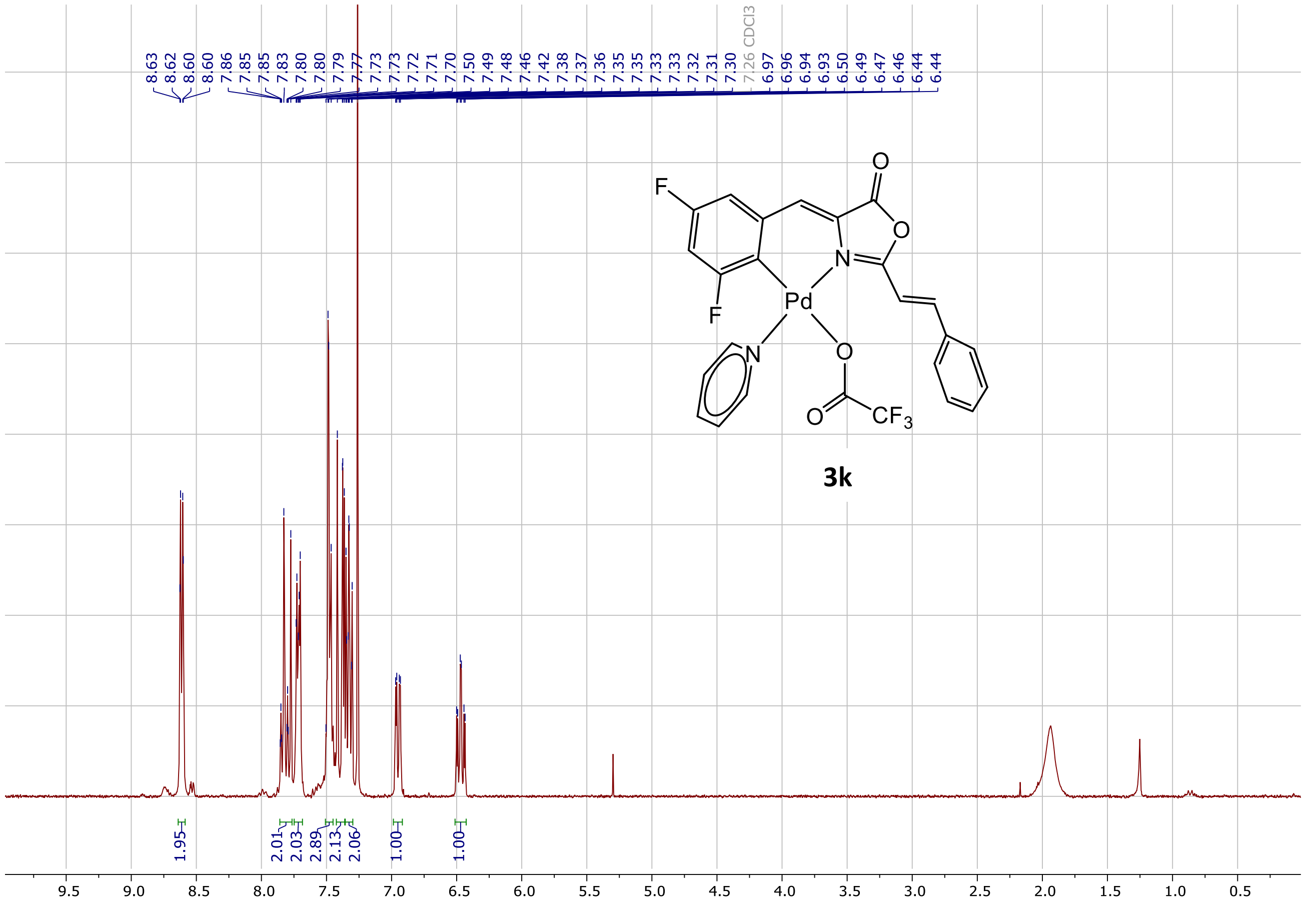

3k

^{1}H-NMR spectrum ($CDCl_3$, 300.13 MHz) of **3k**

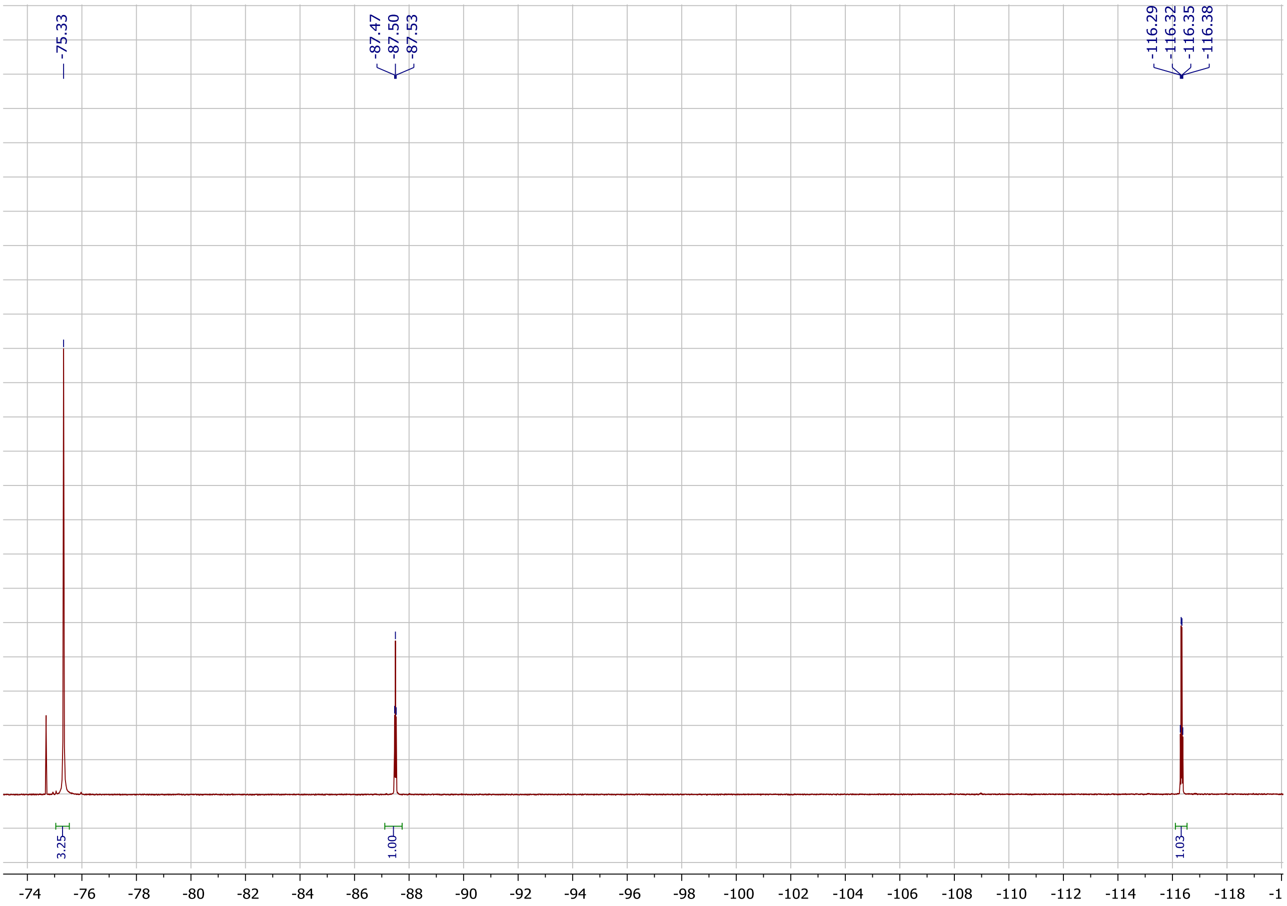

^{19}F-NMR spectrum ($CDCl_3$, 282.40 MHz) of **3k**

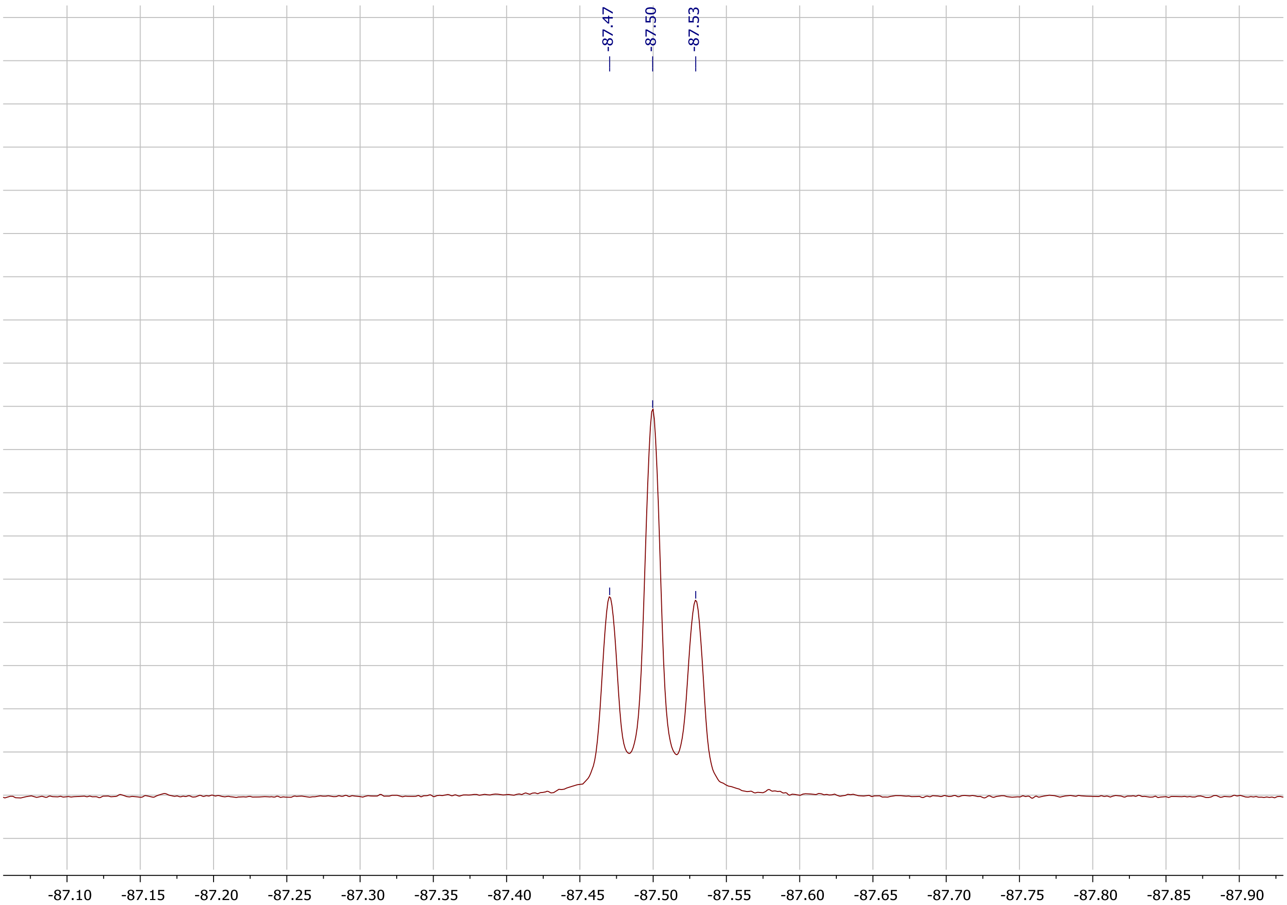

^{19}F-NMR spectrum ($CDCl_3$, 282.40 MHz) of **3k** (amplification)

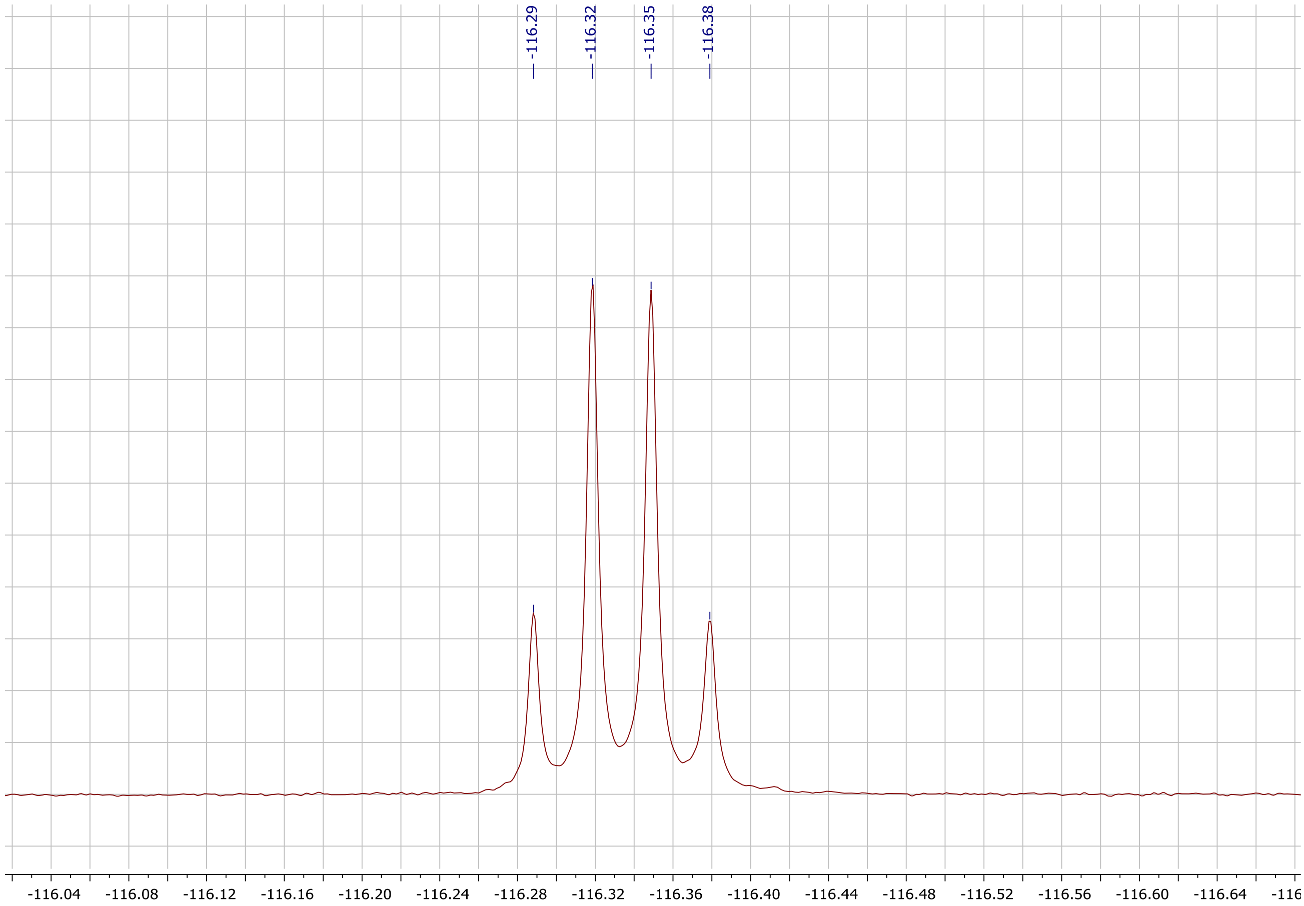

^{19}F-NMR spectrum ($CDCl_3$, 282.40 MHz) of **3k** (amplification)

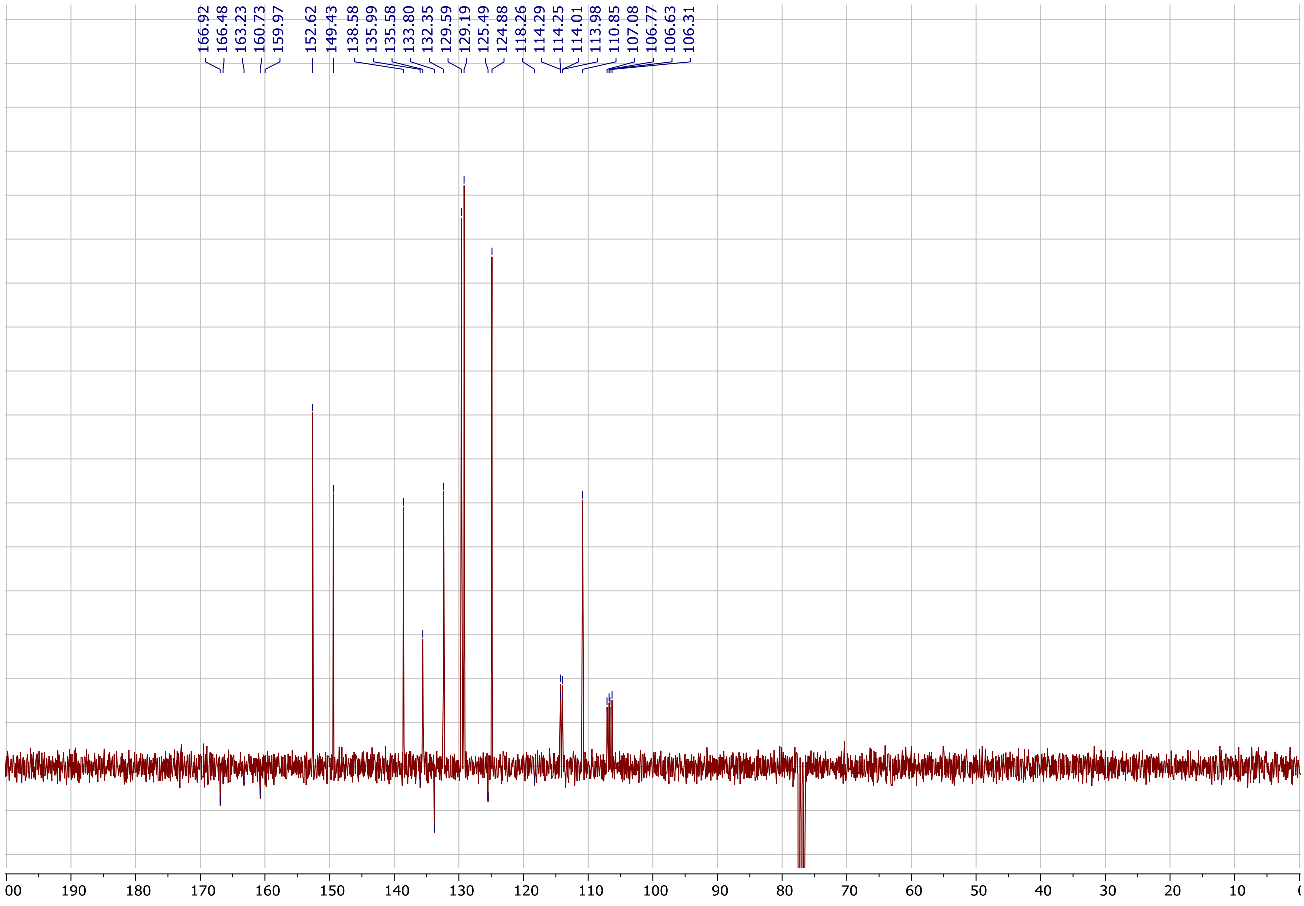

^{13}C{^{1}H} (APT) NMR spectrum ($CDCl_3$, 75.47 MHz) of **3k**

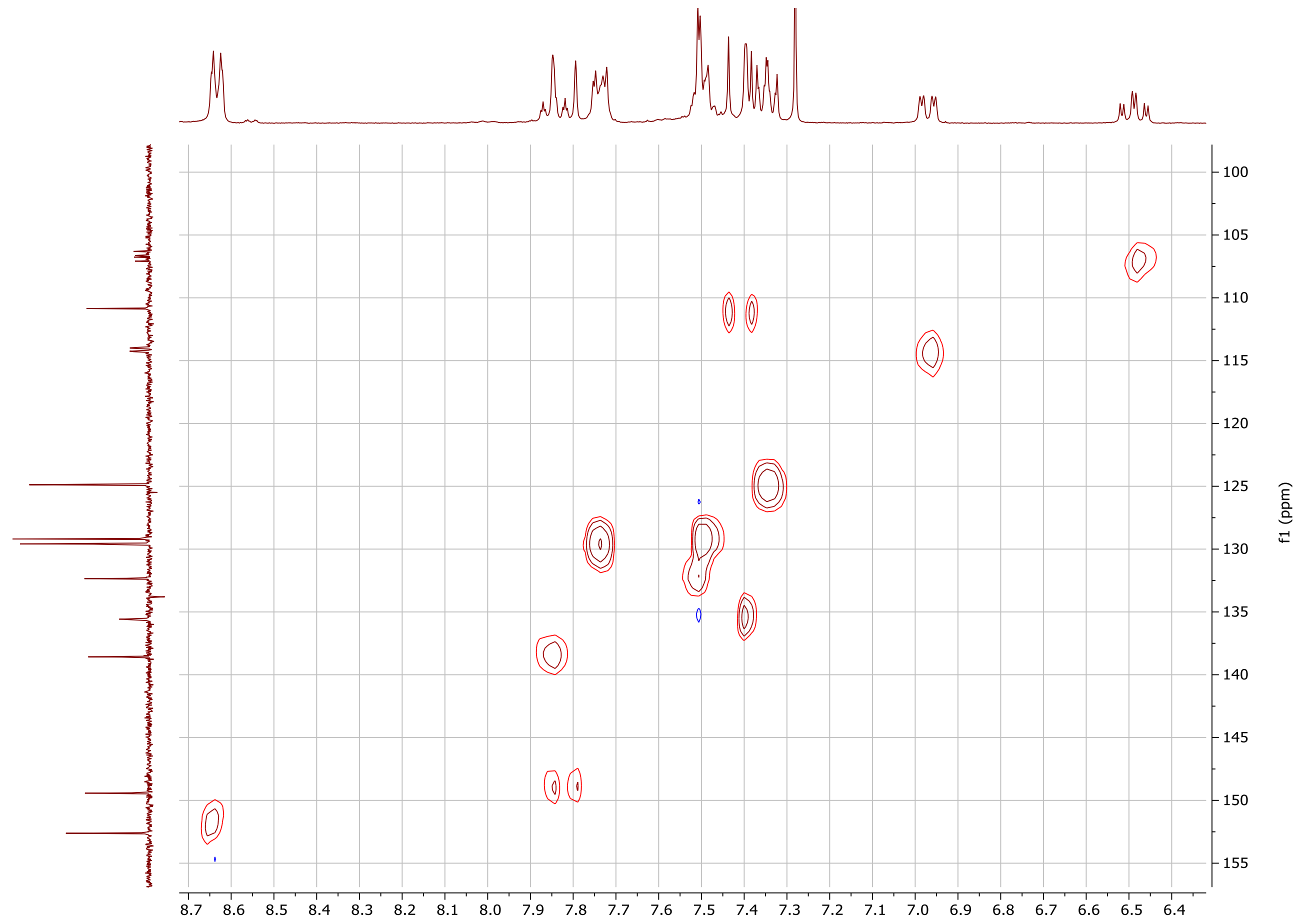

^{1}H - ^{13}C HSQC correlation spectrum of **3k**

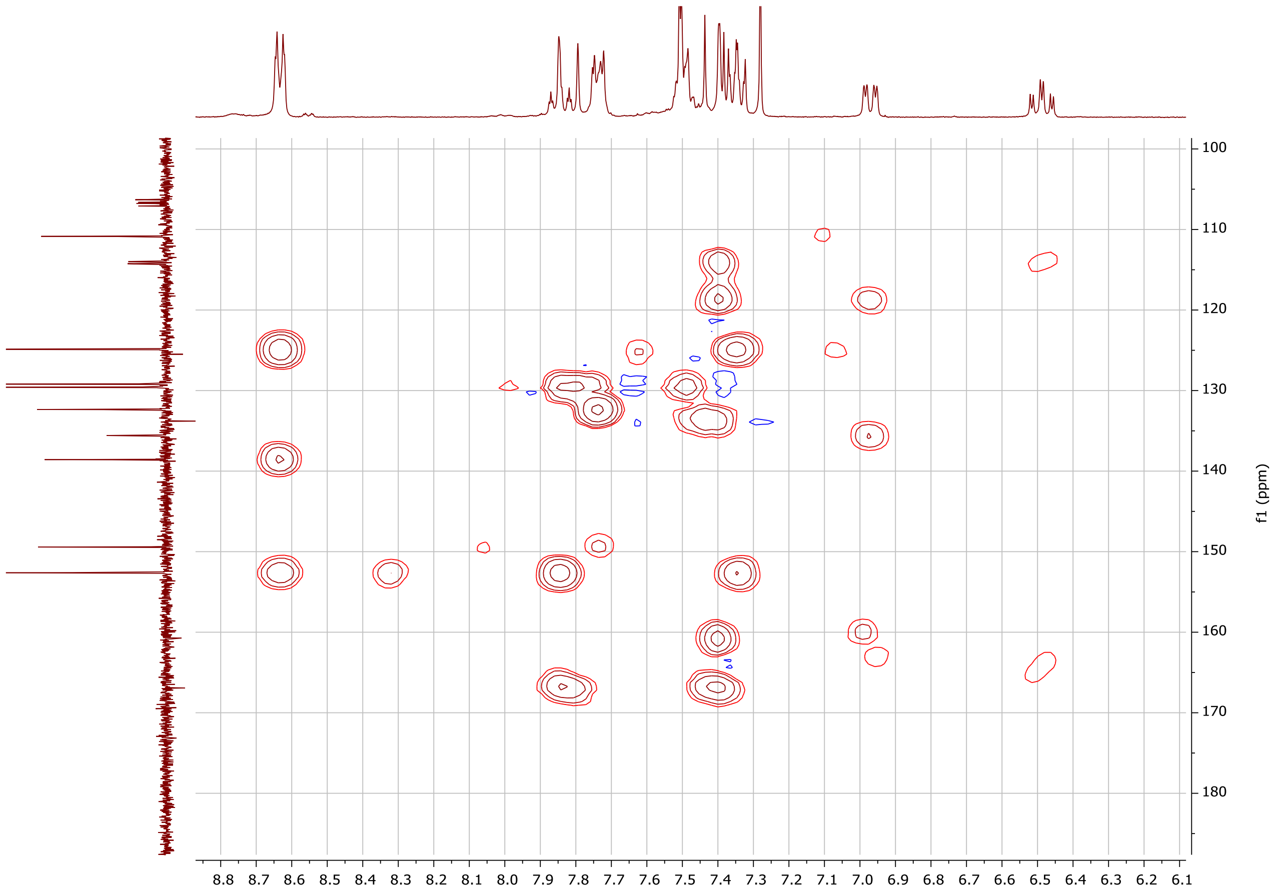

^{1}H - ^{13}C HMBC correlation spectrum of **3k**

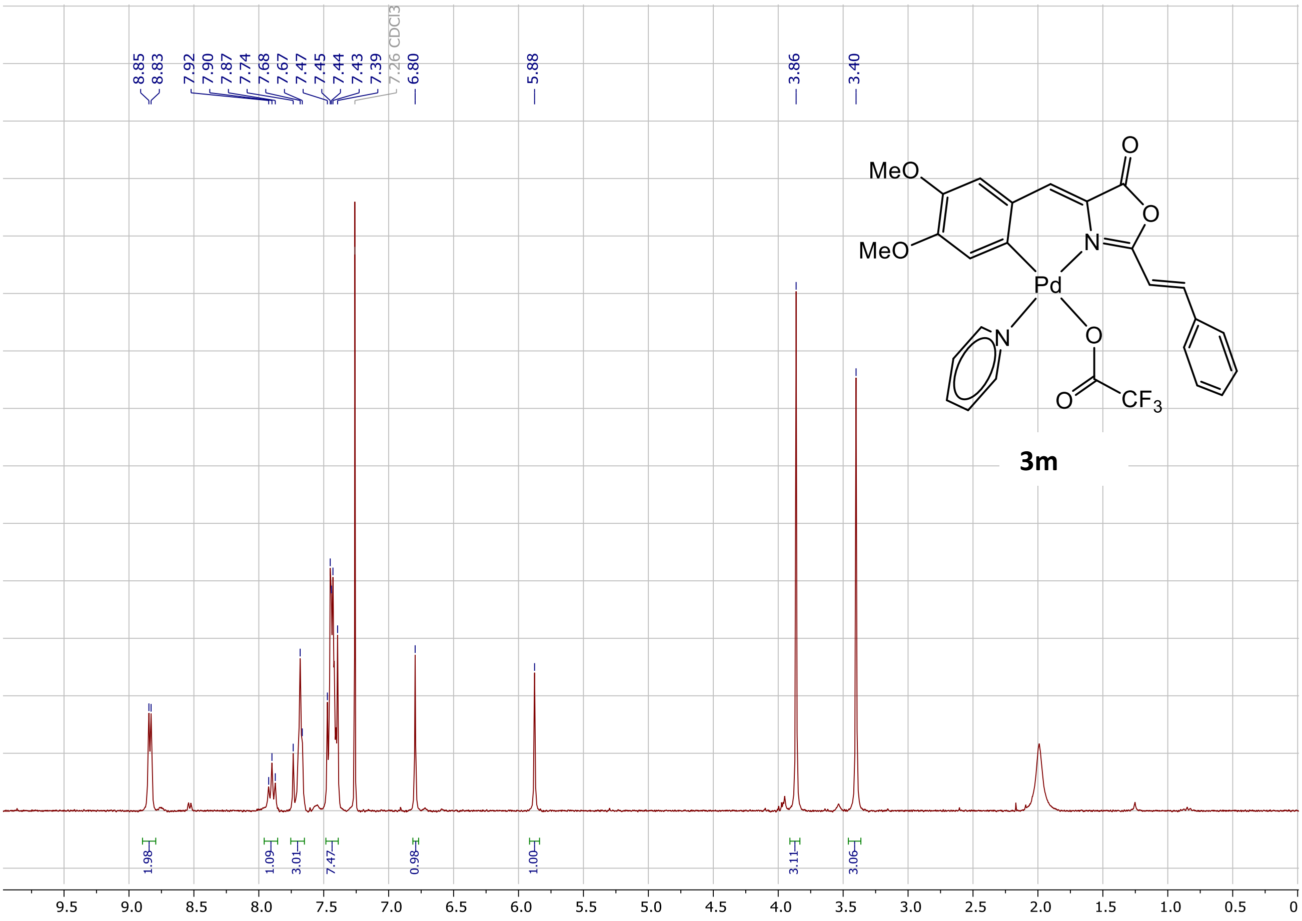

^{1}H-NMR spectrum ($CDCl_3$, 300.13 MHz) of **3m**

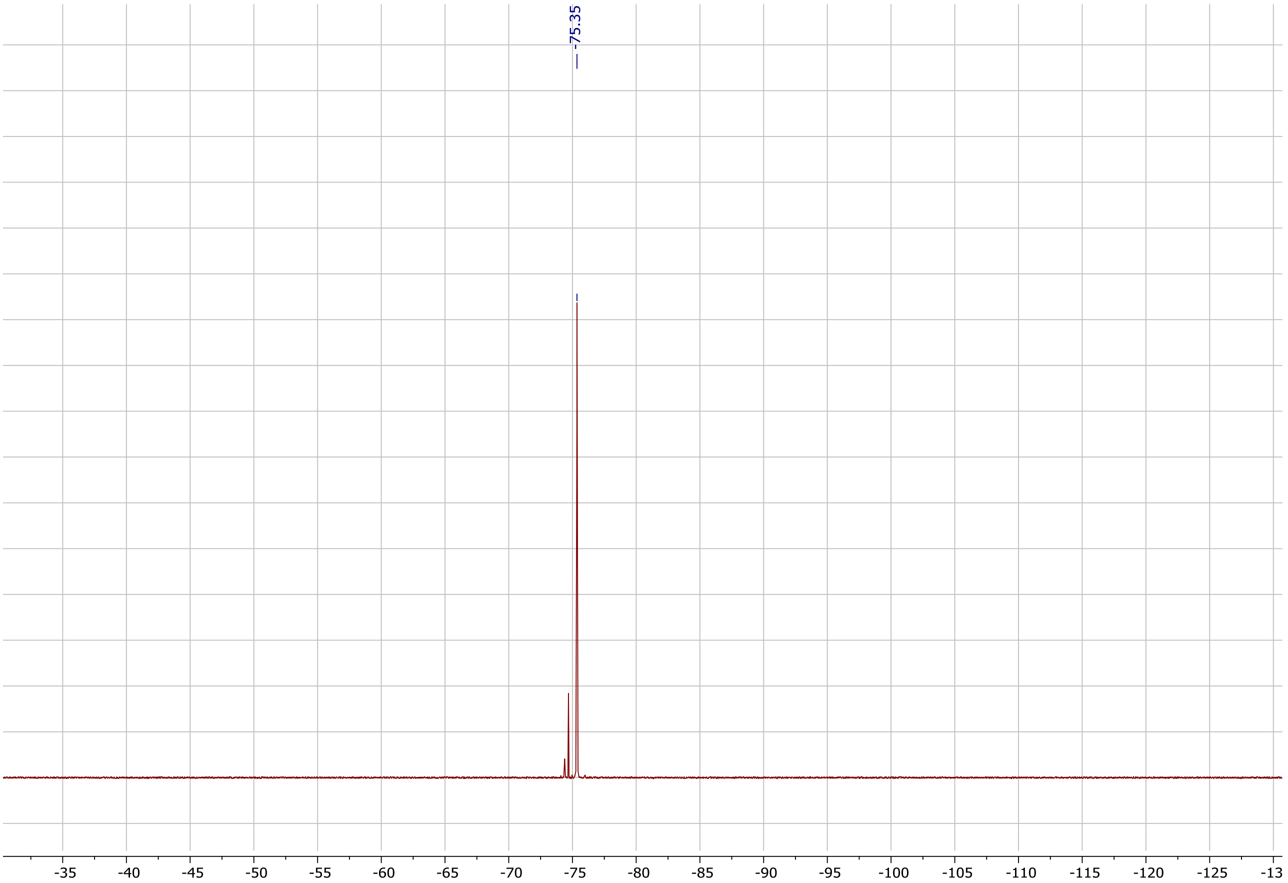

^{19}F-NMR spectrum ($CDCl_3$, 282.40 MHz) of **3m**

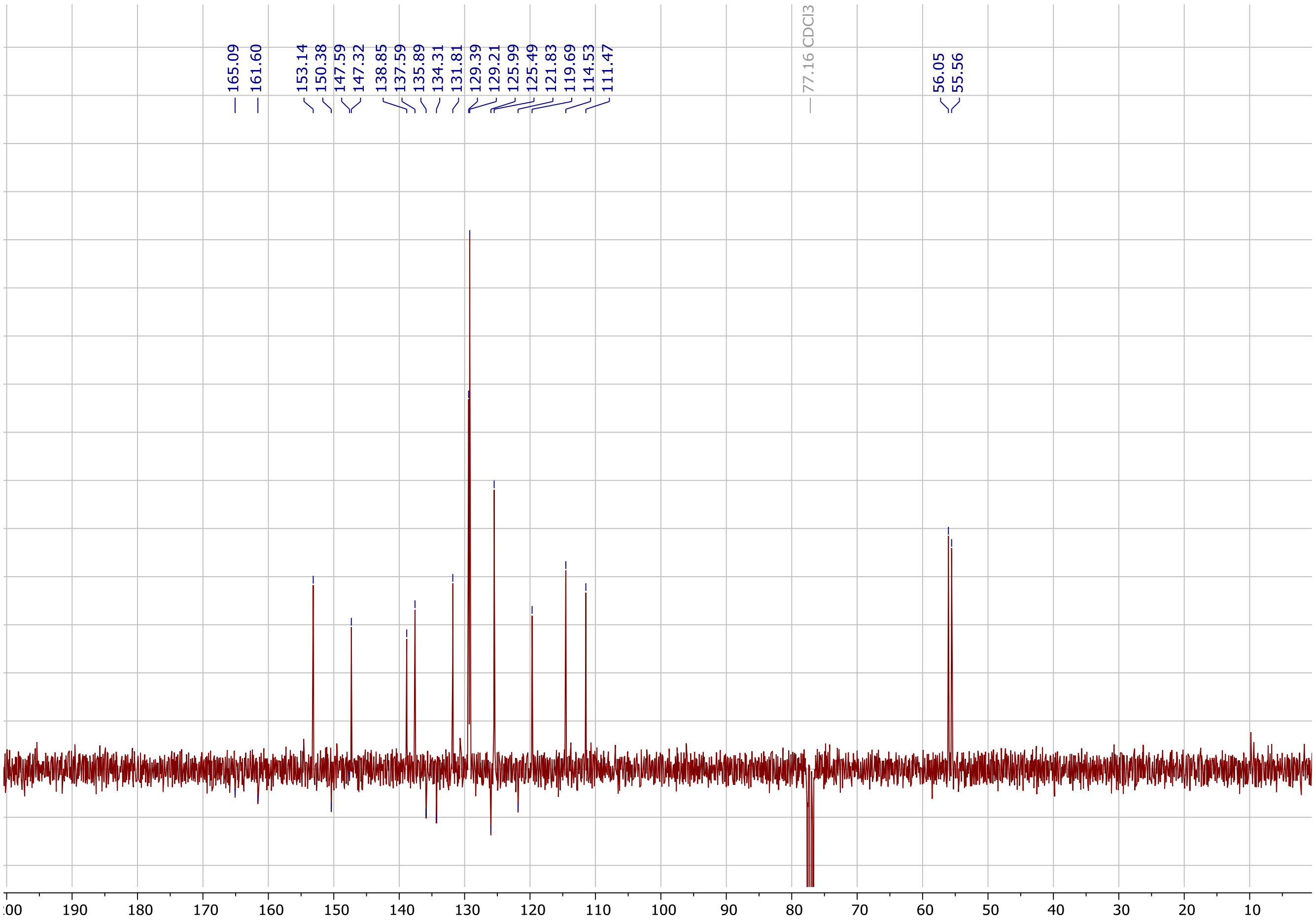

$^{13}C\{^{1}H\}$ (APT) NMR spectrum ($CDCl_3$, 75.47 MHz) of **3m**

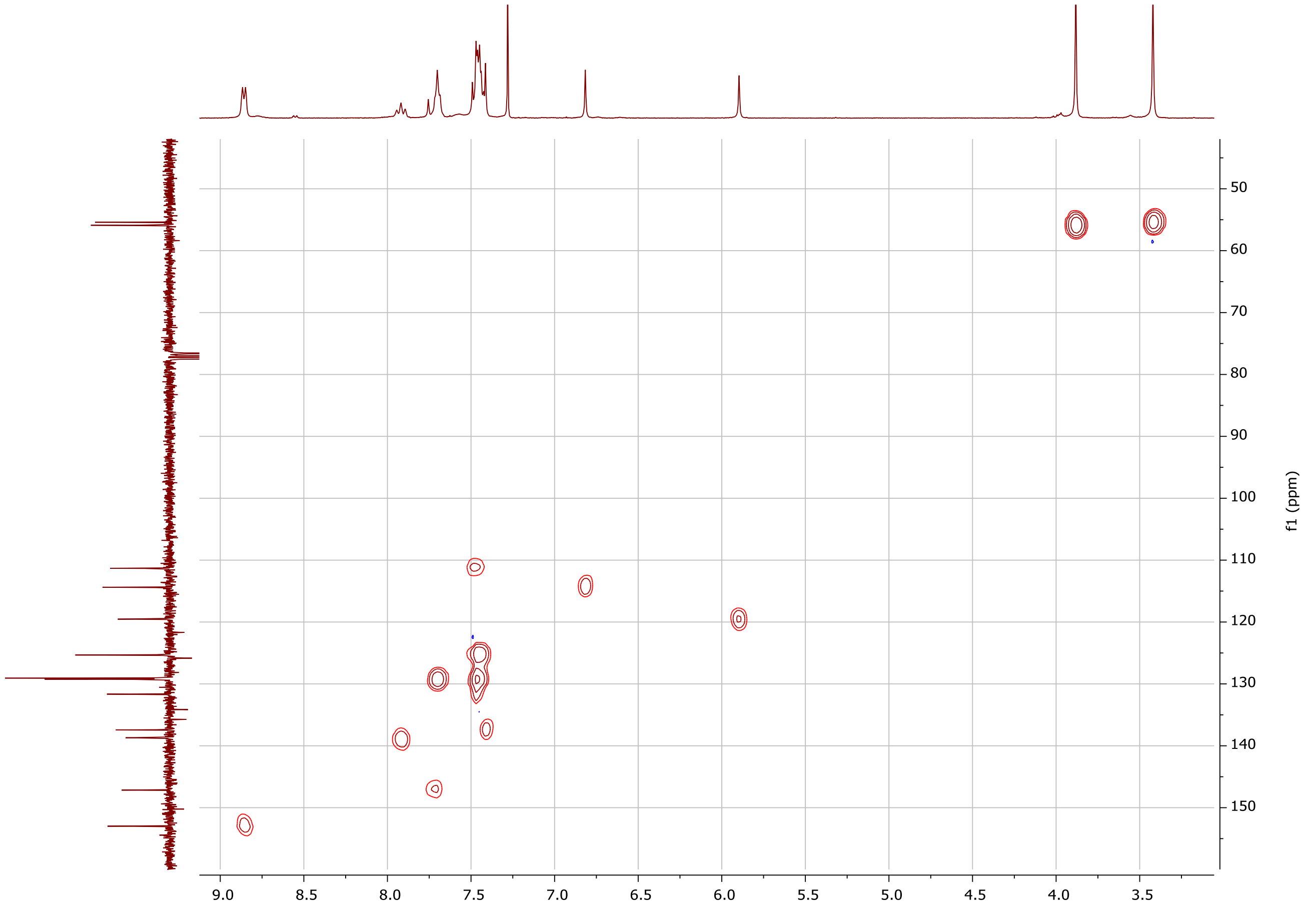

^{1}H - ^{13}C HSQC correlation spectrum of **3m**

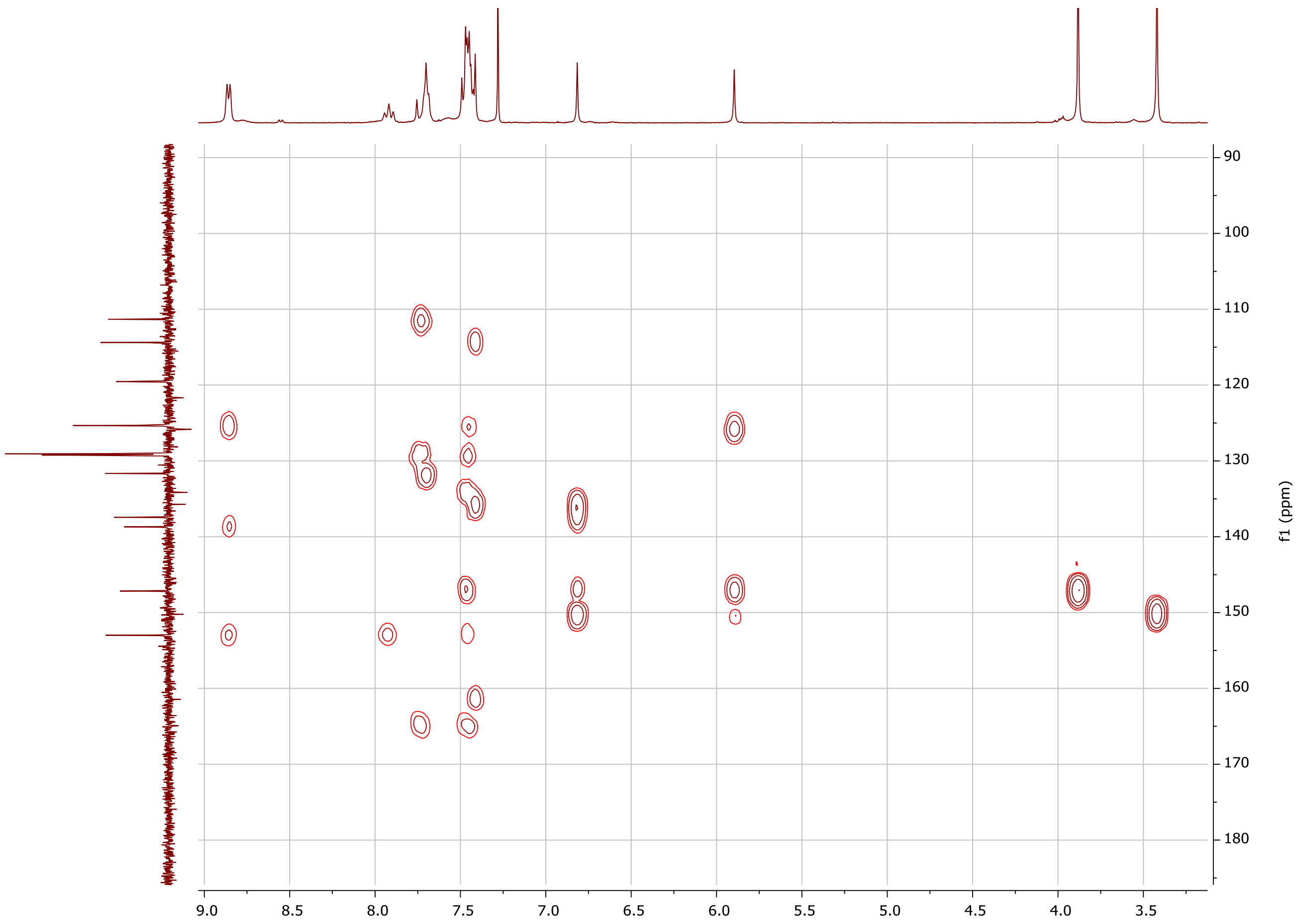

^{1}H - ^{13}C HMBC correlation spectrum of **3m**

5.- NMR spectra of mononuclear bis-pyridine orthopalladated derivatives (4)

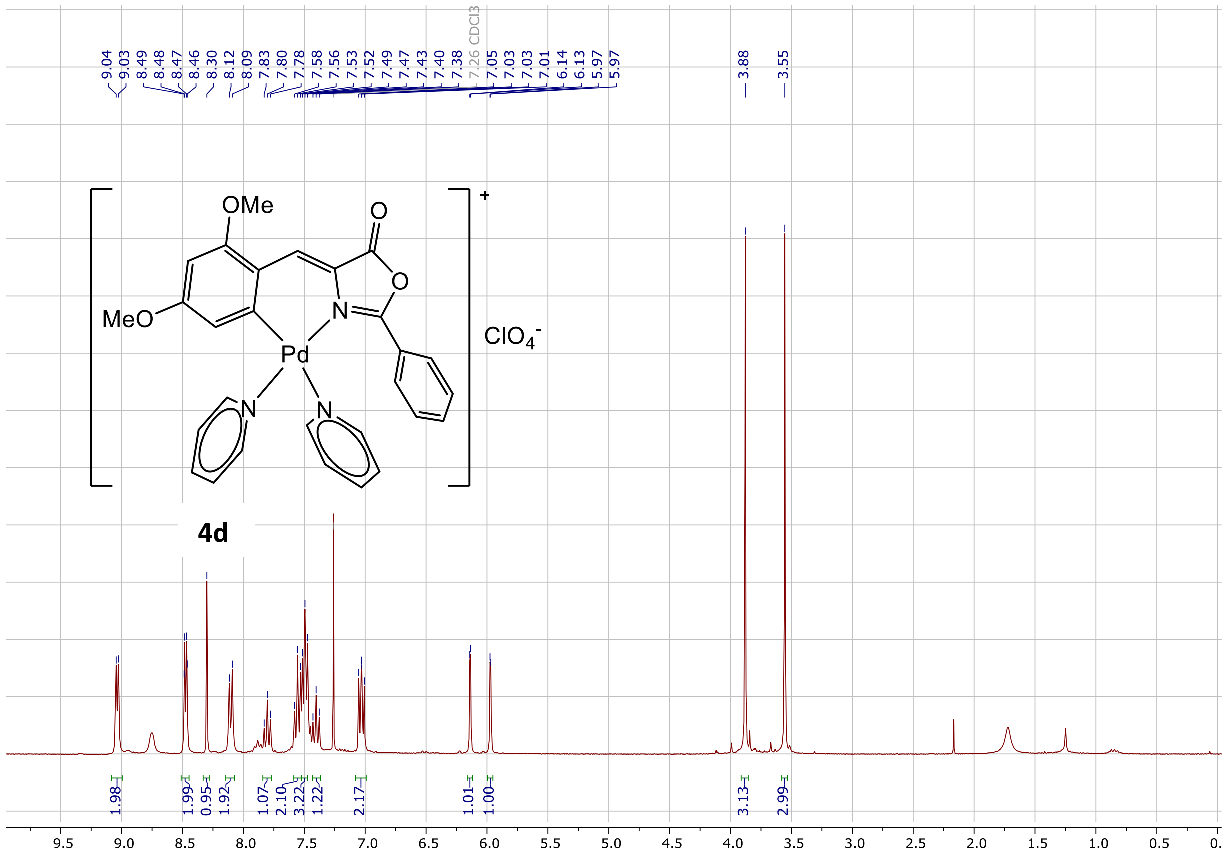

^{1}H-NMR spectrum ($CDCl_3$, 300.13 MHz) of **4d**

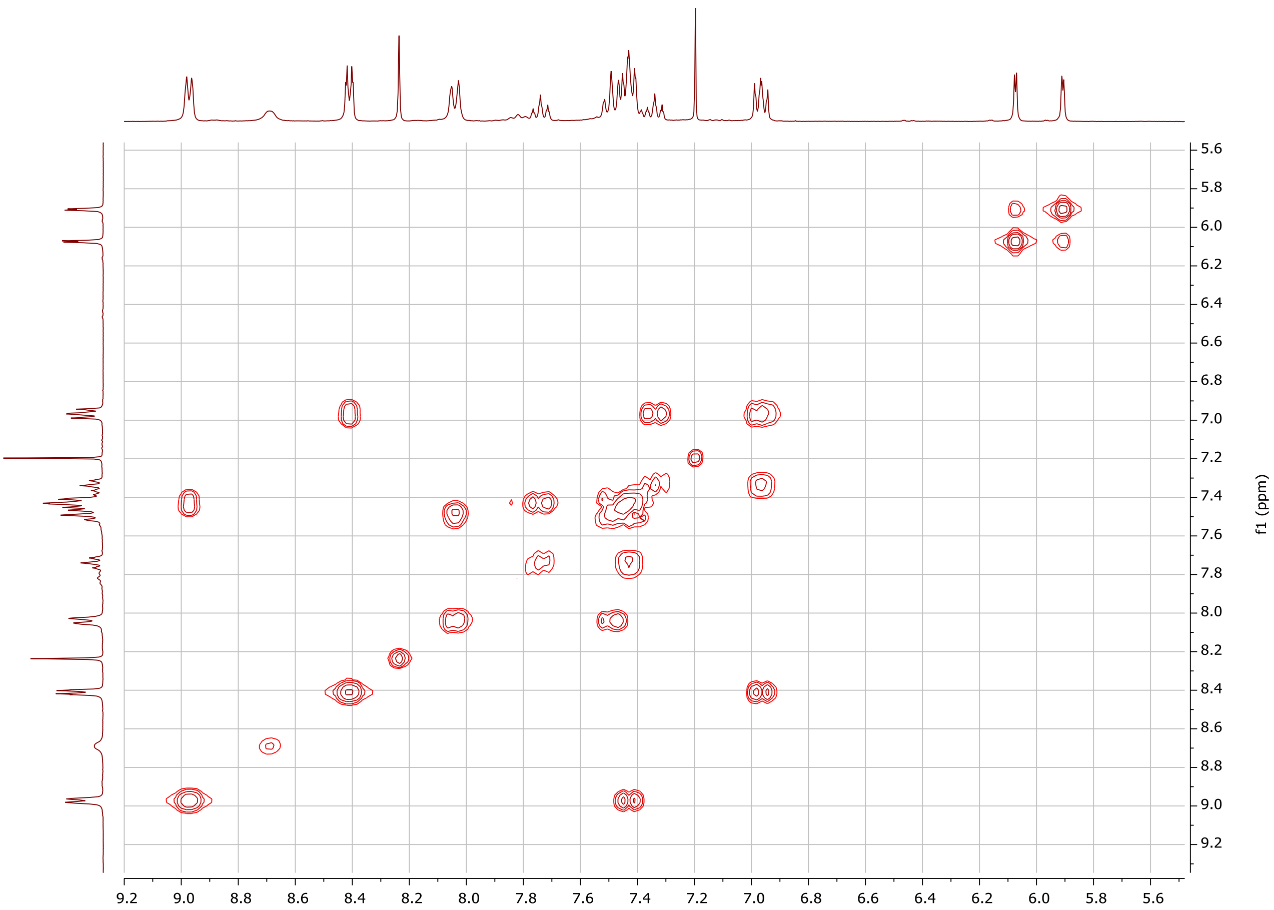

$^1H - ^1H$ COSY correlation spectrum of **4d**

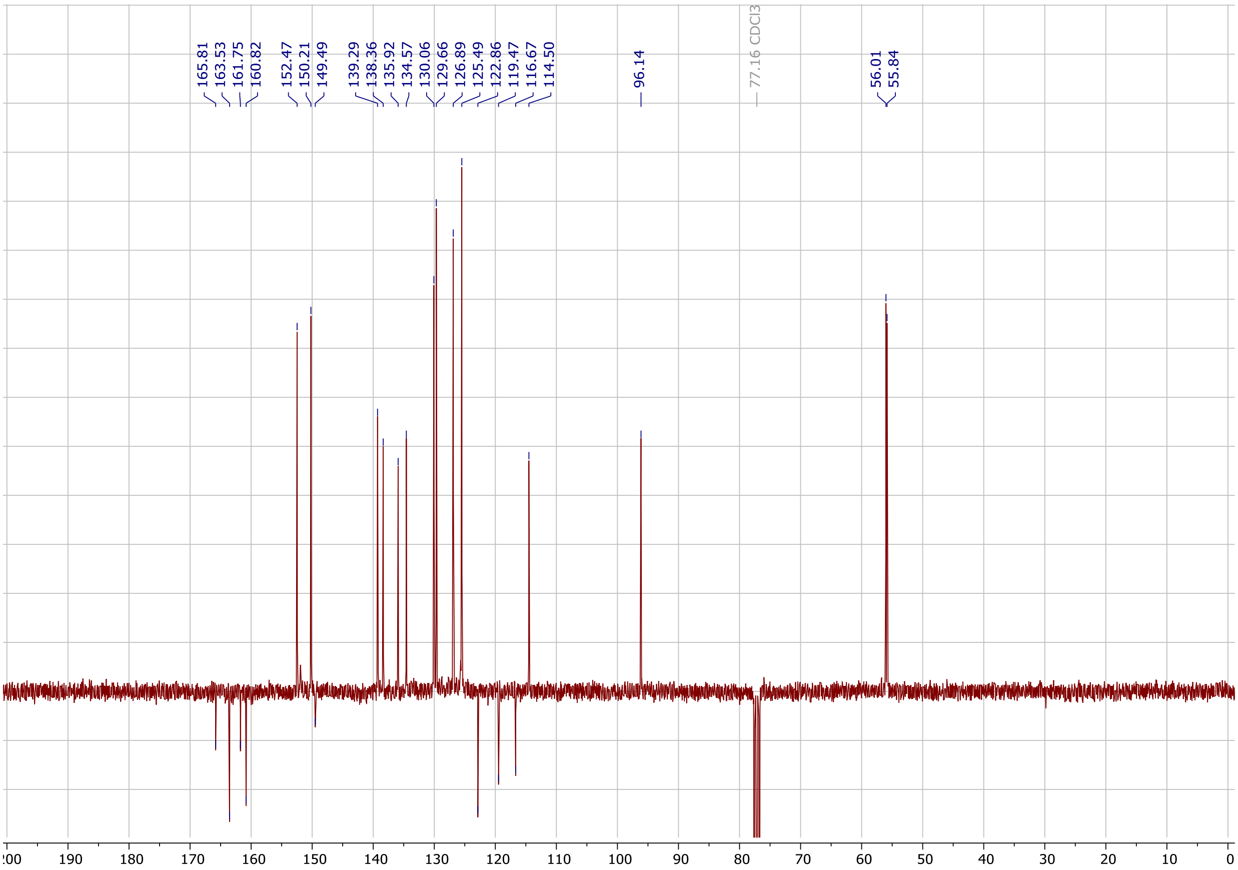

$^{13}C\{^1H\}$ (APT) NMR spectrum ($CDCl_3$, 75.47 MHz) of **4d**

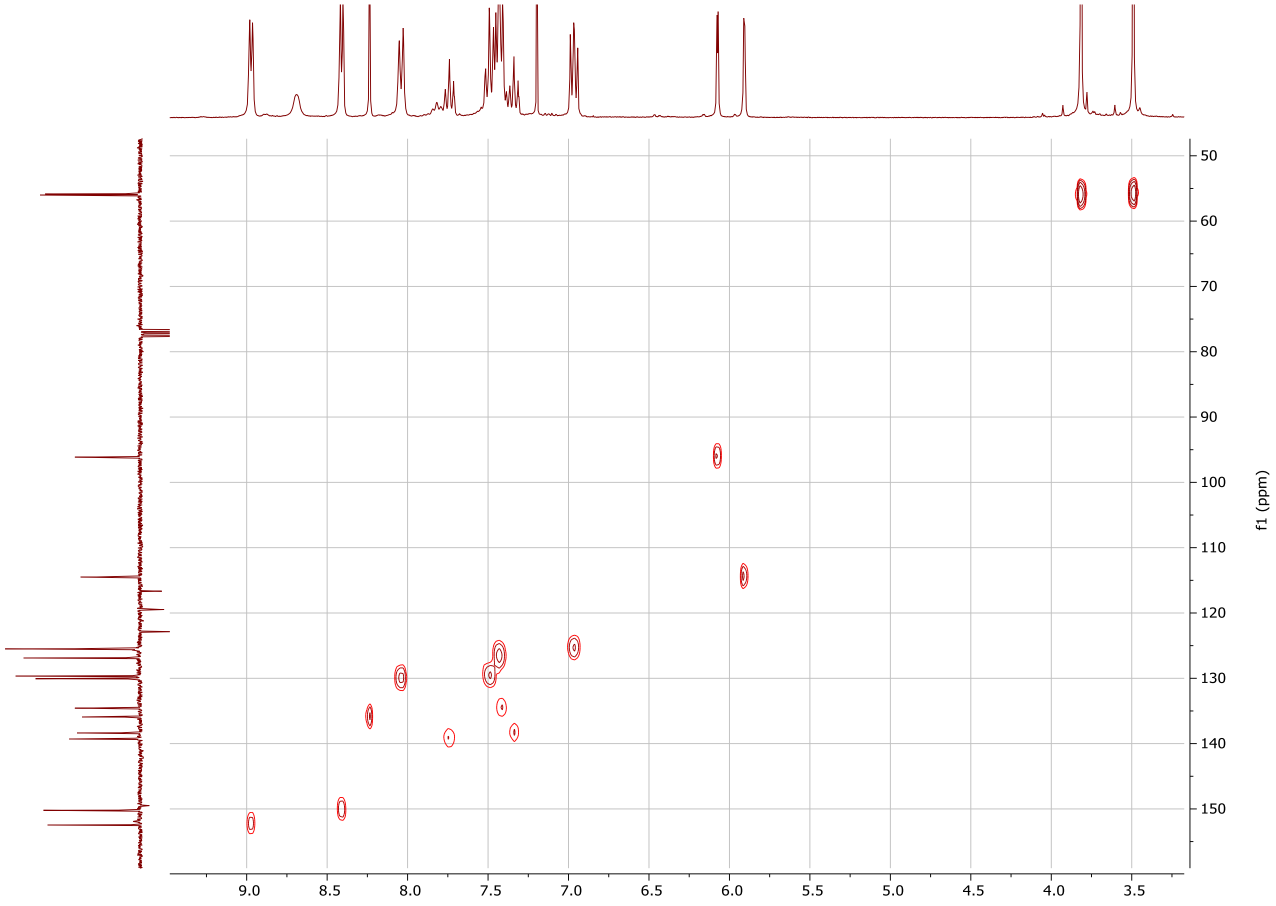

^{1}H - ^{13}C HSQC correlation spectrum of **4d**

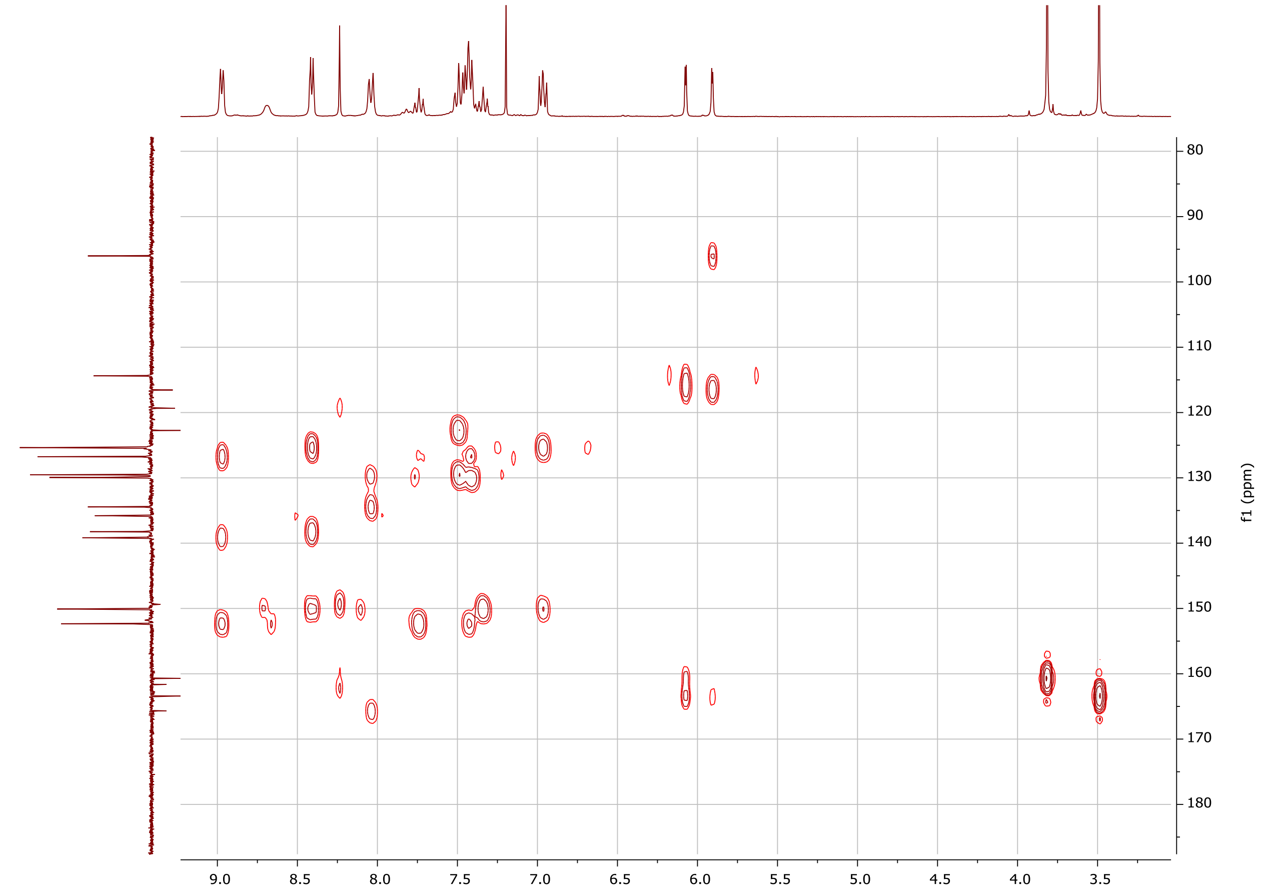

^{1}H - ^{13}C HMBC correlation spectrum of **4d**

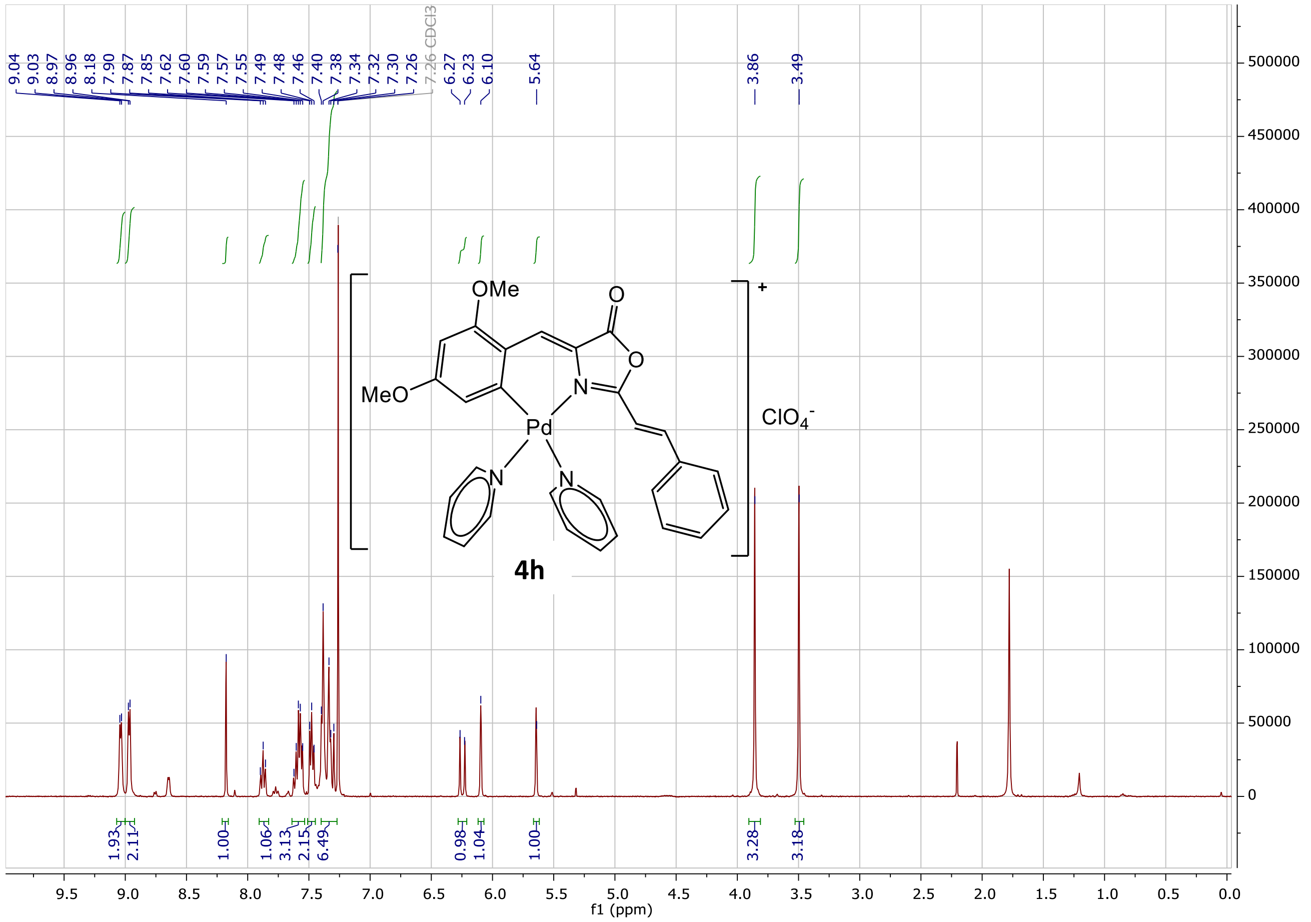

^{1}H-NMR spectrum ($CDCl_3$, 400.13 MHz, 233 K) of **4h**

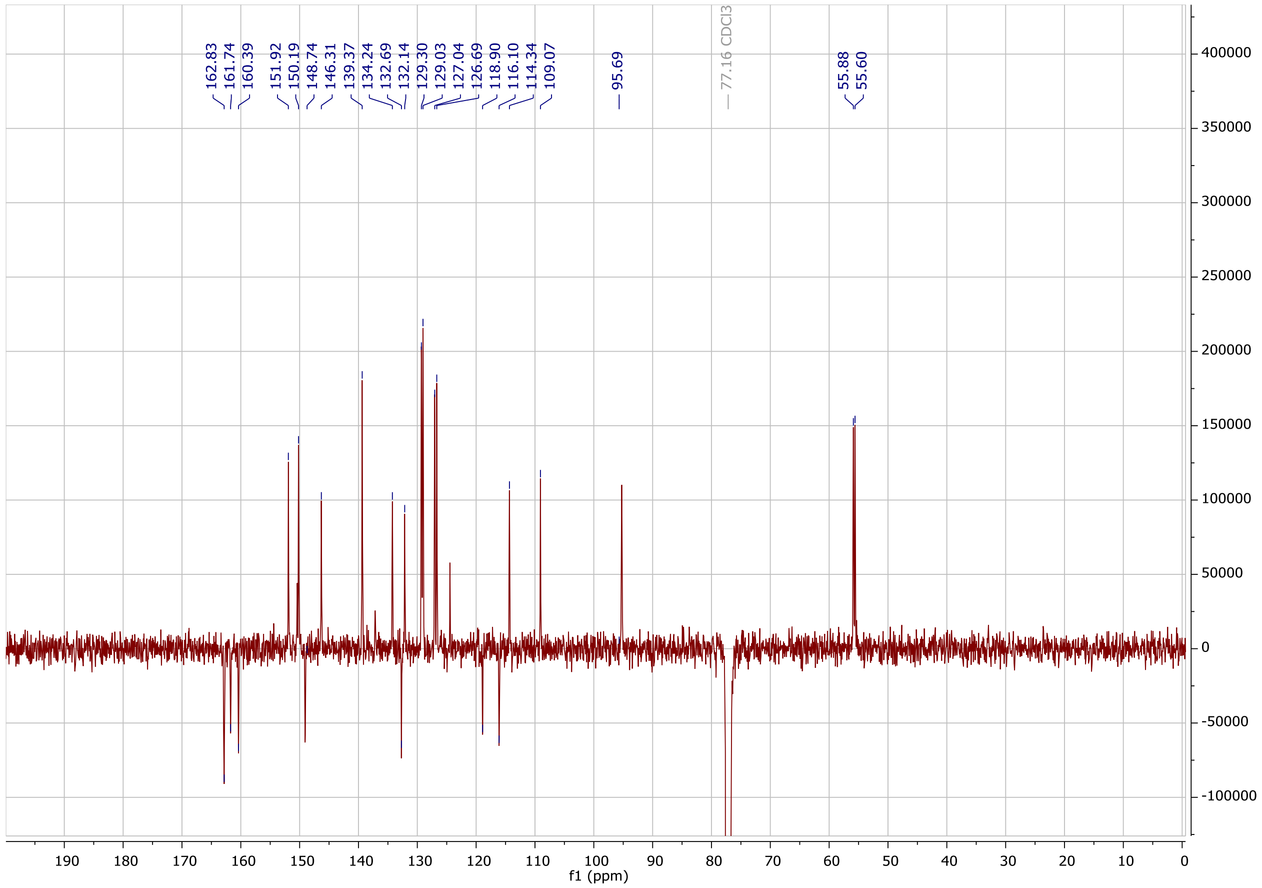

^{13}C{^{1}H} (APT) NMR spectrum ($CDCl_3$, 100.6 MHz, 233 K) of **4h**

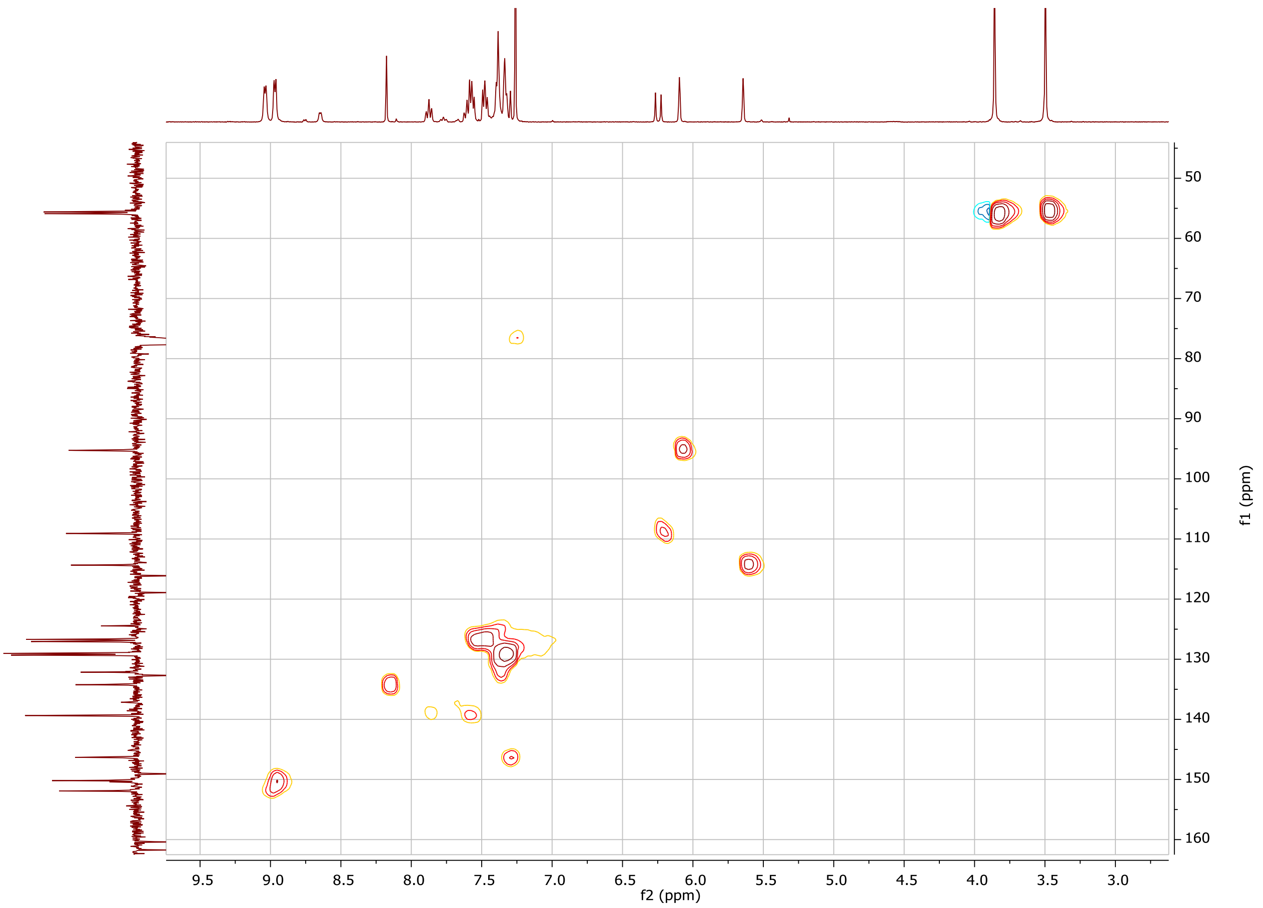

^{1}H - ^{13}C HSQC correlation spectrum of **4h**

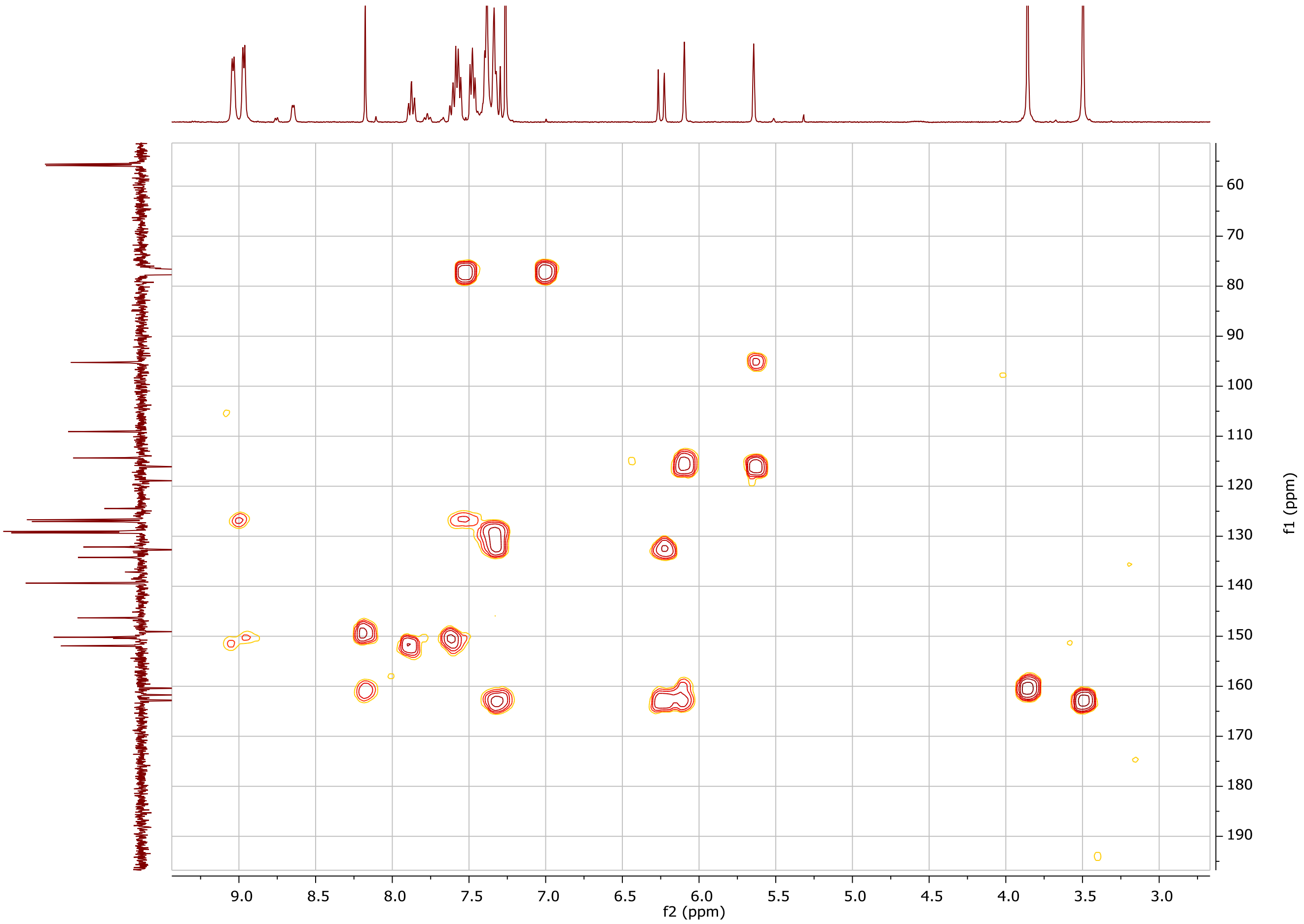

^{1}H - ^{13}C HMBC correlation spectrum of **4h**

6.- NMR spectra of mononuclear orthopalladated derivatives **(5)** and **(6)**

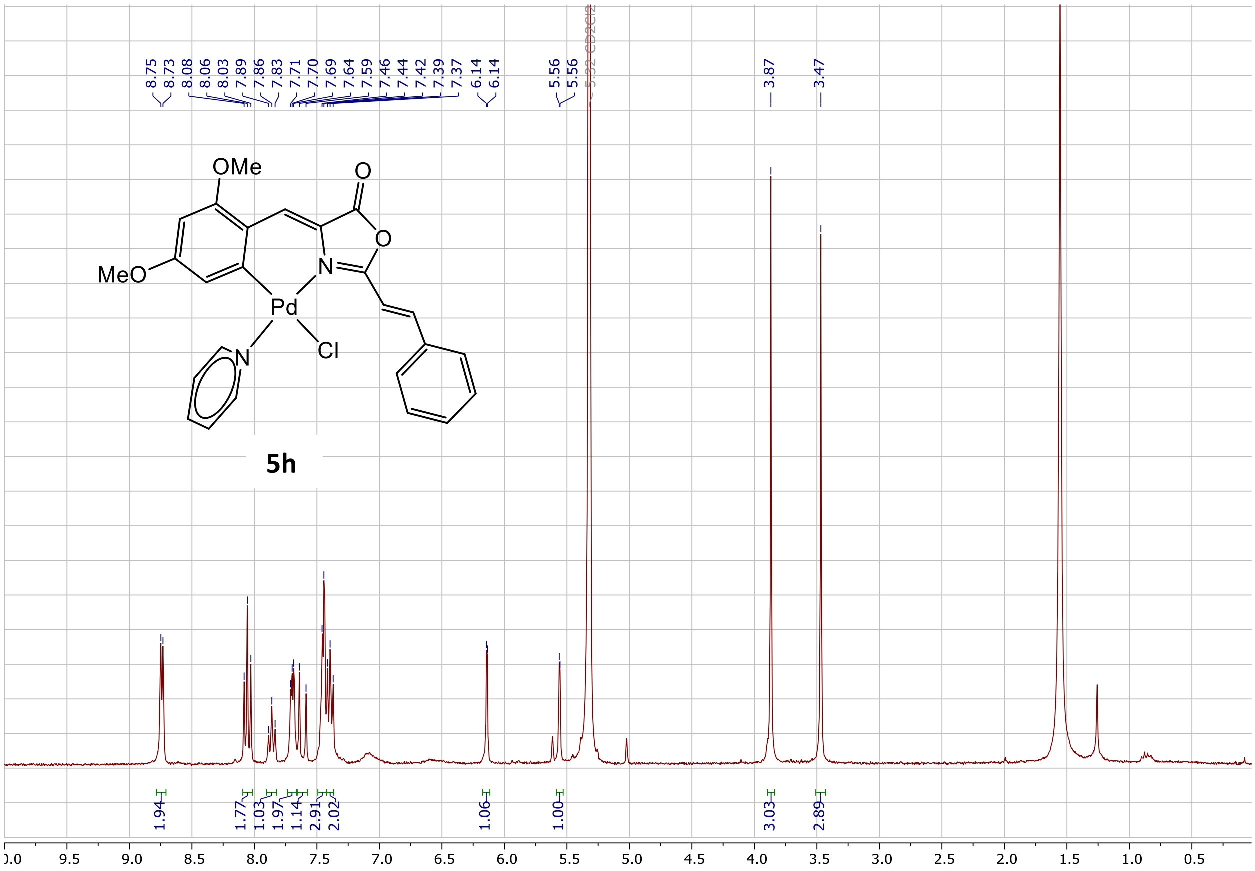

^{1}H-NMR spectrum (CD_2Cl_2, 300.13 MHz) of **5h**

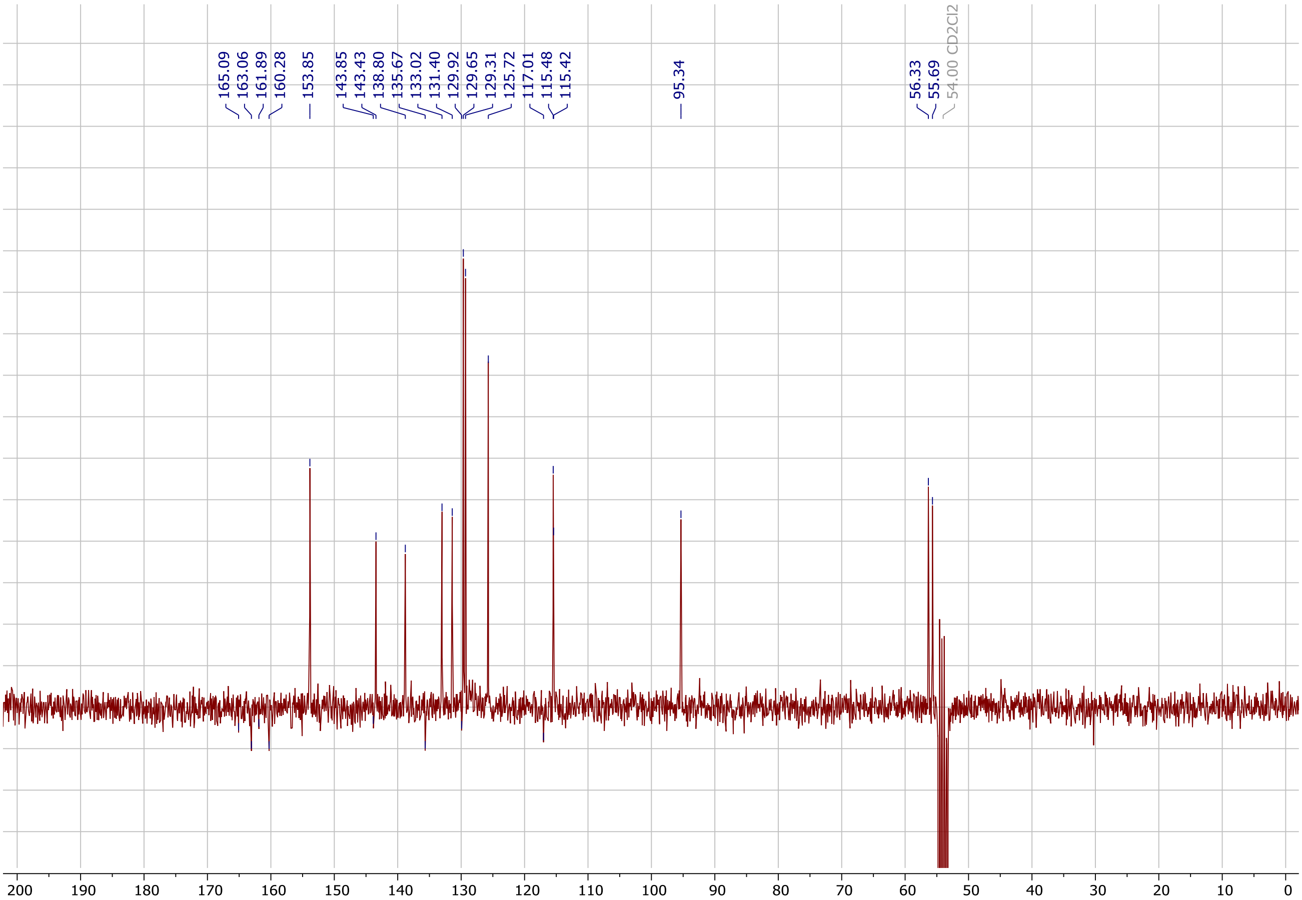

$^{13}C\{^1H\}$ (APT) NMR spectrum (CD_2Cl_2, 75.47 MHz) of **5h**

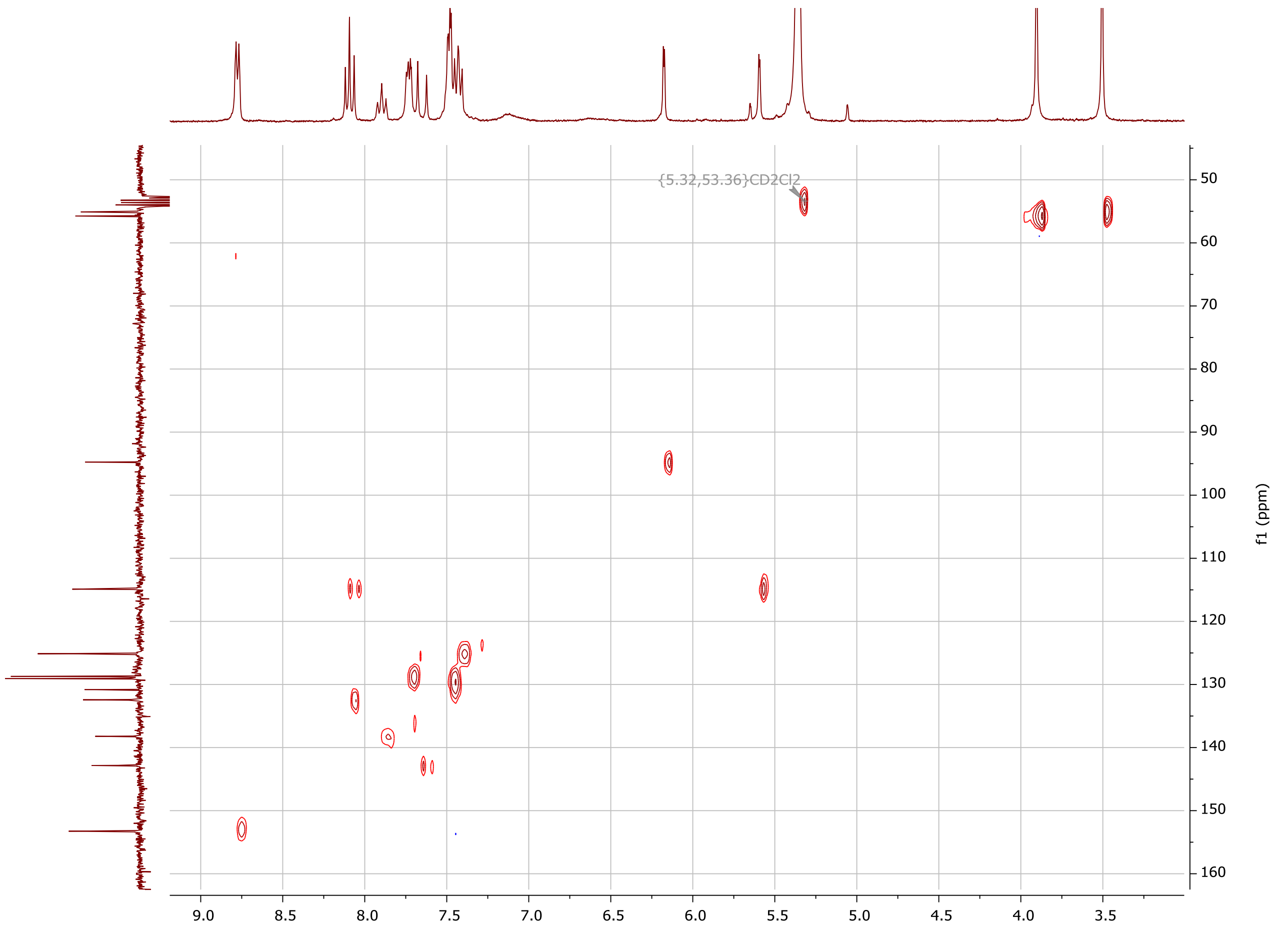

^{1}H - ^{13}C HSQC correlation spectrum of **5h**

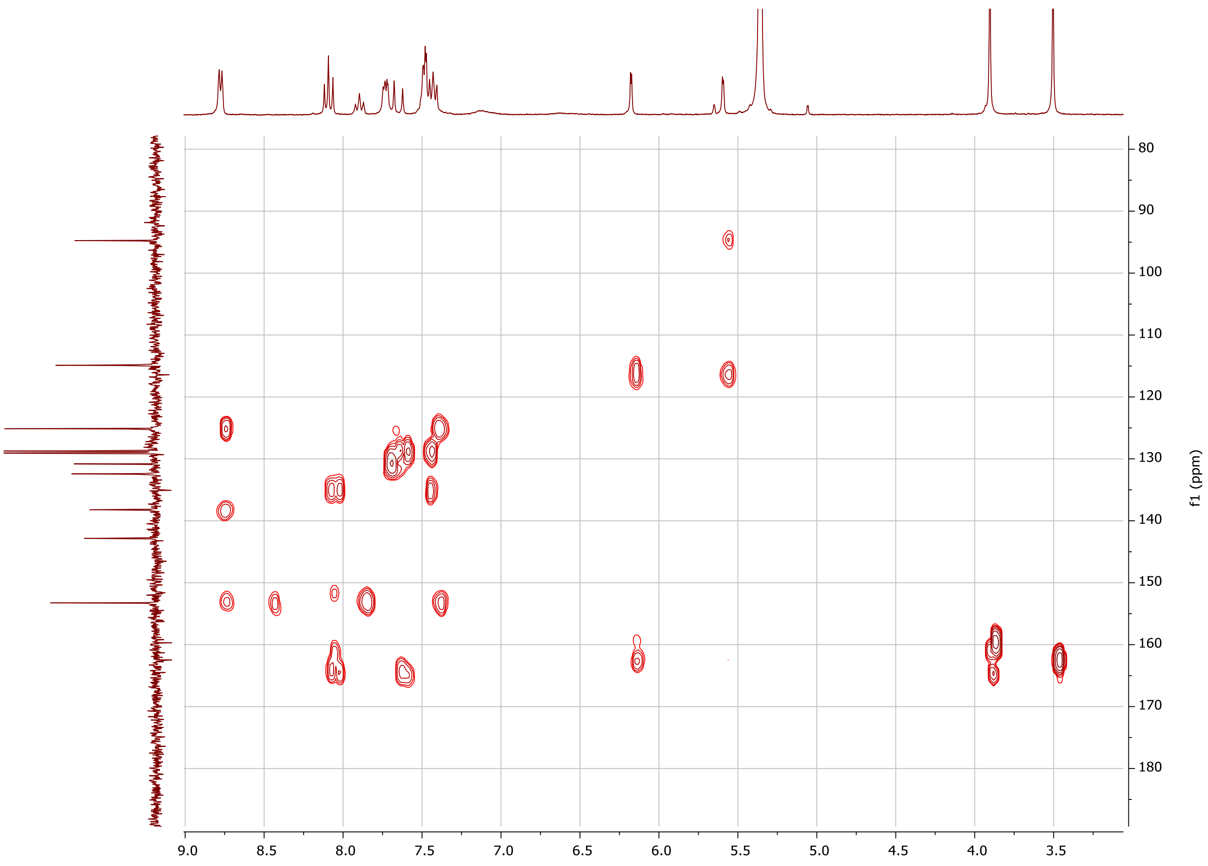

^{1}H - ^{13}C HMBC correlation spectrum of **5h**

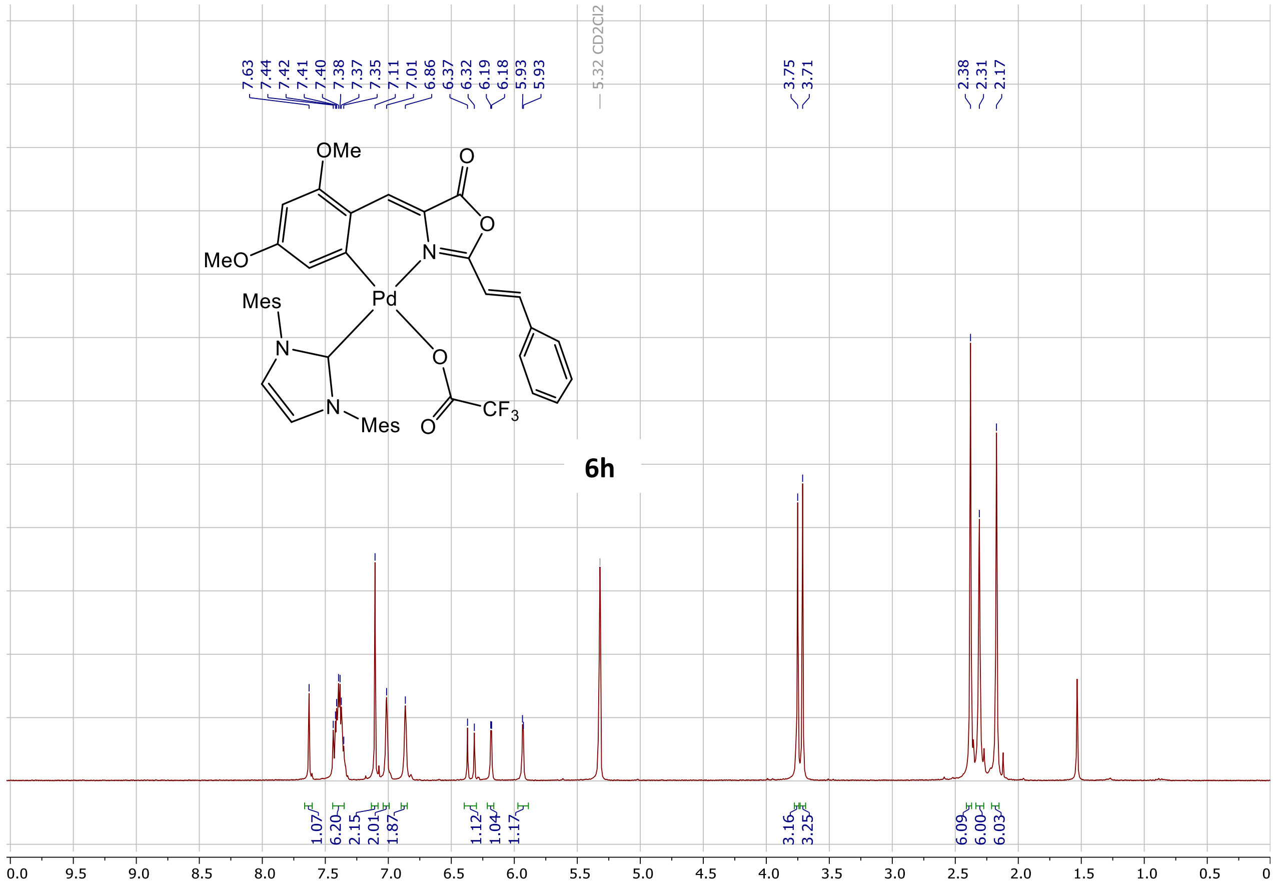

^{1}H-NMR spectrum (CD_2Cl_2, 300.13 MHz) of **6h**

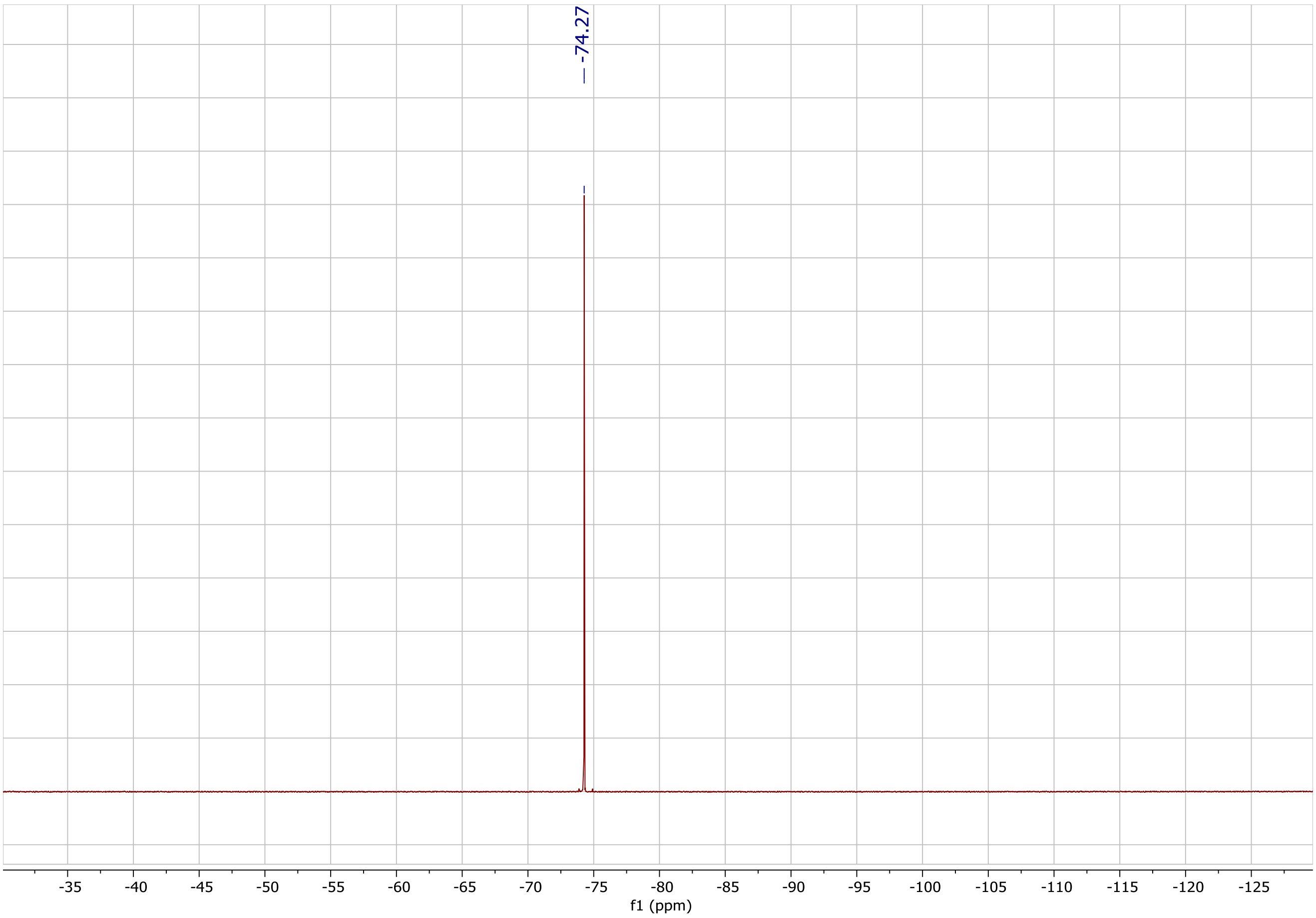

^{19}F-NMR spectrum (CD_2Cl_2, 282.40 MHz) of **6h**

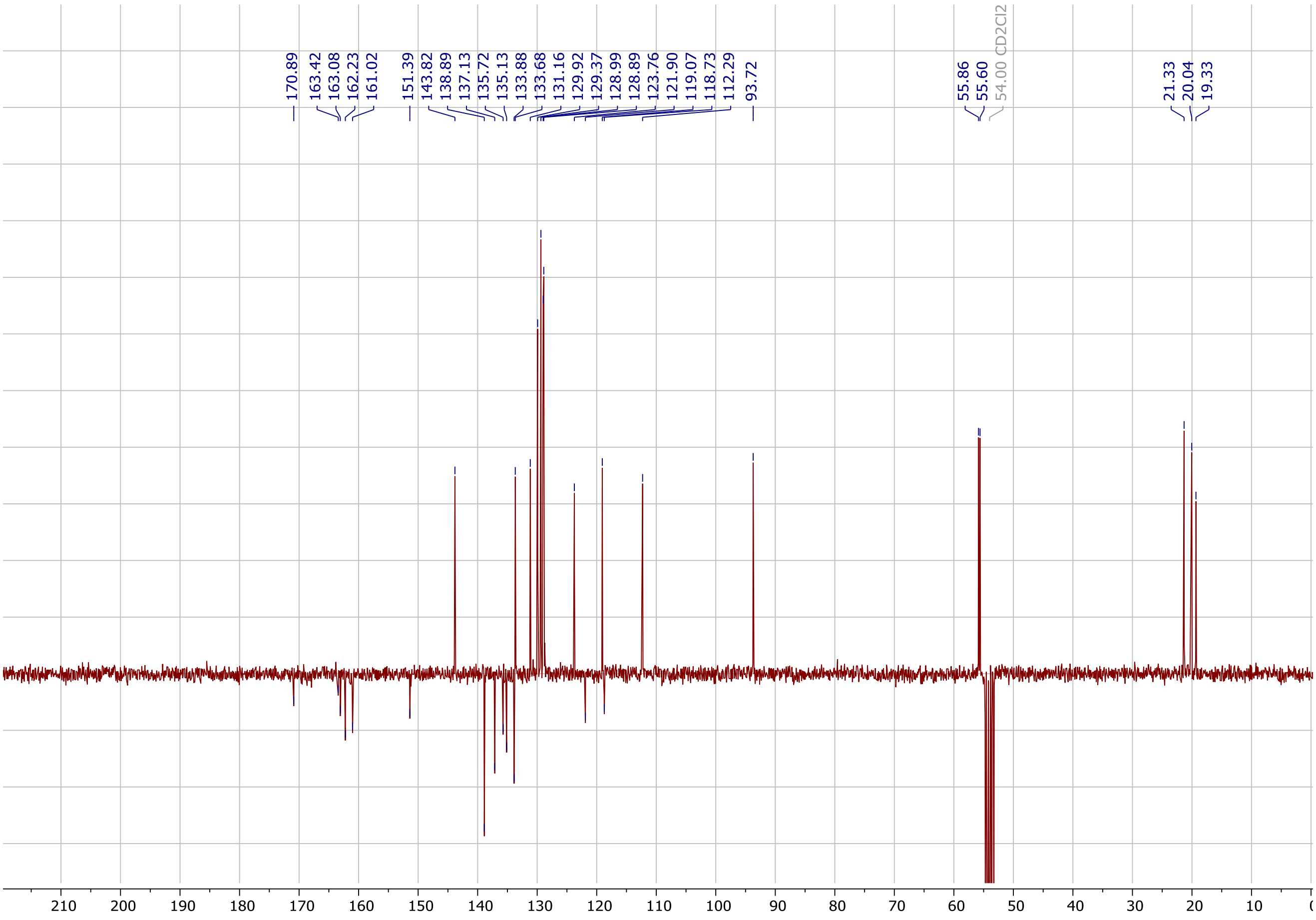

$^{13}C\{^{1}H\}$ (APT) NMR spectrum (CD_2Cl_2, 75.47 MHz) of **6h**

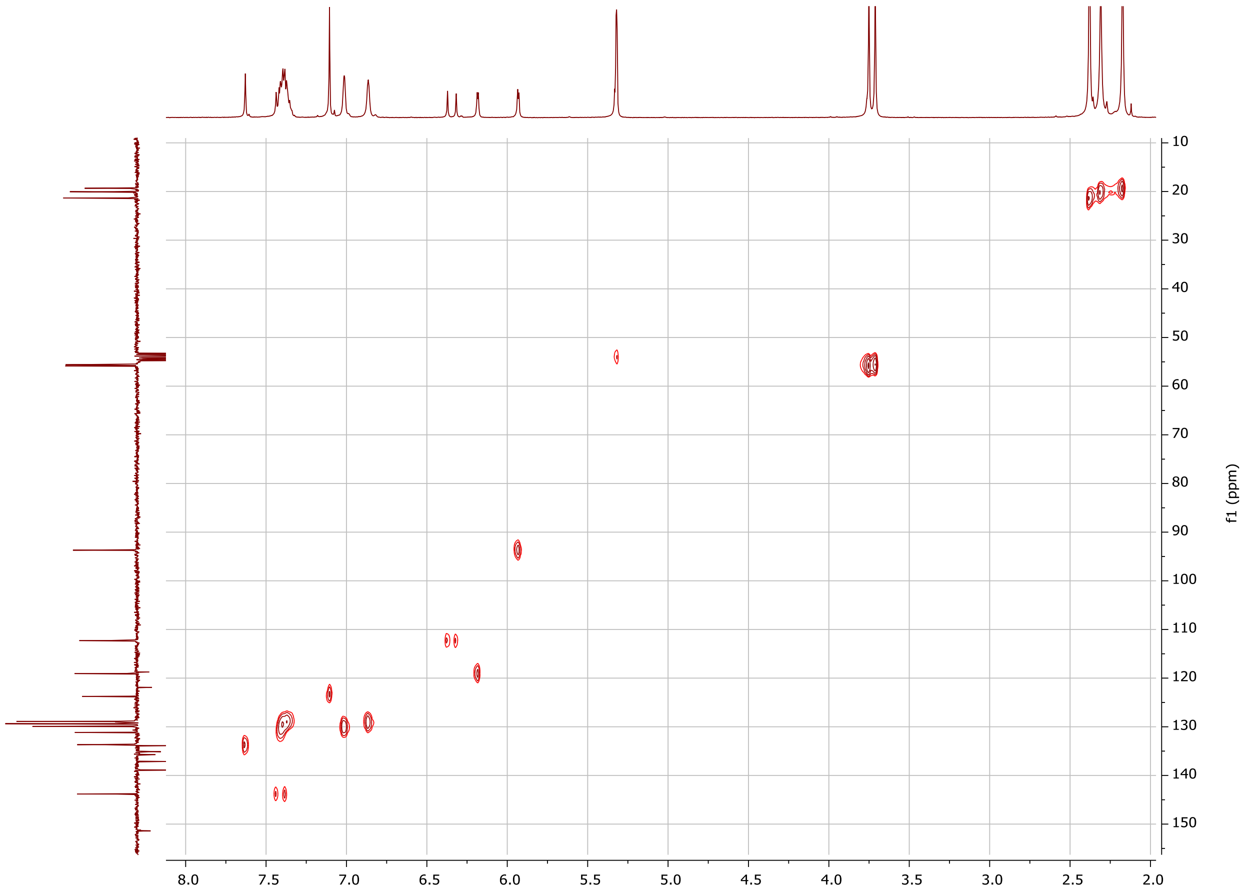

^{1}H - ^{13}C HSQC correlation spectrum of **6h**

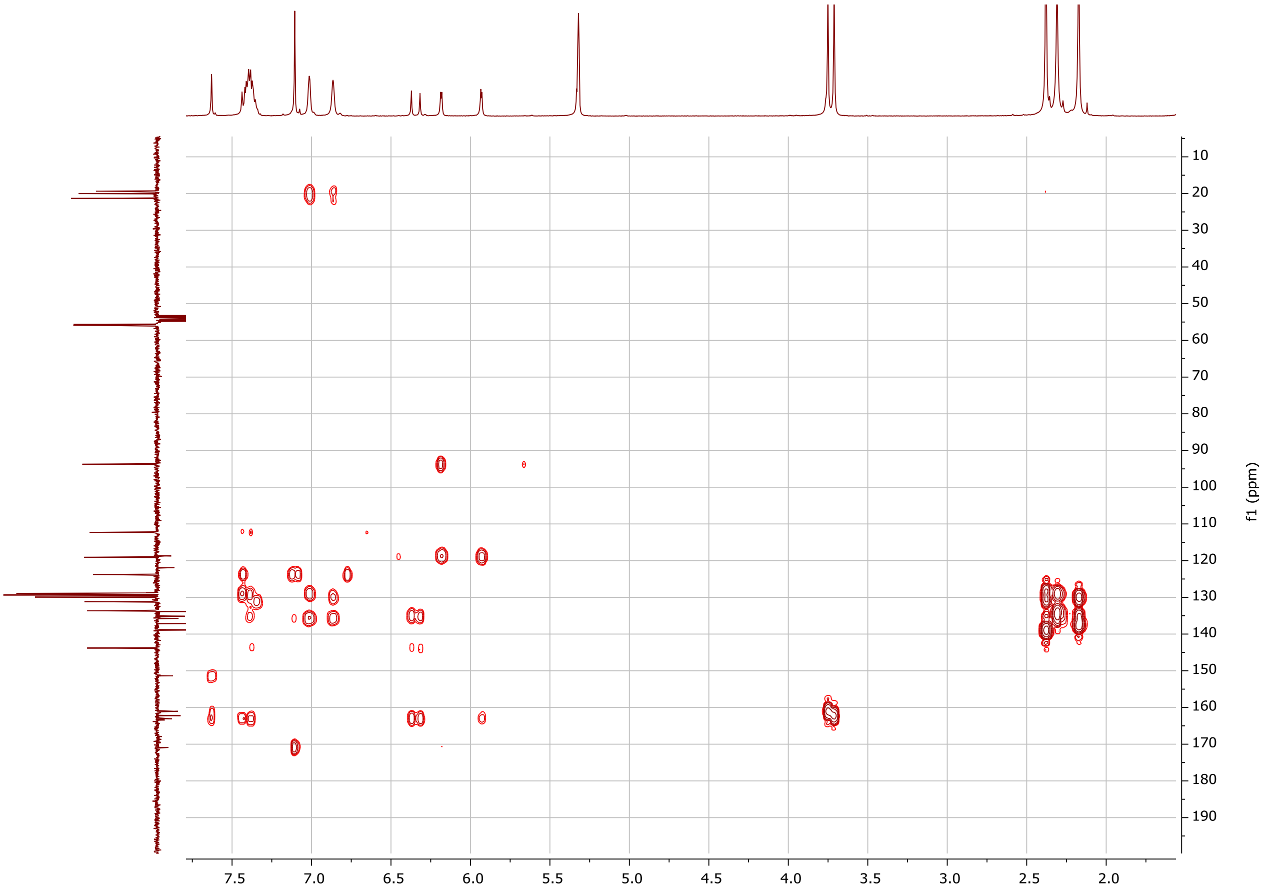

^{1}H - ^{13}C HMBC correlation spectrum of **6h**

7. ABSORPTION UV-VIS SPECTRA

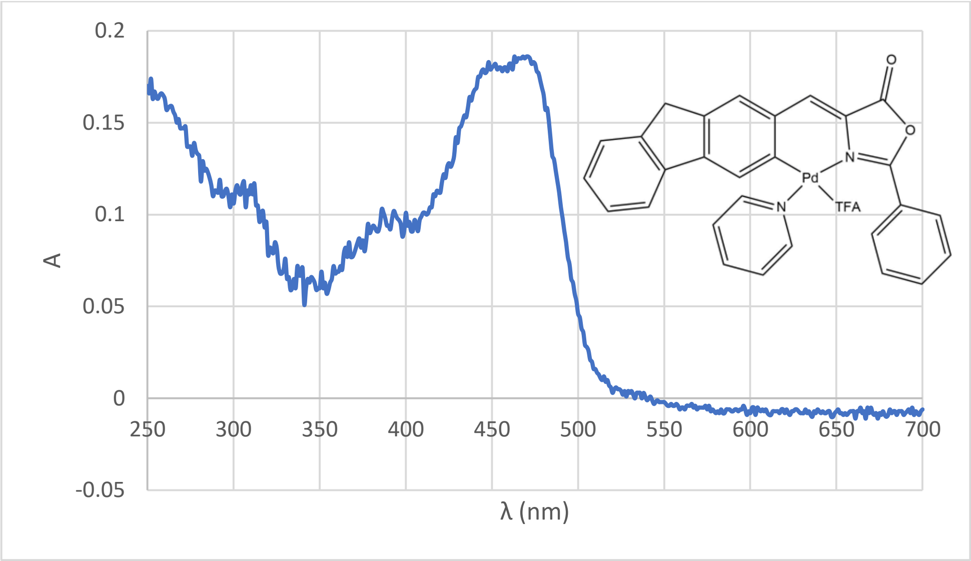

Absorption spectrum **3a**

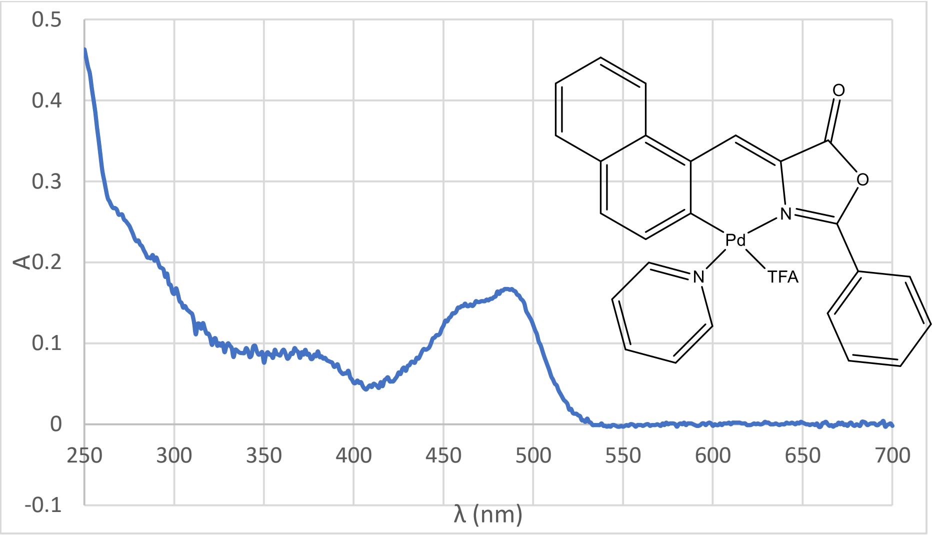

Absorption spectrum **3b**

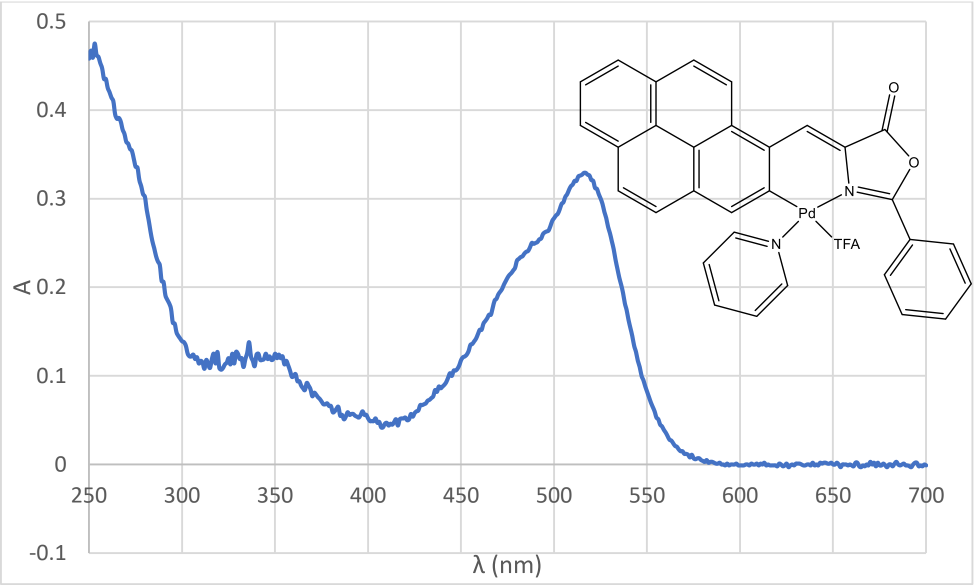

Absorption spectrum **3c**

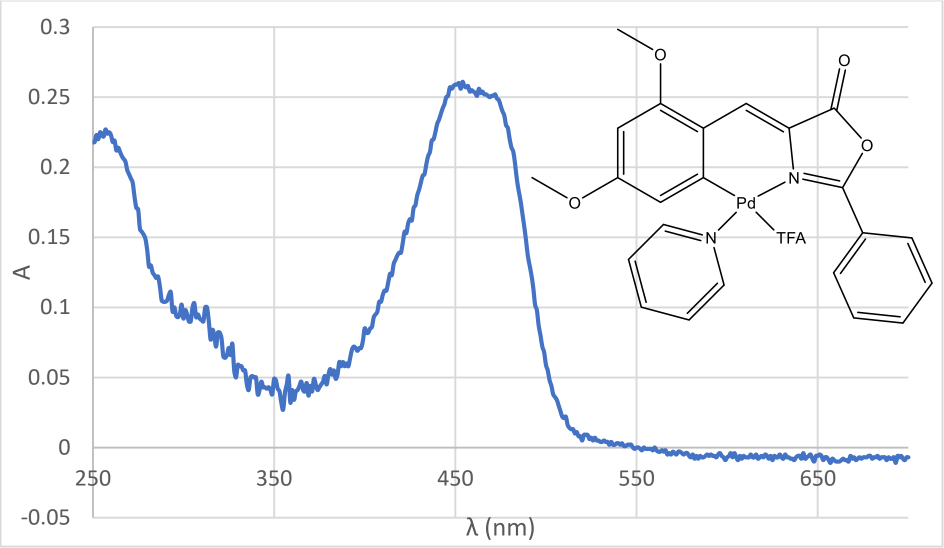

Absorption spectrum **3d**

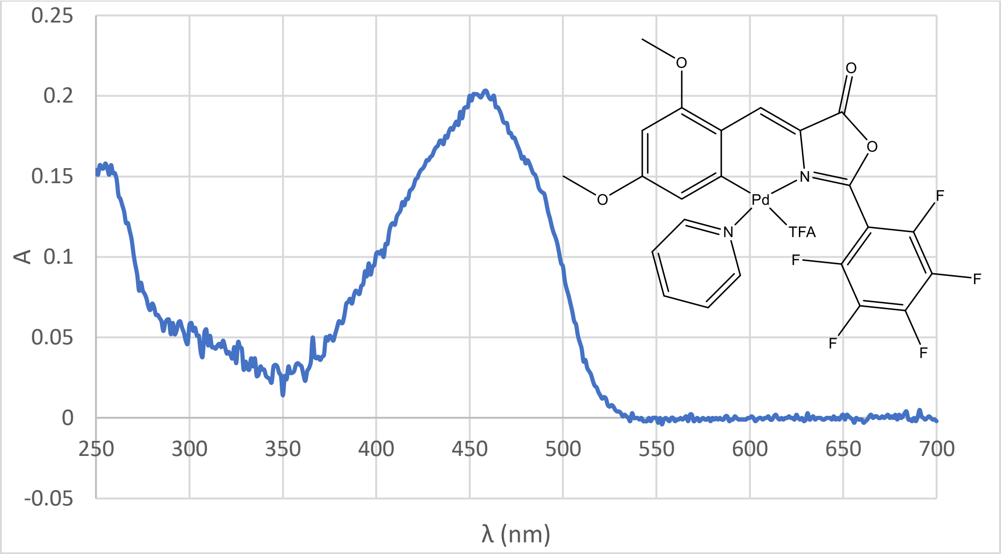

Absorption spectrum **3e**

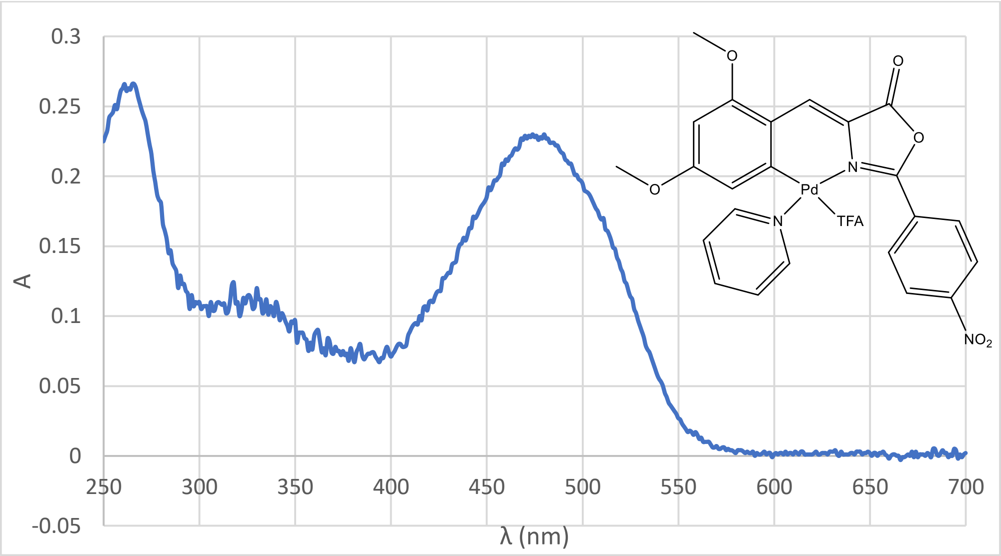

Absorption spectrum **3f**

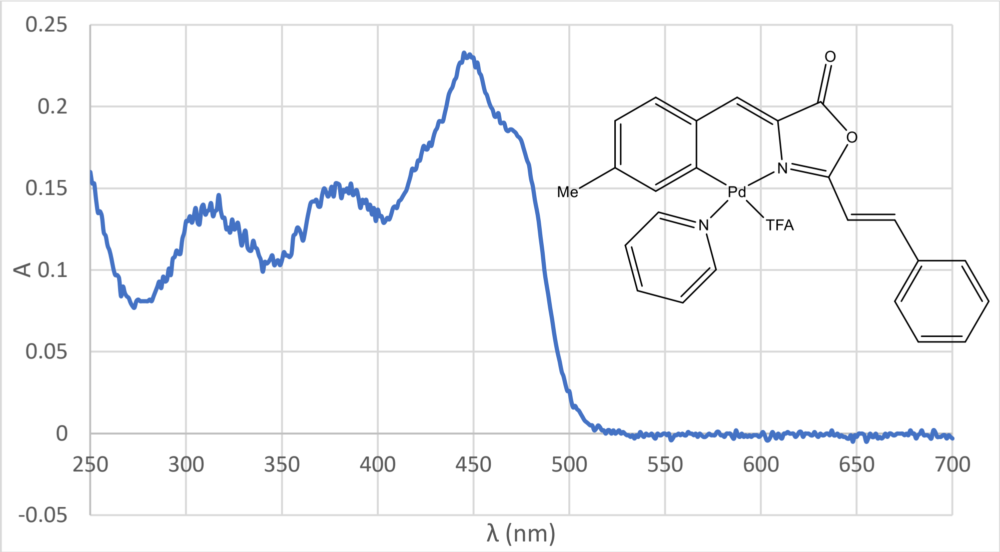

Absorption spectrum **3g**

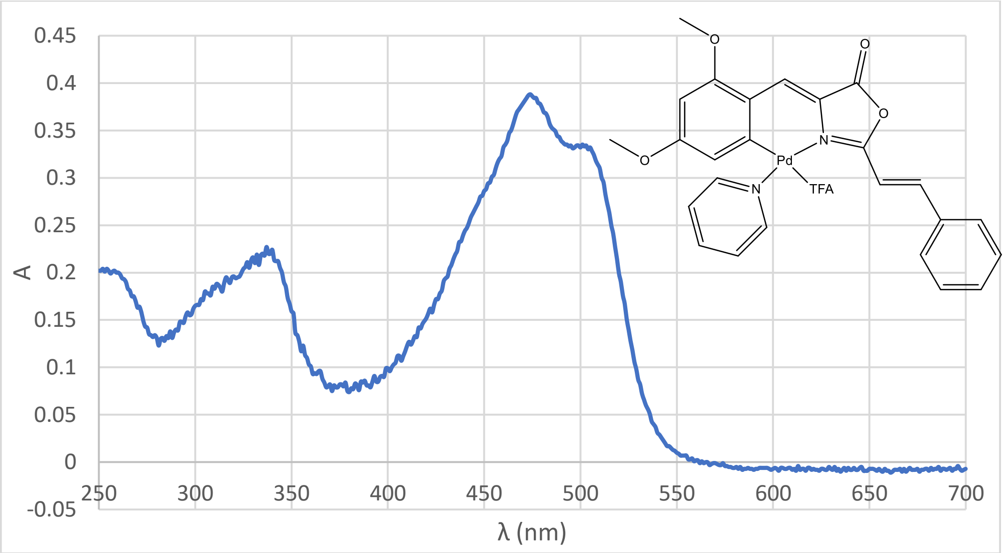

Absorption spectrum **3h**

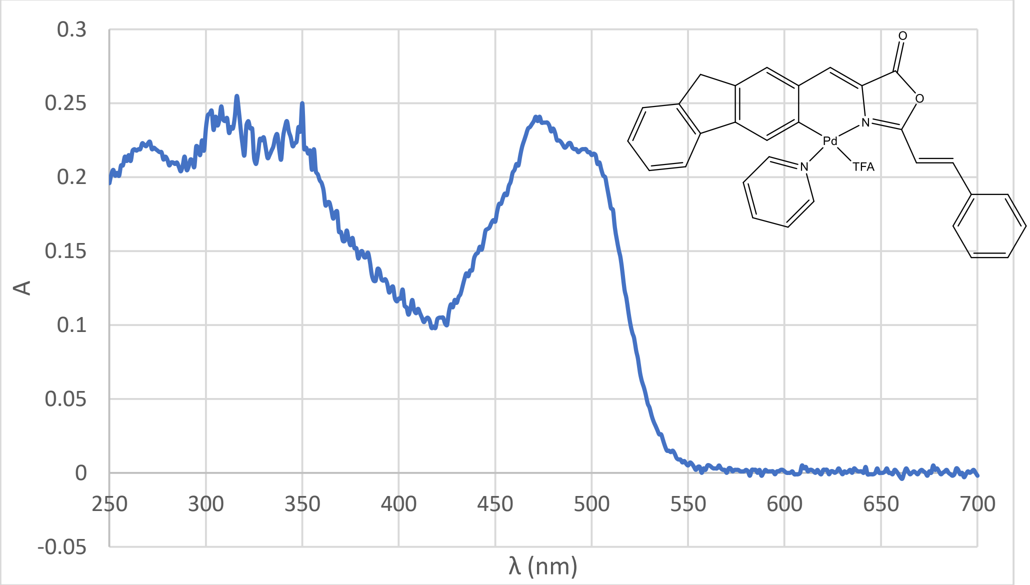

Absorption spectrum **3i**

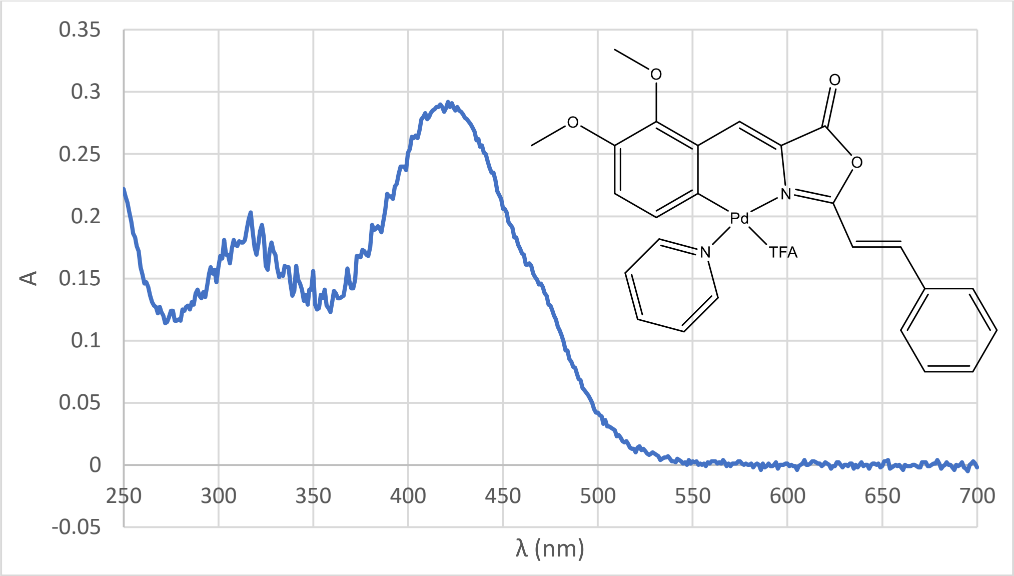

Absorption spectrum **3j**

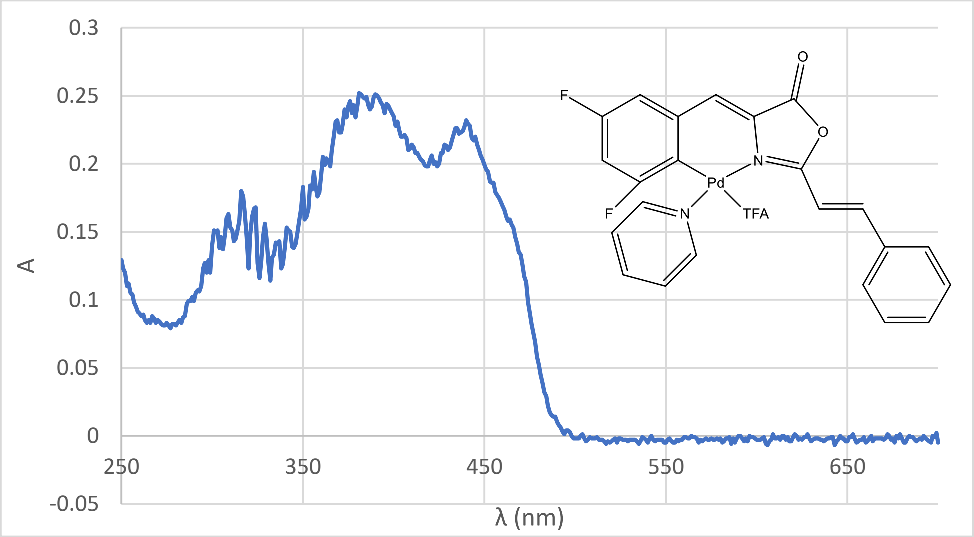

Absorption spectrum **3k**

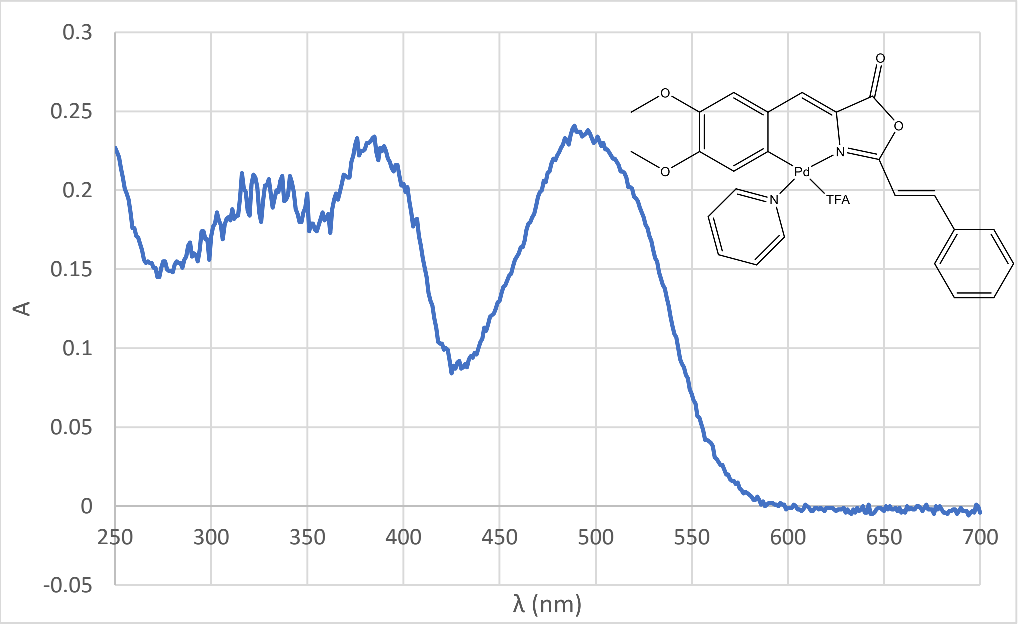

Absorption spectrum **3m**

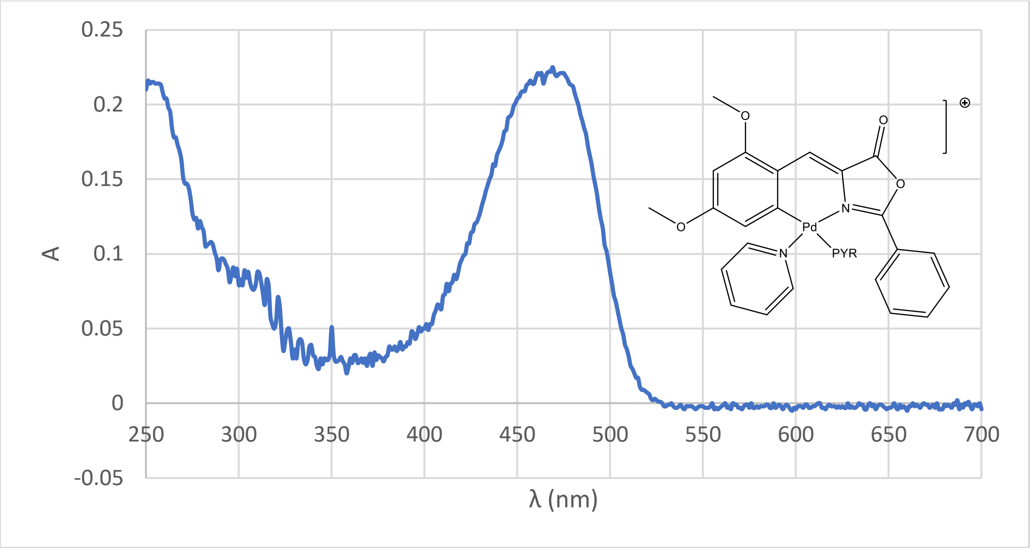

Absorption spectrum **4d**

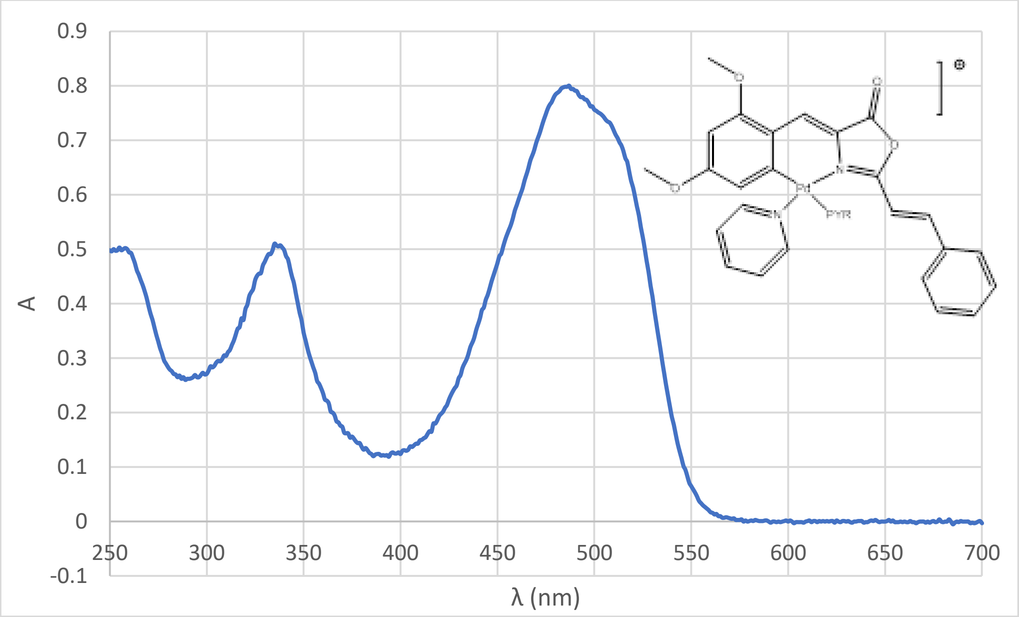

Absorption spectrum **4h**

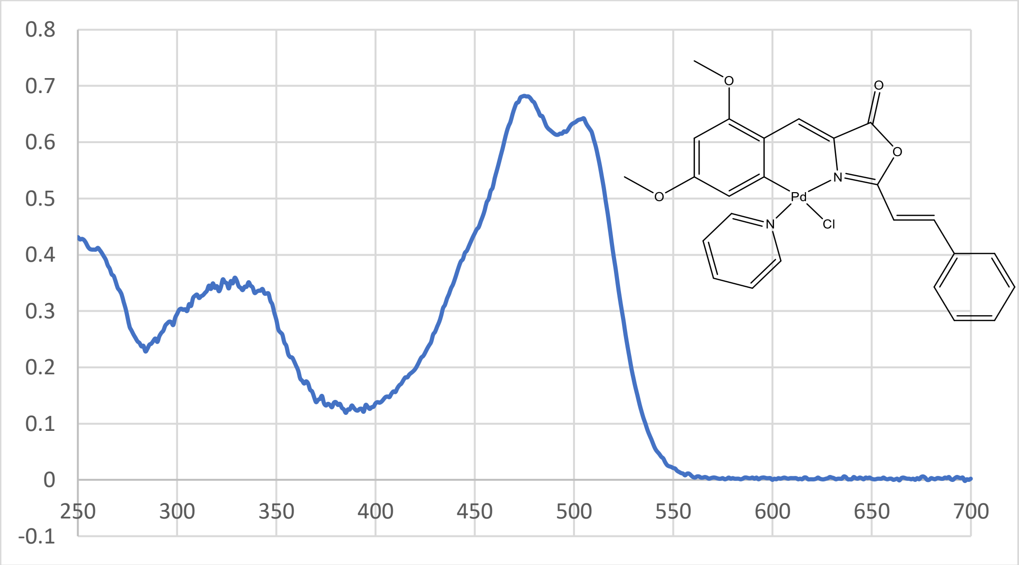

Absorption spectrum **5h**

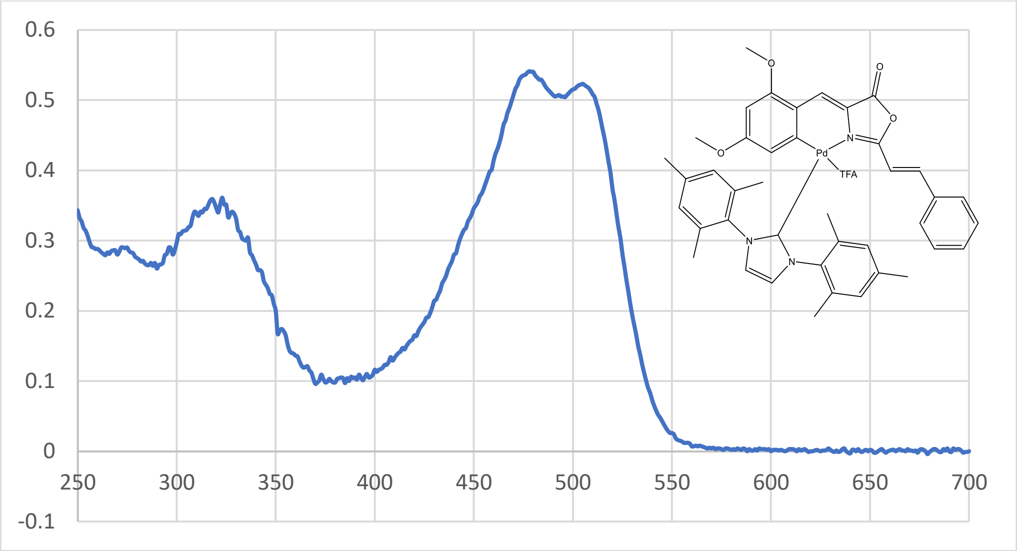

Absorption spectrum **6h**

8. EXCITATION-EMISSION UV-VIS SPECTRA

Complex **3a**

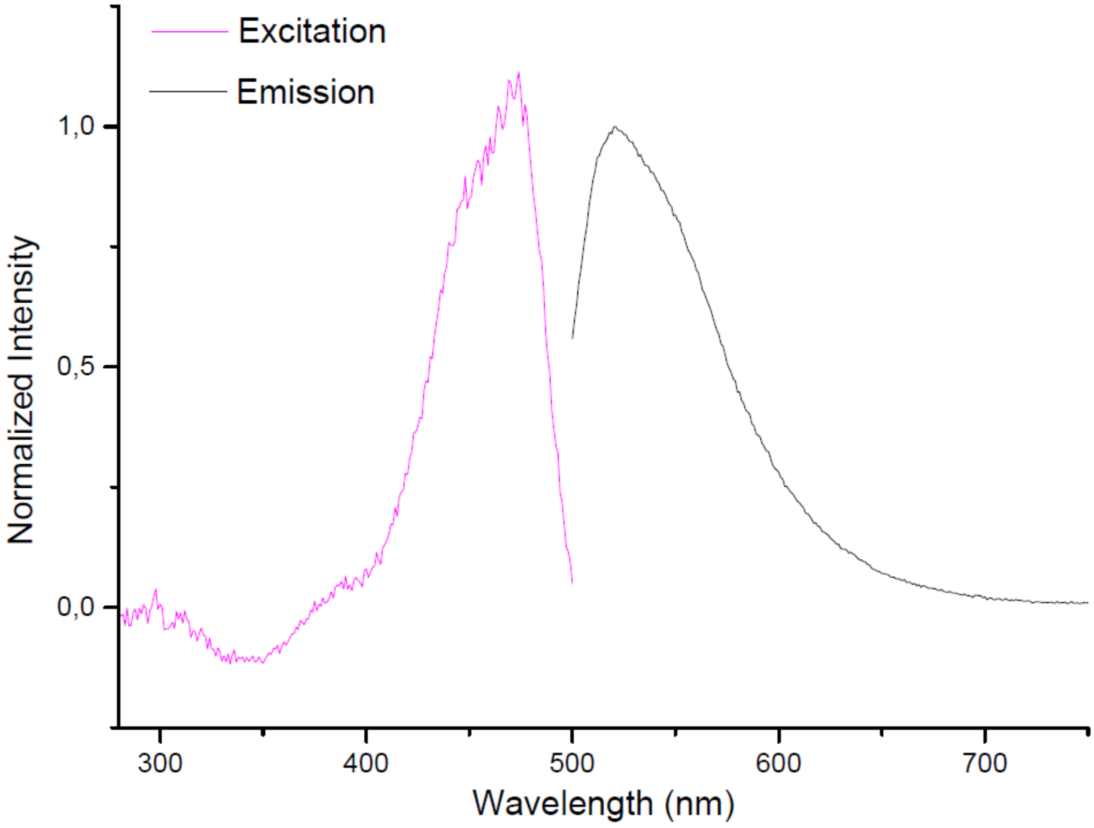

Complex **3b**

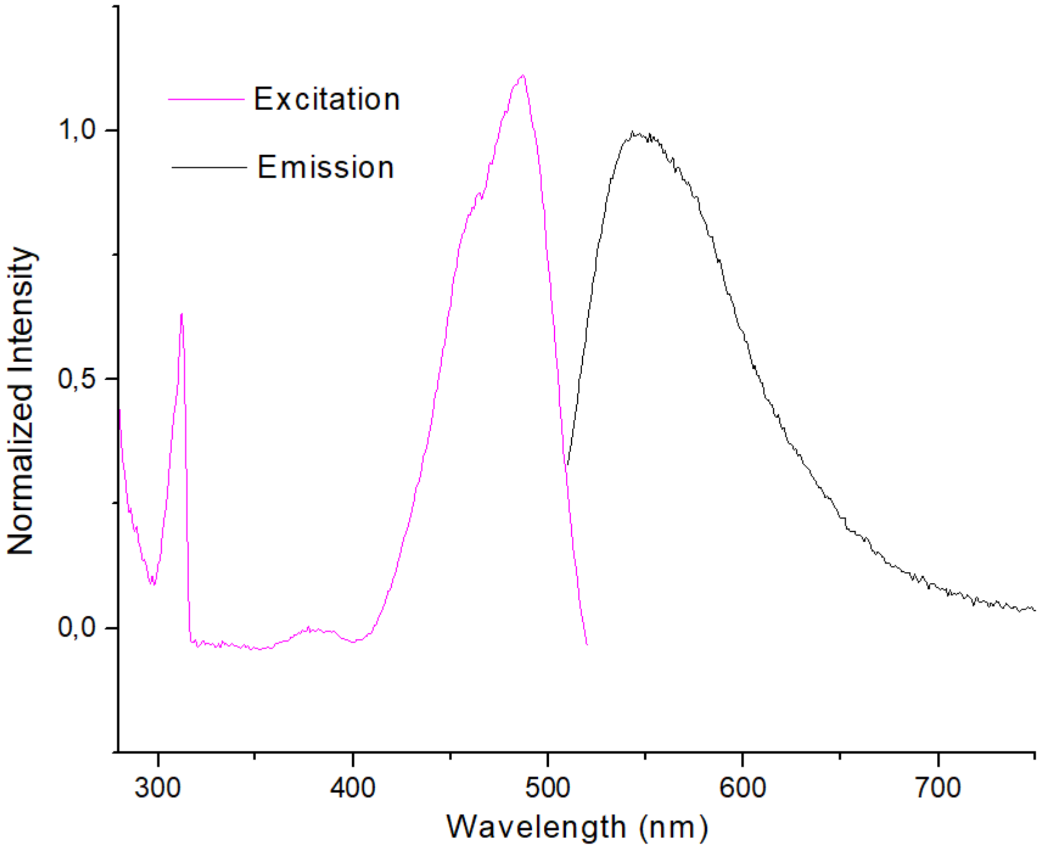

Complex **3c**

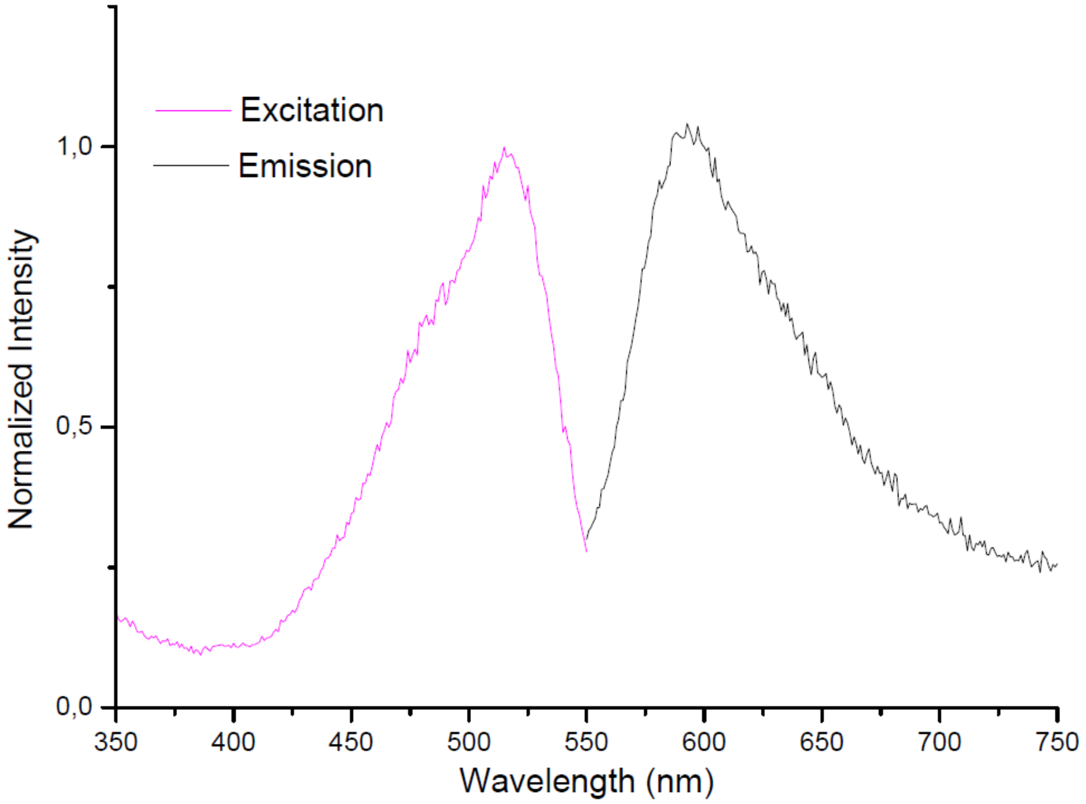

Complex **3d**

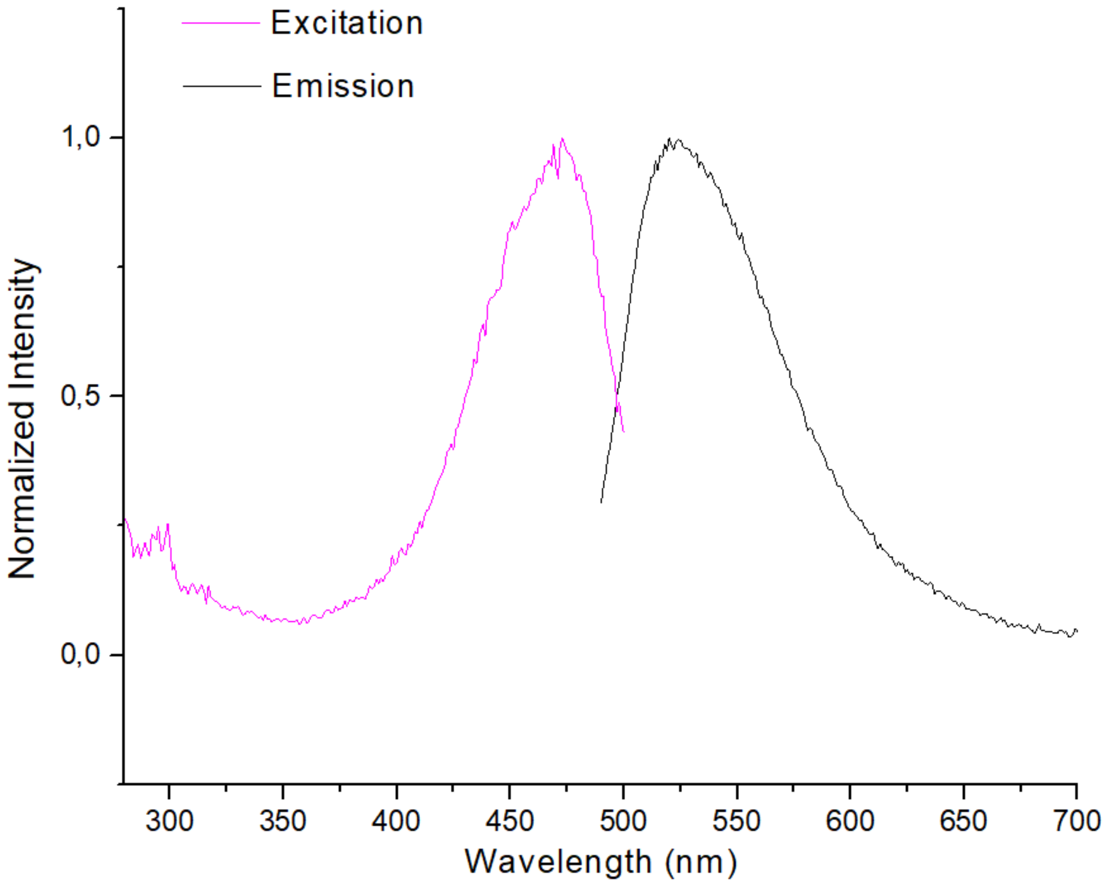

Complex **3e**

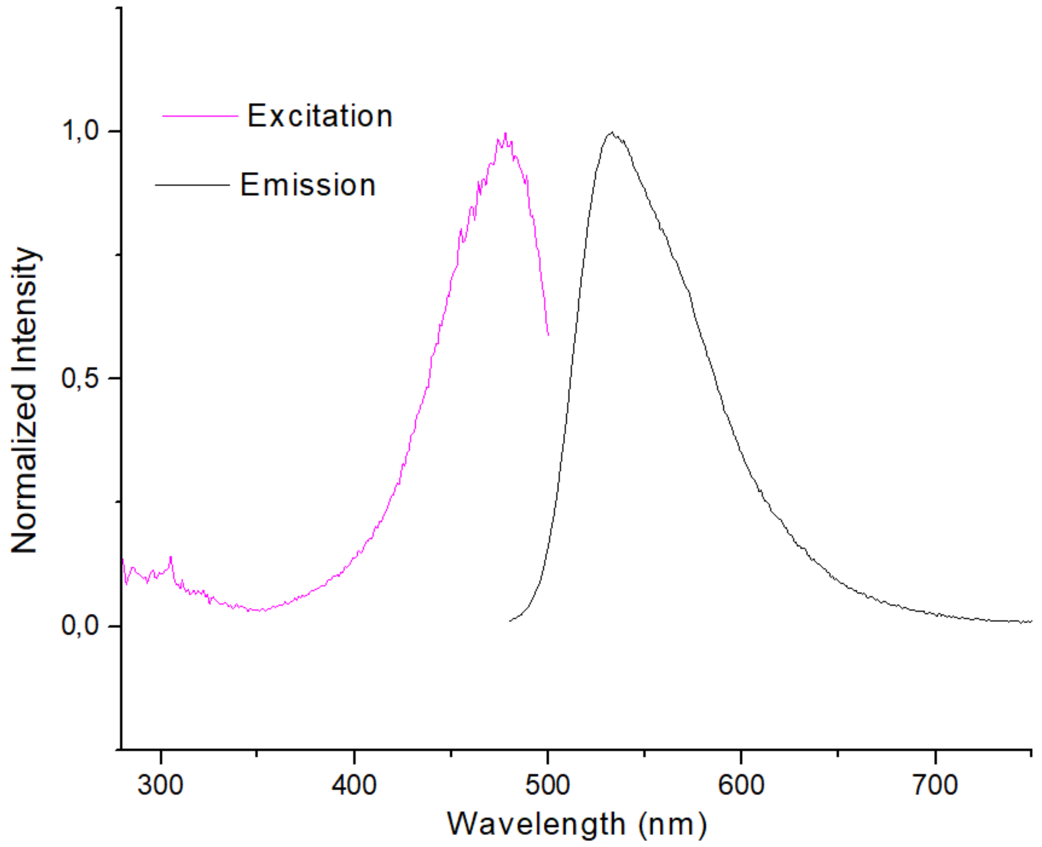

Complex **3f**

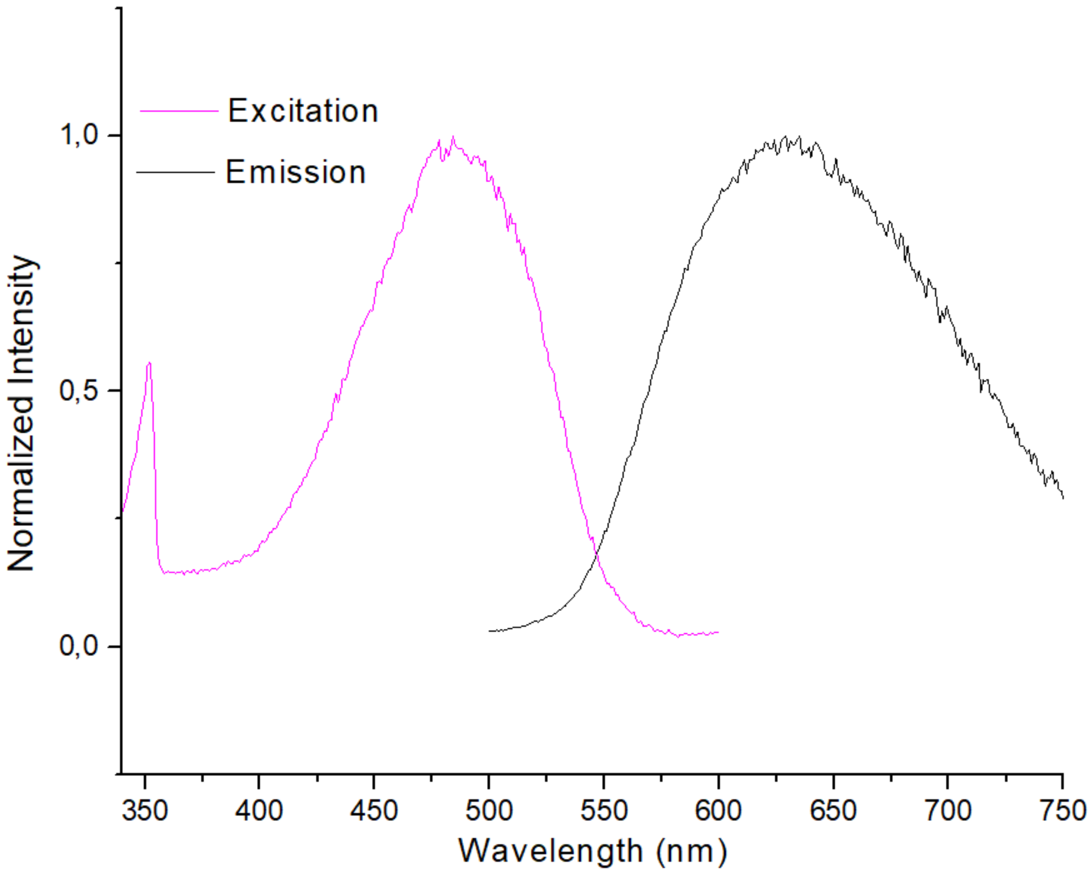

Complex **3g**

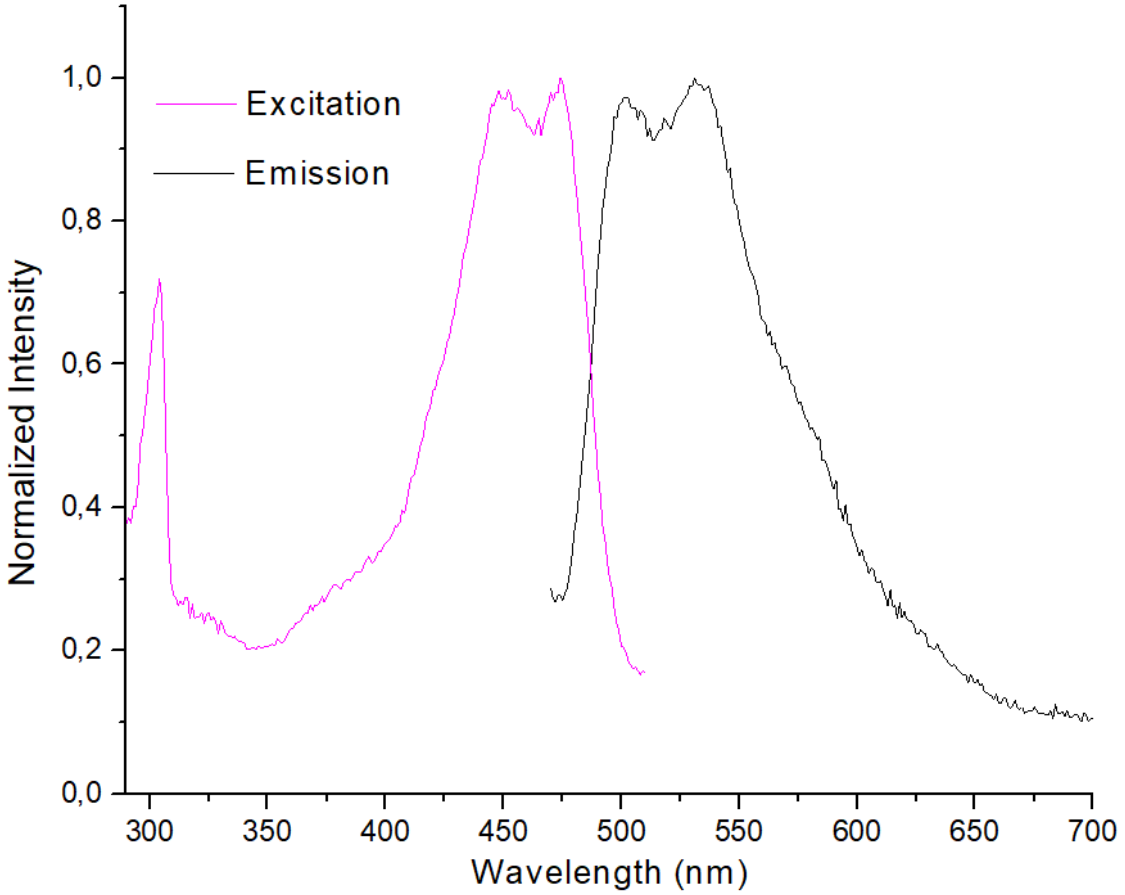

Complex **3h**

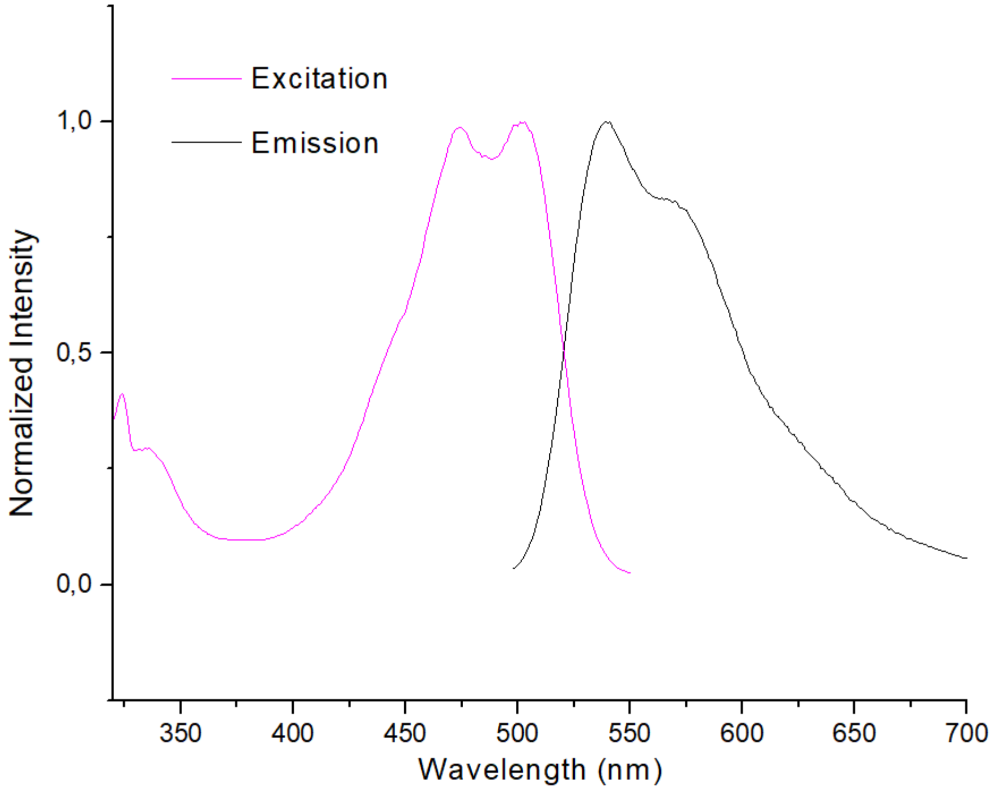

Complex **3i**

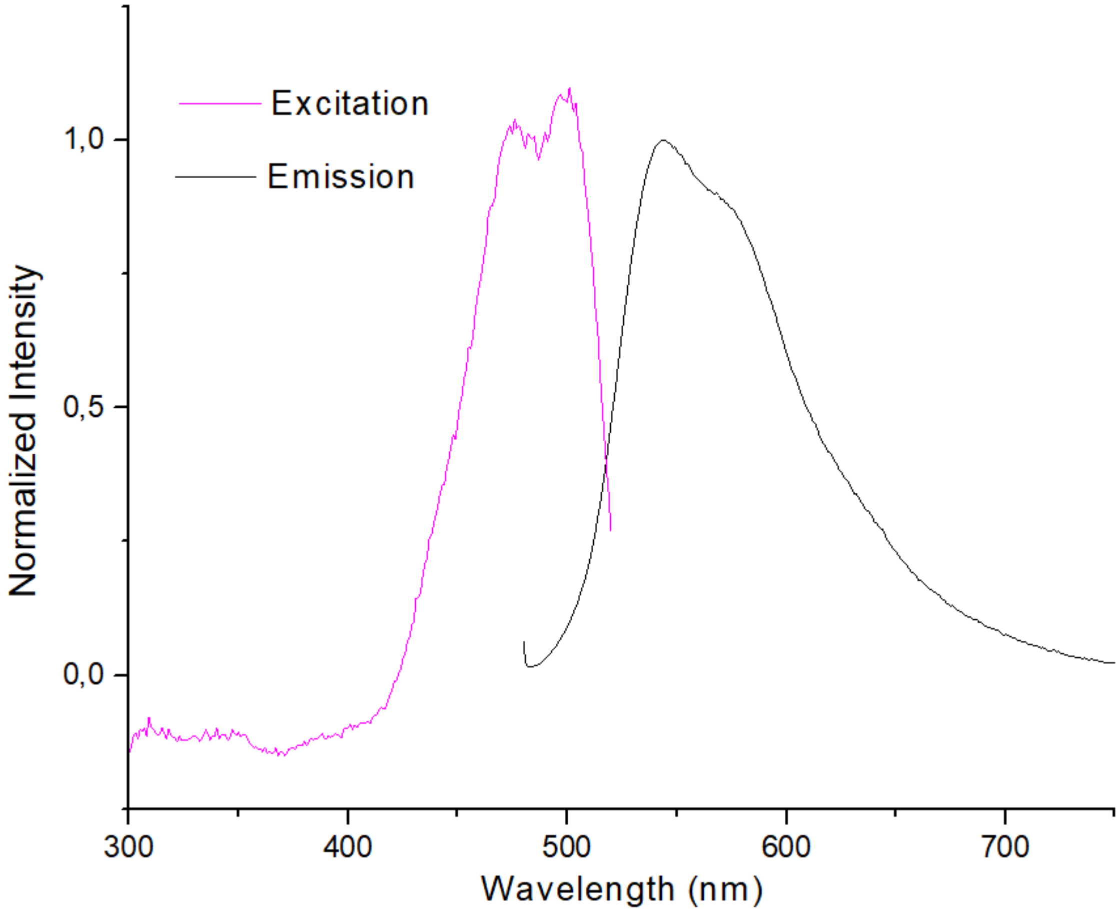

Complex **3j**

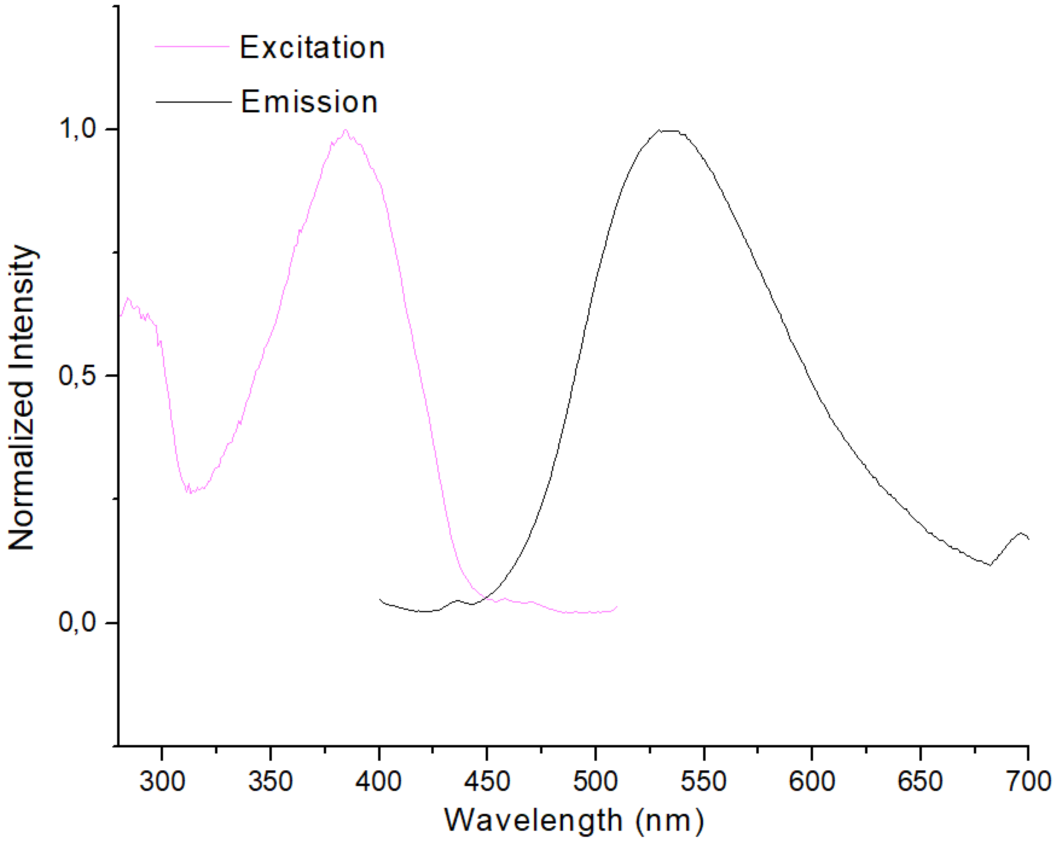

Complex **3k**

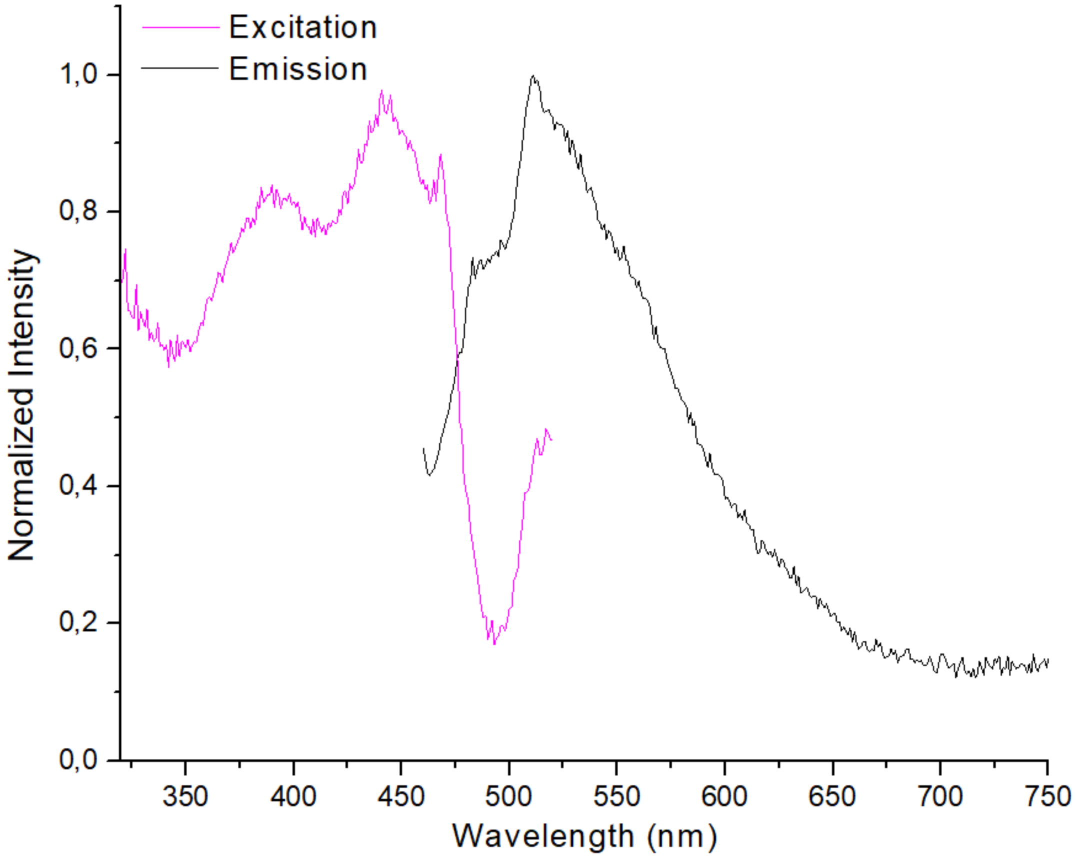

Complex **3m**

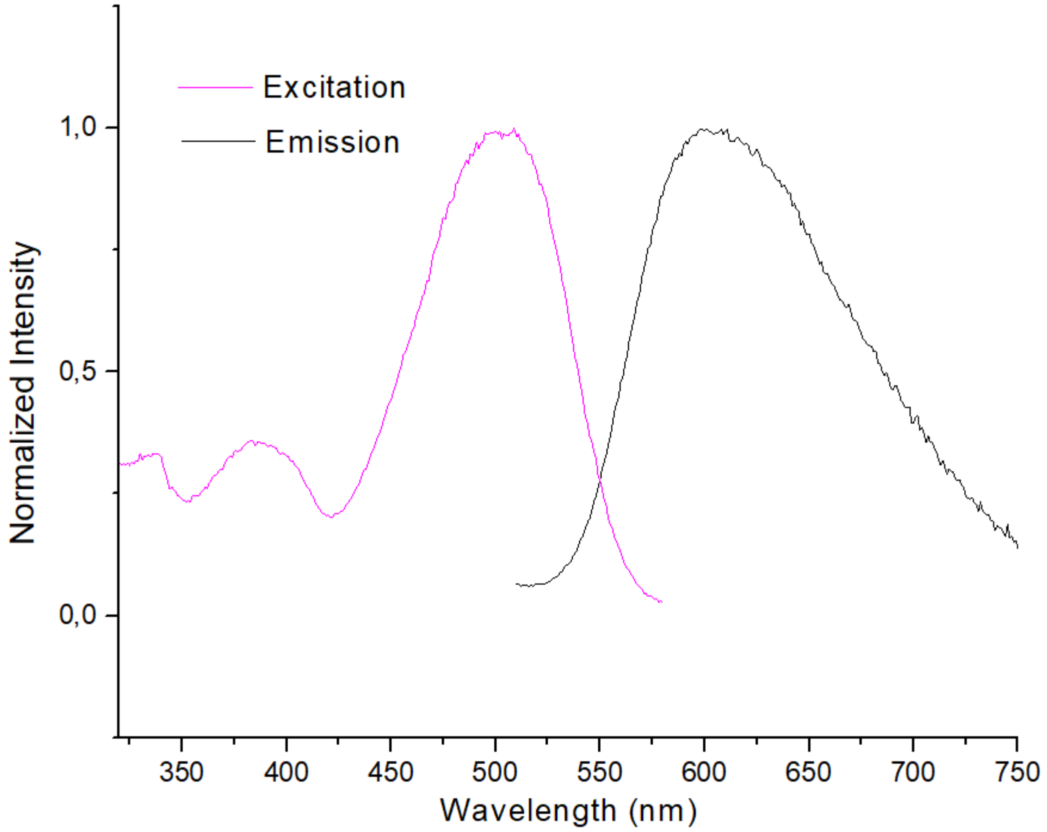

Complex **4d**

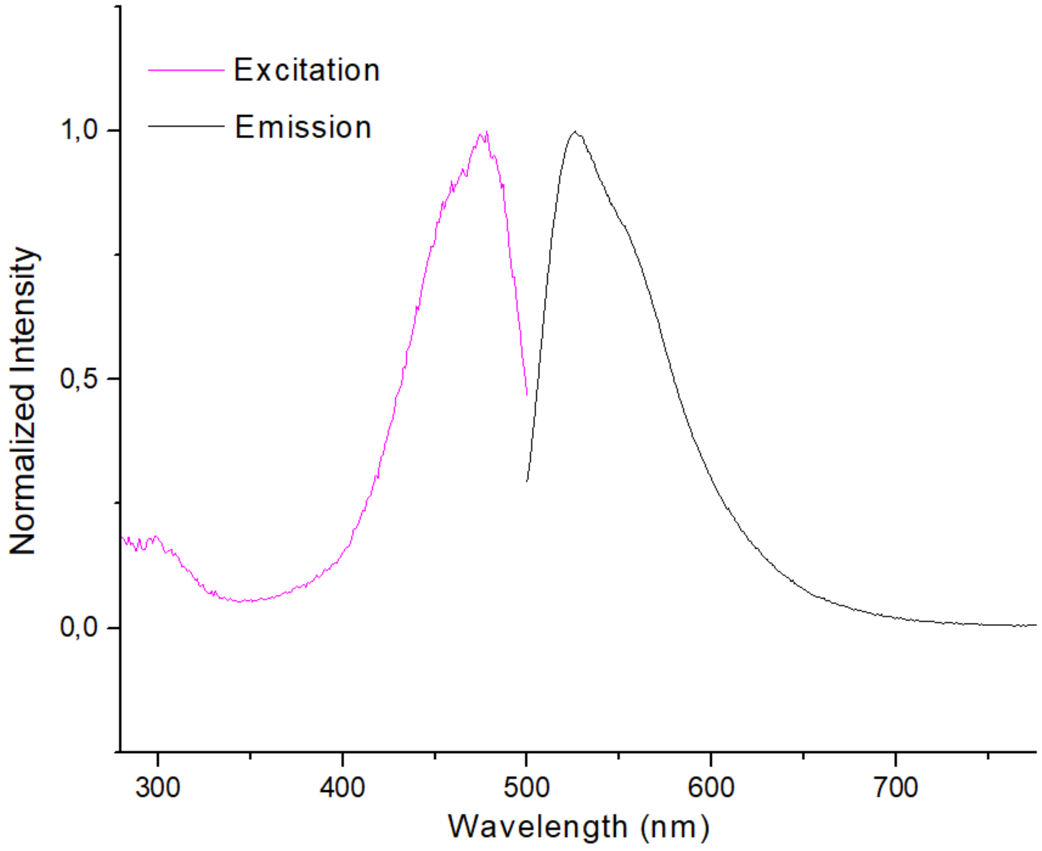

Complex **4h**

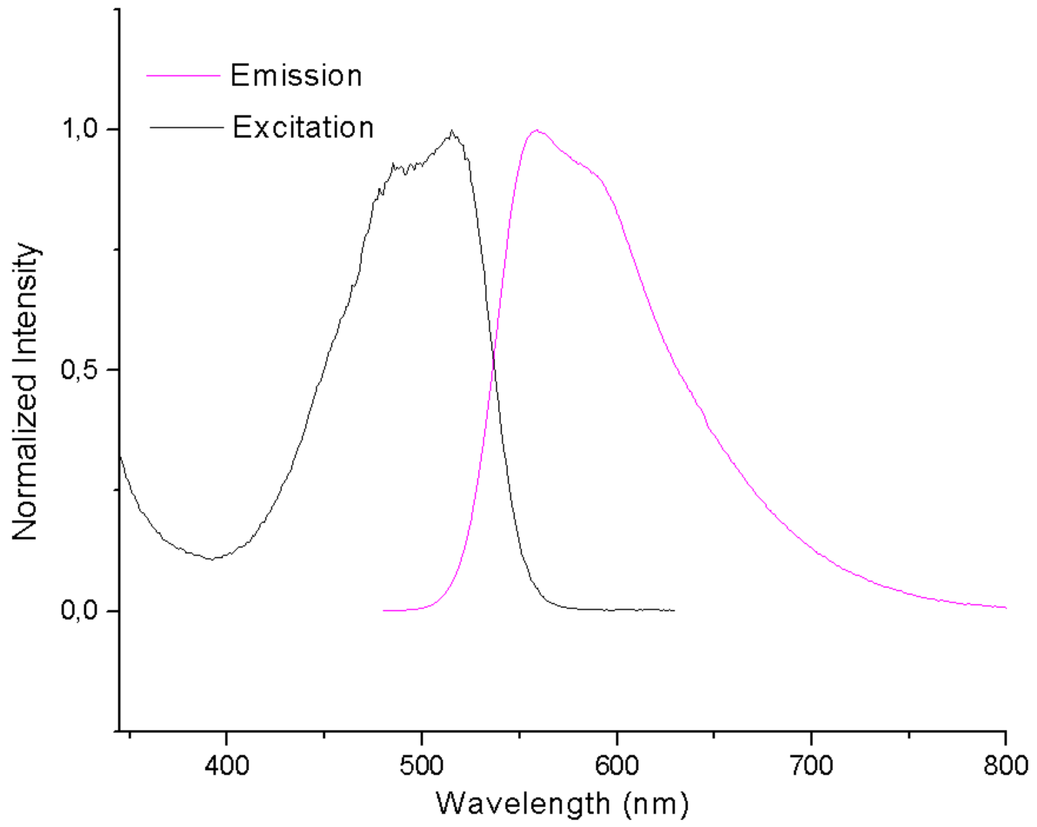

Complex **5h**

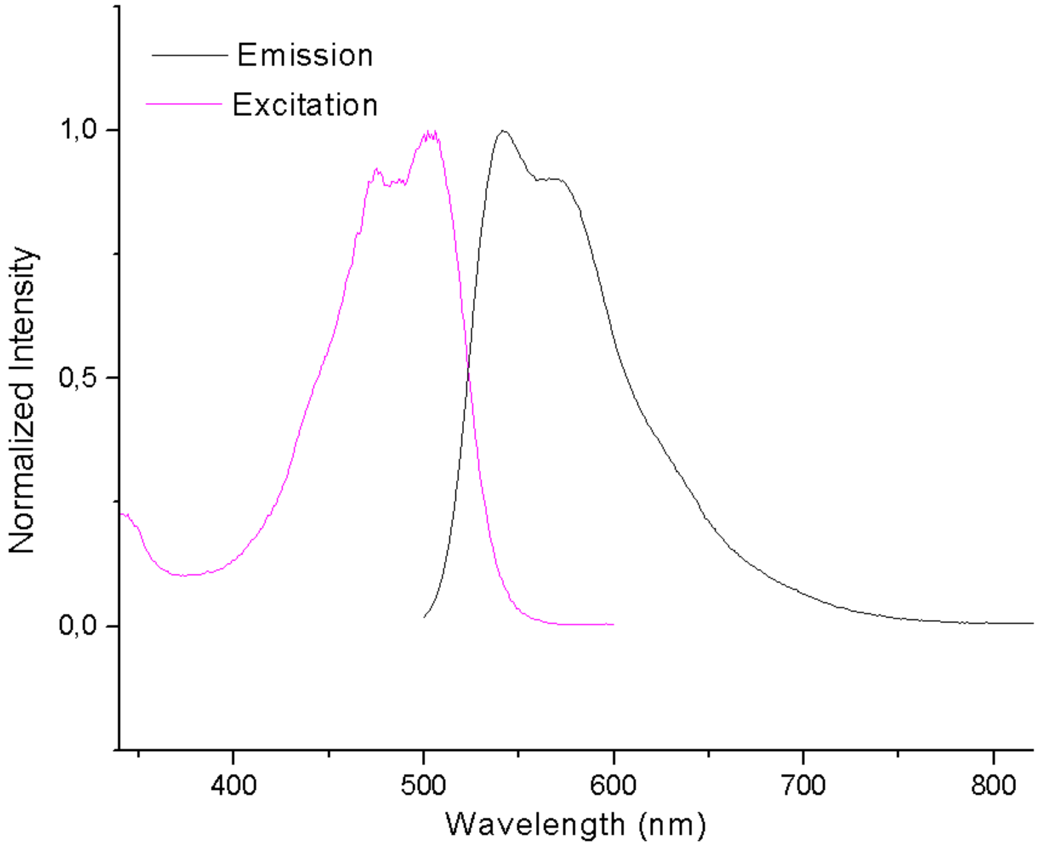

Complex **6h**

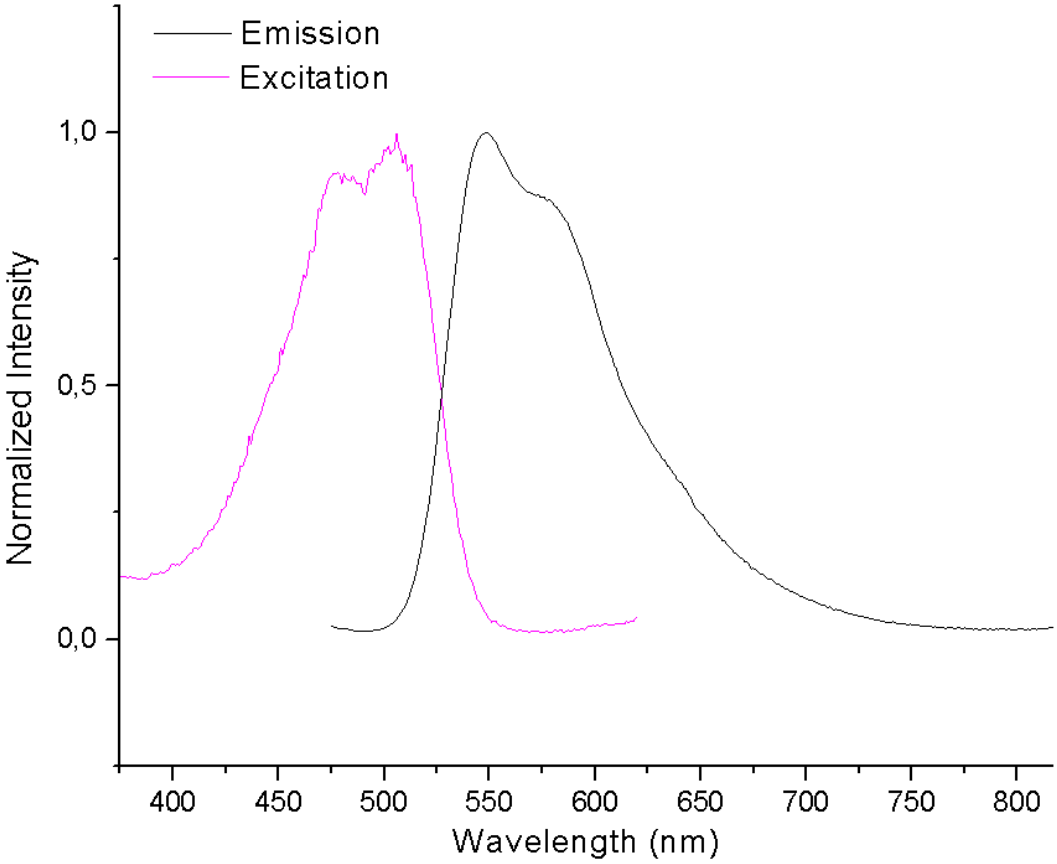

9.- Decay curves and fitting data for the determination of the half-life times of complexes 3

Data Set: 1 / 1

Decay: crv[1]; IRF: crv[0]

Fit

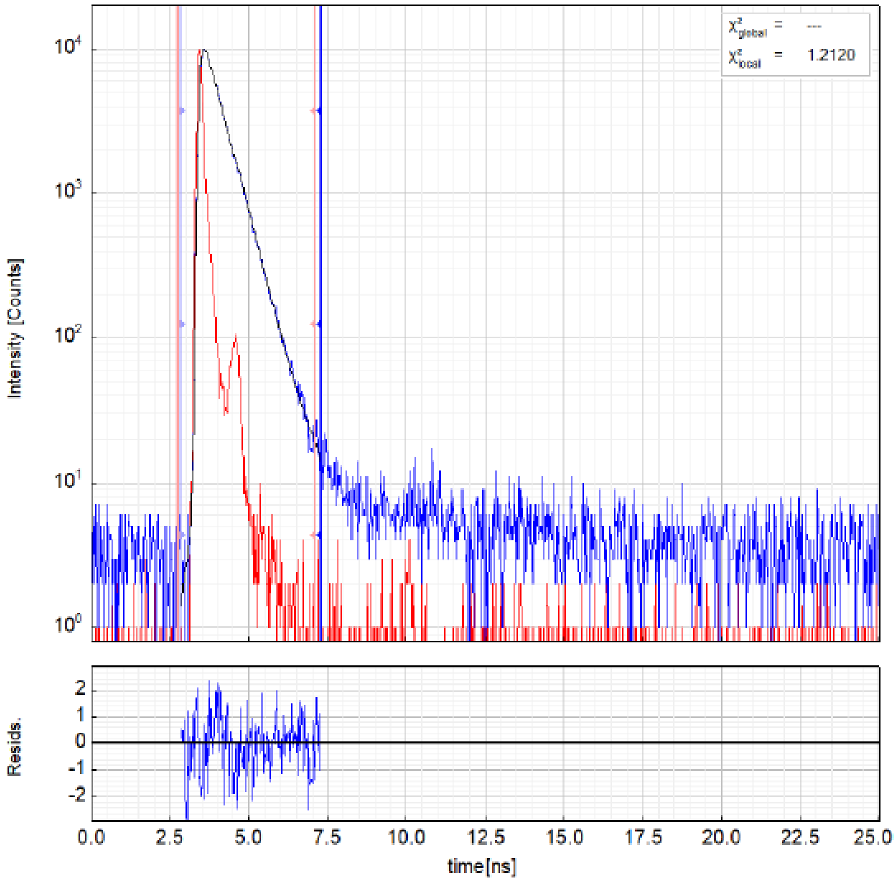

Autocorrelation (Residuals)

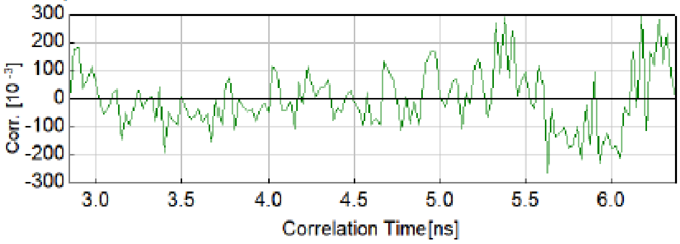

Decay curve and fitting data for complex 3B

Data Set: 1 / 1

Decay: crv[1]; IRF: crv[0]

Fit

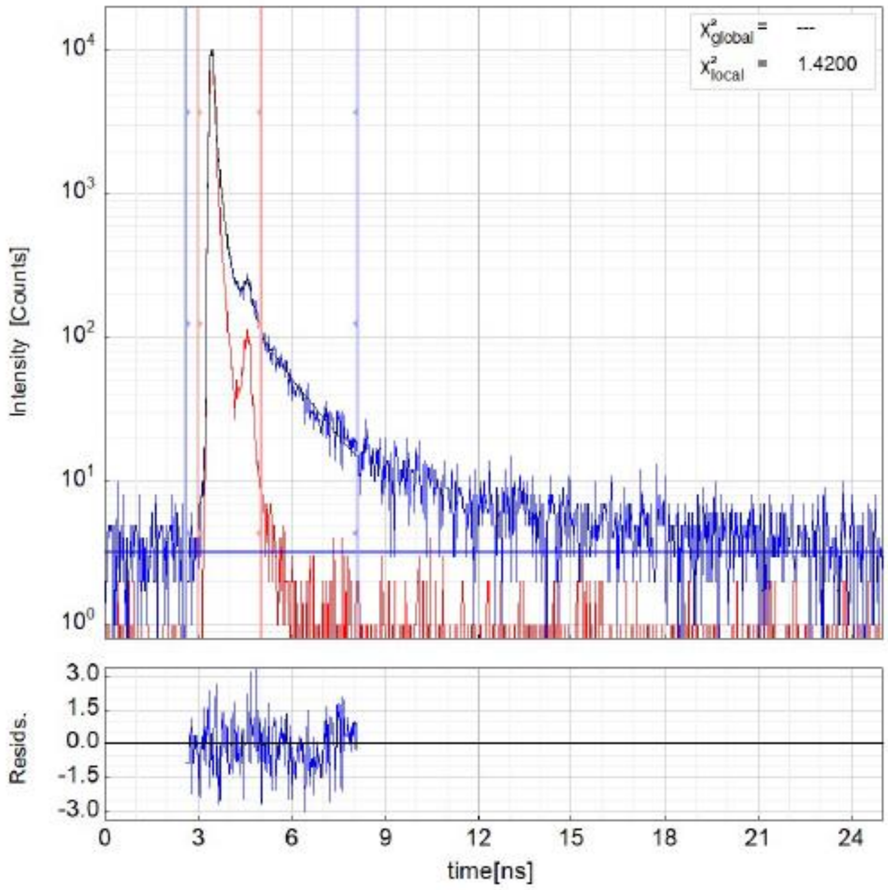

Autocorrelation (Residuals)

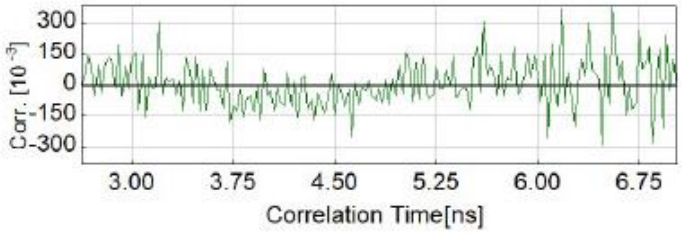

Decay curve and fitting data for complex 3C

Data Set: 1 / 1

Decay: crv[1]; IRF: crv[0]

Fit

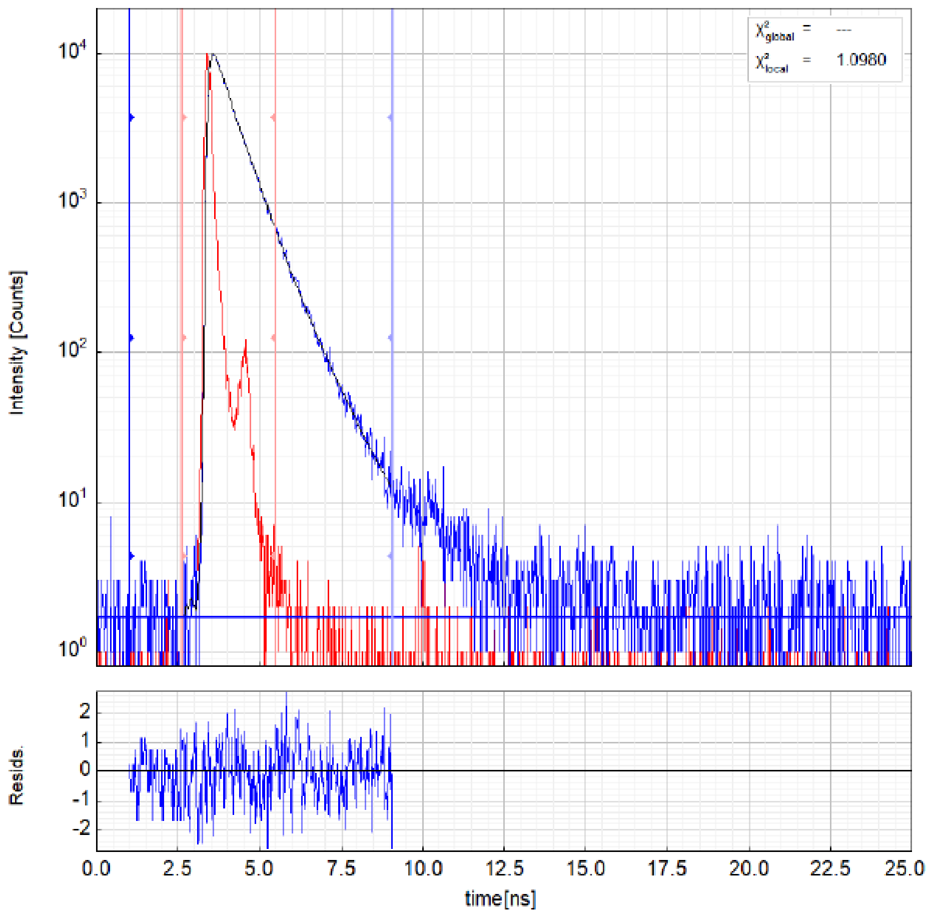

Autocorrelation (Residuals)

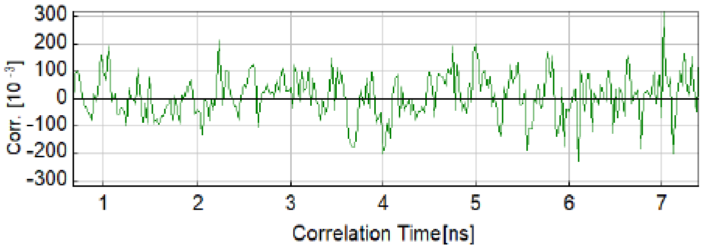

Decay curve and fitting data for complex 3D

Data Set: 1 / 1

Decay: crv[1]; IRF: crv[0]

Fit

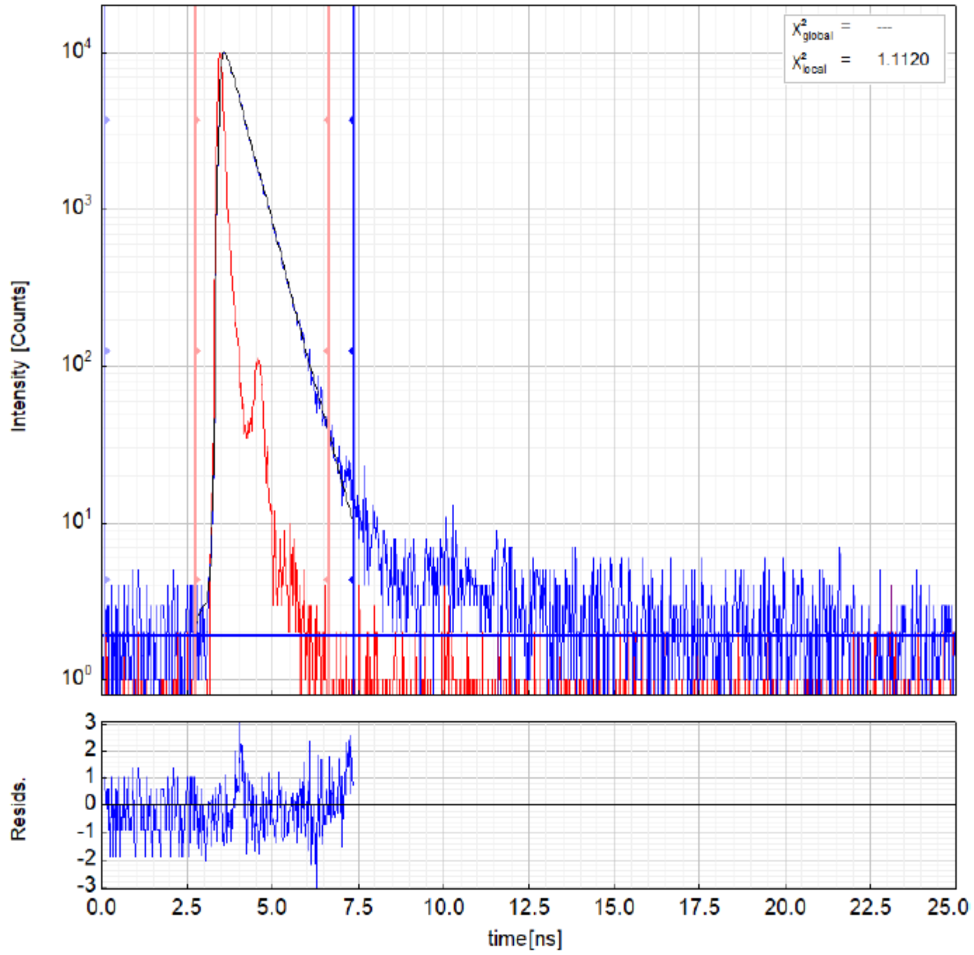

Autocorrelation (Residuals)

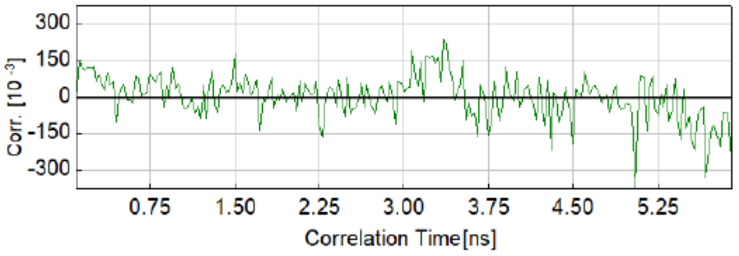

Decay curve and fitting data for complex 3E

Data Set: 1 / 1

Decay: crv[1]; IRF: crv[0]

Fit

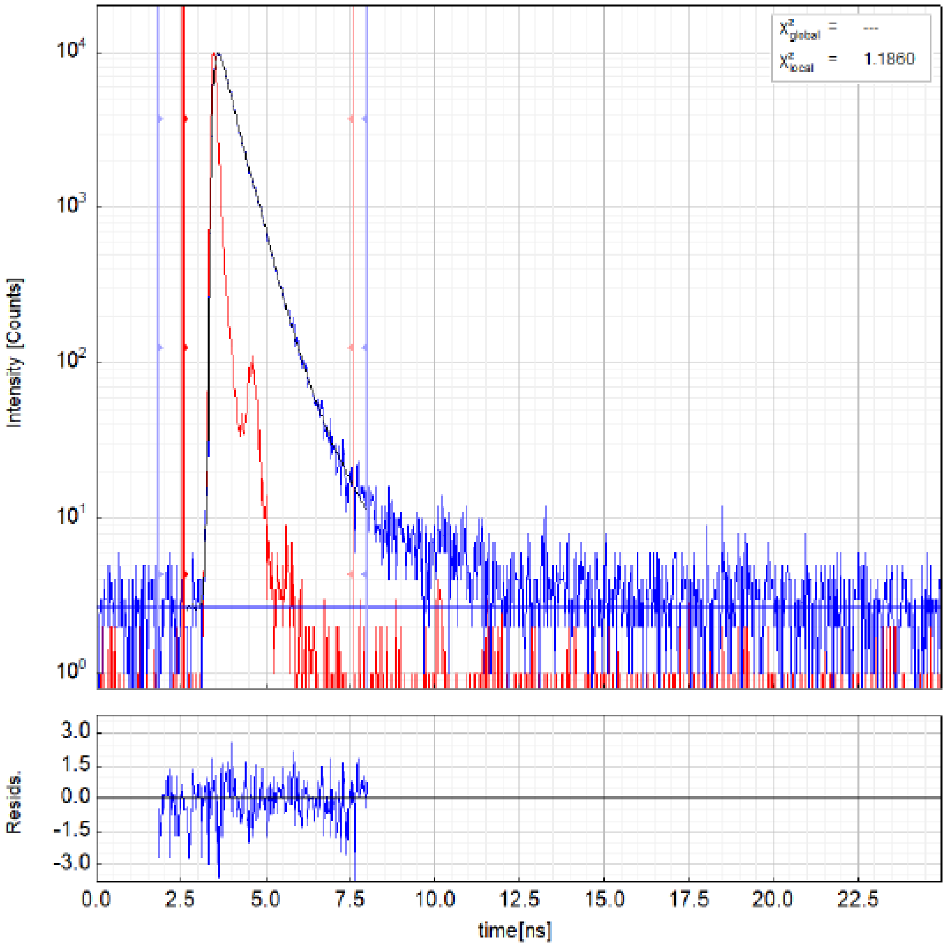

Autocorrelation (Residuals)

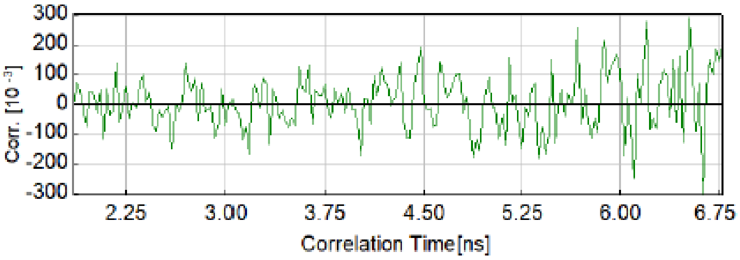

Decay curve and fitting data for complex 3F

Data Set: 1 / 1

Decay: crv[1]; IRF: crv[0]

Fit

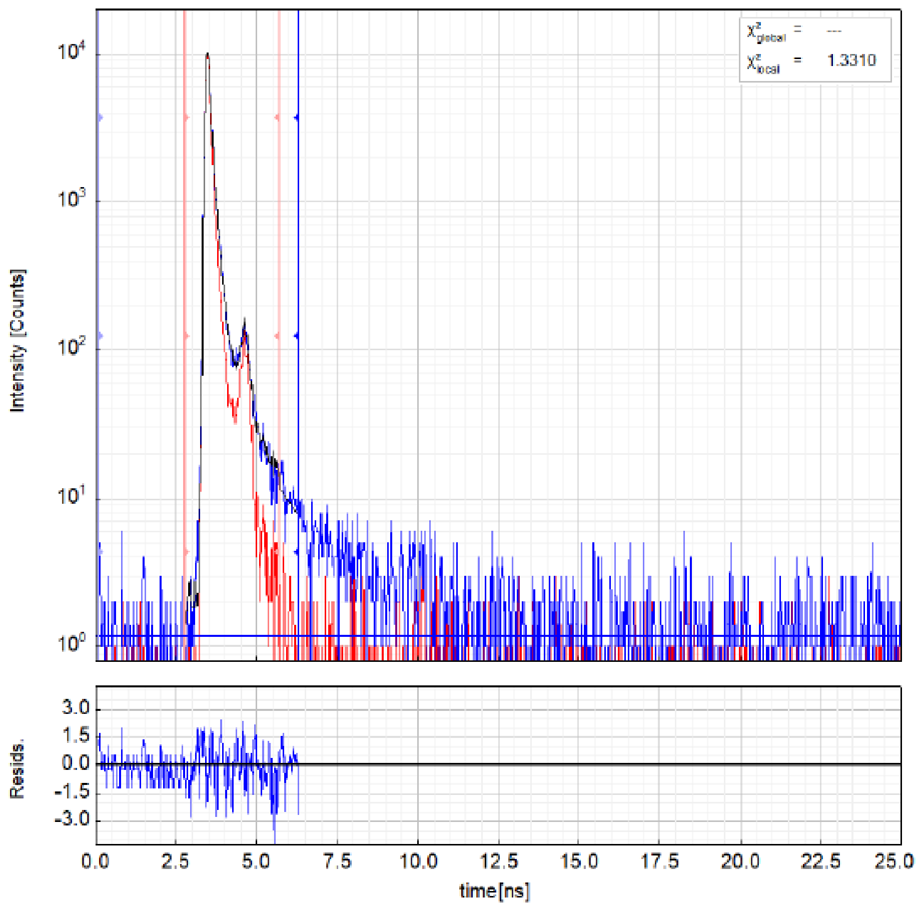

Autocorrelation (Residuals)

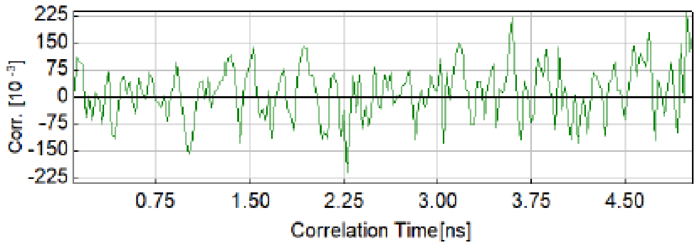

Decay curve and fitting data for complex 3G

Data Set: 1 / 1

Decay: crv[1]; IRF: crv[0]

Fit

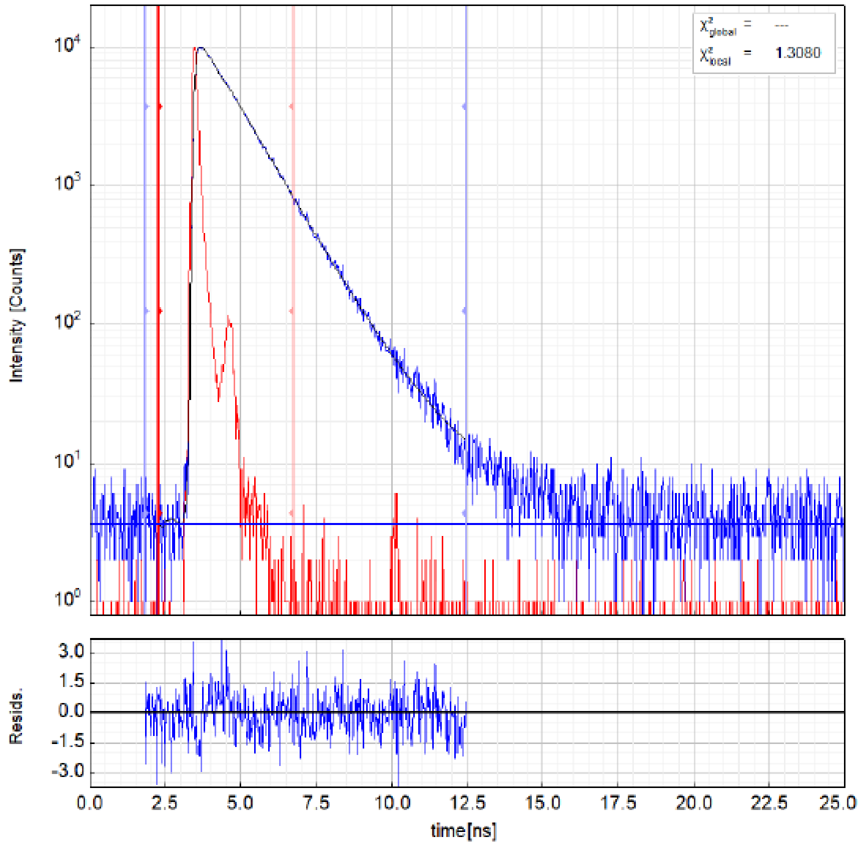

Autocorrelation (Residuals)

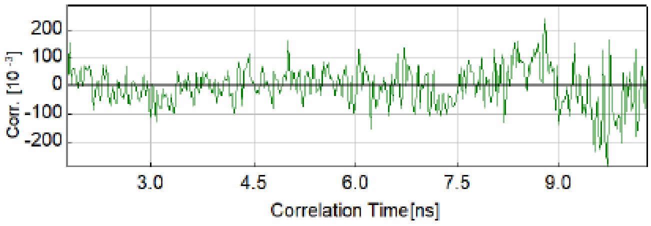

Decay curve and fitting data for complex 3H

Data Set: 1 / 1

Decay: crv[1]; IRF: crv[0]

Fit

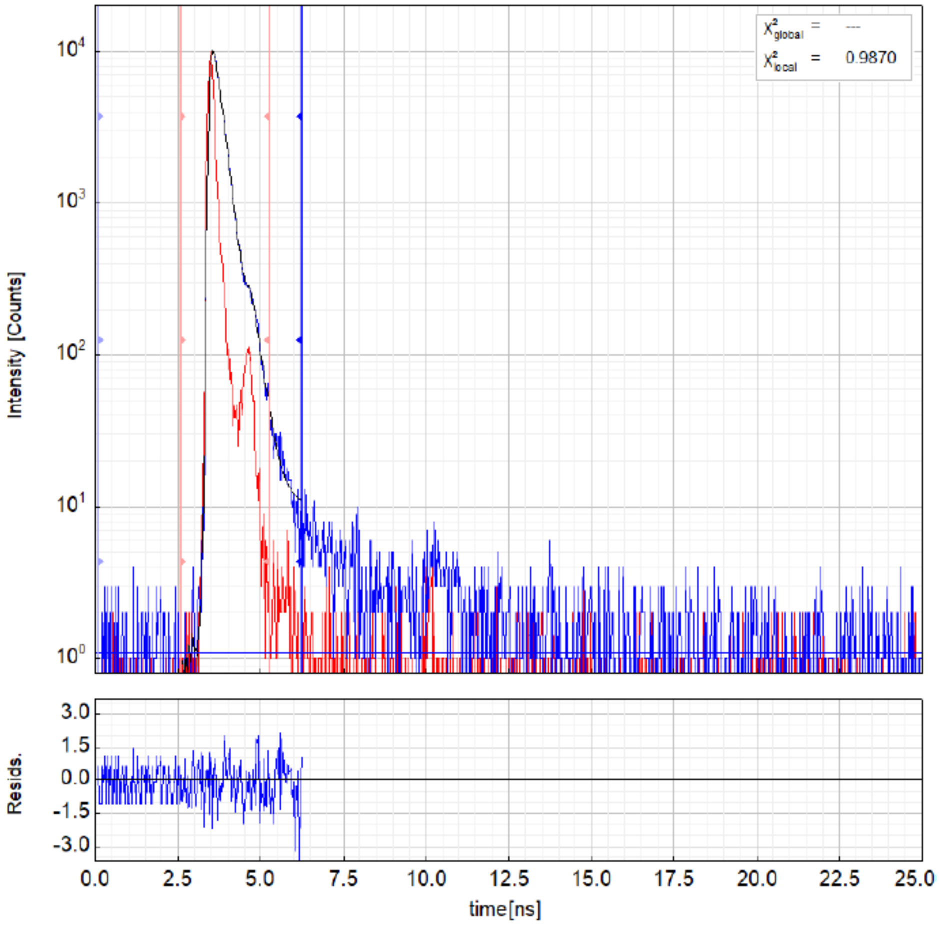

Autocorrelation (Residuals)

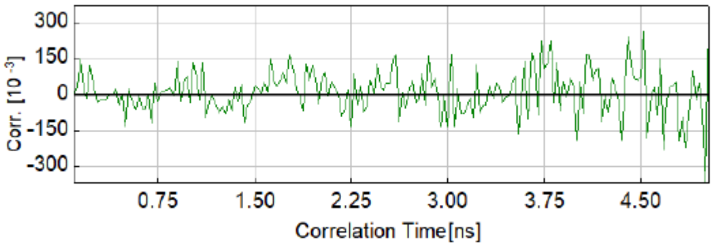

Decay curve and fitting data for complex 3I

Data Set: 1 / 1

Decay: crv[1]; IRF: crv[0]

Fit

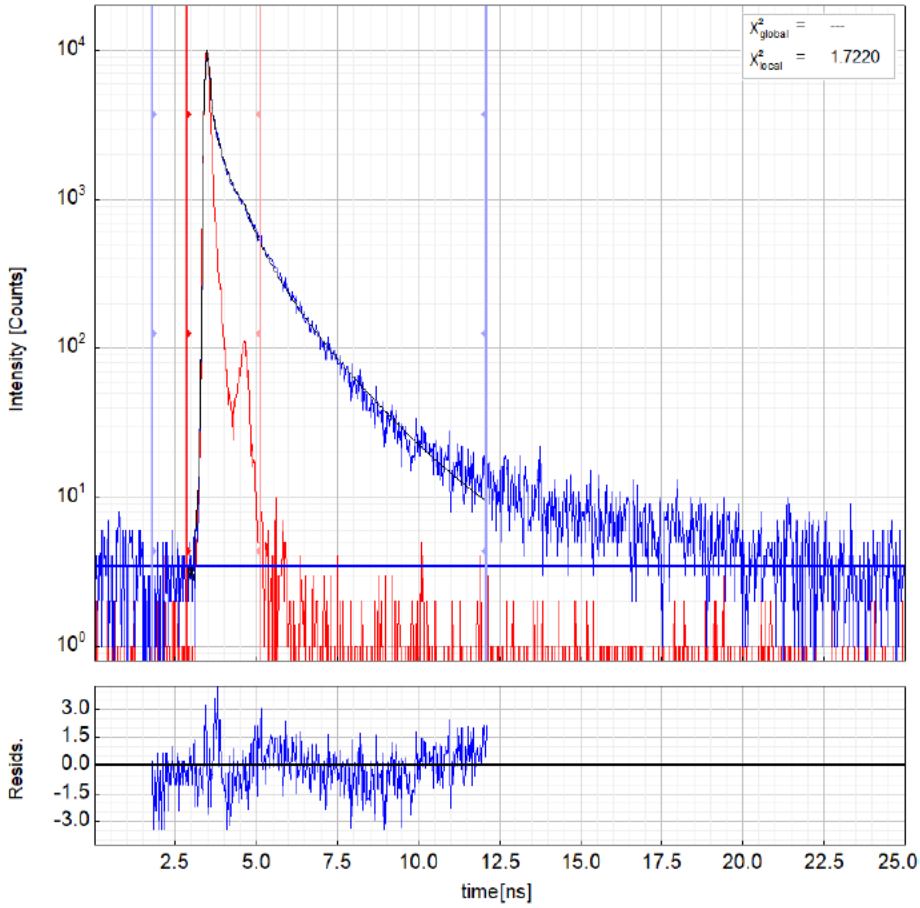

Autocorrelation (Residuals)

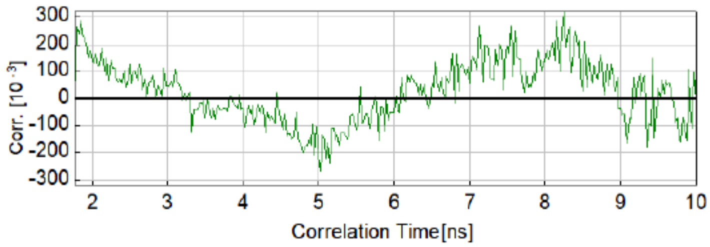

Decay curve and fitting data for complex 3J

Data Set: 1 / 1

Decay: crv[1]; IRF: crv[0]

Fit

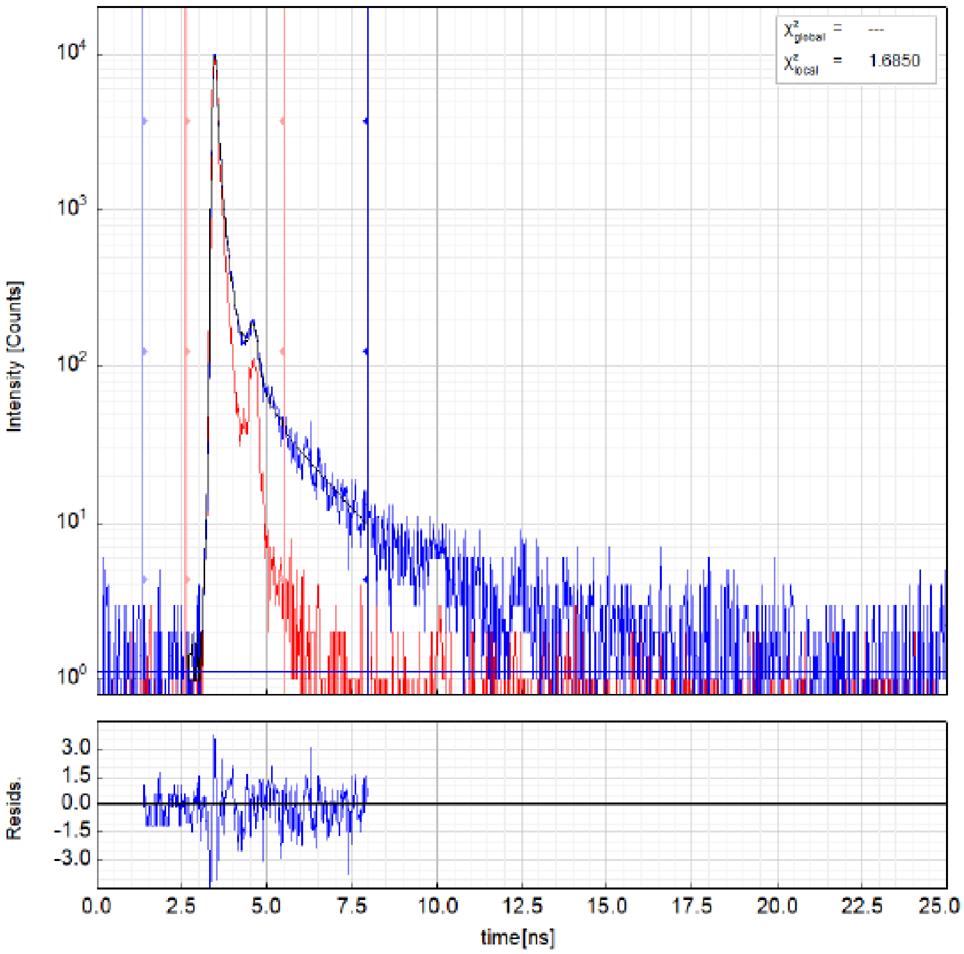

Autocorrelation (Residuals)

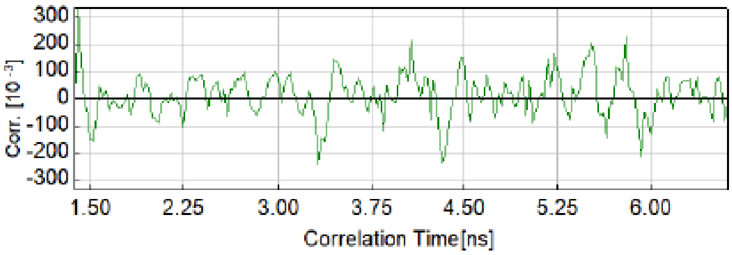

Decay curve and fitting data for complex 3K

Data Set: 1 / 1

Decay: crv[1]; IRF: crv[0]

Fit

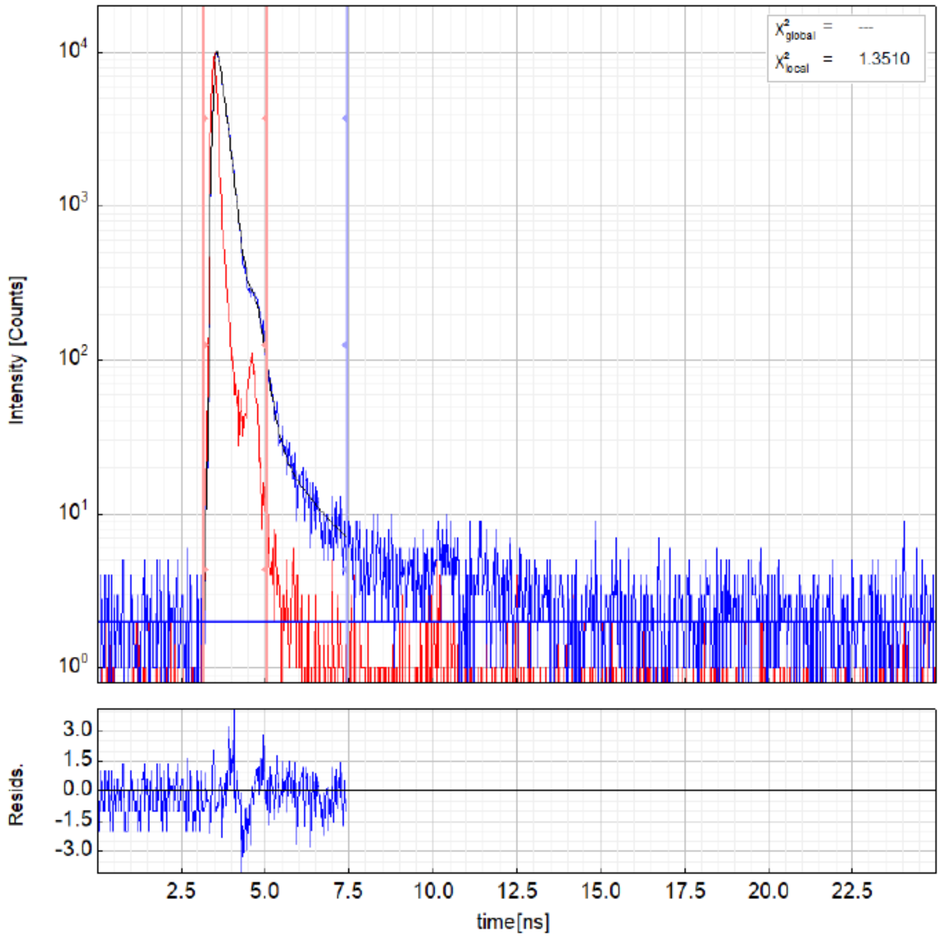

Autocorrelation (Residuals)

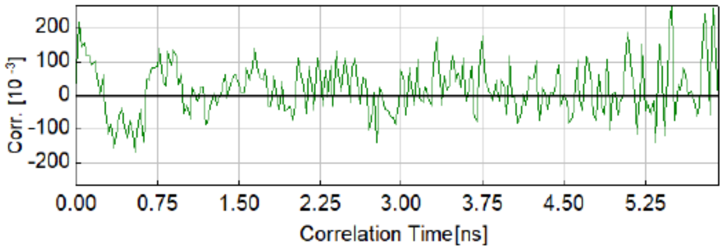

Decay curve and fitting data for complex 3M

Data Set: 1 / 1

Decay: crv[1]; IRF: crv[0]

Fit

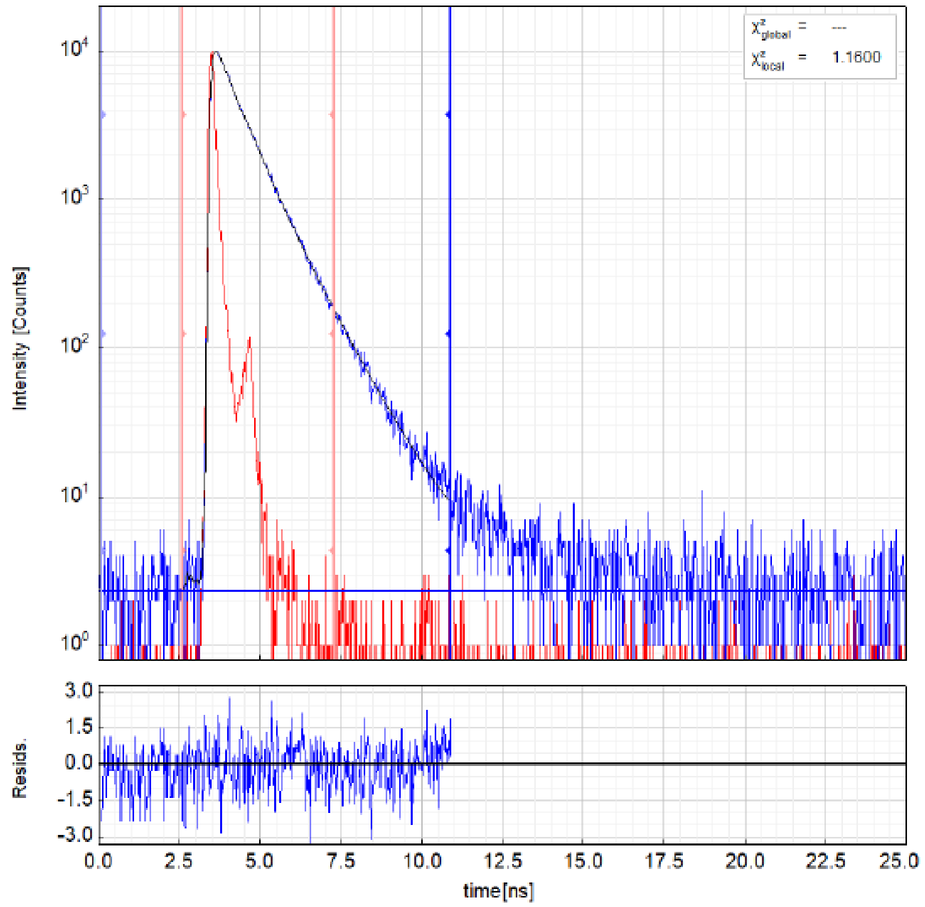

Autocorrelation (Residuals)

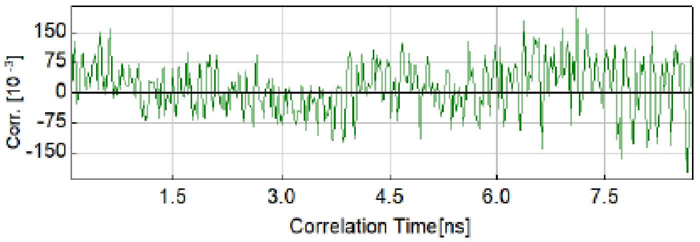

Decay curve and fitting data for complex 4D

Data Set: 1 / 1

Decay: crv[1]; IRF: crv[0]

Fit

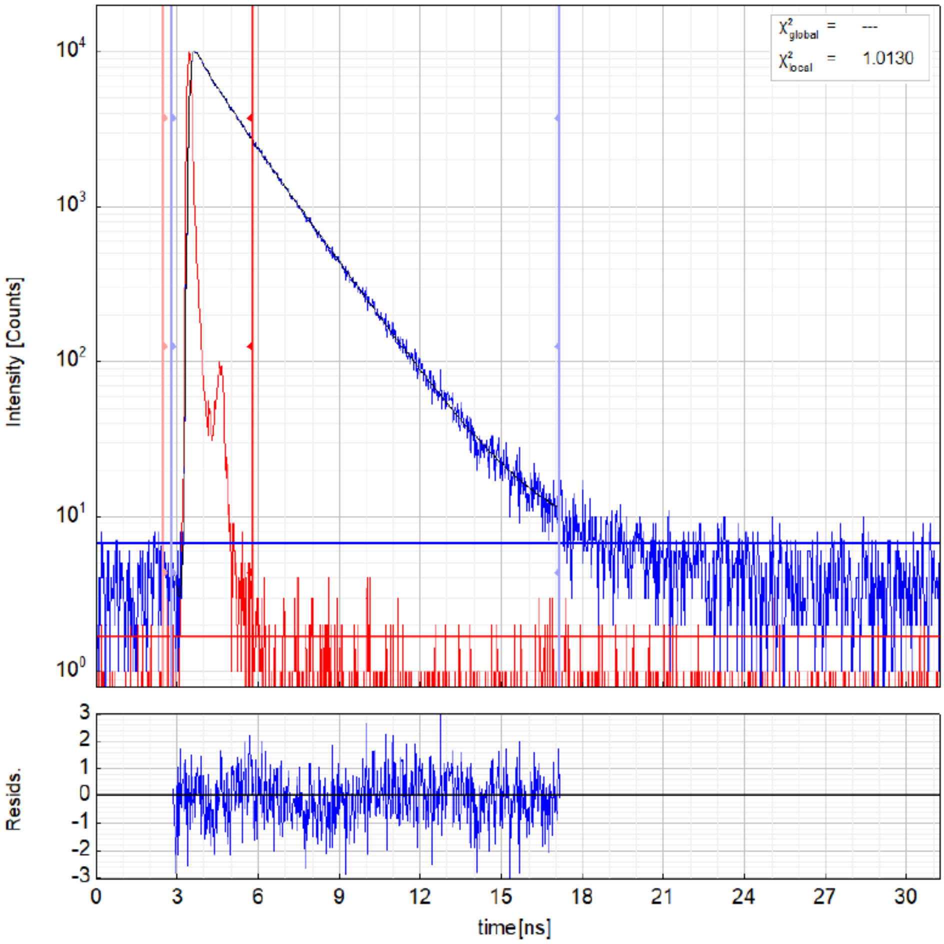

Autocorrelation (Residuals)

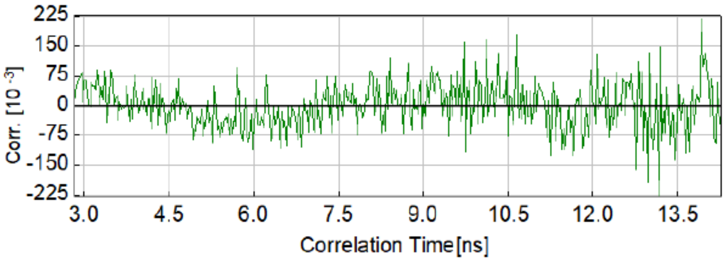

Decay curve and fitting data for complex 4H

Data Set: 1 / 1

Decay: crv[1]; IRF: crv[0]

Fit

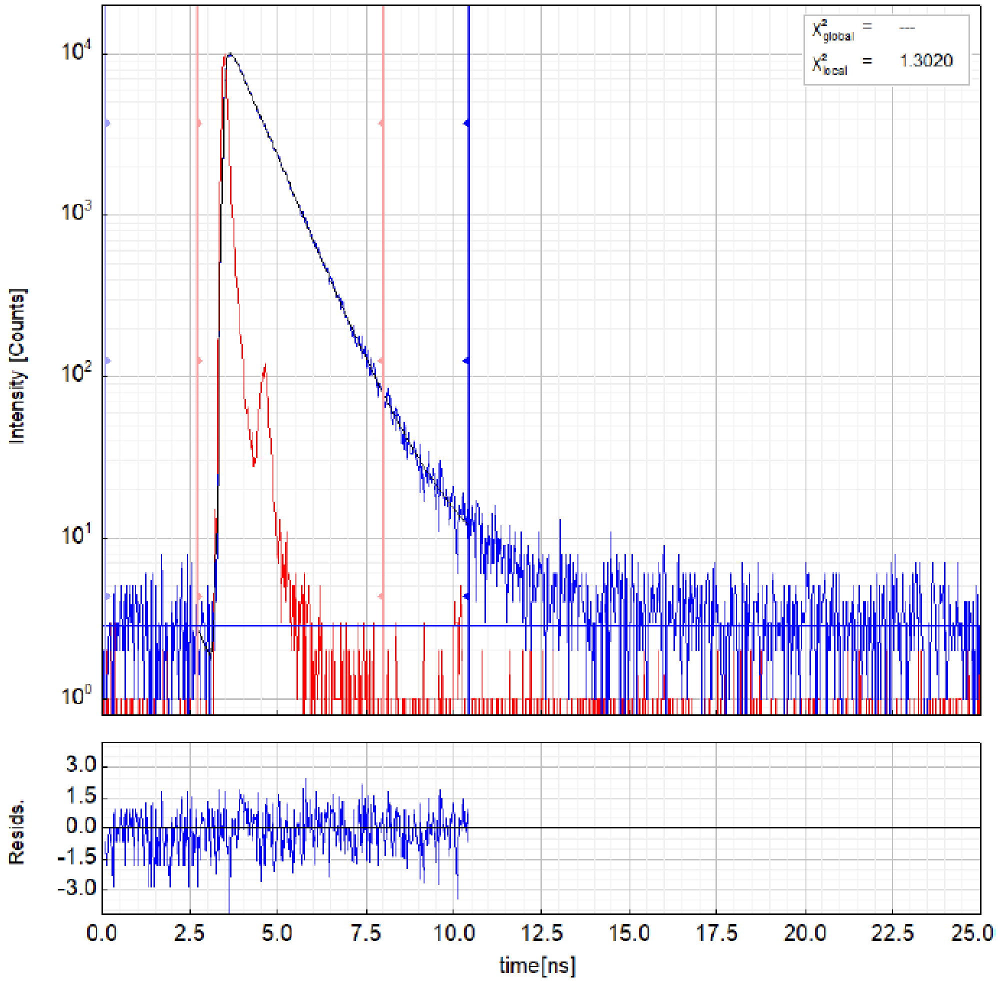

Autocorrelation (Residuals)

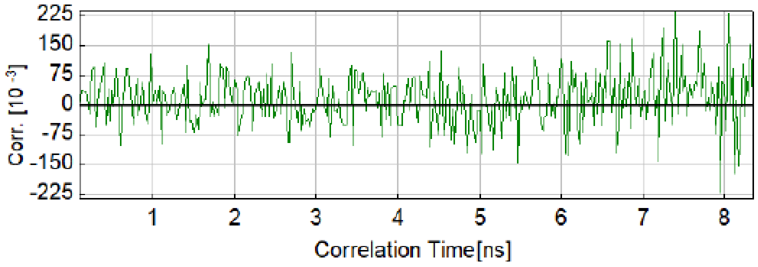

Decay curve and fitting data for complex 5H

Data Set: 1 / 1

Decay: crv[1]; IRF: crv[0]

Fit

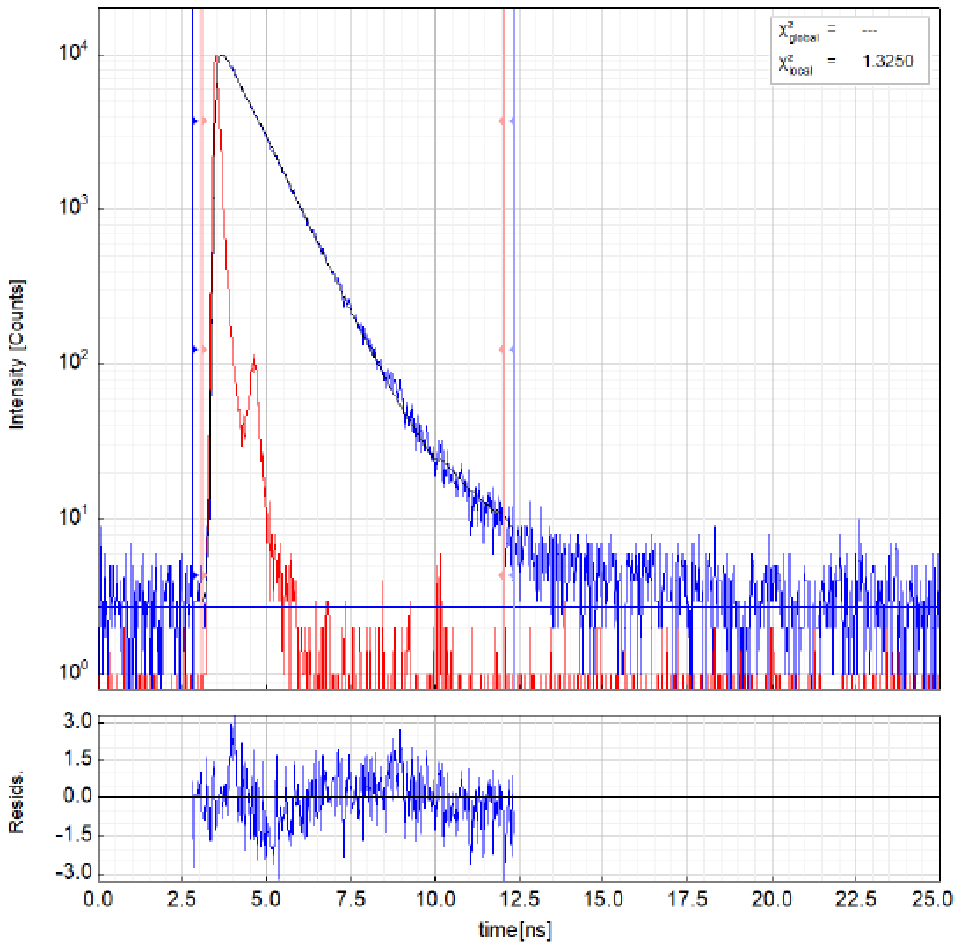

Autocorrelation (Residuals)

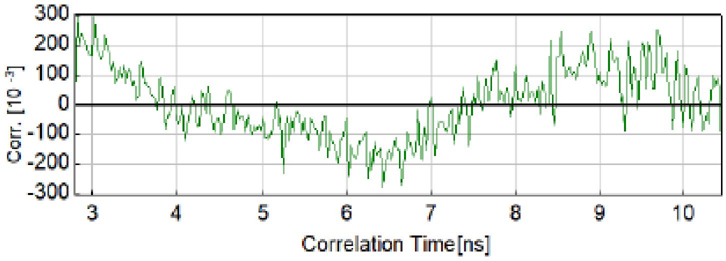

Decay curve and fitting data for complex 6H

Data Set: 1 / 1

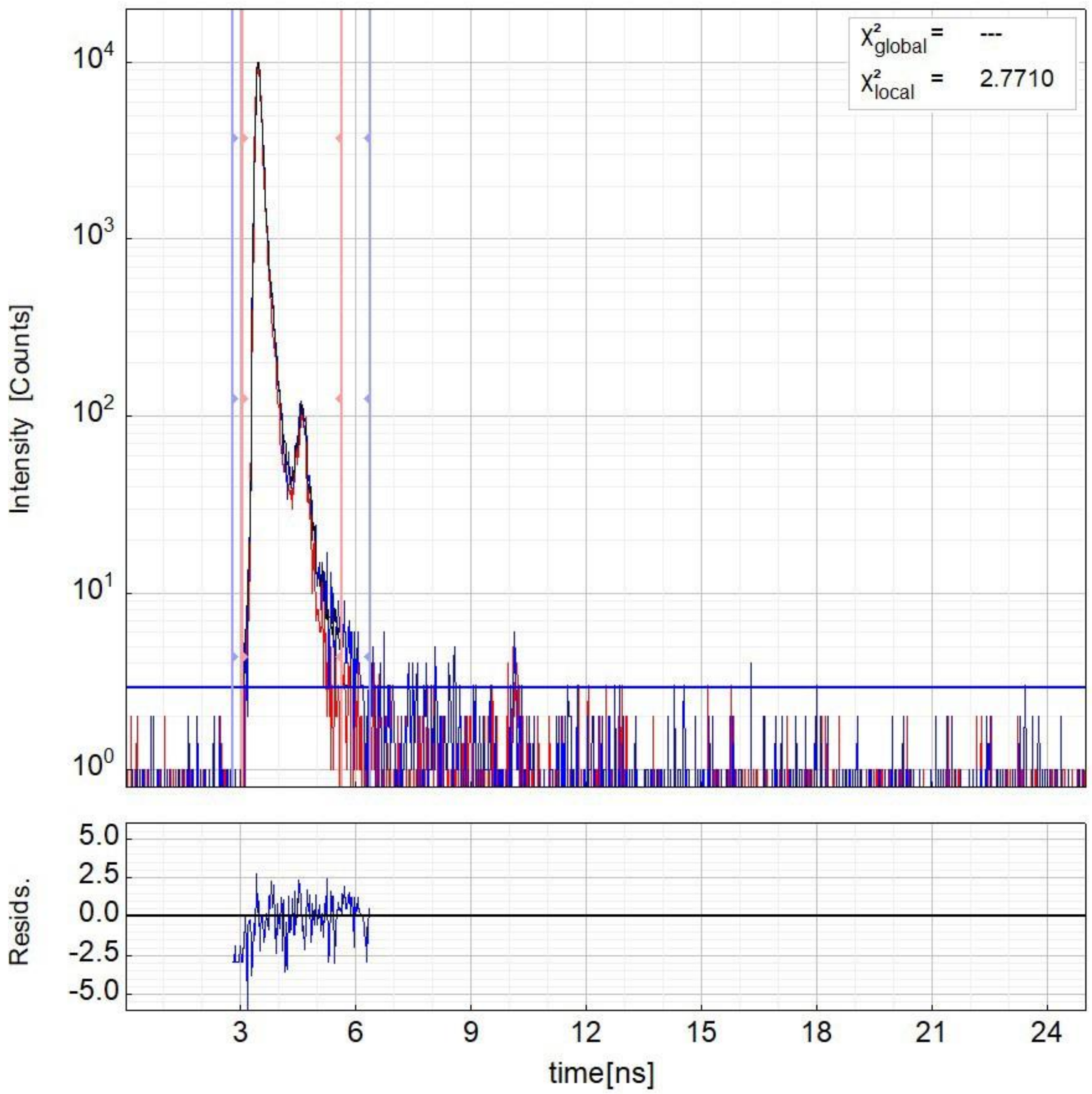

Decay: crv[1]; IRF: crv[0] Fit

Autocorrelation (Residuals)

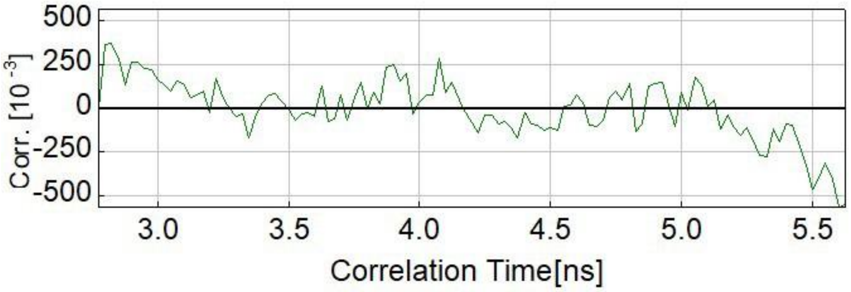

Decay curve and fitting data for oxazolone 1H

10.- Computational Results

In this section the supplementary information regarding the computational results will be provided. In subsection 10.1. the ground state orbital energies and optimized geometries will be given for the selected complexes calculated with both wB97XD and M06-2X functionals. Hence, in subsection 10.2. the calculated vertical excitation energies and oscillator strengths will be given calculated with both functionals. Finally, in subsection 10.3. the emission properties are shown, namely, calculated vertical excitations and oscillator strengths from the optimized geometries of S_1 electronic excited states, along with their optimized geometries.

10.1.- Ground State

In this subsection, the calculated orbital energies (in hartree) for the highest occupied molecular orbitals and lowest unoccupied molecular orbital are given, along with the metal participation in each orbitals. In addition, HOMO – LUMO gap (in eV) and the optimized cartesian coordinates for all selected Pd-Oxazolone complexes will be given (in Å).

10.1.1. wB97XD

Table S2*: calculated orbital energies (hartree) and metal participation, along with the HOMO – LUMO gap (eV) using the wB97XD functional.*

Compound	HOMO-2	HOMO-1	HOMO	Δ_{H-L}	LUMO
**3a**	-0.32242	-0.30765	-0.28816 (12.1%)	6.448	-0.05122 (2.0%)
**3b**	-0.32328	-0.31972	-0.28584 (8.5%)	6.345	-0.05267 (2.0%)
**3c**	-0.32180	-0.29994	-0.27222 (2.3%)	5.829	-0.05802 (2.3%)
**3d**	-0.32287	-0.31218	-0.28325 (6.9%)	6.534	-0.04311 (3.1%)
**3e**	-0.32686	-0.31581	-0.28984 (4.4%)	6.536	-0.04964 (6.1%)
**3f**	-0.32655	-0.31505	-0.28765 (4.3%)	6.169	-0.06096 (1.5%)
**3g**	-0.32093	-0.31932	-0.28785 (10.7%)	6.386	-0.05317 (6.5%)
**3h**	-0.33077	-0.31151	-0.27671 (6.6%)	6.193	-0.04916 (8.8%)
**3i**	-0.32178	-0.29915	-0.28593 (16.9%)	6.246	-0.05639 (1.7%)
**3j**	-0.32004	-0.29714	-0.28875 (19.2%)	6.372	-0.05459 (5.1%)
**3k**	-0.32846	-0.32081	-0.29884 (21.9%)	6.495	-0.06015 (19.0%)
**3m**	-0.32154	-0.30794	-0.27486 (10.6%)	6.080	-0.05143 (4.0%)
**4d**	-0.33932	-0.32132	-0.29381 (3.8%)	6.532	-0.05378 (5.8%)
**4h**	-0.33222	-0.32234	-0.28836 (5.6%)	6.213	-0.06004 (5.5%)
**5h**	-0.29758	-0.28996	-0.25511 (13.6%)	4.765	-0.08000 (18.2%)
**6h**	-0.30986	-0.30510	-0.27289 (6.8%)	6.232	-0.04388 (1.8%)

Cartesian Coordinates

- **Complex: 3a**

59

	X	Y	Z
C	-0.849455	3.326231	0.525943
N	-0.229251	2.456300	-0.289914
C	0.538244	2.930908	-1.287690
C	0.709572	4.289206	-1.505873
C	0.076272	5.192783	-0.657117
C	-0.715091	4.699688	0.374910
Pd	-0.444687	0.424368	-0.055441
C	1.505957	0.285271	0.302205
C	2.155198	1.273164	1.059115
C	3.539570	1.298649	1.223328
C	4.314880	0.321895	0.603528
C	3.700519	-0.692759	-0.136128
C	2.301454	-0.742168	-0.272061
H	3.999997	2.081668	1.819637
C	4.744817	-1.647720	-0.673672
C	1.730231	-1.909995	-0.911988
C	0.445251	-2.304273	-0.806198
N	-0.575241	-1.629448	-0.114453
C	-1.579878	-2.450449	-0.039376
O	-1.374477	-3.627457	-0.682929
C	-0.093088	-3.591301	-1.234289
O	0.346894	-4.506999	-1.876107
C	-2.840894	-2.303849	0.672142
C	-3.965078	-3.023265	0.245162
C	-5.164292	-2.894122	0.934277
C	-5.240316	-2.066474	2.055500
C	-4.115380	-1.367387	2.491229
C	-2.913793	-1.483206	1.803149
O	-2.505833	0.572254	-0.638822
C	-3.419442	1.106914	0.053635
C	-4.794477	1.107182	-0.677936
F	-5.822976	1.285217	0.164384
O	-3.370903	1.631488	1.165033
F	-4.838232	2.116151	-1.579021
F	-5.023863	-0.033571	-1.350476
H	1.571700	2.058146	1.529171
C	5.769569	0.112699	0.580101
H	-3.900967	-3.662740	-0.628361
H	-6.040752	-3.436502	0.596200
H	-6.178887	-1.967424	2.591312
H	-4.174382	-0.724932	3.362673
H	-2.036005	-0.942766	2.140758
H	1.027172	2.193302	-1.912361
H	1.334351	4.623235	-2.325532
H	0.197269	6.261366	-0.798881
H	-1.231101	5.362286	1.059453
H	-1.478319	2.893411	1.294692
C	6.035511	-1.041595	-0.173918
C	6.807366	0.844568	1.153343
C	8.118548	0.407671	0.960969

C	7.341612	-1.473755	-0.364494
C	8.384305	-0.740553	0.208844
H	7.553159	-2.365839	-0.947331
H	9.410742	-1.065726	0.069392
H	8.940953	0.964603	1.399474
H	6.605750	1.737512	1.738069
H	4.606300	-2.665338	-0.289744
H	4.721753	-1.711308	-1.768158
H	2.392371	-2.590074	-1.440829

- **Complex: 3b**

54

	X	Y	Z
C	-2.525133	-1.424817	1.711383
C	-2.413311	-2.255645	0.590886
C	-3.528596	-2.959970	0.117746
C	-4.755872	-2.807215	0.750394
C	-4.870134	-1.969345	1.860579
C	-3.755252	-1.284091	2.341969
C	-1.124116	-2.429479	-0.061047
N	-0.094949	-1.635342	-0.078733
C	0.937744	-2.324681	-0.733934
C	0.389767	-3.590936	-1.201779
O	-0.917480	-3.602370	-0.708957
C	2.239760	-1.966994	-0.784528
C	2.832893	-0.832111	-0.112003
C	2.031259	0.177063	0.437868
C	2.644434	1.145402	1.278086
C	3.994224	1.145493	1.511394
C	4.845853	0.190009	0.897431
C	4.272592	-0.816487	0.071389
Pd	0.108993	0.402176	-0.004567
O	-1.917481	0.646536	-0.659604
C	-2.831549	1.201596	0.016616
O	-2.791898	1.718454	1.131982
H	4.434011	1.895481	2.164044
C	5.157858	-1.756548	-0.523756
O	0.834280	-4.511052	-1.835739
N	0.435138	2.424570	-0.242875
C	-0.203848	3.335367	0.511896
C	0.001259	4.698735	0.349464
C	0.886124	5.138308	-0.629490
C	1.539164	4.192844	-1.415310
C	1.292854	2.847457	-1.189372
C	-4.189938	1.240645	-0.744140
F	-4.438166	0.103434	-1.415768
F	-5.230000	1.453555	0.075326
F	-4.183953	2.245683	-1.650247
H	2.034418	1.909684	1.747778
H	-3.434734	-3.607205	-0.747310
H	-5.624311	-3.338897	0.376507
H	-5.830502	-1.851314	2.352114
H	-3.843519	-0.633259	3.204676
H	-1.655605	-0.894785	2.085325
H	1.792383	2.078241	-1.766090

H	2.235666	4.484650	-2.192160
H	1.063686	6.197889	-0.778880
H	-0.533525	5.395432	0.983923
H	-0.904177	2.943481	1.240303
H	2.874786	-2.677471	-1.301384
C	6.516188	-1.691429	-0.314324
C	6.249376	0.229733	1.105630
C	7.075237	-0.691664	0.511769
H	4.782497	-2.542424	-1.167852
H	7.164781	-2.419802	-0.791014
H	8.147850	-0.655881	0.672140
H	6.657993	1.007472	1.745027

- **Complex 3c**

62

	X	Y	Z
C	6.197564	-1.279842	-0.282537
C	5.448713	-2.264039	-1.011655
C	4.093368	-2.225286	-1.075896
C	3.330296	-1.186776	-0.427426
C	4.054310	-0.193900	0.285647
C	5.481833	-0.241566	0.365831
C	1.902460	-1.116719	-0.461364
C	1.219787	-0.011955	0.115275
C	1.961790	0.929482	0.823385
C	3.354555	0.855344	0.938453
C	1.167853	-2.245172	-0.984992
C	-0.144267	-2.500299	-0.782117
N	-1.058928	-1.693765	-0.087570
C	-2.127219	-2.410668	0.098199
O	-2.061851	-3.640526	-0.468981
C	-0.815127	-3.756945	-1.087901
C	-3.326089	-2.098453	0.861303
C	-3.267929	-1.167232	1.904241
C	-4.410927	-0.892611	2.644777
C	-5.608109	-1.543967	2.349883
C	-5.663339	-2.481141	1.317272
C	-4.523808	-2.767643	0.576113
Pd	-0.718256	0.323495	-0.177860
N	-0.283019	2.300424	-0.562346
C	-0.803492	3.291105	0.182479
C	-0.513349	4.626327	-0.063828
C	0.332163	4.952038	-1.118988
C	0.861145	3.924344	-1.894895
C	0.536181	2.613349	-1.582973
O	-0.495645	-4.751589	-1.684032
O	-2.775379	0.661320	-0.674379
C	-3.603636	1.319514	0.018992
C	-5.015165	1.362527	-0.638035
F	-5.026741	2.250482	-1.658898
O	-3.451412	1.923396	1.079575
F	-5.380883	0.172118	-1.144254
F	-5.971578	1.738650	0.223467
C	4.096358	1.844519	1.676839
H	1.463180	1.770616	1.296342

H	-4.561903	-3.492740	-0.229376
H	-6.595589	-2.986328	1.088359
H	-6.500258	-1.321668	2.926570
H	-4.367557	-0.164696	3.447247
H	-2.334896	-0.661978	2.130711
H	0.940473	1.782949	-2.149079
H	1.522024	4.126120	-2.729360
H	0.574239	5.987236	-1.334501
H	-0.953040	5.390211	0.566336
H	-1.479572	2.987524	0.973265
H	1.695872	-3.038833	-1.500423
H	3.592726	-2.994636	-1.650060
H	5.990802	-3.054933	-1.522386
C	7.596380	-1.314314	-0.195985
C	6.192279	0.748972	1.098002
C	5.448650	1.794330	1.753549
C	8.283916	-0.341478	0.521537
C	7.588827	0.681160	1.163786
H	3.543929	2.638640	2.170985
H	5.997121	2.547329	2.312543
H	8.130260	1.438046	1.724469
H	9.366799	-0.378960	0.582583
H	8.140054	-2.111388	-0.695387

- **Complex 3d**

58

	X	Y	Z
C	0.298724	3.174950	0.332849
N	0.673543	2.176543	-0.484854
C	1.443401	2.459094	-1.550626
C	1.867654	3.748175	-1.834331
C	1.493542	4.782748	-0.981335
C	0.694840	4.488178	0.118818
Pd	0.144533	0.221452	-0.116073
C	2.055918	-0.188443	0.265900
C	2.841079	0.766783	0.915698
C	4.222365	0.591022	1.037804
C	4.860009	-0.539458	0.508539
C	4.092743	-1.510125	-0.111415
C	2.673394	-1.370651	-0.216230
O	5.040760	1.480924	1.636076
C	4.482222	2.676518	2.166984
O	4.614500	-2.640094	-0.632966
C	6.015877	-2.857024	-0.543509
C	1.935256	-2.492600	-0.714805
C	0.595584	-2.671081	-0.605123
N	-0.310127	-1.774308	-0.012257
C	-1.432675	-2.413319	0.127591
O	-1.406818	-3.670160	-0.377472
C	-0.125219	-3.894706	-0.895759
O	0.165714	-4.941295	-1.417679
C	-2.662161	-1.992556	0.787400
C	-3.885941	-2.545142	0.388455
C	-5.056963	-2.155227	1.027016
C	-5.009309	-1.231025	2.071519

C	-3.788091	-0.697104	2.481006
C	-2.612897	-1.075614	1.842917
O	-1.853496	0.651538	-0.747436
C	-2.648477	1.416236	-0.127726
C	-4.003446	1.600563	-0.873637
F	-4.993774	1.971099	-0.047684
O	-2.497617	2.036686	0.923143
F	-3.883692	2.569542	-1.811542
F	-4.408472	0.486866	-1.505863
H	2.384004	1.665422	1.306206
H	5.934185	-0.621758	0.609580
H	3.997314	3.266456	1.381573
H	5.320800	3.236156	2.578660
H	3.763467	2.455538	2.962626
H	6.568540	-2.074385	-1.073507
H	6.196223	-3.818679	-1.021486
H	6.339084	-2.899810	0.501707
H	-3.917793	-3.260513	-0.426148
H	-6.007507	-2.569721	0.708381
H	-5.925622	-0.927690	2.567756
H	-3.749692	0.019715	3.293786
H	-1.660930	-0.664968	2.162089
H	1.727821	1.621142	-2.175657
H	2.485317	3.927265	-2.706241
H	1.817443	5.800175	-1.172687
H	0.372291	5.259757	0.807681
H	-0.345726	2.897935	1.157876
H	2.492868	-3.323982	-1.134563

- **Complex 3e**

56

	X	Y	Z
C	-2.278957	-1.012424	1.675167
C	-2.259670	-1.640328	0.430066
C	-3.453710	-1.710500	-0.289004
C	-4.624941	-1.160066	0.204846
C	-4.617900	-0.536317	1.448552
C	-3.446294	-0.465884	2.188139
C	-1.026512	-2.230382	-0.092529
N	0.135831	-1.675239	-0.198569
C	1.015625	-2.671481	-0.647442
C	0.243411	-3.874702	-0.871375
O	-1.061888	-3.524707	-0.465230
C	2.371251	-2.566057	-0.673969
C	3.138463	-1.455728	-0.211870
C	2.563759	-0.220532	0.197750
C	3.371711	0.711646	0.848619
C	4.737414	0.469190	1.035633
C	5.337470	-0.709691	0.572480
C	4.546596	-1.662427	-0.043742
Pd	0.694380	0.282456	-0.275404
O	-1.236683	0.830827	-1.027933
C	-2.076863	1.534194	-0.395319
O	-2.050654	1.944872	0.763977
O	5.573081	1.336006	1.637263

C	5.060113	2.580705	2.100301
O	5.028617	-2.833117	-0.503853
C	6.408646	-3.128518	-0.331126
O	0.494709	-4.982673	-1.269516
N	1.284177	2.232372	-0.548756
C	0.812806	3.204672	0.251340
C	1.203610	4.528487	0.106396
C	2.100341	4.861334	-0.903844
C	2.575055	3.853684	-1.738832
C	2.148779	2.551413	-1.527854
C	-3.301212	1.943027	-1.267134
F	-3.648644	0.992841	-2.152381
F	-4.386323	2.204470	-0.521766
F	-3.015635	3.060407	-1.968889
H	2.948936	1.643617	1.195865
H	6.401244	-0.840757	0.720183
H	4.641207	3.163853	1.273228
H	5.911854	3.109199	2.525478
H	4.299896	2.429112	2.872848
H	7.033354	-2.400732	-0.858658
H	6.553943	-4.116757	-0.764360
H	6.672906	-3.149327	0.730965
F	-3.473349	-2.284107	-1.489795
F	-5.750138	-1.215068	-0.502928
F	-5.734932	-0.002089	1.923949
F	-3.443767	0.133204	3.374970
F	-1.172498	-0.941366	2.410161
H	2.506891	1.732962	-2.140453
H	3.271094	4.064006	-2.541982
H	2.422616	5.888062	-1.039991
H	0.802246	5.278057	0.777815
H	0.100692	2.897485	1.008457
H	2.907737	-3.455878	-0.988614

- **Complex 3f**

58

	X	Y	Z
C	-2.342820	-0.985070	1.401935
C	-2.319223	-1.863649	0.315294
C	-3.510612	-2.314993	-0.264194
C	-4.727839	-1.857564	0.217489
C	-4.725145	-0.969335	1.287972
C	-3.556399	-0.529297	1.896299
C	-1.037958	-2.345855	-0.197053
N	0.111088	-1.744956	-0.206939
C	1.046379	-2.673727	-0.692073
C	0.325794	-3.876798	-1.054929
O	-1.003597	-3.603695	-0.687483
C	2.396741	-2.533728	-0.647313
C	3.107041	-1.433856	-0.074321
C	2.476432	-0.231369	0.341616
C	3.214207	0.698213	1.075665
C	4.569151	0.480112	1.346616
C	5.226692	-0.669167	0.886384
C	4.503365	-1.616093	0.183399

Pd	0.630116	0.236608	-0.238961
O	-1.280637	0.725257	-1.067529
C	-2.129698	1.489544	-0.522534
O	-2.094409	2.069629	0.560870
O	5.342559	1.344202	2.032137
C	4.768979	2.560399	2.498232
O	5.043928	-2.759837	-0.282158
C	6.418872	-3.023540	-0.035994
O	0.636456	-4.937607	-1.532838
N	1.242507	2.180165	-0.526014
C	0.777128	3.181330	0.240595
C	1.215131	4.489467	0.086110
C	2.154272	4.776202	-0.899126
C	2.623097	3.739054	-1.700712
C	2.148958	2.455051	-1.480939
C	-3.385623	1.717528	-1.415401
F	-3.746142	0.613392	-2.091927
F	-4.451440	2.111099	-0.702584
F	-3.135462	2.682346	-2.328927
H	2.745434	1.610081	1.418391
H	6.280560	-0.783432	1.103371
H	4.394530	3.162730	1.663465
H	5.575756	3.092751	2.999611
H	3.960312	2.366354	3.209935
H	7.052878	-2.259616	-0.497493
H	6.619551	-3.990589	-0.494354
H	6.619667	-3.077012	1.038867
H	-3.484843	-3.003233	-1.100857
H	-5.661421	-2.175301	-0.228312
N	-6.011402	-0.475224	1.796153
H	-3.596025	0.162235	2.727370
H	-1.416220	-0.651830	1.855393
H	2.501888	1.615265	-2.067262
H	3.351592	3.912234	-2.483718
H	2.513645	5.789641	-1.041941
H	0.815593	5.263357	0.730531
H	0.026974	2.911152	0.973702
H	2.972817	-3.385373	-0.995663
O	-5.997480	0.276068	2.761365
O	-7.031508	-0.839264	1.227087

- **Complex 3g**

55

	X	Y	Z
Pd	-1.109489	0.036164	-0.094721
F	3.365754	1.488942	1.647532
F	2.370902	3.285773	2.344181
F	2.641248	2.956674	0.224766
O	1.380114	-3.261829	0.753712
O	0.127000	-4.845542	1.790894
O	0.690338	1.167548	0.192475
O	0.430867	1.401200	2.428057
N	-0.157441	-1.754907	0.184700
N	-2.050896	1.860886	-0.153962
C	-2.833030	-0.913641	-0.407881

C	-3.833531	-0.311966	-1.184236
H	-3.624652	0.623902	-1.694078
C	-5.115163	-0.852123	-1.332247
C	-5.424756	-2.043555	-0.662943
H	-6.421187	-2.469108	-0.741955
C	-4.452805	-2.685829	0.081909
H	-4.684863	-3.626976	0.574097
C	-3.149425	-2.156980	0.195837
C	-6.145102	-0.170978	-2.194714
H	-5.840822	0.846382	-2.452954
H	-7.113662	-0.124485	-1.687789
H	-6.292282	-0.725931	-3.127849
C	-2.167572	-2.998784	0.840925
C	-0.830211	-2.821520	0.795590
C	0.174767	-3.791923	1.212900
C	1.109706	-2.067379	0.167787
C	2.189276	-1.316927	-0.407442
C	3.467774	-1.737211	-0.349061
C	4.624013	-1.025210	-0.893974
C	5.876283	-1.656726	-0.860896
H	5.960584	-2.652703	-0.434821
C	7.007019	-1.022872	-1.368618
H	7.968300	-1.525525	-1.337566
C	6.901406	0.256404	-1.911396
H	7.781548	0.755430	-2.304516
C	5.661222	0.898890	-1.943173
H	5.577623	1.898868	-2.356612
C	4.531846	0.266018	-1.440009
H	3.580487	0.787579	-1.458946
C	-1.601633	2.808133	-0.994874
H	-0.784541	2.523728	-1.646862
C	-2.144375	4.084294	-1.026042
H	-1.755022	4.812956	-1.726926
C	-3.177063	4.398351	-0.147393
H	-3.618202	5.389238	-0.145957
C	-3.631188	3.419731	0.731598
H	-4.427699	3.618252	1.438629
C	-3.047619	2.161977	0.696049
H	-3.376498	1.365674	1.352327
C	1.002519	1.568710	1.354154
C	2.353051	2.344932	1.386904
H	-2.513755	-3.910998	1.320650
H	1.896185	-0.383458	-0.869471
H	3.683171	-2.684616	0.140673

- **Complex 3h**

60

	X	Y	Z
C	-2.279942	2.587703	0.968918
N	-1.360781	2.227302	0.056962
C	-0.840306	3.165331	-0.752245
C	-1.235102	4.493820	-0.690209
C	-2.188626	4.869360	0.251553
C	-2.714240	3.898476	1.098863
Pd	-0.658036	0.297307	-0.040056

C	-2.498569	-0.397165	-0.375319
C	-3.394543	0.410125	-1.083700
C	-4.738884	0.049212	-1.208598
C	-5.231902	-1.125179	-0.624725
C	-4.352597	-1.954801	0.048659
C	-2.964711	-1.626384	0.157173
O	-5.656935	0.789929	-1.864423
C	-2.096334	-2.628020	0.700144
C	-0.742269	-2.621324	0.643824
N	0.059325	-1.609876	0.098180
C	1.275451	-2.079663	0.039576
O	1.389085	-3.337573	0.532696
C	0.123624	-3.740118	0.968846
O	-0.053484	-4.823311	1.466499
C	2.442334	-1.435229	-0.495911
C	3.658173	-2.013543	-0.468954
C	4.897556	-1.421774	-0.975622
C	6.067985	-2.194743	-0.950237
C	7.273133	-1.678984	-1.419282
C	7.325622	-0.378309	-1.916875
C	6.168144	0.403601	-1.942346
C	4.964757	-0.111232	-1.477274
O	1.267534	1.187381	0.246823
C	1.678489	1.445916	1.418018
C	3.090648	2.103011	1.444533
F	3.427086	2.693141	0.284927
O	1.153061	1.225981	2.505962
F	4.028732	1.165801	1.701034
F	3.186258	3.036589	2.406072
H	-3.052239	1.335458	-1.525351
H	-6.284864	-1.351813	-0.728590
O	-4.730665	-3.115986	0.623696
H	6.030053	-3.207882	-0.559408
H	8.169196	-2.290685	-1.393639
H	8.264099	0.028494	-2.280192
H	6.206964	1.419739	-2.321702
H	4.079910	0.516608	-1.492849
H	-0.089024	2.828965	-1.456624
H	-0.794325	5.214149	-1.368854
H	-2.514109	5.901359	0.325820
H	-3.453050	4.143475	1.852404
H	-2.670743	1.796122	1.596361
H	-2.549935	-3.524121	1.111386
H	2.268857	-0.446269	-0.899439
H	3.751828	-3.006705	-0.034613
C	-6.088180	-3.523321	0.526467
C	-5.252548	2.020008	-2.452258
H	-6.382507	-3.657841	-0.519549
H	-6.147744	-4.477124	1.048508
H	-6.750998	-2.798599	1.010501
H	-6.150257	2.440530	-2.903117
H	-4.865527	2.709477	-1.694127
H	-4.496191	1.856788	-3.226764

- **Complex 3i**

63

	X	Y	Z
C	8.729490	-1.502987	0.461956
C	8.552725	-0.347373	1.228791
C	7.286745	0.219754	1.379536
C	6.203581	-0.389724	0.749824
C	6.379923	-1.551222	-0.019137
C	7.641285	-2.113273	-0.168062
C	5.055185	-2.010640	-0.581934
C	4.093427	-0.961565	-0.065380
C	4.778556	-0.030898	0.721101
C	2.703641	-0.864063	-0.259901
C	1.994413	0.231881	0.304870
C	2.710641	1.127084	1.115671
C	4.082267	1.006128	1.335654
Pd	0.087235	0.597441	-0.134536
O	-1.891680	1.059702	-0.862458
C	-2.849353	1.458255	-0.141796
O	-2.920505	1.564331	1.084278
C	2.049471	-1.965924	-0.938992
C	0.724361	-2.212392	-0.913433
N	-0.245748	-1.410321	-0.301166
C	-1.351201	-2.101393	-0.274624
O	-1.243832	-3.316190	-0.872639
C	0.063287	-3.442869	-1.343226
C	-2.594040	-1.726812	0.334877
C	-3.710949	-2.468544	0.197204
C	-5.022739	-2.164713	0.767351
O	0.431230	-4.421914	-1.934594
N	0.450327	2.619743	-0.146546
C	-0.232087	3.426033	0.685459
C	-0.056318	4.802417	0.684699
C	0.846022	5.366920	-0.211658
C	1.544078	4.530089	-1.077406
C	1.323621	3.162336	-1.012330
C	-4.106102	1.824030	-0.984431
F	-4.562067	0.747638	-1.656383
F	-5.116861	2.281731	-0.233554
F	-3.822018	2.775476	-1.896272
H	4.593800	1.722723	1.972443
H	2.192007	1.954688	1.587893
H	1.855894	2.471882	-1.655227
H	2.254998	4.922676	-1.794511
H	1.002309	6.439991	-0.235802
H	-0.624208	5.412099	1.377255
H	-0.935755	2.940517	1.352529
H	7.783656	-3.011753	-0.761968
H	9.721815	-1.930437	0.355378
H	9.409399	0.113033	1.711423
H	7.154388	1.118103	1.975636
H	4.796996	-3.014630	-0.224739
H	5.070575	-2.053726	-1.677507
H	2.666328	-2.719763	-1.419736
C	-6.061838	-3.088076	0.574940
C	-7.331386	-2.849025	1.092890

C	-7.579929	-1.678735	1.808065
C	-6.554696	-0.749922	2.002093
C	-5.286037	-0.987325	1.488398
H	-5.869623	-3.999171	0.014931
H	-8.124534	-3.572974	0.936474
H	-8.569591	-1.487003	2.210624
H	-6.747434	0.165761	2.551825
H	-4.508160	-0.244703	1.638811
H	-3.654800	-3.379965	-0.394686
H	-2.562351	-0.811339	0.914393

- **Complex 3j**

60

	X	Y	Z
C	4.975490	-0.587572	-1.465114
C	4.775524	-1.847539	-0.876430
C	5.851830	-2.745683	-0.820647
C	7.093522	-2.402308	-1.348223
C	7.277518	-1.151000	-1.933685
C	6.215635	-0.244771	-1.988123
C	3.490892	-2.260099	-0.311149
C	2.340002	-1.564086	-0.389004
C	1.121068	-2.031892	0.205512
N	-0.040111	-1.436121	0.209769
C	-0.939434	-2.302111	0.845064
C	-0.180267	-3.466024	1.295178
O	1.112584	-3.240282	0.824322
C	-2.280612	-2.175757	0.892072
C	-3.046718	-1.150609	0.214919
C	-2.450600	-0.021252	-0.407801
C	-3.284073	0.768102	-1.201898
C	-4.651374	0.515726	-1.344118
C	-5.246096	-0.548100	-0.670810
C	-4.434141	-1.388105	0.104323
Pd	-0.554333	0.517723	-0.103444
O	1.456579	1.215213	0.150492
C	1.878289	1.550364	1.298301
O	1.318154	1.508883	2.390209
H	-5.243367	1.173228	-1.970675
O	-4.992892	-2.437108	0.778080
C	-5.355960	-3.552904	-0.039183
O	-0.467138	-4.463181	1.901644
N	-1.057442	2.506786	-0.193632
C	-1.957161	3.037235	0.652555
C	-2.233168	4.396323	0.678330
C	-1.565011	5.236521	-0.207086
C	-0.634562	4.684906	-1.083002
C	-0.402300	3.317998	-1.041776
C	3.358260	2.037122	1.279405
F	3.603920	2.945214	2.236764
F	3.716830	2.590130	0.107082
F	4.187457	0.992301	1.496173
H	-2.881226	1.625430	-1.730377
O	-6.568541	-0.851969	-0.704171
H	5.710654	-3.720301	-0.361523

H	7.915779	-3.108832	-1.299453
H	8.245341	-0.878308	-2.342563
H	6.358684	0.733950	-2.434729
H	4.167321	0.135529	-1.502137
H	0.326470	2.846952	-1.690301
H	-0.087625	5.298252	-1.788998
H	-1.763824	6.302783	-0.212827
H	-2.961223	4.779324	1.383282
H	-2.460866	2.343746	1.314541
H	-2.827130	-2.965372	1.397606
H	2.261996	-0.605474	-0.884830
H	3.488099	-3.214234	0.211760
H	-5.789152	-4.294901	0.631528
H	-6.090975	-3.260439	-0.794099
H	-4.466147	-3.970659	-0.524798
C	-7.417685	-0.038628	-1.497185
H	-8.418472	-0.453411	-1.383221
H	-7.412380	0.999683	-1.146804
H	-7.126814	-0.073235	-2.553247

- **Complex 3k**

52

	X	Y	Z
C	-1.370441	3.006779	-0.855695
N	-1.901105	2.009545	-0.128722
C	-2.960738	2.264757	0.656955
C	-3.530647	3.526720	0.737432
C	-2.990790	4.559331	-0.022915
C	-1.891080	4.291703	-0.832776
Pd	-0.995248	0.174757	-0.126279
O	0.802429	1.233861	0.219973
C	1.238632	1.381766	1.404692
C	2.493579	2.302330	1.438227
F	3.463481	1.842679	0.624051
N	-0.118613	-1.641786	0.189460
C	1.136012	-2.001028	0.215293
O	1.356651	-3.149950	0.904205
C	0.131736	-3.588519	1.401678
C	-0.839323	-2.619777	0.886344
C	2.244418	-1.339680	-0.408441
C	3.505745	-1.800708	-0.286061
C	4.702043	-1.212479	-0.884447
C	4.659929	-0.092684	-1.731978
C	5.830803	0.426474	-2.268553
C	7.062787	-0.161478	-1.970982
C	7.118267	-1.274394	-1.133996
C	5.945306	-1.796229	-0.596482
C	-2.178691	-2.737098	0.951686
C	-3.126357	-1.920859	0.206346
C	-2.728368	-0.751204	-0.487542
C	-3.683490	-0.237214	-1.362254
C	-4.975007	-0.724567	-1.511720
C	-5.327460	-1.820959	-0.742815
C	-4.431419	-2.439418	0.103455
O	0.036217	-4.579206	2.071948

H	-5.672980	-0.265013	-2.201363
H	-4.727298	-3.326514	0.652875
O	0.817199	0.925495	2.461573
F	3.020779	2.411306	2.664017
F	2.181833	3.548766	1.023079
F	-3.365281	0.819035	-2.153039
F	-6.582323	-2.311202	-0.850710
H	5.989382	-2.663376	0.056667
H	8.072364	-1.735274	-0.899952
H	7.975287	0.248543	-2.392154
H	5.785833	1.293054	-2.920212
H	3.713106	0.379285	-1.972466
H	-0.506197	2.754795	-1.457643
H	-1.433643	5.063214	-1.440416
H	-3.417630	5.555650	0.016931
H	-4.382138	3.686572	1.387898
H	-3.352099	1.429749	1.225517
H	-2.564639	-3.582838	1.513985
H	1.989409	-0.444858	-0.961812
H	3.671618	-2.692387	0.314931

- **Complex 3m**

60

	X	Y	Z
C	4.997957	-0.119846	-1.524122
C	4.972536	-1.418397	-0.988301
C	6.159511	-2.166079	-0.975090
C	7.340483	-1.637118	-1.488755
C	7.351579	-0.348577	-2.019518
C	6.177256	0.408115	-2.033695
C	3.759971	-2.022074	-0.434132
C	2.533363	-1.465567	-0.439568
C	1.393403	-2.117835	0.140228
N	0.168757	-1.671158	0.212140
C	-0.598677	-2.685119	0.800188
C	0.301150	-3.776090	1.140021
O	1.545995	-3.359266	0.666162
C	-1.948513	-2.718473	0.884000
C	-2.851746	-1.745707	0.327474
C	-2.421364	-0.524371	-0.233873
C	-3.383479	0.231364	-0.923634
C	-4.718804	-0.152705	-1.011357
C	-5.152778	-1.348189	-0.385581
C	-4.218042	-2.125973	0.263659
Pd	-0.588831	0.220930	0.018567
O	1.328523	1.154204	0.233521
C	1.734823	1.513155	1.379788
O	1.208704	1.381788	2.481417
O	-5.673314	0.550551	-1.652274
C	-5.311563	1.776931	-2.271558
H	-4.513852	-3.062238	0.723921
O	0.155334	-4.845969	1.672868
N	-1.327049	2.137950	0.037161
C	-2.229664	2.529503	0.953039

C	-2.687546	3.836695	1.024381
C	-2.203575	4.770645	0.113209
C	-1.266261	4.363506	-0.831724
C	-0.846083	3.041366	-0.833801
C	3.145343	2.173435	1.345068
F	3.288009	3.105298	2.300718
F	3.424803	2.764628	0.170163
F	4.095172	1.235571	1.554484
H	-3.086993	1.155874	-1.400352
O	-6.474454	-1.630979	-0.495768
H	6.153515	-3.169561	-0.558432
H	8.250005	-2.228907	-1.472014
H	8.271219	0.068489	-2.417706
H	6.184201	1.414796	-2.439193
H	4.099599	0.488604	-1.532157
H	-0.106265	2.682392	-1.539195
H	-0.857512	5.054812	-1.558883
H	-2.548436	5.798653	0.140847
H	-3.412197	4.107320	1.782884
H	-2.588251	1.765138	1.631408
H	-2.372811	-3.613509	1.332827
H	2.331643	-0.488472	-0.858230
H	3.884670	-3.003757	0.018121
H	-6.229706	2.169619	-2.706194
H	-4.920067	2.489078	-1.536519
H	-4.571614	1.616648	-3.062916
C	-6.947080	-2.818058	0.118436
H	-8.018822	-2.847734	-0.074237
H	-6.474133	-3.705467	-0.317888
H	-6.771291	-2.801451	1.200321

- **Complex 4d**

60

	X	Y	Z
C	-2.747488	-0.547004	2.097354
C	-3.010409	-1.365845	0.992796
C	-4.323044	-1.561626	0.551071
C	-5.367409	-0.912112	1.200662
C	-5.107201	-0.093864	2.300238
C	-3.799563	0.082117	2.752512
C	-1.898256	-2.024094	0.315218
N	-0.707427	-1.554172	0.099085
C	0.047820	-2.593092	-0.467091
C	-0.842047	-3.719798	-0.665391
O	-2.067500	-3.291607	-0.116270
C	1.397603	-2.588847	-0.611740
C	2.281905	-1.548322	-0.179877
C	1.836150	-0.282448	0.286670
C	2.757508	0.582314	0.876146
C	4.112206	0.239586	0.953454
C	4.582848	-0.975010	0.437838
C	3.677962	-1.859646	-0.122852
Pd	-0.027113	0.356703	-0.090062
N	-1.961769	1.022790	-0.770849
O	5.055192	1.037727	1.489867

C	4.670468	2.307375	2.004351
O	4.036246	-3.056778	-0.627473
C	5.403042	-3.446133	-0.580099
O	-0.721370	-4.820723	-1.136422
N	0.695224	2.248853	-0.438692
C	0.515172	3.235896	0.455489
C	1.012533	4.514719	0.254637
C	1.715903	4.786011	-0.915536
C	1.894224	3.764541	-1.843420
C	1.372383	2.508772	-1.569515
H	2.433752	1.541121	1.256233
H	5.641848	-1.187570	0.500982
H	4.224460	2.930415	1.221296
H	5.588830	2.770901	2.361103
H	3.968389	2.196828	2.836927
H	6.027061	-2.754401	-1.155038
H	5.442533	-4.435785	-1.032280
H	5.758205	-3.499598	0.453969
H	-4.519319	-2.201433	-0.302876
H	-6.385156	-1.046918	0.850449
H	-5.926632	0.403964	2.808560
H	-3.599638	0.709168	3.614884
H	-1.725620	-0.415896	2.440375
H	1.501334	1.680035	-2.255113
H	2.435277	3.927313	-2.767705
H	2.119462	5.775591	-1.099697
H	0.848572	5.276255	1.007482
H	-0.026891	2.977968	1.358462
H	1.838431	-3.500607	-1.002259
C	-2.751817	1.854489	-0.076371
C	-4.017565	2.219126	-0.511816
C	-4.482542	1.709721	-1.720250
C	-3.659631	0.857242	-2.450412
C	-2.409048	0.538497	-1.941082
H	-2.360262	2.218612	0.866648
H	-4.622978	2.882532	0.094057
H	-5.469951	1.970475	-2.085680
H	-3.976909	0.436908	-3.397284
H	-1.740549	-0.130854	-2.472996

- **Complex 4h**

64

	X	Y	Z
C	-5.031472	-0.145756	-1.742216
C	-5.058139	0.920434	-0.828447
C	-6.284769	1.289915	-0.257687
C	-7.452580	0.600435	-0.572956
C	-7.410957	-0.462435	-1.473332
C	-6.197779	-0.829545	-2.060702
C	-3.853222	1.644445	-0.420343
C	-2.587361	1.260643	-0.667175
C	-1.446584	1.979191	-0.166228
N	-0.224842	1.536177	-0.052730
C	0.552075	2.602599	0.423231
C	-0.339530	3.721424	0.663544

O	-1.596044	3.260389	0.238204
C	1.906719	2.633244	0.481408
C	2.788378	1.599060	0.028648
C	2.345335	0.310158	-0.367873
C	3.253508	-0.554281	-0.980329
C	4.592275	-0.184751	-1.149724
C	5.062645	1.057111	-0.704629
C	4.169945	1.939842	-0.122044
Pd	0.513678	-0.364570	0.106004
N	-1.381416	-1.112613	0.815304
O	5.521620	-0.979816	-1.715639
C	5.139647	-2.277125	-2.156980
O	4.527142	3.162667	0.319408
C	5.878339	3.581161	0.176753
O	-0.195562	4.837703	1.090508
N	1.297722	-2.235538	0.446021
C	2.012502	-2.466894	1.559685
C	2.569617	-3.707628	1.833302
C	2.386688	-4.744729	0.923973
C	1.644007	-4.503370	-0.228310
C	1.114342	-3.237974	-0.430347
H	2.933379	-1.532895	-1.308706
H	6.110665	1.291025	-0.837097
H	-6.319648	2.117965	0.444811
H	-8.392998	0.894759	-0.118536
H	-8.320090	-0.999578	-1.724161
H	-6.164300	-1.649053	-2.771474
H	-4.100563	-0.439435	-2.217058
H	0.544551	-3.003809	-1.322546
H	1.475369	-5.277347	-0.967315
H	2.816898	-5.722992	1.108253
H	3.140932	-3.846677	2.743132
H	2.143806	-1.627369	2.231472
H	2.347920	3.565974	0.818044
H	-2.349191	0.357126	-1.213365
H	-4.014939	2.544632	0.169388
H	6.170480	3.605812	-0.878052
H	5.919974	4.587022	0.591548
H	6.552611	2.925028	0.736557
H	6.044954	-2.730097	-2.558066
H	4.765040	-2.881052	-1.323184
H	4.380788	-2.217143	-2.943767
C	-1.845993	-0.660213	1.990586
C	-3.098095	-1.006214	2.479915
C	-3.901332	-1.856805	1.726820
C	-3.414293	-2.342548	0.516848
C	-2.151467	-1.948573	0.100210
H	-1.194287	0.011548	2.539648
H	-3.432075	-0.605921	3.429683
H	-4.890412	-2.134400	2.074673
H	-4.003707	-3.004626	-0.105954
H	-1.746532	-2.286949	-0.847725

- **Complex 5h**

  54

	X	Y	Z
C	-5.407793	1.118563	0.666594
C	-5.475194	-0.279499	0.545235
C	-6.700523	-0.919453	0.784344
C	-7.825470	-0.188220	1.155921
C	-7.743435	1.197431	1.283025
C	-6.532747	1.847767	1.033288
C	-4.318548	-1.095521	0.168767
C	-3.043439	-0.667170	0.119634
C	-1.958788	-1.525959	-0.267975
N	-0.690227	-1.231443	-0.330587
C	-0.014100	-2.416434	-0.654870
C	-1.013210	-3.446063	-0.874888
O	-2.226033	-2.820343	-0.575861
C	1.320423	-2.628414	-0.544388
C	2.272664	-1.699328	-0.011895
C	1.948410	-0.357552	0.314589
C	2.864853	0.391114	1.058740
C	4.106919	-0.142734	1.411970
C	4.474612	-1.439799	1.029658
C	3.564797	-2.210329	0.326972
Pd	0.317223	0.546578	-0.428829
Cl	-1.515286	1.744694	-1.526750
O	5.043321	0.535042	2.109285
C	4.773130	1.877675	2.492178
O	3.820184	-3.476834	-0.064904
C	5.069193	-4.063601	0.272195
O	-0.968364	-4.605673	-1.201388
N	1.412186	2.272739	-0.636831
C	2.460485	2.323865	-1.474782
C	3.224766	3.471002	-1.631608
C	2.902580	4.601557	-0.887115
C	1.813996	4.546428	-0.021653
C	1.087415	3.367951	0.069593
H	2.626464	1.405796	1.345132
H	5.456451	-1.801700	1.305078
H	-6.767401	-1.999244	0.682443
H	-8.764720	-0.699114	1.342274
H	-8.619691	1.771162	1.568141
H	-6.468686	2.927916	1.118092
H	-4.480269	1.642284	0.456100
H	0.225188	3.283129	0.720023
H	1.521028	5.401581	0.575611
H	3.487623	5.510086	-0.981151
H	4.059397	3.466028	-2.322408
H	2.687589	1.414337	-2.017582
H	1.663694	-3.631771	-0.775334
H	-2.752195	0.346334	0.362878
H	-4.530748	-2.132707	-0.082235
H	5.201201	-4.106821	1.358310
H	5.039176	-5.073980	-0.132889
H	5.897869	-3.511030	-0.182636
H	5.665309	2.220822	3.014002
H	4.595570	2.508563	1.614234
H	3.912182	1.929675	3.166505

- **Complex 6h**

96

	X	Y	Z
O	-0.130713	5.246952	-1.289014
C	-0.387764	4.080700	-1.121363
O	-1.714159	3.622696	-1.110514
C	-1.681139	2.286917	-0.877989
C	-2.906709	1.539278	-0.766528
C	-4.126438	2.082100	-0.921006
C	-5.373154	1.337311	-0.703965
C	-5.445866	0.310298	0.250104
C	-6.637133	-0.382108	0.441060
C	-7.764505	-0.068627	-0.318006
C	-7.703300	0.957824	-1.261139
C	-6.518795	1.666316	-1.442636
N	-0.475514	1.816404	-0.756648
C	0.407069	2.880397	-0.936631
C	1.747290	2.752071	-1.115758
C	2.460428	1.521064	-1.298393
C	3.784702	1.640390	-1.812651
O	4.334489	2.871274	-1.758383
C	5.624912	3.086259	-2.311742
C	4.449240	0.553593	-2.374790
C	3.788642	-0.675537	-2.403184
O	4.302382	-1.781523	-2.979265
C	5.590798	-1.715066	-3.573913
C	2.542258	-0.841977	-1.784431
C	1.868591	0.218421	-1.198268
Pd	0.219334	-0.015250	-0.095982
C	0.759964	-1.862383	0.465067
N	-0.007865	-2.953605	0.221274
C	-1.320306	-2.895698	-0.369504
C	-2.426843	-2.926661	0.489590
C	-2.259520	-3.089998	1.976501
C	-3.694132	-2.795709	-0.077345
C	-3.873931	-2.636741	-1.453569
C	-5.247340	-2.443609	-2.043667
C	-2.746070	-2.645089	-2.274123
C	-1.456133	-2.777430	-1.755945
C	-0.270915	-2.771108	-2.682682
C	0.568424	-4.093705	0.760636
C	1.716842	-3.702590	1.358351
N	1.819336	-2.332354	1.167580
C	2.851890	-1.490768	1.705208
C	4.137432	-1.532258	1.158726
C	4.560895	-2.584454	0.168266
C	5.068587	-0.601024	1.625231
C	4.752274	0.323424	2.620220
C	5.751428	1.365079	3.054450
C	3.485351	0.261818	3.206824
C	2.522796	-0.650055	2.777685
C	1.206982	-0.783302	3.499327
O	-1.428384	-0.115143	1.301027
C	-1.953865	0.624920	2.178195

C	-1.166866	1.900519	2.634432
F	-1.729262	3.005721	2.092864
F	0.129725	1.915393	2.285777
F	-1.213061	2.046941	3.970324
O	-3.048889	0.484743	2.722845
H	-2.778314	0.489967	-0.520796
H	-4.216597	3.123960	-1.222539
H	-4.581428	0.079820	0.867688
H	-6.686306	-1.168128	1.188462
H	-8.690894	-0.614478	-0.168134
H	-8.580052	1.211869	-1.848523
H	-6.474601	2.472482	-2.169978
H	2.296069	3.674933	-1.272346
H	5.635310	2.864866	-3.384038
H	5.838582	4.142602	-2.155955
H	6.378517	2.481689	-1.796386
H	5.436661	0.666600	-2.797291
H	6.354047	-1.455832	-2.831639
H	5.789254	-2.712040	-3.964448
H	5.609584	-0.990568	-4.394732
H	2.140394	-1.846157	-1.765851
H	-1.935799	-4.104945	2.231423
H	-3.202658	-2.895604	2.490728
H	-1.513886	-2.389473	2.360627
H	-4.563028	-2.802890	0.575773
H	-6.021256	-2.870954	-1.401184
H	-5.469809	-1.375826	-2.152801
H	-5.320748	-2.904298	-3.032852
H	-2.868753	-2.547311	-3.350424
H	-0.561906	-3.118428	-3.676854
H	0.131201	-1.758149	-2.784041
H	0.534751	-3.413624	-2.317138
H	0.106439	-5.063390	0.669340
H	2.465654	-4.260046	1.897124
H	3.743938	-2.911948	-0.474862
H	4.937413	-3.465136	0.701413
H	5.367184	-2.214598	-0.468783
H	6.067458	-0.602505	1.194784
H	5.636235	1.609754	4.113918
H	6.777832	1.027735	2.887052
H	5.608445	2.290343	2.485120
H	3.241676	0.935876	4.024427
H	1.133656	-1.769416	3.971630
H	0.356325	-0.684339	2.824011
H	1.117502	-0.025321	4.279687

10.1.2. M06-2X

***Table S3**: calculated orbital energies (hartree) and metal participation, along with the HOMO – LUMO gap (eV) using the M06-2X functional.*

Compound	HOMO-2	HOMO-1	HOMO	Δ_{H-L}	LUMO	ϕ
**3a**	-0.30816	-0.28605	-0.26816 (13.4%)	4.994	-0.08465 (2.0%)	5%
**3b**	-0.30793	-0.30009	-0.26498 (7.7%)	4.875	-0.08582 (2.2%)	4%
**3c**	-0.30576	-0.28098	-0.25150 (2.7%)	4.395	-0.09000 (3.6%)	<1%
**3d**	-0.31155	-0.29371	-0.26227 (7.7%)	5.038	-0.07712 (4.8%)	7%
**3e**	-0.31541	-0.29723	-0.26875 (7.8%)	5.029	-0.08394 (5.3%)	5%
**3f**	-0.31542	-0.29658	-0.26686 (9.1%)	5.610	-0.06067 (4.1%)	4%
**3g**	-0.30702	-0.30066	-0.26732 (9.5%)	4.969	-0.08508 (5.0%)	<1%
**3h**	-0.30039	-0.29203	-0.25686 (4.5%)	4.774	-0.08144 (4.8%)	18 %
**3i**	-0.30570	-0.27809	-0.26608 (12.8%)	4.825	-0.08876 (2.4%)	3%
**3j**	-0.30701	-0.27728	-0.26946 (14.0%)	4.972	-0.08673 (3.7%)	<1%
**3k**	-0.31539	-0.30217	-0.27780 (21.7%)	5.071	-0.09146 (14.8%)	<1%
**3m**	-0.30511	-0.28822	-0.25423 (10.5%)	4.649	-0.08339 (2.6%)	<1%
**4d**	-0.33984	-0.32357	-0.29440 (3.0%)	6.531	-0.05439 (3.9%)	10%
**4h**	-0.31099	-0.30305	-0.26771 (5.4%)	4.766	-0.09330 (4.1%)	28%
**5h**	-0.29775	-0.28984	-0.25588 (11.0%)	4.782	-0.08013 (14.5%)	12%
**6h**	-0.29283	-0.28190	-0.25091 (4.2%)	4.731	-0.07707 (5.5%)	15%

Cartesian Coordinates

- **Complex: 3a**

59

	X	Y	Z
C	-0.839983	3.447198	0.308847
N	-0.134347	2.535630	-0.380530
C	0.739830	2.954586	-1.311472
C	0.937790	4.300244	-1.590291
C	0.214688	5.248646	-0.870880
C	-0.687157	4.812916	0.094773
Pd	-0.415483	0.473808	-0.067249
C	1.504001	0.294175	0.296666
C	2.151822	1.269853	1.068109
C	3.534818	1.268039	1.260208
C	4.301801	0.277717	0.649437
C	3.681288	-0.722101	-0.108756
C	2.284463	-0.746559	-0.270117
H	3.998916	2.039557	1.868668
C	4.716269	-1.695289	-0.633315
C	1.705997	-1.901452	-0.932205

C	0.419585	-2.291056	-0.831230
N	-0.603871	-1.636779	-0.128490
C	-1.616452	-2.449323	-0.106441
O	-1.409930	-3.610219	-0.785701
C	-0.120659	-3.567997	-1.309166
O	0.330089	-4.461240	-1.969035
C	-2.895175	-2.315912	0.575814
C	-3.963648	-3.145505	0.207526
C	-5.180629	-3.029713	0.869436
C	-5.330027	-2.099612	1.899500
C	-4.260076	-1.284983	2.274535
C	-3.039055	-1.394517	1.619522
O	-2.523674	0.667261	-0.617284
C	-3.391598	1.169724	0.150605
C	-4.821889	1.090291	-0.448530
F	-5.772334	1.383234	0.446904
O	-3.265559	1.697298	1.255036
F	-4.954475	1.960750	-1.470268
F	-5.090728	-0.132148	-0.933424
H	1.569437	2.064192	1.524998
C	5.752582	0.038735	0.651510
H	-3.839706	-3.862619	-0.596526
H	-6.013124	-3.662060	0.580485
H	-6.282161	-2.009784	2.412630
H	-4.375818	-0.560237	3.073090
H	-2.203081	-0.765791	1.910844
H	1.294343	2.182928	-1.834184
H	1.649890	4.589167	-2.354090
H	0.352342	6.307877	-1.060829
H	-1.273157	5.511955	0.679622
H	-1.545151	3.055272	1.034446
C	6.009615	-1.117033	-0.105382
C	6.793913	0.746841	1.249478
C	8.099430	0.284359	1.078399
C	7.310666	-1.573930	-0.274527
C	8.356583	-0.864515	0.323312
H	7.515122	-2.466377	-0.859354
H	9.378714	-1.208780	0.201157
H	8.924481	0.821417	1.535651
H	6.598705	1.639806	1.836351
H	4.549973	-2.711998	-0.257773
H	4.712570	-1.751099	-1.728669
H	2.365112	-2.574836	-1.475157

- **Complex: 3b**

54

	X	Y	Z
C	-2.637075	-1.329542	1.556208
C	-2.475506	-2.247511	0.511919
C	-3.555813	-3.030357	0.080922
C	-4.799503	-2.872349	0.681603
C	-4.965354	-1.945214	1.711828
C	-3.885008	-1.176371	2.148688
C	-1.170725	-2.423864	-0.108118
N	-0.128900	-1.649186	-0.071367

C	0.901348	-2.326286	-0.738282
C	0.341490	-3.576041	-1.258168
O	-0.972211	-3.581666	-0.792531
C	2.206655	-1.983587	-0.786374
C	2.814591	-0.866376	-0.090131
C	2.036386	0.168746	0.439382
C	2.654266	1.132777	1.280066
C	4.000771	1.098309	1.539457
C	4.834160	0.109164	0.954047
C	4.248569	-0.891732	0.127937
Pd	0.146676	0.439387	-0.008522
O	-1.924116	0.734470	-0.641135
C	-2.806010	1.258285	0.096572
O	-2.708261	1.768542	1.211842
H	4.452069	1.843326	2.190026
C	5.116462	-1.871214	-0.429787
O	0.789526	-4.476699	-1.911008
N	0.539282	2.484424	-0.315904
C	-0.179216	3.427850	0.314818
C	0.036868	4.784621	0.099045
C	1.018489	5.176773	-0.805734
C	1.755376	4.195406	-1.464308
C	1.489949	2.860863	-1.188021
C	-4.211067	1.238490	-0.564094
F	-4.502647	0.033562	-1.078285
F	-5.187923	1.554084	0.294315
F	-4.267634	2.127125	-1.577238
H	2.051676	1.919009	1.723822
H	-3.419231	-3.744873	-0.723417
H	-5.640093	-3.468996	0.344162
H	-5.938313	-1.821854	2.176626
H	-4.012912	-0.453017	2.946712
H	-1.793957	-0.736966	1.897810
H	2.049763	2.063640	-1.665135
H	2.528364	4.450348	-2.179426
H	1.206689	6.228239	-0.995487
H	-0.562567	5.510864	0.635120
H	-0.944862	3.069227	0.995346
H	2.830871	-2.687442	-1.325912
C	6.471400	-1.843204	-0.190800
C	6.234119	0.109998	1.192754
C	7.043850	-0.845266	0.630962
H	4.730075	-2.659694	-1.063496
H	7.107113	-2.600354	-0.638681
H	8.113246	-0.839727	0.813732
H	6.649840	0.885482	1.830313

- **Complex: 3c**

62

	X	Y	Z
C	6.184481	-1.342308	-0.175393
C	5.436728	-2.330797	-0.899977
C	4.082532	-2.272879	-0.996068
C	3.322721	-1.206333	-0.390094
C	4.046382	-0.213529	0.327150

C	5.470501	-0.281789	0.440056
C	1.897225	-1.112917	-0.463148
C	1.223263	0.006146	0.093701
C	1.958254	0.949028	0.805240
C	3.349832	0.856970	0.950394
C	1.158540	-2.225110	-1.020511
C	-0.154324	-2.481307	-0.825046
N	-1.074975	-1.706613	-0.106711
C	-2.153102	-2.420882	0.012012
O	-2.084638	-3.627872	-0.610166
C	-0.826915	-3.726052	-1.202062
C	-3.376587	-2.134367	0.746463
C	-3.383653	-1.150095	1.741819
C	-4.554150	-0.895919	2.447032
C	-5.710343	-1.628080	2.170716
C	-5.698099	-2.620033	1.188868
C	-4.532678	-2.880292	0.477121
Pd	-0.684811	0.362826	-0.203197
N	-0.209137	2.360022	-0.675367
C	-0.815151	3.383951	-0.052275
C	-0.541451	4.708925	-0.374887
C	0.378809	4.982868	-1.381680
C	0.998996	3.918527	-2.031633
C	0.683057	2.622796	-1.645782
O	-0.493661	-4.690120	-1.833560
O	-2.787192	0.746221	-0.646980
C	-3.552050	1.357136	0.151478
C	-5.023183	1.380286	-0.344757
F	-5.155386	2.229997	-1.383985
O	-3.300946	1.922090	1.215313
F	-5.425468	0.172100	-0.768195
F	-5.879981	1.778035	0.603264
C	4.089551	1.845957	1.689703
H	1.458568	1.803740	1.254038
H	-4.515186	-3.646570	-0.290126
H	-6.597119	-3.188301	0.976068
H	-6.622217	-1.425214	2.723205
H	-4.563319	-0.123548	3.208409
H	-2.482155	-0.584868	1.958034
H	1.154353	1.764989	-2.113101
H	1.720567	4.079770	-2.823662
H	0.609480	6.006811	-1.656289
H	-1.050104	5.502570	0.159476
H	-1.537002	3.116472	0.712621
H	1.681803	-3.001223	-1.567564
H	3.581707	-3.051214	-1.557193
H	5.978829	-3.141893	-1.378172
C	7.581127	-1.395733	-0.059364
C	6.180732	0.710563	1.171499
C	5.440828	1.777037	1.795479
C	8.268826	-0.419658	0.655776
C	7.575583	0.623819	1.267044
H	3.536955	2.654293	2.160081
H	5.990897	2.529225	2.354252
H	8.114732	1.383252	1.826519

H	9.349401	-0.471425	0.739392
H	8.121219	-2.210052	-0.534235

- **Complex: 3d**

56

	X	Y	Z
C	0.351555	3.282692	0.042253
N	0.835639	2.207532	-0.600328
C	1.760352	2.381324	-1.559887
C	2.234691	3.637305	-1.913432
C	1.744368	4.753969	-1.240401
C	0.787744	4.571629	-0.246681
Pd	0.183654	0.260911	-0.135118
C	2.040463	-0.220871	0.291837
C	2.825347	0.706303	0.980726
C	4.193757	0.474724	1.160710
C	4.810046	-0.675509	0.646595
C	4.033686	-1.611198	-0.015743
C	2.626437	-1.419564	-0.177823
O	5.019483	1.326751	1.804041
C	4.474615	2.534444	2.321118
O	4.535891	-2.756860	-0.526079
C	5.927834	-3.006683	-0.383717
C	1.869318	-2.511743	-0.724264
C	0.525483	-2.658695	-0.636722
N	-0.374626	-1.769302	-0.028719
C	-1.514626	-2.385387	0.045831
O	-1.504619	-3.625496	-0.509254
C	-0.217237	-3.860234	-0.996975
O	0.071517	-4.887039	-1.550377
C	-2.757120	-1.963076	0.678918
C	-3.954926	-2.612174	0.349392
C	-5.138745	-2.223632	0.966719
C	-5.129459	-1.198817	1.914206
C	-3.933169	-0.562979	2.251042
C	-2.744320	-0.946937	1.641061
O	-1.838847	0.791070	-0.752241
C	-2.591468	1.511691	-0.037896
C	-4.008399	1.668668	-0.653384
F	-4.880020	2.225593	0.195805
O	-2.363906	2.086434	1.025839
F	-3.962612	2.453498	-1.749315
F	-4.518498	0.487223	-1.034394
H	2.379876	1.619674	1.352518
H	5.874896	-0.798792	0.793621
H	4.050096	3.145529	1.517349
H	5.307496	3.061888	2.782370
H	3.707837	2.325796	3.073305
H	6.515116	-2.228363	-0.880733
H	6.107367	-3.966378	-0.864609
H	6.204934	-3.064876	0.673387
H	-3.953912	-3.405119	-0.390552
H	-6.068773	-2.717565	0.706500
H	-6.055343	-0.895812	2.392636
H	-3.924111	0.235289	2.985378

H	-1.811293	-0.458493	1.906248
H	2.128878	1.482421	-2.042345
H	2.977327	3.727936	-2.697194
H	2.101647	5.748055	-1.487838
H	0.374383	5.409126	0.302754
H	-0.407765	3.087266	0.792103
H	2.412837	-3.344303	-1.161362

- **Complex: 3e**

56

	X	Y	Z
C	-2.240499	-0.932885	1.646269
C	-2.261519	-1.620243	0.432380
C	-3.477457	-1.717802	-0.247243
C	-4.629148	-1.136698	0.254638
C	-4.582441	-0.456650	1.467524
C	-3.390454	-0.360481	2.169200
C	-1.041671	-2.217772	-0.106950
N	0.130220	-1.681238	-0.162057
C	0.998716	-2.661595	-0.657417
C	0.203902	-3.844346	-0.955700
O	-1.100328	-3.490659	-0.553880
C	2.352867	-2.563868	-0.689347
C	3.122971	-1.464593	-0.187930
C	2.566404	-0.223242	0.218942
C	3.367723	0.698796	0.891049
C	4.725479	0.429883	1.110346
C	5.315358	-0.757098	0.653149
C	4.522755	-1.692878	0.011576
Pd	0.736917	0.309240	-0.268797
O	-1.235898	0.864344	-1.034463
C	-2.097781	1.495979	-0.362070
O	-2.047773	1.917626	0.793259
O	5.561214	1.278099	1.739591
C	5.045383	2.520623	2.204546
O	4.998770	-2.870367	-0.442825
C	6.376502	-3.164947	-0.249026
O	0.446244	-4.929815	-1.405992
N	1.375299	2.275876	-0.643379
C	0.748259	3.303895	-0.048150
C	1.108139	4.625225	-0.288936
C	2.139658	4.890244	-1.184719
C	2.778559	3.822075	-1.809915
C	2.371508	2.529250	-1.508539
C	-3.397626	1.787722	-1.164171
F	-3.724240	0.782313	-1.992470
F	-4.450522	1.995224	-0.360486
F	-3.242400	2.891681	-1.917240
H	2.946080	1.637077	1.225217
H	6.372526	-0.907622	0.827088
H	4.653739	3.114754	1.372175
H	5.887285	3.037686	2.660820
H	4.261053	2.361562	2.950352
H	7.005592	-2.429399	-0.759294
H	6.532555	-4.149294	-0.686012

H	6.620089	-3.189774	0.817421
F	-3.535515	-2.336210	-1.423156
F	-5.769927	-1.199574	-0.424871
F	-5.678582	0.118621	1.940696
F	-3.346413	0.305643	3.317212
F	-1.111095	-0.816531	2.337515
H	2.851307	1.666426	-1.957436
H	3.582978	3.978274	-2.518799
H	2.440031	5.911396	-1.394477
H	0.579694	5.422634	0.219839
H	-0.060447	3.044784	0.628296
H	2.887617	-3.441895	-1.039832

- **Complex: 3f**

58

	X	Y	Z
C	-2.445655	-0.885312	1.242591
C	-2.389700	-1.850062	0.231769
C	-3.562528	-2.385813	-0.315374
C	-4.798209	-1.926075	0.119279
C	-4.824689	-0.944539	1.103189
C	-3.676547	-0.416810	1.682071
C	-1.093604	-2.334196	-0.239555
N	0.062498	-1.751481	-0.190267
C	0.996345	-2.676228	-0.681650
C	0.266074	-3.865500	-1.099117
O	-1.065265	-3.581649	-0.766928
C	2.347387	-2.561781	-0.629003
C	3.075434	-1.478847	-0.036422
C	2.480183	-0.254371	0.353746
C	3.222827	0.665124	1.095031
C	4.562637	0.402247	1.407124
C	5.193285	-0.773779	0.975177
C	4.457317	-1.702983	0.260599
Pd	0.680969	0.267663	-0.240167
O	-1.272763	0.853853	-1.017632
C	-2.059855	1.575113	-0.341482
O	-1.895851	2.120065	0.749312
O	5.345456	1.246149	2.108250
C	4.788858	2.479239	2.549350
O	4.972750	-2.870419	-0.178370
C	6.336985	-3.156012	0.102925
O	0.582237	-4.912785	-1.594395
N	1.409373	2.198328	-0.646708
C	0.896257	3.283960	-0.045332
C	1.374317	4.564188	-0.304825
C	2.405576	4.726532	-1.224620
C	2.926784	3.599148	-1.855185
C	2.406561	2.352622	-1.534574
C	-3.433361	1.772901	-1.039324
F	-3.891169	0.626978	-1.569430
F	-4.372365	2.223516	-0.198412
F	-3.330737	2.664638	-2.043536
H	2.771401	1.596922	1.408426
H	6.236005	-0.920422	1.223831

H	4.458470	3.083363	1.697758
H	5.590252	2.993920	3.075938
H	3.950431	2.306627	3.230739
H	6.991878	-2.405724	-0.350525
H	6.532475	-4.131006	-0.339228
H	6.508636	-3.197778	1.182827
H	-3.505103	-3.143798	-1.088081
H	-5.721604	-2.307135	-0.297901
N	-6.131224	-0.434624	1.551046
H	-3.746929	0.347629	2.445462
H	-1.532299	-0.493189	1.677880
H	2.795239	1.446223	-1.986036
H	3.727546	3.674090	-2.581235
H	2.796554	5.713605	-1.447904
H	0.934591	5.410786	0.208995
H	0.080718	3.104114	0.647263
H	2.909820	-3.419136	-0.987721
O	-6.141170	0.429662	2.410288
O	-7.132250	-0.903119	1.036068

- **Complex: 3g**

55

	X	Y	Z
Pd	-1.152913	0.049128	-0.130181
F	3.320061	1.822317	0.653589
F	2.756257	2.522809	2.619600
F	2.034998	3.552922	0.861357
O	1.474487	-3.166459	0.805392
O	0.249363	-4.779942	1.877846
O	0.654662	1.249957	0.123116
O	0.621718	0.961475	2.365962
N	-0.108359	-1.742842	0.140616
N	-2.168903	1.889601	-0.104820
C	-2.822910	-0.948288	-0.425304
C	-3.832218	-0.379016	-1.206883
H	-3.653922	0.570909	-1.705155
C	-5.092982	-0.971066	-1.373492
C	-5.361204	-2.174951	-0.714846
H	-6.338398	-2.639590	-0.808294
C	-4.371248	-2.782785	0.042710
H	-4.570190	-3.734380	0.529187
C	-3.092033	-2.206879	0.172676
C	-6.127776	-0.306982	-2.243137
H	-6.239962	0.749023	-1.980134
H	-7.100216	-0.793962	-2.144932
H	-5.830464	-0.351056	-3.296021
C	-2.087380	-3.013324	0.835614
C	-0.756415	-2.803292	0.781950
C	0.279098	-3.729153	1.255802
C	1.168099	-2.003401	0.167275
C	2.234325	-1.238291	-0.419603
C	3.520427	-1.629639	-0.309952
C	4.678355	-0.947160	-0.884621
C	5.954226	-1.477221	-0.638639
H	6.049362	-2.373596	-0.031789

C	7.091603	-0.865883	-1.159880
H	8.071767	-1.285921	-0.959527
C	6.966340	0.284215	-1.937658
H	7.850128	0.764121	-2.345745
C	5.700082	0.819662	-2.192604
H	5.601261	1.715068	-2.797572
C	4.565081	0.210843	-1.672771
H	3.589457	0.641322	-1.874778
C	-1.653864	2.917967	-0.797891
H	-0.763873	2.704333	-1.379200
C	-2.215599	4.188471	-0.764385
H	-1.771043	4.985370	-1.348507
C	-3.337901	4.405958	0.030003
H	-3.795778	5.388142	0.081081
C	-3.859840	3.342616	0.761876
H	-4.725787	3.465035	1.401477
C	-3.250157	2.098310	0.664052
H	-3.628544	1.238343	1.205287
C	1.059906	1.415300	1.313413
C	2.307013	2.337778	1.374340
H	-2.411846	-3.921596	1.338725
H	1.923190	-0.339915	-0.938938
H	3.737867	-2.534252	0.254888

- **Complex: 3h**

60

	X	Y	Z
C	-2.630061	2.547363	1.049027
N	-1.566509	2.324551	0.259709
C	-0.943465	3.370826	-0.305631
C	-1.377957	4.678112	-0.122915
C	-2.483038	4.910755	0.691097
C	-3.114817	3.825928	1.293694
Pd	-0.753130	0.406114	-0.025688
C	-2.524475	-0.363412	-0.403779
C	-3.448485	0.413223	-1.108754
C	-4.765649	-0.029948	-1.272426
C	-5.194564	-1.247896	-0.726577
C	-4.279073	-2.037390	-0.051103
C	-2.916931	-1.629432	0.094527
O	-5.715949	0.667083	-1.932301
C	-2.001681	-2.587063	0.656596
C	-0.651725	-2.520478	0.569760
N	0.084978	-1.500812	-0.039637
C	1.324849	-1.892799	-0.073840
O	1.523714	-3.113647	0.488925
C	0.287857	-3.570701	0.951924
O	0.173514	-4.630830	1.505172
C	2.448720	-1.205547	-0.654418
C	3.705308	-1.675362	-0.517831
C	4.916856	-1.074396	-1.073918
C	6.158542	-1.607593	-0.696535
C	7.344006	-1.067584	-1.188665
C	7.301468	0.009501	-2.072717
C	6.069622	0.543365	-2.462933

C	4.886921	0.007587	-1.969514
O	1.181805	1.329631	0.364126
C	1.820370	0.952844	1.391564
C	3.252887	1.548202	1.476589
F	3.235623	2.698641	2.181100
O	1.472676	0.220315	2.313777
F	3.770882	1.829616	0.272492
F	4.104439	0.718250	2.093210
H	-3.150013	1.372524	-1.510692
H	-6.228324	-1.537462	-0.861616
O	-4.597801	-3.234908	0.487943
H	6.188764	-2.447440	-0.007789
H	8.297091	-1.487321	-0.884125
H	8.222922	0.431841	-2.460644
H	6.034893	1.378718	-3.154715
H	3.938032	0.431457	-2.282641
H	-0.071365	3.140325	-0.907481
H	-0.849857	5.490076	-0.608376
H	-2.843076	5.920606	0.857079
H	-3.971530	3.957921	1.943781
H	-3.098440	1.671402	1.483845
H	-2.412162	-3.485821	1.106725
H	2.206657	-0.289723	-1.181762
H	3.852588	-2.580583	0.068661
C	-5.936024	-3.696876	0.365330
C	-5.367098	1.931378	-2.481232
H	-6.209199	-3.819447	-0.687323
H	-5.963567	-4.662164	0.867360
H	-6.631807	-3.006892	0.852840
H	-6.273016	2.311684	-2.949681
H	-5.041017	2.620627	-1.695106
H	-4.579223	1.826869	-3.233462

- **Complex: 3i**

63

	X	Y	Z
C	8.721794	-1.551657	0.488344
C	8.554342	-0.387123	1.244690
C	7.293495	0.193307	1.388951
C	6.205475	-0.412809	0.762585
C	6.372267	-1.583634	0.003714
C	7.629064	-2.158460	-0.138171
C	5.043734	-2.036552	-0.558005
C	4.090983	-0.972677	-0.053017
C	4.783624	-0.040342	0.728488
C	2.702636	-0.866254	-0.252647
C	2.007212	0.242855	0.302603
C	2.724791	1.141118	1.106470
C	4.096439	1.009715	1.332936
Pd	0.123833	0.621336	-0.120282
O	-1.903908	1.086485	-0.826321
C	-2.847257	1.432440	-0.064547
O	-2.872086	1.531645	1.164086
C	2.046261	-1.969990	-0.932755
C	0.721890	-2.218846	-0.892846

N	-0.242296	-1.426698	-0.266948
C	-1.356553	-2.099452	-0.274781
O	-1.261575	-3.305604	-0.898648
C	0.049162	-3.440691	-1.353402
C	-2.603304	-1.713328	0.321977
C	-3.724507	-2.444715	0.156635
C	-5.038852	-2.133098	0.713810
O	0.417217	-4.408254	-1.956728
N	0.505210	2.684218	-0.205122
C	-0.284913	3.520673	0.487817
C	-0.134273	4.900860	0.423715
C	0.857888	5.432176	-0.395445
C	1.668225	4.562544	-1.120662
C	1.464012	3.194352	-0.994572
C	-4.158235	1.723598	-0.843689
F	-4.599007	0.608091	-1.453951
F	-5.143000	2.161759	-0.051948
F	-3.966986	2.651213	-1.797653
H	4.612993	1.727263	1.964660
H	2.207677	1.977669	1.565641
H	2.080353	2.478414	-1.527113
H	2.450841	4.928852	-1.774309
H	0.997059	6.505605	-0.468285
H	-0.789133	5.537063	1.007175
H	-1.051864	3.058877	1.102476
H	7.763868	-3.063458	-0.723910
H	9.710313	-1.988444	0.387267
H	9.414691	0.069484	1.723856
H	7.168143	1.098315	1.976485
H	4.774542	-3.034164	-0.190742
H	5.061248	-2.089950	-1.653274
H	2.662516	-2.717289	-1.426107
C	-6.091428	-3.031351	0.480573
C	-7.364350	-2.781419	0.986520
C	-7.600535	-1.625707	1.729804
C	-6.560207	-0.721522	1.964267
C	-5.288506	-0.969734	1.463077
H	-5.904903	-3.929391	-0.102031
H	-8.169056	-3.484745	0.799324
H	-8.592030	-1.425601	2.123382
H	-6.744140	0.182092	2.536412
H	-4.495568	-0.249510	1.646454
H	-3.666427	-3.348636	-0.447354
H	-2.566325	-0.805926	0.915694

- **Complex: 3j**

60

	X	Y	Z
C	5.005771	-0.686094	-1.642222
C	4.837248	-1.828296	-0.840896
C	5.946610	-2.650609	-0.590733
C	7.195682	-2.342076	-1.123114
C	7.350440	-1.205550	-1.915381
C	6.252081	-0.379471	-2.173122
C	3.550674	-2.202765	-0.257318

C	2.394875	-1.518220	-0.381082
C	1.179169	-1.993478	0.219646
N	0.000659	-1.439428	0.179520
C	-0.877259	-2.295479	0.850089
C	-0.086234	-3.423847	1.355703
O	1.203299	-3.176875	0.892638
C	-2.219868	-2.191698	0.913254
C	-3.010453	-1.193351	0.218213
C	-2.454230	-0.048977	-0.413784
C	-3.306487	0.712474	-1.212597
C	-4.667026	0.415476	-1.359405
C	-5.225504	-0.664426	-0.680773
C	-4.389009	-1.470615	0.106244
Pd	-0.592503	0.537999	-0.148105
O	1.446635	1.285163	0.057175
C	1.850632	1.528461	1.234398
O	1.286537	1.368114	2.311549
H	-5.280134	1.047828	-1.991318
O	-4.924055	-2.532164	0.780936
C	-5.223866	-3.660732	-0.046607
O	-0.355864	-4.399687	1.997452
N	-1.151195	2.564491	-0.174774
C	-2.138414	3.038360	0.602795
C	-2.434053	4.393514	0.679854
C	-1.688693	5.288345	-0.083094
C	-0.665068	4.795529	-0.887771
C	-0.420853	3.427752	-0.899019
C	3.288586	2.113132	1.238228
F	3.735604	2.385637	2.468059
F	3.347733	3.255633	0.527756
F	4.157181	1.252801	0.675225
H	-2.925193	1.580565	-1.740654
O	-6.535768	-1.018864	-0.717406
H	5.823373	-3.535867	0.027184
H	8.044712	-2.986442	-0.920117
H	8.322220	-0.961305	-2.332410
H	6.371674	0.506070	-2.788736
H	4.163864	-0.032324	-1.846553
H	0.382184	2.998511	-1.487592
H	-0.055343	5.452631	-1.496371
H	-1.899616	6.351939	-0.048168
H	-3.234397	4.730224	1.327890
H	-2.699152	2.302859	1.168705
H	-2.746986	-2.975183	1.449675
H	2.305743	-0.582430	-0.919154
H	3.546344	-3.121508	0.326292
H	-5.666416	-4.412364	0.606436
H	-5.931849	-3.384448	-0.832085
H	-4.302581	-4.053501	-0.491824
C	-7.400404	-0.234472	-1.524257
H	-8.389676	-0.677092	-1.421005
H	-7.425308	0.804063	-1.177495
H	-7.091594	-0.265089	-2.574712

- **Complex: 3k**

52

	X	Y	Z
C	-1.491188	3.115421	-0.641615
N	-2.041512	2.056419	-0.027895
C	-3.173417	2.222027	0.673995
C	-3.802849	3.455675	0.779866
C	-3.243705	4.553196	0.131253
C	-2.066583	4.378727	-0.591122
Pd	-1.051796	0.222875	-0.129854
O	0.757338	1.333673	0.258209
C	1.348560	1.154623	1.367529
C	2.616632	2.041225	1.491114
F	3.528188	1.691780	0.563516
N	-0.095989	-1.613994	0.099467
C	1.169225	-1.917876	0.150165
O	1.434614	-3.039005	0.873114
C	0.224722	-3.514687	1.370174
C	-0.783340	-2.591013	0.824340
C	2.256206	-1.227929	-0.484696
C	3.531555	-1.640552	-0.327538
C	4.715070	-1.038682	-0.935080
C	4.645454	0.041714	-1.831300
C	5.805423	0.573840	-2.378687
C	7.052744	0.038474	-2.042384
C	7.134642	-1.034648	-1.156394
C	5.972147	-1.570208	-0.608428
C	-2.118734	-2.739855	0.911226
C	-3.090234	-1.944264	0.169387
C	-2.737987	-0.741685	-0.489818
C	-3.707044	-0.226099	-1.345505
C	-4.980430	-0.755180	-1.517969
C	-5.290698	-1.891371	-0.788627
C	-4.375920	-2.504299	0.043828
O	0.156767	-4.489931	2.060624
H	-5.694725	-0.297254	-2.192315
H	-4.644141	-3.418264	0.563122
O	1.076793	0.396810	2.292510
F	3.203809	1.938515	2.687010
F	2.329102	3.340164	1.294278
F	-3.420970	0.873018	-2.083098
F	-6.522582	-2.424853	-0.918628
H	6.032330	-2.406533	0.082636
H	8.100157	-1.453628	-0.893083
H	7.956269	0.458682	-2.472443
H	5.741483	1.408578	-3.068917
H	3.684836	0.469757	-2.099565
H	-0.564256	2.932579	-1.173358
H	-1.591081	5.203295	-1.108610
H	-3.715305	5.528331	0.190753
H	-4.713031	3.542971	1.361077
H	-3.577205	1.338061	1.155479
H	-2.481380	-3.585351	1.490136
H	1.971886	-0.367394	-1.079446
H	3.715761	-2.502507	0.311175

- **Complex: 3m**

60

	X	Y	Z
C	5.058973	-0.252444	-1.631457
C	5.035639	-1.465684	-0.922562
C	6.233701	-2.178018	-0.759868
C	7.427788	-1.693279	-1.287328
C	7.438359	-0.487687	-1.987146
C	6.250690	0.230277	-2.157237
C	3.812015	-2.023749	-0.348503
C	2.587884	-1.459593	-0.393837
C	1.444780	-2.107863	0.189284
N	0.211323	-1.688031	0.211175
C	-0.547692	-2.683452	0.832755
C	0.368895	-3.748677	1.234751
O	1.613666	-3.325847	0.772452
C	-1.896143	-2.727497	0.924312
C	-2.812871	-1.778764	0.340634
C	-2.409609	-0.557105	-0.237070
C	-3.378495	0.173068	-0.944607
C	-4.704594	-0.245096	-1.038096
C	-5.115915	-1.440643	-0.396217
C	-4.171125	-2.188566	0.274295
Pd	-0.611334	0.219622	-0.019207
O	1.328957	1.198125	0.148754
C	1.680962	1.580626	1.305639
O	1.124829	1.434433	2.388713
O	-5.672160	0.422213	-1.698642
C	-5.323937	1.643080	-2.336711
H	-4.450938	-3.123336	0.747529
O	0.225995	-4.795298	1.805857
N	-1.386625	2.171545	0.037451
C	-2.353260	2.522525	0.901201
C	-2.800113	3.832509	1.018275
C	-2.232535	4.810870	0.206033
C	-1.228063	4.445439	-0.686052
C	-0.825688	3.116162	-0.734056
C	3.028202	2.350860	1.268821
F	3.446008	2.724962	2.482019
F	2.922180	3.466151	0.520079
F	4.000197	1.598740	0.721815
H	-3.096282	1.098156	-1.430768
O	-6.430454	-1.753208	-0.514343
H	6.223259	-3.117291	-0.213677
H	8.346377	-2.254810	-1.152375
H	8.366764	-0.105985	-2.399801
H	6.257445	1.169366	-2.700942
H	4.146151	0.318625	-1.767967
H	-0.030500	2.785235	-1.392840
H	-0.753281	5.171914	-1.334656
H	-2.564743	5.841612	0.270374
H	-3.577757	4.070574	1.734168
H	-2.773357	1.723669	1.502324
H	-2.311117	-3.611350	1.404282
H	2.387389	-0.498135	-0.850369
H	3.921291	-2.982035	0.156054

H	-6.241553	2.012737	-2.790659
H	-4.952735	2.372075	-1.608065
H	-4.569295	1.477668	-3.112209
C	-6.870883	-2.943503	0.118657
H	-7.939518	-3.010536	-0.078239
H	-6.364876	-3.820489	-0.299635
H	-6.698175	-2.899677	1.199615

- **Complex: 4d**

60

	X	Y	Z
C	-2.753913	-0.495811	2.094410
C	-3.040351	-1.310174	0.991137
C	-4.360187	-1.499948	0.567101
C	-5.392621	-0.851321	1.238578
C	-5.111127	-0.040969	2.339735
C	-3.794475	0.131241	2.771023
C	-1.943156	-1.977622	0.298446
N	-0.734862	-1.541436	0.125658
C	-0.008719	-2.570754	-0.487058
C	-0.937995	-3.663521	-0.748038
O	-2.154000	-3.218491	-0.197986
C	1.337719	-2.591646	-0.645166
C	2.244571	-1.577325	-0.181244
C	1.841338	-0.299630	0.283744
C	2.777129	0.541123	0.885500
C	4.117635	0.146509	0.990692
C	4.553431	-1.084311	0.481501
C	3.627926	-1.934565	-0.099745
Pd	0.021016	0.388630	-0.070952
N	-1.985993	1.083879	-0.749263
O	5.078738	0.906642	1.551505
C	4.722854	2.187499	2.058009
O	3.954885	-3.144331	-0.599740
C	5.312788	-3.562236	-0.532441
O	-0.841703	-4.741270	-1.266377
N	0.808884	2.289766	-0.474096
C	0.512064	3.331677	0.318382
C	1.014376	4.606090	0.087389
C	1.848372	4.808674	-1.009196
C	2.150657	3.728036	-1.833480
C	1.614994	2.482589	-1.530475
H	2.475458	1.512503	1.253993
H	5.602425	-1.334864	0.567624
H	4.329041	2.825495	1.259685
H	5.642723	2.616490	2.450591
H	3.985361	2.097791	2.861287
H	5.957254	-2.878427	-1.093144
H	5.340626	-4.550526	-0.987113
H	5.647747	-3.623413	0.507348
H	-4.568128	-2.137914	-0.286118
H	-6.416682	-0.981727	0.905489
H	-5.920856	0.453392	2.866639
H	-3.580162	0.751345	3.635012
H	-1.724889	-0.375142	2.422421

H	1.837072	1.608615	-2.133039
H	2.795381	3.837134	-2.697301
H	2.257365	5.791769	-1.216270
H	0.753259	5.415000	0.759120
H	-0.133369	3.127568	1.166699
H	1.759950	-3.496378	-1.072299
C	-2.821812	1.864237	-0.051594
C	-4.112942	2.150894	-0.479933
C	-4.552875	1.610671	-1.685257
C	-3.682603	0.809894	-2.420420
C	-2.409990	0.571930	-1.915267
H	-2.450590	2.253252	0.891373
H	-4.755059	2.776112	0.129500
H	-5.557302	1.808546	-2.044529
H	-3.978271	0.368987	-3.365068
H	-1.703187	-0.055811	-2.451437

- **Complex: 4h**

64

	X	Y	Z
C	-5.085319	-0.226938	-1.690172
C	-5.124091	0.877140	-0.821505
C	-6.367388	1.326520	-0.352097
C	-7.543970	0.681593	-0.725737
C	-7.492003	-0.417330	-1.581800
C	-6.259550	-0.866907	-2.065889
C	-3.918438	1.572864	-0.372935
C	-2.650241	1.170355	-0.586319
C	-1.521798	1.903817	-0.077865
N	-0.282828	1.505838	-0.019840
C	0.466562	2.574852	0.483603
C	-0.463403	3.655900	0.792012
O	-1.710579	3.164194	0.385554
C	1.818934	2.649848	0.529901
C	2.727825	1.655357	0.030526
C	2.337350	0.355036	-0.374702
C	3.260321	-0.467321	-1.021649
C	4.576649	-0.033503	-1.224183
C	5.003608	1.223175	-0.774820
C	4.088741	2.056915	-0.154308
Pd	0.569249	-0.398710	0.106582
N	-1.360286	-1.238295	0.835752
O	5.523829	-0.779176	-1.828269
C	5.176740	-2.084614	-2.273865
O	4.405167	3.290298	0.293325
C	5.736431	3.756326	0.112210
O	-0.341862	4.755533	1.257566
N	1.460840	-2.267669	0.457108
C	2.277359	-2.437938	1.508929
C	2.887027	-3.656235	1.782216
C	2.647676	-4.733640	0.933649
C	1.801293	-4.554982	-0.157661
C	1.225733	-3.307072	-0.359271
H	2.971009	-1.455086	-1.352933
H	6.035989	1.504798	-0.934767

H	-6.406407	2.184580	0.313314
H	-8.498060	1.038380	-0.352147
H	-8.406961	-0.919960	-1.878315
H	-6.218430	-1.715749	-2.740753
H	-4.137326	-0.581769	-2.083557
H	0.573663	-3.120109	-1.206429
H	1.586811	-5.361732	-0.848275
H	3.114306	-5.695410	1.117656
H	3.539142	-3.746436	2.642667
H	2.448089	-1.567684	2.133236
H	2.235089	3.584979	0.892390
H	-2.400967	0.266686	-1.129208
H	-4.078785	2.481398	0.205195
H	5.989717	3.798125	-0.951464
H	5.759340	4.758278	0.536320
H	6.447890	3.114560	0.640713
H	6.084317	-2.500959	-2.706682
H	4.847577	-2.707283	-1.434975
H	4.391587	-2.040739	-3.034805
C	-1.822445	-0.792100	2.012818
C	-3.086222	-1.118366	2.492382
C	-3.901541	-1.942961	1.721949
C	-3.416496	-2.423708	0.507809
C	-2.141181	-2.047387	0.104578
H	-1.159400	-0.140277	2.575412
H	-3.418341	-0.723870	3.445397
H	-4.898470	-2.205451	2.060093
H	-4.015172	-3.066963	-0.126617
H	-1.737168	-2.379764	-0.847621

- **Complex: 5h**

54

	X	Y	Z
C	-5.412193	1.096985	0.625023
C	-5.495100	-0.301251	0.510389
C	-6.727072	-0.930147	0.743965
C	-7.845828	-0.186259	1.110597
C	-7.749318	1.199315	1.234897
C	-6.531287	1.838182	0.985938
C	-4.340345	-1.122464	0.143617
C	-3.062567	-0.696141	0.144022
C	-1.974657	-1.548396	-0.250027
N	-0.706209	-1.256647	-0.277563
C	-0.028561	-2.421334	-0.651845
C	-1.031638	-3.446439	-0.922462
O	-2.245141	-2.830750	-0.614566
C	1.307572	-2.631503	-0.577933
C	2.265202	-1.708670	-0.032943
C	1.959155	-0.369766	0.311884
C	2.881413	0.369886	1.057461
C	4.120095	-0.181422	1.402572
C	4.474777	-1.477620	1.003674
C	3.555194	-2.231960	0.295225
Pd	0.339729	0.551939	-0.361883
Cl	-1.590247	1.842786	-1.334347

O	5.063646	0.479230	2.107932
C	4.793174	1.816061	2.509923
O	3.799757	-3.498171	-0.109466
C	5.054554	-4.081028	0.214658
O	-0.977948	-4.588649	-1.292515
N	1.466586	2.302435	-0.632984
C	2.558007	2.311522	-1.414144
C	3.307506	3.461919	-1.625935
C	2.921561	4.640321	-0.992559
C	1.789605	4.628185	-0.182146
C	1.081648	3.441052	-0.036545
H	2.649650	1.384552	1.351547
H	5.453606	-1.851250	1.273699
H	-6.800947	-2.009565	0.642832
H	-8.791122	-0.686244	1.294878
H	-8.620634	1.782398	1.515552
H	-6.458396	2.917999	1.065470
H	-4.477202	1.606055	0.408262
H	0.180221	3.386299	0.563252
H	1.447865	5.520811	0.328120
H	3.491547	5.553065	-1.130470
H	4.176695	3.423188	-2.271712
H	2.832929	1.365650	-1.868076
H	1.651947	-3.624465	-0.850373
H	-2.772873	0.307547	0.428418
H	-4.550921	-2.151531	-0.141750
H	5.190781	-4.130577	1.299452
H	5.029823	-5.087250	-0.199511
H	5.874785	-3.515506	-0.238264
H	5.681615	2.151436	3.041877
H	4.619928	2.457300	1.638862
H	3.926845	1.855571	3.177355

- **Complex: 6h**

96

	X	Y	Z
O	0.300752	5.189295	-0.678614
C	-0.062285	4.050198	-0.807018
O	-1.415055	3.726565	-0.968125
C	-1.495590	2.372251	-1.064607
C	-2.777442	1.726128	-1.163222
C	-3.943383	2.393290	-1.077175
C	-5.265693	1.764706	-1.087827
C	-5.444085	0.391888	-0.842165
C	-6.719467	-0.160987	-0.866145
C	-7.832602	0.640429	-1.134506
C	-7.666759	2.005445	-1.366754
C	-6.391745	2.564263	-1.333977
N	-0.344868	1.776419	-1.018920
C	0.627826	2.764222	-0.866021
C	1.969637	2.555392	-0.872982
C	2.610776	1.296603	-1.149381
C	3.971282	1.352824	-1.557384
O	4.596583	2.539809	-1.386917
C	5.982488	2.629881	-1.682583

C	4.608950	0.253210	-2.132229
C	3.885869	-0.934682	-2.253202
O	4.391655	-2.057813	-2.809487
C	5.717432	-2.023791	-3.317889
C	2.584691	-1.049177	-1.738778
C	1.940524	0.034611	-1.163296
Pd	0.199037	-0.125576	-0.243805
C	0.804485	-1.814677	0.673415
N	0.117755	-2.979427	0.748146
C	-1.192646	-3.200551	0.191811
C	-2.285238	-3.160527	1.064836
C	-2.104169	-2.890061	2.534427
C	-3.557009	-3.354085	0.522698
C	-3.748049	-3.572599	-0.842754
C	-5.129015	-3.759443	-1.418511
C	-2.628008	-3.607010	-1.677111
C	-1.335860	-3.423548	-1.181609
C	-0.153981	-3.442954	-2.113630
C	0.778401	-3.896524	1.552377
C	1.902727	-3.284961	1.995584
N	1.901626	-2.011080	1.448180
C	2.888491	-0.995838	1.702016
C	4.170417	-1.133999	1.163336
C	4.615854	-2.396432	0.472030
C	5.052540	-0.059750	1.314145
C	4.684940	1.108263	1.981698
C	5.617790	2.290228	2.045204
C	3.422039	1.164620	2.580682
C	2.510423	0.115527	2.468861
C	1.191283	0.148483	3.197909
O	-1.694036	-0.216883	0.872207
C	-2.475204	0.419220	1.628193
C	-1.956761	1.756938	2.246996
F	-2.834440	2.755008	2.052534
F	-0.778019	2.178650	1.753974
F	-1.796536	1.623158	3.577847
O	-3.615795	0.126790	1.984285
H	-2.726209	0.646917	-1.265263
H	-3.920882	3.478380	-0.989434
H	-4.592431	-0.235334	-0.589999
H	-6.850241	-1.218410	-0.659402
H	-8.825724	0.202717	-1.149197
H	-8.528429	2.634366	-1.565636
H	-6.260088	3.629051	-1.506587
H	2.596158	3.437704	-0.781666
H	6.169004	2.467632	-2.748778
H	6.278402	3.641873	-1.410612
H	6.552328	1.906602	-1.088516
H	5.632043	0.322354	-2.471656
H	6.437715	-1.811918	-2.519869
H	5.907277	-3.015055	-3.725606
H	5.812938	-1.274740	-4.110351
H	2.129421	-2.031719	-1.773107
H	-1.722755	-3.771902	3.060593
H	-3.056871	-2.605908	2.983978

H	-1.393367	-2.072449	2.690638
H	-4.419371	-3.308520	1.183475
H	-5.884225	-3.798651	-0.630166
H	-5.381575	-2.935082	-2.093665
H	-5.190011	-4.685396	-1.997929
H	-2.760512	-3.783725	-2.742550
H	-0.383758	-4.023135	-3.009783
H	0.099544	-2.424320	-2.428073
H	0.730245	-3.875772	-1.636685
H	0.384394	-4.884926	1.729083
H	2.693671	-3.626626	2.644373
H	3.812284	-2.851556	-0.111745
H	4.957421	-3.135033	1.206053
H	5.450961	-2.186679	-0.201038
H	6.045697	-0.136136	0.875525
H	5.551357	2.798427	3.010705
H	6.655153	1.986854	1.882706
H	5.354340	3.017486	1.268768
H	3.139978	2.046098	3.152142
H	1.111186	-0.705640	3.879784
H	1.100199	1.066200	3.781552
H	0.342300	0.095527	2.512391

10.2. Absorption Properties

In this section the calculated vertical transition wave lengths are given, calculated with wB97XD (subsection 10.2.1.) and M06-2x (subsection 10.2.2.) functionals. While T_1 and S_1 transitions may be understood as one electron HOMO-LUMO transitions, the rest of excitations have multiple electron character.

10.2.1. wB97XD

Table S4. *Calculated vertical transitions for the lowest T_1, T_2, T_3, S_1 and S_2 excited states with the wB97XD functional. Calculated and experimental wave lengths in nm, and oscillator strengths for S_1 and S_2 states in parenthesis.*

Compound	T_1	T_2	T_3	S_1	S_2	Exp
**3a**	667	464	428	396 (0.2862)	354 (0.0711)	448, 468
**3b**	753	462	439	424 (0.5115)	353 (0.0154)	460, 485
**3c**	831	528	461	446 (1.1219)	375 (0.114)	482, 516
**3d**	687	462	395	406 (0.699)	351 (0.051)	450, 471
**3e**	691	466	390	410 (0.6525)	353 (0.0669)	457
**3f**	700	464	423	419 (0.721)	354 (0.047)	474
**3g**	749	482	459	405 (1.010)	350 (0.104)	445, 466
**3h**	775	488	462	429 (1.077)	351 (0.080)	475, 501
**3i**	749	490	459	412 (0.7206)	361 (0.7853)	474, 500
**3j**	743	482	459	406 (0.7443)	367 (0.6154)	424, 465
**3k**	737	480	472	396 (0.9214)	357 (0.0880)	445
**3m**	792	500	461	437 (0.8377)	351 (0.1566)	486
**4d**	685	430	383	407 (0.6666)	335 (0.0636)	471
**4h**	752	484	434	429 (0.972)	337 (0.099)	488, 510
**5h**	770	488	467	429 (0.9998)	355 (0.0858)	481, 507
**6h**	772	467	413	429 (0.903)	342 (0.009)	483, 510

10.2.2. M06-2X

***Table S5**. Calculated vertical transitions for the lowest T_1, T_2, T_3, S_1 and S_2 excited states with the M06-2X functional. Calculated and experimental wave lengths in nm, and oscillator strengths for S_1 and S_2 states in parenthesis.*

Compound	T_1	T_2	T_3	S_1	S_2	Exp
**3a**	583	553	453	405 (0.052)	390 (0.240)	448, 468
**3b**	646	549	451	424 (0.466)	398 (0.101)	460, 485
**3c**	693	551	472	453 (1.075)	402 (0.054)	482, 516
**3d**	628	551	450	413 (0.470)	396 (0.277)	450, 471
**3e**	642	550	441	417 (0.465)	396 (0.232)	457
**3f**	647	558	452	429 (0.650)	403 (0.101)	474
**3g**	641	544	447	407 (0.667)	394 (0.478)	445, 466
**3h**	675	541	450	431 (0.9653)	397 (0.1110)	475, 501
**3i**	635	538	447	412 (0.590)	394 (0.215)	474, 500
**3j**	630	548	451	406 (0.361)	395 (0.673)	424, 465
**3k**	618	557	465	409 (0.038)	390 (0.984)	445
**3m**	683	548	456	438 (0.8159)	399 (0.1064)	486
**4d**	630	514	421	411 (0.6024)	381 (0.085)	471
**4h**	676	520	444	434 (0.964)	385 (0.073)	488, 510
**5h**	676	559	458	432 (0.891)	405 (0.173)	481, 507
**6h**	692	452	442	438 (0.945)	356 (0.066)	483, 510

10.3.- Emission from S_1

In this section emission properties are given, calculated from the optimized geometries of the S_1 excited states. In subsection 10.3.1. calculated properties and S_1 optimized geometries are given calculated with the wB97XD functional, and in subsection 10.3.2 those obtained with the M06-2X functional. As for absorption properties, T_1 and S_1 transitions are monoelectronic HOMO-LUMO transitions, while the rest have multielectronic character.

10.3.1. wB97XD

Table S6. *Calculated emission properties from the S_1 optimized geometry for the lowest T_1, T_2, T_3, S_1 and S_2 excited states with the wB97XD functional. Calculated and experimental wave lengths in nm, and oscillator strengths for S_1 and S_2 states in parenthesis.*

Compound	T_1	T_2	T_3	S_1	S_2	Exp
**3a**	1102	492	434	495 (0.583)	393 (0.420)	521, 542
**3b**	1393	493	441	548 (0.730)	390 (0.035)	525, 550
**3c**	1675	598	462	588 (1.477)	415 (0.163)	577
**3d**	1046	447	445	510 (0.894)	354 (0.032)	539
**3e**	971	459	433	502 (0.829)	372 (0.025)	534, 564
**3f**	1185	500	451	560 (0.959)	382 (0.027)	630
**3g**	1669	558	440	536 (1.287)	374 (0.015)	534
**3h**	1579	557	448	559 (1.337)	357 (0.066)	539, 570
**3i**	1602	563	466	536 (1.158)	400 (0.561)	544, 574
**3j**	1627	558	439	529 (1.221)	408 (0.396)	533
**3k**	1185	500	451	560 (0.959)	382 (0.027)	485, 510, 550
**3m**	1581	573	442	562 (1.1365)	373 (0.5775)	605
**4d**	1025	447	422	513 (0.877)	345 (0.115)	526, 553
**4h**	1510	558	424	563 (1.288)	353 (0.311)	558, 590
**5h**	1600	561	454	563 (1.244)	362 (0.044)	542, 593
**6h**	1556	549	410	558 (1.224)	366 (0.022)	542, 593

Cartesian Coordinates

- **Complex: 3a**

59

	X	Y	Z
C	-0.636008	3.289262	0.689755
N	-0.121922	2.409800	-0.186401
C	0.625367	2.864307	-1.208365
C	0.880692	4.214188	-1.392191
C	0.355521	5.127968	-0.482447
C	-0.413969	4.654626	0.575107

Pd	-0.436671	0.374885	-0.006194
C	1.452463	0.142974	0.437647
C	2.109682	1.055805	1.283255
C	3.490235	1.091343	1.400098
C	4.264278	0.188832	0.634416
C	3.660732	-0.762541	-0.174054
C	2.246913	-0.873360	-0.250980
H	3.967905	1.818258	2.050230
C	4.702742	-1.632599	-0.829500
C	1.663493	-1.970515	-0.908003
C	0.318322	-2.351977	-0.737252
N	-0.645411	-1.652306	-0.071655
C	-1.725131	-2.434654	0.027056
O	-1.534456	-3.613801	-0.599919
C	-0.245292	-3.627869	-1.120917
O	0.190580	-4.582684	-1.733312
C	-2.951123	-2.231127	0.742210
C	-4.049209	-3.082809	0.499814
C	-5.230998	-2.907354	1.203361
C	-5.337587	-1.895126	2.161384
C	-4.248068	-1.060455	2.418325
C	-3.061990	-1.223660	1.718873
O	-2.428373	0.605368	-0.711374
C	-3.346700	1.262107	-0.134029
C	-4.668705	1.244832	-0.956659
F	-5.697946	1.782723	-0.287833
O	-3.332481	1.881588	0.925863
F	-4.524953	1.947955	-2.101612
F	-5.025766	-0.006389	-1.299937
H	1.524446	1.784652	1.834154
C	5.716002	0.034342	0.550188
H	-3.969421	-3.864081	-0.247766
H	-6.076202	-3.557473	1.002853
H	-6.266584	-1.757965	2.705409
H	-4.324989	-0.275420	3.162781
H	-2.217882	-0.574984	1.926816
H	1.026745	2.116957	-1.882625
H	1.485131	4.533712	-2.232547
H	0.543171	6.190133	-0.596967
H	-0.846734	5.326783	1.306300
H	-1.252581	2.874770	1.476952
C	5.991529	-1.036344	-0.319939
C	6.747785	0.746155	1.163146
C	8.062514	0.374377	0.890750
C	7.304131	-1.401875	-0.588912
C	8.338077	-0.688854	0.022763
H	7.526575	-2.226711	-1.259470
H	9.369517	-0.962942	-0.176220
H	8.882229	0.913508	1.355105
H	6.536208	1.571995	1.836015
H	4.601673	-2.684585	-0.533174
H	4.639554	-1.604613	-1.924478
H	2.291112	-2.671539	-1.447094

- **Complex: 3b**

54

	X	Y	Z
C	-2.714423	-1.132587	1.589346
C	-2.555156	-2.175585	0.659009
C	-3.649850	-3.015183	0.368050
C	-4.874974	-2.795122	0.979939
C	-5.028200	-1.749263	1.893777
C	-3.943358	-0.924737	2.197760
C	-1.284041	-2.433823	0.042143
N	-0.184541	-1.681547	-0.018864
C	0.805573	-2.435859	-0.580585
C	0.237326	-3.716979	-0.940434
O	-1.082756	-3.648640	-0.512161
C	2.168026	-2.100098	-0.706021
C	2.759502	-0.983924	-0.104413
C	1.963158	0.011296	0.586914
C	2.597026	0.833703	1.530473
C	3.967792	0.820806	1.707297
C	4.807930	0.022212	0.878559
C	4.218651	-0.859511	-0.069105
Pd	0.104540	0.336449	0.056334
O	-1.835572	0.676904	-0.747750
C	-2.740708	1.388208	-0.218101
O	-2.746267	2.005060	0.843981
H	4.427548	1.476473	2.442144
C	5.065571	-1.592674	-0.917312
O	0.690204	-4.709527	-1.474888
N	0.536648	2.350722	-0.071705
C	0.046168	3.236519	0.811456
C	0.350071	4.589027	0.741057
C	1.180219	5.042852	-0.278547
C	1.682065	4.122934	-1.195214
C	1.343084	2.786002	-1.056134
C	-4.016380	1.448719	-1.109312
F	-4.445765	0.217779	-1.445522
F	-5.036874	2.074954	-0.507062
F	-3.764432	2.111583	-2.259487
H	2.004852	1.530098	2.115791
H	-3.533511	-3.824363	-0.344186
H	-5.716284	-3.437731	0.742265
H	-5.989709	-1.578060	2.367113
H	-4.056154	-0.112221	2.907409
H	-1.876373	-0.489316	1.833960
H	1.722060	2.033553	-1.737712
H	2.331465	4.427612	-2.007095
H	1.432114	6.094845	-0.358587
H	-0.067336	5.266934	1.475892
H	-0.619035	2.838698	1.567145
H	2.778687	-2.861838	-1.174648
C	6.447602	-1.478722	-0.824480
C	6.212338	0.109793	0.969650
C	7.025587	-0.632863	0.130114
H	4.649836	-2.242061	-1.679599
H	7.078189	-2.047223	-1.500108
H	8.104945	-0.552632	0.205473

H	6.650296	0.776294	1.707244

- **Complex: 3c**

62

	X	Y	Z
C	6.166788	-1.164292	-0.570876
C	5.412951	-2.015503	-1.418838
C	4.034836	-2.008157	-1.391803
C	3.318708	-1.152705	-0.524021
C	4.047235	-0.259227	0.295729
C	5.469211	-0.272474	0.286458
C	1.856268	-1.179206	-0.420080
C	1.186367	-0.074055	0.244856
C	1.936979	0.763163	1.030300
C	3.358920	0.669793	1.128980
C	1.155940	-2.305268	-0.856664
C	-0.203860	-2.552264	-0.603885
N	-1.104716	-1.702050	-0.014990
C	-2.218358	-2.388606	0.203212
O	-2.131925	-3.650058	-0.268836
C	-0.859588	-3.821086	-0.810526
C	-3.418921	-2.016314	0.908835
C	-3.421304	-0.946906	1.819956
C	-4.582346	-0.624156	2.508295
C	-5.749018	-1.362617	2.305638
C	-5.748707	-2.437717	1.413880
C	-4.592942	-2.770558	0.721817
Pd	-0.722154	0.304450	-0.124123
N	-0.211444	2.277994	-0.430295
C	-0.700036	3.255678	0.351885
C	-0.332630	4.584236	0.185077
C	0.559072	4.917621	-0.828745
C	1.055373	3.904182	-1.644206
C	0.653072	2.598396	-1.410325
O	-0.511238	-4.876834	-1.303048
O	-2.732437	0.669692	-0.773037
C	-3.593919	1.399085	-0.202569
C	-4.953828	1.400576	-0.962787
F	-4.870828	2.172953	-2.070501
O	-3.508726	2.090104	0.811092
F	-5.315197	0.169224	-1.364764
F	-5.958243	1.885151	-0.217686
C	4.103104	1.537523	1.963068
H	1.458348	1.569969	1.579475
H	-4.594350	-3.600759	0.024240
H	-6.654365	-3.013761	1.255019
H	-6.655851	-1.102388	2.842162
H	-4.577190	0.210000	3.201470
H	-2.516701	-0.372825	1.988612
H	1.029076	1.777558	-2.009055
H	1.749685	4.112753	-2.449352
H	0.861551	5.947963	-0.982477
H	-0.750028	5.337338	0.842772
H	-1.418130	2.949991	1.102840
H	1.684908	-3.140973	-1.299020

H	3.502652	-2.664074	-2.071274
H	5.935252	-2.681001	-2.099725
C	7.582797	-1.166794	-0.563004
C	6.198249	0.606086	1.137505
C	5.476222	1.504721	1.979609
C	8.284869	-0.314005	0.273453
C	7.602956	0.565690	1.117383
H	3.568456	2.241879	2.593805
H	6.031116	2.174588	2.629974
H	8.160953	1.233338	1.767913
H	9.369661	-0.326485	0.273101
H	8.113424	-1.847620	-1.222293

- **Complex: 3d**

56

	X	Y	Z
C	0.395631	3.151041	0.456119
N	0.734380	2.181439	-0.410500
C	1.533639	2.483666	-1.448749
C	2.024553	3.763817	-1.655105
C	1.687566	4.767269	-0.750960
C	0.858324	4.452781	0.320819
Pd	0.136718	0.228529	-0.121496
C	2.012739	-0.242187	0.300716
C	2.817756	0.634809	1.015305
C	4.207298	0.442454	1.096015
C	4.837209	-0.641503	0.446428
C	4.064450	-1.550058	-0.233208
C	2.611375	-1.427116	-0.273649
O	5.038327	1.270642	1.740624
C	4.510375	2.423199	2.393086
O	4.549690	-2.620614	-0.876090
C	5.954028	-2.850389	-0.866537
C	1.871901	-2.495419	-0.796425
C	0.486122	-2.645815	-0.574609
N	-0.356314	-1.744907	-0.000608
C	-1.538254	-2.353352	0.190384
O	-1.523780	-3.612521	-0.306600
C	-0.261703	-3.866442	-0.812677
O	0.032237	-4.931097	-1.318726
C	-2.703113	-1.917265	0.893973
C	-3.903604	-2.654312	0.777796
C	-5.034590	-2.256385	1.473028
C	-4.995768	-1.130259	2.300848
C	-3.808529	-0.406525	2.433853
C	-2.670332	-0.791685	1.742863
O	-1.818848	0.689540	-0.837400
C	-2.618831	1.525734	-0.322902
C	-3.945707	1.623050	-1.132964
F	-4.850937	2.420458	-0.548099
O	-2.497450	2.233224	0.674248
F	-3.718544	2.118675	-2.369195
F	-4.520195	0.415070	-1.288279
H	2.379579	1.508972	1.478395
H	5.914300	-0.723719	0.511909

H	4.022380	3.087698	1.672474
H	5.367925	2.926596	2.835749
H	3.804176	2.136349	3.177957
H	6.485663	-2.017921	-1.338436
H	6.107699	-3.760514	-1.443388
H	6.314474	-2.994044	0.157004
H	-3.939492	-3.523962	0.131239
H	-5.954724	-2.821900	1.367552
H	-5.885656	-0.819222	2.838662
H	-3.771037	0.467547	3.075324
H	-1.753904	-0.223043	1.857249
H	1.786510	1.668292	-2.116050
H	2.663534	3.959978	-2.507745
H	2.063511	5.776416	-0.881244
H	0.562834	5.200510	1.047068
H	-0.274618	2.862693	1.255682
H	2.381849	-3.334641	-1.251794

- **Complex: 3e**

56

	X	Y	Z
C	-2.243362	-0.744747	1.655007
C	-2.292582	-1.555338	0.507746
C	-3.560612	-1.769697	-0.061111
C	-4.702295	-1.190196	0.461787
C	-4.614132	-0.379037	1.589103
C	-3.380073	-0.161683	2.187560
C	-1.110113	-2.155675	-0.046714
N	0.119872	-1.648232	-0.133710
C	0.938867	-2.637118	-0.592982
C	0.117598	-3.808542	-0.852422
O	-1.166963	-3.430707	-0.487896
C	2.339205	-2.578727	-0.715470
C	3.096012	-1.510466	-0.206788
C	2.528194	-0.257942	0.243146
C	3.323546	0.609766	0.977851
C	4.688494	0.341532	1.188500
C	5.302864	-0.810464	0.648881
C	4.533631	-1.710428	-0.044285
Pd	0.710677	0.291549	-0.306448
O	-1.196022	0.831727	-1.119778
C	-2.055691	1.574488	-0.563833
O	-2.033091	2.129212	0.533925
O	5.511057	1.152850	1.858334
C	5.001822	2.357440	2.429630
O	4.998455	-2.842603	-0.585017
C	6.379555	-3.157814	-0.440682
O	0.369595	-4.915109	-1.279142
N	1.390564	2.210475	-0.633803
C	0.877021	3.244548	0.055479
C	1.334462	4.543564	-0.119552
C	2.346268	4.785495	-1.043047
C	2.866399	3.714173	-1.764128
C	2.367350	2.442365	-1.529101
C	-3.308926	1.811552	-1.458689

F	-3.656060	0.717488	-2.159579
F	-4.381393	2.176870	-0.740118
F	-3.065574	2.797340	-2.348808
H	2.902530	1.533570	1.352150
H	6.363933	-0.949612	0.809471
H	4.616673	3.022495	1.650309
H	5.849975	2.824809	2.926203
H	4.218489	2.138375	3.160920
H	7.001684	-2.389216	-0.909843
H	6.519259	-4.108849	-0.950916
H	6.641718	-3.260441	0.616968
F	-3.677742	-2.502964	-1.167488
F	-5.884018	-1.383925	-0.122135
F	-5.707727	0.185052	2.089559
F	-3.297045	0.598157	3.277246
F	-1.087664	-0.549134	2.287661
H	2.754610	1.577746	-2.054652
H	3.651125	3.852553	-2.498237
H	2.721919	5.790830	-1.200518
H	0.894071	5.344871	0.461652
H	0.073417	3.007491	0.743562
H	2.836181	-3.466819	-1.083594

- **Complex: 3f**

58

	X	Y	Z
C	-2.421431	-0.699248	1.194894
C	-2.387564	-1.800833	0.302781
C	-3.598593	-2.483103	0.007755
C	-4.790633	-2.056196	0.549221
C	-4.794122	-0.948896	1.407460
C	-3.612936	-0.275763	1.739943
C	-1.171990	-2.254670	-0.257844
N	0.055615	-1.693591	-0.243003
C	0.928390	-2.611622	-0.723585
C	0.164608	-3.786993	-1.141891
O	-1.141343	-3.489912	-0.826668
C	2.329599	-2.533511	-0.729516
C	3.038849	-1.502700	-0.102068
C	2.423682	-0.286831	0.382026
C	3.162212	0.548740	1.204074
C	4.514383	0.278107	1.489106
C	5.173560	-0.842586	0.939598
C	4.460483	-1.710430	0.150280
Pd	0.634429	0.264616	-0.262724
O	-1.208364	0.809674	-1.166597
C	-2.021432	1.669127	-0.710351
O	-1.960894	2.357494	0.304565
O	5.280497	1.060546	2.249135
C	4.725649	2.237368	2.838373
O	4.971593	-2.810379	-0.411359
C	6.340577	-3.131237	-0.181534
O	0.493509	-4.831593	-1.652463
N	1.338422	2.189595	-0.467488
C	0.970928	3.168918	0.376391

C	1.485026	4.454320	0.278372
C	2.398827	4.741788	-0.730385
C	2.767871	3.727956	-1.610091
C	2.220896	2.464837	-1.444166
C	-3.271588	1.816095	-1.627147
F	-3.869017	0.626761	-1.835288
F	-4.194307	2.639435	-1.112522
F	-2.926321	2.307287	-2.836663
H	2.712654	1.449927	1.598611
H	6.222367	-0.983111	1.165774
H	4.383341	2.931997	2.065267
H	5.537693	2.687737	3.405631
H	3.901306	1.981417	3.509741
H	6.991877	-2.342289	-0.569809
H	6.519524	-4.057975	-0.723102
H	6.525506	-3.282150	0.886397
H	-3.587653	-3.333526	-0.663348
H	-5.719198	-2.560957	0.314715
N	-6.042083	-0.498881	1.971221
H	-3.641313	0.569078	2.415836
H	-1.505387	-0.185296	1.461031
H	2.492703	1.642459	-2.095270
H	3.473199	3.903009	-2.413583
H	2.815428	5.738223	-0.830989
H	1.162323	5.211106	0.983283
H	0.237217	2.903093	1.126171
H	2.859512	-3.393941	-1.118962
O	-6.029498	0.477786	2.722212
O	-7.072153	-1.109641	1.680288

- **Complex: 3g**

55

	X	Y	Z
Pd	-1.089082	0.029849	-0.063780
F	3.369491	1.391329	1.674882
F	2.426895	3.143889	2.535739
F	2.627201	2.973710	0.389765
O	1.332326	-3.321810	0.732536
O	0.011996	-4.910527	1.676623
O	0.706461	1.140403	0.284242
O	0.400769	1.368123	2.514101
N	-0.198323	-1.767959	0.235747
N	-1.994087	1.877218	-0.180376
C	-2.800676	-0.889459	-0.422348
C	-3.755971	-0.296688	-1.256967
H	-3.494153	0.597765	-1.814584
C	-5.058790	-0.780136	-1.390056
C	-5.446536	-1.915183	-0.634035
H	-6.467511	-2.280092	-0.702691
C	-4.536090	-2.562015	0.156970
H	-4.823263	-3.455409	0.704619
C	-3.174840	-2.116403	0.242724
C	-6.052152	-0.107133	-2.291085
H	-5.619208	0.763107	-2.789368
H	-6.931127	0.219340	-1.723782

H	-6.409644	-0.802644	-3.059084
C	-2.252866	-2.958817	0.884443
C	-0.860650	-2.805948	0.784684
C	0.118323	-3.828033	1.145856
C	1.120278	-2.096598	0.184689
C	2.163740	-1.365380	-0.376171
C	3.473313	-1.796428	-0.407842
C	4.575568	-1.053290	-0.954493
C	5.859878	-1.646008	-0.962627
H	5.981873	-2.646840	-0.557704
C	6.955975	-0.970239	-1.478818
H	7.932688	-1.443449	-1.476591
C	6.802750	0.317840	-1.998146
H	7.659936	0.849103	-2.399621
C	5.541635	0.923822	-1.994530
H	5.421985	1.927541	-2.389979
C	4.442196	0.253754	-1.482336
H	3.477425	0.749913	-1.476388
C	-1.482725	2.809630	-1.002558
H	-0.636402	2.506103	-1.606872
C	-1.999720	4.094889	-1.071397
H	-1.560106	4.811266	-1.754927
C	-3.072100	4.434176	-0.251496
H	-3.494021	5.432981	-0.280121
C	-3.591049	3.470811	0.608255
H	-4.420349	3.689362	1.270204
C	-3.029635	2.202678	0.612540
H	-3.407219	1.418194	1.256838
C	1.000465	1.529605	1.455700
C	2.364698	2.279610	1.511764
H	-2.603522	-3.866161	1.366321
H	1.876058	-0.401190	-0.775254
H	3.706420	-2.770946	0.015469

- **Complex: 3h**

60

	X	Y	Z
C	-2.247423	2.625957	0.894568
N	-1.300013	2.229573	0.027430
C	-0.731539	3.140860	-0.780527
C	-1.104265	4.476947	-0.762128
C	-2.087023	4.889428	0.133139
C	-2.662972	3.946573	0.979347
Pd	-0.633894	0.280970	-0.014784
C	-2.467642	-0.382369	-0.391562
C	-3.332225	0.412016	-1.137419
C	-4.695916	0.096187	-1.254583
C	-5.244770	-1.030478	-0.609760
C	-4.410020	-1.861379	0.098087
C	-2.977579	-1.608612	0.173994
O	-5.576925	0.838557	-1.938423
C	-2.165775	-2.618180	0.703059
C	-0.765977	-2.625671	0.583602
N	0.024068	-1.633786	0.119770
C	1.288055	-2.120461	0.027331

O	1.342425	-3.408018	0.463014
C	0.074745	-3.790819	0.840357
O	-0.169167	-4.895790	1.272982
C	2.422801	-1.485719	-0.475417
C	3.665434	-2.074177	-0.542389
C	4.860933	-1.437127	-1.032652
C	6.060406	-2.182902	-1.081404
C	7.239440	-1.614238	-1.543425
C	7.256250	-0.283297	-1.967088
C	6.080368	0.472816	-1.922123
C	4.899207	-0.090290	-1.463804
O	1.289066	1.148389	0.337351
C	1.664422	1.388372	1.524737
C	3.085905	2.020767	1.599404
F	3.432655	2.681632	0.481332
O	1.098672	1.164810	2.591068
F	4.008321	1.053369	1.792823
F	3.195425	2.886174	2.620578
H	-2.961252	1.311450	-1.609550
H	-6.308270	-1.208806	-0.700518
O	-4.819586	-2.965398	0.738123
H	6.052443	-3.217942	-0.750547
H	8.148784	-2.206282	-1.572598
H	8.178020	0.163793	-2.325811
H	6.090521	1.509864	-2.242957
H	4.003983	0.521519	-1.425497
H	0.040342	2.776690	-1.447874
H	-0.624258	5.174619	-1.437805
H	-2.396141	5.928361	0.172818
H	-3.425680	4.220979	1.698156
H	-2.675513	1.855291	1.523857
H	-2.619183	-3.514679	1.107071
H	2.262228	-0.464429	-0.796789
H	3.771039	-3.099533	-0.194870
C	-6.196191	-3.321620	0.685704
C	-5.133584	2.025132	-2.592014
H	-6.509820	-3.501281	-0.347534
H	-6.284788	-4.239995	1.263211
H	-6.816393	-2.540042	1.136025
H	-6.018623	2.445174	-3.066558
H	-4.728668	2.739316	-1.867736
H	-4.381873	1.793536	-3.352530

- **Complex: 3i**

63

	X	Y	Z
C	8.738311	-1.350777	0.385540
C	8.515817	-0.262472	1.235438
C	7.227845	0.235613	1.431104
C	6.169837	-0.375474	0.760474
C	6.391605	-1.471391	-0.090995
C	7.675765	-1.963477	-0.284292
C	5.082281	-1.945627	-0.674134
C	4.090531	-0.975256	-0.083728
C	4.732205	-0.081407	0.757387

C	2.674986	-0.945829	-0.255442
C	1.936076	0.134310	0.377021
C	2.625331	0.983545	1.257391
C	3.997014	0.889162	1.463905
Pd	0.078831	0.536449	-0.136652
O	-1.844587	1.052397	-0.952720
C	-2.814956	1.476366	-0.262636
O	-2.921251	1.580586	0.960776
C	2.050199	-2.003394	-0.939756
C	0.669548	-2.252892	-0.877791
N	-0.264533	-1.448605	-0.329307
C	-1.434723	-2.129732	-0.290773
O	-1.297724	-3.352848	-0.861854
C	0.016564	-3.498628	-1.265602
C	-2.636746	-1.728136	0.293246
C	-3.790028	-2.477983	0.245815
C	-5.056861	-2.109630	0.821482
O	0.419900	-4.502319	-1.809236
N	0.480822	2.559165	-0.102569
C	-0.206259	3.369566	0.721567
C	0.007539	4.740182	0.751200
C	0.954649	5.294066	-0.104664
C	1.658317	4.452992	-0.961910
C	1.397949	3.091367	-0.928942
C	-4.034503	1.874776	-1.142908
F	-4.504710	0.806951	-1.818219
F	-5.049559	2.369567	-0.422469
F	-3.695587	2.808872	-2.053277
H	4.492321	1.573321	2.147139
H	2.082543	1.765152	1.778235
H	1.931678	2.397761	-1.567594
H	2.403492	4.837599	-1.647837
H	1.141147	6.362558	-0.104344
H	-0.566102	5.353872	1.435380
H	-0.945072	2.892204	1.355459
H	7.855021	-2.809689	-0.941302
H	9.748131	-1.724247	0.245981
H	9.354666	0.198819	1.747399
H	7.059421	1.081341	2.091359
H	4.864681	-2.980576	-0.380556
H	5.084190	-1.921201	-1.771024
H	2.653026	-2.770668	-1.413588
C	-6.142738	-3.005768	0.692925
C	-7.388114	-2.699259	1.221946
C	-7.583705	-1.488489	1.891104
C	-6.522069	-0.587708	2.025447
C	-5.273911	-0.887841	1.502020
H	-5.993127	-3.946482	0.170283
H	-8.209379	-3.400347	1.113133
H	-8.558104	-1.245643	2.303237
H	-6.673907	0.356337	2.539036
H	-4.470638	-0.165104	1.607564
H	-3.758936	-3.429088	-0.281370
H	-2.603860	-0.772177	0.803858

- **Complex: 3j**

60

	X	Y	Z
C	4.914764	-0.522422	-1.503084
C	4.764673	-1.811581	-0.937403
C	5.896298	-2.659340	-0.902559
C	7.117297	-2.242749	-1.412557
C	7.244291	-0.967218	-1.968570
C	6.137677	-0.111988	-2.008918
C	3.524111	-2.290352	-0.391379
C	2.336631	-1.590229	-0.385868
C	1.157291	-2.070583	0.178543
N	-0.057019	-1.462155	0.217703
C	-0.932178	-2.321400	0.778210
C	-0.200124	-3.527086	1.158860
O	1.096780	-3.303380	0.744341
C	-2.323433	-2.171605	0.876483
C	-3.043338	-1.160126	0.218680
C	-2.403479	-0.048889	-0.455043
C	-3.204170	0.726275	-1.295402
C	-4.577915	0.526822	-1.415876
C	-5.227215	-0.475511	-0.670342
C	-4.466543	-1.312288	0.136155
Pd	-0.535752	0.482217	-0.092145
O	1.457216	1.181416	0.232935
C	1.862317	1.476483	1.398729
O	1.280654	1.408752	2.476947
H	-5.146760	1.177248	-2.070687
O	-5.056161	-2.303524	0.861243
C	-5.517553	-3.424896	0.100222
O	-0.539712	-4.553615	1.702040
N	-1.023562	2.480394	-0.209790
C	-1.962368	3.018615	0.587845
C	-2.235016	4.378459	0.591404
C	-1.521108	5.210784	-0.265407
C	-0.550135	4.651094	-1.090865
C	-0.324275	3.284006	-1.029675
C	3.347419	1.946188	1.417812
F	3.594280	2.798913	2.423846
F	3.720460	2.556964	0.279109
F	4.162490	0.881851	1.584484
H	-2.766956	1.542436	-1.860314
O	-6.562590	-0.696485	-0.689094
H	5.800298	-3.650617	-0.468244
H	7.973636	-2.908481	-1.376615
H	8.199742	-0.638459	-2.365022
H	6.236601	0.881931	-2.433666
H	4.074658	0.163365	-1.532247
H	0.434478	2.806687	-1.637958
H	0.033165	5.258460	-1.772447
H	-1.715887	6.277566	-0.287549
H	-2.995522	4.768217	1.257273
H	-2.499841	2.331242	1.229559
H	-2.864101	-2.969558	1.371642
H	2.263069	-0.596395	-0.808335

H	3.540779	-3.281091	0.057242
H	-5.934517	-4.129732	0.819432
H	-6.288048	-3.120983	-0.613255
H	-4.678283	-3.891123	-0.428289
C	-7.374155	0.113141	-1.527995
H	-8.392743	-0.245851	-1.387504
H	-7.317269	1.167120	-1.235811
H	-7.090996	0.001877	-2.580172

- **Complex: 3k**

58

	X	Y	Z
C	-2.421431	-0.699249	1.194894
C	-2.387564	-1.800833	0.302780
C	-3.598593	-2.483102	0.007753
C	-4.790633	-2.056195	0.549220
C	-4.794122	-0.948896	1.407460
C	-3.612936	-0.275763	1.739944
C	-1.171990	-2.254670	-0.257845
N	0.055615	-1.693591	-0.243004
C	0.928390	-2.611623	-0.723585
C	0.164608	-3.786993	-1.141891
O	-1.141343	-3.489912	-0.826668
C	2.329599	-2.533512	-0.729515
C	3.038849	-1.502700	-0.102067
C	2.423682	-0.286831	0.382026
C	3.162212	0.548741	1.204073
C	4.514383	0.278107	1.489106
C	5.173560	-0.842586	0.939599
C	4.460483	-1.710430	0.150280
Pd	0.634429	0.264617	-0.262725
O	-1.208364	0.809674	-1.166597
C	-2.021432	1.669127	-0.710351
O	-1.960894	2.357493	0.304566
O	5.280497	1.060546	2.249135
C	4.725649	2.237369	2.838372
O	4.971593	-2.810379	-0.411358
C	6.340577	-3.131238	-0.181533
O	0.493508	-4.831593	-1.652463
N	1.338422	2.189595	-0.467488
C	0.970927	3.168918	0.376391
C	1.485026	4.454320	0.278372
C	2.398827	4.741788	-0.730384
C	2.767872	3.727956	-1.610091
C	2.220897	2.464838	-1.444166
C	-3.271588	1.816095	-1.627147
F	-3.869017	0.626760	-1.835289
F	-4.194308	2.639434	-1.112521
F	-2.926321	2.307289	-2.836663
H	2.712654	1.449927	1.598610
H	6.222367	-0.983111	1.165775
H	4.383341	2.931998	2.065265
H	5.537693	2.687738	3.405630
H	3.901306	1.981417	3.509740
H	6.991877	-2.342290	-0.569808

H	6.519523	-4.057976	-0.723101
H	6.525505	-3.282150	0.886398
H	-3.587654	-3.333525	-0.663350
H	-5.719198	-2.560956	0.314713
N	-6.042083	-0.498881	1.971222
H	-3.641313	0.569077	2.415838
H	-1.505387	-0.185297	1.461032
H	2.492703	1.642459	-2.095270
H	3.473199	3.903009	-2.413582
H	2.815428	5.738223	-0.830988
H	1.162322	5.211106	0.983284
H	0.237216	2.903093	1.126171
H	2.859512	-3.393942	-1.118961
O	-6.029497	0.477786	2.722213
O	-7.072153	-1.109640	1.680288

- **Complex: 3m**

60

	X	Y	Z
C	4.916526	-0.084416	-1.541197
C	4.931081	-1.411489	-1.055519
C	6.148443	-2.124129	-1.110021
C	7.297385	-1.541622	-1.629227
C	7.262983	-0.229709	-2.106068
C	6.067057	0.493444	-2.056840
C	3.766753	-2.062206	-0.501268
C	2.514101	-1.507548	-0.421168
C	1.408384	-2.151542	0.142769
N	0.139760	-1.695701	0.250899
C	-0.613338	-2.686222	0.783220
C	0.265356	-3.814866	1.065974
O	1.510963	-3.416450	0.631521
C	-2.007582	-2.703330	0.934621
C	-2.864854	-1.743599	0.370408
C	-2.383150	-0.530880	-0.256842
C	-3.297868	0.206922	-1.013943
C	-4.648894	-0.124985	-1.087113
C	-5.147020	-1.263020	-0.362468
C	-4.264885	-2.040943	0.341220
Pd	-0.573333	0.196126	0.054120
O	1.333613	1.120572	0.326626
C	1.726228	1.456536	1.485236
O	1.182733	1.309334	2.575741
O	-5.565487	0.561611	-1.770586
C	-5.160978	1.720026	-2.495605
H	-4.606287	-2.931478	0.855854
O	0.065998	-4.906891	1.553944
N	-1.292038	2.126645	0.031591
C	-2.238160	2.531449	0.896493
C	-2.689829	3.842141	0.934306
C	-2.152310	4.765532	0.042520
C	-1.170327	4.344709	-0.849680
C	-0.760029	3.019881	-0.820237
C	3.140180	2.109795	1.478036
F	3.284245	3.006765	2.466187

F	3.423962	2.741030	0.325139
F	4.084658	1.160992	1.656177
H	-2.959014	1.090848	-1.537989
O	-6.471508	-1.468076	-0.465194
H	6.180572	-3.144583	-0.737922
H	8.222464	-2.108629	-1.661399
H	8.160591	0.228493	-2.509273
H	6.036935	1.516361	-2.419134
H	4.004843	0.502528	-1.500964
H	0.013295	2.650650	-1.483094
H	-0.719266	5.027671	-1.559444
H	-2.490246	5.796203	0.045028
H	-3.450217	4.124015	1.652657
H	-2.635764	1.775404	1.562483
H	-2.429577	-3.592396	1.391653
H	2.315434	-0.505516	-0.779714
H	3.914211	-3.067887	-0.113715
H	-6.066981	2.104724	-2.960014
H	-4.741815	2.472755	-1.819808
H	-4.430437	1.458843	-3.267416
C	-7.028954	-2.578836	0.224884
H	-8.097838	-2.548381	0.020449
H	-6.609225	-3.519465	-0.147257
H	-6.856473	-2.494995	1.303198

- **Complex: 4d**

60

	X	Y	Z
C	-2.815232	-0.231862	1.876379
C	-3.052866	-1.324772	1.015193
C	-4.368865	-1.820785	0.882613
C	-5.410086	-1.219773	1.573334
C	-5.165979	-0.131620	2.416651
C	-3.864017	0.351519	2.569266
C	-1.977888	-1.957361	0.318385
N	-0.727346	-1.520708	0.093944
C	-0.017806	-2.545450	-0.450238
C	-0.929122	-3.662335	-0.640816
O	-2.141973	-3.220783	-0.136813
C	1.372123	-2.580320	-0.681854
C	2.244685	-1.590874	-0.207337
C	1.812520	-0.312807	0.317071
C	2.737191	0.484779	0.972342
C	4.094239	0.116497	1.051848
C	4.567068	-1.070959	0.455428
C	3.671414	-1.902029	-0.171164
Pd	-0.012901	0.368919	-0.115288
N	-1.908990	1.069750	-0.858965
O	5.031642	0.859957	1.646141
C	4.671371	2.104725	2.242935
O	4.001854	-3.054585	-0.763397
C	5.364878	-3.468127	-0.759868
O	-0.788199	-4.770588	-1.112738
N	0.755991	2.251325	-0.425719
C	0.680464	3.196103	0.527933

C	1.188257	4.473063	0.344715
C	1.795159	4.786548	-0.868500
C	1.869703	3.807991	-1.854784
C	1.339626	2.551785	-1.597232
H	2.427890	1.429283	1.399418
H	5.624920	-1.291013	0.516425
H	4.262727	2.789898	1.493248
H	5.595913	2.511359	2.648155
H	3.948813	1.953412	3.050372
H	5.991149	-2.737020	-1.280454
H	5.385078	-4.417460	-1.291510
H	5.721587	-3.608164	0.265130
H	-4.561751	-2.664191	0.228687
H	-6.419712	-1.599636	1.456086
H	-5.985057	0.332701	2.956186
H	-3.668063	1.186340	3.234381
H	-1.803239	0.139149	2.005853
H	1.383198	1.756902	-2.332184
H	2.333985	4.004712	-2.813555
H	2.204704	5.775792	-1.040715
H	1.107464	5.200518	1.143416
H	0.211880	2.902622	1.461452
H	1.769852	-3.493200	-1.105959
C	-2.603849	2.066605	-0.292518
C	-3.846014	2.472174	-0.760167
C	-4.391965	1.820079	-1.860810
C	-3.668424	0.791274	-2.458460
C	-2.433579	0.448215	-1.928385
H	-2.153433	2.541479	0.571758
H	-4.371188	3.277092	-0.260028
H	-5.364035	2.107419	-2.246766
H	-4.050795	0.255613	-3.319085
H	-1.840659	-0.352187	-2.359147

- **Complex: 4h**

64

	X	Y	Z
C	-5.027605	-0.368090	-1.426162
C	-5.080619	0.951263	-0.918730
C	-6.347261	1.569204	-0.812643
C	-7.504017	0.902241	-1.191877
C	-7.430487	-0.401225	-1.688174
C	-6.186208	-1.029696	-1.803105
C	-3.915262	1.684380	-0.495162
C	-2.624446	1.206817	-0.499524
C	-1.522066	1.928777	-0.044408
N	-0.235522	1.512401	0.073574
C	0.507381	2.573045	0.460751
C	-0.393259	3.705584	0.660605
O	-1.643330	3.232396	0.323518
C	1.906848	2.642307	0.549311
C	2.756120	1.639994	0.065304
C	2.301552	0.346046	-0.389615
C	3.188688	-0.453716	-1.095664
C	4.529202	-0.069981	-1.285782

C	5.029697	1.133119	-0.750468
C	4.169322	1.967397	-0.078244
Pd	0.517062	-0.369789	0.165172
N	-1.309746	-1.165878	0.992443
O	5.428774	-0.813402	-1.937940
C	5.039793	-2.072379	-2.483641
O	4.530359	3.136881	0.462642
C	5.881706	3.568811	0.337924
O	-0.205588	4.842972	1.028095
N	1.328714	-2.253303	0.370281
C	1.974216	-2.576877	1.502650
C	2.526788	-3.833913	1.702876
C	2.408567	-4.790153	0.699268
C	1.737439	-4.453604	-0.473283
C	1.212183	-3.176884	-0.600179
H	2.865424	-1.409612	-1.483617
H	6.077290	1.363849	-0.892971
H	-6.409825	2.582808	-0.426149
H	-8.465961	1.396633	-1.101443
H	-8.334069	-0.924247	-1.984666
H	-6.124612	-2.042137	-2.189980
H	-4.075457	-0.878919	-1.525078
H	0.696722	-2.866130	-1.502855
H	1.620830	-5.162760	-1.283976
H	2.833583	-5.779629	0.826944
H	3.041773	-4.048523	2.631458
H	2.048040	-1.799590	2.253899
H	2.323411	3.585398	0.880243
H	-2.399364	0.207690	-0.850879
H	-4.087081	2.690351	-0.119229
H	6.152151	3.688392	-0.715691
H	5.930097	4.531343	0.843516
H	6.560173	2.860282	0.823093
H	5.934205	-2.473530	-2.956492
H	4.703964	-2.750485	-1.692643
H	4.252049	-1.945129	-3.231980
C	-1.805751	-0.608011	2.108677
C	-3.009497	-1.009137	2.670512
C	-3.731411	-2.026975	2.054483
C	-3.211786	-2.617222	0.906123
C	-1.998645	-2.160916	0.411468
H	-1.220935	0.194983	2.544934
H	-3.371400	-0.521825	3.567877
H	-4.682178	-2.353878	2.461170
H	-3.736998	-3.412683	0.390981
H	-1.569616	-2.586932	-0.488718

- **Complex: 5h**

54

	X	Y	Z
C	5.332757	-1.073389	0.703683
C	5.435984	0.330279	0.564335
C	6.701864	0.925043	0.764825
C	7.809208	0.157866	1.100595
C	7.685800	-1.226266	1.241092

C	6.442973	-1.834717	1.038153
C	4.317591	1.173907	0.223112
C	3.015886	0.752484	0.084640
C	1.960081	1.595690	-0.261846
N	0.642218	1.293089	-0.357704
C	-0.029088	2.444631	-0.577412
C	0.947945	3.520633	-0.701648
O	2.168660	2.922226	-0.480044
C	-1.421475	2.638817	-0.537921
C	-2.304636	1.684630	-0.018571
C	-1.913770	0.338744	0.328734
C	-2.777916	-0.421228	1.109891
C	-4.049955	0.055229	1.462552
C	-4.507409	1.316474	1.027131
C	-3.661024	2.111928	0.293211
Pd	-0.296304	-0.506039	-0.478348
Cl	1.515675	-1.637050	-1.669390
O	-4.933099	-0.644144	2.189019
C	-4.585907	-1.951706	2.637274
O	-3.983527	3.331576	-0.161721
C	-5.274670	3.854050	0.127431
O	0.836870	4.705415	-0.931820
N	-1.341164	-2.274168	-0.654023
C	-2.445929	-2.351563	-1.414323
C	-3.171817	-3.526437	-1.545619
C	-2.749592	-4.659117	-0.856358
C	-1.604038	-4.576933	-0.070235
C	-0.921530	-3.370870	-0.001513
H	-2.487500	-1.416075	1.419507
H	-5.511235	1.620338	1.294276
H	6.802793	2.001571	0.656270
H	8.771466	0.636783	1.252383
H	8.550920	-1.827958	1.501417
H	6.344566	-2.911327	1.136637
H	4.382817	-1.570572	0.534742
H	-0.016625	-3.264552	0.584546
H	-1.233007	-5.432235	0.481709
H	-3.301439	-5.589825	-0.932448
H	-4.053519	-3.541443	-2.175076
H	-2.749373	-1.441086	-1.916700
H	-1.779344	3.635243	-0.765079
H	2.734403	-0.283012	0.229878
H	4.537629	2.227886	0.067625
H	-5.422041	3.945023	1.208313
H	-5.303733	4.840009	-0.332910
H	-6.055357	3.220353	-0.305548
H	-5.454229	-2.313234	3.185269
H	-4.382839	-2.612422	1.788048
H	-3.717449	-1.917944	3.302123

- **Complex: 6h**

96

	X	Y	Z
O	-0.078025	5.175868	-1.536190
C	-0.383109	4.024042	-1.305975

O	-1.700280	3.602755	-1.263739
C	-1.710051	2.278247	-0.965947
C	-2.899166	1.554198	-0.827598
C	-4.154994	2.066913	-1.026319
C	-5.373010	1.334848	-0.745164
C	-5.401241	0.269004	0.179939
C	-6.587297	-0.403950	0.440839
C	-7.766301	-0.042426	-0.214088
C	-7.755680	1.017065	-1.125031
C	-6.576804	1.707139	-1.378096
N	-0.452791	1.807557	-0.821815
C	0.397828	2.823178	-1.038805
C	1.804234	2.718631	-1.137494
C	2.462970	1.496787	-1.314437
C	3.849962	1.565790	-1.761559
O	4.419952	2.767970	-1.625555
C	5.755307	2.960281	-2.077612
C	4.483715	0.481148	-2.330926
C	3.772955	-0.727982	-2.414845
O	4.264309	-1.829279	-2.998201
C	5.572441	-1.802824	-3.559234
C	2.504492	-0.871963	-1.819746
C	1.842166	0.181315	-1.220083
Pd	0.203367	-0.012420	-0.108412
C	0.719248	-1.850221	0.516356
N	-0.063080	-2.941361	0.320113
C	-1.376926	-2.903008	-0.268513
C	-2.484979	-2.885647	0.589592
C	-2.321592	-2.965566	2.083695
C	-3.751823	-2.789205	0.013808
C	-3.929530	-2.714679	-1.370004
C	-5.301551	-2.565529	-1.975399
C	-2.799512	-2.765963	-2.186219
C	-1.510486	-2.862758	-1.658518
C	-0.322335	-2.902880	-2.580976
C	0.502745	-4.067985	0.896793
C	1.662571	-3.671286	1.466606
N	1.785111	-2.311901	1.218088
C	2.845817	-1.475417	1.706845
C	4.119791	-1.569037	1.141618
C	4.493471	-2.664526	0.178853
C	5.082928	-0.647190	1.558888
C	4.806379	0.322296	2.522471
C	5.837503	1.355119	2.898990
C	3.547456	0.316354	3.129369
C	2.555072	-0.586035	2.750473
C	1.244705	-0.653152	3.490793
O	-1.412286	-0.031843	1.324803
C	-1.912193	0.749093	2.181706
C	-1.118572	2.048232	2.551930
F	-1.700827	3.125642	1.979387
F	0.168060	2.050397	2.167043
F	-1.125126	2.253851	3.881492
O	-2.992204	0.634862	2.760641
H	-2.769386	0.520030	-0.523106

H	-4.260173	3.079184	-1.411042
H	-4.500403	-0.001655	0.723343
H	-6.595507	-1.213418	1.164538
H	-8.689781	-0.574959	-0.008686
H	-8.670439	1.307353	-1.632546
H	-6.573036	2.535570	-2.081332
H	2.349095	3.644726	-1.266084
H	5.829630	2.778296	-3.154461
H	5.991714	4.000813	-1.863123
H	6.444761	2.304682	-1.535878
H	5.491021	0.563972	-2.713616
H	6.322344	-1.595804	-2.788490
H	5.736872	-2.797512	-3.969726
H	5.639490	-1.058404	-4.358737
H	2.089164	-1.870957	-1.821769
H	-2.000062	-3.965228	2.395583
H	-3.266157	-2.740883	2.582948
H	-1.576587	-2.245649	2.430367
H	-4.621705	-2.761296	0.665244
H	-6.076838	-2.951499	-1.308451
H	-5.526001	-1.508350	-2.156010
H	-5.370080	-3.091308	-2.931942
H	-2.919790	-2.731170	-3.266605
H	-0.607925	-3.308984	-3.554096
H	0.071964	-1.894096	-2.741022
H	0.485992	-3.518653	-2.177003
H	0.027529	-5.034290	0.845111
H	2.410371	-4.219719	2.015952
H	3.655383	-2.984116	-0.441676
H	4.848637	-3.541018	0.733147
H	5.302883	-2.341495	-0.478561
H	6.074024	-0.690525	1.112886
H	5.746483	1.643933	3.949629
H	6.852562	0.986536	2.728189
H	5.706494	2.260684	2.296039
H	3.333043	1.029119	3.921903
H	1.150144	-1.617177	4.003203
H	0.389437	-0.557528	2.820133
H	1.184929	0.137683	4.240783

10.3.2. M06-2X

***Table S7**. Calculated emission properties from the S_1 optimized geometry for the lowest T_1, T_2, T_3, S_1 and S_2 excited states with the M06-2X functional. Calculated and experimental wave lengths in nm, and oscillator strengths for S_1 and S_2 states in parenthesis.*

Compound	T_1	T_2	T_3	S_1	S_2	
**3a**	1429	905	866	489 (0.586)	395 (0.5749)	521, 542
**3b**	1024	521	451	541 (0.759)	387 (0.032)	525, 550
**3c**	1087	537	530	585 (1.471)	409 (0.128)	577
**3d**	890	533	420	509 (0.904)	394 (0.036)	539
**3e**	887	547	443	518 (0.853)	401 (0.040)	534, 564
**3f**	971	537	491	566 (0.952)	399 (0.039)	630
**3g**	1053	523	415	532 (1.3525)	389 (0.0731)	534
**3h**	1087	519	504	560 (1.3015)	388 (0.0721)	539, 570
**3i**	1031	513	504	532 (1.212)	407 (0.536)	544, 574
**3j**	1040	514	498	526 (1.262)	405 (0.347)	533
**3k**	1014	560	503	514 (1.345)	411 (0.050)	510, 550
**3m**	1077	524	512	561 (1.167)	389 (0.131)	605
**4d**	889	503	426	516 (0.884)	379 (0.042)	526, 553
**4h**	1078	511	505	567 (1.300)	383 (0.079)	558, 590
**5h**	1087	542	504	562 (1.273)	401 (0.062)	542, 593
**6h**	1086	510	443	560 (1.301)	355 (0.107)	542, 593

Cartesian Coordinates

- **Complex: 3a**

59

	X	Y	Z
C	-1.257021	3.686924	0.229081
N	-0.363906	2.853767	-0.322942
C	0.573674	3.351847	-1.140664
C	0.654684	4.705665	-1.445107
C	-0.269159	5.574774	-0.869044
C	-1.239587	5.056929	-0.016007
Pd	-0.425027	0.596731	-0.011766
C	1.683979	0.398437	0.309588
C	2.388062	1.450577	0.911631
C	3.778669	1.445394	1.058377
C	4.499946	0.357488	0.569883
C	3.833318	-0.714180	-0.038083
C	2.430145	-0.715122	-0.154349

H	4.284586	2.281433	1.535011
C	4.832751	-1.767110	-0.472071
C	1.818045	-1.895372	-0.747154
C	0.535485	-2.317286	-0.681647
N	-0.563862	-1.747408	-0.019151
C	-1.539079	-2.589280	-0.142759
O	-1.244267	-3.701952	-0.876292
C	0.084088	-3.590623	-1.267390
O	0.626475	-4.431667	-1.929767
C	-2.873267	-2.528086	0.440348
C	-3.886356	-3.366747	-0.043054
C	-5.151382	-3.317557	0.533131
C	-5.404710	-2.442747	1.590438
C	-4.391568	-1.614503	2.076973
C	-3.124124	-1.658560	1.508309
O	-2.638606	0.646813	-0.659116
C	-3.475851	1.023712	0.201116
C	-4.942833	0.922146	-0.298824
F	-5.839945	1.036720	0.688398
O	-3.293043	1.468224	1.338361
F	-5.200863	1.908123	-1.184707
F	-5.180234	-0.240903	-0.924193
H	1.840074	2.318843	1.273077
C	5.944862	0.083082	0.561485
H	-3.681835	-4.042833	-0.866131
H	-5.940028	-3.959801	0.156100
H	-6.393505	-2.405170	2.036417
H	-4.587908	-0.930323	2.895498
H	-2.332239	-1.023445	1.891791
H	1.281513	2.637194	-1.551855
H	1.427876	5.062583	-2.115353
H	-0.232499	6.637994	-1.082444
H	-1.978267	5.695663	0.454033
H	-1.999682	3.228973	0.876281
C	6.153974	-1.163652	-0.052387
C	7.019513	0.832778	1.037604
C	8.309146	0.319928	0.890321
C	7.439259	-1.670860	-0.197968
C	8.518430	-0.920015	0.278146
H	7.606484	-2.633962	-0.672215
H	9.528638	-1.302779	0.172173
H	9.159059	0.888713	1.254335
H	6.861584	1.796484	1.513400
H	4.655427	-2.730078	0.021475
H	4.797163	-1.949609	-1.553261
H	2.475550	-2.578695	-1.282945

- **Complex: 3b**

54

	X	Y	Z
C	-2.757039	-1.112497	1.485830
C	-2.591732	-2.152971	0.552095
C	-3.689714	-2.976740	0.225829
C	-4.927371	-2.739669	0.805581
C	-5.089623	-1.693288	1.719300

C	-4.000265	-0.885914	2.058578
C	-1.309448	-2.415463	-0.034297
N	-0.200517	-1.676848	-0.033072
C	0.790500	-2.417385	-0.604780
C	0.215936	-3.684503	-1.025414
O	-1.112214	-3.614461	-0.629077
C	2.160551	-2.105904	-0.695909
C	2.760503	-1.001102	-0.077682
C	1.970701	0.034292	0.555949
C	2.586506	0.880894	1.486721
C	3.951687	0.835313	1.722421
C	4.796324	-0.026182	0.968362
C	4.219795	-0.927703	0.029550
Pd	0.138948	0.385909	0.018057
O	-1.868153	0.764129	-0.728384
C	-2.756905	1.380768	-0.073951
O	-2.685578	1.965751	1.005510
H	4.402668	1.506710	2.448492
C	5.080374	-1.729508	-0.739286
O	0.676131	-4.657645	-1.581903
N	0.648347	2.418460	-0.225662
C	0.010521	3.392059	0.444067
C	0.338737	4.733998	0.281564
C	1.350390	5.079362	-0.608702
C	2.004760	4.067186	-1.307099
C	1.629140	2.749793	-1.083195
C	-4.131001	1.367324	-0.797075
F	-4.461079	0.134898	-1.213218
F	-5.125514	1.805767	-0.016461
F	-4.099843	2.160071	-1.887702
H	1.989426	1.619259	2.013847
H	-3.561600	-3.783349	-0.487740
H	-5.772128	-3.367952	0.543400
H	-6.061450	-1.508238	2.165456
H	-4.120472	-0.072654	2.766567
H	-1.912287	-0.486827	1.756928
H	2.122031	1.929117	-1.593705
H	2.797507	4.285089	-2.012773
H	1.625169	6.118372	-0.756939
H	-0.199969	5.485587	0.846426
H	-0.787484	3.071791	1.105195
H	2.764779	-2.881396	-1.150683
C	6.461197	-1.660283	-0.579719
C	6.197921	0.016949	1.127434
C	7.024333	-0.791880	0.364027
H	4.680465	-2.399982	-1.491319
H	7.101488	-2.283279	-1.195274
H	8.100667	-0.747107	0.490953
H	6.620542	0.701273	1.857753

- **Complex: 3c**

62

	X	Y	Z
C	6.180246	-1.209445	-0.432067
C	5.445948	-2.073359	-1.285665

C	4.067329	-2.049543	-1.308337
C	3.329697	-1.161846	-0.488291
C	4.042345	-0.258193	0.336892
C	5.463866	-0.289323	0.378949
C	1.865388	-1.167073	-0.441800
C	1.190308	-0.053315	0.195755
C	1.921347	0.798866	0.985812
C	3.339302	0.697493	1.127531
C	1.164630	-2.283402	-0.913366
C	-0.196347	-2.532328	-0.681281
N	-1.102669	-1.719085	-0.057328
C	-2.222015	-2.413238	0.096172
O	-2.130133	-3.651070	-0.441613
C	-0.850462	-3.795301	-0.966742
C	-3.432448	-2.069986	0.793789
C	-3.461826	-0.989704	1.693285
C	-4.634749	-0.682573	2.369056
C	-5.784234	-1.451381	2.169226
C	-5.754606	-2.538527	1.290647
C	-4.588614	-2.853845	0.607382
Pd	-0.695261	0.335746	-0.166071
N	-0.150442	2.332198	-0.575990
C	-0.744908	3.363155	0.046349
C	-0.403934	4.684288	-0.224996
C	0.573553	4.947758	-1.179046
C	1.181713	3.876804	-1.829663
C	0.797171	2.585374	-1.495433
O	-0.487117	-4.818161	-1.507026
O	-2.766401	0.751028	-0.725523
C	-3.577382	1.414071	-0.019683
C	-5.010601	1.406257	-0.618561
F	-5.050797	2.115907	-1.764492
O	-3.398517	2.039410	1.024785
F	-5.417611	0.160596	-0.911199
F	-5.918193	1.938806	0.208298
C	4.065034	1.575374	1.968609
H	1.428926	1.616633	1.507493
H	-4.563470	-3.691890	-0.080642
H	-6.646029	-3.137394	1.136104
H	-6.699832	-1.204853	2.697195
H	-4.651696	0.163015	3.048602
H	-2.569317	-0.394303	1.858475
H	1.256214	1.722264	-1.965447
H	1.945505	4.029643	-2.582844
H	0.856855	5.968516	-1.413413
H	-0.906693	5.483374	0.306836
H	-1.517251	3.104976	0.763056
H	1.694753	-3.113176	-1.365564
H	3.551754	-2.716213	-1.989806
H	5.985454	-2.761674	-1.929641
C	7.595900	-1.229359	-0.375106
C	6.174450	0.599157	1.235809
C	5.437672	1.525931	2.033472
C	8.280474	-0.365534	0.466328
C	7.579957	0.541501	1.266100

H	3.515614	2.298447	2.564605
H	5.981432	2.201403	2.687575
H	8.120774	1.218613	1.921460
H	9.364152	-0.390915	0.504019
H	8.138156	-1.932512	-1.000764

- **Complex: 3d**

56

	X	Y	Z
C	0.405894	3.282085	0.122233
N	0.869211	2.210708	-0.541451
C	1.815201	2.383821	-1.480590
C	2.331764	3.634096	-1.792238
C	1.862123	4.746318	-1.097242
C	0.884083	4.565155	-0.124496
Pd	0.176942	0.259233	-0.132841
C	2.012144	-0.251364	0.322902
C	2.803667	0.623113	1.052375
C	4.187442	0.394666	1.194707
C	4.816484	-0.715085	0.587528
C	4.047571	-1.613672	-0.110728
C	2.599820	-1.456549	-0.212192
O	5.009206	1.210835	1.862137
C	4.476323	2.382356	2.476738
O	4.531557	-2.705582	-0.716639
C	5.930941	-2.957011	-0.640656
C	1.858857	-2.515119	-0.755805
C	0.465205	-2.642029	-0.583357
N	-0.386528	-1.752160	-0.008241
C	-1.584587	-2.344786	0.092878
O	-1.570854	-3.587202	-0.449384
C	-0.290373	-3.846391	-0.901704
O	0.016212	-4.893602	-1.428773
C	-2.775273	-1.905369	0.747120
C	-3.980763	-2.622065	0.566020
C	-5.134680	-2.210984	1.214664
C	-5.113705	-1.093803	2.057453
C	-3.920294	-0.392054	2.254587
C	-2.758136	-0.790970	1.611945
O	-1.818331	0.790766	-0.817041
C	-2.597318	1.554204	-0.178510
C	-4.002540	1.630938	-0.835540
F	-4.888215	2.292522	-0.081216
O	-2.405357	2.212911	0.842286
F	-3.938416	2.264692	-2.024001
F	-4.506829	0.408130	-1.066440
H	2.366620	1.518506	1.476671
H	5.887501	-0.821856	0.699602
H	4.036819	3.044388	1.723996
H	5.321860	2.873317	2.953586
H	3.727291	2.116160	3.227580
H	6.492695	-2.138839	-1.101316
H	6.096540	-3.879093	-1.193547
H	6.239853	-3.084395	0.401141
H	-3.997283	-3.483989	-0.092052

H	-6.059004	-2.758652	1.062601
H	-6.021647	-0.773225	2.558007
H	-3.896674	0.474300	2.907558
H	-1.833930	-0.245426	1.774016
H	2.165715	1.488133	-1.982513
H	3.089232	3.723804	-2.561805
H	2.251351	5.735963	-1.312258
H	0.484894	5.399479	0.440057
H	-0.375876	3.089997	0.848904
H	2.368519	-3.363249	-1.196004

- **Complex: 3e**

56

	X	Y	Z
C	-2.329049	-0.530979	1.428876
C	-2.373507	-1.527539	0.432177
C	-3.655178	-1.896373	-0.026780
C	-4.802555	-1.293291	0.451385
C	-4.717234	-0.296255	1.418024
C	-3.472039	0.078643	1.911299
C	-1.183765	-2.141095	-0.068207
N	0.063021	-1.660764	-0.089087
C	0.873352	-2.656261	-0.538266
C	0.035941	-3.813582	-0.846984
O	-1.250322	-3.412200	-0.531974
C	2.274714	-2.641984	-0.628588
C	3.060935	-1.586125	-0.135914
C	2.540390	-0.298236	0.259989
C	3.358479	0.568927	0.967373
C	4.710792	0.249830	1.213656
C	5.284557	-0.948773	0.733537
C	4.486093	-1.841121	0.062267
Pd	0.755388	0.300865	-0.283253
O	-1.193244	0.900841	-1.041777
C	-1.970639	1.713819	-0.465567
O	-1.814743	2.375800	0.558363
O	5.552150	1.053647	1.864954
C	5.076840	2.300557	2.372450
O	4.911793	-3.013131	-0.422336
C	6.279095	-3.366421	-0.234946
O	0.288216	-4.914488	-1.276241
N	1.541884	2.204391	-0.716588
C	1.092021	3.300576	-0.084007
C	1.618866	4.563256	-0.335219
C	2.632681	4.696514	-1.278846
C	3.087337	3.558954	-1.942091
C	2.520777	2.330892	-1.628747
C	-3.325592	1.880582	-1.209586
F	-3.746660	0.732209	-1.763813
F	-4.298992	2.304510	-0.391855
F	-3.210776	2.784208	-2.200782
H	2.972573	1.525207	1.296991
H	6.335532	-1.125829	0.920456
H	4.731291	2.939914	1.554406
H	5.930771	2.758850	2.866197

H	4.270594	2.138938	3.092846
H	6.932132	-2.635647	-0.720931
H	6.399191	-4.341626	-0.701382
H	6.513231	-3.429212	0.831824
F	-3.788315	-2.804171	-0.992496
F	-5.993452	-1.638523	-0.034970
F	-5.818702	0.291754	1.869701
F	-3.390990	1.004050	2.862347
F	-1.161553	-0.177973	1.962386
H	2.854904	1.416347	-2.106813
H	3.870715	3.612383	-2.688738
H	3.060646	5.669489	-1.496158
H	1.229487	5.419194	0.203139
H	0.283075	3.145378	0.622019
H	2.747958	-3.553976	-0.970765

- **Complex: 3f**

58

	X	Y	Z
C	-2.469972	-0.690511	1.138196
C	-2.417620	-1.803213	0.259504
C	-3.622928	-2.478956	-0.076088
C	-4.831038	-2.030278	0.410210
C	-4.852544	-0.907697	1.250567
C	-3.677959	-0.242162	1.626267
C	-1.185522	-2.267513	-0.256103
N	0.039158	-1.712771	-0.198974
C	0.918912	-2.623453	-0.682057
C	0.157845	-3.798933	-1.129680
O	-1.153152	-3.498559	-0.837425
C	2.316920	-2.554969	-0.683311
C	3.034291	-1.518489	-0.066977
C	2.438287	-0.284037	0.384554
C	3.177176	0.561859	1.192718
C	4.524783	0.269571	1.502755
C	5.171487	-0.873797	0.984339
C	4.450157	-1.744487	0.204343
Pd	0.676084	0.284621	-0.256878
O	-1.223730	0.867853	-1.118811
C	-2.033465	1.652788	-0.545349
O	-1.908362	2.303067	0.490175
O	5.291475	1.053061	2.256809
C	4.743019	2.248136	2.816279
O	4.948377	-2.864055	-0.328425
C	6.313920	-3.186722	-0.077031
O	0.497455	-4.834983	-1.640505
N	1.459290	2.211740	-0.578291
C	0.982553	3.288169	0.067913
C	1.517686	4.557948	-0.122917
C	2.569278	4.720442	-1.019167
C	3.053684	3.603249	-1.695845
C	2.476599	2.366314	-1.443530
C	-3.376826	1.774814	-1.315288
F	-3.846618	0.573585	-1.688657
F	-4.329717	2.367532	-0.587854

F	-3.214105	2.504462	-2.437058
H	2.738386	1.482558	1.555763
H	6.214680	-1.027146	1.227126
H	4.427372	2.931574	2.022627
H	5.549542	2.695455	3.392495
H	3.900864	2.012007	3.471593
H	6.969183	-2.405384	-0.472548
H	6.495877	-4.124250	-0.597344
H	6.483010	-3.313813	0.996071
H	-3.590168	-3.338817	-0.734911
H	-5.758420	-2.523961	0.148303
N	-6.116661	-0.429709	1.748951
H	-3.727575	0.615928	2.285131
H	-1.557104	-0.184753	1.433755
H	2.833130	1.467598	-1.935360
H	3.867773	3.678896	-2.406828
H	3.003693	5.699789	-1.189911
H	1.104267	5.396978	0.424025
H	0.144294	3.111568	0.732665
H	2.842342	-3.423012	-1.063629
O	-6.122492	0.561266	2.476702
O	-7.138562	-1.033555	1.426712

- **Complex: 3g**

55

	X	Y	Z
Pd	-1.102086	-0.001000	-0.103351
F	3.295339	1.482023	1.354735
F	2.450854	3.135864	2.464082
F	2.508114	3.203981	0.305013
O	1.369834	-3.359592	0.770726
O	0.036411	-4.943163	1.705020
O	0.740970	1.133759	0.161635
O	0.203012	1.721547	2.278167
N	-0.163976	-1.823885	0.215809
N	-2.032227	1.885371	-0.178130
C	-2.799970	-0.928062	-0.406014
C	-3.767653	-0.334258	-1.221461
H	-3.522565	0.572681	-1.767458
C	-5.067383	-0.835950	-1.352779
C	-5.434637	-1.986169	-0.609694
H	-6.451299	-2.363485	-0.676780
C	-4.507750	-2.629736	0.166143
H	-4.774814	-3.534444	0.705538
C	-3.150913	-2.167259	0.246867
C	-6.073625	-0.167291	-2.240561
H	-5.660183	0.724878	-2.714315
H	-6.963495	0.119381	-1.669559
H	-6.408122	-0.854256	-3.026229
C	-2.218692	-3.012152	0.874426
C	-0.828731	-2.852059	0.772983
C	0.152728	-3.869774	1.165977
C	1.156190	-2.139839	0.202985
C	2.208124	-1.401654	-0.331183
C	3.522089	-1.831826	-0.318815

C	4.631844	-1.087433	-0.839847
C	5.919973	-1.675526	-0.819955
H	6.034649	-2.674845	-0.409089
C	7.022427	-0.997056	-1.317388
H	8.001130	-1.465282	-1.295265
C	6.872826	0.289485	-1.845238
H	7.734950	0.822827	-2.232668
C	5.608096	0.891442	-1.867735
H	5.493138	1.893361	-2.268787
C	4.501857	0.219681	-1.373947
H	3.533243	0.709617	-1.383176
C	-1.484291	2.853037	-0.930174
H	-0.622665	2.568013	-1.523710
C	-1.978986	4.151470	-0.940631
H	-1.509310	4.897935	-1.569971
C	-3.068334	4.461250	-0.130878
H	-3.473503	5.467436	-0.112600
C	-3.626000	3.459301	0.658916
H	-4.468411	3.654562	1.311712
C	-3.082205	2.182378	0.604117
H	-3.487441	1.368244	1.194903
C	0.923924	1.689942	1.287115
C	2.305209	2.395060	1.360961
H	-2.562839	-3.924113	1.352984
H	1.925731	-0.441779	-0.744756
H	3.743932	-2.804340	0.115626

- **Complex: 3h**

60

	X	Y	Z
C	2.573319	2.610115	-0.872088
N	1.482983	2.322289	-0.142450
C	0.820943	3.321409	0.462265
C	1.240353	4.643787	0.379962
C	2.372501	4.943464	-0.372678
C	3.046090	3.908285	-1.016043
Pd	0.695781	0.373531	-0.010632
C	2.458066	-0.382343	0.412166
C	3.335707	0.358241	1.193894
C	4.673643	-0.047403	1.363060
C	5.176398	-1.202033	0.727699
C	4.318200	-1.974134	-0.019362
C	2.907376	-1.634841	-0.145669
O	5.568498	0.630957	2.091645
C	2.057369	-2.594243	-0.717058
C	0.658898	-2.535968	-0.608223
N	-0.079028	-1.535521	-0.087665
C	-1.364375	-1.948344	-0.030911
O	-1.495504	-3.203823	-0.543714
C	-0.248171	-3.639925	-0.936733
O	-0.061643	-4.727191	-1.428194
C	-2.458331	-1.284355	0.526480
C	-3.729629	-1.818347	0.546989
C	-4.885616	-1.208693	1.147342
C	-6.130120	-1.874367	1.055981

C	-7.275700	-1.332502	1.622162
C	-7.211903	-0.110766	2.298277
C	-5.988262	0.561971	2.401939
C	-4.840800	0.026059	1.838247
O	-1.208926	1.314496	-0.467179
C	-1.747016	1.037483	-1.582653
C	-3.116259	1.749004	-1.753807
F	-2.967052	3.088040	-1.746170
O	-1.350462	0.319207	-2.493612
F	-3.952284	1.439416	-0.746736
F	-3.729332	1.423394	-2.896082
H	3.000898	1.280329	1.651730
H	6.221959	-1.447383	0.860323
O	4.683789	-3.098949	-0.650650
H	-6.180213	-2.823948	0.530014
H	-8.220816	-1.859413	1.538268
H	-8.106313	0.315175	2.741481
H	-5.935688	1.510920	2.926273
H	-3.903678	0.566057	1.926567
H	-0.070030	3.039994	1.012469
H	0.680105	5.415780	0.893825
H	2.721510	5.966911	-0.460524
H	3.925041	4.093734	-1.621895
H	3.072083	1.770138	-1.342927
H	2.474799	-3.500249	-1.139065
H	-2.237131	-0.307701	0.942633
H	-3.883844	-2.785705	0.073329
C	6.037152	-3.526623	-0.543308
C	5.158007	1.823961	2.755495
H	6.294060	-3.724729	0.501625
H	6.104400	-4.444639	-1.123157
H	6.712266	-2.772650	-0.959030
H	6.040353	2.187750	3.277650
H	4.820141	2.571032	2.030569
H	4.361459	1.610212	3.473709

- **Complex: 3i**

63

	X	Y	Z
C	8.736701	-1.405677	0.408155
C	8.525207	-0.299851	1.239162
C	7.242673	0.215691	1.425709
C	6.178508	-0.396686	0.764118
C	6.388929	-1.511145	-0.068459
C	7.668280	-2.019923	-0.252037
C	5.074126	-1.982107	-0.644359
C	4.092694	-0.989131	-0.073427
C	4.744550	-0.086480	0.753637
C	2.678117	-0.948335	-0.248168
C	1.952171	0.150749	0.366748
C	2.645152	1.010553	1.230145
C	4.018707	0.905482	1.441994
Pd	0.110530	0.561488	-0.123811
O	-1.862679	1.089619	-0.916165
C	-2.820890	1.468306	-0.188656

O	-2.889415	1.554746	1.039461
C	2.049017	-2.013868	-0.920340
C	0.668594	-2.257307	-0.858713
N	-0.264523	-1.461902	-0.304801
C	-1.439116	-2.131187	-0.298521
O	-1.305997	-3.350957	-0.885705
C	0.011310	-3.500556	-1.274041
C	-2.646428	-1.724138	0.268482
C	-3.800686	-2.478031	0.200474
C	-5.069281	-2.111595	0.765050
O	0.421455	-4.494311	-1.822510
N	0.528232	2.624417	-0.145017
C	-0.253979	3.461885	0.555739
C	-0.067535	4.839001	0.531287
C	0.953524	5.366237	-0.254288
C	1.756540	4.495729	-0.986740
C	1.515656	3.130553	-0.901483
C	-4.087762	1.819438	-1.013556
F	-4.573477	0.717456	-1.614292
F	-5.068406	2.328735	-0.260361
F	-3.815293	2.713540	-1.978652
H	4.519971	1.597179	2.113461
H	2.106697	1.808145	1.731860
H	2.124453	2.414079	-1.442396
H	2.561025	4.859117	-1.614946
H	1.120750	6.437316	-0.295761
H	-0.717715	5.476145	1.118930
H	-1.044396	3.003481	1.142358
H	7.839148	-2.879317	-0.894022
H	9.742637	-1.791312	0.275782
H	9.368914	0.161059	1.742845
H	7.082372	1.074841	2.070530
H	4.843818	-3.008296	-0.329368
H	5.077463	-1.979004	-1.741765
H	2.651071	-2.786210	-1.388063
C	-6.158843	-3.003230	0.619884
C	-7.406496	-2.695452	1.141668
C	-7.601774	-1.488070	1.819852
C	-6.536337	-0.591734	1.970559
C	-5.285382	-0.892591	1.455373
H	-6.005659	-3.939353	0.090066
H	-8.230282	-3.391420	1.021365
H	-8.578099	-1.244574	2.226581
H	-6.688879	0.347953	2.491770
H	-4.476430	-0.177680	1.576663
H	-3.759951	-3.426612	-0.331062
H	-2.615275	-0.771130	0.786797

- **Complex: 3j**

60

	X	Y	Z
C	4.888390	-0.452957	-1.813842
C	4.817809	-1.623864	-1.018323
C	5.994151	-2.396381	-0.861169
C	7.183164	-2.017871	-1.466343

C	7.232383	-0.858446	-2.246818
C	6.079244	-0.081251	-2.415645
C	3.613774	-2.063723	-0.375111
C	2.393193	-1.416140	-0.424506
C	1.247000	-1.916904	0.189386
N	0.002082	-1.382610	0.183148
C	-0.824510	-2.241893	0.803955
C	-0.024487	-3.382861	1.266548
O	1.259960	-3.110845	0.841180
C	-2.222055	-2.158243	0.897809
C	-2.985154	-1.196037	0.208945
C	-2.407820	-0.050962	-0.465255
C	-3.234985	0.672027	-1.321088
C	-4.594767	0.384642	-1.468986
C	-5.190365	-0.655414	-0.731125
C	-4.393605	-1.435488	0.099471
Pd	-0.593072	0.575113	-0.099348
O	1.395706	1.376077	0.283796
C	1.902964	1.154518	1.425982
O	1.447045	0.543613	2.386162
H	-5.193430	0.993780	-2.136705
O	-4.939307	-2.458800	0.813966
C	-5.300949	-3.604690	0.032428
O	-0.312273	-4.384106	1.874306
N	-1.195901	2.593134	-0.148987
C	-2.261457	3.042313	0.534160
C	-2.595913	4.389624	0.581827
C	-1.805911	5.302819	-0.111311
C	-0.700858	4.835719	-0.817537
C	-0.422807	3.474481	-0.803651
C	3.325765	1.762355	1.550651
F	3.866156	1.573590	2.758160
F	3.312265	3.088178	1.319161
F	4.158380	1.212166	0.646658
H	-2.835262	1.514334	-1.876844
O	-6.507404	-0.969232	-0.776218
H	5.954049	-3.296603	-0.254129
H	8.074537	-2.622111	-1.332913
H	8.162036	-0.560314	-2.720859
H	6.118047	0.819350	-3.019903
H	4.007829	0.166284	-1.949831
H	0.442722	3.065596	-1.312618
H	-0.053682	5.508099	-1.367895
H	-2.045107	6.360903	-0.097639
H	-3.460115	4.706145	1.153533
H	-2.854076	2.294119	1.048845
H	-2.726917	-2.966425	1.414840
H	2.261332	-0.473440	-0.943874
H	3.676514	-2.987813	0.195749
H	-5.709916	-4.333993	0.730919
H	-6.052307	-3.338721	-0.714815
H	-4.411300	-4.017608	-0.455593
C	-7.342848	-0.214490	-1.643627
H	-8.341446	-0.632779	-1.531078
H	-7.354070	0.841653	-1.356019

H -7.018498 -0.313663 -2.684591

- **Complex: 3k**

52

	X	Y	Z
C	-1.313861	3.080183	-0.644137
N	-1.912682	2.032866	-0.055517
C	-3.038969	2.234619	0.646842
C	-3.612157	3.492471	0.779896
C	-3.002168	4.578321	0.157824
C	-1.832213	4.366742	-0.565917
Pd	-1.010659	0.142884	-0.194462
O	0.853285	1.193888	0.090244
C	1.228442	1.346847	1.296299
C	2.530877	2.185163	1.387203
F	3.535392	1.577658	0.729198
N	-0.125048	-1.713457	0.086384
C	1.196013	-2.020260	0.195497
O	1.377799	-3.140076	0.944868
C	0.137197	-3.599690	1.344667
C	-0.821054	-2.649499	0.758060
C	2.272034	-1.365647	-0.394432
C	3.583632	-1.785488	-0.228692
C	4.731792	-1.166338	-0.813274
C	4.652672	-0.044307	-1.679342
C	5.801688	0.510969	-2.214796
C	7.058756	-0.027717	-1.907994
C	7.158733	-1.134143	-1.056859
C	6.013495	-1.697373	-0.517990
C	-2.212320	-2.779843	0.851168
C	-3.132032	-1.961601	0.162624
C	-2.737675	-0.743801	-0.498492
C	-3.700884	-0.171186	-1.324950
C	-5.006086	-0.621959	-1.458551
C	-5.367997	-1.751408	-0.715663
C	-4.481428	-2.428092	0.075148
O	-0.013108	-4.603288	2.028439
H	-5.713546	-0.119984	-2.108483
H	-4.788330	-3.329709	0.593994
O	0.722856	0.931209	2.335956
F	2.934744	2.379046	2.645722
F	2.364903	3.397281	0.826051
F	-3.372252	0.909240	-2.070482
F	-6.636276	-2.191672	-0.823723
H	6.085981	-2.556157	0.143500
H	8.131749	-1.550905	-0.818935
H	7.955000	0.414438	-2.331231
H	5.728190	1.369448	-2.874257
H	3.688339	0.389540	-1.922083
H	-0.394499	2.868532	-1.177991
H	-1.318515	5.180043	-1.064437
H	-3.428548	5.572452	0.239764
H	-4.518687	3.607204	1.362056
H	-3.481622	1.361188	1.113003
H	-2.575168	-3.647069	1.394458

H	2.016072	-0.492242	-0.981797
H	3.763859	-2.654927	0.399823

- **Complex: 3m**

60

	X	Y	Z
C	-4.989380	-0.194588	1.507799
C	-4.987974	-1.499615	0.960979
C	-6.200812	-2.225038	0.968783
C	-7.360103	-1.676879	1.501223
C	-7.341766	-0.387289	2.039521
C	-6.150573	0.347912	2.037282
C	-3.812097	-2.116212	0.400631
C	-2.560787	-1.545012	0.359972
C	-1.446370	-2.179889	-0.195721
N	-0.173955	-1.734194	-0.260433
C	0.580668	-2.705508	-0.818853
C	-0.303657	-3.824996	-1.153935
O	-1.552223	-3.431428	-0.727632
C	1.971454	-2.726177	-0.969313
C	2.836352	-1.776216	-0.390162
C	2.369794	-0.567359	0.254094
C	3.286510	0.151484	1.023843
C	4.635870	-0.202094	1.096771
C	5.122718	-1.333046	0.351659
C	4.232701	-2.090158	-0.366074
Pd	0.578856	0.177407	-0.016840
O	-1.361074	1.126854	-0.256147
C	-1.618288	1.630999	-1.391741
O	-0.947957	1.645197	-2.417840
O	5.558712	0.455058	1.796825
C	5.158653	1.594751	2.554569
H	4.564553	-2.976389	-0.894304
O	-0.097527	-4.896396	-1.674149
N	1.312402	2.150256	0.010585
C	2.304443	2.547452	-0.802697
C	2.726443	3.869365	-0.863409
C	2.104719	4.810892	-0.047205
C	1.074499	4.397617	0.793103
C	0.701307	3.059010	0.787130
C	-3.008851	2.320318	-1.409839
F	-3.303856	2.861475	-2.595973
F	-3.068784	3.306464	-0.493562
F	-3.984717	1.444435	-1.111907
H	2.956616	1.032252	1.560206
O	6.444936	-1.552476	0.452975
H	-6.216311	-3.227611	0.549761
H	-8.280495	-2.252036	1.497357
H	-8.247058	0.043951	2.454558
H	-6.134044	1.352173	2.448969
H	-4.080169	0.398190	1.502797
H	-0.110500	2.692082	1.405533
H	0.558083	5.094223	1.442664
H	2.415071	5.850169	-0.069112
H	3.525720	4.145549	-1.540746

H	2.763759	1.777106	-1.412447
H	2.390608	-3.609101	-1.441478
H	-2.371910	-0.550810	0.746518
H	-3.939662	-3.111540	-0.019835
H	6.062736	1.956704	3.038989
H	4.751362	2.368786	1.896361
H	4.417150	1.312697	3.307506
C	6.981149	-2.657191	-0.264238
H	8.051307	-2.648498	-0.067961
H	6.545846	-3.596332	0.091756
H	6.798394	-2.545762	-1.337809

- **Complex: 4d**

60

	X	Y	Z
C	-2.885524	-0.239997	1.809547
C	-3.098369	-1.351042	0.962467
C	-4.401625	-1.887936	0.843119
C	-5.455547	-1.309279	1.533071
C	-5.237972	-0.202398	2.361596
C	-3.948343	0.320542	2.500822
C	-2.013053	-1.965833	0.267755
N	-0.757723	-1.531076	0.084718
C	-0.050470	-2.532831	-0.501183
C	-0.968667	-3.640635	-0.748127
O	-2.182658	-3.211276	-0.240255
C	1.338497	-2.579863	-0.727334
C	2.222154	-1.611183	-0.226088
C	1.811649	-0.333100	0.307919
C	2.735457	0.439119	0.991300
C	4.084699	0.034118	1.096970
C	4.544501	-1.154592	0.491147
C	3.641870	-1.953903	-0.167090
Pd	0.018625	0.385708	-0.101539
N	-1.935196	1.164906	-0.795779
O	5.021685	0.743915	1.728043
C	4.668778	1.982645	2.342317
O	3.958980	-3.105240	-0.768075
C	5.315683	-3.539672	-0.730014
O	-0.822954	-4.727055	-1.259562
N	0.855435	2.280412	-0.448029
C	0.833104	3.241184	0.490147
C	1.376023	4.501147	0.275208
C	1.963839	4.775587	-0.957324
C	1.985487	3.779051	-1.928923
C	1.420583	2.543487	-1.636345
H	2.437702	1.384325	1.425990
H	5.595370	-1.399623	0.573172
H	4.295846	2.688346	1.593679
H	5.587706	2.361784	2.783649
H	3.918056	1.825873	3.121798
H	5.964469	-2.809447	-1.222379
H	5.339076	-4.483133	-1.270592
H	5.637223	-3.691802	0.304340
H	-4.569028	-2.746789	0.202165

H	-6.453979	-1.721044	1.429272
H	-6.066690	0.243888	2.901283
H	-3.773881	1.168721	3.155160
H	-1.882561	0.157490	1.934112
H	1.419798	1.735736	-2.360020
H	2.433253	3.945204	-2.901331
H	2.399328	5.749016	-1.155688
H	1.335886	5.243583	1.063285
H	0.377762	2.979693	1.441086
H	1.726239	-3.489901	-1.167829
C	-2.557030	2.201678	-0.219212
C	-3.808103	2.647380	-0.629663
C	-4.439739	1.986200	-1.679165
C	-3.792543	0.911695	-2.285421
C	-2.541110	0.535235	-1.813265
H	-2.040508	2.678139	0.609034
H	-4.273400	3.486224	-0.125692
H	-5.420182	2.301786	-2.019854
H	-4.244474	0.367930	-3.106433
H	-2.003163	-0.301843	-2.250358

- **Complex: 4h**

64

	X	Y	Z
C	-5.070151	-0.406759	-1.376700
C	-5.118919	0.922961	-0.892389
C	-6.381394	1.556646	-0.817892
C	-7.538298	0.895620	-1.206201
C	-7.469877	-0.417955	-1.679413
C	-6.229509	-1.062239	-1.761833
C	-3.953697	1.651243	-0.468588
C	-2.662974	1.164851	-0.462502
C	-1.566558	1.904087	-0.018709
N	-0.273923	1.513325	0.081768
C	0.454454	2.584414	0.462162
C	-0.467200	3.707334	0.668676
O	-1.711575	3.208644	0.347052
C	1.849889	2.683659	0.541681
C	2.718743	1.689641	0.065807
C	2.301866	0.376929	-0.364892
C	3.207981	-0.417148	-1.051222
C	4.540803	-0.000137	-1.250152
C	5.007895	1.230041	-0.746596
C	4.122421	2.053273	-0.091980
Pd	0.551805	-0.380195	0.172073
N	-1.322945	-1.239184	1.000749
O	5.457599	-0.738941	-1.880945
C	5.095435	-2.026386	-2.377401
O	4.452148	3.245317	0.419550
C	5.796921	3.695474	0.282813
O	-0.291705	4.845373	1.027378
N	1.422731	-2.289891	0.358544
C	2.149035	-2.589799	1.446059
C	2.751029	-3.831106	1.615289
C	2.595023	-4.794826	0.623196

C	1.838265	-4.483501	-0.504404
C	1.269966	-3.220106	-0.598918
H	2.910600	-1.389143	-1.419474
H	6.048347	1.486316	-0.896970
H	-6.436038	2.577542	-0.449778
H	-8.496330	1.401200	-1.141449
H	-8.373591	-0.936453	-1.982782
H	-6.172961	-2.081710	-2.130374
H	-4.120053	-0.926576	-1.450159
H	0.691897	-2.925726	-1.470091
H	1.690040	-5.199276	-1.304174
H	3.056405	-5.771164	0.725167
H	3.331770	-4.026414	2.508803
H	2.252237	-1.805479	2.187786
H	2.249342	3.639140	0.859037
H	-2.433991	0.160530	-0.799869
H	-4.117885	2.665045	-0.109832
H	6.062690	3.787935	-0.774188
H	5.831735	4.671212	0.762232
H	6.483519	3.007365	0.784762
H	5.998055	-2.429042	-2.831604
H	4.768692	-2.675272	-1.558536
H	4.306223	-1.941597	-3.129550
C	-1.864166	-0.653495	2.079182
C	-3.089850	-1.043545	2.605998
C	-3.783390	-2.081026	1.988301
C	-3.216248	-2.701559	0.877925
C	-1.983431	-2.251453	0.420081
H	-1.298447	0.164800	2.515848
H	-3.489015	-0.533570	3.474686
H	-4.749173	-2.400297	2.365402
H	-3.718243	-3.513318	0.364426
H	-1.516006	-2.700780	-0.450675

- **Complex: 5h**

54

	X	Y	Z
C	5.348717	-1.051187	0.683814
C	5.461378	0.352061	0.531913
C	6.736675	0.939507	0.698944
C	7.844319	0.166324	1.018572
C	7.712314	-1.216597	1.174164
C	6.459858	-1.817734	1.001537
C	4.341810	1.195846	0.206058
C	3.034815	0.770536	0.114348
C	1.976918	1.607960	-0.239122
N	0.660892	1.306297	-0.309250
C	-0.009574	2.445236	-0.578967
C	0.971725	3.520583	-0.745073
O	2.191490	2.927014	-0.507537
C	-1.399116	2.646646	-0.564200
C	-2.297889	1.701232	-0.049369
C	-1.933480	0.353545	0.310846
C	-2.811336	-0.392872	1.086932
C	-4.080572	0.109423	1.431067

C	-4.517121	1.373749	0.982165
C	-3.651535	2.149733	0.248406
Pd	-0.317563	-0.520721	-0.412684
Cl	1.603113	-1.769848	-1.450016
O	-4.974206	-0.569724	2.161224
C	-4.636427	-1.872636	2.630977
O	-3.952529	3.371676	-0.214589
C	-5.241694	3.903776	0.069225
O	0.855902	4.692805	-1.015097
N	-1.400289	-2.311977	-0.635476
C	-2.523752	-2.357071	-1.368791
C	-3.242860	-3.531466	-1.552398
C	-2.790214	-4.697574	-0.940835
C	-1.625997	-4.648421	-0.179034
C	-0.952744	-3.438278	-0.059469
H	-2.537634	-1.391217	1.402262
H	-5.517367	1.694568	1.242100
H	6.840449	2.014435	0.577736
H	8.813514	0.637971	1.145769
H	8.577776	-1.823051	1.421109
H	6.356411	-2.892833	1.109371
H	4.390766	-1.539564	0.532881
H	-0.028394	-3.354753	0.500777
H	-1.232370	-5.530051	0.312618
H	-3.333743	-5.629122	-1.058426
H	-4.139133	-3.520752	-2.161168
H	-2.848853	-1.420725	-1.809044
H	-1.747881	3.642030	-0.810565
H	2.753149	-0.258747	0.298974
H	4.559092	2.245477	0.019214
H	-5.389021	3.994761	1.149619
H	-5.264269	4.888373	-0.392993
H	-6.022314	3.270737	-0.363442
H	-5.504092	-2.217214	3.189557
H	-4.442952	-2.546743	1.790424
H	-3.762923	-1.831339	3.287795

- **Complex: 6h**

96

	X	Y	Z
O	-0.371078	-5.216665	-0.756929
C	0.040718	-4.083715	-0.837709
O	1.385152	-3.787797	-0.945893
C	1.510749	-2.431317	-1.005874
C	2.756944	-1.804148	-1.080659
C	3.961072	-2.469786	-1.099382
C	5.250665	-1.829878	-1.085703
C	5.415568	-0.442390	-0.854688
C	6.682179	0.123213	-0.848330
C	7.816327	-0.666666	-1.069201
C	7.672255	-2.040199	-1.286098
C	6.408848	-2.615060	-1.287512
N	0.309834	-1.826972	-0.958860
C	-0.633845	-2.780918	-0.848405
C	-2.026399	-2.592775	-0.829315

C	-2.632611	-1.352659	-1.105087
C	-4.036950	-1.371036	-1.472667
O	-4.677952	-2.512430	-1.190909
C	-6.067042	-2.606250	-1.485043
C	-4.637066	-0.301696	-2.114466
C	-3.869511	0.856149	-2.319681
O	-4.337551	1.947719	-2.941044
C	-5.664286	1.935335	-3.458082
C	-2.559186	0.968003	-1.805960
C	-1.936278	-0.082159	-1.163088
Pd	-0.206054	0.089671	-0.235250
C	-0.788859	1.823626	0.617498
N	-0.087247	2.980344	0.668289
C	1.235078	3.171255	0.130678
C	2.309572	3.156588	1.026575
C	2.096883	2.938647	2.500691
C	3.592427	3.327442	0.503845
C	3.810754	3.503522	-0.863904
C	5.202906	3.671548	-1.418163
C	2.707683	3.513692	-1.721219
C	1.405548	3.348068	-1.245805
C	0.241526	3.332862	-2.200280
C	-0.747838	3.932218	1.430740
C	-1.886829	3.350055	1.876261
N	-1.893966	2.059111	1.370154
C	-2.894921	1.066355	1.653174
C	-4.173001	1.204819	1.105154
C	-4.590278	2.440411	0.350623
C	-5.079758	0.159975	1.309929
C	-4.739916	-0.978832	2.039546
C	-5.702259	-2.130737	2.172906
C	-3.477030	-1.034379	2.639226
C	-2.540743	-0.014609	2.473591
C	-1.221494	-0.041869	3.202670
O	1.700511	0.244308	0.829432
C	2.497263	-0.358413	1.596443
C	1.976883	-1.646367	2.310121
F	2.833581	-2.668965	2.161385
F	0.778713	-2.076200	1.876953
F	1.853195	-1.422347	3.634269
O	3.650930	-0.058820	1.900173
H	2.710423	-0.719876	-1.100026
H	3.950090	-3.557955	-1.118770
H	4.552095	0.180862	-0.637638
H	6.793327	1.185448	-0.653693
H	8.803688	-0.216178	-1.060378
H	8.548148	-2.660087	-1.449418
H	6.297146	-3.683412	-1.452434
H	-2.632603	-3.488708	-0.770613
H	-6.239830	-2.531215	-2.562831
H	-6.380058	-3.585282	-1.127359
H	-6.621408	-1.821693	-0.958753
H	-5.667841	-0.353315	-2.435603
H	-6.393204	1.803577	-2.651735
H	-5.808176	2.906272	-3.927389

H	-5.780903	1.141792	-4.202048
H	-2.088702	1.940149	-1.899314
H	1.703440	3.838067	2.986721
H	3.039688	2.672502	2.980982
H	1.383460	2.126053	2.670283
H	4.441449	3.299477	1.182507
H	5.944813	3.717510	-0.617742
H	5.460859	2.835047	-2.075860
H	5.280177	4.587780	-2.010983
H	2.861825	3.654662	-2.789076
H	0.488589	3.877056	-3.114229
H	-0.005983	2.302297	-2.479140
H	-0.651127	3.784353	-1.757371
H	-0.341875	4.919949	1.582508
H	-2.681715	3.721422	2.503450
H	-3.775621	2.851020	-0.250891
H	-4.918347	3.221970	1.045440
H	-5.429776	2.215805	-0.312321
H	-6.072617	0.238921	0.870767
H	-5.659390	-2.570584	3.172692
H	-6.730101	-1.815606	1.975770
H	-5.446282	-2.917673	1.454298
H	-3.215476	-1.891762	3.255209
H	-1.117405	0.846441	3.835913
H	-1.154045	-0.927874	3.836359
H	-0.372589	-0.049920	2.514909

10.4.- Table S8: Summary Table for wB97XD

	Ground State		Absorption			Emission			
**Compound**	**HOMO**	**LUMO**	**T_1**	**S_1**	**Exp**	**S_1**	**Exp**	**T_1 vs S_0**	**ϕ**
**3a**	-0.28816 (12%)	-0.05122 (2%)	667	396 (0.286)	468	495 (0.583)	542	Cross	5%
**3b**	-0.28584 (9%)	-0.05267 (2%)	753	424 (0.512)	485	548 (0.730)	550	Cross	4%
**3c**	-0.27222 (2%)	-0.05802 (2%)	831	446 (1.122)	516	588 (1.477)	577	Cross	<1%
**3d**	-0.28325 (7%)	-0.04311 (3%)	687	406 (0.699)	450	510 (0.894)	539	Cross	7%
**3e**	-0.28984 (4%)	-0.04964 (6%)	691	410 (0.653)	457	502 (0.829)	564	Cross	5%
**3f**	-0.28765 (4%)	-0.06096 (2%)	700	419 (0.721)	474	560 (0.959)	630	Cross	4%
**3g**	-0.28785 (11%)	-0.05317 (7%)	749	405 (1.010)	445	536 (1.287)	534	Cross	<1%
**3h**	-0.27671 (7%)	-0.04916 (9%)	775	429 (1.077)	475	559 (1.337)	570	Cross	18 %
**3i**	-0.28593 (17%)	-0.05639 (2%)	749	412 (0.721)	474	536 (1.158)	574	Cross	3%
**3j**	-0.28875 (19%)	-0.05459 (5%)	743	406 (0.744)	424	529 (1.221)	533	Cross	<1%
**3k**	-0.29884 (22%)	-0.06015 (19%)	737	396 (0.921)	445	560 (0.959)	550	Cross	<1%
**3m**	-0.27486 (11%)	-0.05143 (4%)	792	437 (0.838)	486	562 (1.137)	605	Cross	<1%
**4d**	-0.29381 (4%)	-0.05378 (6%)	685	407 (0.667)	471	513 (0.877)	553	Cross	10%
**4h**	-0.28836 (6%)	-0.06004 (6%)	752	429 (0.972)	488	563 (1.288)	590	Cross	28%
**5h**	-0.25511 (14%)	-0.08000 (18%)	770	429 (1.000)	481	563 (1.244)	593	Cross	12%
**6h**	-0.27289 (7%)	-0.04388 (2%)	772	429 (0.903)	483	555 (1.224)	593	Cross	15%

10.5.- T_1 *vs* S_0 relative energies

During the geometry optimizations of the T1 state, we observed in the TDDFT calculations the mentioned T1/S0 state crossing. Nevertheless, it is well known that TDDFT fails when two electronic states are near-degenerate. This near-degeracy does not imply, indeed, that the inverse ISC takes place. TDDFT calculations do not support this, they cannot due to the methodological formulation. In order to study in detail the triplet evolution other more sophisticated methods such as multiconfigurational methods should be needed, which go beyond the scope of this work.

In order to clarify this, further standard DFT calculations have been performed to calculate the S0/T1 energy differences at S0, S1 and T1 geometries. S0 and T1 optimal geometries were optimized with standard DFT calculations, but the S1 ones were taken from the TDDFT optimizations. Notice that with standard DFT only the lowest-lying electronic states of each spin may be optimized. The calculated results are summarized in Table S9.

Table S9 Energy differences between S0 and T1 electronic states calculated at different geometries by standard DFT methodology.

		S0 (eV)	T1 (eV)
3d	Geometry S0	0.00	2.24
	Geometry S1	0.24	1.92
	Geometry T1	0.35	1.89
3h	Geometry S0	0.00	2.99
	Geometry S1	0.27	1.73
	Geometry T1	0.55	1.67

According to these values, T1 state lies above S0, but the energy difference in the T1 geometry has decreased to values in the IR region. Of course, these results and the TDDFT ones should be taken with caution, since multireference calculations should be carried out for quantitatively accurate results. Nevertheless, both approaches suggest that the evolution from T1 to S0 would take place via a non-radiative process, supporting the observed fluorescent quantum yields.

11.- Crystallographic Results

Table S10: Crystal data of compound 1a

Empirical formula	$C_{23}H_{15}NO_2$
Formula Weight	337.36
Temperature	100(2) K
Wavelength	0.71073 Å
Crystal system, space group	monoclinic, *P2₁/c*
Unit cell dimensions	a= 17.6603(11) Å<br>b= 11.6297(8) Å β = 94.211(3)°<br>c= 7.8516(5)
Volume	1608.24(18) $Å^3$
Z	4
Absorption coefficient	0.089 mm^{-1}
F(000)	704
Crystal Size	0.036 x 0.100 x 0.130 mm
Absorption correction	Multi-scan
T_{min} , T_{max}	0.9052, 0.9991
θ_{min},θ_{max}	2.313, 30.652
Limiting indices	-25≤h≤25, -16≤k≤16, -11≤l≤11
Reflections collected / unique	72380 / 4578 [R(int) = 0.0889]
Completeness to θ_{max}	99.1% (99.9 % up to θ =25.242°)
Refinement method	Full-matrix least-squares on F^2
Data / restraints / parameters	4926 /0 / 235
Goodness-of-fit on F^2	1.284
Final R indices [I>2sigma(I)]	R1=0.1052; wR2=0.1992 [4578 refl.]
R indices (all data)	R1=0.1128; wR2=0.2026
Largest diff. peak and hole	0.555 / -0.461

Table S11: Crystal data of compound 1e

Empirical formula	$C_{18}H_{10}F_5NO_4$
Formula Weight	399.27
Temperature	100(2) K
Wavelength	0.71073 Å
Crystal system, space group	monoclinic, *P2₁/n*
Unit cell dimensions	a= 8.6498(2) Å<br>b= 9.5740(2) Å β=93.8360(10)°<br>c= 19.1379(4)
Volume	1571.32(6) Å³
Z	4
Absorption coefficient	0.157 mm^{-1}
F(000)	808
Crystal Size	0.050 x 0.090 x 0.140 mm
Absorption correction	Multi-scan
T_{min} , T_{max}	0.8349, 0.9604
θ_{min},θ_{max}	2.380, 30.552
Limiting indices	-12≤h≤12, -13≤k≤13, -27≤l≤21
Reflections collected / unique	46299 / 4710 [R(int) = 0.0332]
Completeness to θ_{max}	96.9% (97.0 % up to θ =25.5°)
Refinement method	Full-matrix least-squares on F^2
Data / restraints / parameters	5860 /0 / 242
Goodness-of-fit on F^2	1.078
Final R indices [I>2sigma(I)]	R1=0.0565; wR2=0.1313 [4377 refl.]
R indices (all data)	R1=0.0613; wR2=0.1337
Largest diff. peak and hole	0.587 / -0.313

Discussion of the X-ray crystal structures of oxazolones 1a and 1e (Figures S1 and S2)

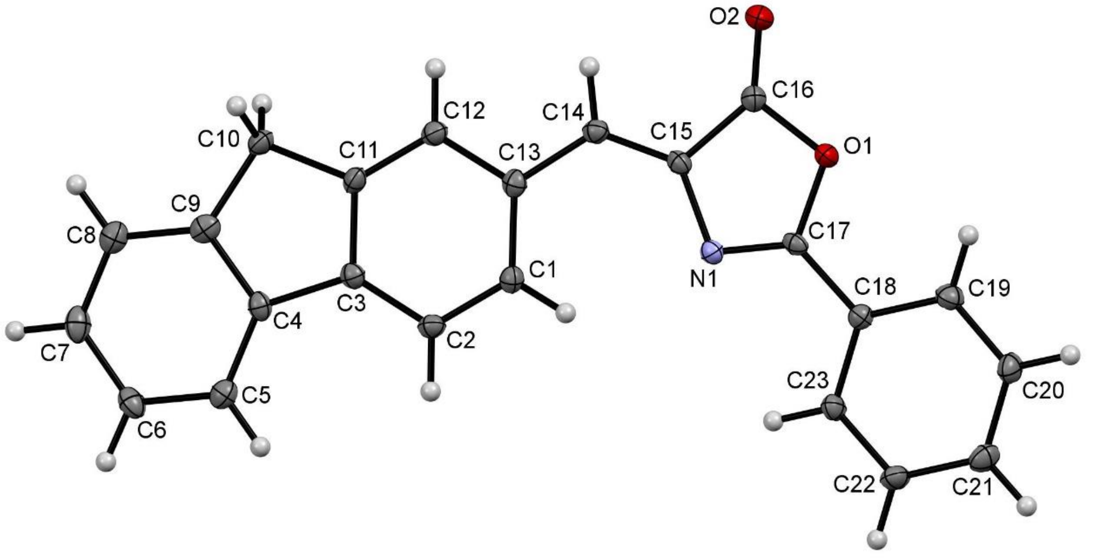

Figure S1. Molecular draw of oxazolone **1a**. Thermal ellipsoids are drawn at 50% probability level

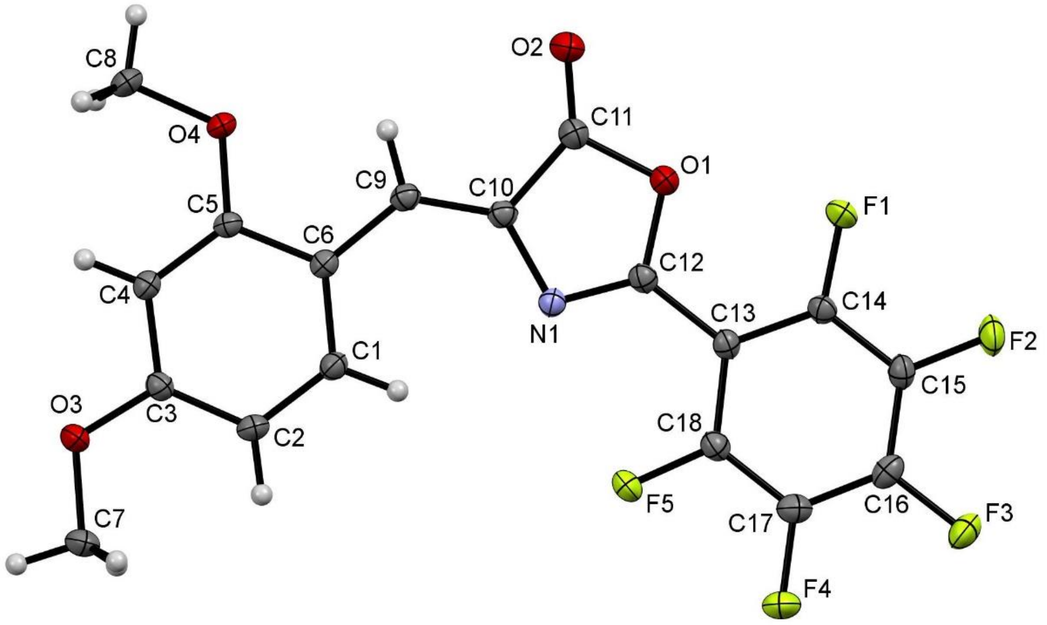

Figure S2. Molecular draw of oxazolone **1e**. Thermal ellipsoids are drawn at 50% probability level

Both structures show that the exocyclic C=C bond of the oxazolone adopts the *Z*-configuration, as is usual for this type of compounds due to its higher thermodynamic stability with respect to the *E*-isomer. The molecular structure of **1a** is essentially planar, showing values of the torsion angles C1-C13-C14-C15 (0.72(2)°), C13-C14-C15-N1 (-0.28(2)°), N1-C17-C18-C23 (1.30(2)°), and O1-C17-C18-C19 (-0.16(2)°), close to zero in all measured cases. This planarity has also been observed in a very closely related fluorenylidene-oxazolone (BMO-PF), having a methyl group instead of the

phenyl unit at the 2-position of the heterocycle.[20] The internal bond distances and angles of **1a** are identical, within experimental error, to those found in its methyl counterpart BMO-PF. The only minor difference found between the two structures is the orientation of the fluorenyl group, because in **1a** the methylene C10 points to the same side than the carbonyl, while in BMO-PF the methylene points to the same side than the nitrogen. The molecular structure of **1e** does not show the same planarity than **1a**, the position of the C_6F_5 ring showing the larger deviations as deduced from the values of the dihedral angles O1-C12-C13-C14 (-5.39(2)°), C12-C13-C14-F1 (6.30(2)°), and N1-C12-C13-C18 (-7.04(2)°). These deviations can be probably due to the minimization of intramolecular interactions between the ortho-fluorine atoms F5 and F1 and the lone pairs located at the nitrogen N1 and oxygen O1 atoms of the heterocycle, respectively. In addition, the methoxyde O4-C8 in 2-position adopts a *syn* orientation (towards the vinyl proton instead towards N1) probably by the same reason. In fact, a quick inspection in the literature of X-ray structures of oxazolones having *ortho*-methoxydes as substituents in the arylidene ring shows a clear conformational preference for this arrangement.[21,22] As a result, the intramolecular distance O4-H9 is only of 2.270(2) Å, clearly shorter than the sum of the van der Waals radii (2.72 Å).[23]

Table S12: Crystal data of compound 3c

Empirical formula	$C_{33}H_{19}F_3N_2O_4$ Pd
Formula Weight	670.90
Temperature	100(2) K
Wavelength	0.71073 Å
Crystal system, space group	triclinic, $P\overline{1}$
Unit cell dimensions	a= 13.2828(8) Å α = 70.776(2)°<br>b= 13.3193(7) Å β=88.908(2)°<br>c= 16.0248(9) γ =86.898(2)°
Volume	2673.1(3) Å^3
Z	4
Absorption coefficient	0.759 mm^{-1}
F(000)	1344
Crystal Size	0.026 x 0.035 x 0.060 mm
Absorption correction	Multi-scan
T_{min} , T_{max}	0.6758, 0.7457
θ_{min},θ_{max}	2.041, 28.333
Limiting indices	-17≤h≤17, -17≤k≤17, -21≤l≤21
Reflections collected / unique	131915 / 13288 [R(int) = 0.1139]
Completeness to θ_{max}	99.6% (99.9 % up to θ =25.5°)
Refinement method	Full-matrix least-squares on F^2
Data / restraints / parameters	13288 / 73 / 787
Goodness-of-fit on F^2	1.138
Final R indices [I>2sigma(I)]	R1=0.0775; wR2=0.1382 [11896 refl.]
R indices (all data)	R1=0.0916; wR2=0.1465
Largest diff. peak and hole	1.253 / -1.561

Table S13: Crystal data of compound 3d

Empirical formula	$C_{25}H_{19}F_3N_2O_6Pd{\cdot}2(CH_2Cl_2)$
Formula Weight	776.67
Temperature	100(2) K
Wavelength	0.71073 Å
Crystal system, space group	triclinic, $P\bar{1}$
Unit cell dimensions	a= 14.8067(7) Å α = 67.8450(10)°<br>b= 14.9247(7) Å β = 70.2500(10)°<br>c= 16.1001(8) Å γ = 69.1310(10)°
Volume	2992.6(3) $Å^3$
Z	4
Absorption coefficient	1.041 mm^{-1}
F(000)	1552
Crystal Size	0.096 x 0.154 x 0.220 mm
Absorption correction	Multi-scan
T_{min} , T_{max}	0.8181, 0.8879
θ_{min},θ_{max}	1.514, 29.054
Limiting indices	-20≤h≤20, -20≤k≤20, -21≤l≤21
Reflections collected / unique	57733 / 14849 [R(int) = 0.0358]
Completeness to θ_{max}	92.9% (99.9 % up to θ =25.24°)
Refinement method	Full-matrix least-squares on F^2
Data / restraints / parameters	14849 /0 / 684
Goodness-of-fit on F^2	1.027
Final R indices [I>2sigma(I)]	R1=0.0415; wR2=0.0952 [12522 refl.]
R indices (all data)	R1=0.0520; wR2=0.1018
Largest diff. peak and hole	0.980 / -0.894

Table S14: Crystal data of compound 3g

Empirical formula	$3(C_{26}H_{19}F_3N_2O_4Pd)\cdot2(CH_2Cl_2)$
Formula Weight	1930.34
Temperature	150(2) K
Wavelength	0.71073 Å
Crystal system, space group	triclinic, $P\bar{1}$
Unit cell dimensions	a= 11.9947(10) Å α = 91.5730(10)°<br>b= 12.8806(11) Å β = 95.5880(10)°<br>c= 26.697(2) Å γ = 102.6590(10)°
Volume	4000.2(6) Å^3
Z	2
Absorption coefficient	0.886 mm^{-1}
F(000)	1932
Crystal Size	0.103 x 0.188 x 0.460 mm
Absorption correction	Multi-scan
T_{min} , T_{max}	0.7872, 0.8496
θ_{min},θ_{max}	0.767, 28.434
Limiting indices	-16≤h≤16, -17≤k≤16, -33≤l≤35
Reflections collected / unique	80811 / 19947 [R(int) = 0.0308]
Completeness to θ_{max}	99.0% (100 % up to θ =25.24°)
Refinement method	Full-matrix least-squares on F^2
Data / restraints / parameters	19947 /1 / 1075
Goodness-of-fit on F^2	1.021
Final R indices [I>2sigma(I)]	R1=0.0463; wR2=0.1147 [16333 refl.]
R indices (all data)	R1=0.0595; wR2=0.1233
Largest diff. peak and hole	1.884 / -1.300

Table S15: Crystal data of compound 7h

Empirical formula	$C_{41}H_{40}ClN_3O_4Pd\cdot 2(CHCl_3)$
Formula Weight	1019.34
Temperature	100 (2) K
Wavelength	0.71073 Å
Crystal system, space group	monoclinic, $P2_1/c$
Unit cell dimensions	a= 16.8457(9) Å<br>b= 19.6405(10) Å β = 107.911(2)°<br>c= 14.6458(8) Å
Volume	4610.8(4) $Å^3$
Z	4
Absorption coefficient	0.852 mm^{-1}
F(000)	2072
Crystal Size	0.030 x 0.118 x 0.136 mm
Absorption correction	Multi-scan
T_{min} , T_{max}	0.6348, 0.7457
θ_{min},θ_{max}	2.074, 28.366
Limiting indices	-22≤h≤22, -26≤k≤26, -19≤l≤19
Reflections collected / unique	180043 / 11478 [R(int) = 0.1145]
Completeness to θ_{max}	99.5%
Refinement method	Full-matrix least-squares on F^2
Data / restraints / parameters	11478 /0 / 513
Goodness-of-fit on F^2	1.082
Final R indices [I>2sigma(I)]	R1=0.0669; wR2=0.1663 [8060 refl.]
R indices (all data)	R1=0.1055; wR2=0.1942
Largest diff. peak and hole	2.185 / -1.457

Discussion of the X-ray crystal structures of orthopalladated complexes 3c, 3d, 3g and 7h (Figures S3 - S7)

The determination of the molecular structures of complexes **3c**, **3d** and **3g**, whose molecular drawns are shown in Figures S3 to S6, provides additional information. The three structures display the same arrangment of ligands around the Pd center, showing the orthopalladated oxazolone acting as a C^N-chelate, the *N*-bonded pyridine located *cis* to the palladated C atom and the monodentate O-bonded CF_3CO_2 ligand in *trans* to the same C atom. In all cases the Pd center shows a square-planar environment, only slightly distorted [sum of angles around Pd in the range: 357.2(2)°-360.2(1)°].

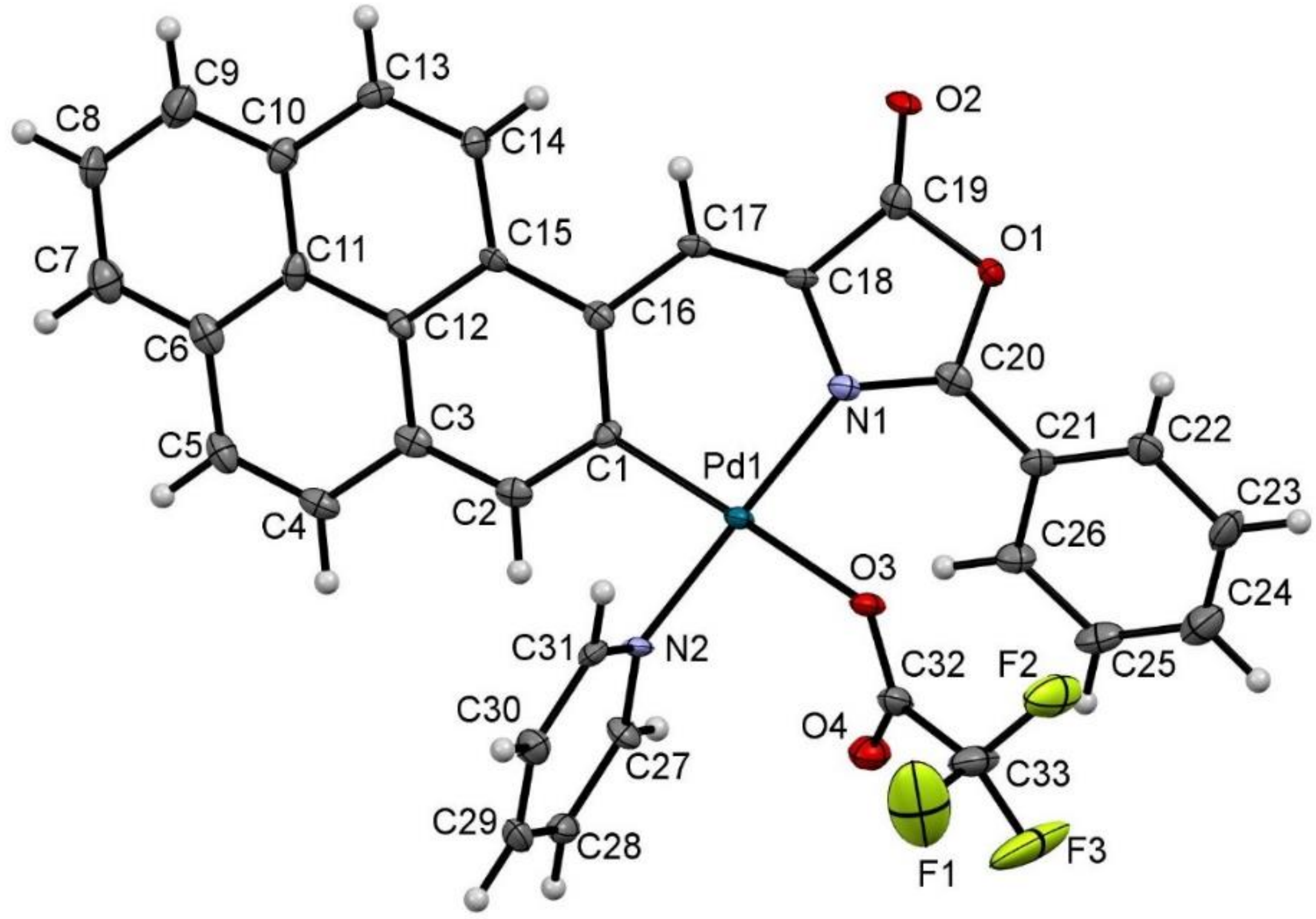

Figure S3. Molecular draw of orthopalladated **3c**. Thermal ellipsoids are drawn at 50% probability level in both figures. The H atoms have been removed in figure (b) for clarity.

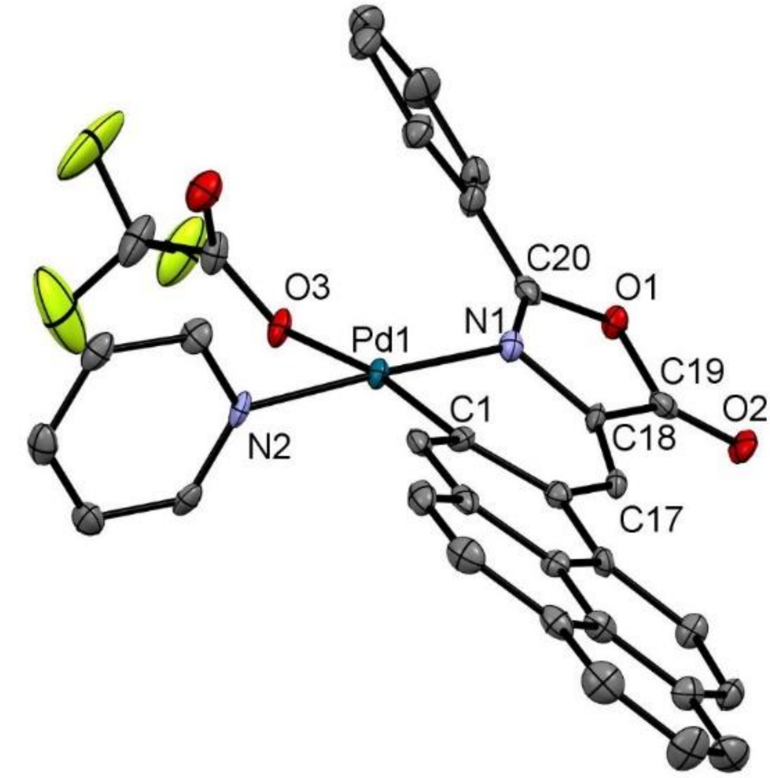

Figure S4. Molecular draw of orthopalladated **3c**, different orientation. Thermal ellipsoids are drawn at 50% probability level. The H atoms have been removed for clarity.

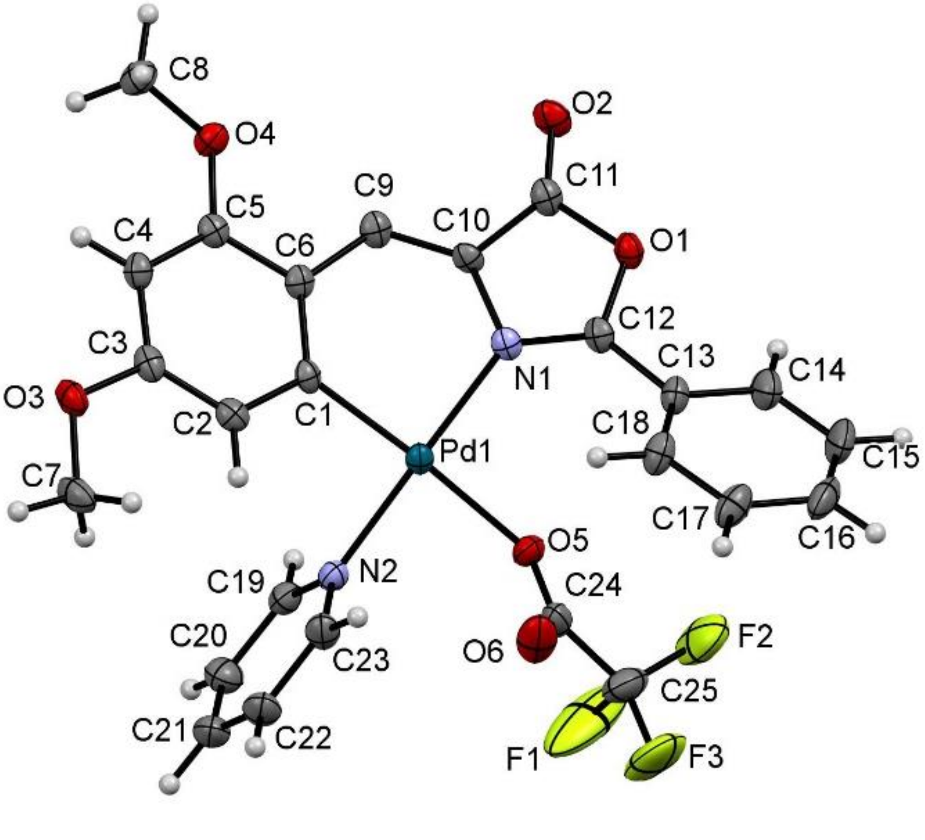

Figure S5. Molecular draw of oxazolone **3d**. Thermal ellipsoids are drawn at 50% probability level

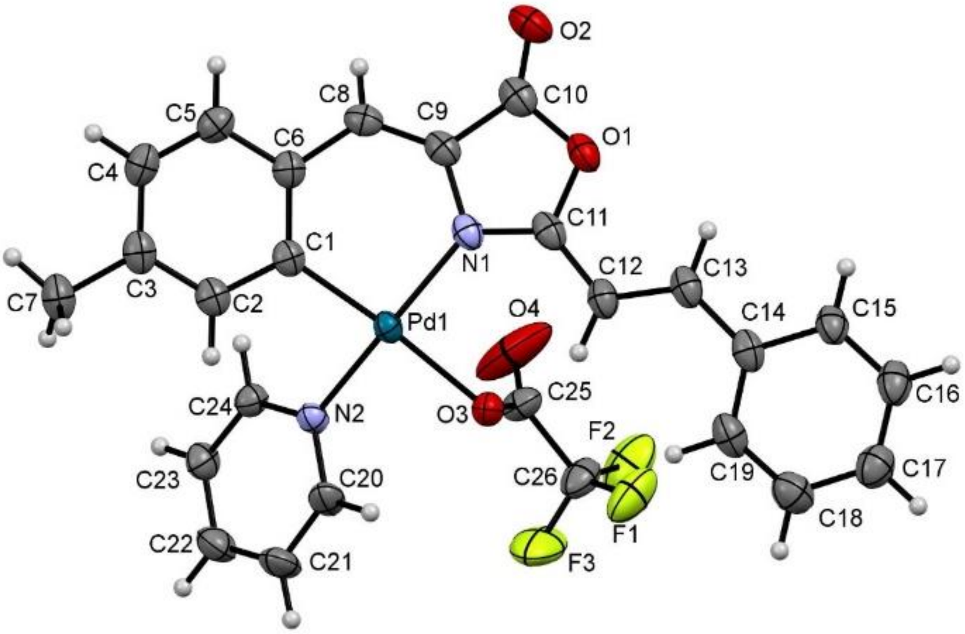

Figure S6. Molecular draw of oxazolone **3g**. Thermal ellipsoids are drawn at 50% probability level

Table S15. Comparison of bond angles (°) and bond distances (Å) of **3c**, **3d**, **3g** and related previous examples

**Plane angle (°)**	**3c**	**3d**	**ref. 19b**<br>**ref. 24**	**3g**	**ref. 19a**
1-2	33.27(3)	28.68(3)	37.16(4)<br>32.42(3)	32.94(3)	40.76(3)
1-3	32.46(3)	30.51(3)	36.48(4)<br>33.87(3)	33.10(3)	37.57(3)
1-4	55.25(3)	56.75(4)	44.16(4)<br>53.18(3)	36.30(3)	50.30(3)
2-3	10.31(3)	12.34(3)	13.93(4)<br>13.73(3)	15.81(3)	19.89(3)
2-4	21.29(3)	29.61(3)	17.86(4)<br>21.23(4)	36.09(3)	48.36(3)
3-4	26.62(3)	27.11(3)	7.80(4)<br>22.95(3)	20.83(3)	28.59(3)
Dihedral angle<br>N-C-C-C (°)	26.35(4)	-26.17(3)	11.84(4)<br>-28.56(3)		
**Bond distance**	**3c**	**3d**	**ref. 19b**	**3g**	**ref. 19a**
Pd-C1 (Å)	2.003(5)<br>2.005(5)	2.007(3)<br>1.991(3)	1.984(2)	1.997(4)<br>1.983(3)<br>1.992(3)	2.013(3)
Pd-N1 (Å)	2.036(4)<br>2.038(5)	2.042(2)<br>2.033(2)	2.044(2)	2.039(3)<br>2.017(3)<br>2.029(3)	2.006(3)
Pd-N2 (Å)	2.034(4)<br>2.030(4)	2.045(3)<br>2.049(2)		2.033(3)<br>2.036(3)<br>2.041(3)	2.060(3)
Pd-O (Å)	2.144(4)<br>2.133(4)	2.129(2)<br>2.132(2)		2.130(3)<br>2.133(3)<br>2.145(2)	

Despite the square-planar immediate environment of the Pd centers, the whole structures **3c**, **3d** and **3g** appear notably deviated from planarity. The oxazolone ligands show a strong U-shaped distortion, and are far to be coplanar with the coordination plane. These distortions can be defined through the values of the angles between the following best least-square planes: (1) the coordination plane Pd-C1-N1-N2-O; (2) the arylidene ring C1-C2-C3-C4(C12)-C5(C15)-C6(C16); (3) the central ring C1-C16(C6)-C17(C9)-C18(C10)-N1; (4) the 2-Ph ring C21-to-C26 (**3c**) or C13-to-C18 (**3d**), or the styryl fragment C9-to-C19 (**3g**). The values measured here, collected in Table S15, are compared to those found in closely related complexes previously reported by us, two of them containing a GLP-like orthopalladated oxazolone and *O,O'*-acac as a chelate ancillary ligand,[19b] or two NCMe ligands,[24] respectively, and another one having an styryl substituent at the oxazolone ring (Kaede-like) and *N,N*-bpy as the chelating auxiliary ligand.[19a]

For complexes **3c** and **3d**, which show a GLP-like oxazolone (2-Ph as substituent), the largest deviation from ideal planarity is found in the angle formed between the molecular plane (1) and the 2-Ph ring of the oxazolone (4), which results from the minimization of the steric interactions between the ligand in *cis* to the N-oxazolone and the mentioned Ph ring. Therefore, the smallest value corresponds to the less sterically demanding acac derivative [44.16(4)°],[19b] while for the more bulky O_2CCF_3 (**3c**, **3d**) or NCMe ligands[24] the angle reach values in the range 53-57°, showing very similar distortions despite the presence of different ligands. For complex **3g**, the *trans* arrangement of the C=C bond of the styryl group points outwards the Ph ring and leaves a much less congested arrangement.[19a] In comparison with previous examples, the smaller steric requirements of the monodentate CF_3CO_2 and pyridine ligands in **3g** with respect to N,N-chelating bipyridine[19a] are reflected in smaller values of the angles between planes (Table S15). Therefore, it is possible to see a correlation between the size of the Pd-ancillary ligands and the distortion of the oxazolone, which deviates notably from planarity.

Concerning bond distances, the Pd-C1 and Pd-N1 bond distances appear in the ranges 1.983(3)-2.007(3) Å and 2.017(3)-2.044(2) Å, respectively, which are identical within experimental error to the respective Pd-C and Pd-N bond distances found in other closely related examples (Table S15).[19a,b] The Pd-N (pyridine) bond distances are also identical, within experimental error, to those found in the bibliography for this type of bond.[25] The Pd-O bond distances measured for the terminal CF_3CO_2 ligand (range 2.129(2)-2.145(2)Å) fall as well in the usual range of distances found when this ligand is *trans* to an orthopalladated C atom.[26] This value, however, is clearly longer than those measured

for this bond in Pd complexes having *trans* O-, N- or S-donors.[27] This elongation reflects the high *trans* influence of the C atom and is related with the dynamic behaviour of this ligand observed in the ^{13}C NMR spectra of complexes **3**.

Attempts to grow crystals of **6h**, containing a bulky NHC ligand and CF_3CO_2 as ancillary ligands, gave always a precipitated solid of **6h** and crops of few crystals which showed to be corresponding to complex **7h**, which still have the NHC ligand but shows a chloride instead of the trifluoroacetate ligand (Figure S7). This metathesis of ligands is probably related with the fact that the crystallization solvent was $CHCl_3$, which can contain small amounts of HCl.

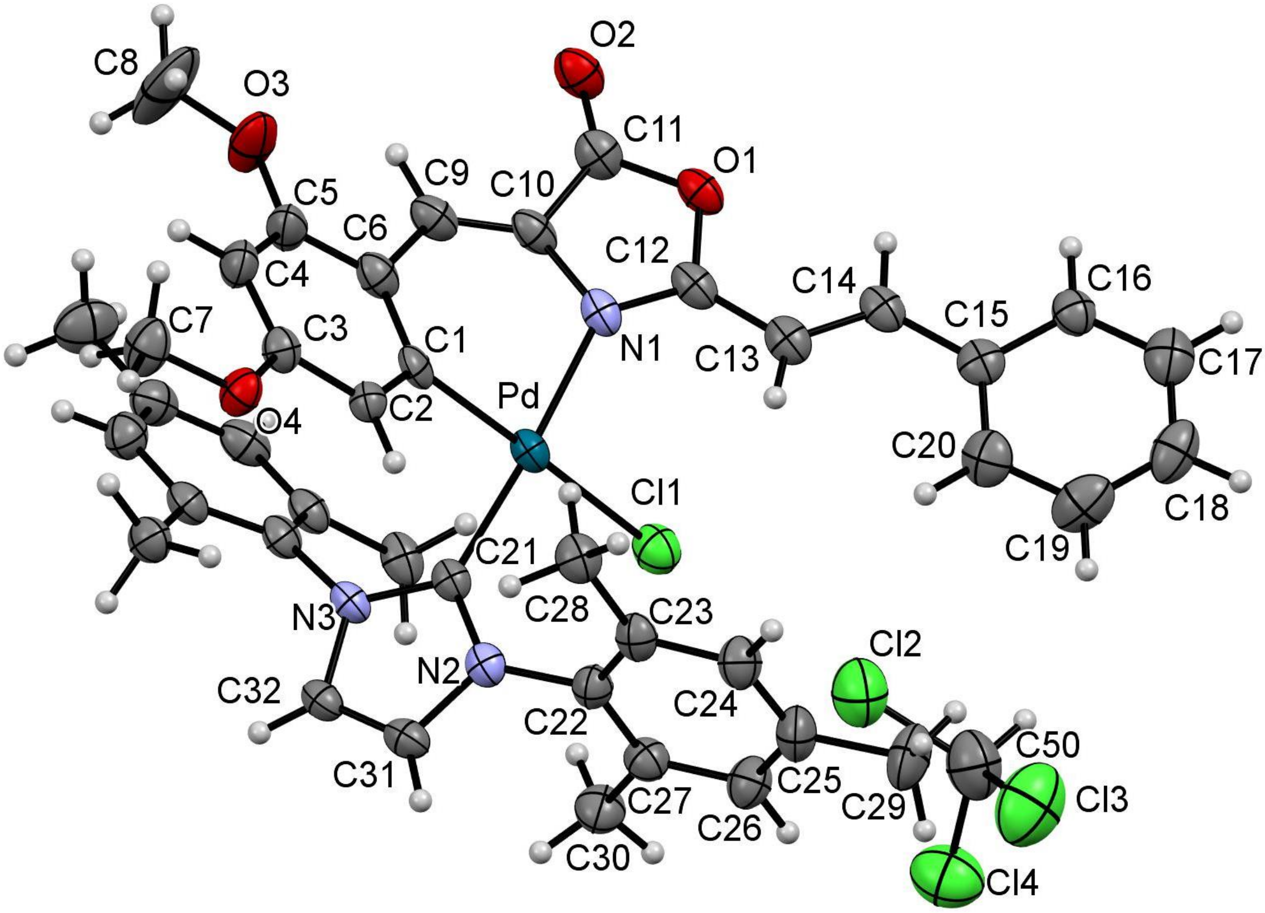

Figure S7. Molecular draw of oxazolone **7h**. Thermal ellipsoids are drawn at 50% probability level

The distortion of the oxazolone ligand in **7h** is intermediate between that found in **3g** and that in previous bipy-complexes,[19a] as it can be deduced from the values of the angles between the same planes defined in Table S15: 1-2 = 40.54(3)°, 1-3 = 39.75(3)°, 1-4 = 40.01(3)°, 2-3 = 19.25(3)°, 2-4 = 35.66(3)° and 3-4 = 16.90(3)°. This is surprising, considering the very large steric requirements of the NHC ligand. Probably the small size of the *cis* chloride ligand helps to counterbalance this situation and to decrease intramolecular repulsions. In addition, the bonding of the NHC ligand merits a more detailed analysis. The best least-square plane containing the NHC ligand (C21-N2-N3-C31-C32) is not

perpendicular to the coordination plane, the angle between these two planes being 57.37(3)°. This rotation around the Pd1-C21 bond seems to be related to the establishment of an intramolecular π-π stacking between the orthopalladated ring (C1-C2-C3-C4-C5-C6) and one of the mesityl rings (C33-C34-C35-C36-C37-C38). This π-π stacking is characterized by the distance between the centroids of the respective rings, which results to be 3.744(4) Å,[7] indicating a strong π-π interaction which contributes to stabilize the resulting molecule.[28,29] As a summary, the molecule can accomodate a bulky ligand establishing intramolecular stabilizing interactions, resulting in a lower distortion of the oxazolone fragment.

References corresponding to the Supporting Information

1 (a) Plöchl, J. Ueber Phenylglycidasäure (Phenyloxacrylsäure), *Chem. Ber.* **1883**, *16*, 2815; (b) Plöchl, J. Ueber einige Derivate der Benzoylimidozimmtsäure, *Chem. Ber.* **1884**, *17*, 1623; (c) Erlenmeyer, E. Ueber die Condensation der Hippursäure mit Phtalsäureanhydrid und mit Benzaldehyd, *Justus Liebigs Annalen der Chemie* **1893**, *275*, 1; (d) Carter, H. E. *Azlactones*, Chapter 5 of the book series *Organic Reactions* **1946**, *3*, 198; (e) Filler, R. *Advances in Heterocyclic Chemistry*, A. R. Katrizky, Editor, Academic Press, New York, 1954, ch. 4, p. 75; (f) Rao, Y. S.; Filler, R. Geometric Isomers of 2-Aryl(Aralkyl)-4-arylidene(alkylidene)-5(4*H*)-oxazolones, *Synthesis* **1975**, *12*, 749; (g) Cativiela, C.; Díaz de Villegas, M. D.; Meléndez, E. On the synthesis of geometric isomers of 2-methyl (or phenyl)-4-[α-arylethylidene]-5(4*H*)-oxazolones, *J. Heterocycl. Chem.* **1985**, *22*, 1655; (h) Bautista, F. M.; Campelo, J. M.; García, A.; Luna, D.; Marinas, J. M.; Romero, A. A. Study on dry-media microwave azlactone synthesis on different supported KF catalysts: influence of textural and acid–base properties of supports. *J. Chem. Soc., Perkin Trans 2*, **2002**, 227; (i) Arenal, I.; Bernabe, M.; Fernández-Alvarez, E. *Anales de Química, Serie C: Química Orgánica y Bioquímica* **1981**, *77*, 56. (j) Rao, Y. S.; Filler, R. Oxazoles, In *The Chemistry of Heterocyclic Compounds*, Vol. 45; Turchi, I. J. Editor, John Wiley & Sons, Inc. New York, 1986, ch. 3, pp. 363-691.

2 Kim, Y.; Ko, Y. H.; Jung, M.; Selvapalam, N.; Kim, K. A new photo-switchable “on-off” host–guest system. *Photochem. Photo-biol. Sci.* **2011**, *10*, 1415-1419.

3 SAINT; Version 5.0 ed.; Bruker Analytical X-Ray Systems: Madison, WI, 1998.

4 Sheldrick, G. M. SADABS, Program for absorption and other corrections, Göttingen University, 1996.

5 Sheldrick, G. M. SHELXT − Integrated Space-Group and Crystal- Structure Determination, *Acta Crystallogr., Sect. A: Found. Adv.* **2015**, *A71*, 3−8.

6 Sheldrick, G. M. Crystal structure refinement with SHELXL. *Acta Crystallogr., Sect. C: Struct. Chem.* **2015**, *C71*, 3–8.

7 Farrugia, L. J. WinGX and ORTEP for Windows: an update. *J. Appl. Crystallogr.* **2012**, *45*, 849-854.

8 (a) Hohenberg, P.; Kohn, W. Inhomogeneous electron gas, *Phys. Rev.* **1964,** *136*, B864. (b) Kohn, W.; Sham, L. J. Self-Consistent Equations Including Exchange and Correlation Effects, *Phys. Rev.* **1965,** *140*, A1133.

9 *Gaussian 16, Revision C.01*. Frisch, M. J.; Trucks, G. W.; Schlegel, H. B.; Scuseria, G. E.; Robb, M. A.; Cheeseman, J. R.; Scalmani, G.; Barone, V.; Petersson, G. A.; Nakatsuji, H.; Li, X.; Caricato, M.; Marenich, A. V.; Bloino, J.; Janesko, B. G.; Gomperts, R.; Mennucci, B.; Hratchian, H. P.; Ortiz, J. V.; Izmaylov, A. F.; Sonnenberg, J. L.; Williams-Young, D.; Ding, F.; Lipparini, F.; Egidi, F.; Goings, J.; Peng, B.; Petrone, A.; Henderson, T.; Ranasinghe, D.; Zakrzewski, V. G.; Gao, J.; Rega, N.; Zheng, G.; Liang, W.; Hada, M.; Ehara, M.; Toyota, K.; Fukuda, R.; Hasegawa, J.; Ishida, M.; Nakajima, T.; Honda, Y.; Kitao, O.; Nakai, H.; Vreven, T.; Throssell, K.; Montgomery Jr., J. A.; Peralta, J. E.; Ogliaro, F.; Bearpark, M. J.; Heyd, J. J.; Brothers, E. N.; Kudin, K. N.; Staroverov, V. N.; Keith, T. A.; Kobayashi, R.; Normand, J.; Raghavachari, K.; Rendell, A. P.; Burant, J. C.; Iyengar, S. S.; Tomasi, J.; Cossi, M.; Millam, J. M.; Klene, M.; Adamo, C.; Cammi, R.; Ochterski, J. W.; Martin, R. L.; Morokuma, K.; Farkas, O.; Foresman, J. B.; Fox, D. J. Gaussian, Inc., Wallingford CT, 2016.

10 Chai, J.-D.; Head-Gordon, M. Long-range corrected hybrid density functionals with damped atom-atom dispersion corrections, *Phys. Chem. Chem. Phys*., **2008**, *10*, 6615.

11 Zhao, Y.; Truhlar, D. G. The M06 suite of density functionals for main group thermochemistry, thermochemical kinetics, noncovalent interactions, excited states, and transition elements: two new functionals and systematic testing of four M06-class functionals and 12 other functionals, *Theor. Chem. Acc.*, **2008**, *120*, 215.

12 (a) Hehre, W. J.; Ditchfield, R.; Pople, J. A. Self—Consistent Molecular Orbital Methods. XII. Further Extensions of Gaussian—Type Basis Sets for Use in Molecular Orbital Studies of Organic Molecules, *J. Chem. Phys*. **1972**, *56*, 2257. (b) Hariharan, P. C.; Pople, J. A. The influence of polarization functions on molecular orbital hydrogenation energies, *Theor. Chem. Acc.* **1973**, *28*, 213. (c) Francl, M. M.; Pietro, W. J.; Hehre, W. J.; Binkley, J. S.; DeFrees, D. J.; Pople, J. A.; Gordon, M. S. Self-Consistent Molecular Orbital Methods. XXIII. A Polarization- Type Basis Set for Second-Row Elements, *J. Chem. Phys.* **1982**, *77*, 3654.

13 (a) Dolg, M.; Wedig, U.; Stoll, H.; Preuss, H. Energy-adjusted ab initio pseudopotentials for the first row transition elements, *J. Chem. Phys.* **1987**, *86*, 866. (b) Martin, J. M. L.; Sundermann, A. Correlation consistent valence

basis sets for use with the Stuttgart–Dresden–Bonn relativistic effective core potentials: The atoms Ga–Kr and In–Xe, *J. Chem. Phys.* **2001**, *114*, 3408.

14 (a) Scalmani, G.; Frisch, M. J. Continuous surface charge polarizable continuum models of solvation. I. General formalism, *J. Chem. Phys.* **2010,** *132*, 114110. (b) Tomasi, J.; Mennucci, B.; Cammi, R. Quantum mechanical continuum solvation models, *Chem. Rev.* **2005**, *105*, 2999. (c) Caricato, M. Absorption and Emission Spectra of Solvated Molecules with the EOM-CCSD-PCM Method, *J. Chem. Theory & Comput.* **2012**, *8*, 4494.

15 (a) Runge, E.; Gross, E. K. U. Density-Functional Theory for Time-Dependent Systems, *Phys. Rev. Lett*. **1984**, *52*, 997. (b) Bauernschmitt, R.; Ahlrichs, R. Treatment of electronic excitations within the adiabatic approximation of time dependent density functional theory, *Chem. Phys. Lett.* **1996**, *256*, 454. (b) Casida, M. E.; Jamorski, C.; Casida, K. C.; Salahub, D. R. Molecular excitation energies to high-lying bound states from time-dependent density-functional response theory: Characterization and correction of the time-dependent local density approximation ionization threshold. *J. Chem. Phys.* **1998**, *108*, 4439. (c) Stratmann, R. E.; Scuseria, G. E.; Frisch, M. J. An efficient implementation of time-dependent density-functional theory for the calculation of excitation energies of large molecules, *J. Chem. Phys.* **1998**, *109*, 8218. (d) Scalmani, G.; Frisch, M. J.; Mennucci, B.; Tomasi, J.; Cammi, R.; Barone, V. Geometries and properties of excited states in the gas phase and in solution: Theory and application of a time-dependent density functional theory polarizable continuum model, *J. Chem. Phys.* **2006**, *124*, 094107. (e) Liu, J.; Liang, W. Analytical approach for the excited-state Hessian in time-dependent density functional theory: formalism, implementation and performance. *J. Chem. Phys.* **2011**, *135*, 184111. (f) Adamo, F.; Jacquemin, D. The calculations of excited-state properties with Time-Dependent Density Functional Theory, *Chem. Soc. Rev.* **2013**, *42*, 845.

16 The PyMOL Molecular Graphics System, version 2.5.4, Schrödinger, LLC.

17 https://gist.github.com/bobbypaton.

18 (a) Shimanskaya, N. P.; Lysova, I. V.; Kotok, L. A.; Afanasiadi, L. Polarography of 4-substituted derivatives of 5-oxazolone, *Zhurnal Obshchei Khimii* **1978**, *48*, 2315-2319. (b) Jensen, K. A.; Christensen, S. A. K. Researches on Plant-Growth Substances. II. On 1-Naphthylacetaldehyde, *Acta Chemica Scandinavica* **1950**, *4*, 703-709. (c) Lettré, H.; Buchholz, K.; Fernholz, M.-E. Chemically labelled antigens. III. Introduction of 4-ring systems into proteins, *Z. Physiol. Chem.* **1940**, *267*, 108-114. (d) Deulofeu, V. Amino acids. V. A modification of the reduction of benzoylaminoacrylic acids in the Erlenmeyer synthesis, *Anales de la Real Sociedad Espanola de Fisica y Quimica* **1934**, *32*, 152. (e) Hayashida, S.; Taya, M. Morita, M. Heat-resistant composition, active optical waveguide, and its manufacture. Patent *Jpn. Kokai*

Tokkyo Koho 1996, JP 08041331 A 19960213. (f) Mustafa, A.; Asker, W.; Harhash, A. H.; Abdin, T. M. S.; Zayed, E. M. Reactions with 2,4-disubstituted 2-oxazolin-5-ones. *Justus Liebigs Ann. Chem.* **1968**, *714*, 146-154.

19 (a) Laga, E.; Dalmau, D.; Arregui, S.; Crespo, O.; Jiménez, A. I.; Pop, A.; Silvestru, C.; Urriolabeitia, E. P. Fluorescent Orthopalladated Complexes of 4-Aryliden-5(4*H*)-oxazolones from the Kaede Protein: Synthesis and Characterization, *Molecules* **2021**, *26*, 1238. (b) Garcia-Sanz, C.; Andreu, A.; de las Rivas, B.; Jimenez, A. I.; Pop, A.; Silvestru, C.; Urriolabeitia, E. P.; Palomo, J. M. Pd-Oxazolone Complexes Conjugated to an Engineered Enzyme: Improving Fluorescence and Catalytic Properties, *Org. Biomol. Chem.* **2021**, *19*, 2773.

20 Jiang, M.; He, Z.; Zhang, Y.; Sung, H. H. Y.; Lam, J. W. Y.; Peng, Q.; Yan, Y.; Wong, K. S.; Williams, I. D.; Zhao, Y.; Tang, B. Z. Development of benzylidene-methyloxazolone based AIEgens and decipherment of their working mechanism, *J. Mater. Chem. C* **2017**, *5*, 7191.

21 Chatterjee, T.; Mandal, M.; Gude, V.; Bag, P. P. Mandal, P. K. Strong electron donation induced differential nonradiative decay pathways for *para* and *meta* GFP chromophore analogues, *Phys. Chem. Chem. Phys.* **2015**, *17*, 20515-20521.

22 (a) Blanco-Lomas, M.; Campos, P. J.; Sampedro, D. Benzylidene-Oxazolones as Molecular Photoswitches, *Org.Lett.* **2012**, *14*, 4334. (b) Asiri, A. M.; Ng, S. W. (*E*)-4-(2,5-Dimethoxy-benzylidene)-2-phenyl-1,3-oxazol-5(4*H*)-one, *Acta Crystallogr., Sect. E: Struct. Rep. Online* **2009**, *65*, o1746. (c) Asiri, A. M.; Akkurt, M.; Khan, I. U.; Arshad, M. N. 4-(2-Methoxy-benzylidene)-2-phenyl-1,3-oxazol-5(4*H*)-one, *Acta Crystallogr., Sect. E: Struct. Rep. Online* **2009**, *65*, o842.

23 Bondi, A. van der Waals Volumes and Radii, *J. Phys. Chem.* **1964**, *68*, 441.

24 Roiban, G.-D.; Serrano, E.; Soler, T.; Contel, M.; Grosu, I.; Cativiela, C.; Urriolabeitia, E. P. Ortho-Palladation of (*Z*)-2-Aryl-4-Arylidene-5(4*H*)-Oxazolones. Structure and Functionalization, *Organometallics* **2010**, *29*, 1428.

25 Guy Orpen, A.; Brammer, L.; Allen, F. H.; Kennard, O.; Watson, D. G.; Taylor, R. Supplement. Tables of bond lengths determined by X-ray and neutron diffraction. Part 2. Organometallic compounds and co-ordination complexes of the d- and f-block metals. *J. Chem. Soc. Dalton Trans.* **1989**, S1.

26 (a) Chartoire, A.; Lesieur, M.; Slawin, A. M. Z.; Nolan, S. P.; Cazin, C. S. J. Highly Active Well-Defined Palladium Precatalysts for the Efficient Amination of Aryl Chlorides, *Organometallics* **2011**, *30*, 4432. (b) Dudkina, Y. B.;

Mikhaylov, D. Y.; Gryaznova, T. V.; Tufatullin, A. I.; Kataeva, O. N.; Vicic, D. A.; Budnikova, Y. H. Electrochemical Ortho Functionalization of 2-Phenylpyridine with Perfluorocarboxylic Acids Catalyzed by Palladium in Higher Oxidation States, *Organometallics* **2013**, *32*, 4785. (c) Bedford, R. B.; Cazin, C. S. J.; Coles, S.; Gelbrich, T.; Hursthouse, M. B.; Scordia, V. J. M. Phosphine and arsine adducts of N-donor palladacycles as catalysts in the Suzuki coupling of aryl bromides, *Dalton Trans*. **2003**, 3350. (d) Bergbreiter, D. E.; Frels, J. D.; Rawson, J.; Li, J.; Reibenspies, J. H. Synthesis and characterization of electronically varied XCX palladacycles with functional arene groups, *Inorg. Chim. Acta* **2006**, *359*, 1912; (e) Benito-Garagorri, D.; Bocokic, V.; Mereiter, K.*;* Kirchner, K. A Modular Approach to Achiral and Chiral Nickel(II), Palladium(II), and Platinum(II) PCP Pincer Complexes Based on Diaminobenzenes. *Organometallics* **2006**, *25*, 3817. (f) Bedford, R. B.; Cazin, C. S. J.; Coles, S. J.; Gelbrich, T.; Horton, P. N.; Hursthouse, M. B.; Light, M. E. High-Activity Catalysts for Suzuki Coupling and Amination Reactions with Deactivated Aryl Chloride Substrates: Importance of the Palladium Source, *Organometallics* **2003**, *22*, 987.

27 (a) Efimenko, I. A.; Churakov, A. V.; Erofeeva, O. S.; Ivanova N. A.; Demina, L. I. Effect of the Nature of Haloacetic Acids on the Type of Morpholine Complexes Formed. Crystal Structure of the First Palladium Tetracarboxylate with Monocarboxylic Acid: Morpholinium Tetrakis(trifluoroacetato)palladate(II), $(O(CH_2CH_2)_2NH_2)_2[Pd(CF_3COO)_4]$. *Russ. J. Coord. Chem.* **2019**, *45*, 615. (b) Izawa, Y.; Stahl, S. S. Aerobic Oxidative Coupling of o-Xylene: Discovery of 2-Fluoropyridine as a Ligand to Support Selective Pd-Catalyzed C-H Functionalization, *Adv. Synth. Catal.* **2010**, *352*, 3223. (c) Kumar, A.; Naaz, A.; Prakasham, A. P.; Gangwar, M. K.; Butcher, R. J.; Panda, D.; Ghosh, P. Potent Anticancer Activity with High Selectivity of a Chiral Palladium N-Heterocyclic Carbene Complex, *ACS Omega* **2017**, *2*, 4632. (d) White, P. B.; Jaworski, J. N.; Fry, C. G.; Dolinar, B. S.; Guzei, I.A.; Stahl, S.S. Structurally Diverse Diazafluorene-Ligated Palladium(II) Complexes and Their Implications for Aerobic Oxidation Reactions, *J. Am. Chem. Soc.* **2016**, *138*, 4869. (e) Diao, T.; White, P.; Guzei, I.; Stahl, S. S. Characterization of DMSO Coordination to Palladium(II) in Solution and Insights into the Aerobic Oxidation Catalys, $Pd(DMSO)_2(TFA)_2$, *Inorg. Chem.* **2012**, *51*, 11898.

28 (a) Martinez, C. R.; Iverson, B. L. Rethinking the term "pi-stacking". *Chem. Sci.* **2012**, *3*, 2191. (b) Dance, I.; Scudder, M. Molecules embracing in crystals. *CrystEngComm* **2009**, *11*, 2233. (c) Janiak, C. A critical account on π–π stacking in metal complexes with aromatic nitrogen-containing ligands. *J. Chem. Soc., Dalton Trans.* **2000**, 3885. (d) Meyer, E. A.; Castellano, R. K.; Diederich, F. Interactions with Aromatic Rings in Chemical and Biological Recognition,

Angew. Chem. Int. Ed. **2003**, *42*, 1210. (e) Chen, T.; Li, M.; Liu, J. π−π Stacking Interaction: A Nondestructive and Facile Means in Material Engineering for Bioapplications, *Cryst. Growth Des.* **2018**, *18*, 2765.

29 (a) Deng, J. H.; Luo, J.; Mao, Y. L.; Lai, S.; Gong, Y. N.; Zhong, D. C.; Lu, T. B. π-π stacking interactions: Non-negligible forces for stabilizing porous supramolecular frameworks, *Sci. Adv.* **2020**, *6*, eaax9976. (b) Riwar, L. J.; Trapp, N.; Kuhn, B.; Diederich, F. Substituent Effects in Parallel-Displaced π–π Stacking Interactions: Distance Matters. *Angew. Chem. Int. Ed.* **2017**, *56*, 11252.
